# Stereoselective Synthesis of Chiral *C*_2_-Symmetric 1,3- and 1,5-Bis-Sulfoxides Guided by the Horeau Principle: Understanding the Influence of the Carbon Chain Nature in Its Ability for Metal Coordination

**DOI:** 10.1021/acs.joc.4c01729

**Published:** 2024-10-02

**Authors:** Nazaret Moreno-Rodríguez, L. Alberto Prieto, Victoria Valdivia, Rocío Recio, Inmaculada Fernández

**Affiliations:** Departamento de Química Orgánica y Farmacéutica, Facultad de Farmacia, Universidad de Sevilla, C/Profesor García González, 2, 41012 Sevilla, Spain

## Abstract

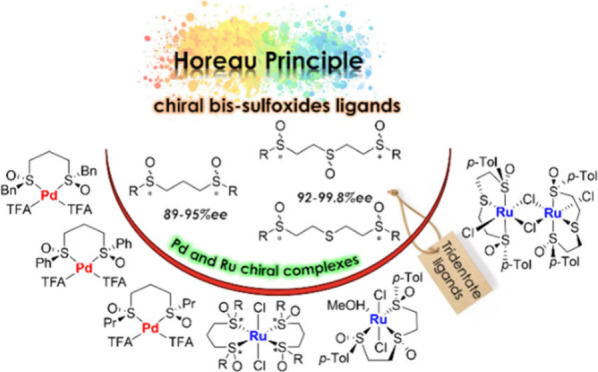

The stereoselective synthesis of two distinct types of *C*_2_-symmetric chiral bis-sulfoxides, 1,3- and 1,5-bis(sulfinyl) derivatives, has been achieved based on the DAG methodology. The 1,5-bis(sulfinyl) derivatives constitute a new family of tridentate chiral ligands thanks to the presence of an additional sulfenyl or sulfinyl group in the carbon chain acting as a bridge. A systematic development and optimization of two synthetic routes, one for each ligand family, have been undertaken, highlighting the strategic utilization of Horeau’s law to enhance enantioselectivity. Additionally, palladium (Pd) and ruthenium (Ru) complexes derived from the synthesized bis-sulfoxides were prepared, and their structures were elucidated through spectroscopic analysis. Isolation of Pd(II) complexes involving 1,3-bis-sulfoxides was exclusively achieved using trifluoroacetates as coligands. In the case of Ru(II) complexes, the trans geometry could be determined for 1,3-bis-sulfoxides. The introduction of a third sulfur atom as a coordinating element in the 1,5-bis(sulfinyl) derivatives facilitates the formation of two distinct tricoordinated Ru(II) complexes. The structure of these complexes is intricately influenced by the oxidation state adopted by the central sulfur on the chain, whether as a thioether or as a sulfoxide.

## Introduction

Since 1972, when Kagan introduced the chiral ligand DIOP with *C*_2_ symmetry as a precursor for an effective chiral catalyst in asymmetric synthesis, significant progress has been made in the field of catalysis.^1^ Bidentate ligands with *C*_2_ symmetry have garnered more attention than their monodentate counterparts owing to the ease of obtaining both enantiomeric forms from readily available commercial precursors and the inherent advantages that they offer in asymmetric catalysis. By incorporating a *C*_2_ symmetry axis in the chiral auxiliary, the number of potential competing diastereomeric transition states is significantly reduced. This reduction in complexity not only streamlines the synthesis process but also enhances the efficiency and selectivity of the catalytic reactions, making these ligands invaluable tools in the realm of asymmetric synthesis. Over the years, numerous structural modifications have been proposed to design new catalysts with *C*_2_ symmetry. These modifications have not only expanded our understanding of asymmetric synthesis but also paved the way for the execution of diverse catalytic processes with remarkable enantioselectivity.

**Figure 1:**
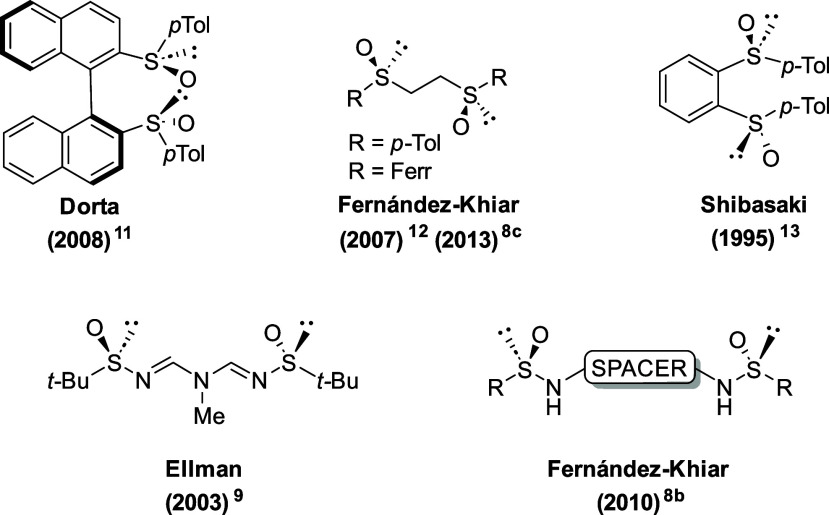
Some representative examples of *C*_2_-symmetric sulfinyl bidentate ligands described in the literature.^8−13^

Various families of bidentate ligands exist, categorized based on the heteroatoms coordinating with metals. Examples include phosphorus-based ligands such as DIOP,^1^ Chiraphos,^2^ Dipamp,^3^ or Binap,^4^ oxygen-based ligands like BINOL or TADDOL and its derivatives,^5^*N*,*N*-ligands like Box- or Salen-type ligands,^6^ and sulfur-derived ligands including thioethers,^7^ sulfoxides,^8^ and sulfinamides.^8b,9^ Among the sulfur-derived ligands, chiral *C*_2_-symmetric bis-sulfoxides and bis-sulfinamides have gained prominence in organic and organometallic catalysis due to their demonstrated coordination ability.^10^ The specific configuration on sulfur places the achiral environment near the metal coordination sphere. Several representative *C*_2_-symmetric sulfinyl ligands are shown in Figure 1. Notably, besides their efficacy, chiral bidentate SO/SO ligands offer stability in the presence of air, oxygen, and humidity at room temperature, and they can be easily purified without degradation.

Even if directly oxidizing a bis-thioether appears to be the simplest method for synthesizing the corresponding bis-sulfoxide,^14^ currently there are no widely applicable techniques to achieve this oxidation in an enantioselective manner. Fortunately, the nucleophilic addition of an organometallic reagent to a compound containing two well-defined chiral electrophilic sulfur atoms offers a universally applicable technique for synthesizing enantiopure *C*_2_-symmetric bis-sulfoxides. One noteworthy example is diaceton-d-glucose 1,2-bis-sulfinate esters, pioneered by our research group in the first decade of the 21st century.^15^ This approach allows for the enantioselective synthesis of *C*_2_-symmetric 1,2-bis(alkyl- or arylsulfinyl)ethanes. Ligands synthesized using this method have found application in various organic and organometallic catalytic processes, consistently delivering exceptional results.^8,12^ Lastly, the dimerization of a chiral sulfoxide stands out as a potent method for synthesizing enantiopure bis-sulfoxides, where the optical purity and sulfur chirality are predetermined by those of the precursor monomers. A notable example is the copper-catalyzed dimerization of enantiopure lithiomethylsulfoxides, providing access to the corresponding enantiopure 1,2-bis-sulfoxides with high yields.^15^

The discovery of ruthenium complexes of sulfinyl derivatives with potential applications in cancer treatment and bioorganic chemistry intensified the interest for enantiopure *C*_2_-symmetric 1,2-bis-sulfoxides.^16^ Previous studies on racemic 1,2-bis-sulfoxides have shown promising results in bioinorganic chemistry for their potential anticancer activity.^16^ However, the lack of suitable methods for enantioselective synthesis of chiral 1,2-bis-sulfoxides has often restricted the study of their Ru complexes to achiral *meso* derivatives. On the other hand, the C–H activation reaction catalyzed by palladium complexes derived from 1,2-bis(benzylsulfinyl)ethane as a ligand has been used for diverse reactions such as alkylation,^17^ allylic oxidation,^18^ and amination,^19^ showcasing its versatility. However, in this case too, only the racemic version of these processes has been described so far using the *meso* form of the ligand due to challenges in preparing enantiopure derivatives.

Considering the interest in these metal complexes, we decided to focus on the study of Pd(II) and Ru(II) complexes of enantiopure *C*_2_-symmetric bis-sulfoxides. Otherwise, given the significant influence that the length and nature of the connecting chain between the heteroatoms, as anchoring points to the metal, can have on the coordination capability and catalytic activity of the metal complex, we have focused on developing appropriate methodologies for synthesizing two distinct types of enantiopure *C*_2_-symmetric bis-sulfinyl ligands. In both cases, the effectiveness of the Horeau principle decisively influences the optical purity of the final bis-sulfoxides.^20^

First, 1,3-bis(sulfinyl)propanes are explored as superior homologues of 1,2-bis(sulfinyl)ethanes.^15a^ Second, bis(alkylsulfinylethyl)thioethers are investigated, aiming to understand not only the impact of chain length but also the presence of an additional sulfur atom within the chain as a third coordination point with the metal.

## Results and Discussion

### Asymmetric Synthesis of *C*_2_-Symmetric 1,3-Bis(Sulfinyl)propanes

A broadening of the diacetone-d-glucose methodology (DAG methodology), previously developed for synthesizing 1,2-bis(sulfinyl)ethanes,^15a^ would allow us to create enantiopure 1,*n*-bis-sulfoxides (*n* ≥ 2), where the carbon chain’s length is determined by the corresponding 1,*n*-bis(sulfinyl) chloride used as starting material. Thus, to synthesize 1,3-bis-sulfinylpropanes, we focused on preparing the corresponding 1,3-bis(sulfinyl)propane dichloride.

**Scheme 1:**
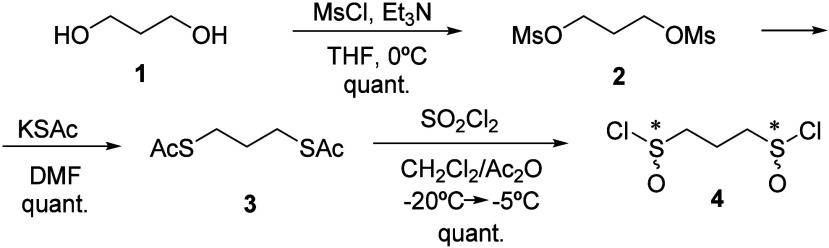
Synthesis of Dichloride **4** from Propane-1,3-diol

This dichloride can be synthesized from propane-1,3-dithiol through a two-step process involving chlorine treatment.^15a^ Despite the high yield of this reaction, the off-putting odor of the starting material and the practical challenges associated with handling chlorine as a reagent led us to explore an alternative three-step pathway with propane-1,3-diol as the starting material (Scheme 1).

**Scheme 2:**
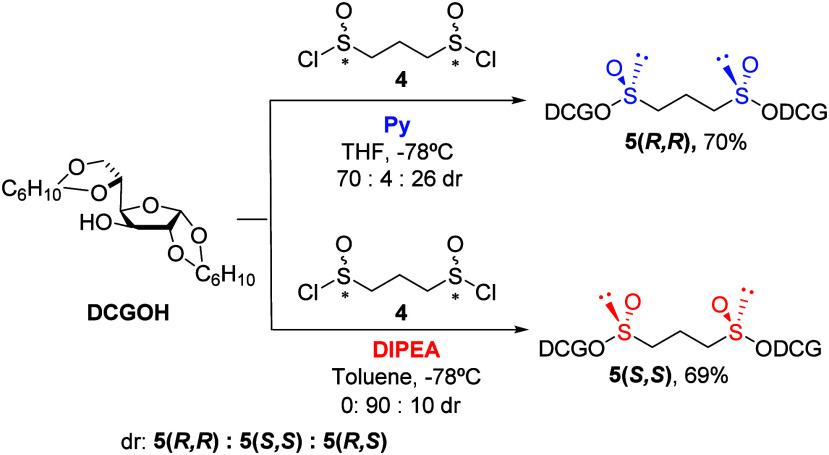
Synthesis of Propane-1,3-bis(sulfinates) by Stereodivergent Dynamic Kinetic Resolution of Propane-1,3-bis(sulfinyl) Chloride

Propane-1,3-diol was converted into dimesylated **2** with excellent yield. Subsequently, the mesyl groups were substituted with thioacetate and then oxidized to sulfinyl chlorides using sulfuryl chloride and acetic anhydride in dichloromethane. This process resulted in the formation of a high-purity pale pink oil, propane-1,3-bis(sulfinyl) chloride **4**, with a high yield. Treating **4** with a secondary chiral alcohol produced the corresponding 1,3-bis-sulfinate, generating two chiral centers on sulfur. The diastereoselectivity and stereochemical outcome of the reaction were determined by the base present in the reaction medium. Diacetone-d-glucose (DAGOH) and dicyclohexylidene-d-glucose (DCGOH) were shown to be the best chiral alcohols, with DCGOH chosen for the stability of the corresponding diastereomeric bis-sulfinate esters (Scheme 2) as the direct precursors for propane-1,3-bis-sulfoxides.

The analysis of the ^1^H NMR spectrum of the crude mixture in C_6_D_6_ showed full conversion and, by analogy with the results previously obtained with 1,2-bis-sulfinates,^15^ enabled the determination of the stereoisomeric ratio obtained in each case, demonstrating a high degree of diastereoselectivity (see Supporting Information). When pyridine was used as base, the (*R*,*R*)-bis-sulfinate ester **5(*R*,*R*)** emerged as the major product within a mixture of the three possible stereoisomers, two exhibit *C*_2_ symmetry **5(*R*,*R*)** and **5(*S*,*S*)** and the non-*C*_2_-symmetric **5(*R*,*S*)**, in a 70:4:26 ratio, respectively.

Changing the amine, from the aromatic pyridine to the aliphatic and sterically hindered diisopropylethylamine (DIPEA), led to a significant diastereoselectivity shift in the reaction outcome. The major product became the (*S*,*S*)-bis-sulfinate ester **5(*S*,*S*)**, accompanied by the non-*C*_2_-symmetric bis-sulfinate ester **5(*R*,*S*)** in a 90:10 ratio. Notably, no trace of the **5(*R*,*R*)** bis-sulfinate ester was observed. The enhanced stability conferred by dicyclohexylidene-d-glucose, in contrast to diacetone-d-glucose, facilitated the purification of both propane-1,3-bis-sulfinate esters, **5(*R*,*R*)** and **5(*S*,*S*)**, which after removing the *meso* product via column chromatography were successfully obtained in 70% and 69% yields, respectively.

Given that in the synthesis of DCG propane-1,3-bis-sulfinates two stereogenic sulfinylic sulfur centers are simultaneously and independently generated in a dynamic kinetic resolution process, the significant diastereoselectivity observed for the *C*_2_-symmetric *RR*/*SS* diastereomers can be explained through the Horeau amplification phenomenon. Thus, by extrapolating the diastereomeric ratio of bis-sulfinates from the observed *S*/*R* ratio in the formation of the monosulfinate,^21^ specifically DCG propanesulfinate, we predicted the diastereomeric distribution as outlined in Table 1. As can be verified from Table 1, the theoretical calculation aligns with the experimental results.

**Table 1:**
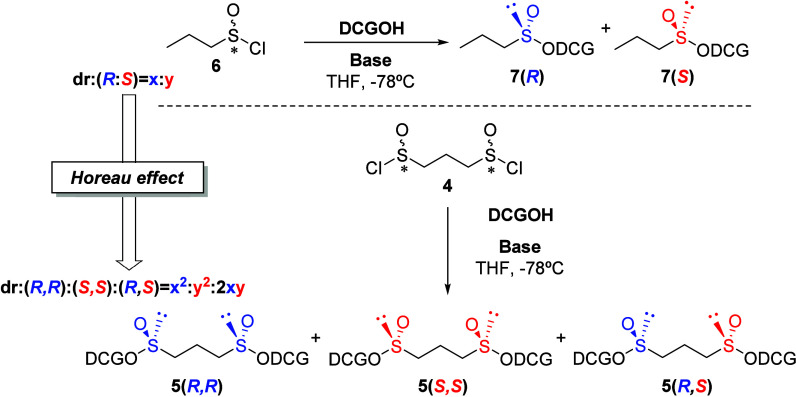
Comparing the Experimental Results and Theoretical Calculations Based on the Horeau Effect for Diastereomeric Ratios in DCG 1,3-Propane-bis(sulfinates) Synthesis

entry	base	sulfinate dr **7*****S***:**7*****R***^a^	major bis-sulfinate	experimental dr **(*****R***,***R*****)**:**(*****S***,***S*****)**:**(*****R***,***S*****)**^a^	theoretical dr **(*****R***,***R*****)**:**(*****S***,***S*****)**:**(*****R***,***S*****)**
1	Py	15:85	**5(*****R***,***R*****)**	70:4:26	72:2:26
2	DIPEA	96:4	**5(*****S***,***S*****)**	0:90:10	0:92:8

a Determined by ^1^H NMR of the crude mixture.

**Scheme 3:**
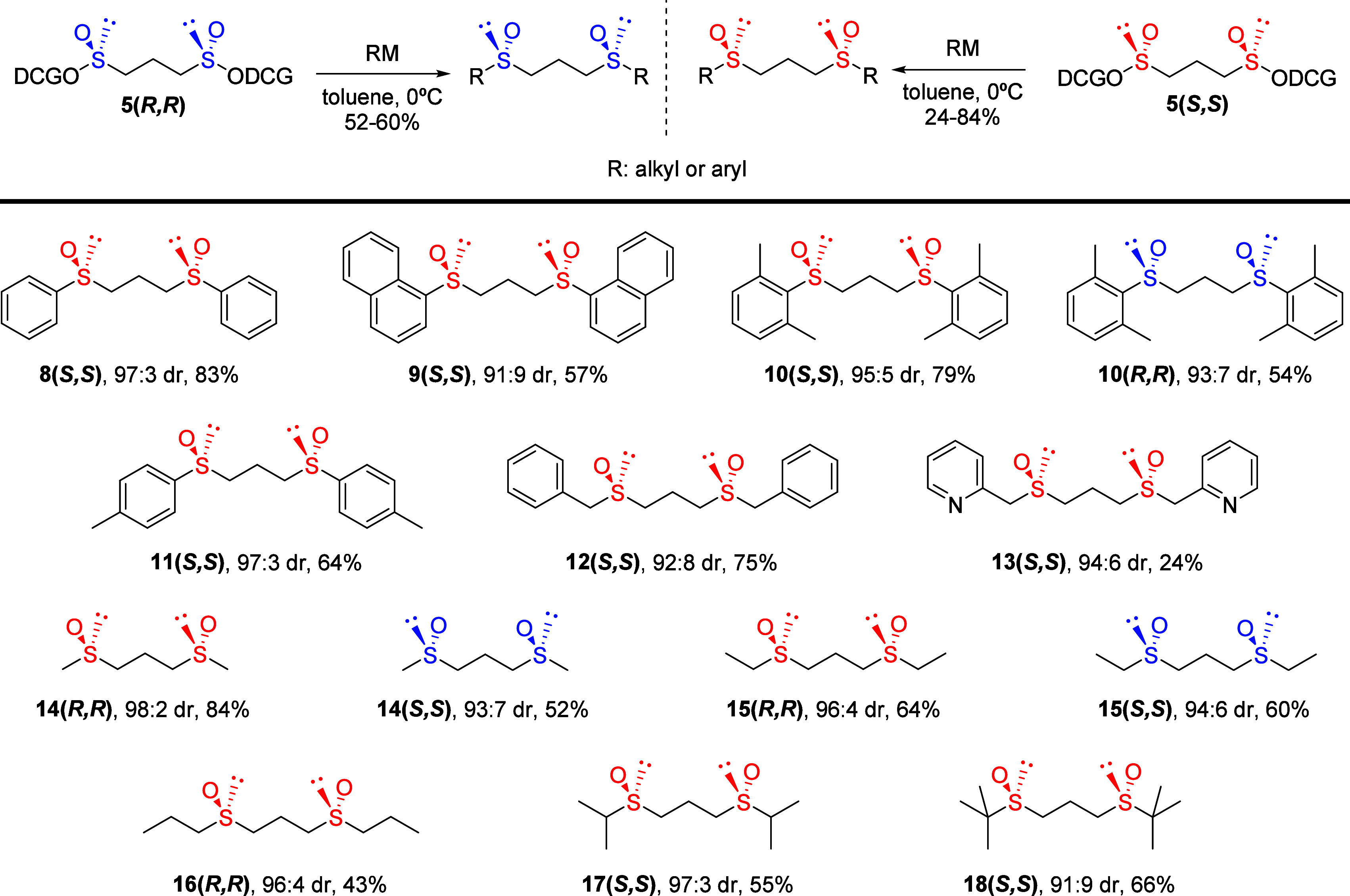
Synthesis of Optically Active *C*_2_-Symmetric 1,3-Bis(sulfinyl)propane from Chiral Propane-1,3-bis(sulfinate)

This result holds significant experimental importance because the crude reaction mixture of bis-sulfinates can lead to *C*_2_-symmetric bis-sulfoxides in high ee’s (90% ee and 100% ee from **5(*R*,*R*)** and **5(*S*,*S*)**, respectively) and may be used directly in the next step without further purification. This possibility is particularly interesting from an experimental standpoint when working on a multigram scale. However, owing to the stability and excellent resolution of bis-sulfinate mixtures in flash chromatography, diastereomerically pure DCG bis-sulfinate esters were successfully isolated by removing the (*R,S*)*-*diastereomer via column chromatography. These were subsequently employed in the synthesis of *C*_2_-symmetric bis-sulfoxides through treatment with alkyl and aryl Grignard reagents (Scheme 3). In this way, several 1,3-bis(aryl-) and 1,3-bis(alkylsulfoxides) have been obtained in modest to good yields (Scheme 3).

The configuration of the 1,3-bis-sulfoxides was assigned based on the configuration of the precursor bis-sulfinates considering that the alcohol displacement reaction in the sulfinates proceeds with inversion of configuration at the sulfur atom.

### Asymmetric Allylation Using the *C*_2_-Symmetric 1,3-Bis(sulfinyl)propanes as Organocatalysts

Taking advantage of the capability that sulfides have as Lewis bases, we have studied the catalytic and inductive capacity of these 1,3-bis-sulfoxides as chiral organocatalysts in the allylation of benzoylhydrazones with allyl trichlorosilane as the model reaction. To compare these results with those previously obtained using 1,2-bis-sulfoxides,^12^*N*-(benzoyl) isopropylhydrazone **19** was reacted with allyl trichlorosilane **20** at −78 °C using different bis-sulfoxides as organocatalysts in the presence of 2-methyl-2-butene to suppress ligand racemization (Table 2). The allylation product **21** is generally obtained with high yields (from 70% to quantitative) and enantioselectivities ranging from good to moderate. Overall, there is no significant improvement observed in the enantioselectivity of the process compared to the results obtained with the homologous 1,2-bis-sulfoxides as organocatalysts. Contrary to what might be expected, a larger steric volume of the aryl or alkyl sulfinyl group is not associated with a greater enantioselectivity of the process (compare entries 2 and 3 with entries 1 and 4 for aromatic derivatives and entry 6 with entry 5 in the case of aliphatic bis-sulfoxides).

**Table 2:**
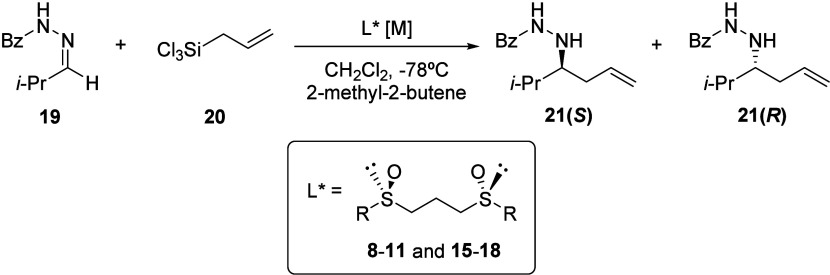
Asymmetric Allylation Using Different Propylene-Bridged Bis-Sulfoxides as Organocatalysts

	L*^a^			catalysis	
entry	R	bis-sulfoxide	dr	yield (%)^b^	**21(*****R*****)**:**21(*****S*****)**^c^
1	Ph	**8(*****S***,***S*****)**	97:3	95	25:75
2	2,6-Me_2_Ph	**10(*****S***,***S*****)**	95:5	93	30:70
3	*p*-Tol	**11(*****S***,***S*****)**	97:3	80	18:82
4	1-Naph	**9(*****S***,***S*****)**	91:9	90	43:57
5	*t-*Bu	**18(*****S***,***S*****)**	91:9	quant	78:22
6	*i*-Pr	**17(*****S***,***S*****)**	97:3	70	84:16
7	Pr	**16(*****R***,***R*****)**	96:4	quant	70:30
8	Et	**15(*****R***,***R*****)**	96:4	quant	65:35

a The reaction was carried out with 150 mol % of L* and at a concentration of 0.46 M.

b Isolated yield.

c Determined by chiral HPLC.

### Asymmetric Synthesis of *C*_2_-Symmetric 1,5-Bis(sulfinyl)-3-thio Derivatives

**Scheme 4:**
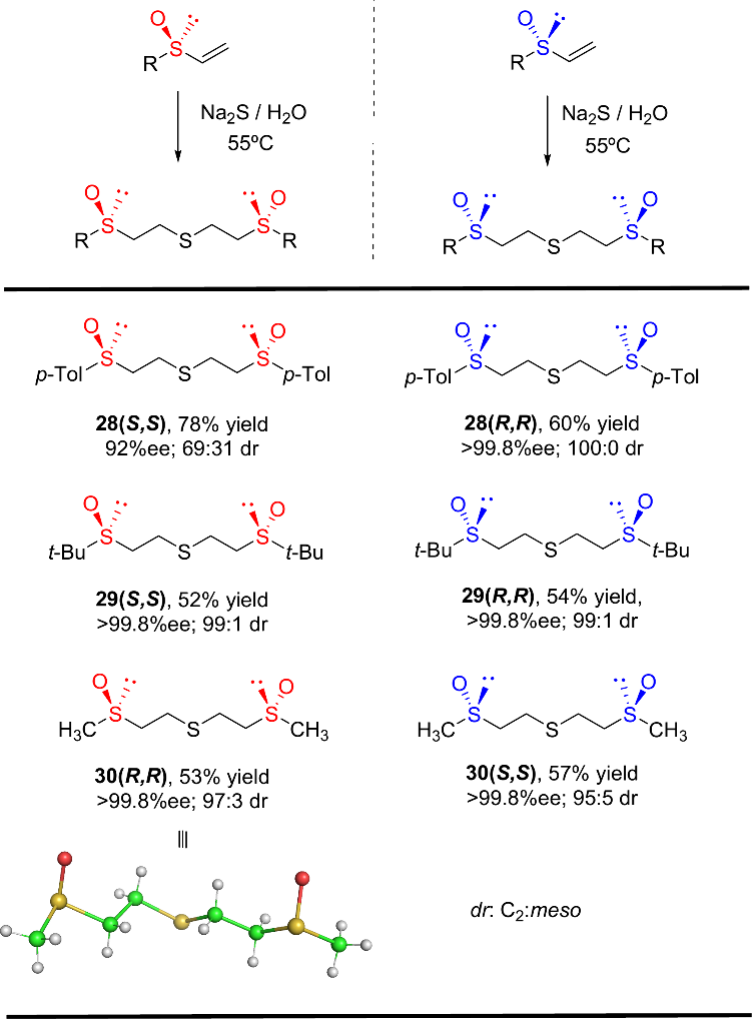
Synthesis of *C*_2_-Symmetric Enantiopure (*R*,*R*)- and (*S*,*S*)-1,5-Bis(sulfinyl)-3-thio Derivatives **28–30** by Dimerization of Enantiomerically Pure Vinyl Sulfoxides, **25–27**

Considering the impact of not only the chain length but also its nature on the catalytic activity of ligands, we embarked on synthesizing 1,5-bis(sulfinyl) derivatives incorporating a sulfur atom in the carbon chain as a third coordination element. While the literature documents the synthesis of these bis-sulfinyl derivatives in racemic form,^22^ a notable gap exists in achieving their pure enantiomeric forms. Thus, we aimed to develop an enantioselective approach. Building upon our established DAG methodology for generating enantiopure *R* and *S* vinyl sulfoxides (Table 3),^21^ we focused on optimizing the dimerization process using sodium disulfide (Scheme 4).

**Table 3:**
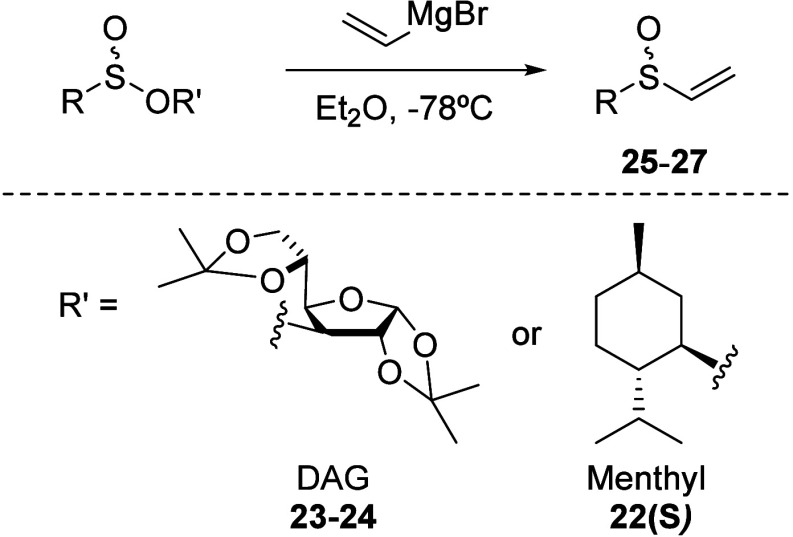
Synthesis of *R* and *S* Vinyl Sulfoxides **23**–**25**

entry	R	sulfinate	dr^a^ (*R*:*S*)	yield (%)	vinyl sulfoxide	er^b^ (*R*:*S*)	yield (%)
1	*p*-Tol	**22(*****S*****)**	>3:97	80	**25(*****R*****)**	>99.9:0.1	87
2	*t*-Bu	**23(*****R*****)**	>97:3	97	**26(*****S*****)**	>0.1:99.9	73
3	*t*-Bu	**23(*****S*****)**	>3:97	74	**26(*****R*****)**	>99.9:0.1	65
4	Me	**24(*****R*****)**	>97:3	quant	**27(*****R*****)**	99:1^c^	83
5	Me	**24(*****S*****)**	>3:97	quant	**27(*****S*****)**	2:98^c^	83

a Determined by ^1^H NMR.

b Determined by HPLC.

c Both enantiomers could not be separated by chiral HPLC; the er was determined from the data of the corresponding bis-sulfoxides based on the Horeau effect.

The reaction of DAGOH with the corresponding alkyl- or arylsulfinyl chloride in the presence of DIPEA or pyridine as a base at −78 °C yielded the different DAG sulfinates epimers in the sulfur as previously described.^21^ All sulfinates were stable enough to be purified by silica gel column chromatography, except for the DAG *p*-toluenesulfinates, where the two isomers are not sufficiently resolved for a convenient chromatographic separation.^21^ Fortunately, in this case, enantiopure vinyl *p*-tolyl sulfoxide **23(*****S*****)** was obtained from the corresponding (*S*)-menthyl *p*-toluenesulfinate **22(*****S*****)**.^23^

Subsequently, the vinyl sulfoxides were dimerized by treating them with sodium sulfide under moderate heating in an aqueous medium. The sulfide was added to the electrophilic vinyl double bond, which was activated by the sulfinyl group, yielding the corresponding bis(sulfinylethyl)sulfides, **28**–**30**, in moderate to good yields (52–78% yield, Scheme 4).

While in the synthesis of 1,3-bis-sulfinates discussed in the previous section the two chiral centers on sulfur were simultaneously generated in the process, in the synthesis of these 1,5-bis-sulfoxides, the chirality of sulfur in vinyl sulfoxides is predefined, as the way they combine during dimerization is what determines the configuration of the final bis-sulfoxide. Thus, the dimerization process of vinyl sulfoxides constitutes a paradigmatic case of the Horeau effect (Table 4).

**Table 4:**
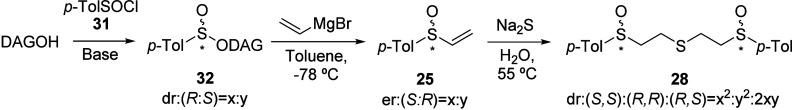
Horeau Effect in the Synthesis of 1,5-Bis-sulfoxide by Dimerization with NaS_2_

					stereoisomeric ratio (*R*,*R*):(*S*,*S*):(*R*,*S*)	
entry	base	sulfinate (*R*:*S*)^a^	vinyl sulfoxide **25*****R***:**25*****S***^b^	bis-sulfoxide	calcd^c^	expl^b^
1	Py	**32** (81:19)	18:82	**28(*****S***,***S*****)**	3:67:30	3:66:31
2	DIPEA	**32** (5:95)	94:6	**28(*****R***,***R*****)**	90:0:10	90:0:10

a Determined by ^1^H NMR of the crude mixture.

b Determined by HPLC.

c Calculated from the starting sulfinates applying the Horeau distribution.

Enantiomeric excesses in vinyl sulfoxides and the relation of diastereoisomers *C*_2_/*meso* in 1,5-bis(sulfoxides) was determined by chiral HPLC. Given the optimal conditions, DAG *p*-toluenesulfinate esters **32(*****R*****)** and **32(*****S*****)** were obtained with 62% and 90% diastereomeric excess (de), respectively (Table 4). The corresponding vinyl sulfoxides **25(*****R*****)** and **25(*****S*****)** exhibited a comparable enantiomeric excess, while bis-sulfoxides **28(*S*,*S*)** and **28(*R*,*R*)** were obtained with 92% and 100% enantiomeric excess, respectively.

**Scheme 5:**
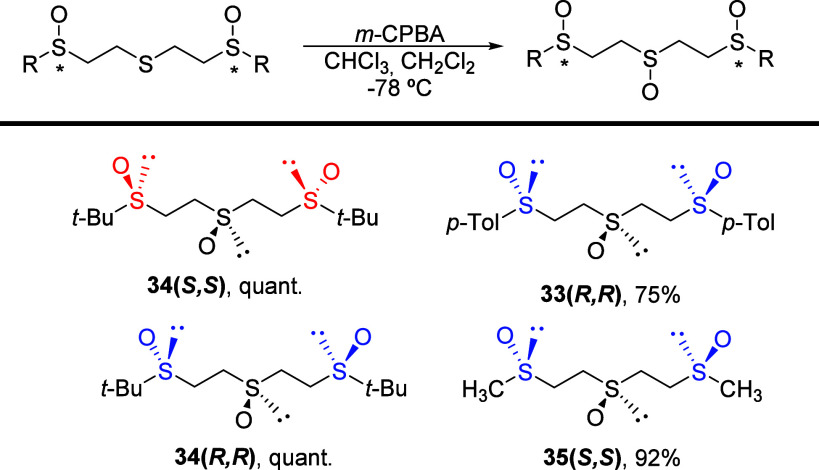
Regioselective Oxidation of 1,5-Bis(sulfinyl)-3-thio Derivatives

The regioselective oxidation of the sulfenyl sulfur in 1,5-bis(sulfinyl)-3-thio derivatives (**28**–**30**) lead to the corresponding trisulfoxides (**33**–**35**) as a new tridentate-type ligand (Scheme 5).

Despite the apparent simplicity of the obtained tris-sulfoxides, as no chiral center is generated in the oxidation process, an analysis of their spectroscopic features reveals a significant increase in the complexity of their coupling pattern compared to that of the precursor bis-sulfoxides, which can be attributed to the loss of the *C*_2_ axis as a symmetry element (see Figure S5, SI).

### Chiral Metal Complexes Containing *C*_2_-Symmetric Bis-Sulfoxides

#### 1,3-Bis(sulfinyl)propanes Palladium(II) Complexes

**Scheme 6:**
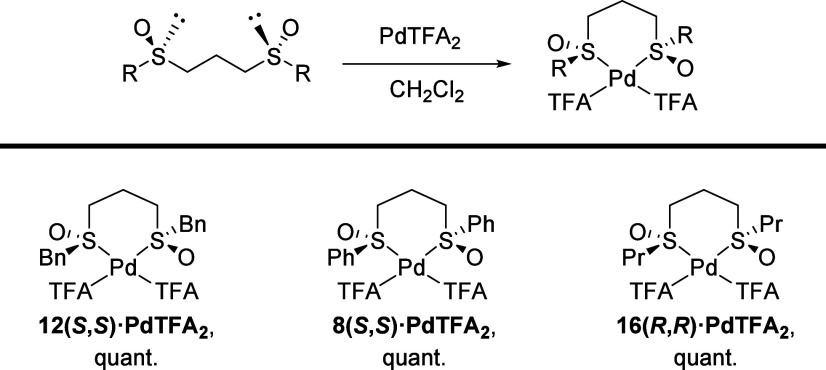
Synthesis of Palladium(II) Complexes

Following the methodology described for preparing 1,2-bis(sulfinyl)ethane palladium(II) complexes,^24^ we selected the homologous (*S*,*S*)-1,3-bis(benzylsulfinyl)propane, **12(*S*,*S*)**, as the model ligand and reacted it with palladium(II) acetate in CH_2_Cl_2_ under argon atmosphere. After 12 h at reflux, a red solid was obtained, whose ^1^H NMR spectrum showed an identical NMR pattern as the free ligand. The same result was obtained when changing the palladium precursor and using Pd(CH_3_CN)_2_Cl_2_. This result, similar to that described by the White^25^ and Dorta groups,^26^ can be justified by admitting that the interaction between the bis-sulfinyl ligand and the metal is not strong enough to remain bonded as a complex in solution. To solve this problem, the Pd precursor was changed again, and (*S*,*S*)-1,3-bis(benzylsulfinyl)propane, **12(*S*,*S*)**, was treated with Pd(TFA)_2_ in dry CH_2_Cl_2_ under argon atmosphere, Scheme 6.

This time, IR and ^1^H NMR data of the isolated solid showed that coordination of 1,3-bis(benzylsulfinyl)propane to Pd(TFA)_2_ was quite effective. Thus, substantial changes in the ^1^H NMR spectrum are evident, especially for the methylenic protons adjacent to the sulfinyl groups (see Figure S6 Supporting Information), and the IR spectra of the palladium complex exhibit an absorption band displacement from 1026 to 1178 and 1145 cm^–1^ with no significant absorption observed in the O-bound region of sulfoxides (1100–800 cm^–1^). Both results are consistent with the coordination of **12(*S*,*S*)** to palladium through the sulfinyl sulfurs. The ^19^F NMR spectrum shows one signal for both CF_3_ groups of TFA.

Under similar conditions, phenyl and propyl 1,3-bis-sulfinylpropane palladium(II) complexes, namely, **8(*S*,*S*)·PdTFA**_**2**_ and **16(*R*,*R*)·PdTFA**_**2**_, were synthesized in quantitative yields. In the case of bulky bis-sulfoxides, **10(*R*,*R*)**, **10(*S*,*S*)**, and **18(*S*,*S*)**, the steric hindrance posed by the *tert*-butyl groups or the ortho-disubstituted aromatic ring of the sulfinyl groups prevents the formation of the corresponding palladacycles.

#### 1,3- and 1,5-Bis(sulfinyl) Ruthenium(II) Complexes

**Scheme 7:**
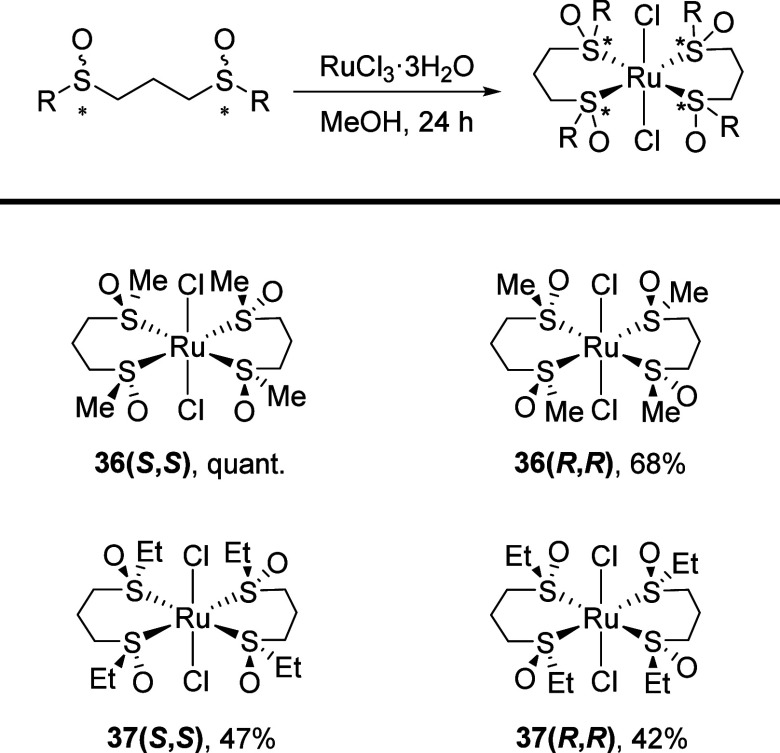
Synthesis of Ruthenium(II) Complexes

The research previously carried out on ruthenium(II) complexes of 1,2-bis(sulfinyl)ethanes has shown that the preferential formation of the *cis* or *trans* isomer depends largely on the substituent and chirality of the sulfinyl groups.^27^

(*S*,*S*)-1,3-Bis(methylsulfinyl)propane, (*R*,*R*)-1,3-bis(methylsulfinyl)propane, (*S*,*S*)-1,3-bis(ethylsulfinyl)propane, and (*R*,*R*)-1,3-bis(ethylsulfinyl)propane, **14(*S*,*S*)**, **14(*R*,*R*)**, **15(*S*,*S*)**, and **15(*R*,*R*)**, respectively, were reacted with ruthenium(III) chloride, previously activated in refluxing MeOH for 4 h, and after 24 h, the corresponding Ru(II) complexes were obtained. These ruthenium(II) complexes were not stable enough to be purified by silica gel chromatography, but they were purified by precipitation with Et_2_O from cold MeOH (Scheme 7). Although suitable crystals for determining the structure through X-ray diffraction were not available for these complexes, their *trans* geometry could be determined based on the simplicity of ^1^H NMR due to the *C*_2_ symmetry of the molecule. The strong deshielding of the neighboring methylene protons to the sulfinyl group (0.7 ppm, H_a_ protons in Table 5) together with the shift of the ν(S–O) band in IR from 995 and 1013 to 1080 cm^–1^ point out that the coordination of the sulfoxide to the metal takes place by the sulfur atoms.

**Table 5:** ^1^H NMR Data of Free Bis-Sulfoxide Ligands and Their Ru(II) Metal Complexes

			Δν (Hz)^a^			
entry	R	ligand	H_a_	H_b_	complex	Δν (Hz)^a^^,^^b^ H_b_
1	Me	**14(*****R***,***R*****)**	50		**36(*****S***,***S*****)**	
2	Me	**14(*****S***,***S*****)**	50		**36(*****R***,***R*****)**	
5	Et	**15(*****R***,***R*****)**	44	51	**37(*****S***,***S*****)**	270
6	Et	**15(*****S***,***S*****)**	44	51	**37(*****R***,***R*****)**	270

a Difference in chemical shift of the methylene protons (500 MHz) C*H*_b_–S(O)C*H*_a_CH_2_C*H*_a_S(O)–C*H*_b_ in MeOD.

b Δν of the H_a_ methylene protons are 0 Hz.

A decrease in the nonequivalence of the H_a_ methylene protons (Table 5) compared to free ligands was observed, rendering them equivalent in the four complexes. Additionally, a significant increase in the nonequivalence of the methylene protons of the ethyl substituent was noted in **37(*S*,*S*)** and **37(*R*,*R*)**, increasing from 51 Hz in the free ligands **15(*R*,*R*)** and **15(*S*,*S*)** to 270 Hz in the complexes.

**Scheme 8:**
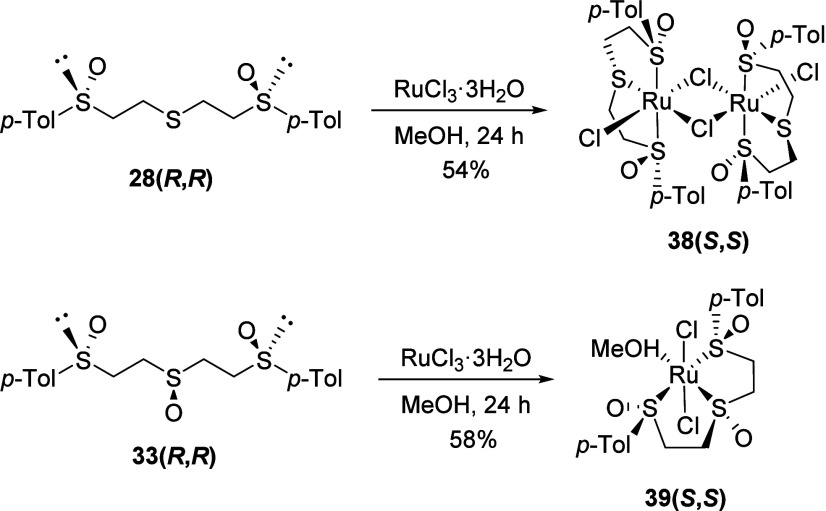
Ruthenium(II) Complex of 1,5-Bis-Sulfoxides: Influence of the Oxidation State of the Chain Central Sulfur in the Structure

Regarding the *C*_2_-symmetric 1,5-bis(sulfinyl) derivatives, the *tert*-butyl and methyl derivatives, **29(*R*,*R*)**, **29(*S*,*S*), 30(*R*,*R*)**, and **30(*S*,*S*)**, under similar conditions to those described previously for 1,3-bis(sulfinyl)propane led to a highly insoluble brown substance which could not be properly characterized. However, when the reaction was conducted with the *p*-tolyl derivative **28(*R*,*R*)** as the ligand, the resulting Ru complex displayed high solubility in organic solvents, facilitating thorough characterization. Moreover, it proved stable enough for purification through flash chromatography, yielding a 54% yield of the **38(*S*,*S*)** Ru(II) complex. The proposed structure of a symmetric dinuclear Ru(II) complex was supported by NMR and mass spectrometry data. In this structure, both Ru atoms are coordinated to a chlorine atom and the ligand via three sulfur atoms with two bridging chlorine atoms shared between them (Scheme 8).

While the ^1^H NMR spectrum of the starting ligand **28(*R*,*R*)** shows two signal sets corresponding to the two fragments of the ABXY systems of both ethylene fragments, in the spectrum of the Ru(II) complex **38(*S*,*S*)**, all protons of each ligand are diastereotopic, resulting in 8 multiplets that integrate for two protons (see Figures S7 and S8, SI).

Finally, the trisulfoxide ligand **33(*R*,*R*)** under the same conditions yielded a mononuclear Ru complex, denoted as **39(*S*,*S*)**. In this complex, Ru is bonded to a lone ligand molecule, positioned occupying three of the four equatorial positions, with two chlorine atoms in the axial positions. Mass spectrometry data led us to propose reversible binding of a methanol molecule, present in the reaction, at the fourth equatorial position, as previously described by James for other Ru complexes.^28^ A deshielding effect is observed for all protons in the NMR spectrum of the complex, with all of them being diastereotopic, as in the precursor ligand. It is worth noting the high nonequivalence of both *p*-tolyl groups in **39(*S*,*S*)**, including both the aromatic fragment and the *para*-methyl groups.

## Conclusion

In conclusion, the two developed strategies for the synthesis of 1,3-bis(sulfinyl)propanes and bis(alkyl- and arylsulfinylethyl) thioethers allow access to two new families of *C*_2_-symmetric chiral ligands in enantiopure form. The resulting synthetic routes not only offer practical and scalable approaches for chiral ligands but also provide insights into the use of Horeau’s law to achieve the desired stereochemical outcomes. The 1,3-bis-sulfoxides have proven to be effective organocatalysts in the allylation of hydrazones, similar to their lower homologues 1,2-bis(sulfinyl)ethanes, with no significant improvement in the enantioselectivity of the process compared to them. Finally, the synthesis and characterization of several metal, Pd and Ru, complexes have been carried out with potential applications in various fields, including coordination chemistry and catalysis. The inclusion of a third sulfur as a coordinating element in the 1,5-bis-sulfinyl derivatives facilitates the generation of two distinct tricoordinated Ru complexes whose structural configuration is intricately dictated by the oxidation state assumed by the sulfur as a thioether or a sulfoxide.

## Experimental Section

All reactions were run under an atmosphere of dry argon using oven-dried glassware and dried solvents. MeOH, toluene, THF, DMF, CH_2_Cl_2_, and diethyl ether were dried using molecular sieves, and the highest quality solvents were used. Chemicals were obtained from commercial sources and used without further purification. TLC was carried out on silica gel GF254 (Merck), and compounds were detected by charring with phosphomolybdic acid/EtOH. For flash chromatography, Merck 230–400 mesh silica gel was used. Chromatographic columns were eluted with a positive pressure of air, and eluents are given as volume-to-volume ratios (v/v). NMR spectra were recorded with Bruker Avance 300 and 500 MHz spectrometers. Chemical shifts are reported in ppm, and coupling constants are reported in Hertz (Hz). Routine ^1^H and ^13^C spectra were referenced to the residual proton or carbon signals of the solvent, respectively. High-resolution mass spectra (HRMS) were recorded with a Orbitrap Elite Hybrid Ion Trap-Orbitrap Mass Spectrometer. Optical rotations were determined with a Perkin–Elmer 341 polarimeter. Melting points were measured with a Stuart SMP3 apparatus in open-ended capillary tubes. Methyl, *p*-tolyl, and *tert*-butylsulfinyl chlorides were obtained by the method reported by Herrmann^29^ and Ellman.^30^*p*-Toluenesulfinates **32(*****R*****)** and **32(*****S*****)** and optically pure alkanesulfinates **23(*****R*****)**, **23(*****S*****)**, **24(*****R*****)**, and **24(*****S*****)** were prepared as previously described following the DAG methodology.^21,31^ Menthyl *p*-toluenesulfinate **22(*****S*****)** was prepared as described by Solladiè.^23^

### Stereoselective Synthesis of Propane-1,3-bis-sulfoxides

#### Propane-1,3-diyl Dimethanesulfonate, **2**

To a solution of propane-1,3-diol (9.5 mL, 13.15 mmol, 100 mol %) and Et_3_N (45.8 mL, 32.88 mmol, 250 mol %) in THF (200 mL), methanesulfonyl chloride (25.4 mL, 32.88 mmol, 250 mol %) was added dropwise at 0 °C. The mixture was warmed and stirred at room temperature overnight. Then, the reaction was quenched with saturated aqueous NH_4_Cl solution (100 mL), and the aqueous phase was extracted with CH_2_Cl_2_ (3 × 50 mL). The combined organic phases were washed with saturated aqueous NaHCO_3_ solution and saturated aqueous NaCl solution and dried with anhydrous Na_2_SO_4_. The solvent was removed under vacuum to give **2** (30.2 g, 13.00 mmol, quant.) as a yellow solid with no further purification. Mp: 49–51 °C. ^1^H NMR (500 MHz, CDCl_3_): δ 4.36 (t, *J* = 5.9 Hz, 4H), 3.04 (s, 6H), 2.20 (quint, *J* = 5.9 Hz, 2H) ppm. ^13^C{^1^H} NMR (125 MHz, CDCl_3_): δ 65.4, 37.6, 29.2 ppm. HRMS (ESI) *m*/*z*: [M + Na]^+^ calcd for C_5_H_12_O_6_S_2_Na 254.9968; found 254.9967.

#### Propane-1,3-diyl Dithioacetate, **3**

To a solution of **2** (10.0 g, 4.31 mmol, 100 mol %) in DMF (500 mL) at 0 °C, potassium thioacetate (12.3 g, 10.76 mmol, 250 mol %) was added slowly. The mixture was warmed and stirred at rt overnight. Then, the reaction was quenched with saturated aqueous NaHCO_3_ solution (250 mL), and the aqueous phase was extracted with CH_2_Cl_2_ (3 × 100 mL). The combined organic phases were washed with saturated aqueous NaCl solution and dried with anhydrous Na_2_SO_4_. The solvent was removed under vacuum to give **3** (8.3 g, 4.31 mmol, quant. yield) as a brown liquid with no further purification. ^1^H NMR (500 MHz, CDCl_3_): δ 2.92 (t, *J* = 7.2 Hz, 4H), 2.33 (s, 6H), 1.86 (quint, *J* = 7.2 Hz, 2H) ppm. ^13^C{^1^H} NMR (125 MHz, CDCl_3_): δ 195.6, 30.7, 29.7, 28.1 ppm. HRMS (ESI) *m*/*z*: [M + Na]^+^ calcd for C_7_H_12_O_2_S_2_Na 215.0171; found 215.0170.

#### Propane-1,3-bis(sulfinyl) Chloride, **4**

To a solution of **3** (1.0 g, 5.20 mmol, 100 mol %) and acetic anhydride (1.0 mL, 10.40 mmol, 200 mol %) in CH_2_Cl_2_ (6 mL), sulfuryl chloride (1.7 mL, 20.80 mmol, 400 mol %) was added dropwise at −20 °C. The resulting mixture was stirred for 2 h at −5 °C. Then, the solvent was coevaporated with toluene (3 × 8 mL), and the desired compound was obtained as a pink oil with no further purification (1.1 g, 5.26 mmol, quant. yield). ^1^H NMR (300 MHz, CDCl_3_): δ 3.59 (t, *J* = 7.6 Hz, 4H), 2.91–2.60 (m, 2H) ppm.

#### Di(1,2:5,6-di-*O*-cyclohexylidene-α-d-glucofuranosyl) (*S*,*S*)-Propane-1,3-bis(sulfinate), **5(*S*,*S*)**

To a solution of 1,2:5,6-di-*O*-cyclohexylidene-α-d-glucofuranose (DCGOH) (3.2 g, 9.45 mmol, 200 mol %) and diisopropylethyl amine (1.9 mL, 10.87 mmol, 230 mol %) in a mixture of dry toluene (100 mL) and dry CH_2_Cl_2_ (2 mL) under argon atmosphere, at −78 °C, a solution of bis(sulfinyl) chloride **4** (1.1 g, 5.20 mmol, 110 mol %) in toluene (2 mL) was added dropwise with vigorous stirring. The reaction was stirred for 1 h at −78 °C, then HCl (10% aq) was added, and the aqueous phase was extracted with CH_2_Cl_2_ (3 × 70 mL). The combined organic phases were washed with saturated aqueous NaHCO_3_ solution and saturated aqueous NaCl solution and dried with anhydrous Na_2_SO_4_. The solvent was removed under vacuum to give bis-sulfinate **5** (3.9 g, 4.78 mmol, quant. yield) in a (*S*,*S*):(*R*,*S*):(*R*,*R*) ratio of 90:10:0 as a yellow solid. Purification by column chromatography (TBME/hexane, 1:1.4) gave diastereomerically pure sulfinate **5(*S*,*S*)** (2.7 g, 3.26 mmol, 69% yield) as a yellow solid. Mp: 66–69 °C. [α]_D_^20^: −46.7 (*c* 1, CHCl_3_). ^1^H NMR (500 MHz, C_6_D_6_): δ 5.90 (d, *J* = 3.7 Hz, 2H), 4.87 (d, *J* = 2.8 Hz, 2H), 4.54 (d, *J* = 3.7 Hz, 2H), 4.50 (dd, *J* = 7.5 Hz, *J* = 2.8 Hz, 2H), 4.47–4.43 (m, 2H), 4.14–4.09 (m, 4H), 2.40 (t, *J* = 7.7 Hz, 4H), 1.90 (quint, *J* = 7.7 Hz, 2H), 1.74–1.15 (m, 40H) ppm. ^13^C{^1^H} NMR (125 MHz, C_6_D_6_): δ 113.2, 110.2, 105.2, 83.8, 81.1, 79.9, 72.7, 67.1, 55.4, 37.1, 36.9, 36.2, 35.2, 25.6, 25.2, 24.5, 24.3, 24.2, 23.9, 13.5 ppm. HRMS (ESI) *m*/*z*: [M + Na]^+^ calcd for C_39_H_60_O_14_S_2_Na 839.3317; found 839.3303.

#### Di(1,2:5,6-di-*O*-cyclohexylidene-α-d-glucofuranosyl) (*R*,*R*)-Propane-1,3-bis(sulfinate), **5(*R*,*R*)**

To a solution of DCGOH (3.2 g, 9.45 mmol, 200 mol %) and pyridine (0.9 mL, 10.87 mmol, 230 mmol %) in dry THF (100 mL) under argon atmosphere at −78 °C, a solution of bis(sulfinyl) chloride **4** (1.1 g, 3.20 mmol, 110 mol %) in toluene (6 mL) was added dropwise with vigorous stirring. The reaction was stirred for 1 h at −78 °C, then HCl (10% aq) was added, and the aqueous phase was extracted with CH_2_Cl_2_ (3 × 30 mL). The combined organic phases were washed with a saturated aqueous NaHCO_3_ solution and saturated aqueous NaCl solution and dried with anhydrous Na_2_SO_4_. The solvent was removed under vacuum to give bis-sulfinate **5** (3.9 g, 4.73 mmol, quant. yield) in a (*S*,*S*):(*R*,*S*):(*R*,*R*) ratio of 4:26:70 as a brown oil. Purification by column chromatography (TBME/hexane, 1:5) gave diastereomerically pure sulfinate **5(*R*,*R*)** (2.7 g, 3.31 mmol, 70% yield) as a yellow oil. [α]_D_^20^: −1.3 (*c* 1, CHCl_3_). ^1^H NMR (500 MHz, C_6_D_6_): δ 5.93 (d, *J* = 3.6 Hz, 2H), 4.90–4.89 (m, 4H), 4.42 (dd, *J* = 8.7 Hz, *J* = 3.0 Hz, 2H), 4.31–4.27 (m, 2H), 4.04 (d, *J* = 5.3 Hz, 4H), 2.56 (t, *J* = 7.5 Hz, 4H), 2.11 (quint, *J* = 7.5 Hz, 2H), 1.71–1.11 (m, 40H) ppm. ^13^C{^1^H} NMR (125 MHz, C_6_D_6_): δ 113.1, 110.2, 105.6, 84.1, 83.7, 81.5, 72.7, 67.7, 56.0, 37.2, 36.9, 35.9, 35.3, 25.5, 25.1, 24.5, 24.3, 24.2, 23.9, 13.1 ppm. HRMS (ESI) *m*/*z*: [M + Na]^+^ calcd for C_39_H_60_O_14_S_2_Na 839.3317; found 839.3307. The diastereomerically pure sulfinate **5(*R*,*S*)** (245.0 mg, 0.30 mmol, 6% yield) was formed as a yellow oil. Mp: 67–69 °C. [α]_D_^20^: −18.1 (*c* 1, CHCl_3_). ^1^H NMR (500 MHz, C_6_D_6_): δ 5.92 (d, *J* = 3.4 Hz, 1H), 5.87 (d, *J* = 3.6 Hz, 1H), 4.91–4.89 (m, 2H), 4.87 (d, *J* = 2.7 Hz, 1H), 4.53 (d, *J* = 3.6 Hz, 1H), 4.51–4.42 (m, 3H), 4.29–4.25 (m, 1H), 4.15–4.09 (m, 2H), 4.07–4.01 (m, 2H), 2.51 (t, *J* = 7.7 Hz, 2H), 2.43 (t, *J* = 7.7 Hz, 2H), 2.06–1.95 (m, 2H), 1.74–1.13 (m, 40H) ppm. ^13^C{^1^H} NMR (125 MHz, C_6_D_6_): δ 113.2, 113.1, 110.3, 110.2, 105.6, 105.2, 84.0, 83.8 (2), 81.4, 81.1, 79.6, 72.6 (2), 67.6, 67.0, 55.8, 55.5, 37.2, 37.1, 36.9 (2), 36.1, 36.0, 35.9, 35.3, 25.6, 25.5, 25.1, 24.5, 24.4, 24.3 (2), 24.2 (2), 23.9 (2), 13.5 ppm. HRMS (ESI) *m*/*z*: [M + Na]^+^ calcd for C_39_H_60_O_14_S_2_Na 839.3317; found 839.3304.

### General Procedure for Preparation of Chiral Bis-Sulfoxides by Nucleophilic Substitution on Chiral Bis-Sulfinates

To a solution of 1,3-bis-sulfinate esters **5(*S*,*S*)** or **5(*R*,*R*)** (100 mol %) in dry toluene under argon atmosphere at 0 °C, the corresponding solution of RMgX or RLi (R = alkyl or aryl, X = Br or Cl) (300–500 mol %) was added dropwise. The mixture was stirred for 1 h and quenched with saturated aqueous NH_4_Cl solution, and the aqueous phase was extracted with CH_2_Cl_2_. The combined organic phases were washed with saturated aqueous NaCl solution and dried with anhydrous Na_2_SO_4_. The residue was purified by flash chromatography. In the case of 1,3-bis(alkylsulfinyl)propanes **14**–**18**, the work up was different. After the reaction mixture was stirred for 1 h, 100 mol % of TFA was added and the solvent was removed in vacuo. The residue was dissolved in MeOH and treated with mixed bed resin (Sigma TMD-8; 1:1 mixture of strong cation and anion exchange resin) to remove the remaining salts. After that, it was purified by column chromatography on silica gel.

#### (*S*,*S*)-1,3-Bis(phenylsulfinyl)propane, **8(*S*,*S*)**

**8(*S*,*S*)** was prepared following the general procedure from bis-sulfinate **5(*S*,*S*)** (1.5 g, 1.84 mmol), 1 M PhMgBr solution (5.5 mL, 5.52 mmol, 300 mol %), and toluene (40 mL). The residue was purified by flash chromatography (CH_2_Cl_2_/isopropanol, 40:1) to obtain bis-sulfoxide **8(*S*,*S*)** (444.0 mg, 1.52 mmol, 83% yield) as a yellow solid. Mp: 107–109 °C. [α]_D_^20^: −276.6 (*c* 1, CHCl_3_). ^1^H NMR (500 MHz, CDCl_3_): δ 7.57–7.54 (m, 4H), 7.53–7.49 (m, 6H), 2.93 (AB fragment of an ABX_2_ system, Δν = 80 Hz, *J*_AX_ = 7.0 Hz, *J*_BX_ = 7.6 Hz, *J*_AB_ = 13.5 Hz, 4H), 2.11 (quint, *J* = 7.3 Hz, 2H) ppm. ^13^C{^1^H} NMR (125 MHz, CDCl_3_): δ 143.3, 131.3, 129.5, 124.1, 55.2, 15.6 ppm. HRMS (ESI) *m*/*z*: [M + Na]^+^ calcd for C_15_H_16_O_2_NaS_2_ 315.0484; found 315.0484. 97:3:0 dr [(*S*,*S*):(*R*,*S*):(*R*,*R*)]. HPLC: ADH Chiracel column (*n*-hexane/isopropanol 60:40; 0.6 mL/min.; 23 °C) *t*_R_ = 12.32 min [(*S*,*S*)-isomer], *t*_R_ = 13.18 min [(*S*,*R*)-isomer].

#### (*S*,*S*)-Bis(1-naphthylsulfinyl)propane, **9(*S*,*S*)**

**9(*S*,*S*)** was prepared following the general procedure from bis-sulfinate **5(*S*,*S*)** (882.7 mg, 1.01 mmol), 0.25 M 1-naphthylmagnesium bromide solution (20.2 mL, 5.05 mmol, 500 mol %), and toluene (40 mL). The residue was purified by flash chromatography (TBME/hexane, 4:1) to obtain bis-sulfoxide **9(*S*,*S*)** (227.0 mg, 0.58 mmol, 57% yield) as a crystalline solid. Mp: 145–148 °C. [α]_D_^20^: −552.4 (*c* 1, CHCl_3_). ^1^H NMR (500 MHz, CDCl_3_): δ 7.96–7.88 (m, 8H), 7.60–7.54 (m, 6H), 3.06 (AB fragment of an ABX_2_ system, Δν = 140 Hz, *J*_AX_ = 7.1 Hz, *J*_BX_ = 7.7 Hz, *J*_AB_ = 13.6 Hz, 4H), 2.13 (quint, *J* = 7.4 Hz, 2H) ppm. ^13^C{^1^H} NMR (125 MHz, CDCl_3_): δ 138.6, 133.7, 131.5, 129.3, 128.9, 127.6, 126.9, 125.6, 123.4, 121.5, 53.3, 15.9 ppm. HRMS (ESI) *m*/*z*: [M + Na]^+^ calcd for C_23_H_20_O_2_NaS_2_ 415.0797; found 415.0788. 91:9:0 dr [(*S*,*S*):(*R*,*S*):(*R*,*R*)]. HPLC: AD Chiracel column (*n*-hexane/isopropanol 25:75; 0.2 mL/min.; 23 °C) *t*_R_ = 46.57 min [(*R*,*S*)-isomer], *t*_R_ = 54.43 min [(*S*,*S*)-isomer].

#### (*S*,*S*)-1,3-Bis[(2,6-dimethylphenyl)sulfinyl]propane, **10(*S*,*S*)**

**10(*S*,*S*)** was prepared following the general procedure from bis-sulfinate **5(*S*,*S*)** (712.5 mg, 0.87 mmol), 1 M 2,6-Me_2_PhMgBr solution (4.4 mL, 3.91 mmol, 450 mol %), and toluene (40 mL). The residue was purified by flash chromatography (TBME/hexane, 4:1) to obtain bis-sulfoxide **10(*S*,*S*)** (239.0 mg, 0.69 mmol, 79% yield) as a yellow solid. Mp: 149–152 °C. [α]_D_^20^: −232.3 (*c* 1, CHCl_3_). ^1^H NMR (500 MHz, CDCl_3_): δ 7.24 (t, *J* = 7.6 Hz, 2H), 7.05 (d, *J* = 7.6 Hz, 4H), 3.22 (AB fragment of an ABX_2_ system, Δν = 190 Hz, *J*_AX_ = 7.8 Hz, *J*_BX_ = 6.9 Hz, *J*_AB_ = 13.3 Hz, 4H), 2.57 (s, 12H), 2.35 (quint, *J* = 7.4 Hz, 2H) ppm. ^13^C{^1^H} NMR (125 MHz, CDCl_3_): δ 138.4, 137.8, 131.2, 130.5, 50.6, 19.4, 19.3 ppm. HRMS (ESI) *m*/*z*: [M + Na]^+^ calcd for C_19_H_24_O_2_NaS_2_ 371.1110; found 371.1102. 95:5:0 dr [(*S*,*S*):(*R*,*S*):(*R*,*R*)]. HPLC: ADH Chiracel column (*n*-hexane/isopropanol 60:40; 0.6 mL/min.; 23 °C) *t*_R_ = 10.08 min [(*R*,*S*)-isomer], *t*_R_ = 12.12 min [(*S*,*S*)-isomer].

#### (*R*,*R*)-1,3-Bis[(2,6-dimethylphenyl)sulfinyl]propane, **10(*R*,*R*)**

**10(*R*,*R*)** was prepared following the general procedure from bis-sulfinate **5(*R*,*R*)** (500.0 mg, 0.61 mmol), 1 M 2,6-Me_2_PhMgBr solution (1.8 mL, 1.84 mmol, 300 mol %), and toluene (15 mL). The residue was purified by flash chromatography (CH_2_Cl_2_/MeOH, 25:1) to obtain bis-sulfoxide **10(*R*,*R*)** (115.0 mg, 0.33 mmol, 54% yield) as a yellow solid with similar physicochemical and spectroscopic characteristics to **10(*S*,*S*)**. [α]_D_^20^: +243.8 (*c* 1, CHCl_3_). HRMS (ESI) *m*/*z*: [M + Na]^+^ calcd for C_19_H_24_O_2_NaS_2_ 371.1110; found 371.1107. 0:7:93 dr [(*S*,*S*):(*R*,*S*):(*R*,*R*)]. HPLC: ADH Chiracel column (*n*-hexane/isopropanol 60:40; 0.6 mL/min.; 23 °C) *t*_R_ = 10.89 min [(*R*,*S*)-isomer], *t*_R_ = 11.82 min [(*R*,*R*)-isomer].

#### (*S*,*S*)-1,3-Bis(*p*-tolylsulfinyl)propane, **11(*S*,*S*)**

**11(*S*,*S*)** was prepared following the general procedure from bis-sulfinate **5(*S*,*S*)** (1.8 g, 2.15 mmol), 0.5 M *p*-MePhMgBr solution (12.9 mL, 6.46 mmol, 300 mol %), and toluene (40 mL). The residue was purified by flash chromatography (CH_2_Cl_2_/MeOH, 60:1) to obtain bis-sulfoxide **11(*S*,*S*)** (440.0 mg, 1.37 mmol, 64% yield) as a yellow solid. Mp: 108–111 °C. [α]_D_^20^: −238.2 (*c* 1, CHCl_3_). ^1^H NMR (500 MHz, MeOD): δ 7.50 (d, *J* = 8.1 Hz, 4H), 7.39 (d, *J* = 8.1 Hz, 4H), 2.99 (AB fragment of an ABX_2_ system, Δν = 40 Hz, *J*_AX_ = 7.4 Hz, *J*_BX_ = 7.7 Hz, *J*_AB_ = 13.5 Hz, 4H), 2.42 (s, 6H), 1.92 (quint, *J* = 7.6 Hz, 2H) ppm. ^13^C{^1^H} NMR (125 MHz, MeOD): δ 143.6, 140.1, 131.2, 125.4, 55.2, 21.4, 16.2 ppm. HRMS (ESI) *m*/*z*: [M + Na]^+^ calcd for C_17_H_20_O_2_NaS_2_ 343.0796; found 343.0797. 97:3:0 dr [(*S*,*S*):(*R*,*S*):(*R*,*R*)]. HPLC: ADH Chiracel column (*n*-hexane/isopropanol 60:40; 0.4 mL/min.; 23 °C) *t*_R_ = 30.47 min [(*R*,*S*)-isomer], *t*_R_ = 31.71 min [(*S*,*S*)-isomer].

#### (*S*,*S*)-1,3-Bis(benzylsulfinyl)propane, **12(*S*,*S*)**

**12(*S*,*S*)** was prepared following the general procedure from bis-sulfinate **5(*S*,*S*)** (1.0 g, 1.22 mmol), 2 M BnMgBr solution (1.8 mL, 3.67 mmol, 300 mol %), and toluene (30 mL). The residue was purified by flash chromatography (CH_2_Cl_2_/isopropanol, 40:1) to obtain bis-sulfoxide **12(*S*,*S*)** (292.0 mg, 0.92 mmol, 75% yield) as a yellow solid. Mp: 167–170 °C. [α]_D_^20^: −117.5 (*c* 1, CHCl_3_). ^1^H NMR (500 MHz, CDCl_3_): δ 7.40–7.34 (m, 6H), 7.28–7.26 (m, 4H), 3.99 (dd, *J* = 12.9 Hz, *J* = 35.3 Hz, 4H), 2.69 (AB fragment of an ABX_2_ system, Δν = 45 Hz, *J*_AX_ = 6.9 Hz, *J*_BX_ = 7.8 Hz, *J*_AB_ = 13.3 Hz, 4H), 2.29 (quint, *J* = 7.5 Hz, 2H) ppm. ^13^C{^1^H} NMR (125 MHz, CDCl_3_): δ 130.1, 129.6, 129.2, 128.7, 58.5, 49.2, 16.4 ppm. HRMS (ESI) *m*/*z*: [M + Na]^+^ calcd for C_17_H_20_O_2_NaS_2_ 343.0793; found 343.0797. 92:8:0 dr [(*S*,*S*):(*R*,*S*):(*R*,*R*)]. HPLC: AD Chiracel column (*n*-hexane/isopropanol 50:50; 0.2 mL/min.; 23 °C) t_R_ = 33.81 min [(*S*,*S*)-isomer], t_R_ = 36.37 min [(*R*,*S*)-isomer].

#### (*S*,*S*)-1,3-Bis[(pyridin-2-ylmethyl)sulfinyl]propane, **13(*S*,*S*)**

**13(*S*,*S*)** was prepared following the general procedure from bis-sulfinate **5(*S*,*S*)** (1.3 g, 1.58 mmol), 2-methylpyridine (0.7 mL, 6.71 mmol, 425 mol %), 1.87 M *n-*BuLi solution (3.4 mL, 6.32 mmol, 400 mol %), and THF (50 mL). The residue was purified by flash chromatography (CH_2_Cl_2_/MeOH, 20:1) to obtain bis-sulfoxide **13(*S*,*S*)** (120.0 mg, 0.37 mmol, 24% yield) as a brown oil. [α]_D_^20^: +23.5 (*c* 1, MeOH). ^1^H NMR (500 MHz, CDCl_3_): δ 8.60 (d, *J* = 4.4 Hz, 2H), 7.70 (dt, *J* = 7.7 Hz, *J* = 1.7 Hz, 2H), 7.35 (d, *J* = 7.8 Hz, 2H), 7.28–7.26 (m, 2H), 4.16 (dd, *J* = 13.0 Hz, *J* = 42.2 Hz, 4H), 2.87 (AB fragment of an ABX_2_ system, Δν = 62 Hz, *J*_AX_ = 7.1 Hz, *J*_BX_ = 7.9 Hz, *J*_AB_ = 13.3 Hz, 4H), 2.32 (quint, *J* = 7.5 Hz, 2H) ppm. ^13^C{^1^H} NMR (125 MHz, CDCl_3_): δ 150.6, 150.2, 137.1, 125.5, 123.3, 59.7, 50.0, 16.6 ppm. HRMS (ESI) *m*/*z*: [M + H]^+^ calcd for C_15_H_19_N_2_O_2_S_2_ 323.0879; found 323.0882. 94:6:0 dr [(*S*,*S*):(*R*,*S*):(*R*,*R*)]. HPLC: AD Chiracel column (*n*-hexane/isopropanol 60:40; 0.6 mL/min.; 23 °C) *t*_R_ = 44.03 min [(*S*,*S*)-isomer], *t*_R_ = 50.13 min [(*R*,*S*)-isomer].

#### (*R*,*R*)-1,3-Bis(methylsulfinyl)propane, **14(*R*,*R*)**

**14(*R*,*R*)** was prepared following the general procedure from bis-sulfinate **5(*S*,*S*)** (2.8 g, 3.43 mmol), 3 M MeMgBr solution (3.4 mL, 10.29 mmol, 300 mol %), and toluene (120 mL). The residue was purified by flash chromatography (CH_2_Cl_2_/MeOH, 20:1) to obtain bis-sulfoxide **14(*R*,*R*)** (483.0 mg, 2.87 mmol, 84% yield) as a yellow oil. [α]_D_^20^: −167.1 (*c* 1, MeOH). ^1^H NMR (500 MHz, MeOD): δ 2.97 (AB fragment of an ABX_2_ system, Δν = 50 Hz, *J*_AX_ = 7.3 Hz, *J*_BX_ = 7.9 Hz, *J*_AB_ = 13.3 Hz, 4H), 2.67 (s, 6H), 2.24 (quint, *J* = 7.6 Hz, 2H) ppm. ^13^C{^1^H} NMR (125 MHz, MeOD): δ 53.1, 38.2, 17.4 ppm. HRMS (ESI) *m*/*z*: [M + Na]^+^ calcd for C_5_H_12_O_2_NaS_2_ 191.0171; found 191.0171. 0:2:98 dr [(*S*,*S*):(*R*,*S*):(*R*,*R*)]. HPLC: IH Chiracel column (acetonitrile/water 95:5; 0.8 mL/min.; 25 °C) *t*_R_ = 9.63 min [(*R*,*S*)-isomer], *t*_R_ = 12.11 min [(*R*,*R*)-isomer]. FTIR (cm^–1^): 995 (ν_SO_).

#### (*S*,*S*)-1,3-Bis(methylsulfinyl)propane, **14(*S*,*S*)**

**14(*S*,*S*)** was prepared following the general procedure from bis-sulfinate **5(*R*,*R*)** (1.6 g, 1.95 mmol), 3 M MeMgBr solution (2.0 mL, 5.85 mmol, 300 mol %), and toluene (70 mL). The residue was purified by flash chromatography (CH_2_Cl_2_/MeOH, 40:1) to obtain bis-sulfoxide **14(*S*,*S*)** (171.0 mg, 1.02 mmol, 52% yield) as a yellow oil with similar physicochemical and spectroscopic characteristics to **14(***R*,*R***)**. [α]_D_^20^: +169.5 (*c* 1, MeOH). HRMS (ESI) *m*/*z*: [M + H]^+^ calcd for C_5_H_13_O_2_S_2_ 169.0347; found 169.0351. 93:7:0 dr [(*S*,*S*):(*R*,*S*):(*R*,*R*)]. HPLC: IH Chiracel column (acetonitrile/water 95:5; 0.8 mL/min.; 25 °C) *t*_R_ = 7.80 min [(*S*,*S*)-isomer], *t*_R_ = 9.73 min [(*R*,*S*)-isomer]. FTIR (cm^–1^): 995 (ν_SO_).

#### (*R*,*R*)-1,3-Bis(ethylsulfinyl)propane, **15(*R*,*R*)**

**15(*R*,*R*)** was prepared following the general procedure from bis-sulfinate **5(*S*,*S*)** (422.4 mg, 0.52 mmol), 3 M EtMgBr solution (0.87 mL, 2.6 mmol, 500 mol %), and toluene (10 mL). The residue was purified by flash chromatography (TBME/MeOH, 10:1) to obtain bis-sulfoxide **15(*R*,*R*)** (65.0 mg, 0.33 mmol, 64% yield) as a yellow solid. Mp: 127–129 °C. [α]_D_^20^: −61.2 (*c* 1, MeOH). ^1^H NMR (500 MHz, MeOD): δ 2.99 (dt, *J* = 8.0 Hz, *J* = 13.3 Hz, 2H), 2.94–2.86 (m, 4H), 2.80 (dq, *J* = 7.4 Hz, *J* = 13.3 Hz, 2H), 2.25 (quint, *J* = 7.6 Hz, 2H), 1.34 (t, *J* = 7.5 Hz, 6H) ppm. ^13^C{^1^H} NMR (125 MHz, MeOD): δ 50.5, 46.3, 17.8, 7.0 ppm. HRMS (ESI) *m*/*z*: [M + Na]^+^ calcd for C_7_H_16_O_2_NaS_2_ 219.0483; found 219.0484. 0:4:96 dr [(*S*,*S*):(*R*,*S*):(*R*,*R*)]. HPLC: ADH Chiracel column (*n*-hexane/isopropanol 60:40; 0.6 mL/min.; 23 °C) *t*_R_ = 8.05 min [(*R*,*R*)-isomer], *t*_R_ = 9.14 min [(*R*,*S*)-isomer]. FTIR (cm^–1^): 1013 (ν_SO_).

#### (*S*,*S*)-1,3-Bis(ethylsulfinyl)propane, **15(*S*,*S*)**

**15(*S*,*S*)** was prepared following the general procedure from bis-sulfinate **5(*R*,*R*)** (1.9 g, 2.31 mmol), 3 M EtMgBr solution (2.3 mL, 6.93 mmol, 300 mol %), and toluene (40 mL). The residue was purified by flash chromatography (CH_2_Cl_2_/MeOH, 40:1) to obtain bis-sulfoxide **15(*S*,*S*)** (273.0 mg, 1.39 mmol, 60% yield) as a yellow solid with similar physicochemical and spectroscopic characteristics to **15(*R*,*R*)**. [α]_D_^20^: +65.1 (*c* 1, MeOH). HRMS (ESI) *m*/*z*: [M + H]^+^ calcd for C_7_H_17_O_2_S_2_ 197.0661; found 197.0664; 94:6:0 dr [(*S*,*S*):(*R*,*S*):(*R*,*R*)]. HPLC: ADH Chiracel column (*n*-hexane/isopropanol 60:40; 0.6 mL/min.; 23 °C) *t*_R_ = 9.35 min [(*R*,*S*)-isomer], *t*_R_ = 14.04 min [(*S*,*S*)-isomer]. FTIR (cm^–1^): 1013 (ν_SO_).

#### (*R*,*R*)-1,3-Bis(propylsulfinyl)propane, **16(*R*,*R*)**

**16(*R*,*R*)** was prepared following the general procedure from bis-sulfinate **5(*S*,*S*)** (1.6 g, 1.97 mmol), 1 M PrMgCl solution (6.0 mL, 6.00 mmol, 300 mol %), and toluene (30 mL). The residue was purified by flash chromatography (CH_2_Cl_2_/MeOH, 30:1) to obtain bis-sulfoxide **16(*R*,*R*)** (188.0 mg, 0.84 mmol, 43% yield) as a yellow solid. Mp: 121–123 °C. [α]_D_^20^: −20.5 (*c* 1, MeOH). ^1^H NMR (500 MHz, MeOD): δ 2.95 (AB fragment of an ABX_2_ system, Δν = 50 Hz, *J*_AX_ = 7.3 Hz, *J*_BX_ = 7.9 Hz, *J*_AB_ = 13.3 Hz, 4H), 2.81 (t, *J* = 7.5 Hz, 4H), 2.25 (quint, *J* = 7.6 Hz, 2H), 1.87–1.76 (m, 4H), 1.11 (t, *J* = 7.4 Hz, 6H) ppm. ^13^C{^1^H} NMR (125 MHz, MeOD): δ 54.7, 51.2, 17.8, 17.4, 13.5 ppm. HRMS (ESI) *m*/*z*: [M + Na]^+^ calcd for C_9_H_20_O_2_NaS_2_ 247.0796; found 247.0797. 0:4:96 dr [(*S*,*S*):(*R*,*S*):(*R*,*R*)]. HPLC: ADH Chiracel column (*n*-hexane/isopropanol 60:40; 0.6 mL/min.; 23 °C) *t*_R_ = 7.77 min [(*R*,*R*)-isomer], *t*_R_ = 8.69 min [(*R*,*S*)-isomer].

#### (*S*,*S*)-1,3-Bis(isopropylsulfinyl)propane, **17(*S*,*S*)**

**17(*S*,*S*)** was prepared following the general procedure from bis-sulfinate **5(*S*,*S*)** (1.6 g, 1.96 mmol), 2 M *i*-PrMgBr solution (3.0 mL, 6.00 mmol, 300 mol %), and toluene (30 mL). The residue was purified by flash chromatography (CH_2_Cl_2_/MeOH, 30:1) to obtain bis-sulfoxide **17(*S*,*S*)** (241.0 mg, 1.07 mmol, 55% yield) as a yellow solid. Mp: 65–67 °C. [α]_D_^20^: −94.6 (*c* 1, MeOH). ^1^H NMR (500 MHz, MeOD): δ 3.00–2.84 (m, 6H), 2.26 (quint, *J* = 7.6 Hz, 2H), 1.31 (d, *J* = 6.9 Hz, 6H), 1.29 (d, *J* = 6.9 Hz, 6H) ppm. ^13^C{^1^H} NMR (125 MHz, MeOD): δ 51.5, 47.9, 18.6, 16.4, 14.5 ppm. HRMS (ESI) *m*/*z*: [M + H]^+^ calcd for C_9_H_21_O_2_S_2_ 225.0978; found 225.0977. 97:3:0 dr [(*S*,*S*):(*R*,*S*):(*R*,*R*)]. HPLC: ADH Chiracel column (*n*-hexane/isopropanol 70:30; 0.7 mL/min.; 23 °C) *t*_R_ = 7.35 min [(*S*,*S*)-isomer], *t*_R_ = 7.95 min [(*R*,*S*)-isomer].

#### (*S*,*S*)-1,3-Bis(*tert*-butylsulfinyl)propane, **18(*S*,*S*)**

**18(*S*,*S*)** was prepared following the general procedure from bis-sulfinate **5(*S*,*S*)** (2.4 g, 2.94 mmol), 1.7 M *t*-BuMgBr solution (5.2 mL, 8.82 mmol, 300 mol %), and toluene (40 mL). The residue was purified by flash chromatography (CH_2_Cl_2_/MeOH, 30:1) to obtain bis-sulfoxide **18(*S*,*S*)** (491.0 mg, 1.95 mmol, 66% yield) as a yellow solid. Mp: 103–105 °C. [α]_D_^20^: −136.8 (*c* 1, CHCl_3_). ^1^H NMR (500 MHz, MeOD): δ 2.82 (AB fragment of an ABX_2_ system, Δν = 100 Hz, *J*_AX_ = 7.2 Hz, *J*_BX_ = 8.0 Hz, *J*_AB_ = 13.1 Hz, 4H), 2.30 (quint, *J* = 7.6 Hz, 2H), 1.28 (s, 18H) ppm. ^13^C{^1^H} NMR (125 MHz, MeOD): δ 54.6, 45.0, 22.9, 20.2 ppm. HRMS (ESI) *m*/*z*: [M + Na]^+^ calcd for C_11_H_24_O_2_NaS_2_ 275.1117; found 275.1110. 91:9:0 dr [(*S*,*S*):(*R*,*S*):(*R*,*R*)]. HPLC: ADH Chiracel column (*n*-hexane/isopropanol 80:20; 0.3 mL/min.; 23 °C) *t*_R_ = 18.49 min [(*S*,*S*)-isomer], *t*_R_ = 21.14 min [(*R*,*S*)-isomer].

### General Procedure for Preparation of Enantiopure Vinyl Sulfoxides

To a solution of the corresponding DAG alkane- or arenesulfinate (100 mol %) in dry Et_2_O under argon atmosphere at −78 °C, 1 M vinylmagnesium bromide solution (150–160 mol %) was added dropwise. The mixture was stirred for 1 h and quenched with saturated aqueous NH_4_Cl solution, and the aqueous phase was extracted with CH_2_Cl_2_. The combined organic phases were washed with saturated aqueous NaCl solution and dried with anhydrous Na_2_SO_4_.

#### (*R*)-*p*-Tolyl Vinyl Sulfoxide, **25(*R*)**

**25(*R*)** was prepared following the general procedure from *p*-toluenesulfinate **22(*****S*****)** (783.3 mg, 2.66 mmol), 1 M vinylmagnesium bromide solution (4.0 mL, 3.99 mmol, 150 mol %), and Et_2_O (10 mL). The residue was purified by flash chromatography (CH_2_Cl_2_/EtOAc, 40:1) to obtain vinyl sulfoxide **25(*****R*****)** (385.7 mg, 2.32 mmol, 87%) as a volatile yellow liquid. [α]_D_^20^: +301.8 (*c* 1, CHCl_3_). ^1^H NMR (500 MHz, CDCl_3_): δ 7.50 (d, *J* = 8.2 Hz, 2H), 7.30 (d, *J* = 8.0 Hz, 2H), 6.57 (dd, *J* = 16.5 Hz, *J* = 9.6 Hz, 1H), 6.18 (d, *J* = 16.5 Hz, 1H), 5.87 (d, *J* = 9.6 Hz, 1H), 2.39 (s, 3H) ppm. ^13^C{^1^H} NMR (125 MHz, CDCl_3_): δ 143.2, 142.0, 140.3, 130.3, 125.0, 120.5, 21.5 ppm. HRMS (ESI) *m*/*z*: [M + Na]^+^ calcd for C_9_H_10_ONaS 189.0345; found 189.0341. 0:100 er [(*S*):(*R*)]. HPLC: OB Chiracel column (*n*-hexane/isopropanol 85:15; 1 mL/min.; 25 °C) *t*_R_ = 15.43 min [(*R*)-isomer].

#### (*S*)-*p*-Tolyl Vinyl Sulfoxide, **25(*S*)**

**25(*S*)** was prepared following the general procedure from scalemic (62% de) *p*-toluenesulfinate **32(*****R*****)** (1.6 g, 4.04 mmol), 1 M vinylmagnesium bromide solution (6.1 mL, 6.06 mmol, 150 mol %), and Et_2_O (40 mL). The residue was purified by flash chromatography (CH_2_Cl_2_/Et_2_O, 30:1) to obtain vinyl sulfoxide **25(*****S*****)** (606.6 mg, 3.65 mmol, 90%) as a volatile yellow liquid with similar physicochemical and spectroscopic characteristics to **25(*****R*****)**. HRMS (ESI) *m*/*z*: [M + Na]^+^ calcd for C_9_H_10_ONaS 189.0345; found 189.0342. 82:18 er [(*S*):(*R*)]. HPLC: OB Chiracel column (*n*-hexane/isopropanol 85:15; 1 mL/min.; 25 °C) *t*_R_ = 8.89 min [(*S*)-isomer], *t*_R_ = 13.73 min [(*R*)-isomer].

#### (*R*)-*tert*-Butyl Vinyl Sulfoxide, **26(*R*)**

**26(*R*)** was prepared following the general procedure from *tert-*butanesulfinate **23(***S***)** (1.6 g, 4.39 mmol), 1 M vinylmagnesium bromide solution (10.5 mL, 10.54 mmol, 240 mol %), and Et_2_O (24 mL) at 0 °C. The residue was dissolved in MeCN (5 mL), and water (2 mL) and TFA (0.17 mL, 2.20 mmol) were added. The reaction mixture was stirred at rt for 24 h and then neutralized with saturated aqueous NaHCO_3_ solution (4.7 mL). The aqueous phase was extracted with CH_2_Cl_2_; the combined organic phases were washed with saturated aqueous NaCl solution, dried with anhydrous Na_2_SO_4_, and evaporated under vacuum. The residue was purified by flash chromatography (CH_2_Cl_2_/acetone, 30:1) to obtain vinyl sulfoxide **26(*****R*****)** (376.2 mg, 2.85 mmol, 65%) as a volatile yellow liquid. [α]_D_^20^: +286.7 (*c* 1, CHCl_3_). ^1^H NMR (500 MHz, CDCl_3_): δ 6.56 (dd, *J* = 16.6 Hz, *J* = 9.9 Hz, 1H), 6.09 (d, *J* = 16.6 Hz, 1H), 6.02 (d, *J* = 9.9 Hz, 1H), 1.24 (s, 9H) ppm. ^13^C{^1^H} NMR (125 MHz, CDCl_3_): δ 136.8, 124.0, 54.9, 23.0 ppm. HRMS (ESI) *m*/*z*: [M + H]^+^ calcd for C_6_H_13_OS 133.0682; found 133.0679. 0:100 er [(*S*):(*R*)]. HPLC: AD Chiracel column (*n*-hexane/isopropanol 98:2; 0.5 mL/min.; 25 °C) *t*_R_ = 33.51 min [(*R*)-isomer].

#### (*S*)-*tert*-Butyl Vinyl Sulfoxide, **26(*S*)**

**26(*S*)** was prepared following the procedure described for vinyl sulfoxide **26(*****R*****)** from *tert-*butanesulfinate **23(*****R*****)** (1.5 g, 4.12 mmol), 1 M vinylmagnesium bromide solution (9.9 mL, 9.88 mmol, 240 mol %), and Et_2_O (24 mL). The residue was dissolved in MeCN (3 mL), and water (2 mL) and TFA (0.16 mL, 2.06 mmol) were added. The residue was purified by flash chromatography (CH_2_Cl_2_/acetone, 30:1) to obtain vinyl sulfoxide **26(*****S*****)** (396.0 mg, 3.00 mmol, 73%) as a volatile yellow liquid with similar physicochemical and spectroscopic characteristics to **26(*****R*****)**. [α]_D_^20^: −289.5 (*c* 1, CHCl_3_). HRMS (ESI) *m*/*z*: [M + Na]^+^ calcd for C_6_H_12_ONaS 155.0501; found 155.0499. 100:0 er [(*S*):(*R*)]. HPLC: AD Chiracel column (*n*-hexane/isopropanol 98:2; 0.5 mL/min.; 25 °C) *t*_R_ = 36.46 min [(*S*)-isomer].

#### (*S*)-Methyl Vinyl Sulfoxide, **27(*S*)**

**27(*S*)** was prepared following the general procedure from methanesulfinate **24(*****S*****)** (1.9 g, 5.78 mmol), 1 M vinylmagnesium bromide solution (8.7 mL, 8.67 mmol, 150 mol %), and Et_2_O (35 mL). The residue was purified by flash chromatography (EtOAc) to obtain vinyl sulfoxide **27(*****S*****)** (434.3 mg, 4.82 mmol, 83%) as a volatile yellow liquid. [α]_D_^20^: +278.4 (*c* 1, CHCl_3_). ^1^H NMR (500 MHz, CDCl_3_): δ 6.68 (dd, *J* = 16.5 Hz, *J* = 9.8 Hz, 1H), 6.12 (d, *J* = 16.4 Hz, 1H), 5.94 (d, *J* = 9.8 Hz, 1H), 2.61 (s, 3H) ppm. ^13^C{^1^H} NMR (125 MHz, CDCl_3_): δ 142.6, 121.5, 40.5 ppm. HRMS (ESI) *m*/*z*: [M + Na]^+^ calcd for C_3_H_6_ONaS 113.0032; found 113.0030.

#### (*R*)-Methyl Vinyl Sulfoxide, **27(*R*)**

**27(*R*)** was prepared following the general procedure from methanesulfinate **24(*****R*****)** (3.7 g, 11.56 mmol), 1 M vinylmagnesium bromide solution (17.3 mL, 17.30 mmol, 150 mol %), and Et_2_O (70 mL). The residue was purified by flash chromatography (EtOAc) to obtain vinyl sulfoxide **27(*****S*****)** (862.0 mg, 9.58 mmol, 83%) as a volatile yellow liquid with similar physicochemical and spectroscopic characteristics to **27(*****S*****)**. [α]_D_^20^: −304.5 (*c* 1, CHCl_3_). HRMS (ESI) *m*/*z*: [M + H]^+^ calcd for C_3_H_7_OS 91.0212; found 91.0213.

### General Procedure for Preparation of 1,5-Bis(sulfinyl)-3-thio Derivatives

A solution of the corresponding vinyl sulfoxide (200 mol %) and Na_2_S (100 mol %) in H_2_O was stirred at 55 °C overnight. Then, the reaction mixture was extracted with CH_2_Cl_2_ (3 × 20 mL). The combined organic phases were dried with anhydrous Na_2_SO_4_; the residue was evaporated under vacuum and purified by flash chromatography.

#### (*R*,*R*)-Bis[2-(*p*-tolylsulfinyl)ethyl] Sulfide, **28(*R*,*R*)**

**28(*R*,*R*)** was prepared following the general procedure from enantiopure (*R*)-*p*-tolyl vinyl sulfoxide, **25(*****R*****)** (385.7 mg, 1.45 mmol), and Na_2_S (174.0 mg, 0.72 mmol) in H_2_O (0.7 mL). The residue was purified by flash chromatography (CH_2_Cl_2_/MeOH, 90:1) to obtain bis-sulfoxide **28(*R*,*R*)** (157.6 mg, 0.43 mmol, 60%) as a colorless solid. Mp: 117–121 °C. [α]_D_^20^: +253.1 (*c* 1, CHCl_3_). ^1^H NMR (500 MHz, CDCl_3_): δ 7.49 (d, *J* = 8.2 Hz, 4H), 7.33 (d, *J* = 8.0 Hz, 4H), 3.04–2.89 (m, 6H), 2.72–2.64 (m, 2H), 2.42 (s, 6H) ppm. ^13^C{^1^H} NMR (125 MHz, CDCl_3_): δ 141.9, 139.9, 130.2, 124.1, 56.4, 24.4, 21.6 ppm. HRMS (ESI) *m*/*z*: [M + Na]^+^ calcd for C_18_H_22_O_2_NaS_3_ 389.0674; found 389.0667. 0:0:100 dr [(*S*,*S*):(*R*,*S*):(*R*,*R*)]. HPLC: ADH Chiracel column (*n*-hexane/isopropanol 60:40; 0.4 mL/min.; 25 °C) *t*_R_ = 32.75 min [(*R*,*R*)-isomer]. FTIR (cm^–1^): 1038 (ν_SO_).

#### (*S*,*S*)-Bis[2-(*p*-tolylsulfinyl)ethyl] Sulfide, **28(*S*,*S*)**

**28(*S*,*S*)** was prepared following the general procedure from scalemic (*S*)-*p*-tolyl vinyl sulfoxide, **25(*****S*****)** (200.0 mg, 1.20 mmol), and Na_2_S (144.6 mg, 0.60 mmol) in H_2_O (0.6 mL). The residue was purified by flash chromatography (CH_2_Cl_2_/MeOH, 60:1) to obtain bis-sulfoxide **28(*S*,*S*)** (173.0 mg, 0.47 mmol, 78%) as a colorless oil. ^1^H NMR (500 MHz, CDCl_3_): δ 7.50–7.48 (m, 4H), 7.33 (d, *J* = 8.0 Hz, 4H), 3.04–2.87 (m, 6H), 2.71–2.63 (m, 2H), 2.42 (s, 6H) ppm. ^13^C{^1^H} NMR (125 MHz, CDCl_3_): δ 142.0 (*meso*), 141.9 (*S,S*), 139.9 (*S,S*), 139.8 (*meso*), 130.2 ((*S,S*) + *meso*), 124.1 ((*S,S*) + *meso*), 56.4 (*S,S*), 56.3 (*meso*), 24.4 (*S,S*), 24.3 (*meso*), 21.6 ((*S,S*) + *meso*) ppm. HRMS (ESI) *m*/*z*: [M + Na]^+^ calcd for C_18_H_22_O_2_NaS_3_ 389.0674; found 389.0667. 66:31:3 dr [(*S*,*S*):(*R*,*S*):(*R*,*R*)]. HPLC: ADH Chiracel column (*n*-hexane/isopropanol 60:40; 0.4 mL/min.; 25 °C) *t*_R_ = 31.82 min [(*R*,*R*)-isomer], *t*_R_ = 35.00 min [(*R*,*S*)-isomer], *t*_R_ = 38.88 min [(*S*,*S*)-isomer].

#### (*R*,*R*)-Bis[2-(*tert*-butylsulfinyl)ethyl] Sulfide, **29(*R*,*R*)**

**29(*R*,*R*)** was prepared following the general procedure from (*R*)-*tert*-butyl vinyl sulfoxide, **26(*****R*****)** (333.0 mg, 2.52 mmol), and Na_2_S (302.6 mg, 1.26 mmol) in H_2_O (0.9 mL). The residue was purified by flash chromatography (EtOAc/MeOH, 30:1) to obtain bis-sulfoxide **29(*R*,*R*)** (185.9 mg, 0.62 mmol, 49%, 54% corrected yield) as a yellow solid. Mp: 56–58 °C. [α]_D_^20^: +213.9 (*c* 1, CHCl_3_). ^1^H NMR (500 MHz, MeOD): δ 3.10–3.05 (m, 2H), 3.02–2.94 (m, 4H), 2.86–2.81 (m, 2H), 1.29 (s, 18H) ppm. ^13^C{^1^H} NMR (125 MHz, MeOD): δ 54.6, 46.6, 26.7, 23.0 ppm. HRMS (ESI) *m*/*z*: [M + Na]^+^ calcd for C_12_H_26_O_2_NaS_3_ 321.0987; found 321.0979. 0:1:99 dr [(*S*,*S*):(*R*,*S*):(*R*,*R*)]. HPLC: IF Chiracel column (MeOH 100%; 1 mL/min.; 25 °C) *t*_R_ = 6.66 min [(*R*,*S*)-isomer], *t*_R_ = 7.17 min [(*R*,*R*)-isomer]. FTIR (cm^–1^): 1033 (ν_SO_).

#### (*S*,*S*)-Bis[2-(*tert*-butylsulfinyl)ethyl] Sulfide, **29(*S*,*S*)**

**29(*S*,*S*)** was prepared following the general procedure from (*S*)-*tert*-butyl vinyl sulfoxide, **26(*****S*****)** (349.1 mg, 2.64 mmol), and Na_2_S (317.0 mg, 1.32 mmol) in H_2_O (0.9 mL). The residue was purified by flash chromatography (EtOAc/MeOH, 30:1) to obtain bis-sulfoxide **29(*S*,*S*)** (175.0 mg, 0.59 mmol, 45%, 52% corrected yield) as a yellow solid with similar physicochemical and spectroscopic characteristics to **29(*R*,*R***). Mp: 56–58 °C. [α]_D_^20^: −215.6 (*c* 1, CHCl_3_). HRMS (ESI) *m*/*z*: [M + Na]^+^ calcd for C_12_H_26_O_2_NaS_3_ 321.0987; found 321.0979. 99:1:0 dr [(*S*,*S*):(*R*,*S*):(*R*,*R*)]. HPLC: IF Chiracel column (MeOH 100%; 1 mL/min.; 25 °C) *t*_R_ = 5.94 min [(*S*,*S*)-isomer], *t*_R_ = 6.69 min [(*R*,*S*)-isomer]. FTIR (cm^–1^): 1033 (ν_SO_).

#### (*S*,*S*)-Bis[2-(methylsulfinyl)ethyl] Sulfide, **30(*S*,*S*)**

**30(*S*,*S*)** was prepared following the general procedure from (*S*)-methyl vinyl sulfoxide, **27(*****S*****)** (148.0 mg, 1.64 mmol), and Na_2_S (197.4 mg, 0.82 mmol) in H_2_O (0.5 mL). The residue was purified by flash chromatography (CH_2_Cl_2_/MeOH, 20:1) to obtain bis-sulfoxide **30(*S*,*S*)** (101.4 mg, 0.47 mmol, 57%) as a white solid. Mp: 89–91 °C. [α]_D_^20^: +217.9 (*c* 1, CHCl_3_). ^1^H NMR (500 MHz, DMSO-*d*_6_): δ 3.09–3.02 (m, 2H), 2.95–2.82 (m, 6H), 2.58 (s, 6H) ppm. ^13^C{^1^H} NMR (125 MHz, DMSO-*d*_6_): δ 52.8, 37.8, 23.8 ppm. HRMS (ESI) *m*/*z*: [M + Na]^+^ calcd for C_6_H_14_O_2_NaS_3_ 237.0048; found 237.0045. 95:5:0 dr [(*S*,*S*):(*R*,*S*):(*R*,*R*)]. HPLC: IF Chiracel column (MeOH 100%; 1 mL/min.; 25 °C) *t*_R_ = 8.24 min [(*R*,*S*)-isomer], *t*_R_ = 9.37 min [(*S*,*S*)-isomer]. FTIR (cm^–1^): 1015 (ν_SO_).

#### (*S*,*S*)-Bis[2-(methylsulfinyl)ethyl] Sulfide, **30(*R*,*R*)**

**30(*R*,*R*)** was prepared following the general procedure from (*R*)-methyl vinyl sulfoxide, **27(*****R*****)** (769.0 mg, 8.54 mmol), and Na_2_S (1.0 g, 4.27 mmol) in H_2_O (2 mL). The residue was purified by flash chromatography (CH_2_Cl_2_/MeOH, 20:1) to obtain bis-sulfoxide **30(*R*,*R*)** (481.3 mg, 2.25 mmol, 53%) as a white solid with similar physicochemical and spectroscopic characteristics to **30(***S*,*S***)**. [α]_D_^20^: −217.3 (*c* 1, CHCl_3_). HRMS (ESI) *m*/*z*: [M + Na]^+^ calcd for C_6_H_14_O_2_NaS_3_ 237.0048; found 237.0048. 0:3:97 dr [(*S*,*S*):(*R*,*S*):(*R*,*R*)]. HPLC: IF Chiracel column (MeOH 100%; 1 mL/min.; 25 °C) *t*_R_ = 7.70 min [(*R*,*R*)-isomer], *t*_R_ = 8.05 min [(*R*,*S*)-isomer]. FTIR (cm^–1^): 1016 (ν_SO_).

### Synthesis of 1,3,5-Tris-sulfoxides

#### General Procedure

To a solution of the corresponding bis[2-(alkyl or arylsulfinyl)ethyl] sulfide (100 mol %) in CH_2_Cl_2_ at −78 °C, a solution of *m*-CPBA (100 mol %) in CHCl_3_ was added dropwise. The mixture was stirred 30 min at −78 °C and quenched with a saturated aqueous NaHCO_3_ solution, and the aqueous phase was extracted with CH_2_Cl_2_. The combined organic phases were dried with anhydrous Na_2_SO_4_, and the residue was purified by flash chromatography. In the case of the alkylsulfinyl derivatives, **34** and **35**, the work up was different: After the reaction mixture was stirred for 30 min at −78 °C, the reaction was warmed to rt and the solvent was evaporated under vacuum. The residue was purified by flash chromatography.

##### (*R*,*R*)-Bis[2-(*p*-tolylsulfinyl)ethyl] Sulfoxide, **33(*R*,*R*)**

**33(*R*,*R*)** was prepared following the general procedure from (*R*,*R*)-bis[2-(*p*-tolylsulfinyl)ethyl] sulfide, **28(*R*,*R*)** (139.0 mg, 0.38 mmol), and *m*-CPBA 70% (93.4 mg, 0.38 mmol) in CHCl_3_ (3 mL) and CH_2_Cl_2_ (1.3 mL). This was purified by flash chromatography (CH_2_Cl_2_/MeOH, 50:1) to obtain bis-sulfoxide **33(*R*,*R*)** (99.9 mg, 0.28 mmol, 75%) as a colorless solid. Mp: 73–76 °C. [α]_D_^20^: +254.0 (*c* 1, CHCl_3_). ^1^H NMR (500 MHz, MeOD): δ 7.58 (dd, *J* = 1.9 Hz, *J* = 8.3 Hz, 4H), 7.44–7.42 (m, 4H), 3.39 (ddd, *J* = 13.1 Hz, *J* = 10.2 Hz, *J* = 5.3 Hz, 1H), 3.35 (ddd, *J* = 2.4 Hz, *J* = 13.3 Hz, *J* = 5.1 Hz, 1H), 3.26–3.04 (m, 4H), 2.97 (ddd, *J* = 13.1 Hz, *J* = 10.4 Hz, *J* = 5.3 Hz, 1H), 2.83 (ddd, *J* = 13.3 Hz, *J* = 10.1 Hz, *J* = 4.7 Hz, 1H), 2.43 (s, 6H) ppm. ^13^C{^1^H} NMR (125 MHz, MeOD): δ 143.9, 139.7 (2), 131.4, 125.5, 125.4, 44.5, 44.4, 21.4 ppm. HRMS (ESI) *m*/*z*: [M + Na]^+^ calcd for C_18_H_22_O_3_NaS_3_ 405.0623; found 405.0618. FTIR (cm^–1^): 1035 (ν_SO_).

##### (*R*,*R*)-Bis[2-(*tert*-butylsulfinyl)ethyl] Sulfoxide, **34(*R*,*R*)**

**34(*R*,*R*)** was prepared following the general procedure from (*R*,*R*)-bis[2-(*tert*-butylsulfinyl)ethyl] sulfide, **29(*R*,*R*)** (90.0 mg, 0.30 mmol), and *m*-CPBA 75% (69.5 mg, 0.30 mmol) in CHCl_3_ (3 mL) and CH_2_Cl_2_ (2 mL). This was purified by flash chromatography (CH_2_Cl_2_/MeOH, 30:1) to obtain bis-sulfoxide **34(*R*,*R*)** (78.7 mg, 0.25 mmol, 84%, quant. corrected yield) as a colorless solid. Mp: 90–93 °C. [α]_D_^20^: +159.3 (*c* 1, MeOH). ^1^H NMR (500 MHz, MeOD): δ 3.43–3.25 (m, 4H), 3.19–3.09 (m, 2H), 3.03–2.97 (m, 2H), 1.31 (s, 18H) ppm. ^13^C{^1^H} NMR (125 MHz, MeOD): δ 55.5, 55.4, 46.8 (2), 39.4, 39.3, 22.8 ppm. HRMS (ESI) *m*/*z*: [M + H]^+^ calcd for C_12_H_27_O_3_S_3_ 315.1117; found 315.1111. FTIR (cm^–1^): 1032 and 1018 (ν_SO_).

##### (*S*,*S*)-Bis[2-(*tert*-butylsulfinyl)ethyl] Sulfoxide, **34(*S*,*S*)**

**34(*S*,*S*)** was prepared following the general procedure from (*S*,*S*)-bis[2-(*tert*-butylsulfinyl)ethyl] sulfide, **29(*S*,*S*)** (100.0 mg, 0.34 mmol), and *m*-CPBA 75% (77.1 mg, 0.34 mmol) in CHCl_3_ (3 mL) and CH_2_Cl_2_ (2 mL). This was purified by flash chromatography (CH_2_Cl_2_/MeOH, 30:1) to obtain bis-sulfoxide **34(*S*,*S*)** (97.0 mg, 0.31 mmol, 92%, quant. corrected yield) as a colorless solid with similar physicochemical and spectroscopic characteristics to **34(*R*,*R***). Mp: 90–93 °C. [α]_D_^20^: −158.1 (*c* 1, MeOH). HRMS (ESI) *m*/*z*: [M + Na]^+^ calcd for C_12_H_26_O_3_NaS_3_ 337.0936; found 337.0930. FTIR (cm^–1^): 1032 and 1018 (ν_SO_).

##### (*S*,*S*)-Bis[2-(methylsulfinyl)ethyl] Sulfoxide, **35(*S*,*S*)**

**35(*S*,*S*)** was prepared following the general procedure from (*S*,*S*)-bis[2-(methylsulfinyl)ethyl] sulfide, **30(*S*,*S*)** (100.0 mg, 0.47 mmol), and *m*-CPBA 75% (107.5 mg, 0.47 mmol) in CHCl_3_ (3 mL) and CH_2_Cl_2_ (2 mL). This was purified by flash chromatography (CH_2_Cl_2_/MeOH, 20:1) to obtain bis-sulfoxide **35(*S*,*S***)**** (98.6 mg, 0.43 mmol, 92%) as a colorless solid. Mp: 167–169 °C. [α]_D_^20^: +113.3 (*c* 1, MeOH). ^1^H NMR (500 MHz, MeOD): δ 3.41–3.23 (m, 6H), 3.19–3.13 (m, 2H), 2.73 (s, 6H) ppm. ^13^C{^1^H} NMR (125 MHz, MeOD): δ 46.8, 46.6, 45.1 (2), 38.4, 38.3 ppm. HRMS (ESI) *m*/*z*: [M + Na]^+^ calcd for C_6_H_14_O_3_NaS_3_ 252.9997; found 252.9997. FTIR (cm^–1^): 1010 (ν_SO_).

#### General Procedure for the Synthesis of Pd(II) Complexes

A solution of the corresponding bis(sulfinyl)propane ligand (100 mol %) and Pd(TFA)_2_ (100 mol %) in dry CH_2_Cl_2_ under argon atmosphere was stirred at rt. After 24 h, the solvent was evaporated under reduced pressure to obtain the desired compounds without further purification.

##### *cis*-[(*R*,*R*)-1,3-Bis(benzylsulfinyl)propane]palladium(II) Trifluoroacetate, **12(*S*,*S*)·PdTFA**_**2**_

**12(*S*,*S*)·PdTFA**_**2**_ was prepared following the general procedure from (*S*,*S*)-1,3-bis(benzylsulfinyl)propane, **12(*S*,*S*)** (25.0 mg, 78.01 mmol, 100 mol %), Pd(TFA)_2_ (25.9 mg, 78.01 mmol, 100 mol %), and CH_2_Cl_2_ (1.5 mL). After evaporation of the solvent, **12(*S*,*S*)·PdTFA**_**2**_ (50.9 mg, 78.01 mmol, quant.) was obtained as a dark red solid. Mp: decomposes before melting. ^1^H NMR (500 MHz, CDCl_3_): δ 7.48–7.45 (m, 2H), 7.43–7.40 (m, 4H), 7.33 (d, *J* = 7.1 Hz, 4H), 5.43 (d, *J* = 14.5 Hz, 2H), 5.21 (d, *J* = 14.5 Hz, 2H), 3.47 (bs, 4H), 2.25–2.23 (m, 2H) ppm. ^13^C{^1^H} NMR (125 MHz, CDCl_3_): δ 162.1 (q, *J* = 37.6 Hz), 132.0, 130.4, 129.6, 124.5, 115.4 (q, *J* = 291.0 Hz), 57.9, 45.8, 15.9 ppm. ^19^F NMR (470 MHz, CDCl_3_): δ −74.1 ppm. FTIR (cm^–1^): 1178 and 1145 (ν_SO-Pd_).

##### *cis*-[(*R*,*R*)-1,3-Bis(phenylsulfinyl)propane]palladium(II) Trifluoroacetate, **8(*S*,*S*)·PdTFA**_**2**_

**8(*S*,*S*)·PdTFA**_**2**_ was prepared following the general procedure from (*S*,*S*)-1,3-bis(phenylsulfinyl)propane, **8(*S*,*S*)** (22.8 mg, 77.97 mmol, 100 mol %), Pd(TFA)_2_ (25.9 mg, 77.97 mmol, 100 mol %), and CH_2_Cl_2_ (1.5 mL). After evaporation of the solvent, **8(*S*,*S*)·PdTFA**_**2**_ (48.7 mg, 77.97 mmol, quant.) was obtained as a dark orange solid. Mp: decomposes before melting. ^1^H NMR (500 MHz, CDCl_3_): δ 8.43 (d, *J* = 6.9 Hz, 4H), 7.81–7.74 (m, 6H), 4.12–4.07 (bs, 2H), 4.01–3.96 (m, 2H), 2.36–2.32 (m, 2H) ppm. ^13^C{^1^H} NMR (125 MHz, CDCl_3_): δ 161.5 (q, *J* = 37.7 Hz), 137.4, 135.0, 130.3, 126.9, 115.1 (q, *J* = 289.6 Hz), 55.1, 16.1 ppm. ^19^F NMR (470 MHz, CDCl_3_): δ −74.0 ppm. FTIR (cm^–1^): 1177 and 1136 (ν_SO-Pd_).

##### *cis*-[(*S*,*S*)-1,3-Bis(propylsulfinyl)propane]palladium(II) Trifluoroacetate, **16(*R*,*R*)·PdTFA**_**2**_

**16(*R*,*R*)·PdTFA**_**2**_ was prepared following the general procedure from (*R*,*R*)-1,3-bis(propylsulfinyl)propane, **16(*R*,*R*)** (27.0 mg, 120.33 mmol, 100 mol %), Pd(TFA)_2_ (40.0 mg, 120.33 mmol, 100 mol %), and CH_2_Cl_2_ (1.5 mL). After evaporation of the solvent, **16(*R*,*R*)·PdTFA**_**2**_ (67.0 mg, 120.33 mmol, quant.) was obtained as a dark orange solid. Mp: decomposes before melting. ^1^H NMR (500 MHz, CDCl_3_): δ 4.01 (ddd, *J* = 4.7 Hz, *J* = 11.4 Hz, *J* = 14.3 Hz, 2H), 3.86–3.77 (m, 4H), 3.53 (ddd, *J* = 5.1 Hz, *J* = 11.2 Hz, *J* = 14.4 Hz, 2H), 5.52–5.47 (m, 2H), 2.11–2.04 (m, 2H), 1.86–1.79 (m, 2H), 1.14 (t, *J* = 7.4 Hz, 6H) ppm. ^13^C{^1^H} NMR (125 MHz, CDCl_3_): δ 161.9 (q, *J* = 38.2 Hz), 115.3 (q, *J* = 289.4 Hz), 54.2, 48.6, 16.5, 14.7, 12.9 ppm. ^19^F NMR (470 MHz, CDCl_3_): δ −74.3 ppm. FTIR (cm^–1^): 1178 and 1138 (ν_SO-Pd_).

#### General Procedure for the Synthesis of Ru(II) Complexes

A solution of RuCl_3_·3H_2_O (100–130 mol %) in dry MeOH under argon atmosphere was refluxed until the solution became light orange (4 h). Then, a solution of the corresponding ligand (100–200 mol %) in dry MeOH was added dropwise and refluxed until the starting material was consumed.

##### *trans*-Dichlorobis[(*R*,*R*)-1,3-bis(methylsulfinyl)propane]ruthenium(II), **36(*R*,*R*)**

**36(*R*,*R*)** was prepared following the general procedure from bis-sulfoxide ligand **14(*S*,*S*)** (100.0 mg, 0.59 mmol, 200 mol %) and RuCl_3_·3H_2_O (77.7 mg, 0.30 mmol, 100 mol %) in MeOH (14 mL). After 24 h, the complex was precipitated with Et_2_O and filtered to give **36(*R*,*R***)**** (103.2 mg, 0.20 mmol, 68%) as a yellow solid. Mp: decomposes before melting. [α]_546_^20^: +1.9 (*c* 1, MeOD). ^1^H NMR (500 MHz, MeOD): δ 3.71 (dt, *J* = 5.8 Hz, *J* = 6.5 Hz, 8H), 3.34 (s, 12H), 2.42–2.38 (m, 4H) ppm. ^13^C{^1^H} NMR (125 MHz, MeOD): δ 54.4, 44.6, 18.6 ppm. HRMS (ESI) *m*/*z*: [M + Na]^+^ calcd for C_10_H_24_O_4_Cl_2_NaRuS_4_ 530.8870; found 530.8855. FTIR (cm^–1^): 1083 (ν_SO–Ru_).

##### *trans*-Dichlorobis[(*S*,*S*)-1,3-bis(methylsulfinyl)propane]ruthenium(II), **36(*S*,*S*)**

**36(*S*,*S*)** was prepared following the general procedure from bis-sulfoxide ligand **14(*R*,*R*)** (200.0 mg, 1.19 mmol, 200 mol %) and RuCl_3_·3H_2_O (155.4 mg, 0.59 mmol, 100 mol %) in MeOH (27 mL). After 24 h, the reaction mixture was evaporated to give **36(*S*,*S*)** (302.0 mg, 0.59 mmol, quant. yield) as a yellow solid with similar physicochemical and spectroscopic characteristics to **36(*R*,*R*)**. [α]_546_^20^: −1.5 (*c* 1, MeOD). HRMS (ESI) *m*/*z*: [M + Na]^+^ calcd for C_10_H_24_O_4_Cl_2_NaRuS_4_ 530.8870; found 530.8855. FTIR (cm^–1^): 1083 (ν_SO–Ru_).

##### *trans*-Dichlorobis[(*R*,*R*)-1,3-bis(ethylsulfinyl)propane]ruthenium(II), **37(*R*,*R*)**

**37(*R*,*R*)** was prepared following the general procedure from bis-sulfoxide ligand **15(*S*,*S*)** (146.0 mg, 0.74 mmol, 200 mol %) and RuCl_3_·3H_2_O (126.4 mg, 0.48 mmol, 130 mol %) in MeOH (29 mL). After 24 h, the complex was precipitated with Et_2_O and filtered to give **37(*R*,*R*)** (88.8 mg, 0.16 mmol, 42%) as a yellow solid. Mp: decomposes before melting. [α]_546_^20^: +6.1 (*c* 1, MeOD). ^1^H NMR (500 MHz, MeOD): δ 3.94 (dq, *J* = 7.4 Hz, *J* = 14.7 Hz, 4H), 3.66 (dt, *J* = 6.0 Hz, *J* = 2.2 Hz, 8H), 3.39 (dq, *J* = 7.3 Hz, *J* = 14.6 Hz, 4H), 2.32–2.27 (m, 4H), 1.39 (t, *J* = 7.4 Hz, 4H) ppm. ^13^C{^1^H} NMR (125 MHz, MeOD): δ 50.2, 17.7, 5.9 ppm. HRMS (ESI) *m*/*z*: [M + Na]^+^ calcd for C_14_H_32_O_4_Cl_2_NaRuS_4_ 586.9496; found 586.9482. FTIR (cm^–1^): 1081 (ν_SO–Ru_).

##### *trans*-Dichlorobis[(*S*,*S*)-1,3-bis(ethylsulfinyl)propane]ruthenium(II), **37(*S*,*S*)**

**37(*S*,*S*)** was prepared following the general procedure from bis-sulfoxide ligand **15(*R*,*R*)** (46.7 mg, 0.24 mmol, 200 mol %) and RuCl_3_·3H_2_O (41.0 mg, 0.16 mmol, 130 mol %) in MeOH (10 mL). After 24 h, the complex was precipitated with Et_2_O and filtered to give **37(*S*,*S*)** (31.3 mg, 0.06 mmol, 47%) as a yellow solid with similar physicochemical and spectroscopic characteristics to **37(***R*,*R***)**. [α]_546_^20^: −8.8 (*c* 1, MeOD). HRMS (ESI) *m*/*z*: [M + Na]^+^ calcd for C_14_H_32_O_4_Cl_2_NaRuS_4_ 586.9496; found 586.9482. FTIR (cm^–1^): 1076 (ν_SO–Ru_).

##### *mer*-[28(*R*,*R*)·RuCl_2_]_2_, **38(*S*,*S*)**

**38(*S*,*S*)** was prepared following the general procedure from bis-sulfoxide ligand **28(*R*,*R*)** (50.0 mg, 0.14 mmol, 100 mol %) and RuCl_3_·3H_2_O (35.7 mg, 0.14 mmol, 100 mol %) in MeOH (11 mL). After 24 h, the complex was purified by flash chromatography (CH_2_Cl_2_/MeOH, 30:1) to give **38(*S*,*S*)** (41.3 mg, 0.04 mmol, 54%) as a yellow solid. Mp: decomposes before melting. [α]_546_^20^: −125.3 (*c* 1, MeOD). ^1^H NMR (500 MHz, MeOD): δ 8.23 (d, *J* = 8.4 Hz, 4H), 8.18 (d, *J* = 8.4 Hz, 4H), 7.38 (d, *J* = 8.2 Hz, 4H), 7.30 (d, *J* = 8.1 Hz, 4H), 4.03 (dd, *J* = 3.7 Hz, *J* = 13.0 Hz, 2H), 3.89 (ddd, *J* = 6.5 Hz, *J* = 12.4 Hz, *J* = 14.2 Hz, 2H), 3.82 (ddd, *J* = 5.2 Hz, *J* = 13.0 Hz, *J* = 14.1 Hz, 2H), 3.46–3.40 (m, 4H), 3.35–3.31 (m, 2H), 3.08 (ddd, *J* = 4.4 Hz, *J* = 14.4 Hz, *J* = 14.4 Hz, 2H), 2.56 (dd, *J* = 5.3 Hz, *J* = 13.5 Hz, 2H), 2.44 (s, 6H), 2.41 (s, 6H) ppm. ^13^C{^1^H} NMR (125 MHz, MeOD): δ 144.8, 144.6, 139.9, 139.7, 130.3, 129.8, 128.9, 66.3, 62.0, 33.4, 28.4, 21.4 ppm. HRMS (ESI) *m*/*z*: [M + Na]^+^ calcd for C_36_H_44_O_4_Cl_4_NaRu_2_S_6_ 1098.8297; found 1098.8282. FTIR (cm^–1^): 1065 (ν_SO–Ru_).

##### *trans*-Dichloro{[(*S*,*S*)-Bis(2-(*p*-tolylsulfinyl)ethyl] sulfoxide-κ^3^S}(methanol)ruthenium(II), **39(*S*,*S*)**

**39(*S*,*S*)** was prepared following the general procedure from tris-sulfoxide ligand **33(*R*,*R*)** (80.0 mg, 0.21 mmol, 100 mol %) and RuCl_3_·3H_2_O (54.6 mg, 0.21 mmol, 100 mol %) in MeOH (10 mL). After 24 h, the complex was precipitated with Et_2_O and filtered to give **39(*S*,*S*)** (68.9 mg, 0.14 mmol, 58%) as a yellow solid. Mp: decomposes before melting. [α]_546_^20^: −69.2 (*c* 1, MeOD). ^1^H NMR (500 MHz, MeOD): δ 8.24 (d, *J* = 8.5 Hz, 2H), 8.09 (d, *J* = 8.5 Hz, 2H), 7.41 (d, *J* = 8.2 Hz, 2H), 7.30 (d, *J* = 8.2 Hz, 2H), 4.69 (dd, *J* = 4.3 Hz, *J* = 13.8 Hz, 1H), 4.47 (ddd, *J* = 7.2 Hz, *J* = 12.3 Hz, *J* = 13.6 Hz, 1H), 4.02–3.94 (m, 2H), 3.87–3.81 (m, 2H), 3.57–3.47 (m, 2H), 2.45 (s, 3H), 2.41 (s, 3H) ppm. ^13^C{^1^H} NMR (125 MHz, MeOD): δ 145.2 (2), 139.2, 138.9, 130.4, 129.9, 129.6, 129.0, 63.2, 58.0, 57.0, 55.7, 21.5, 21.4 ppm. HRMS (ESI) *m*/*z*: [M + Na]^+^ calcd for C_19_H_26_O_4_Cl_2_NaRuS_3_ 608.9306; found 608.9293. FTIR (cm^–1^): 1065 (ν_SO–Ru_).

## Data Availability

The data underlying this study are available in the published article and its Supporting Information.

## Supporting Information Available

Diastereomeric excess determination of 1,3-bisulfinates, HPLC chromatograms of selected compounds, experimental procedures, characterization data for selected compounds including ^1^H NMR, ^13^C{^1^H} NMR, ^19^F NMR, and 2D NMR (COSY, NOESY, and HSQC) and crystallographic data for **30(***R*,*R***)** (CCDC 2309479) (PDF)
FAIR data, including the primary NMR FID files, for compounds **2**–**5**, **8**–**18**, **25**–**30**, **33**–**39**, **12(***S*,*S***)·Pd(TFA)**_**2**_, **8(***S*,*S***)·Pd(TFA)**_**2**_, and **16(***R*,*R***)·Pd(TFA)**_**2**_ (ZIP)
