# Supplementary material for: Stereoselective Synthesis of Chiral C2-Symmetric 1,3- and 1,5-Bis-Sulfoxides Guided by the Horeau Principle: Understanding the Influence of the Carbon Chain Nature in Its Ability for Metal Coordination
Source: J Org Chem. 2024 Oct 2;89(20):15048–61. doi: 10.1021/acs.joc.4c01729 (PMC11494659; doi:10.1021/acs.joc.4c01729)
Supplement: Supplementary file 1 — jo4c01729_si_001.pdf [file jo4c01729_si_001.pdf]

Stereoselective Synthesis of Chiral C_2-Symmetric 1,3- and 1,5-Bis-sulfoxides Guided by Horeau Principle: Understanding the Influence of the Carbon Chain Nature in its Ability for Metal Coordination.

Nazaret Moreno-Rodríguez,[a] L. Alberto Prieto,[a] Victoria Valdivia,[a] Rocío Recio [a]* and Inmaculada Fernández[a]*

[a]Departamento de Química Orgánica y Farmacéutica. Facultad de Farmacia. Universidad de Sevilla. C/ Profesor García González, 2, 41012, Sevilla, Spain. inmaff@us.es

Supporting Information

Table of contents

Diastereomeric excess determination of 1,3-bis-sulfinates. **S2**

Experimental data of 1,3-bis(sulfinyl)propanes. **S4**

Experimental data of allylation of *N*-(benzoyl)*iso*butylhydrazone **S9**

Experimental data of vinyl sulfoxides. **S11**

Experimental data of 1,5-bis(sulfinyl)-3-thioderivatives. **S12**

Figures of regioselective oxidation of 1,5-bis(sulfinyl)-3-thioderivatives **S13**

Figures of palladium(II) and ruthenium(II) complexes formation **S14**

HPLC chromatograms of selected compounds. **S16**

^{1}H NMR, $^{13}C\{^{1}H\}$ NMR and ^{19}F NMR of selected compounds. **S25**

2D NMR of selected compounds. **S151**

X-ray Structural Analysis for 30(*R*,*R*) (CDCC 2309479). **S155**

References **S158**

Diastereomeric excess determination of 1,3-bis-sulfinates:

Due to the superposition of the diastereomeric bis-sulfinate signals, the ^{1}H NMR spectrum of the crude mixture in $CDCl_3$ did not allow us to determine the diastereomeric rate. Fortunately, variation of solvent revealed that deuterated benzene was the best for the determination of the stereochemical outcome of the reaction, with clean separation of the anomeric and H-3 protons for the species present in the mixture.

Considering the C_2 symmetry of bis-sulfinate with identical configuration of sulfinylic sulfur, the two anomeric protons of the glucose fragment must appear as the only signal. On the contrary, bis-sulfinate esters with opposite configurations in both sulfurs can originate two different signals in the ^{1}H NMR spectrum, one for each anomeric proton.

In Figure S1, the spectrum zone corresponding to anomeric and H-3 protons of the sugar fragment is represented for each bis-sulfinate ester. As can be seen in this figure, the doublet at 5.93 ppm can be assigned to the **5(*R*,*R*)** anomeric proton, while the doublet at 5.90 ppm can be assigned to the **5(*S*,*S*)** anomeric proton. Finally, bis-sulfinate **5(*R*,*S*)**, without C_2 symmetry, has two different anomeric protons as two doublets at 5.91 and 5.87 ppm. Unlike ethane 1,2-bis-sulfinate esters, these doublets are not far enough apart to be able to calculate the diastereomeric excess, and it is necessary to calculate it using the H-3 proton signals as well.

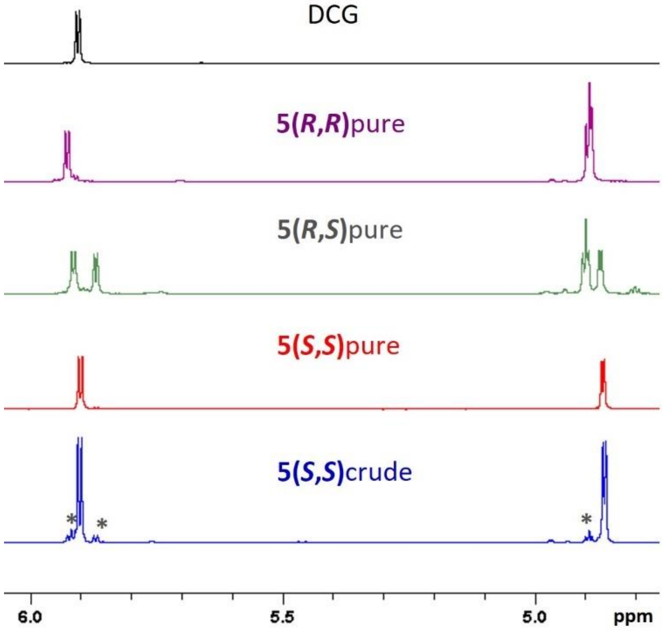

Figure S1. Analysis of the anomeric and 3-H proton signals from DCG fragment in bis-sulfinate **5** ^{1}H NMR spectrum (500 MHz, C_6D_6).

To calculate the ratio between bis-sulfinate **5(*S*,*S*)** and **5(*R*,*S*)** in the crude (*S*,*S*) bis-sulfinate mixture, the spectrum must be integrated as shown in Figure S2. Starting with H-3 signals, it can be observed that two protons of **5(*R*,*S*)** have a x integral value (0.2075), therefore, one proton of (*R*,*S*) will have a $x/2$ integral value (0.1038). On the other hand, two protons of **5(*S*,*S*)** can be

calculated as $(y - x/2)$ (1.8962). Thus, the percentage of **5(*R,S*)** presents in the mixture can be calculated as $x/(y + x/2) \cdot 100$ (10%).

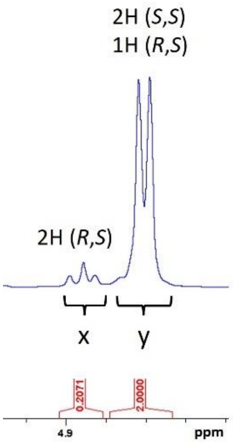

Figure S2. Analysis of 3-H proton signal for the crude (*S*,*S*) bis-sulfinate **5(*S,S*)** mixture in ^{1}H NMR spectrum (500 MHz, C_6D_6).

In the case of the crude (*R*,*R*)-bis-sulfinate mixture, it is easier to calculate the ratio between bis-sulfinate **5(*R,R*)**, **5(*R,S*)** and **5(*S,S*)** in the alkyl chain proton signals despite the anomeric and H-3 proton signals. In the same way as in the case of anomeric protons, the C_2-symmetric bis-sulfinate will have equivalent protons in the chain and the bis-sulfinate ester with opposite configuration in both sulfurs will have diastereotopic protons in the chain.

In Figure S3, the spectrum zone corresponding to alkyl chain protons is represented for each bis-sulfinate ester. As can be seen in this figure, the triplet at 2.56 ppm and the quintet at 2.10 ppm can be assigned to **5(*R,R*)** chain protons, while the triplet at 2.39 ppm and the quintet at 1.89 ppm can be assigned to **5(*S,S*)** chain protons. Finally, bis-sulfinate **5(*R,S*)** chain protons, without C_2 symmetry, appear as two triplets at 2.51 ppm and 2.44 ppm, respectively, and a multiplet between 2.06-1.94 ppm.

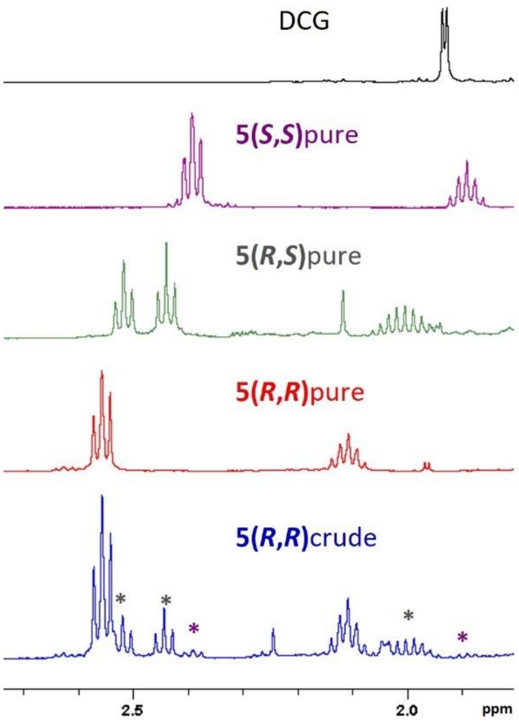

Figure S3. Analysis of chain proton signals in bis-sulfinate **5** ^{1}H NMR (500 MHz, C_6D_6) spectrum.

To calculate the ratio between bis-sulfinate **5(*R*,*R*)**, **5(*R*,*S*)** and **5(*S*,*S*)** in the crude (*R*,*R*)-bis-sulfinate mixture, the spectrum must be integrated as shown in Figure S4. It can be observed that two protons of **5(*R*,*S*)** have a z integral value (0.7716), therefore, two protons of (*S*,*S*) will have a $y/2$ integral value (0.1272) and two protons of **5(*R*,*R*)** have x integral value (2.0000). Thus, the percentage of **5(*R*,*S*)** present in the mixture can be calculated as $z/(z + x + y/2)\ \cdot 100$ (26%), the percentage of **5(*S*,*S*)** present in the mixture can be calculated as $(y/2)/(z + x + y/2)\ \cdot 100$ (4%).

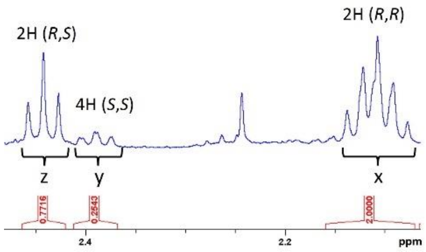

Figure S4. Analysis of chain proton signals for the crude (*R*,*R*)-bis-sulfinate **5(*R*,*R*)** mixture in ^{1}H NMR spectrum (500 MHz, C_6D_6).

Non stereoselective synthesis of 1,3-bis(sulfinyl)propanes.

To a solution of propane-1,3-bis(sulfinyl) chloride **4** (500.0 mg, 2.39 mmol, 100 mol%) in toluene (10 mL), RMgBr (R = alkyl or aryl, 7.17 mmol, 300 mol%) was added dropwise at

0 °C. The mixture was stirred for 2 h, quenched with saturated aqueous NH_4Cl solution, extracted with CH_2Cl_2, and dried with anhydrous Na_2SO_4. The residue was purified by flash chromatography. In the case of 1,3-bis(alkylsulfinyl)propanes **14-18**, the work up was different: After the reaction mixture was stirred for 1 h, 100 mol% of TFA was added and the solvent removed under vacuo. The residue was dissolved in MeOH and treated with mixed bed resin (Sigma TMD-8; 1:1 mixture of strong cation and anion exchange resin) to remove the remaining salts. After that, it was purified by column chromatography on silica gel.

(S,S)/(R,R)/(R,S)-1,3-Bis(phenylsulfinyl)propane, **8(*rac+meso*)**
It was prepared following the general procedure and was purified by flash chromatography (TBME/MeOH, 20:1) to obtain bis-sulfoxide **8(*rac+meso*)** (181.0 mg, 0.62 mmol, 26% yield) as a yellow solid of a mixture of diastereomers in a 1:1 ratio; mp 106-108 °C; ^{1}H NMR (500 MHz, $CDCl_3$): δ 7.59-7.50 (m, 10H *rac*, 10H *meso*), 3.04-2.93 (m, 2H *rac*, 2H *meso*), 2.90-2.82 (m, 2H *rac*, 2H *meso*), 2.35-2.26 (m, 1H *meso*), 2.11 (quint, *J* = 7.3 Hz, 2H *rac*), 2.07-2.01 (m, 1H *meso*) ppm; ^{13}C{^{1}H} NMR (125 MHz, $CDCl_3$): δ 143.5 (*meso*), 143.3 (*rac*), 131.4 (*meso*), 131.3 (*rac*), 129.5 (2) (*meso* + *rac*), 124.1 (2) (*meso* + *rac*), 55.6 (*meso*), 55.2 (*rac*), 16.1 (*meso*), 15.6 (*rac*) ppm; HRMS (ESI) m/z: $[M+H]^+$ Calcd for $C_{15}H_{17}O_2S_2$ 293.0664; found 293.0666; 25:50:25 dr [(*S*,*S*):(*R*,*S*):(*R*,*R*)]; HPLC: ADH Chiracel column, (n-hexane/*iso*propanol 60:40; 0.6 mL/min.; 23 ˚C) t_R = 12.35 min. [(*S*,*S*)-isomer], t_R = 13.18 min. [(*R*,*S*)-isomer], t_R = 14.00 min. [(*R*,*R*)-isomer].

(S,S)/(R,R)/(R,S)-1,3-Bis[(2,6-dimethylphenyl)sulfinyl]propane, **10(*rac+meso*)**
It was prepared following the general procedure and was purified by flash chromatography (CH_2Cl_2/MeOH, 80:1) to obtain bis-sulfoxide **10(*rac+meso*)** (408.0 mg, 1.17 mmol, 49% yield) as a yellow solid of a mixture of diastereomers in a 1:1 ratio; mp 150-153 °C; ^{1}H NMR (500 MHz, $CDCl_3$): δ 7.24 (t, *J* = 7.6 Hz, 2H *rac*, 2H *meso*), 7.05 (d, *J* = 7.6 Hz, 4H *rac*, 4H *meso*), 3.50-3.45 (m, 2H *meso*), 3.41 (dt, *J* = 6.8 Hz, *J* = 13.3 Hz, 2H *rac*), 3.06-2.99 (m, 2H *rac*, 2H *meso*), 2.58 (s, 12H *meso*), 2.57 (s, 12H *rac*), 2.45-2.33 (m, 2H *rac*, 2H *meso*) ppm; ^{13}C{^{1}H} NMR (125 MHz, $CDCl_3$): δ 138.5 (*meso*), 138.4 (*rac*), 137.9 (*meso*), 137.8 (*rac*), 131.2 (2) (*meso* + *rac*), 130.5 (*meso* + *rac*), 50.6 (*meso*), 50.5 (*rac*), 19.4 (3) (*meso* + *rac*) ppm; HRMS (ESI) m/z: $[M+H]^+$ Calcd for $C_{19}H_{25}O_2S_2$ 349.1290; found 349.1285; 25:50:25 dr [(*S*,*S*):(*R*,*S*):(*R*,*R*)]; HPLC: ADH Chiracel column, (n-hexane/*iso*propanol 60:40; 0.6 mL/min.; 23 ˚C) t_R = 10.08 min. [(*S*,*S*)-isomer], t_R = 11.12 min. [(*R*,*R*)-isomer], t_R = 12.15 min. [(*R*,*S*)-isomer].

(S,S)/(R,R)/(R,S)-1,3-Bis(p-tolylsulfinyl)propane, **11(*rac+meso*)**
It was prepared following the general procedure and was purified by flash chromatography (CH_2Cl_2/MeOH, 40:1) to obtain bis-sulfoxide **11(*rac+meso*)** (328.0 mg, 1.02 mmol, 43% yield) as a yellow solid of a mixture of diastereomers in a 1:1 ratio; mp 110-113 °C; ^{1}H NMR (500 MHz, MeOD): δ 7.50 (d, *J* = 8.2 Hz, 4H *meso*), 7.50 (d, *J* = 8.2 Hz, 4H *rac*), 7.39 (d, *J* = 8.0 Hz, 4H *rac*, 4H *meso*), 3.09-3.01 (m, 2H *rac*, 2H *meso*), 2.98-

2.89 (m, 2H *rac*, 2H *meso*), 2.42 (s, 6H *rac*, 6H *meso*), 2.11-2.02 (m, 1H *meso*), 1.91 (quint, *J* = 7.6 Hz, 2H *rac*), 1.86-1.78 (m, 1H *meso*) ppm; $^{13}C\{^1H\}$ NMR (125 MHz, MeOD): δ 143.6 (2) (*meso* + *rac*), 140.1 (2) (*meso* + *rac*), 131.2 (*meso* + *rac*), 125.5 (*meso* + *rac*), 55.3 (*meso*), 55.2 (*rac*), 21.4, 16.4 (*meso*), 16.2 (*rac*) ppm; HRMS (ESI) m/z: $[M+H]^+$ Calcd for $C_{17}H_{21}O_2S_2$ 321.0977; found 321.0974; 25:50:25 dr [(*S*,*S*):(*R*,*S*):(*R*,*R*)]; HPLC: ADH Chiracel column, (n-hexane/*iso*propanol 60:40; 0.4 mL/min.; 23 ˚C) t_R = 24.18 min. [(*R*,*S*)-isomer], t_R = 25.31 min. [(*S*,*S*)-isomer], t_R = 26.17 min. [(*R*,*R*)-isomer].

(S,S)/(R,R)/(R,S)-1,3-Bis(methylsulfinyl)propane, **14(*rac*+*meso*)**

It was prepared following the general procedure and was purified by flash chromatography (CH_2Cl_2/MeOH, 10:1) to obtain bis-sulfoxide **14(*rac*+*meso*)** (185.0 mg, 1.10 mmol, 46% yield) as a yellow oil of a mixture of diastereomers in a 1:1 ratio; ^1H NMR (500 MHz, MeOD): δ 3.06-2.99 (m, 2H *rac*, 2H *meso*), 2.95-2.89 (m, 2H *rac*, 2H *meso*), 2.67 (s, 6H *rac*, 6H *meso*), 2.31-2.15 (m, 2H *rac*, 2H *meso*) ppm; $^{13}C\{^1H\}$ NMR (125 MHz, MeOD): δ 53.2 (*meso*), 53.1 (*rac*), 38.3 (*meso*), 38.2 (*rac*), 17.7 (*meso*), 17.4 (*rac*) ppm; HRMS (ESI) m/z: $[M+Na]^+$ Calcd for $C_5H_{12}O_2NaS_2$ 191.0171; found 191.0171; 25:50:25 dr [(*S*,*S*):(*R*,*S*):(*R*,*R*)]; HPLC: IH Chiracel column, (acetonitrile/water 95:5; 0.8 mL/min.; 25 ˚C) t_R =7.74 min. [(*S*,*S*)-isomer], t_R = 9.61 min. [(*R*,*S*)-isomer], t_R = 12.17 min. [(*R*,*R*)-isomer].

(S,S)/(R,R)/(R,S)-1,3-Bis(ethylsulfinyl)propane, **15(*rac*+*meso*)**

It was prepared following the general procedure and was purified by flash chromatography (CH_2Cl_2/MeOH, 20:1) to obtain bis-sulfoxide **15(*rac*+*meso*)** (167.0 mg, 0.85 mmol, 36% yield) as a yellow solid of a mixture of diastereomers in a 1:1 ratio; mp 125-128 °C; ^1H NMR (500 MHz, MeOD): δ 3.02-2.96 (m, 2H *rac*, 2H *meso*), 2.93-2.86 (m, 4H *rac*, 4H *meso*), 2.83-2.75 (m, 2H *rac*, 2H *meso*), 2.32-2.17 (m, 2H *rac*, 2H *meso*), 1.34 (t, *J* = 7.5 Hz, 6H *rac*, 6H *meso*) ppm; $^{13}C\{^1H\}$ NMR (125 MHz, MeOD): δ 50.6 (2) (*meso* + *rac*), 46.4 (*meso*), 46.3 (*rac*), 18.1 (*meso*), 17.8 (*rac*), 7.0 (2) (*meso* + *rac*) ppm; HRMS (ESI) m/z: $[M+H]^+$ Calcd for $C_7H_{17}O_2S_2$ 197.0664; found 197.0663; 25:50:25 dr [(*S*,*S*):(*R*,*S*):(*R*,*R*)]; HPLC: ADH Chiracel column, (n-hexane/*iso*propanol 60:40; 0.6 mL/min.; 23 ˚C) t_R = 8.08 min. [(*R*,*R*)-isomer], t_R = 8.85 min. [(*R*,*S*)-isomer], t_R = 12.01 min. [(*S*,*S*)-isomer].

(S,S)/(R,R)/(R,S)-1,3-Bis(propylsulfinyl)propane, **16(*rac*+*meso*)**

It was prepared following the general procedure and was purified by flash chromatography (CH_2Cl_2/MeOH, 30:1) to obtain bis-sulfoxide **16(*rac*+*meso*)** (71.0 mg, 0.32 mmol, 13% yield) as a yellow solid of a mixture of diastereomers in a 1:1 ratio; mp 120-123 °C; ^1H NMR (500 MHz, MeOD): δ 3.04-2.97 (m, 2H *rac*, 2H *meso*), 2.94-2.88 (m, 2H *rac*, 2H *meso*), 2.83-2.79 (m, 4H *rac*, 4H *meso*), 2.30-2.21 (m, 2H *rac*, 2H *meso*), 1.87-1.76 (m, 4H *rac*, 4H *meso*), 1.11 (t, *J* = 7.5 Hz, 6H *rac*, 6H *meso*) ppm; $^{13}C\{^1H\}$ NMR (125 MHz, MeOD): δ 54.8 (*meso*), 54.7 (*rac*), 51.2 (2) (*meso* + *rac*), 18.1 (*meso*), 17.8 (*rac*), 17.4 (*meso*), 17.3 (*rac*), 13.5 (*meso* + *rac*) ppm; HRMS (ESI) m/z: $[M+H]^+$ Calcd for

$C_9H_{21}O_2S_2$ 225.0977; found 225.0976; 25:50:25 dr [(*S*,*S*):(*R*,*S*):(*R*,*R*)]; HPLC: ADH Chiracel column, (n-hexane/*iso*propanol 60:40; 0.6 mL/min.; 23 ˚C) t_R = 7.78 min. [(*R*,*R*)-isomer], t_R = 8.55 min. [(*R*,*S*)-isomer], t_R = 12.03 min. [(*S*,*S*)-isomer].

(S,S)/(R,R)/(R,S)-1,3-Bis(isopropylsulfinyl)propane, **17(*rac+meso*)**

It was prepared following the general procedure and was purified by flash chromatography (CH_2Cl_2/MeOH, 30:1) to obtain bis-sulfoxide **17(*rac+meso*)** (239.0 mg, 1.07 mmol, 45% yield) as a yellow solid of a mixture of diastereomers in a 1:1 ratio; mp 67-69 °C; ^{1}H NMR (500 MHz, MeOD): δ 3.00-2.84 (m, 6H *rac*, 6H *meso*), 2.35-2.19 (m, 2H *rac*, 2H *meso*), 1.32-1.29 (m, 12H *rac*, 12H *meso*) ppm; ^{13}C{^{1}H} NMR (125 MHz, MeOD): δ 51.5 (2) (*meso* + *rac*), 47.9 (2) (*meso* + *rac*), 18.7 (*meso*), 18.5 (*rac*), 16.4 (2) (*meso* + *rac*), 14.5 (2) (*meso* + *rac*) ppm; HRMS (ESI) m/z: $[M+Na]^+$ Calcd for $C_9H_{20}O_2NaS_2$ 247.0797; found 247.0796; 25:50:25 dr [(*S*,*S*):(*R*,*S*):(*R*,*R*)]; HPLC: ADH Chiracel column, (n-hexane/*iso*propanol 70:30; 0.7 mL/min.; 23 ˚C) t_R = 7.36 min. [(*S*,*S*)-isomer], t_R = 7.88 min. [(*R*,*S*)-isomer], t_R = 8.30 min. [(*R*,*R*)-isomer].

(S,S)/(R,R)/(R,S)-1,3-Bis(tert-butylsulfinyl)propane, **18(*rac+meso*)**

It was prepared following the general procedure and was purified by flash chromatography (TBME/MeOH, 20:1) to obtain bis-sulfoxide **18(*rac+meso*)** (108.0 mg, 0.43 mmol, 18% yield) as a yellow solid of a mixture of diastereomers in a 1:1 ratio; mp 104-107 °C; ^{1}H NMR (500 MHz, MeOD): δ 2.94-2.88 (m, 2H *rac*, 2H *meso*), 2.78-2.69 (m, 2H *rac*, 2H *meso*), 2.42-2.34 (m, 1H *meso*), 2.30 (quint, *J* = 7.6 Hz, 2H *rac*), 2.28-2.15 (m, 1H *meso*), 1.28 (s, 18H *rac*, 18H *meso*) ppm; ^{13}C{^{1}H} NMR (125 MHz, MeOD): δ 54.6 (2) (*meso* + *rac*), 45.0 (*rac*), 44.9 (*meso*), 22.9 (*meso* + *rac*), 20.2 (*meso* + *rac*) ppm; HRMS (ESI) m/z: $[M+H]^+$ Calcd for $C_{11}H_{25}O_2S_2$ 253.1290; found 253.1291; 25:50:25 dr [(*S*,*S*):(*R*,*S*):(*R*,*R*)]; HPLC: ADH Chiracel column, (n-hexane/*iso*propanol 80:20; 0.3 mL/min.; 23 ˚C) t_R =18.53 min. [(*S*,*S*)-isomer], t_R = 20.02 min. [(*R*,*R*)-isomer], t_R = 20.77 min. [(*R*,*S*)-isomer].

(S,S)/(R,R)/(R,S)-Bis(1-naphthylsulfinyl)propane, **9(*rac+meso*)**

To a solution of dibenzyl propane-1,3-bis(sulfinate) (530.0 mg, 1.50 mmol, 100 mol%) in dry toluene (30 mL) under argon atmosphere at 0 °C, 0.25M 1-naphthylmagnesium bromide solution (18.0 mL, 4.51 mmol, 300 mol%) was added dropwise. The mixture was stirred for 1 h, quenched with saturated aqueous NH_4Cl solution and the aqueous phase was extracted with CH_2Cl_2. The combined organic phases were washed with saturated aqueous NaCl solution and dried with anhydrous Na_2SO_4. The residue was purified by flash chromatography (CH_2Cl_2/MeOH, 80:1) to obtain bis-sulfoxide **9(*rac+meso*)** (327.0 mg, 0.83 mmol, 55% yield) as a brown solid of a mixture of diastereomers in a 1:1 ratio; mp 145-148 °C; ^{1}H NMR (500 MHz, $CDCl_3$): δ 8.05 (dd, *J* = 1 Hz, *J* = 7.3 Hz, 2H *meso*), 7.97-7.93 (m, 6H *rac*, 4H *meso*), 7.90-7.85 (m, 2H *rac*, 2H *meso*), 7.64-7.53 (m, 6H *rac*, 6H *meso*), 3.23-3.14 (m, 2H *rac*, 2H *meso*), 2.95-2.85 (m, 2H *rac*, 2H *meso*), 2.50-2.42 (m, 1H *meso*), 2.13 (quint, *J* = 7.4 Hz, 2H *rac*), 2.05-1.96 (m, 1H *meso*)

ppm; $^{13}C\{^1H\}$ NMR (125 MHz, $CDCl_3$): δ 138.8 (*meso*), 138.5 (*rac*), 133.6 (2) (*rac* + *meso*), 131.5 (2) (*meso* + *rac*), 129.3 (2) (*rac* + *meso*), 128.8 (*rac* + *meso*), 127.6 (*rac*), 127.5 (*meso*), 126.9 (*rac* + *meso*), 125.7 (*meso*), 125.6 (*rac*), 123.4 (*rac*), 123.3 (*meso*), 121.5 (*rac* + *meso*), 53.7 (*meso*), 53.3 (*rac*), 16.0 (*meso*), 15.8 (*rac*) ppm; HRMS (ESI) m/z: $[M+Na]^+$ Calcd for $C_{23}H_{20}O_2NaS_2$ 415.0797; found 415.0794; 25:50:25 dr [(*S*,*S*):(*R*,*S*):(*R*,*R*)]; HPLC: AD Chiracel column, (n-hexane/*iso*propanol 25:75; 0.2 mL/min.; 23 ˚C) t_R =46.42 min. [(*R*,*S*)-isomer], t_R = 50.01 min. [(*R*,*R*)-isomer], t_R = 53.73 min. [(*S*,*S*)-isomer].

(S,S)/(R,R)/(R,S)-1,3-Bis(benzylsulfinyl)propane, **12(*rac+meso*)**

To a solution of 1,3-bis(benzylthio)propane (869.0 mg, 3.01 mmol, 100 mol%) in CH_2Cl_2 (20 mL) at -78 °C, a solution of *m*-CPBA (1.4 g, 6.03 mmol, 200 mol%) in CH_2Cl_2 (20 mL) was added dropwise. The mixture was stirred for 2 h at -78 °C, quenched with saturated aqueous $NaHCO_3$ solution and the aqueous phase was extracted with CH_2Cl_2. The combined organic phases were washed with saturated aqueous NaCl solution and dried with anhydrous Na_2SO_4. The residue was purified by flash chromatography (CH_2Cl_2/*iso*propanol, 40:1) to obtain bis-sulfoxide **12(*rac+meso*)** (789.0 mg, 2.46 mmol, 82% yield) as a yellow solid of a mixture of diastereomers in a 1:1 ratio; mp 165-168 °C; ^1H NMR (500 MHz, $CDCl_3$): δ 7.39-7.34 (m, 6H *rac*, 6H *meso*), 7.28-7.26 (m, 4H *rac*, 4H *meso*), 4.03-3.94 (m, 4H *rac*, 4H *meso*), 2.77-2.70 (m, 2H *rac*, 2H *meso*), 2.68-2.62 (m, 2H *rac*, 2H *meso*), 2.31-2.23 (m, 2H *rac*, 2H *meso*) ppm; $^{13}C\{^1H\}$ NMR (125 MHz, $CDCl_3$): δ 130.1 (2) (*meso* + *rac*), 129.6 (*meso* + *rac*), 129.2 (2) (*meso* + *rac*), 128.7 (*meso* + *rac*), 58.6 (*meso*), 58.5 (*rac*), 49.4 (*meso*), 49.1 (*rac*), 16.8 (*meso*), 16.3 (*rac*) ppm; HRMS (ESI) m/z: $[M+Na]^+$ Calcd for $C_{17}H_{20}O_2NaS_2$ 343.0797; found 343.0793; 25:50:25 dr [(*S*,*S*):(*R*,*S*):(*R*,*R*)]; HPLC: AD Chiracel column, (n-hexane/*iso*propanol 50:50; 0.2 mL/min.; 23 ˚C) t_R =33.35 min. [(*S*,*S*)-isomer], t_R = 35.69 min. [(*R*,*S*)-isomer], t_R = 45.08 min. [(*R*,*R*)-isomer].

(S,S)/(R,R)/(R,S)-Bis[(pyridin-2-ylmethyl)sulfinyl]propane, **13(*rac+meso*)**

To a solution of 2-methylpyridine (0.7 mL, 7.10 mmol, 500 mol%) in dry THF (30 mL) under argon atmosphere at -78 °C, 2.5M *t*-BuLi solution (2.6 mL, 6.53 mmol, 460 mol%) was added dropwise. After 30 min at -50 °C, the reaction mixture was cannulated into a second flask under argon atmosphere and at 0 °C containing a solution of dibenzyl propane-1,3-disulfinate (500.0 mg, 1.42 mmol, 100 mol%) in dry THF (11 mL). The mixture was stirred for 1 h, quenched with saturated aqueous NaCl solution and the aqueous phase was extracted with CH_2Cl_2. The combined organic phases were dried with anhydrous Na_2SO_4 and the residue was purified by flash chromatography (CH_2Cl_2/MeOH, 15:1) to obtain bis-sulfoxide **13(*rac+meso*)** (316.0 mg, 0.98 mmol, 69% yield) as a brown solid of a mixture of diastereomers in a 1:1 ratio; mp 93-96 °C; ^1H NMR (500 MHz, $CDCl_3$): δ 8.60 (d, *J* = 4.45 Hz, 2H *rac*, 2H *meso*),), 7.71 (dt, *J* = 7.7 Hz, *J* = 1.8 Hz, 2H *rac*, 2H *meso*), 7.35-7.34 (m, 2H *rac*, 2H *meso*), 7.28-7.26 (m, 2H *rac*, 2H *meso*), 4.22-4.10 (m, 2H *rac*, 2H *meso*), 2.96-2.90 (m, 2H *rac*, 2H *meso*), 2.83-2.78 (m, 2H *rac*,

2H *meso*), 2.32 (quint, *J* = 7.5 Hz, 2H *rac*, 2H *meso*) ppm; $^{13}C\{^1H\}$ NMR (125 MHz, $CDCl_3$): δ 150.6 (2) (*meso* + *rac*), 150.2 (*meso* + *rac*), 137.1 (*meso* + *rac*), 125.5 (*meso* + *rac*), 123.3 (*meso* + *rac*), 59.8 (*meso*), 59.7 (*rac*), 50.1 (*meso*), 50.0 (*rac*), 16.8 (*meso*), 16.6 (*rac*) ppm; HRMS (ESI) m/z: $[M+Na]^+$ Calcd for $C_{15}H_{18}O_2N_2NaS_2$ 345.0702; found 345.0700; 25:50:25 dr [(*S*,*S*):(*R*,*S*):(*R*,*R*)]; HPLC: AD Chiracel column, (n-hexane/*iso*propanol 60:40; 0.6 mL/min.; 23 ˚C) t_R =30.79 min. [(*R*,*R*)-isomer], t_R = 44.56 min. [(*S*,*S*)-isomer], t_R = 48.05 min. [(*R*,*S*)-isomer].

General Procedure for enantioselective allylation of *N*-(benzoyl)*iso*butylhydrazone. [1]

To a solution of *N*-(benzoyl)*iso*butylhydrazone **19** (20.5 mg, 0.12 mmol, 100 mol%), the chiral ligand (150 mol%) and 2-methyl-2-butene (27.0 µL, 0.05 mmol, 50 mol%) in the corresponding volume of dichloromethane for the desired molarity, allyltrichlorosilane (23.5 µL, 0.16 mmol, 150 mol%) was added dropwise at -78 ˚C. After stirred at -78 ˚C overnight, the reaction was quenched by adding saturated aqueous $NaHCO_3$ (1.1 mL). After warming to r.t., saturated NaCl aqueous solution was added, and the mixture was extracted with CH_2Cl_2 (3 x 10 mL). The combined organic layers were dried over Na_2SO_4 and concentrated in vacuo. The residue was purified by column chromatography (EtOAc/hexane, 1:6) to afford the corresponding *N*'-(1-*iso*propylbut-3-enyl)benzohydrazide **21** in high chemical yields as a white solid; mp 73-74 °C; ^1H NMR (500 MHz, $CDCl_3$): δ 7.75-7.78 (m, 2H), 7.58 (bs, 1H), 7.53-7.45 (m, 1H), 7.45-7.40 (m, 2H), 6.00-5.90 (m, 1H), 5.23-5.10 (m, 2H), 2.90 (quint, *J* = 4.4 Hz, 1H), 2.35-2.25 (m, 1H), 2.19-2.10 (m, 1H), 1.85-1.95 (m, 1H), 0.93 (d, *J* = 6.8Hz, 3H), 0.92 (d, *J* = 6.8 Hz, 3H) ppm; HPLC: ODH Chiracel column, (n-hexane/*iso*propanol 90:10; 0.6 mL/min.; 25 ˚C) t_R =12.91 min. [(*R*)-isomer], t_R = 14.32 min. [(*S*)-isomer].

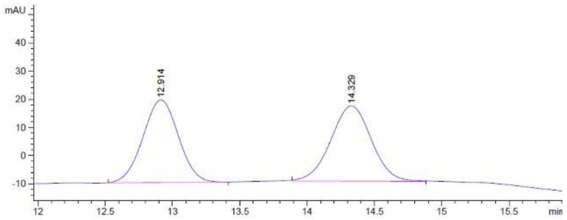

Peak #	RetTime [min]	Area %
1	12.914	48.9256
2	14.329	51.0744

21(*rac*)

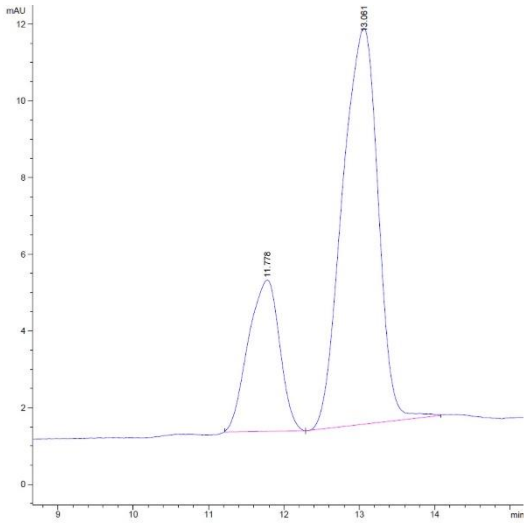

Peak #	Time [min]	Area [mAU*s]	Area %
1	11.778	113.99583	25.1707
2	13.061	338.89520	74.8293

21(*S*) obtained with **8(*S*,*S*)**

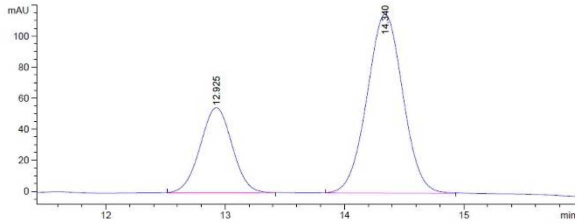

Peak #	RetTime [min]	Area %
1	12.925	29.8710
2	14.340	70.1290

21(*S*) obtained with **10(*S*,*S*)**

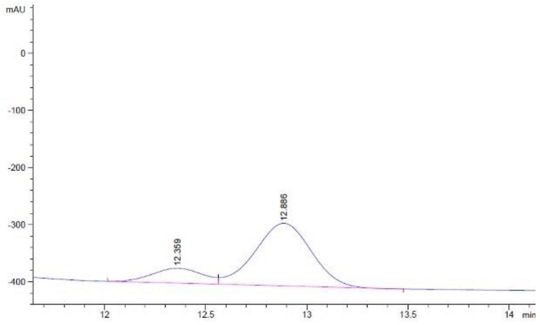

Peak #	RetTime [min]	Area %
1	12.364	18.2778
2	12.886	81.7222

21(*S*) obtained with **11(*S*,*S*)**

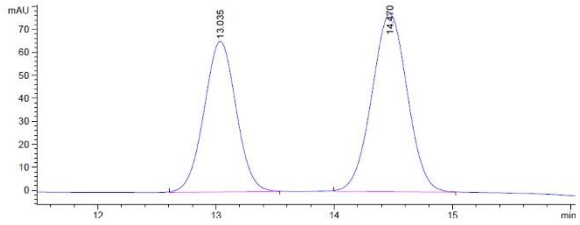

Peak #	RetTime [min]	Area %
1	13.036	43.3315
2	14.470	56.6685

21(*S*) obtained with **9(*S*,*S*)**

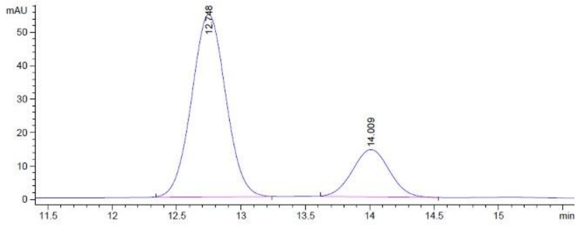

Peak #	RetTime [min]	Area %
1	12.748	78.0301
2	14.009	21.9699

21(*R*) obtained with **18(*S*,*S*)**

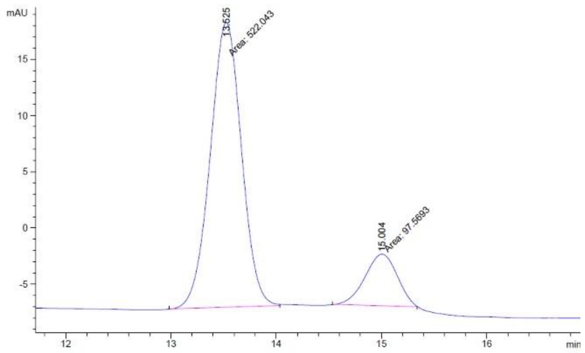

Peak #	RetTime [min]	Area %
1	13.525	84.2532
2	15.004	15.7468

21(*R*) obtained with **17(*S,S*)**

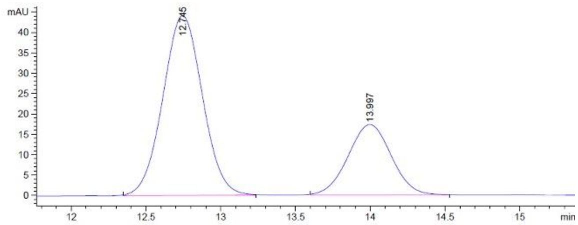

Peak #	RetTime [min]	Area %
1	12.745	70.0830
2	13.997	29.9170

21(*R*) obtained with **16(*S,S*)**

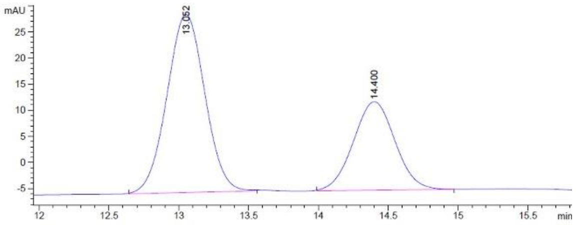

Peak #	RetTime [min]	Area %
1	13.052	64.6451
2	14.400	35.3549

21(*R*) obtained with **15(*R,R*)**

General Procedure for preparation of racemic vinyl sulfoxides

To a solution of the corresponding sulfinyl chloride (100 mol%) in dry Et_2O under argon atmosphere at -78 °C, 1M vinylmagnesium bromide solution (120 mol%) was added dropwise. The mixture was stirred for 20 min, quenched with distillated H_2O and the aqueous phase was extracted with CH_2Cl_2. The combined organic phases were dried with anhydrous Na_2SO_4 to obtain the desired compound.

(rac)-p-Tolyl vinyl sulfoxide, **25(*rac*)**

It was prepared following the general procedure from *p*-tolylsulfinyl chloride (735.0 g, 4.21 mmol), 1M vinylmagnesium bromide solution (5.1 mL, 6.05 mmol, 120 mol%) and Et_2O (25 mL). It was obtained vinyl sulfoxide **25(*rac*)** (700.0 mg, 4.21 mmol, quant. yield) as a volatile yellow liquid with similar physicochemical and spectroscopic characteristics than **25(*R*)**; HRMS (ESI) m/z: $[M+H]^+$ Calcd for $C_9H_{11}OS$ 167.0525; found 167.0524; 50:50 er [(*S*):(*R*)]; HPLC: OB Chiracel column, (n-hexane/*iso*propanol 85:15; 1 mL/min.; 25 ˚C) t_R = 9.15 min. [(*S*)-isomer], t_R = 14.24 min. [(*R*)-isomer].

(rac)-tert-Butyl vinyl sulfoxide, **26(*rac*)**

It was prepared following the general procedure from *tert*-butylsulfinyl chloride (1.0 g, 7.14 mmol), 1M vinylmagnesium bromide solution (8.6 mL, 8.57 mmol, 120 mol%) and Et_2O (24 mL). The residue obtained was purified by flash chromatography (CH_2Cl_2/acetone, 30:1) to obtain vinyl sulfoxide **26(*rac*)** (332.8 mg, 2.52 mmol, 35%) as a volatile yellow liquid with similar physicochemical and spectroscopic characteristics than **26(*R*)**; HRMS (ESI) m/z: $[M+Na]^+$ Calcd for $C_6H_{12}ONaS$ 155.0501; found 155.0495; 50:50 er [(*S*):(*R*)]; HPLC: AD Chiracel column, (n-hexane/*iso*propanol 98:2; 0.5 mL/min.; 25 ˚C) t_R = 31.84 min. [(*R*)-isomer], t_R = 34.03 min. [(*S*)-isomer].

(rac)-Methyl vinyl sulfoxide, **27(*rac*)**

It was prepared following the general procedure from methylsulfinyl chloride (1.0 g, 10.15 mmol), 1M vinylmagnesium bromide solution (12.2 mL, 12.17 mmol, 120 mol%) and Et_2O (34 mL). It was obtained vinyl sulfoxide **27(*rac*)** (911.0 mg, 10.10 mmol, quant. yield) as a volatile yellow liquid with similar physicochemical and spectroscopic characteristics than **27(*S*)**; HRMS (ESI) m/z: $[M+H]^+$ Calcd for C_3H_7OS 91.0212; found 91.0214.

General Procedure for non-stereoselective synthesis of 1,5-bis(sulfinyl)-3-thioderivatives

A solution of the corresponding vinyl sulfoxide (200 mol%) and Na_2S (100 mol%) in H_2O was stirring at 55 ˚C overnight. Then, the reaction mixture was extracted with CH_2Cl_2 (3 x 20 mL). The combined organic phases were dried with anhydrous Na_2SO_4, the residue was evaporated to vacuum and purified by flash chromatography.

(S,S)/(R,R)/(R,S)-Bis[2-(p-tolylsulfinyl)ethyl] sulfide, **28(*rac* + *meso*)**

It was prepared following the general procedure from (*rac*)-*p*-tolyl vinyl sulfoxide, **25(*rac*)** (200.0 mg, 1.20 mmol) and Na_2S (144.1 mg, 0.60 mmol) in H_2O (0.6 mL). The residue obtained was purified by flash chromatography (CH_2Cl_2/MeOH, 40:1) to obtain bis-sulfoxide **28(*rac* + *meso*)** (139.0 mg, 0.38 mmol, 63%) as a colorless oil; ^1H NMR (500 MHz, $CDCl_3$): δ 7.50-7.48 (m, 4H), 7.33 (d, *J* = 7.2 Hz, 4H), 3.04-2.87 (m, 6H), 2.71-2.63 (m, 2H), 2.42 (s, 6H) ppm; $^{13}C\{^1H\}$ NMR (125 MHz, $CDCl_3$): δ 141.9 (2) (*meso* + (*S,S*)), 139.9 (*S,S*), 139.8 (*meso*), 130.2 ((*S,S*) + *meso*), 124.1 ((*S,S*) + *meso*), 56.4 (*S,S*), 56.3 (*meso*),

24.4 (*S,S*), 24.3 (*meso*), 21.6 ((*S,S*) + *meso*) ppm; HRMS (ESI) m/z: $[M+Na]^+$ Calcd for $C_{18}H_{22}O_2NaS_3$ 389.0674; found 389.0670; 25:50:25 dr [(*S*,*S*):(*R*,*S*):(*R*,*R*)]; HPLC: ADH Chiracel column, (n-hexane/*iso*propanol 60:40; 0.4 mL/min.; 25 ˚C) t_R = 33.48 min. [(*R*,*R*)-isomer], t_R = 36.70 min. [(*R*,*S*)-isomer], t_R = 41.77 min. [(*S*,*S*)-isomer].

(S,S)/(R,R)/(R,S)-Bis[2-(tert-butylsulfinyl)ethyl] sulfide, **29(*rac* + *meso*)**
It was prepared following the general procedure from (*rac*)-*tert*-butyl vinyl sulfoxide, **26(*rac*)** (200.0 mg, 1.51 mmol) and Na_2S (182.5 mg, 0.76 mmol) in H_2O (0.5 mL). The residue obtained was purified by flash chromatography (EtOAc/MeOH, 30:1) to obtain bis-sulfoxide **29(*rac* + *meso*)** (64.8 mg, 0.22 mmol, 29%) as a yellow oil; ^1H NMR (500 MHz, MeOD): δ 3.23-2.90 (m, 7H), 2.86-2.79 (m, 1H), 1.29-1.28 (m, 18H) ppm; $^{13}C\{^1H\}$ NMR (125 MHz, MeOD): δ 53.6 (*meso*), 54.5 (*rac)*, 46.7 (*meso*), 46.6 (*rac*), 26.8 (*meso*), 26.7 (*rac*), 23.0 (*meso* + *rac*) ppm; HRMS (ESI) m/z: $[M+Na]^+$ Calcd for $C_{12}H_{26}O_2NaS_3$ 321.0987; found 321.0986; 25:50:25 dr [(*S*,*S*):(*R*,*S*):(*R*,*R*)]; HPLC: IF Chiracel column, (EtOH 100 %; 1 mL/min.; 23 ˚C) t_R = 5.94 min. [(*S*,*S*)-isomer], t_R = 6.67 min. [(*R*,*S*)-isomer], t_R = 7.19 min. [(*R*,*R*)-isomer].

(S,S)/(R,R)/(R,S)-Bis[2-(methylsulfinyl)ethyl] sulfide, **30(*rac* + *meso*)**
It was prepared following the general procedure from (*rac*)-methyl vinyl sulfoxide, **27(*rac*)** (912.8 mg, 10.14 mmol) and Na_2S (1.2 g, 5.07 mmol) in H_2O (5.1 mL). The residue obtained was purified by flash chromatography (CH_2Cl_2/MeOH, 20:1) to obtain bis-sulfoxide **30(*rac* + *meso*)** (131.8 mg, 0.62 mmol, 12%) as a colorless oil; ^1H NMR (500 MHz, DMSO-d_6): δ 3.08-3.02 (m, 2H),2.95-2.82 (m, 6H), 2.57 (s, 6H) ppm; $^{13}C\{^1H\}$ NMR (125 MHz, DMSO-d_6): δ 52.8, 37.8 (2) (*rac* + *meso*), 23.8 ppm; HRMS (ESI) m/z: $[M+Na]^+$ Calcd for $C_6H_{14}O_2NaS_3$ 237.0048; found 237.0046; 25:50:25 dr [(*S*,*S*):(*R*,*S*):(*R*,*R*)]; HPLC: IF Chiracel column, (MeOH 100 %; 1 mL/min.; 25 ˚C) t_R = 7.94 min. [(*R*,*R*)-isomer], t_R = 8.36 min. [(*R*,*S*)-isomer], t_R = 9.53 min. [(*S*,*S*)-isomer].

Regioselective oxidation of 1,5-bis(sulfinyl)-3-thioderivatives.

As an example, Figure S5 shows how the most important effect in the case of the obtained tris-sulfoxides **33(*R,R*)**, is noticeable in the alkyl chain, where the diastereotopicity and non-equivalence of proton signals increased. In the thio derivative **28(*R,R*)**, the chain protons are equivalent in pairs, whereas in the tris(sulfinyl) derivative **33(*R,R*)**, all chain protons were found to be non-equivalent. Moreover, the aromatic protons also exhibited diastereotopy (Figure S5).

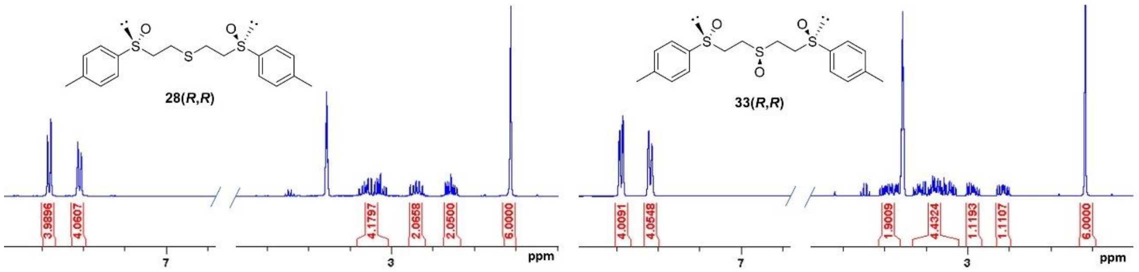

Figure S5. ${}^{1}H$ NMR spectrums (500 MHz, MeOD) of compounds **28(*R,R*)** and **33(*R,R*)**.

Chiral metal complexes containing C₂-symmetric bis-sulfoxides.

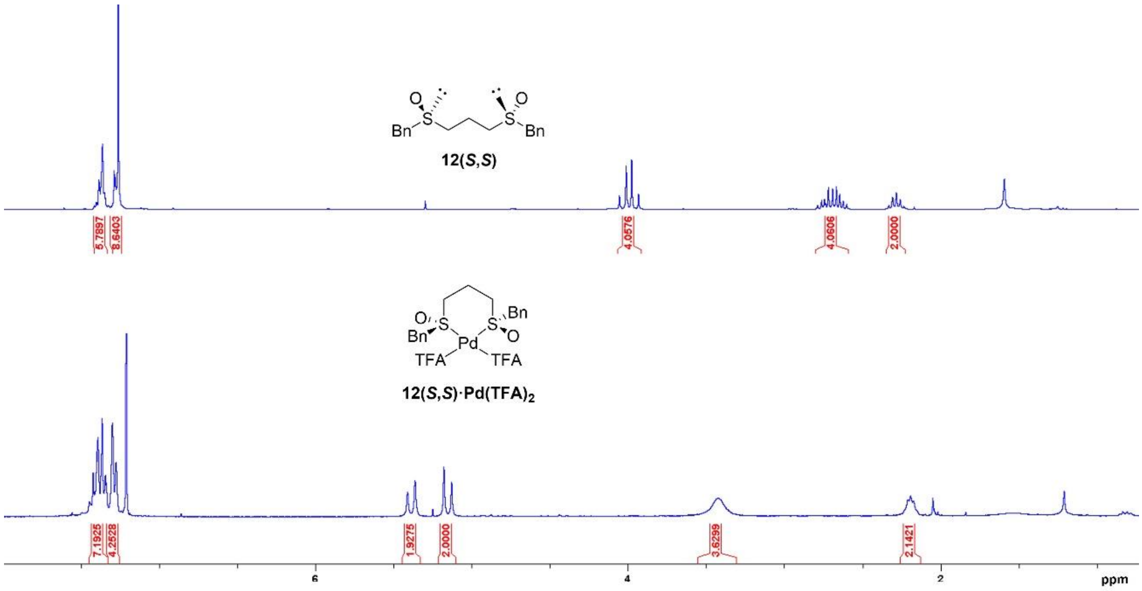

Figure S6. Comparison of ${}^{1}H$ NMR spectra (500 MHz, $CDCl_3$) of (*S*,*S*)-1,3-bis(benzylsulfinyl) propane, **12(*S,S*)** and its palladium (II) complex, **12(*S,S*)·Pd(TFA)₂**.

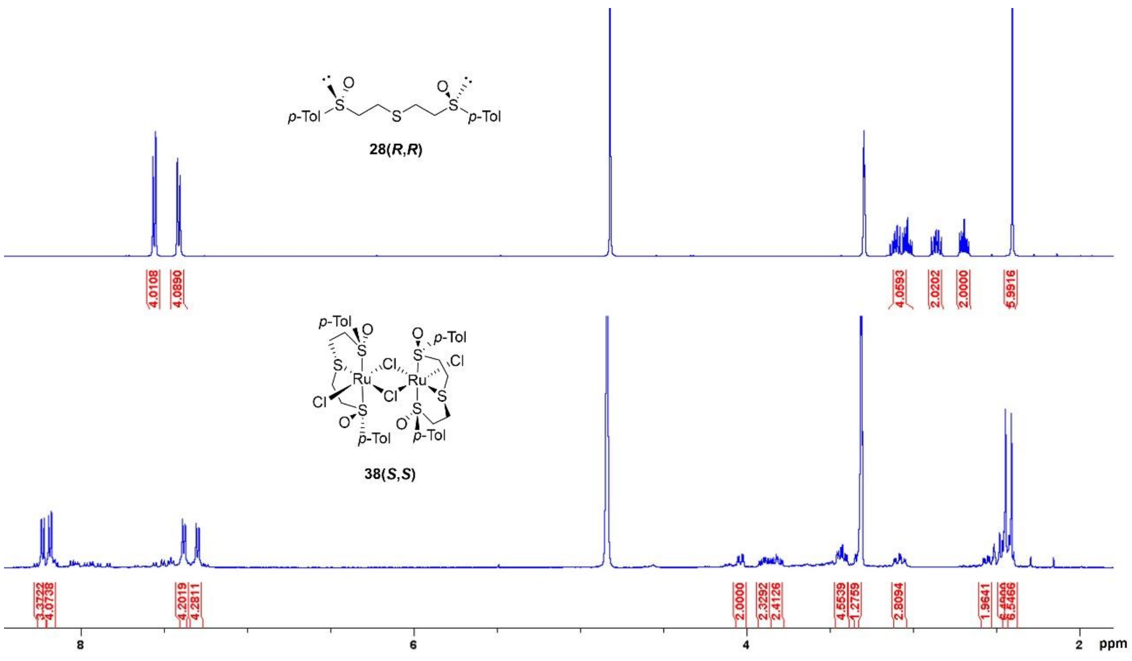

Figure S7. Comparison of ^{1}H NMR (500 MHz, MeOD) spectrums of (*R*,*R*)-Bis[2-(*p*-tolylsulfinyl)ethyl] sulfide, **28(*R*,*R*)** and the dinuclear Ru complex **38(*S,S*)**.

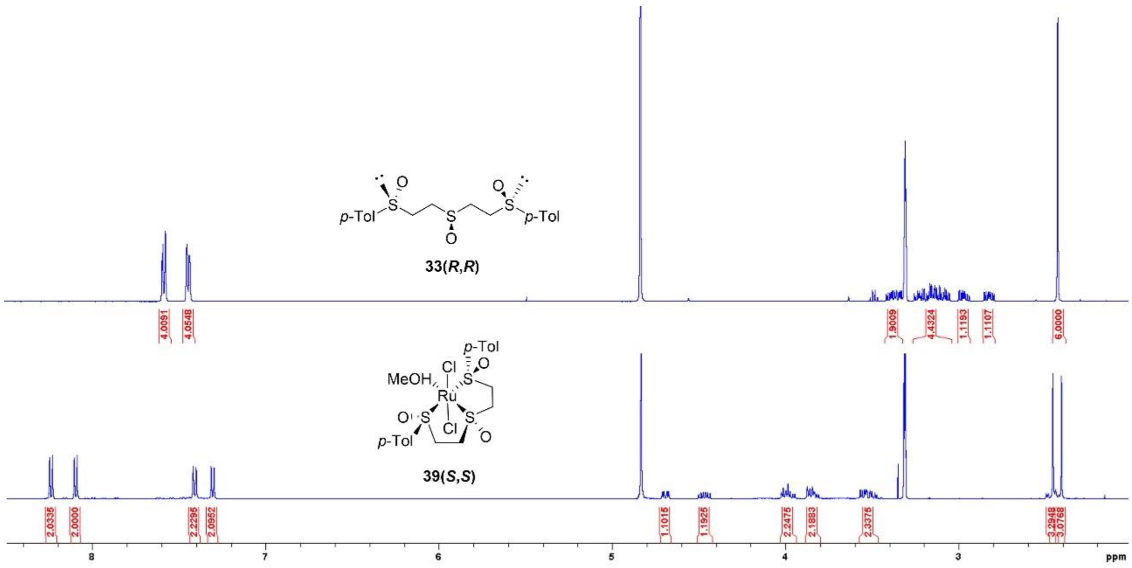

Figure S8. Comparison of ^{1}H NMR (500 MHz, MeOD) spectrums of (*R*,*R*)-Bis[2-(*p*-tolylsulfinyl)ethyl] sulfoxide, **33(*R*,*R*)** and the mononuclear Ru complex **39(*S,S*)**.

Table S1. Spectroscopic data (IR) of 1,3-bis(sulfinyl)propanes as a free ligand and in its Ru metal complexes.

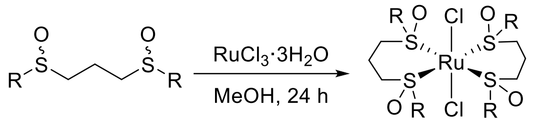

Entry	R	Ligand	Complex	IR, $\nu_{S\text{-}O}$ (cm^{-1})	
				Ligand	Complex
**1**	Me	**14(*R*,*R*)**	**36(*S*,*S*)**	995	1083
**2**	Me	**14(*S*,*S*)**	**36(*R*,*R*)**	995	1083
**5**	Et	**15(*R*,*R*)**	**37(*S*,*S*)**	1013	1080
**6**	Et	**15(*S*,*S*)**	**37(*R*,*R*)**	1013	1081

HPLC chromatograms of selected compounds.

1,3-Bis(alkyl- and arylsulfinyl)propanes

(S,S)-1,3-Bis(phenylsulfinyl)propane, **8(*S,S*)**

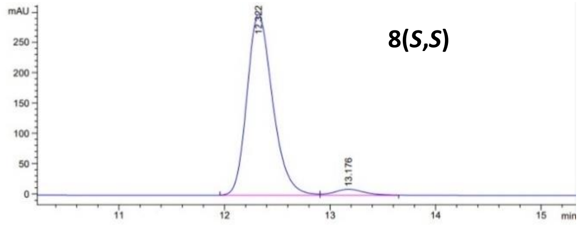

Peak #	RetTime [min]	Area %
1	12.322	96.5878
2	13.176	3.4122

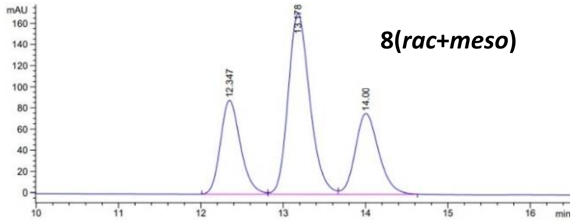

Peak #	RetTime [min]	Area %
1	12.347	24.4392
2	13.178	51.0715
3	14.004	24.4893

(S,S)-Bis(1-naphthylsulfinyl)propane, **9(*S,S*)**

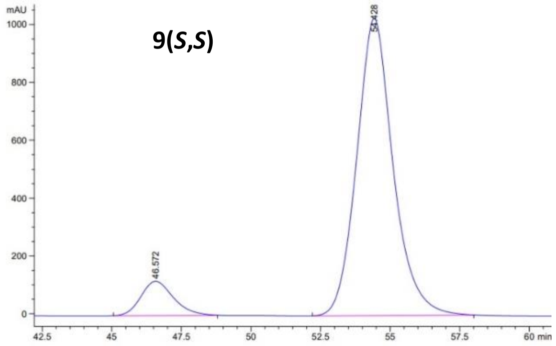

Peak #	RetTime [min]	Area %
1	46.572	9.3989
2	54.428	90.6011

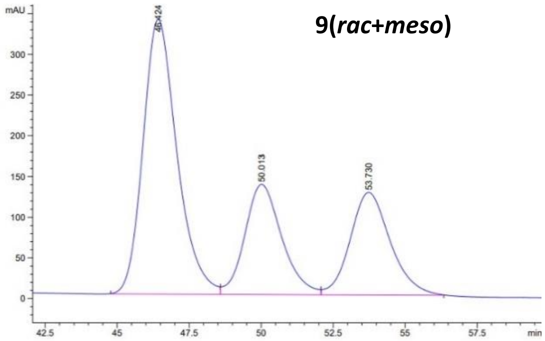

Peak #	RetTime [min]	Area %
1	46.424	53.1717
2	50.013	23.2825
3	53.730	23.5458

(S,S)-1,3-Bis[(2,6-dimethylphenyl)sulfinyl]propane, **10(*S,S*)**

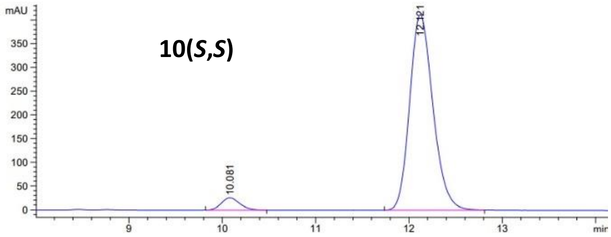

Peak #	RetTime [min]	Area %
1	10.081	4.6526
2	12.121	95.3474

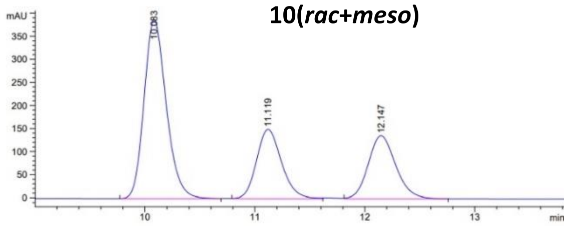

Peak #	RetTime [min]	Area %
1	10.083	53.9228
2	11.119	23.0455
3	12.147	23.0317

(R,R)-1,3-Bis[(2,6-dimethylphenyl)sulfinyl]propane, **10(*R,R*)**

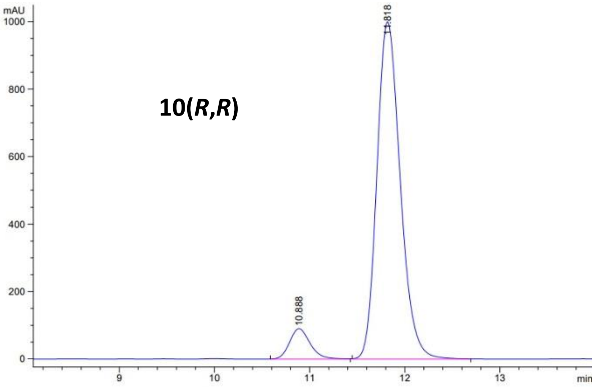

Peak #	RetTime [min]	Area %
1	10.888	7.3821
2	11.818	92.6179

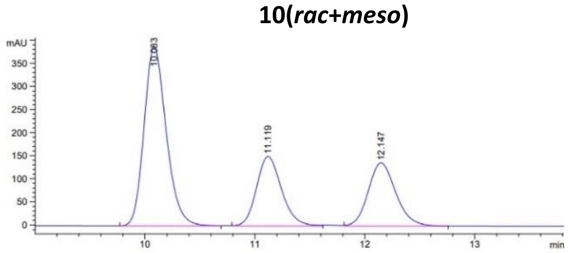

Peak #	RetTime [min]	Area %
1	10.083	53.9228
2	11.119	23.0455
3	12.147	23.0317

(S,S)-1,3-Bis(p-tolylsulfinyl)propane, **11(*S,S*)**

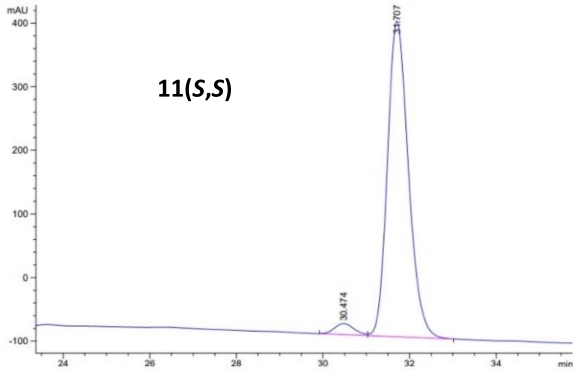

Peak #	RetTime [min]	Area %
1	30.474	3.1895
2	31.707	96.8105

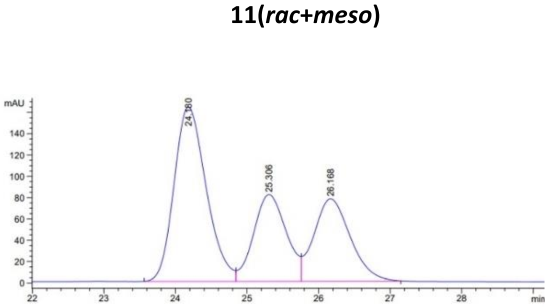

Peak #	RetTime [min]	Area %
1	24.180	49.0314
2	25.306	24.7485
3	26.168	26.2201

(S,S)-1,3-Bis(benzylsulfinyl)propane, **12(*S,S*)**

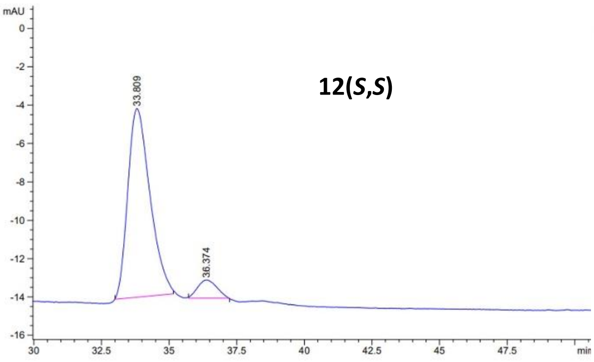

Peak #	RetTime [min]	Area %
1	33.809	92.2764
2	36.374	7.7236

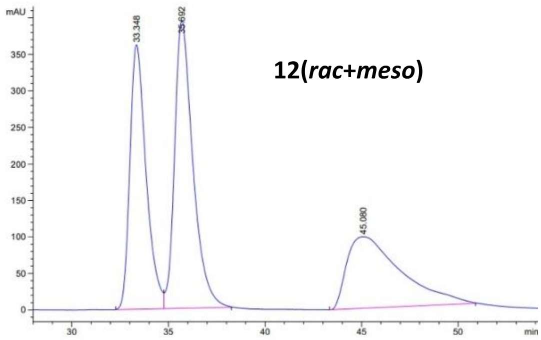

Peak #	RetTime [min]	Area %
1	33.348	31.9661
2	35.692	39.2167
3	45.080	28.8172

(S,S)-1,3-Bis[(pyridine-2-ylmethyl)sulfinyl]propane, **13(*S,S*)**

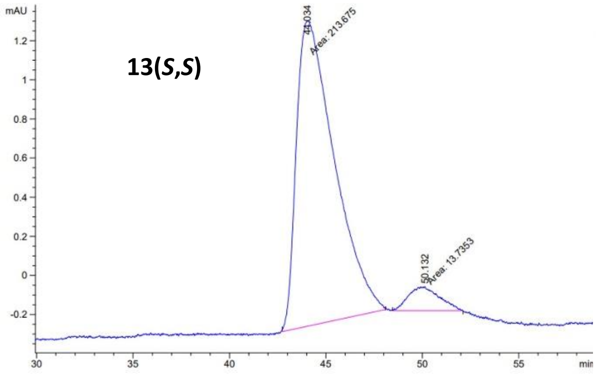

Peak #	RetTime [min]	Area %
1	44.034	93.9601
2	50.132	6.0399

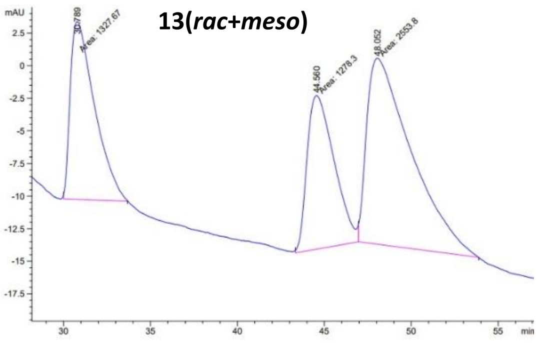

Peak #	RetTime [min]	Area %
1	30.789	25.7312
2	44.560	24.7744
3	48.052	49.4944

(R,R)-1,3-Bis(methylsulfinyl)propane, **14(*R,R*)**

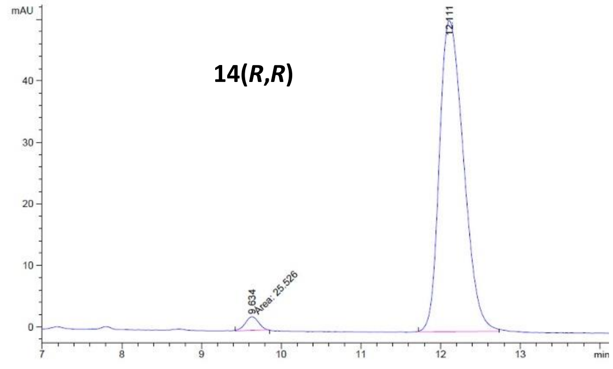

Peak #	RetTime [min]	Area %
1	9.634	2.3417
2	12.111	97.6583

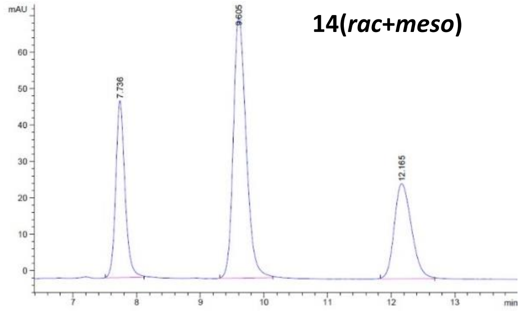

Peak #	RetTime [min]	Area %
1	7.736	24.6844
2	9.605	50.6077
3	12.165	24.7079

(S,S)-1,3-Bis(methylsulfinyl)propane, **14(*S,S*)**

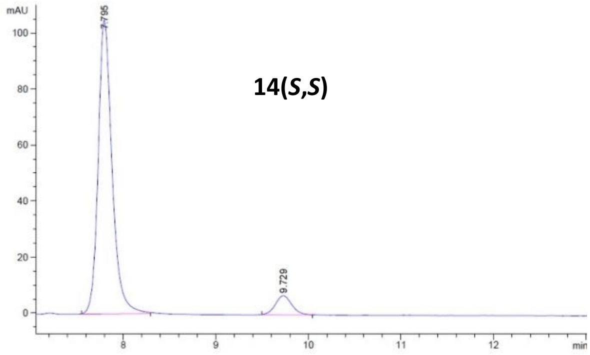

Peak #	RetTime [min]	Area %
1	7.795	92.7104
2	9.729	7.2896

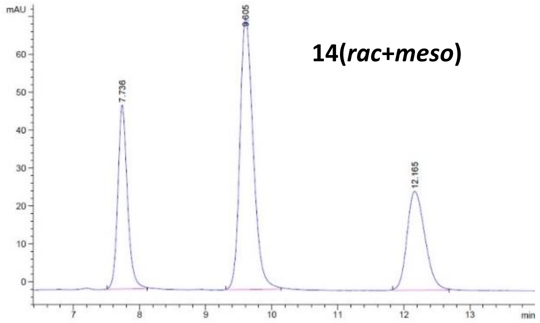

Peak #	RetTime [min]	Area %
1	7.736	24.6844
2	9.605	50.6077
3	12.165	24.7079

(R,R)-1,3-Bis(ethylsulfinyl)propane, **15(*R*,*R*)**

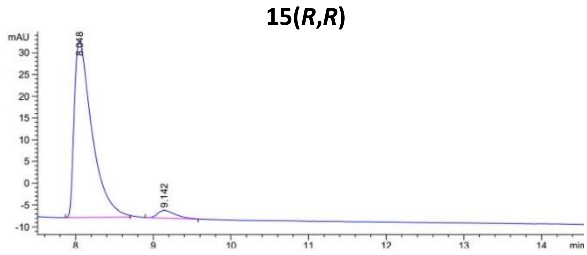

Peak #	RetTime [min]	Area %
1	8.048	95.8178
2	9.142	4.1822

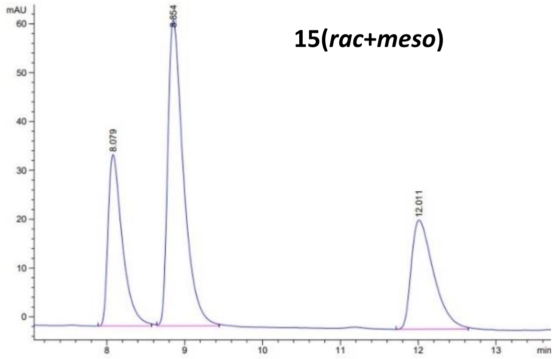

Peak #	RetTime [min]	Area %
1	8.079	25.2953
2	8.854	49.5110
3	12.011	25.1937

(S,S)-1,3-Bis(ethylsulfinyl)propane, **15(*S*,*S*)**

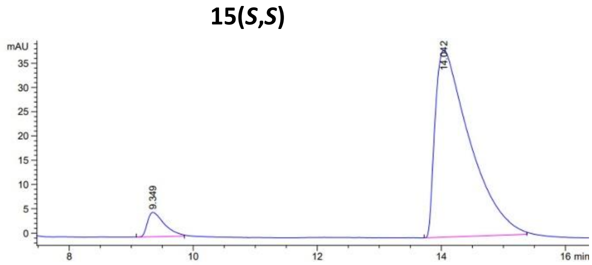

Peak #	RetTime [min]	Area %
1	9.349	5.9592
2	14.042	94.0408

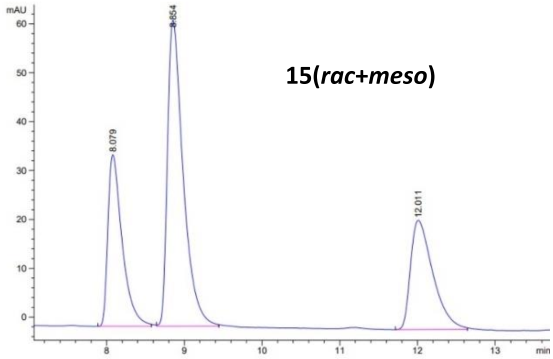

Peak #	RetTime [min]	Area %
1	8.079	25.2953
2	8.854	49.5110
3	12.011	25.1937

(R,R)-1,3-Bis(propylsulfinyl)propane, **16(*R*,*R*)**

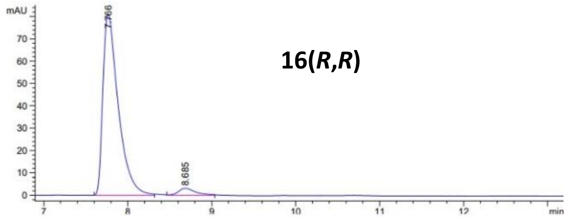

Peak #	RetTime [min]	Area %
1	7.766	95.7409
2	8.685	4.2591

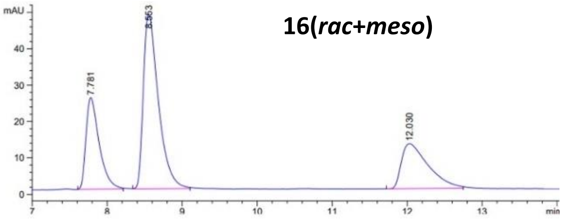

Peak #	RetTime [min]	Area %
1	7.781	24.0827
2	8.553	52.2154
3	12.030	23.7019

(S,S)-1,3-Bis(isopropylsulfinyl)propane, **17(*S,S*)**

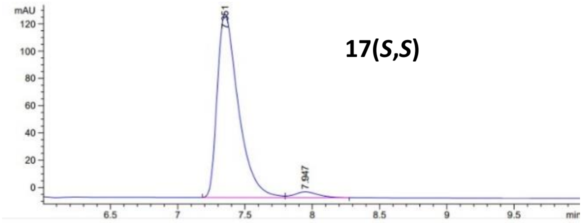

Peak #	RetTime [min]	Area %
1	7.351	96.6138
2	7.947	3.3862

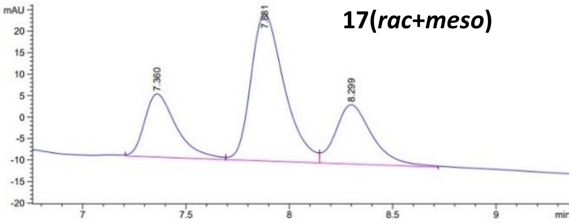

Peak #	RetTime [min]	Area %
1	7.360	22.1975
2	7.881	53.9722
3	8.299	23.8302

(S,S)-1,3-Bis(tert-butylsulfinyl)propane, **18(*S,S*)**

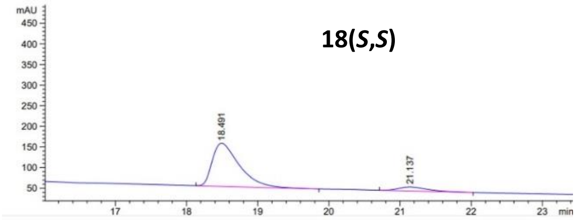

Peak #	RetTime [min]	Area %
1	18.491	90.9253
2	21.137	9.0747

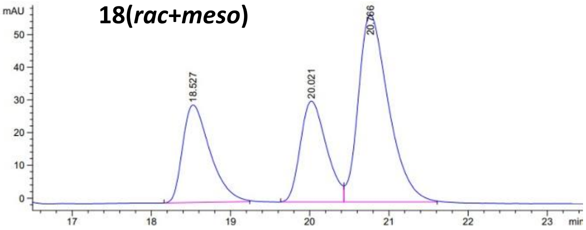

Peak #	RetTime [min]	Area %
1	18.527	24.4809
2	20.021	23.9126
3	20.766	51.6066

<u>Alkyl and aryl vinyl sulfoxides</u>

(R)-p-Tolyl vinyl sulfoxide, **25(*R*)**

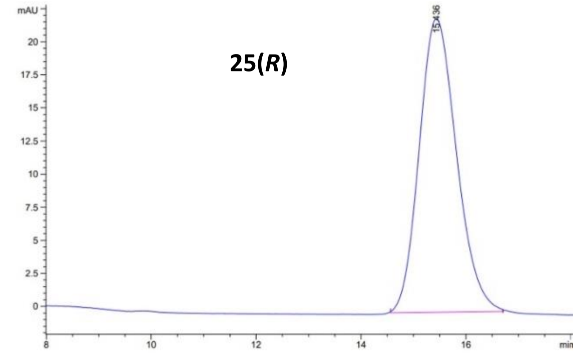

Peak #	RetTime [min]	Area %
1	15.434	100.0000

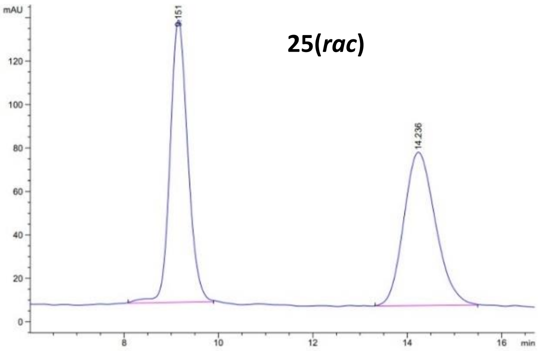

Peak #	RetTime [min]	Area %
1	9.151	50.7734
2	14.236	49.2266

(S)-p-Tolyl vinyl sulfoxide, **25(*S*)**

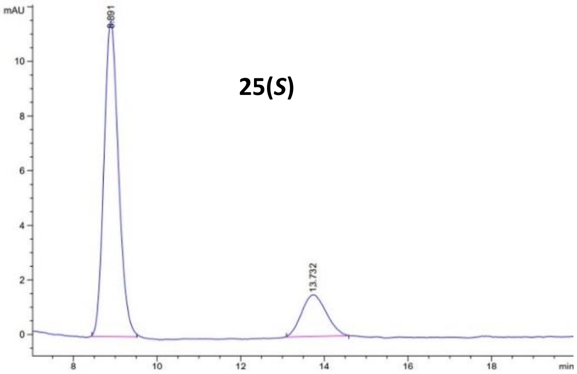

Peak #	RetTime [min]	Area %
1	8.891	81.5820
2	13.732	18.4180

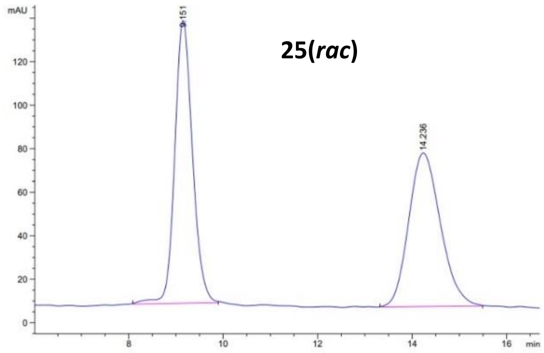

Peak #	RetTime [min]	Area %
1	9.151	50.7734
2	14.236	49.2266

(R)-tert-Butyl vinyl sulfoxide, **26(*R*)**

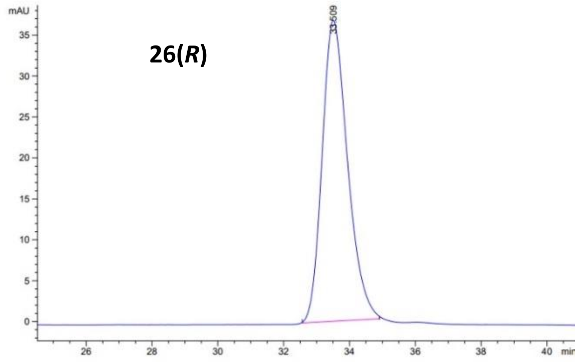

Peak #	RetTime [min]	Area %
1	33.509	100.0000

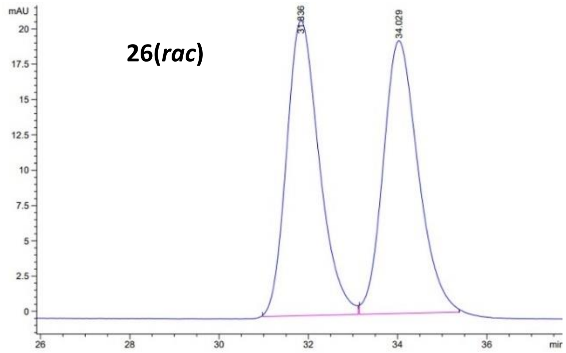

Peak #	RetTime [min]	Area %
1	31.836	50.2462
2	34.029	49.7538

(S)-tert-Butyl vinyl sulfoxide, **26(*S*)**

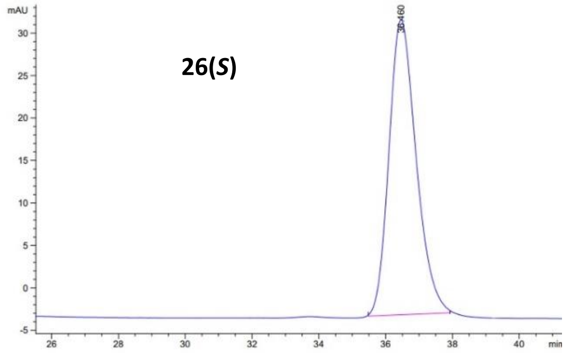

Peak #	RetTime [min]	Area %
1	36.460	100.0000

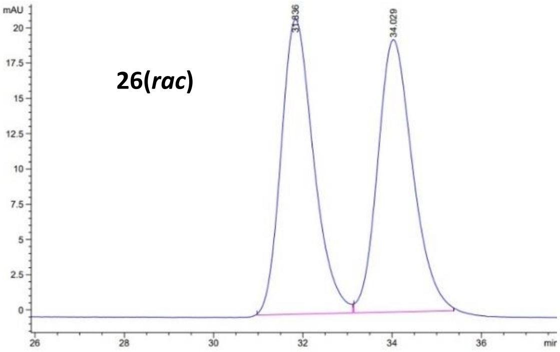

Peak #	RetTime [min]	Area %
1	31.836	50.2462
2	34.029	49.7538

1,5-bis(alkyl- and arylsulfinyl)-3-thioderivatives

(R,R)-Bis[2-(p-tolylsulfinyl)ethyl] sulfide, **28(*R*,*R*)**

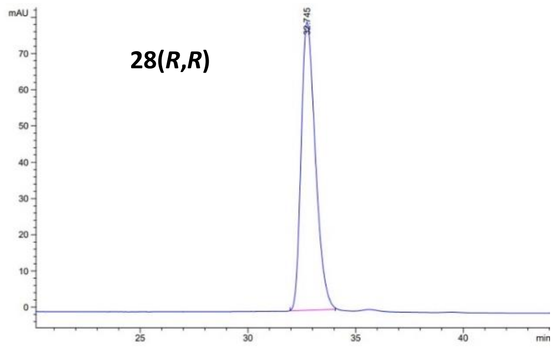

Peak #	RetTime [min]	Area %
1	32.745	100.0000

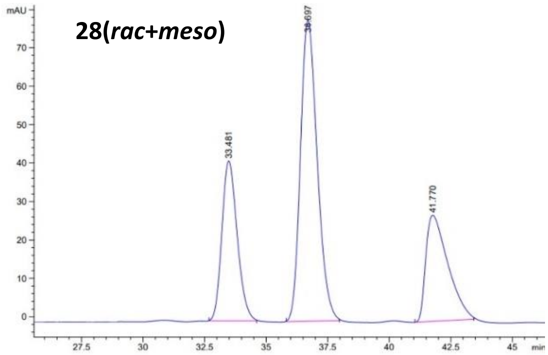

Peak #	RetTime [min]	Area %
1	33.481	24.8644
2	36.697	51.3029
3	41.770	23.8327

(S,S)-Bis[2-(p-tolylsulfinyl)ethyl] sulfide, **28(*S*,*S*)**

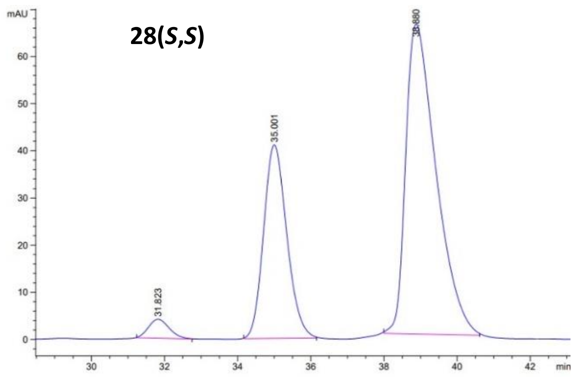

Peak #	RetTime [min]	Area %
1	31.823	2.7401
2	35.001	31.4324
3	38.880	65.8276

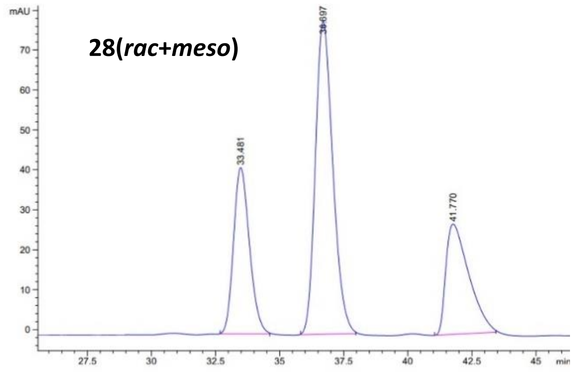

Peak #	RetTime [min]	Area %
1	33.481	24.8644
2	36.697	51.3029
3	41.770	23.8327

(R,R)-Bis[2-(tert-butylsulfinyl)ethyl] sulfide, **29(*R*,*R*)**

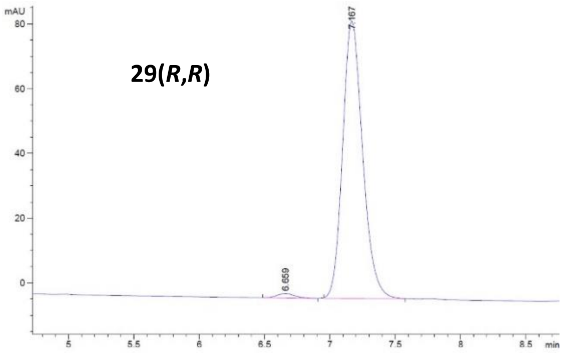

Peak #	RetTime [min]	Area %
1	6.659	1.3543
2	7.167	98.6457

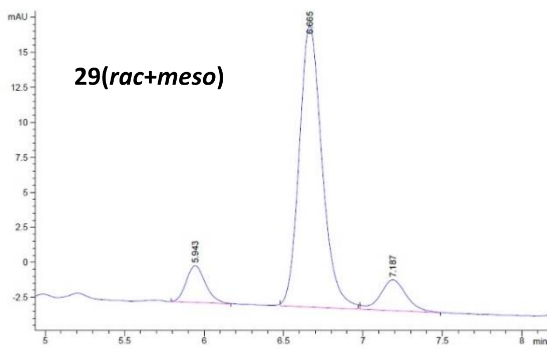

Peak #	RetTime [min]	Area %
1	5.943	8.8999
2	6.665	81.0571
3	7.187	10.0430

(S,S)-Bis[2-(tert-butylsulfinyl)ethyl] sulfide, **29(*S,S*)**

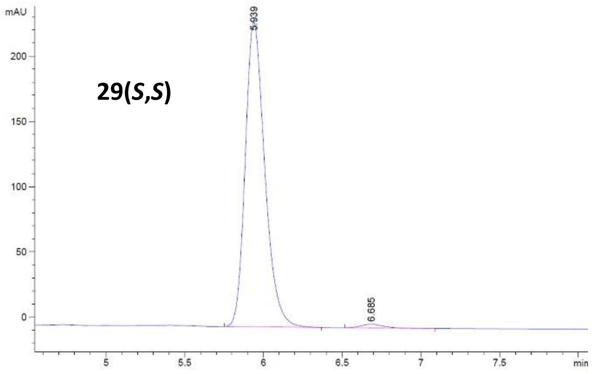

Peak #	RetTime [min]	Area %
1	5.939	98.6677
2	6.685	1.3323

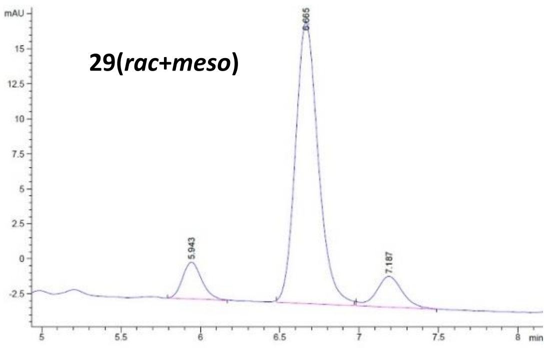

Peak #	RetTime [min]	Area %
1	5.943	8.8999
2	6.665	81.0571
3	7.187	10.0430

(S,S)-Bis[2-(methylsulfinyl)ethyl] sulfide, **30(*S,S*)**

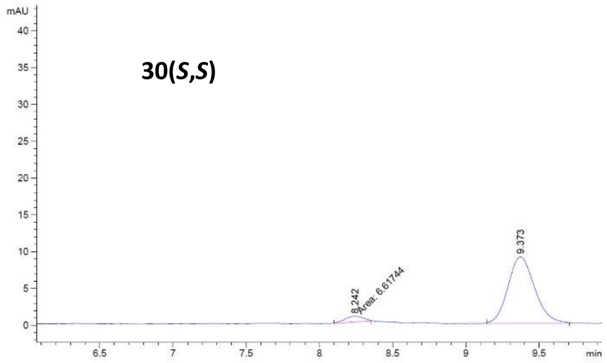

Peak #	RetTime [min]	Area %
1	8.242	5.4518
2	9.373	94.5482

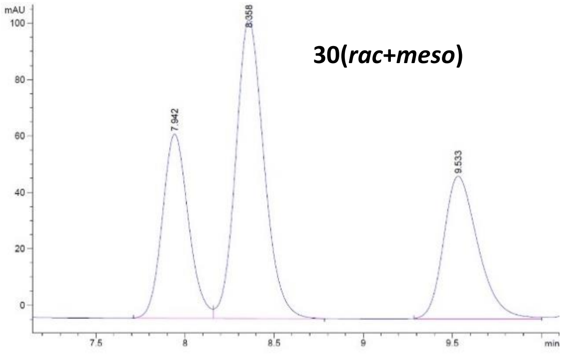

Peak #	RetTime [min]	Area %
1	7.942	26.4264
2	8.358	46.5036
3	9.533	27.0700

(S,S)-Bis[2-(methylsulfinyl)ethyl] sulfide, **30(*R,R*)**

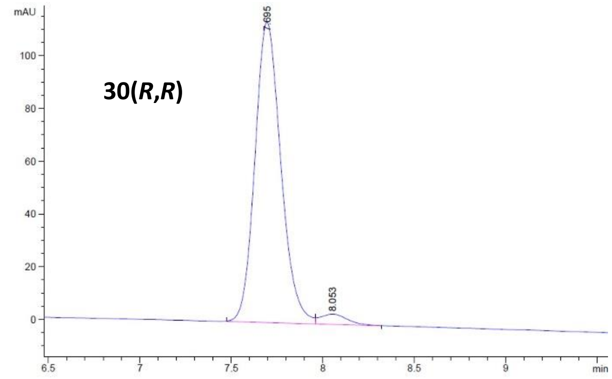

Peak #	RetTime [min]	Area %
1	7.695	96.6302
2	8.053	3.3698

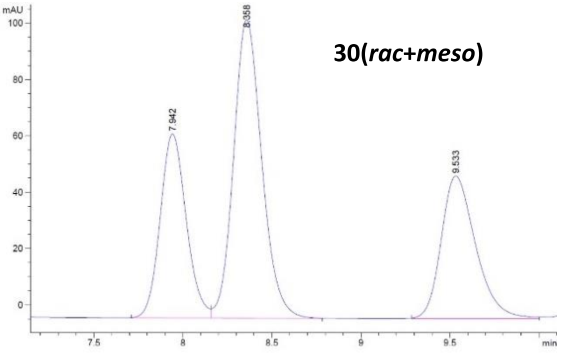

Peak #	RetTime [min]	Area %
1	7.942	26.4264
2	8.358	46.5036
3	9.533	27.0700

^{1}H NMR, ^{13}C{^{1}H} NMR, ^{19}F NMR of selected compounds

Propane-1,3-diyl dimethanesulfonate, **2**

^{1}H NMR (500 MHz, $CDCl_3$)

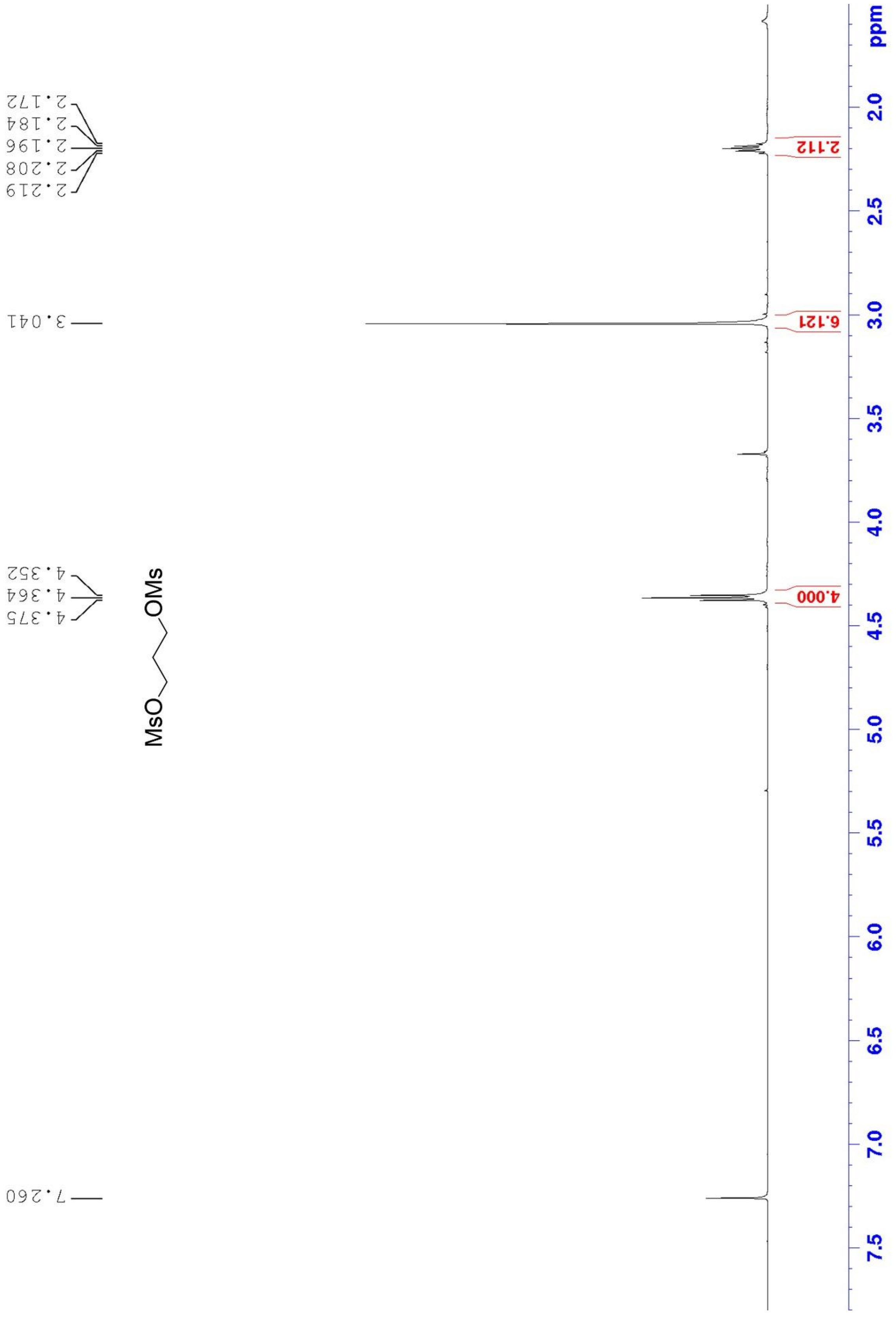

$^{13}C\{^1H\}$ NMR (125 MHz, $CDCl_3$)

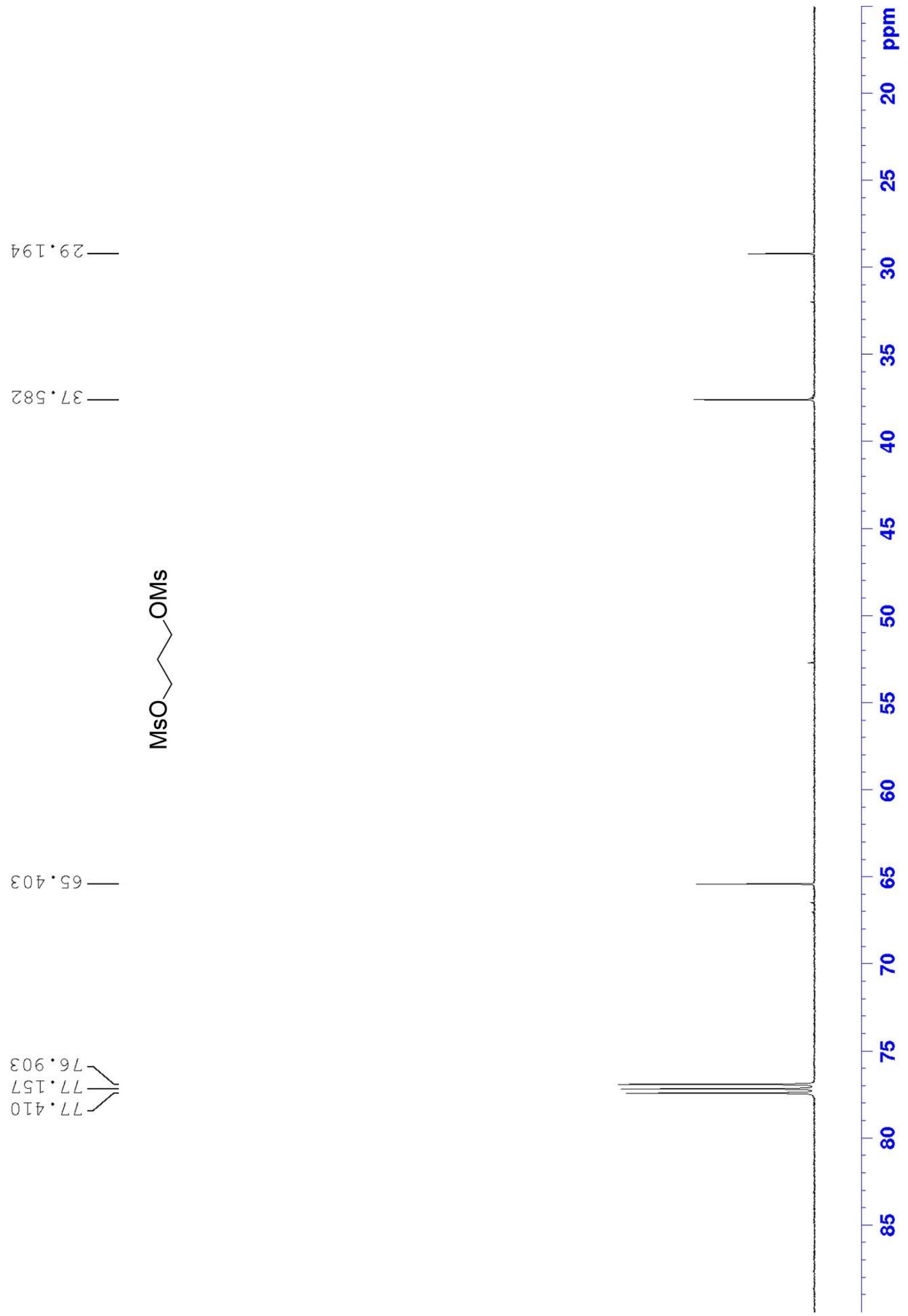

Propane-1,3-diyl dithioacetate, **3**

^{1}H NMR (500 MHz, $CDCl_3$)

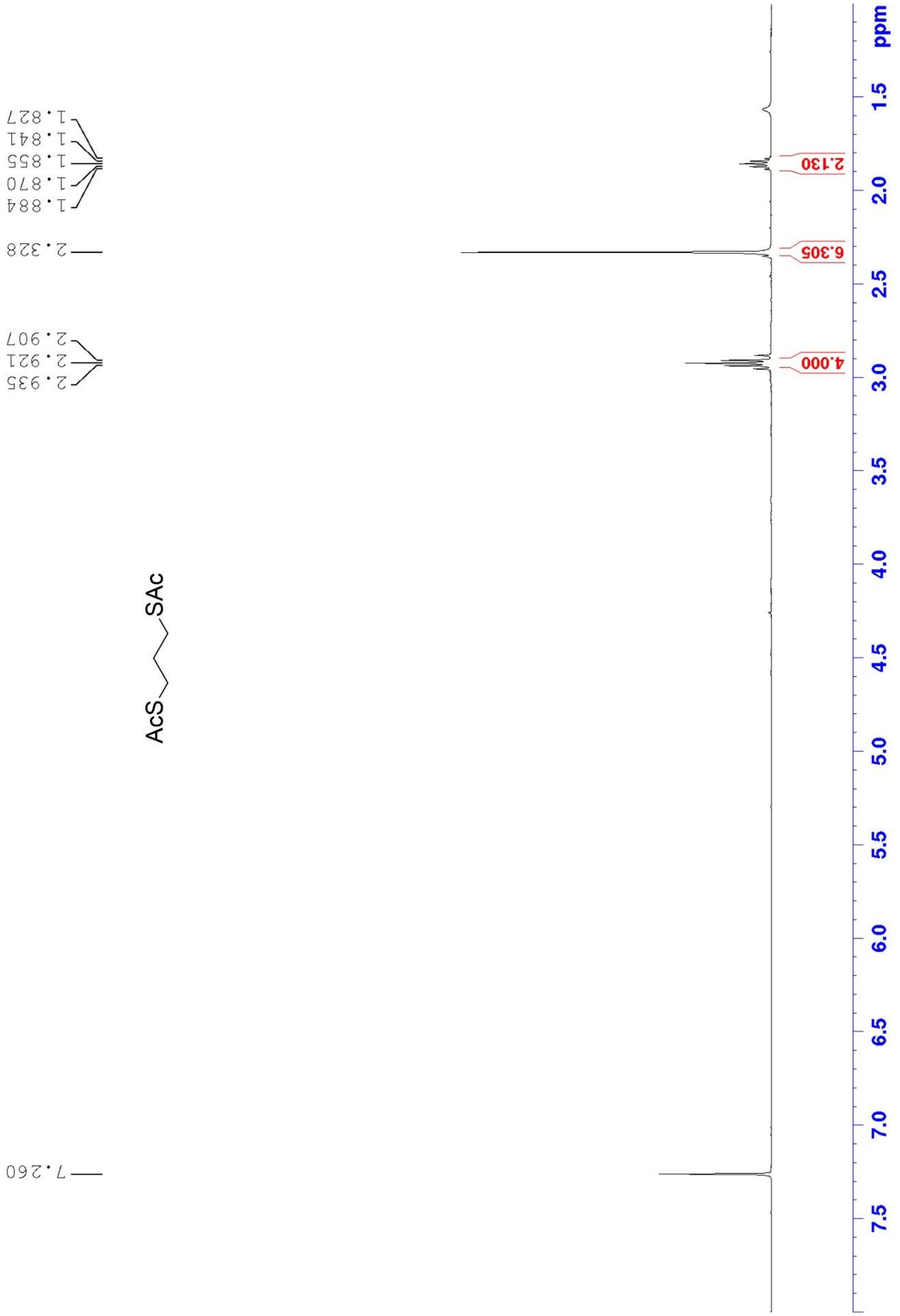

$^{13}C\{^{1}H\}$ NMR (125 MHz, $CDCl_3$)

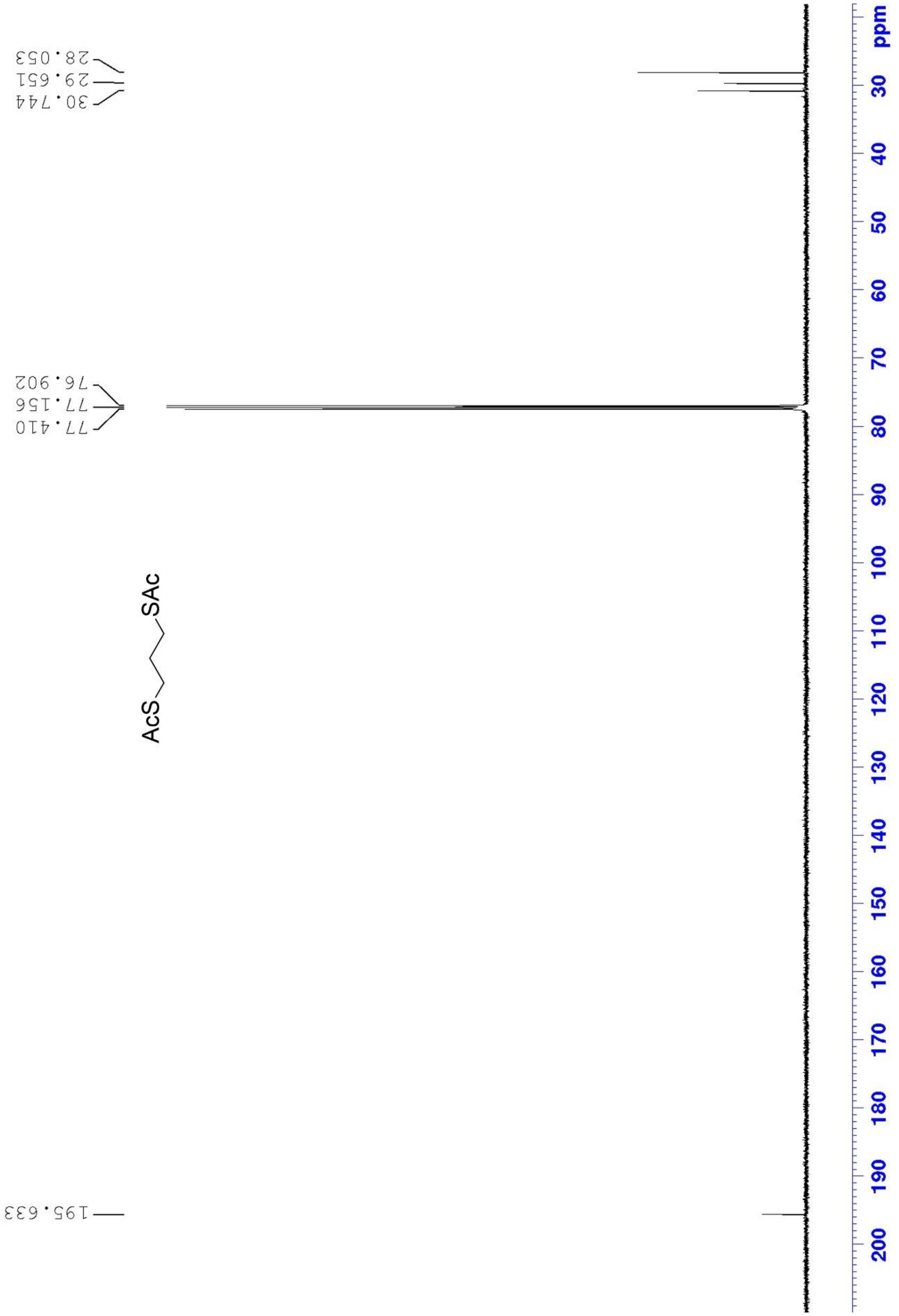

Propane-1,3-bis(sulfinyl) chloride, **4**

^{1}H NMR (300 MHz, $CDCl_3$)

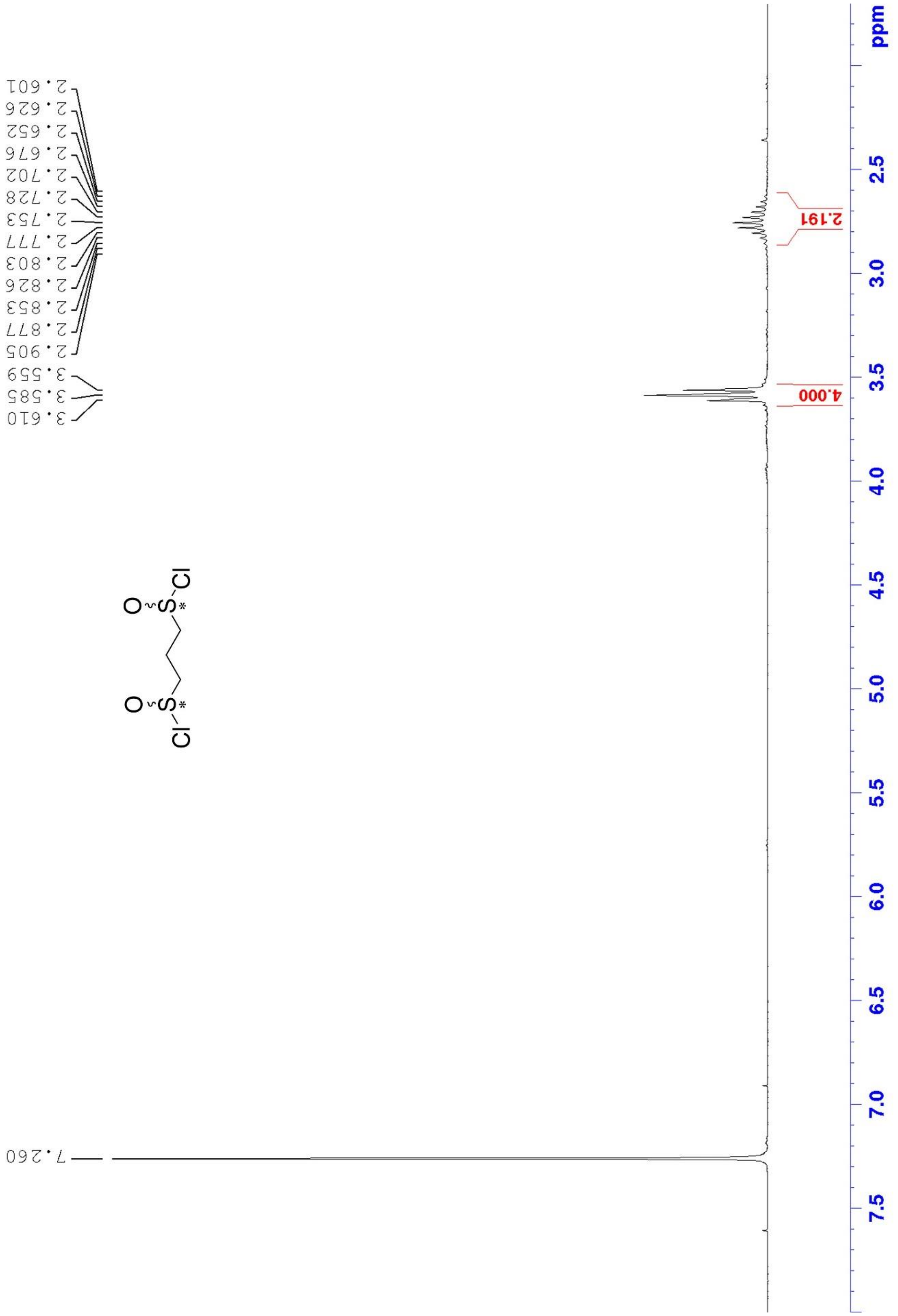

Di(1,2:5,6-di-O-cyclohexylidene-α-D-glucofuranosyl) (S,S)-Propane-1,3-bis(sulfinate), **5(*S,S*)**

^{1}H NMR (500 MHz, C_6D_6)

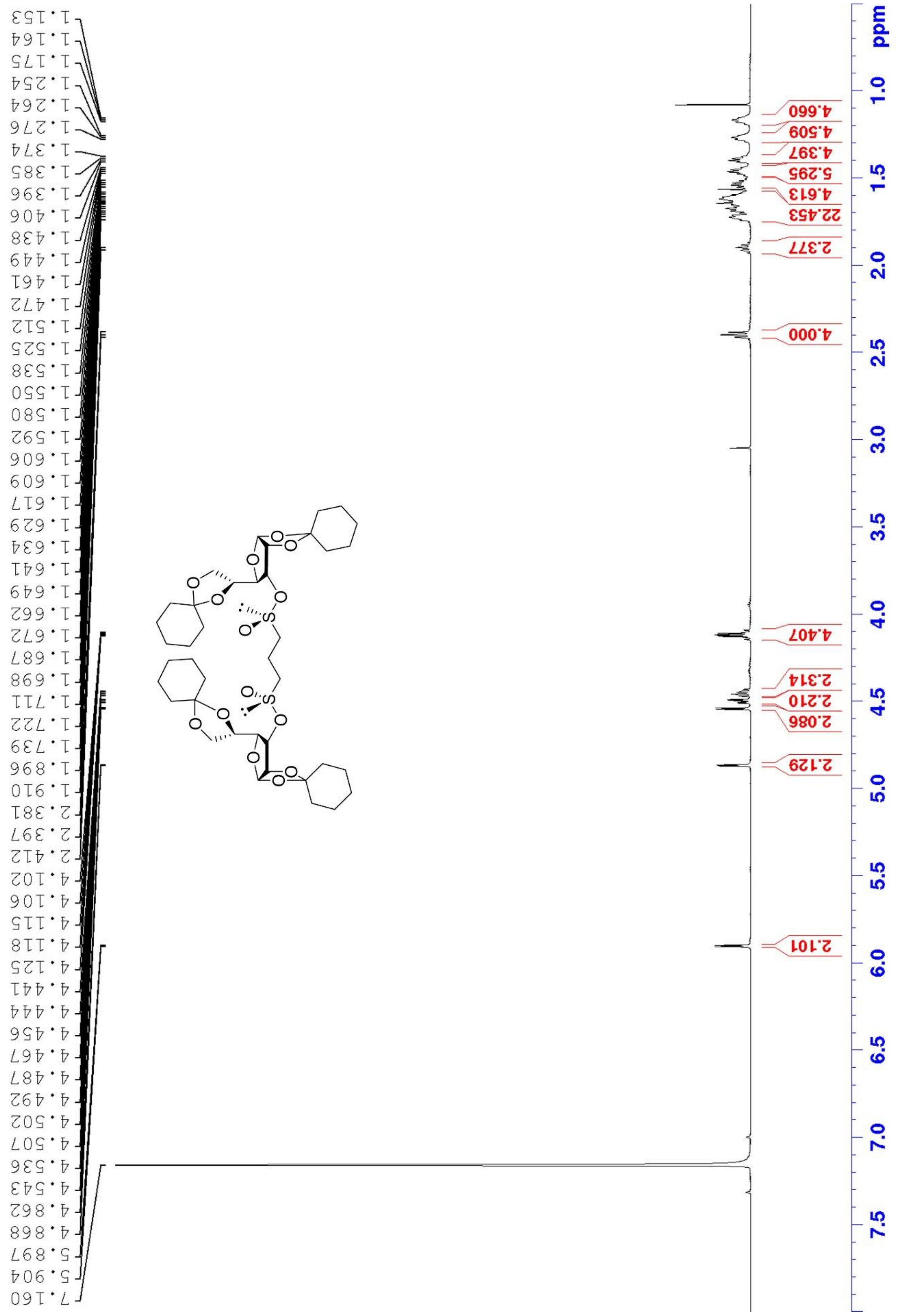

$^{13}C\{^{1}H\}$ NMR (125 MHz, C_6D_6)

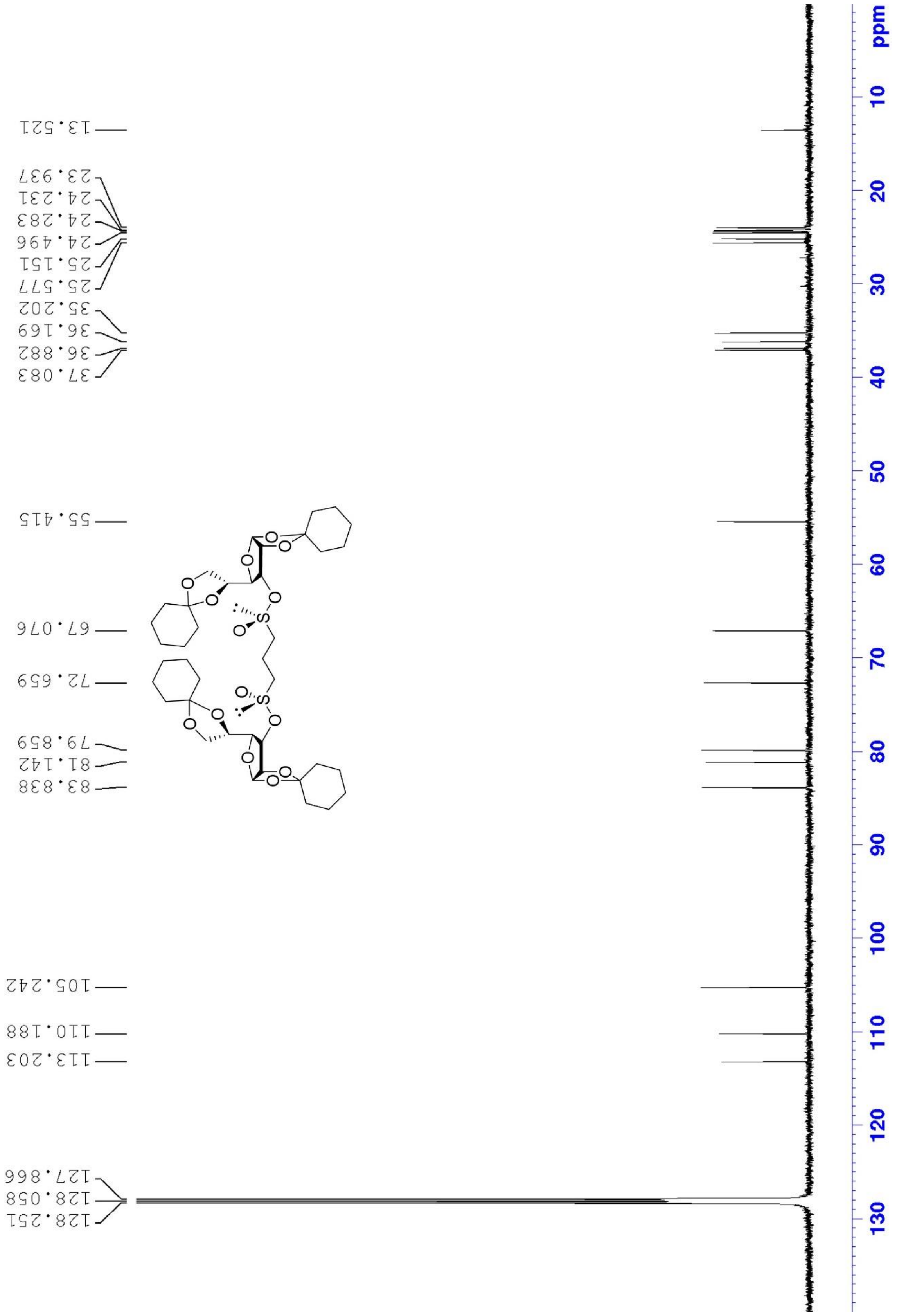

Di(1,2:5,6-di-O-cyclohexylidene-α-D-glucofuranosyl) (R,R)-Propane-1,3-bis(sulfinate), **5(*R*,*R*)**

^{1}H NMR (500 MHz, C_6D_6)

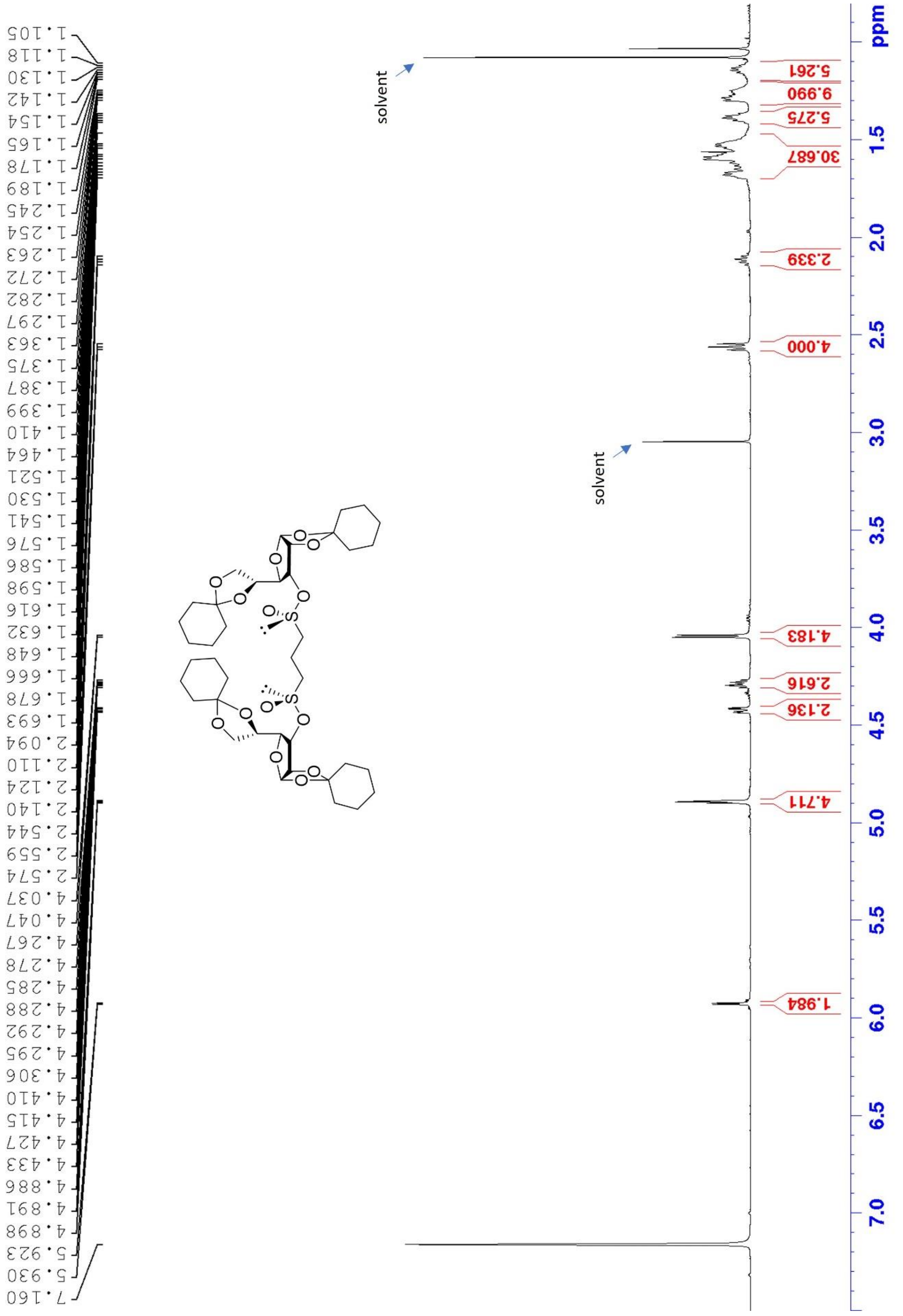

$^{13}C\{^1H\}$ NMR (125 MHz, C_6D_6)

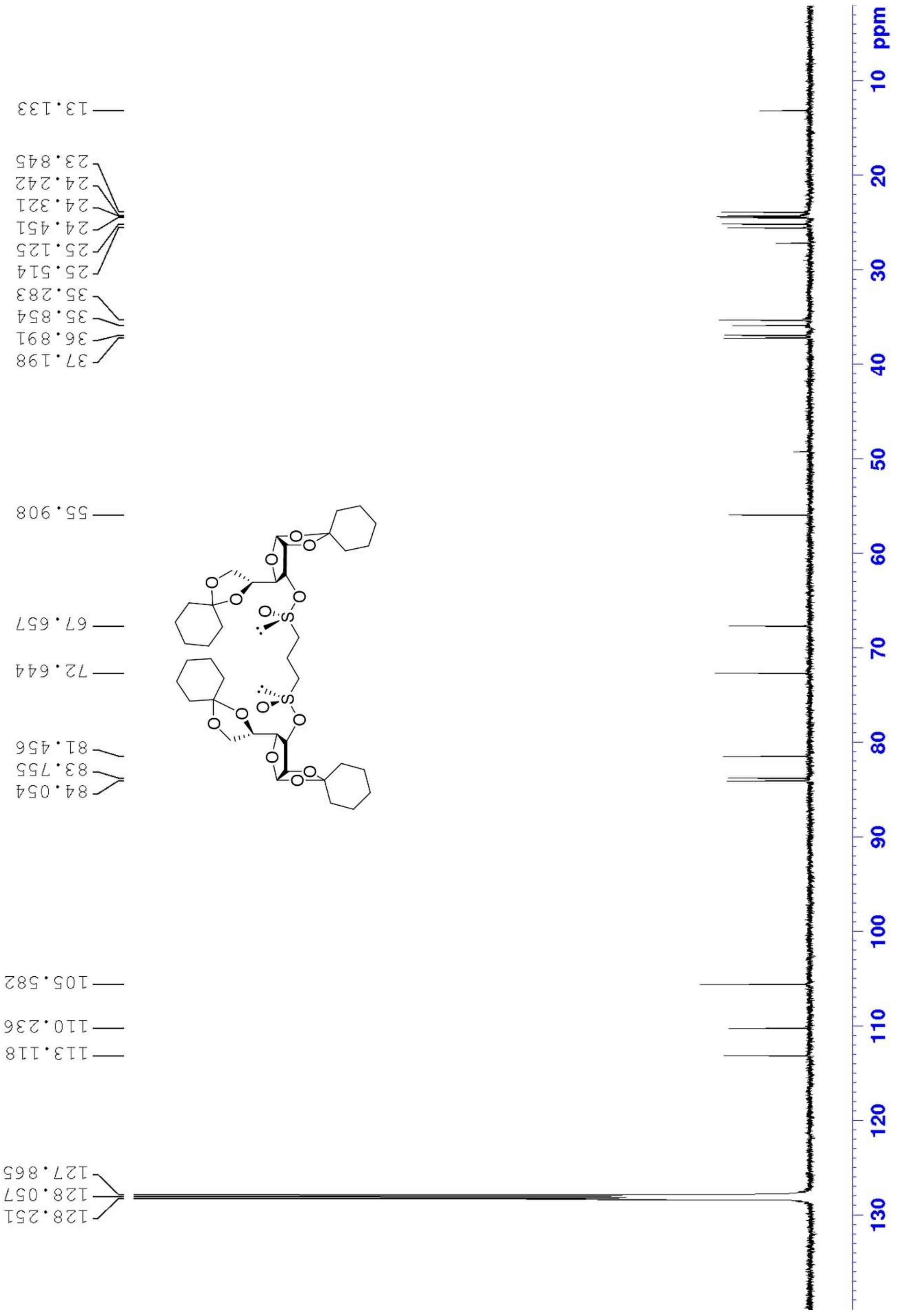

Di(1,2:5,6-di-O-cyclohexylidene-α-D-glucofuranosyl) (R,S)-Propane-1,3-bis(sulfinate), **5(*R,S*)**

^{1}H NMR (500 MHz, C_6D_6)

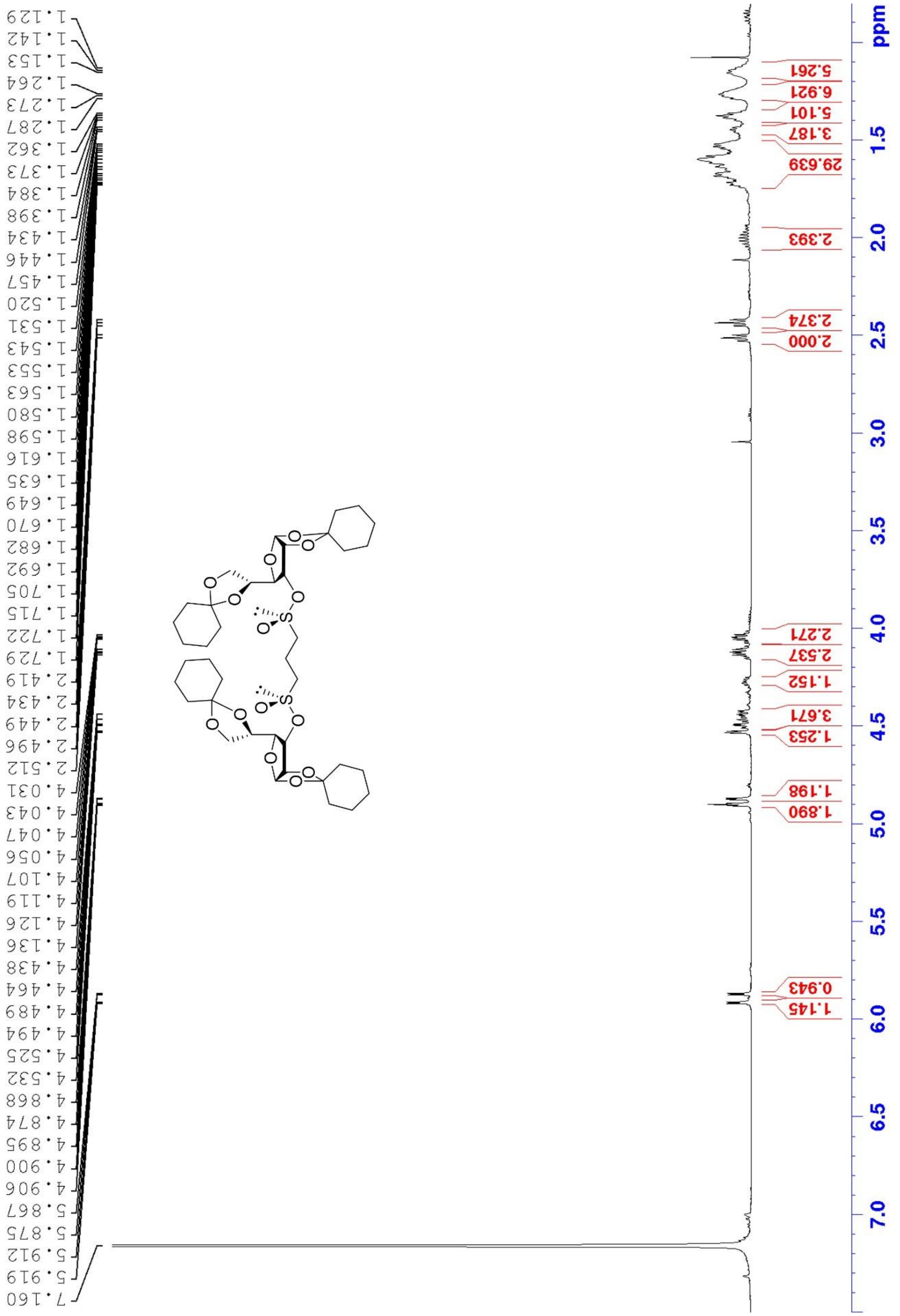

$^{13}C\{^1H\}$ NMR (125 MHz, C_6D_6)

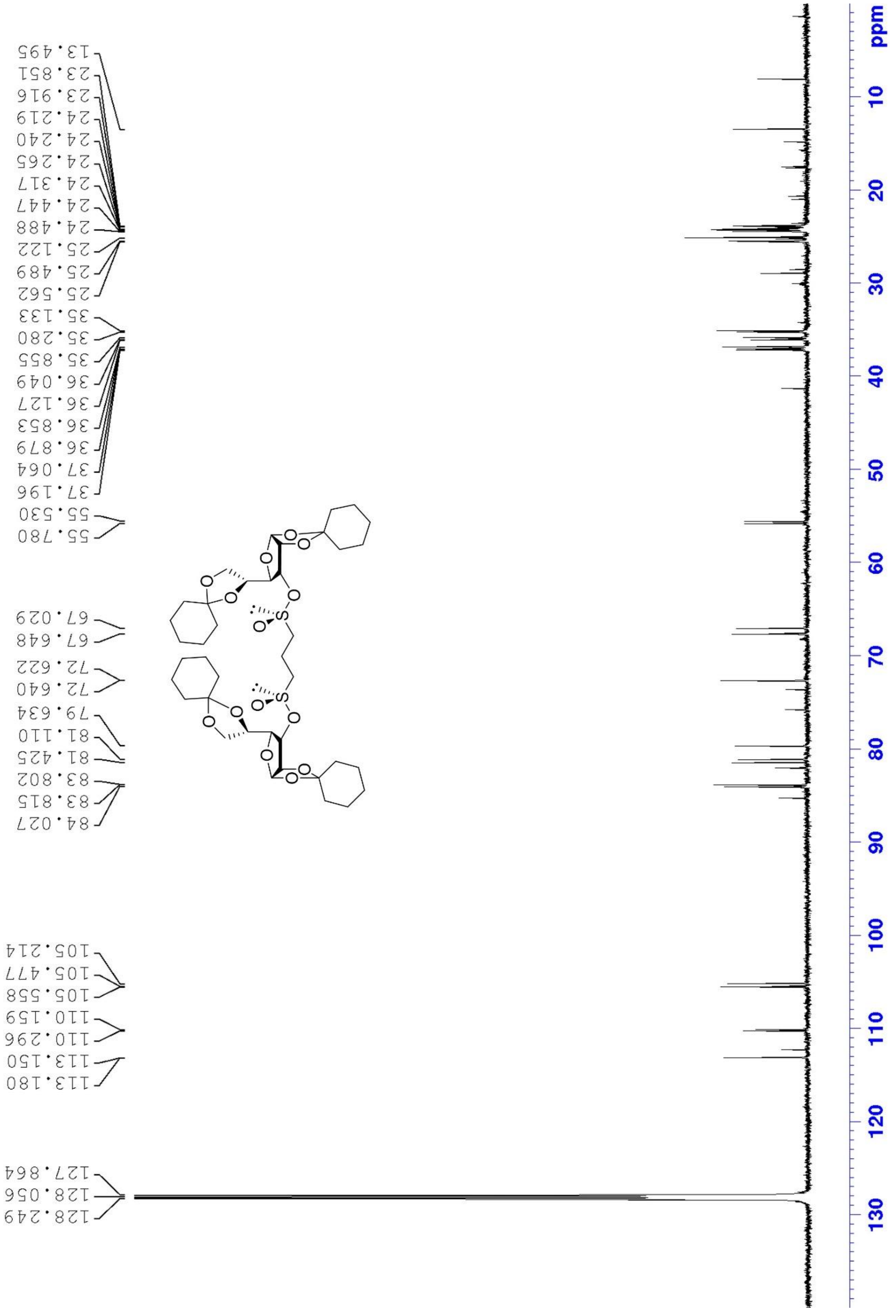

(S,S)-1,3-Bis(phenylsulfinyl)propane, **8(*S*,*S*)**

^{1}H NMR (500 MHz, $CDCl_3$)

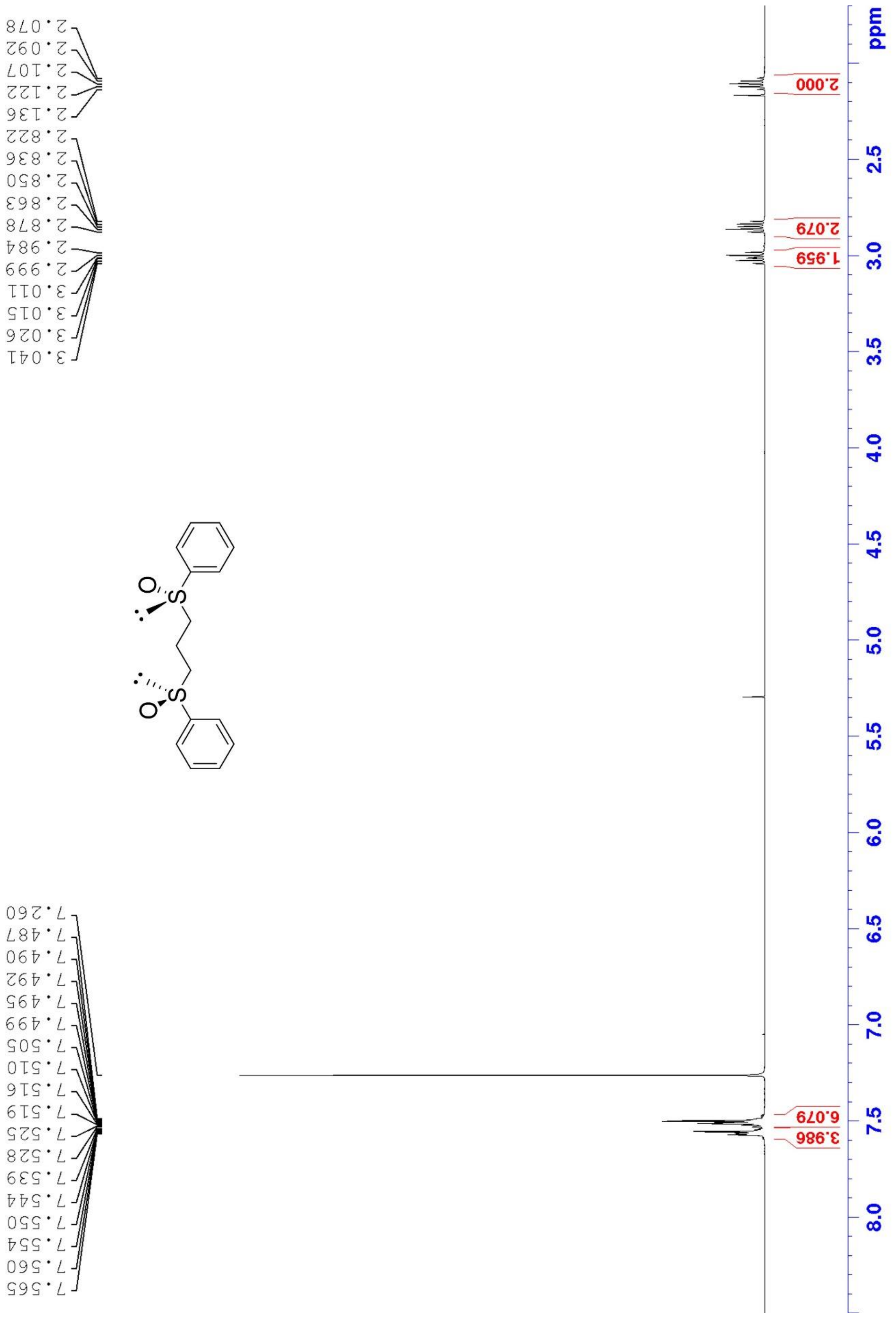

$^{13}C\{^{1}H\}$ NMR (125 MHz, $CDCl_3$)

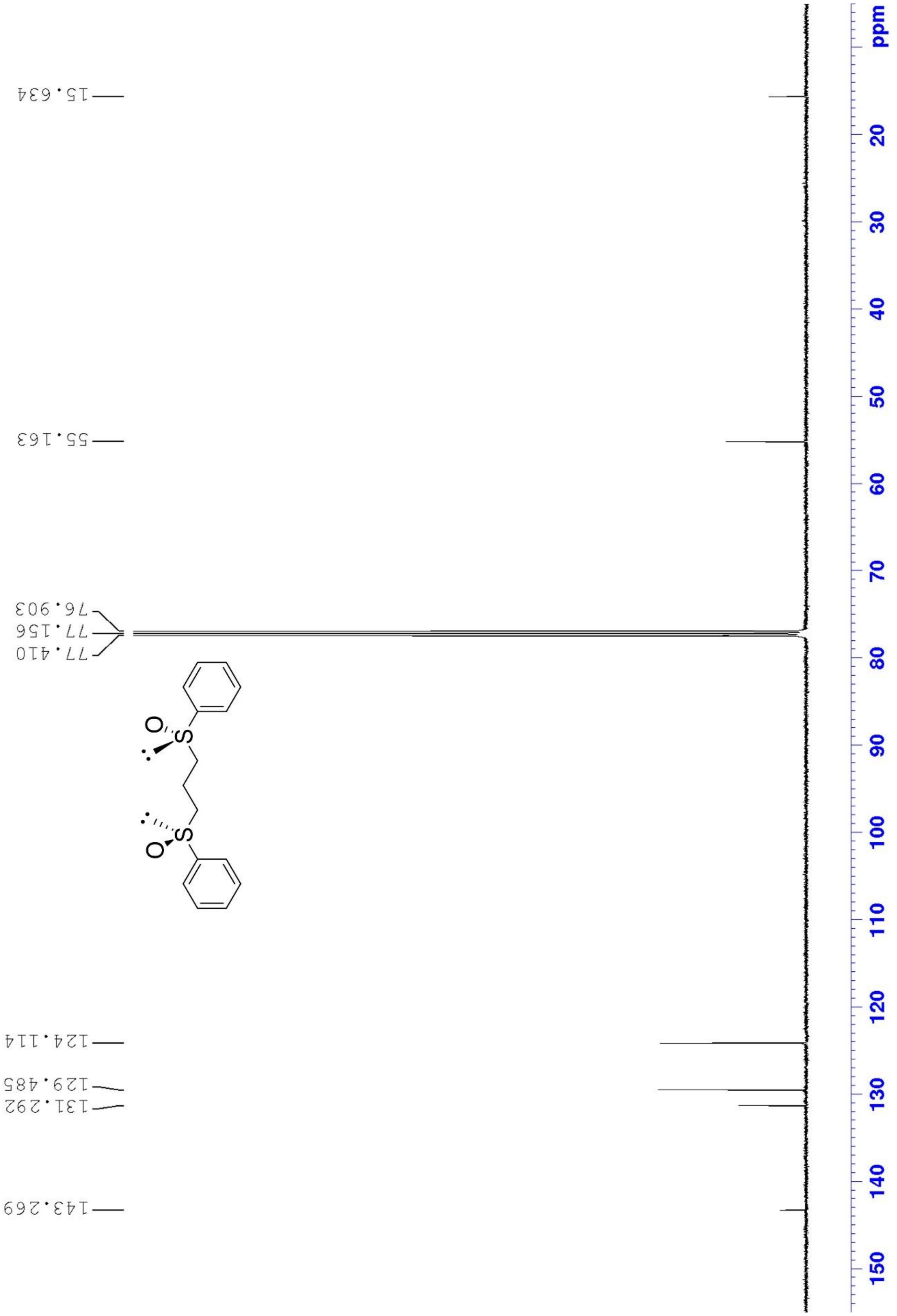

(S,S)-Bis(1-naphthylsulfinyl)propane, **9(*S*,*S*)**

^{1}H NMR (500 MHz, $CDCl_3$)

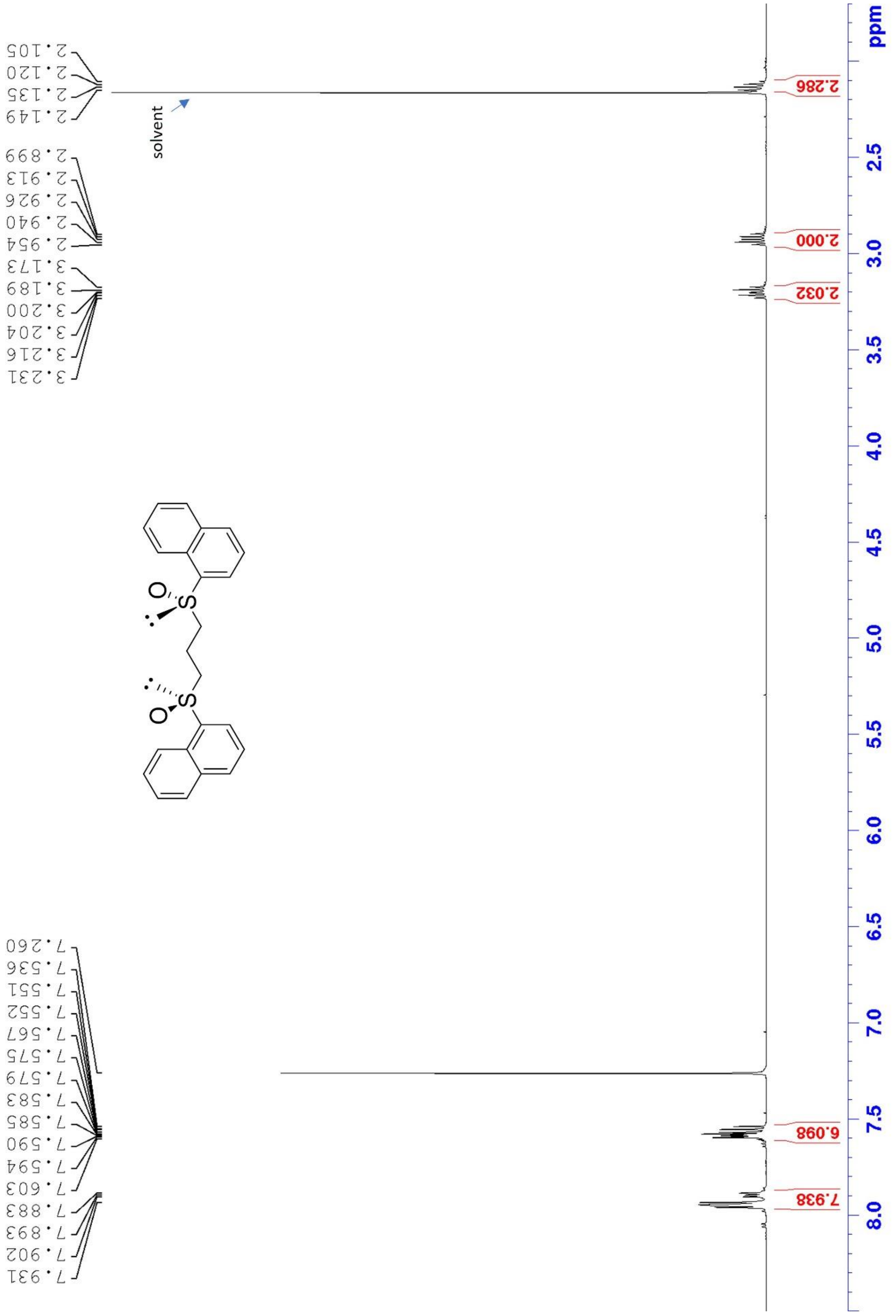

$^{13}C\{^{1}H\}$ NMR (125 MHz, **$CDCl_3$**)

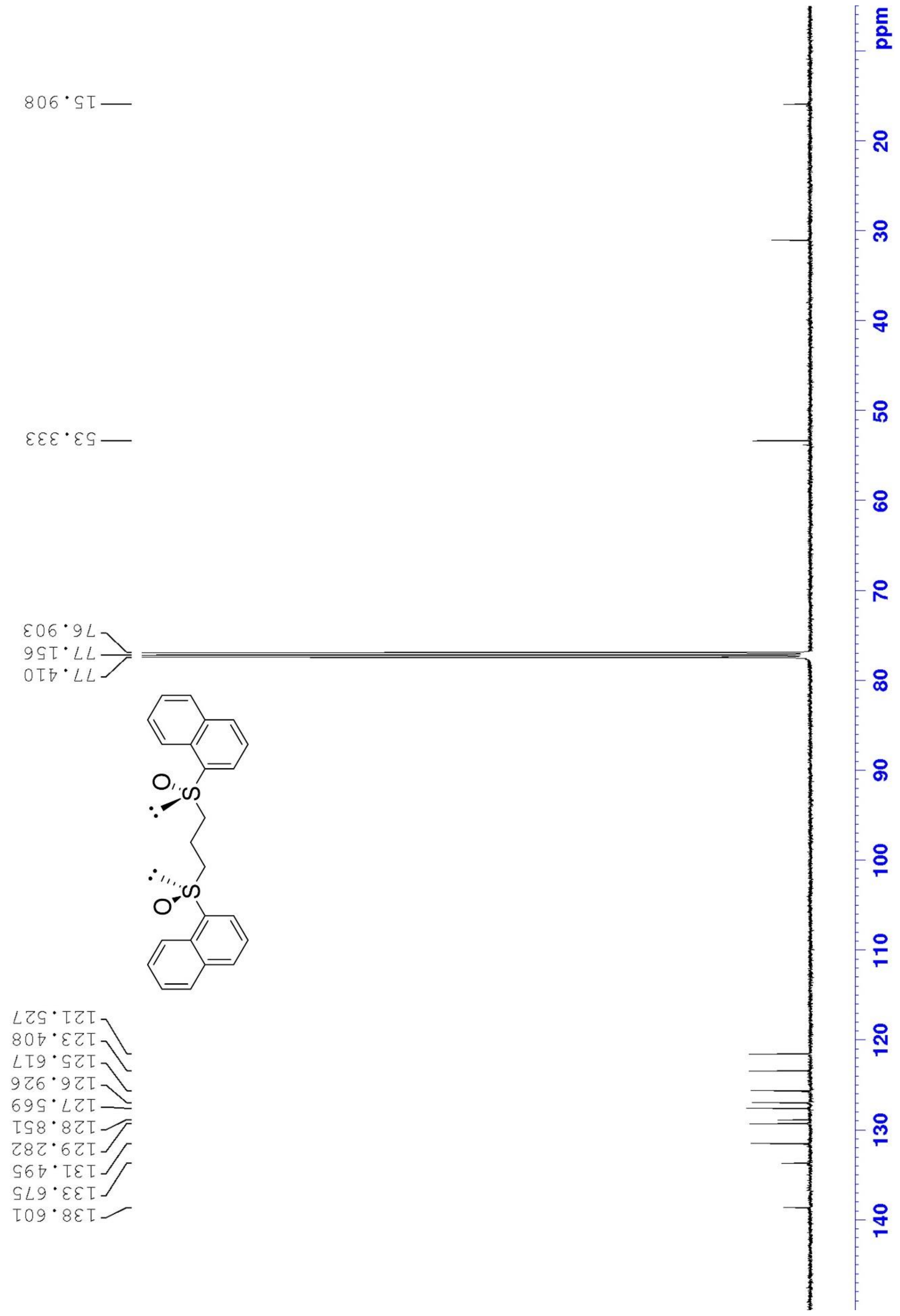

(S,S)-1,3-Bis[(2,6-dimethylphenyl)sulfinyl]propane, **10(*S,S*)**

^{1}H NMR (500 MHz, $CDCl_3$)

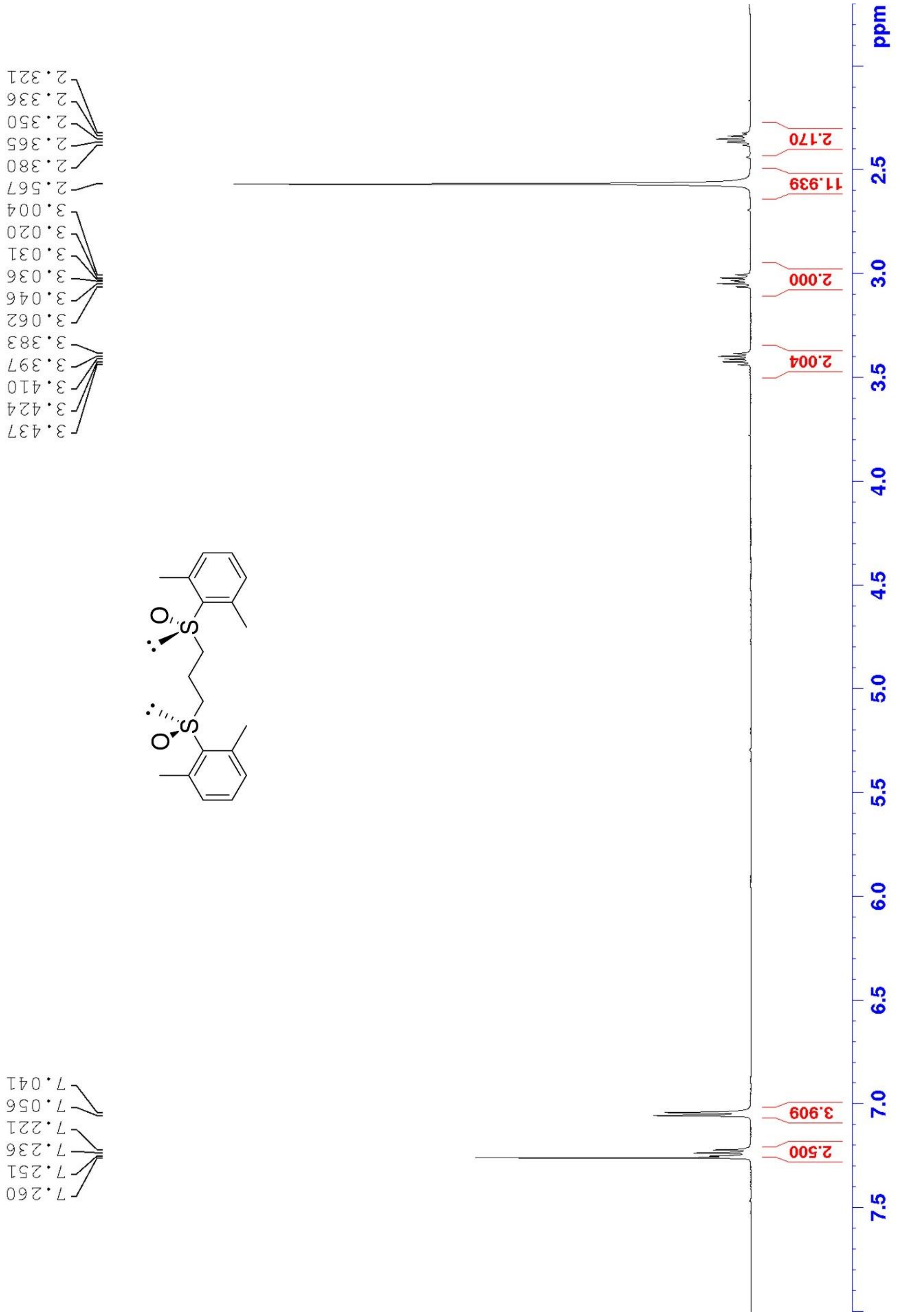

$^{13}C\{^1H\}$ NMR (125 MHz, $CDCl_3$)

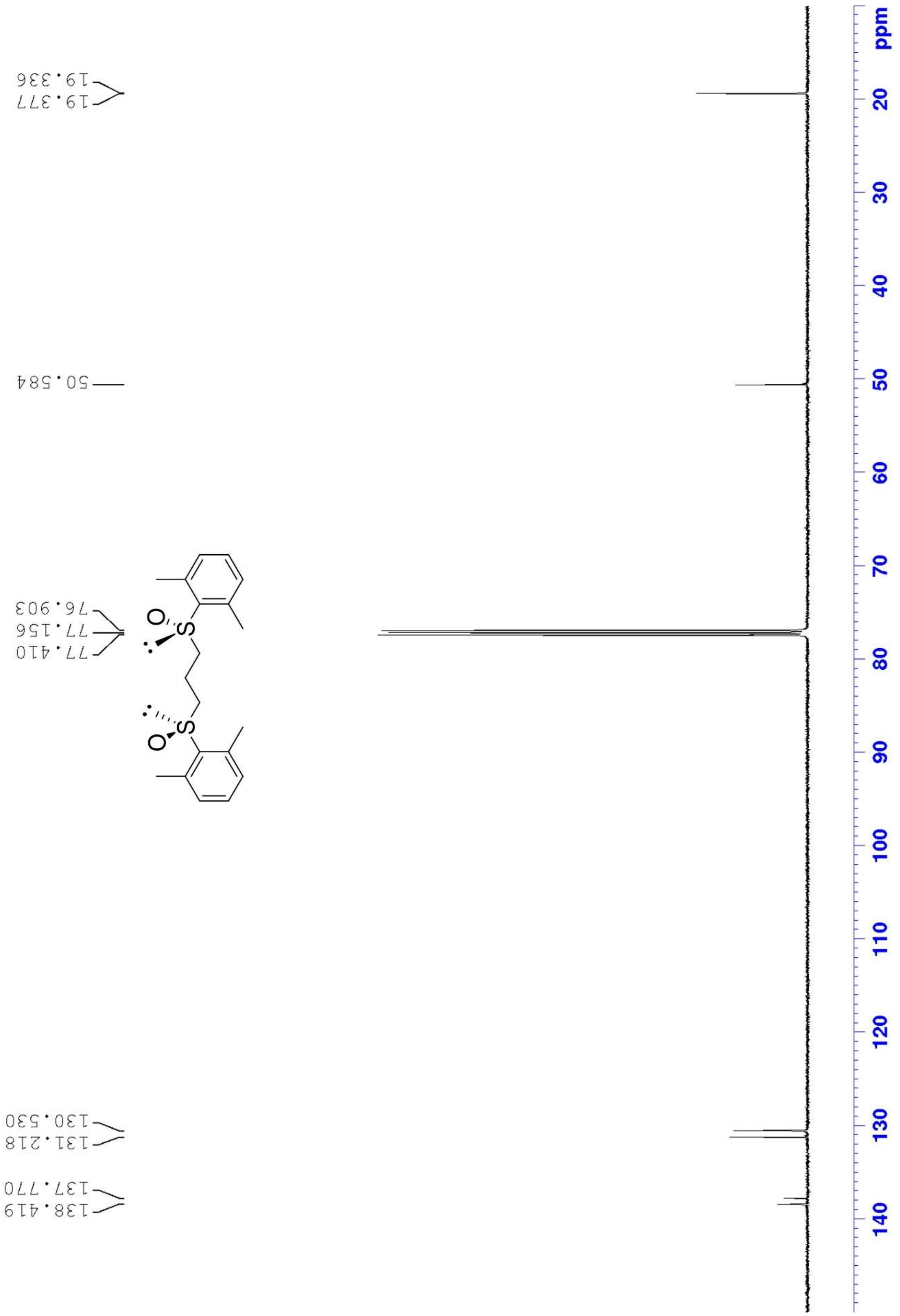

(R,R)-1,3-Bis[(2,6-dimethylphenyl)sulfinyl]propane, **10(*R*,*R*)**

^{1}H NMR (500 MHz, $CDCl_3$)

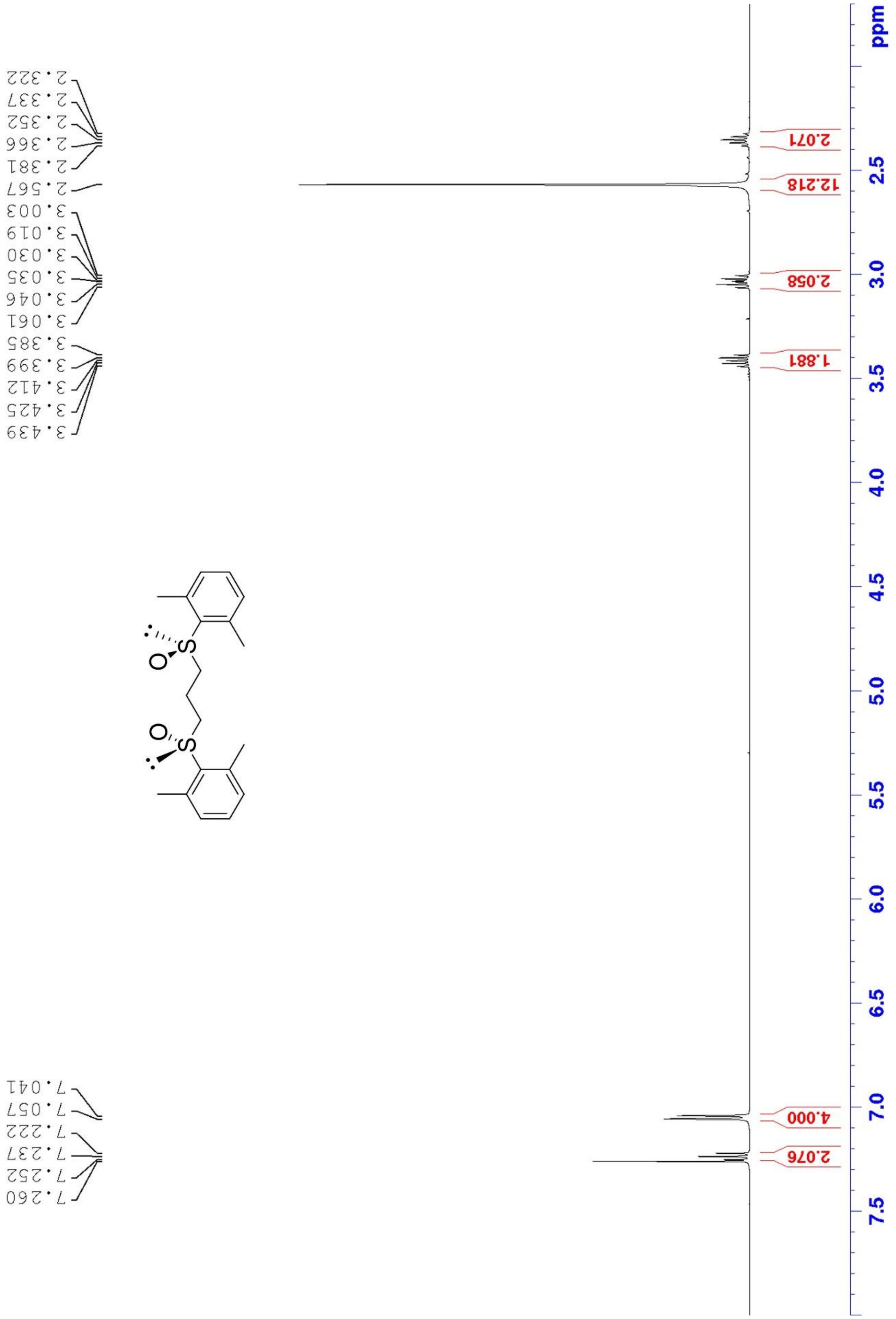

$^{13}C\{^{1}H\}$ NMR (125 MHz, $CDCl_3$)

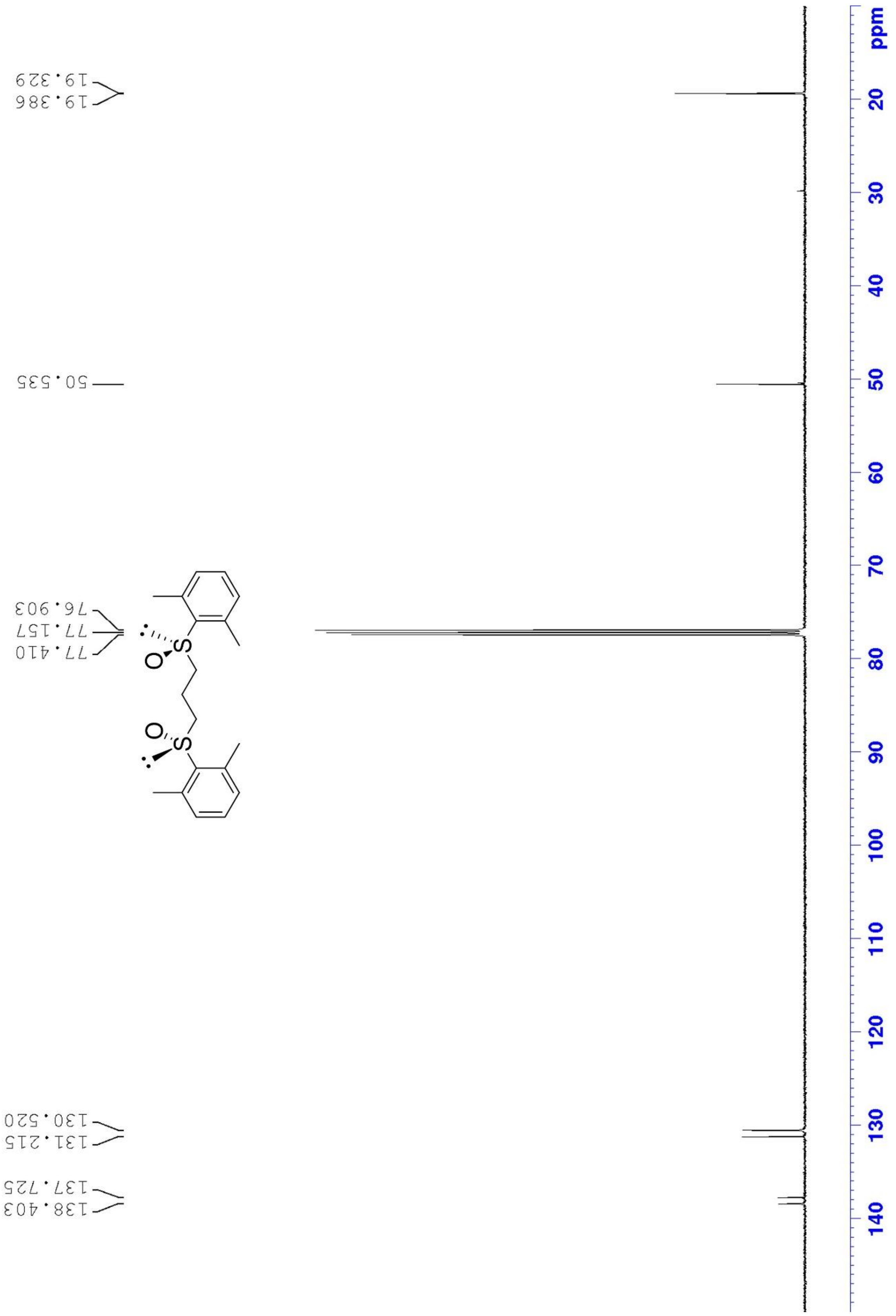

(S,S)-1,3-Bis(p-tolylsulfinyl)propane, **11(*S,S*)**

^{1}H NMR (500 MHz, MeOD)

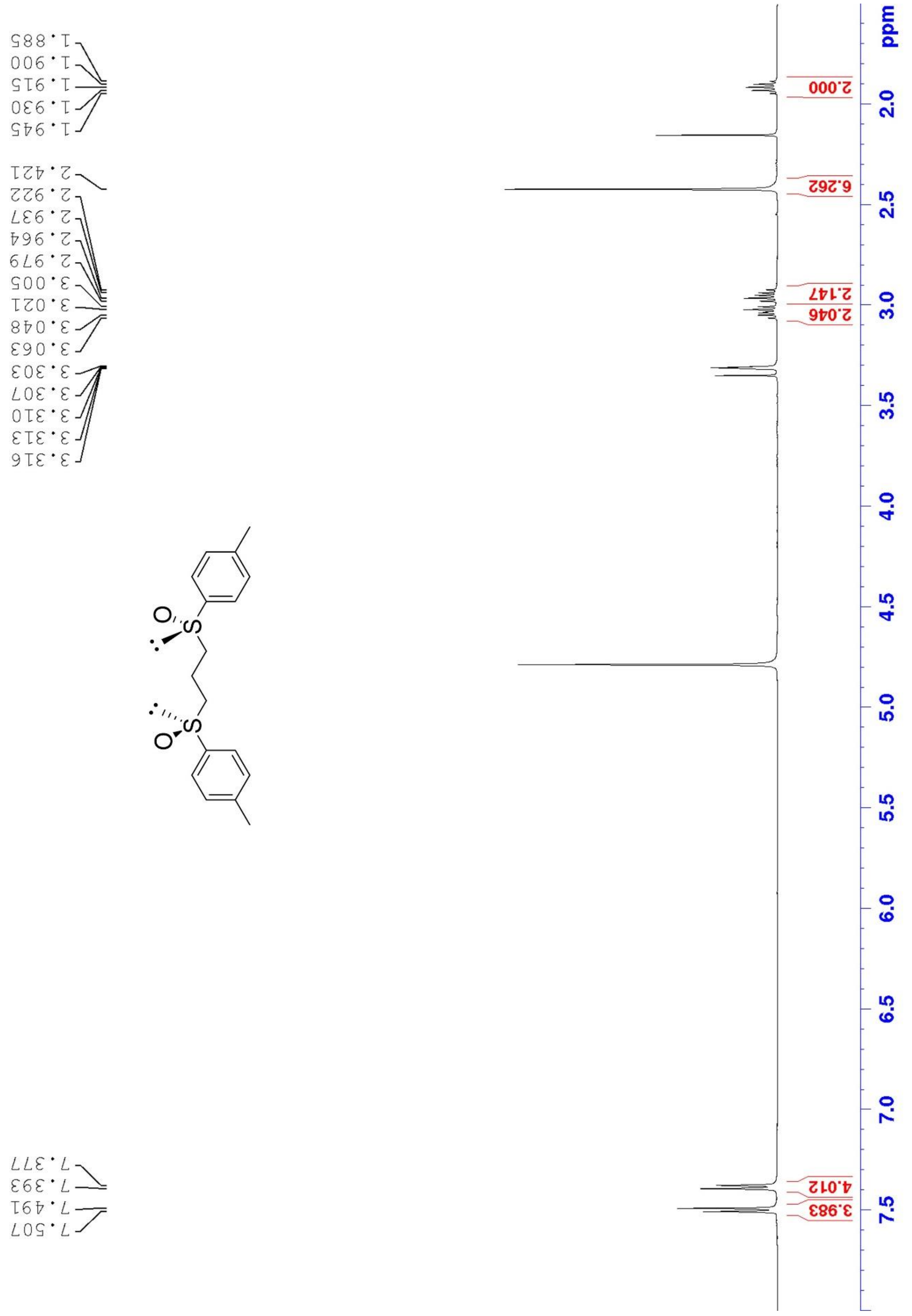

$^{13}C\{^1H\}$ NMR (125 MHz, MeOD)

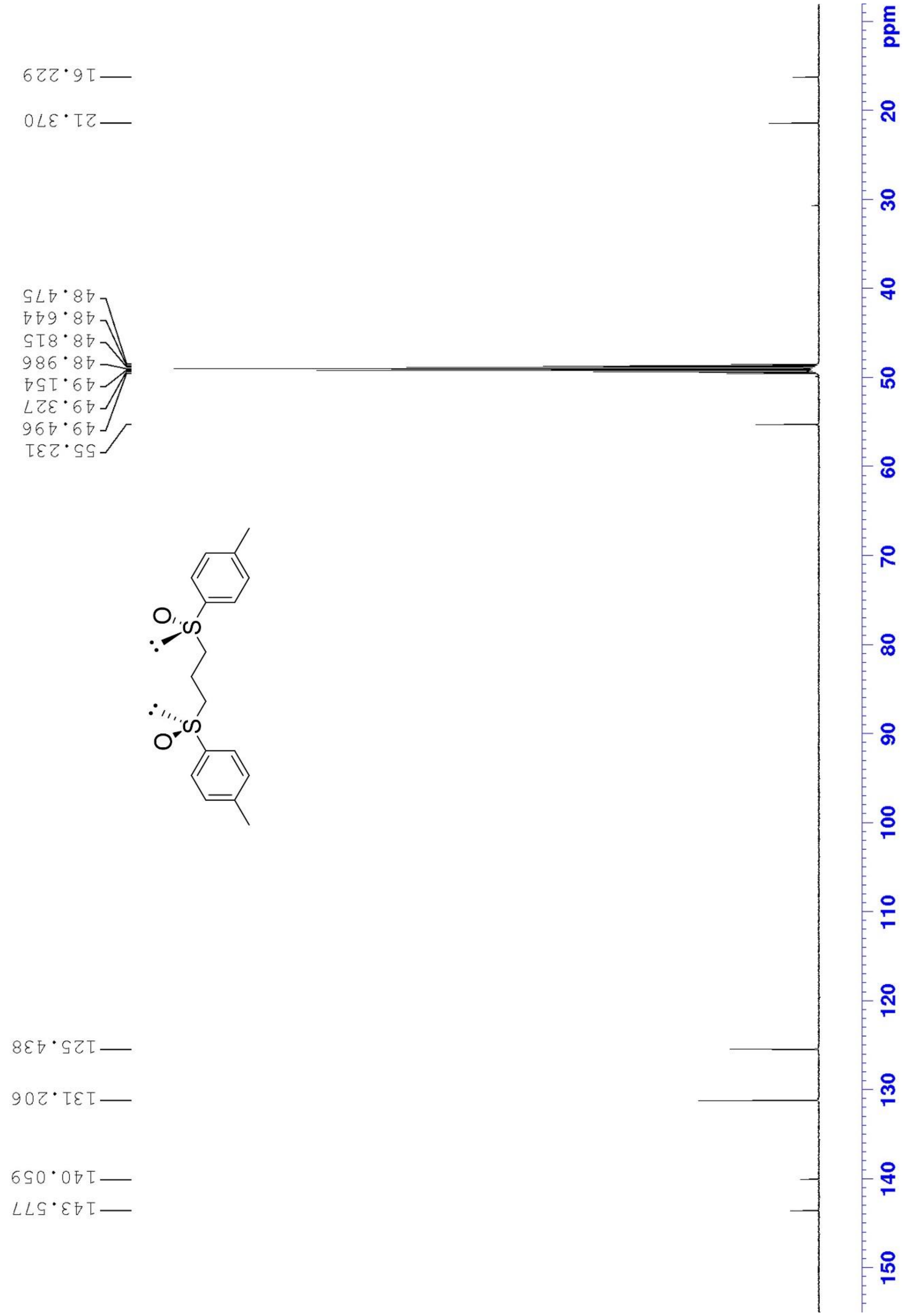

(S,S)-1,3-Bis(benzylsulfinyl)propane, **12(*S,S*)**

^{1}H NMR (500 MHz, $CDCl_3$)

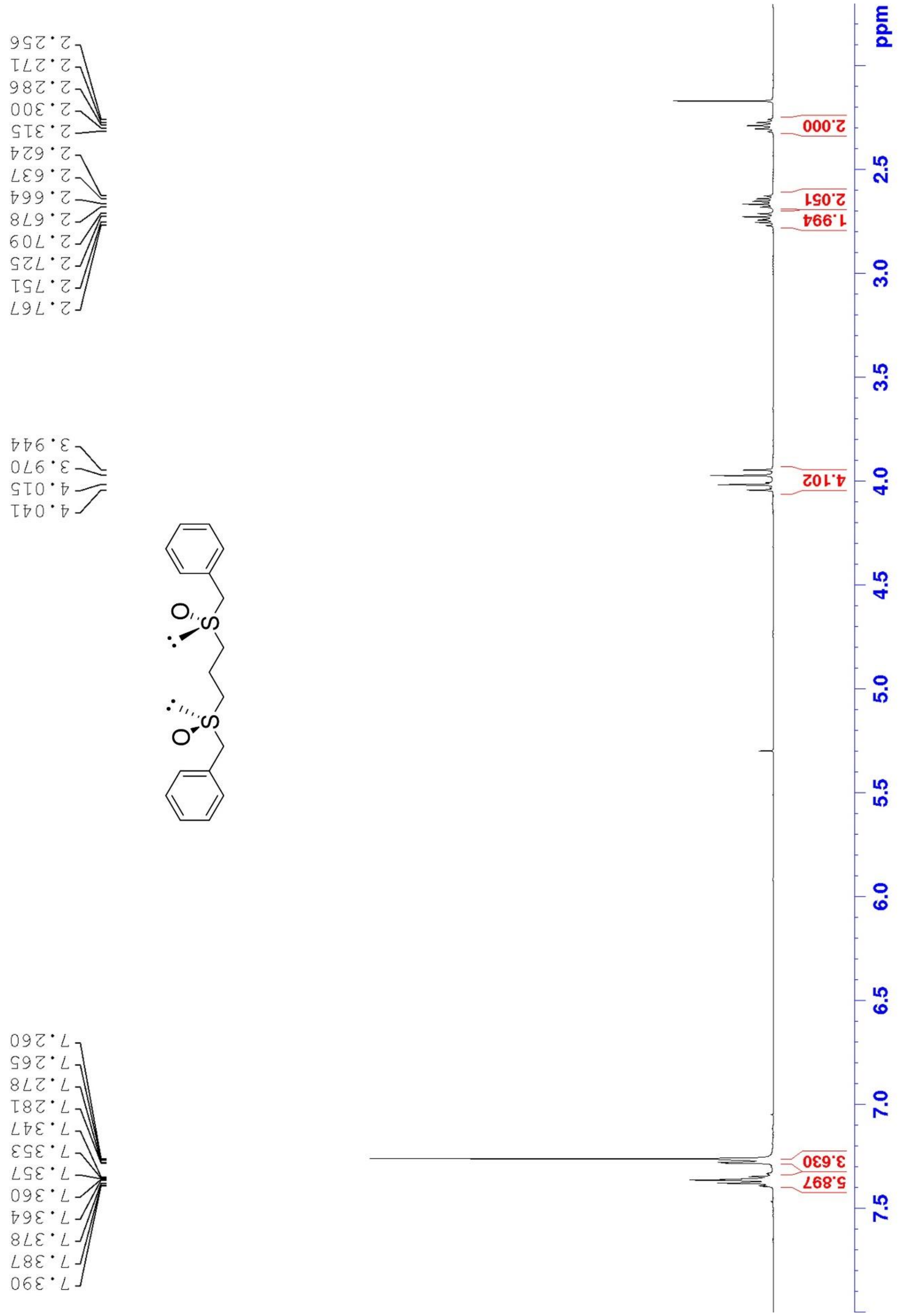

$^{13}C\{^{1}H\}$ NMR (125 MHz, $CDCl_3$)

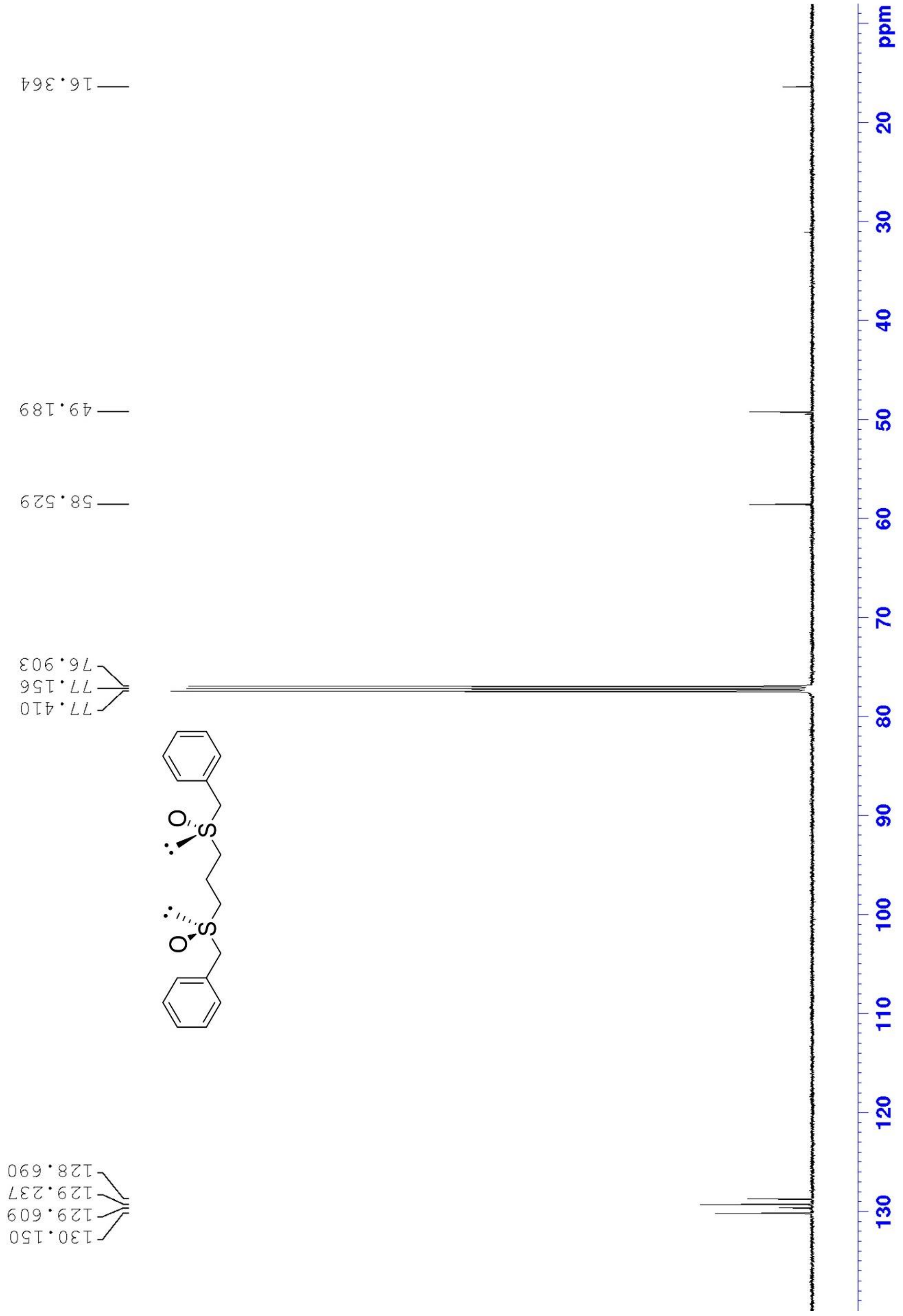

(S,S)-1,3-Bis[(pyridin-2-ylmethyl) sulfinyl]propane, **13(*S*,*S*)**

^{1}H NMR (500 MHz, $CDCl_3$)

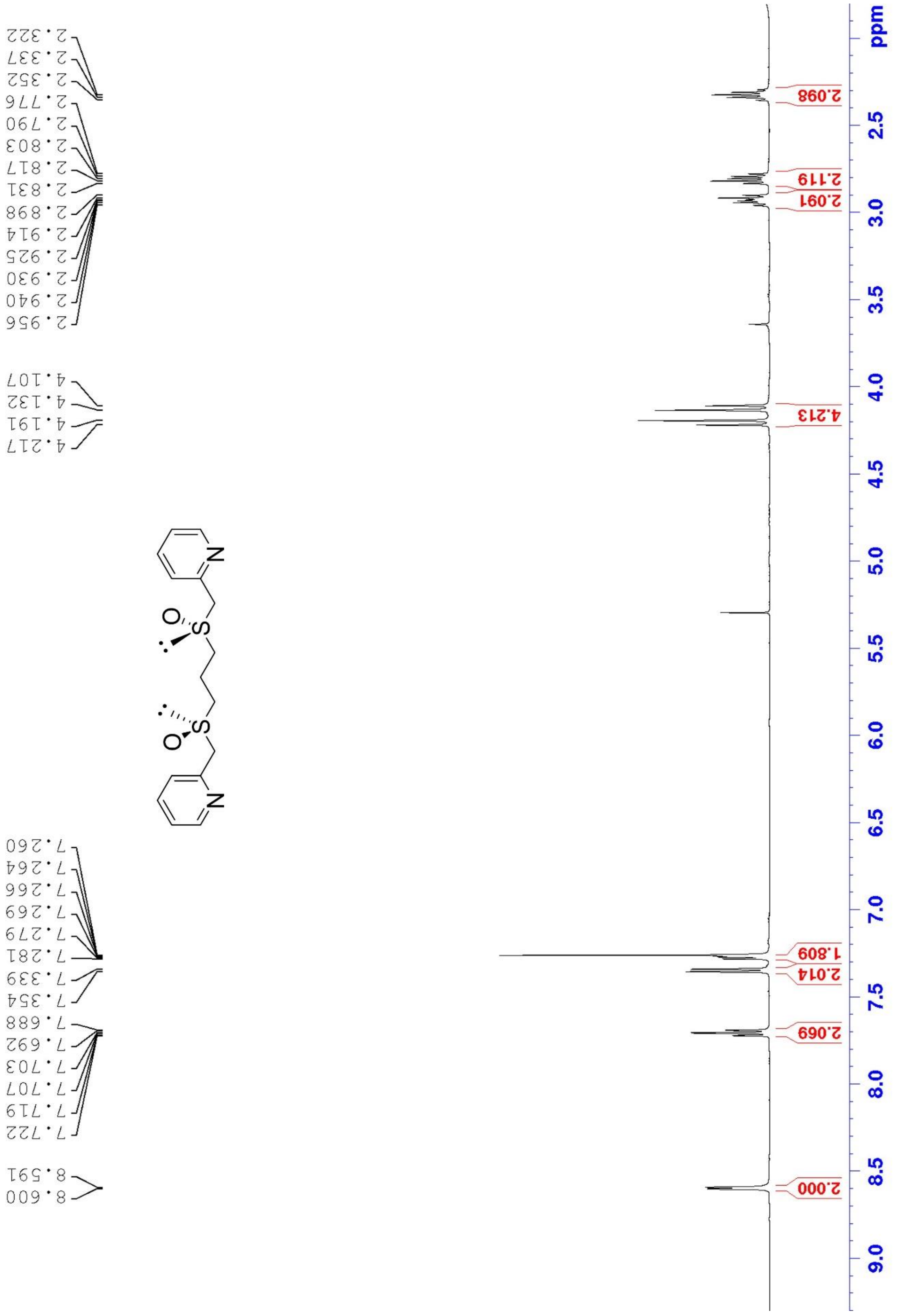

$^{13}C\{^{1}H\}$ NMR (125 MHz, $CDCl_3$)

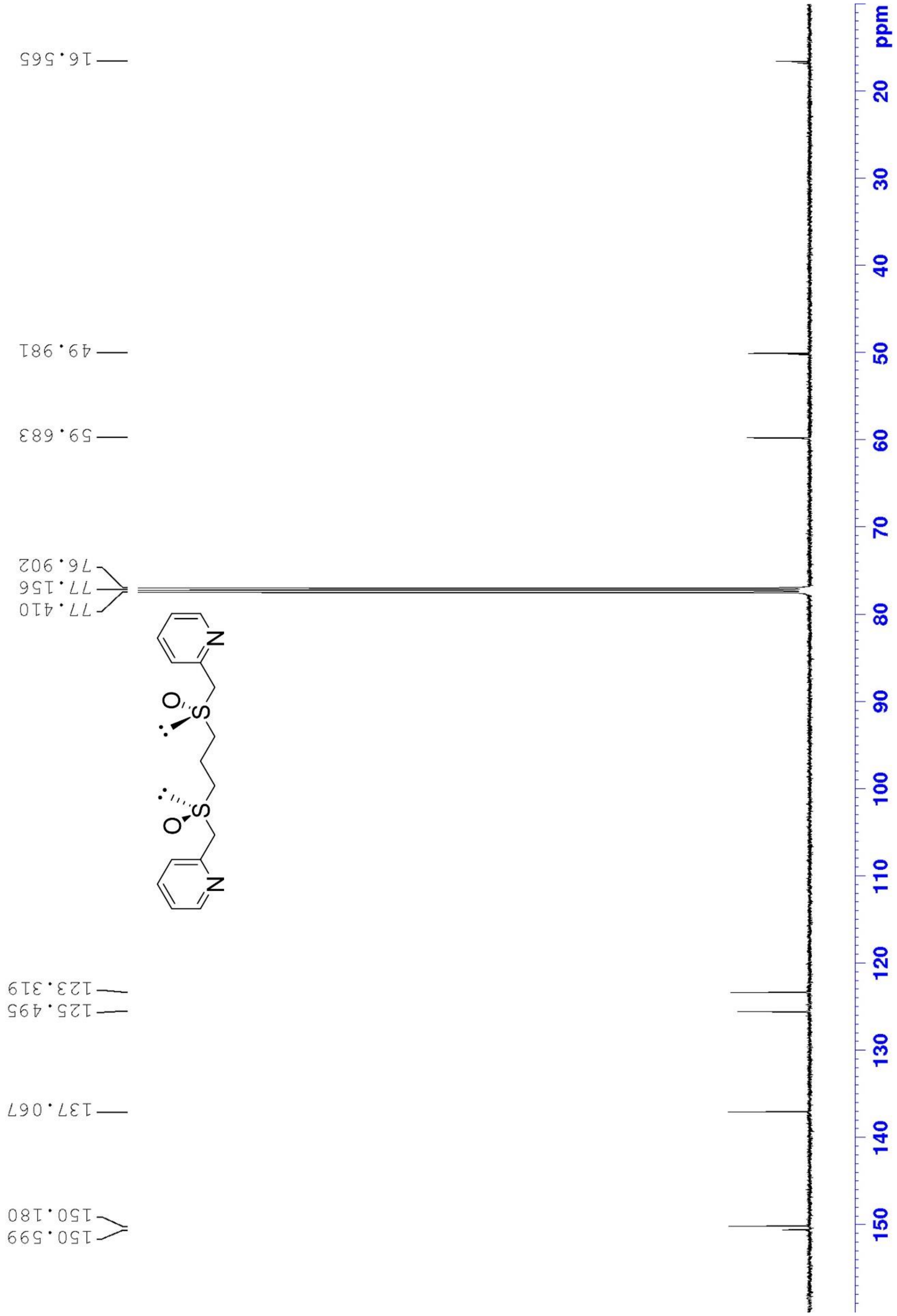

(R,R)-1,3-Bis(methylsulfinyl)propane, **14(*R,R*)**

¹H NMR (500 MHz, MeOD)

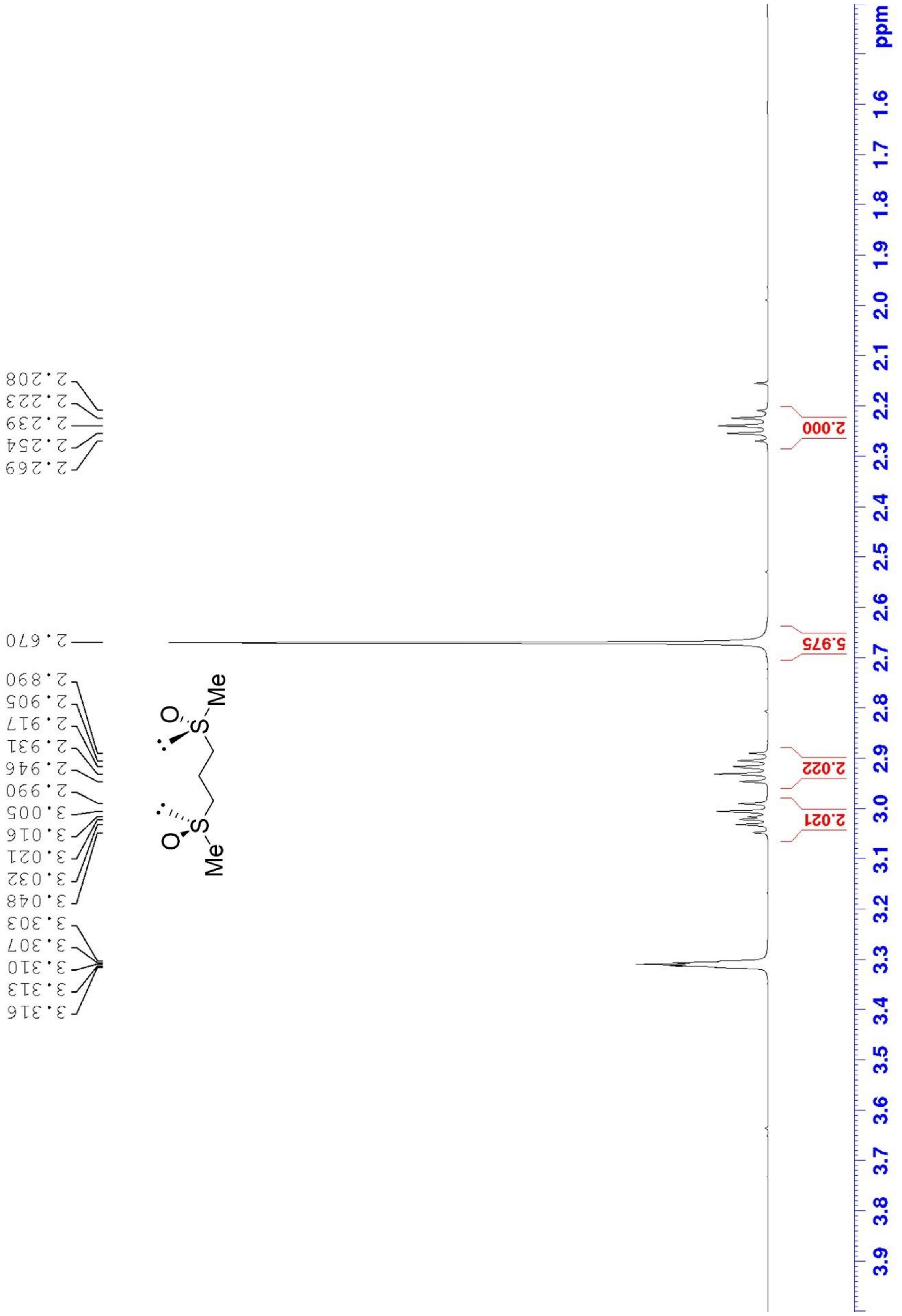

$^{13}C\{^{1}H\}$ NMR (125 MHz, MeOD)

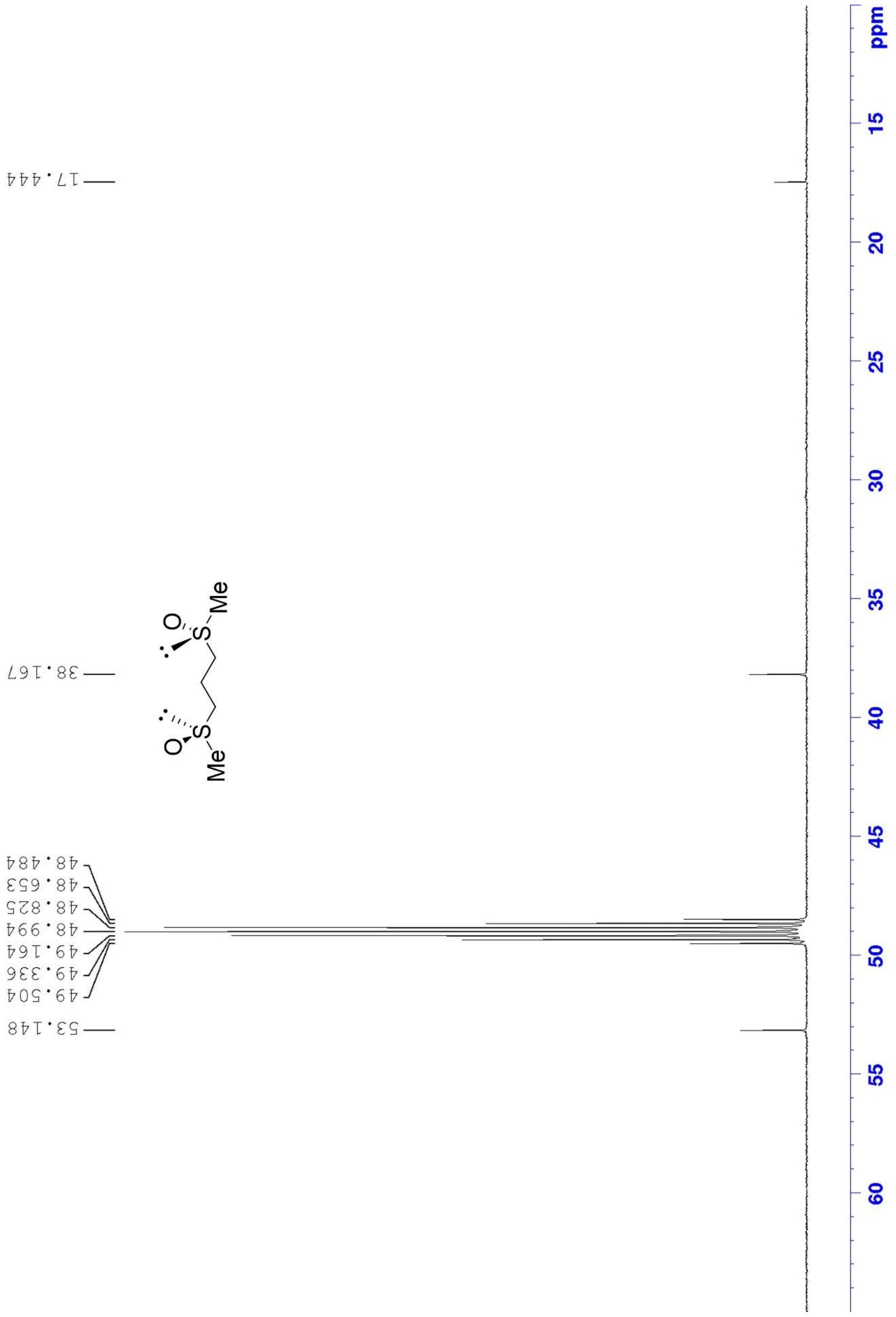

(S,S)-1,3-Bis(methylsulfinyl)propane, **14(*S,S*)**

^{1}H NMR (500 MHz, MeOD)

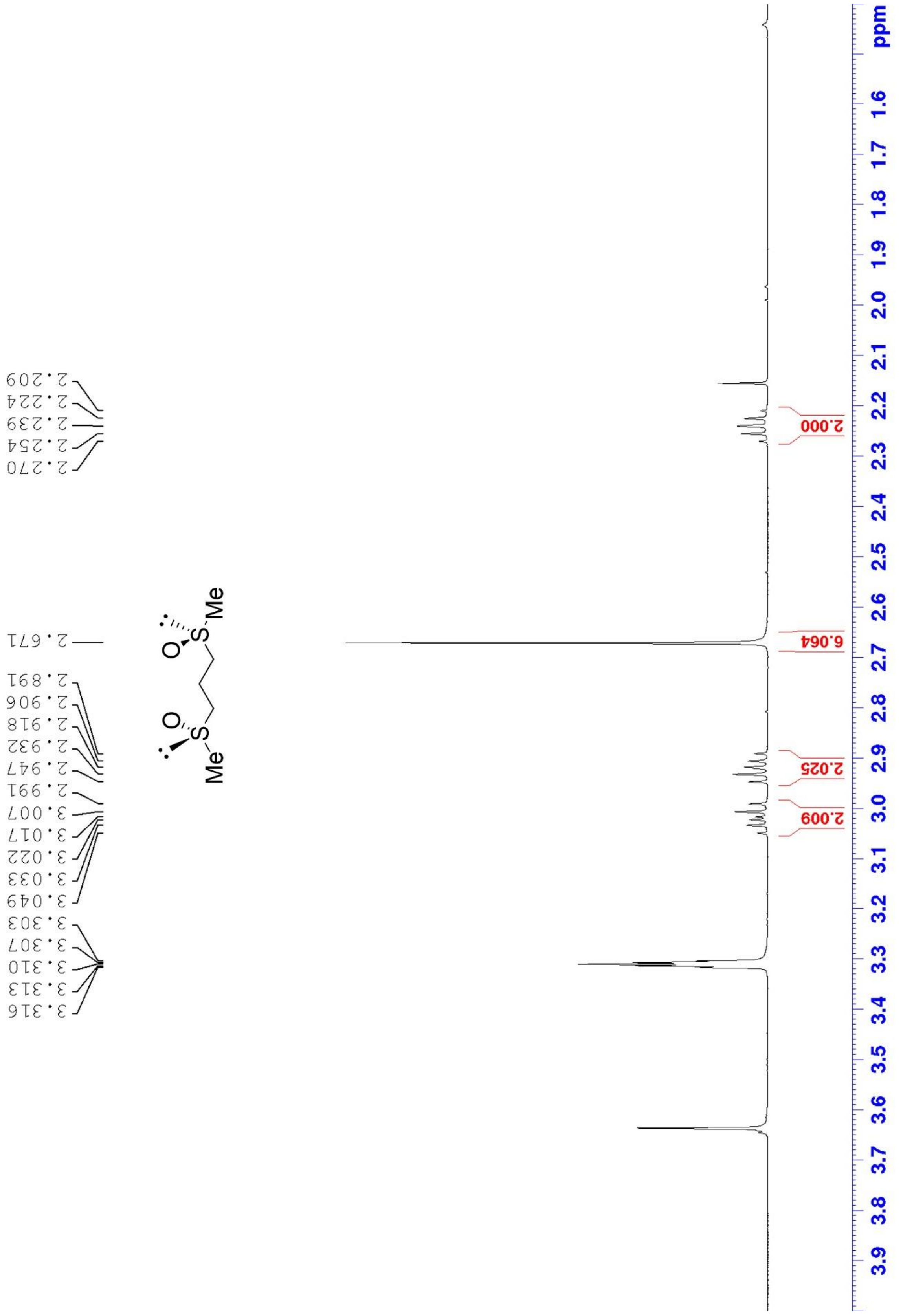

$^{13}C\{^{1}H\}$ NMR (125 MHz, MeOD)

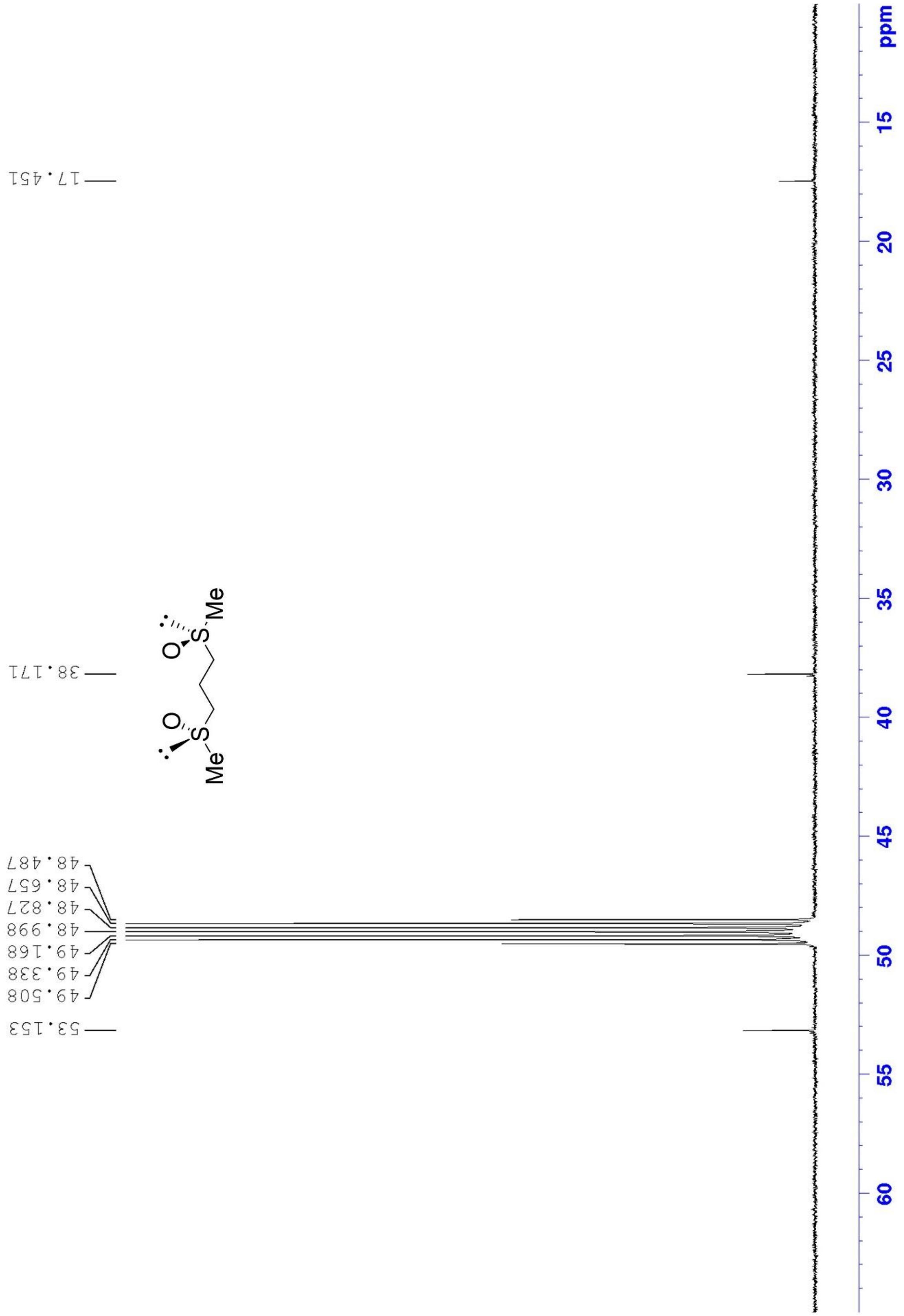

(R,R)-1,3-Bis(ethylsulfinyl)propane, **15(*R*,*R*)**

^{1}H NMR (500 MHz, MeOD)

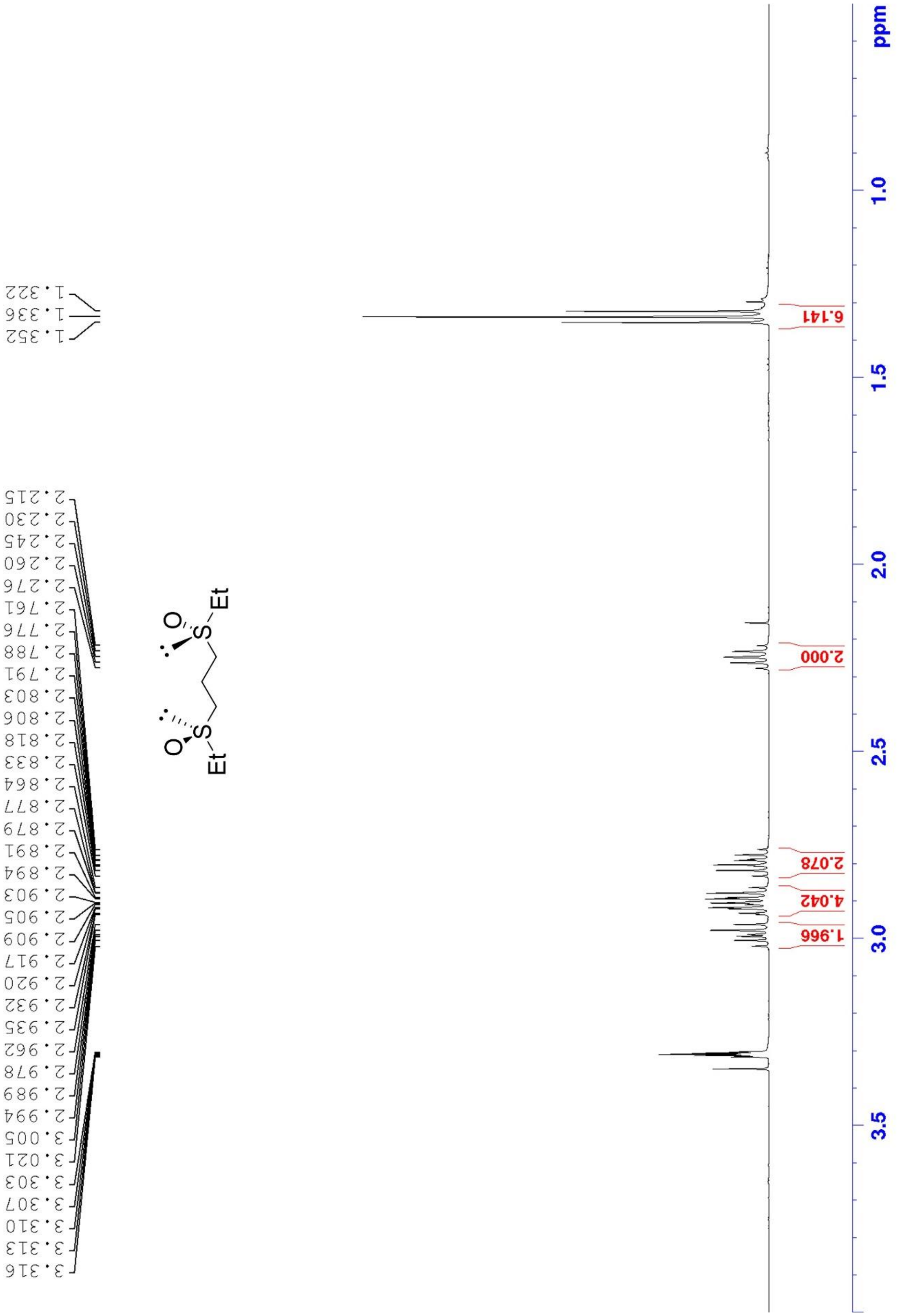

$^{13}C\{^1H\}$ NMR (125 MHz, MeOD)

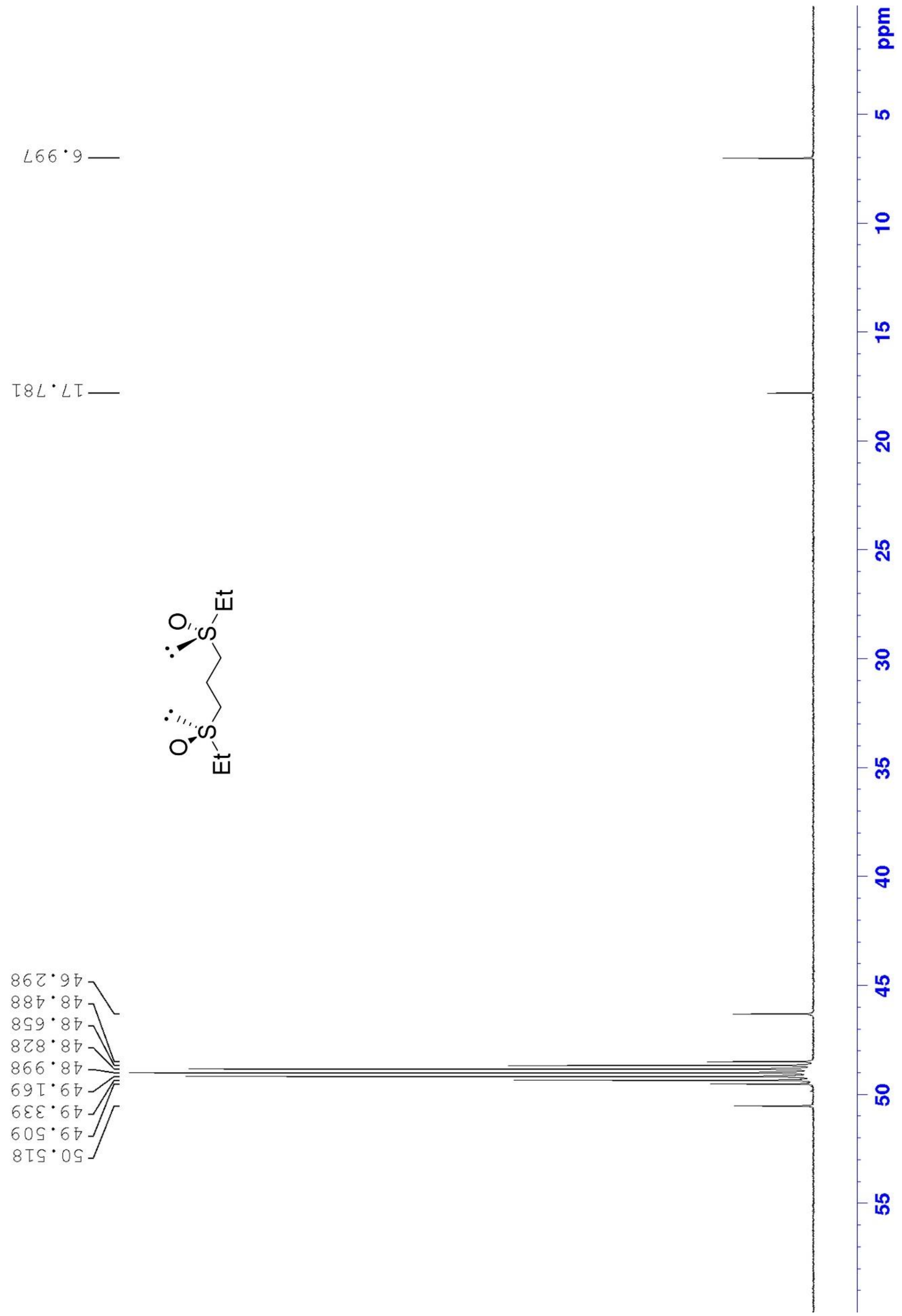

(S,S)-1,3-Bis(ethylsulfinyl)propane, **15(*S,S*)**

^{1}H NMR (500 MHz, MeOD)

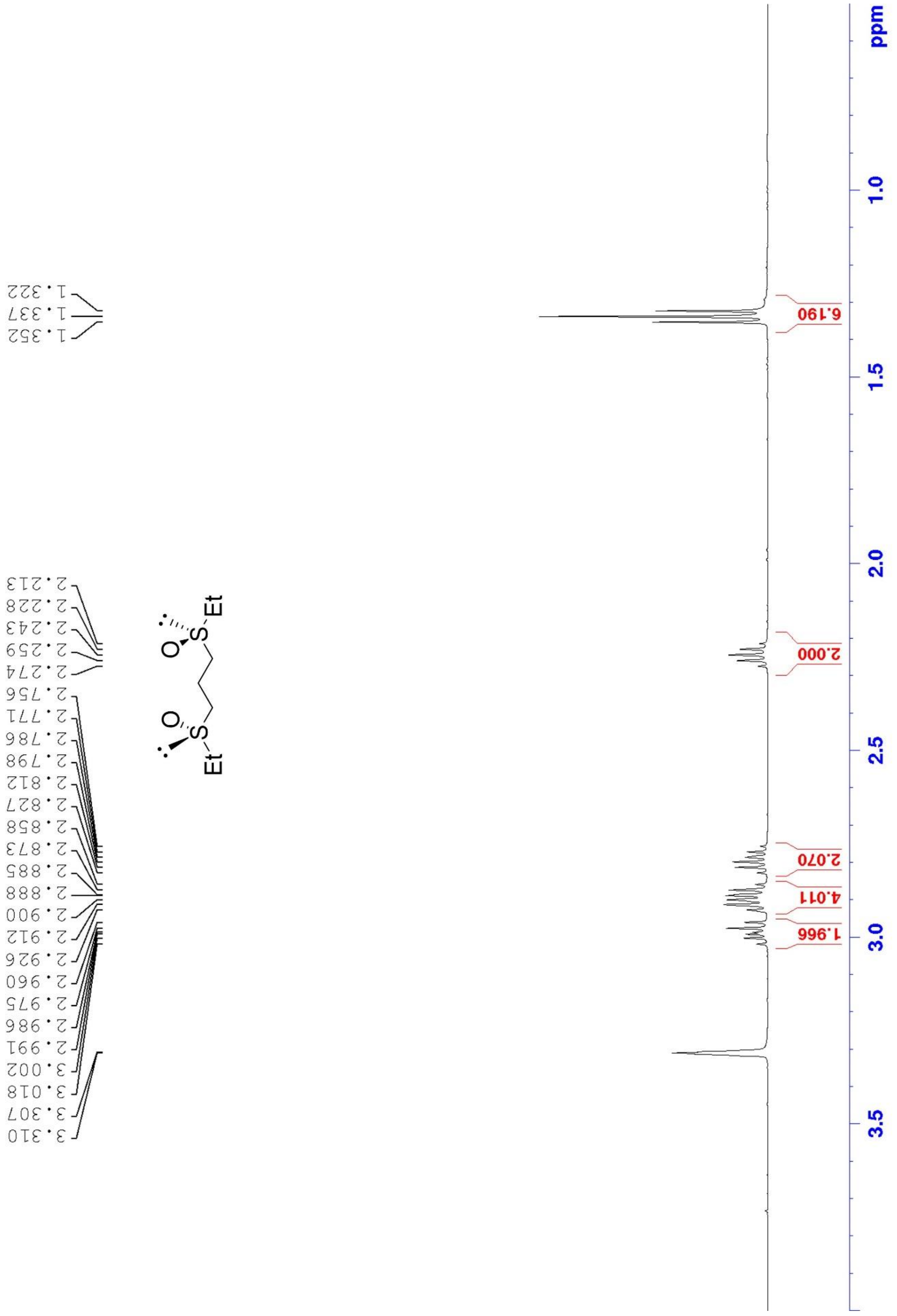

$^{13}C\{^{1}H\}$ NMR (125 MHz, MeOD)

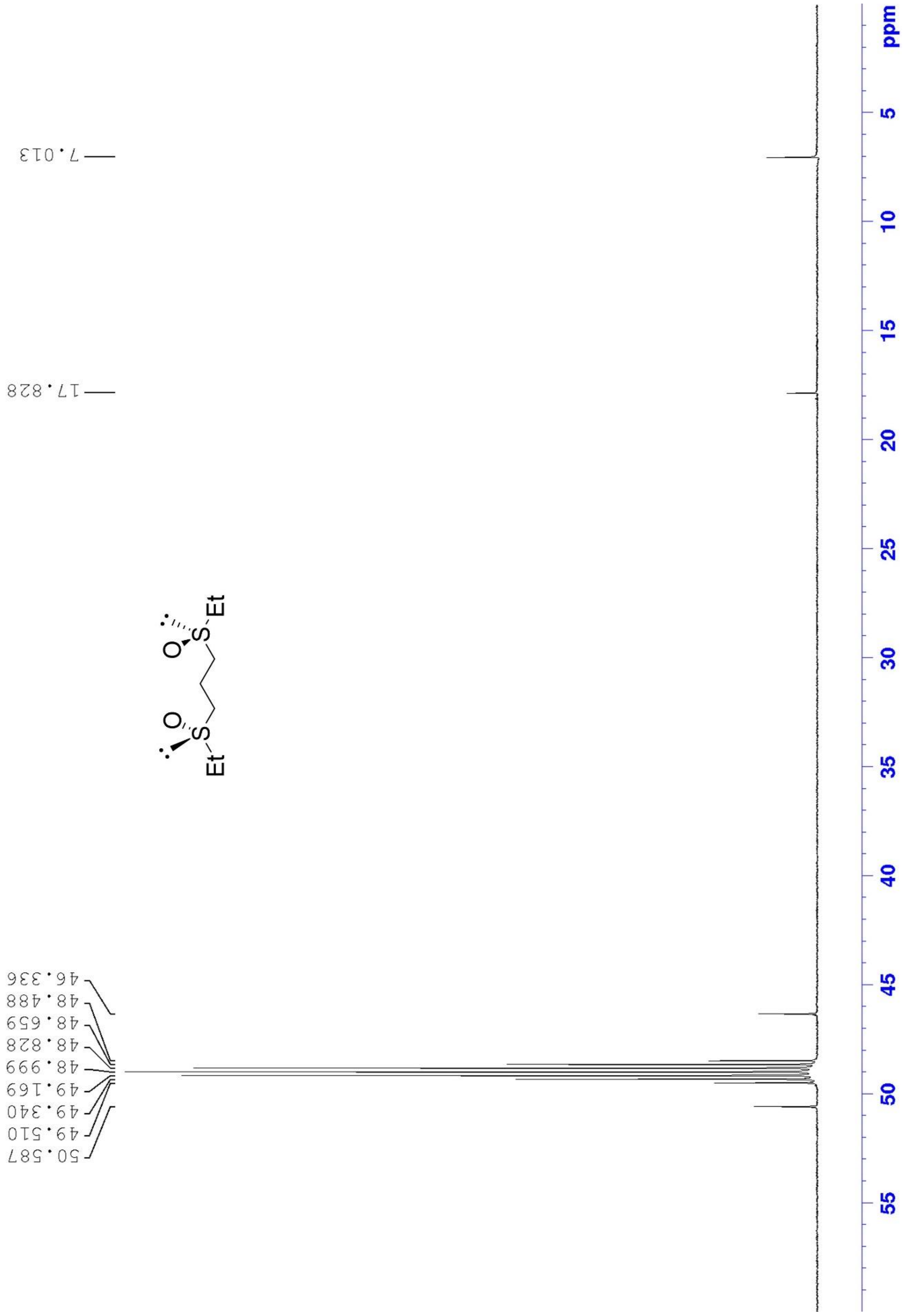

(R,R)-1,3-Bis(propylsulfinyl)propane, **16(*R,R*)**

^{1}H NMR (500 MHz, MeOD)

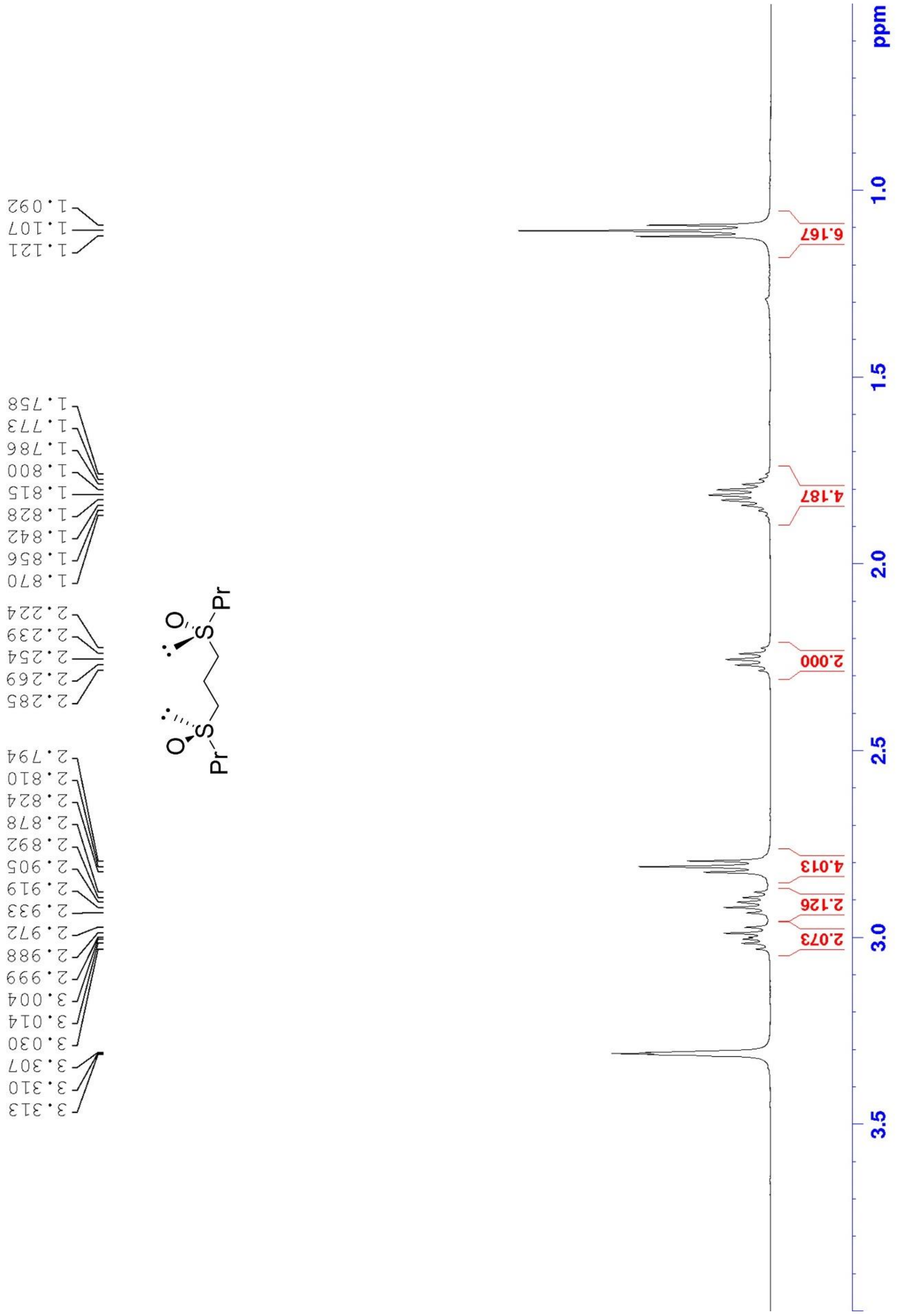

$^{13}C\{^1H\}$ NMR (125 MHz, MeOD)

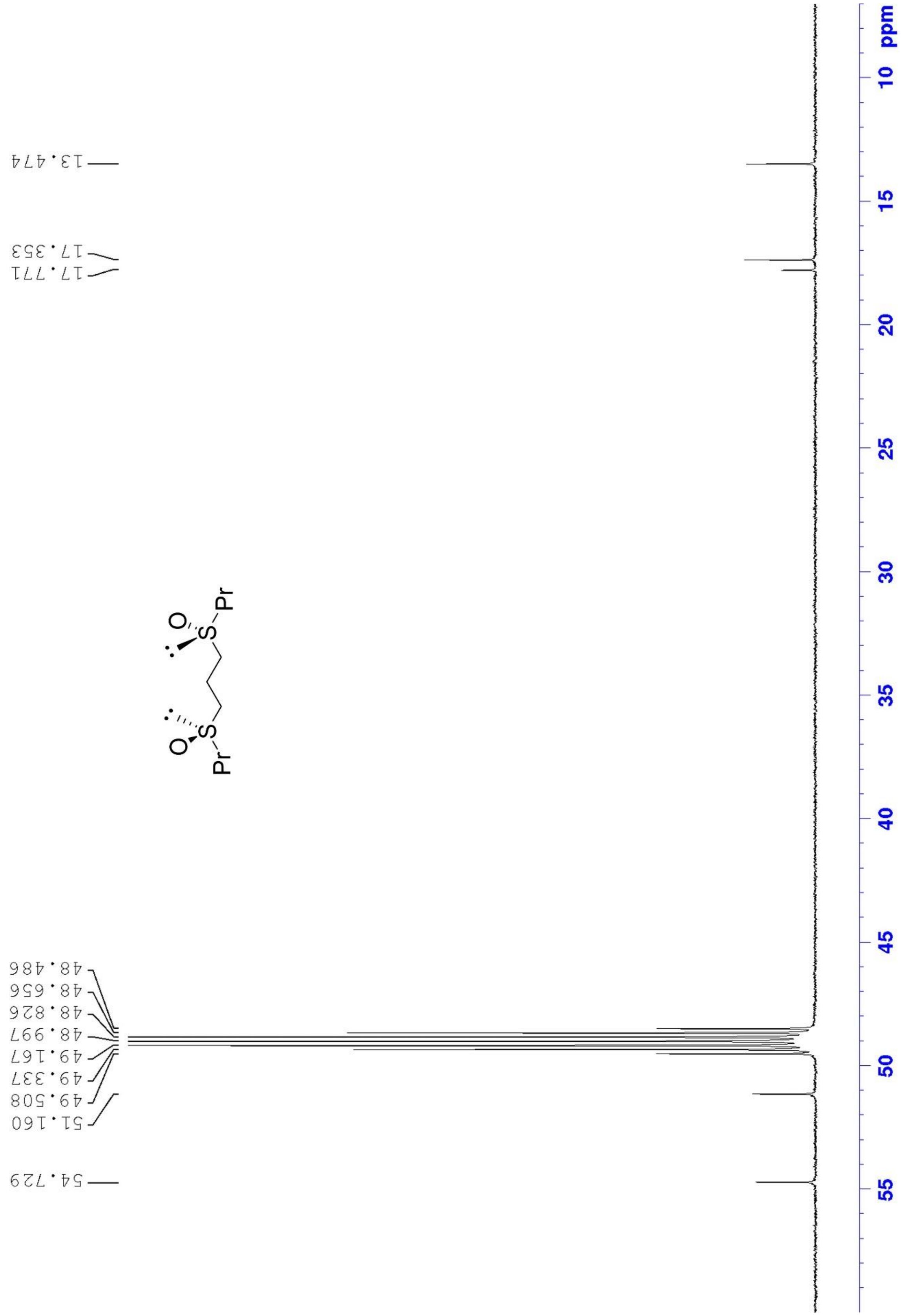

(S,S)-1,3-Bis(isopropylsulfinyl)propane, **17(*S*,*S*)**

^{1}H NMR (500 MHz, MeOD)

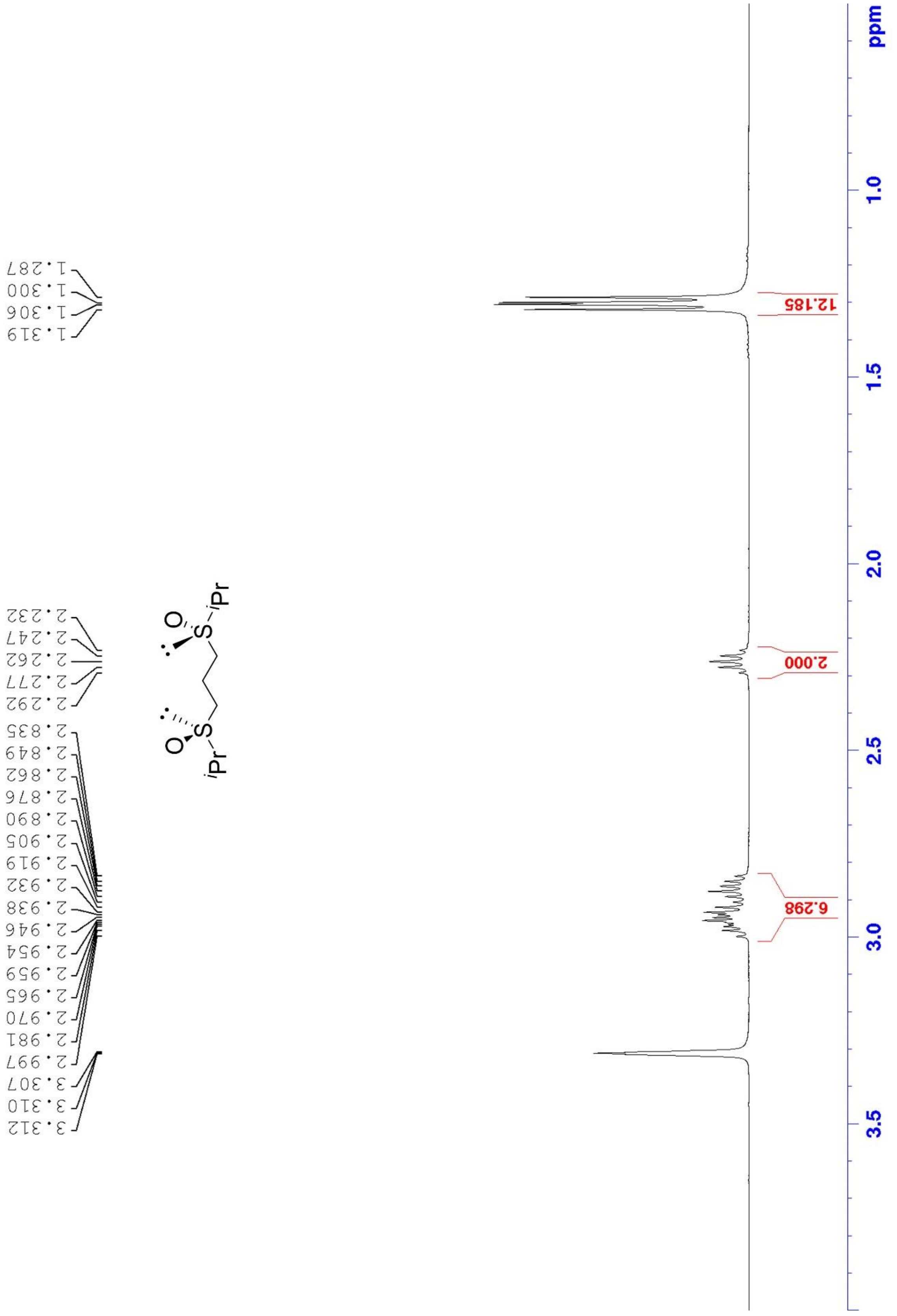

$^{13}C\{^{1}H\}$ NMR (125 MHz, MeOD)

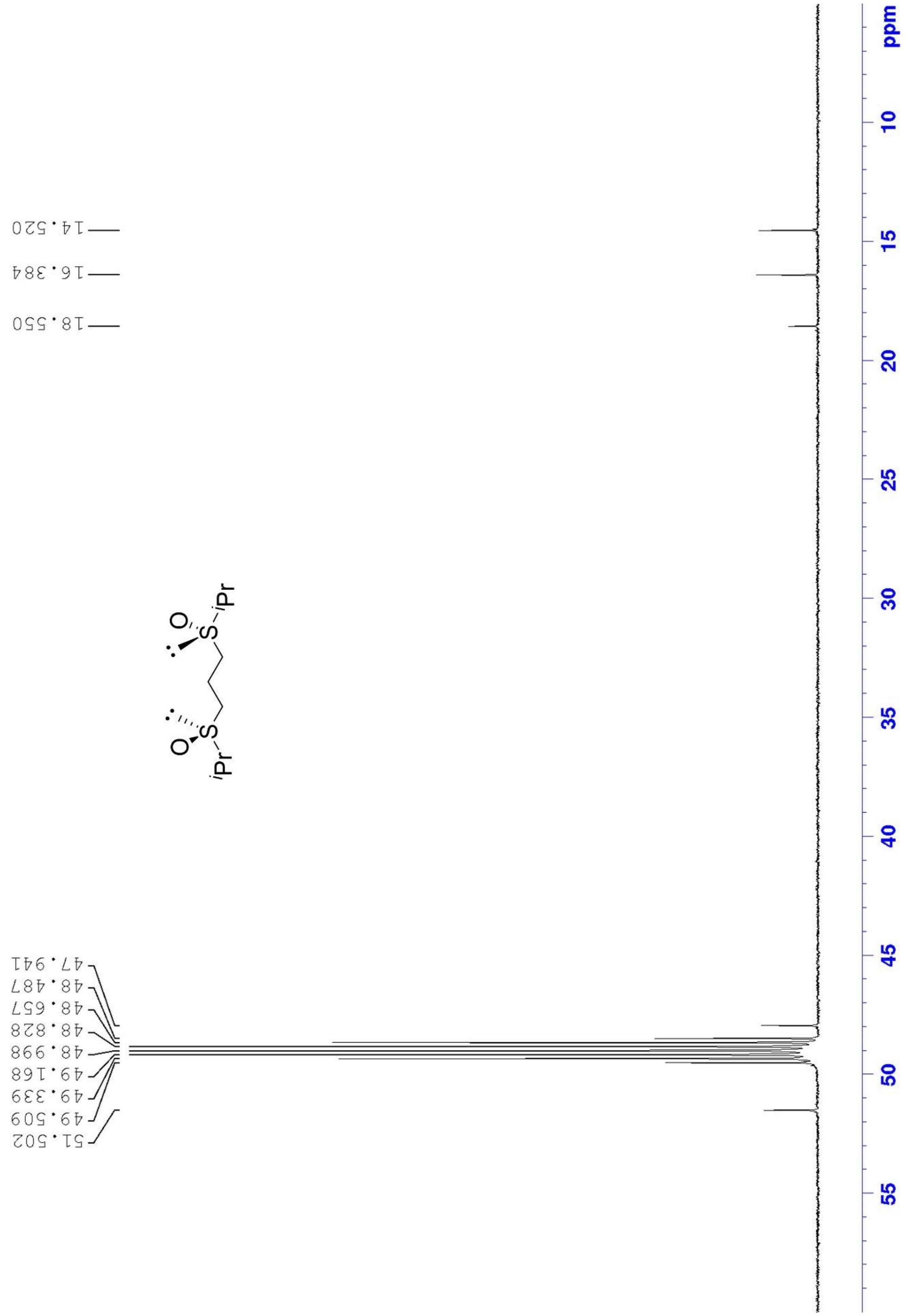

(S,S)-1,3-Bis(tert-butylsulfinyl)propane, **18(*S*,*S*)**

^{1}H NMR (500 MHz, MeOD)

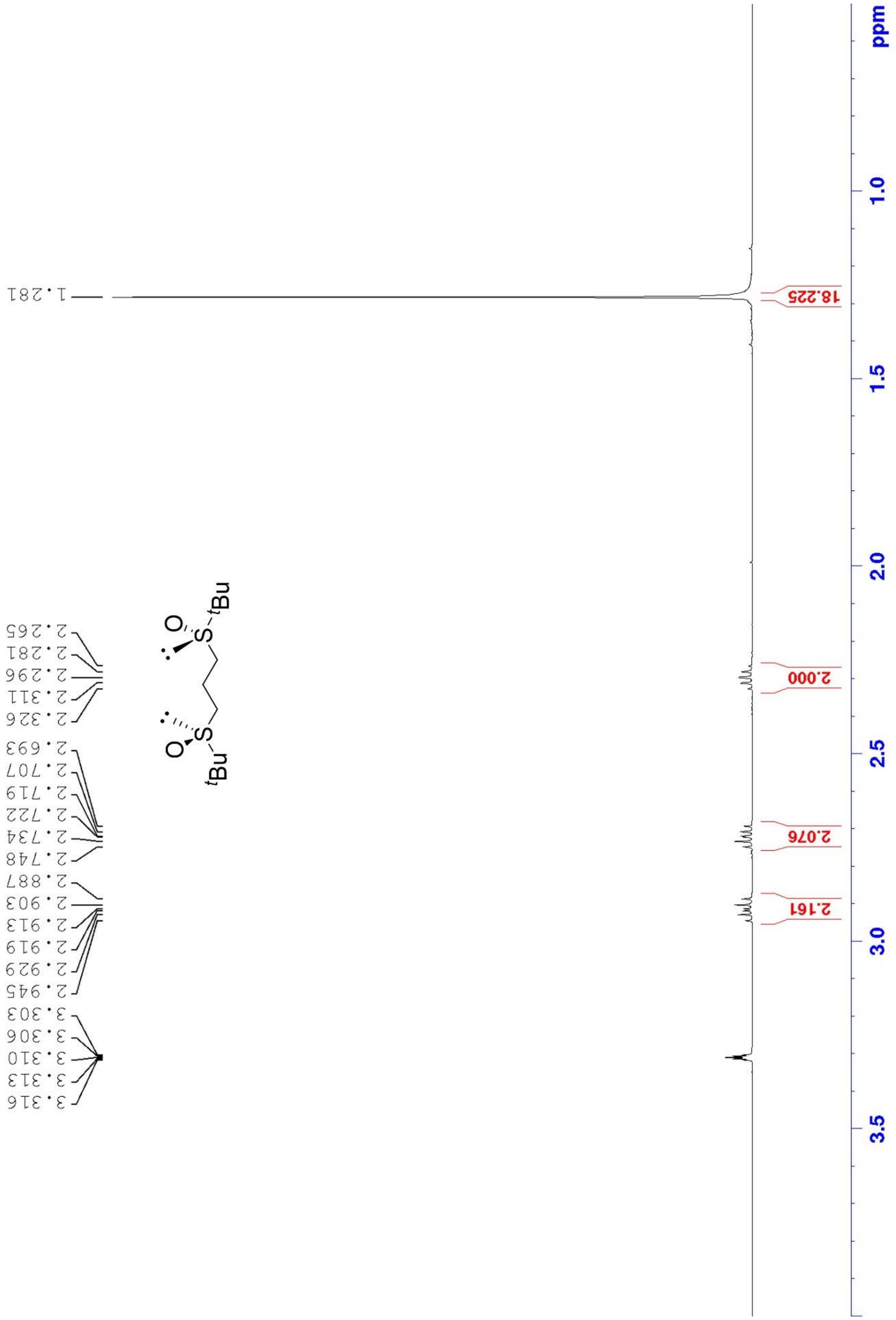

$^{13}C\{^1H\}$ NMR (125 MHz, MeOD)

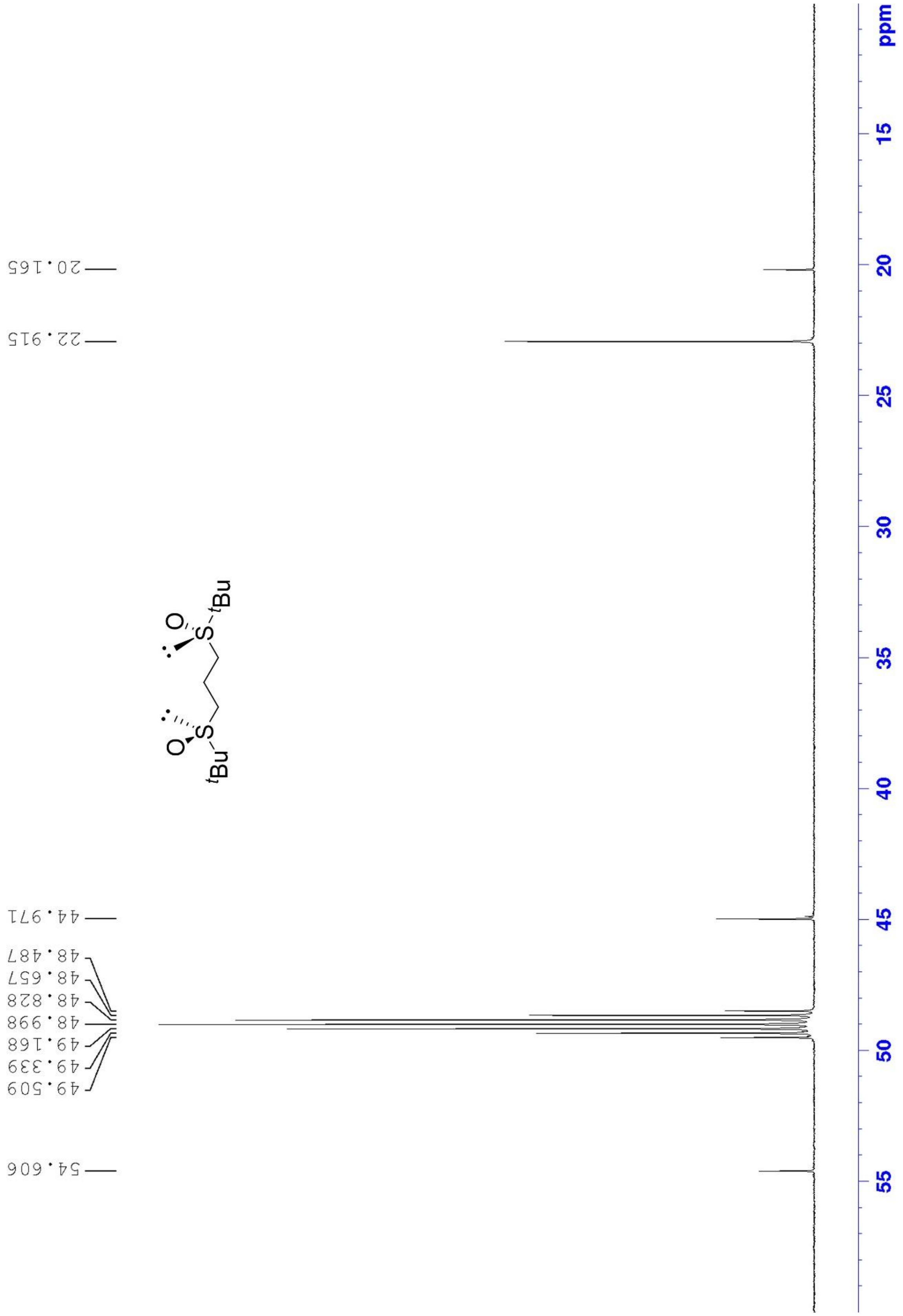

(S,S)/(R,R)/(S,R)-1,3-Bis(phenylsulfinyl)propane, **8(*rac+meso*)**

^{1}H NMR (500 MHz, $CDCl_3$)

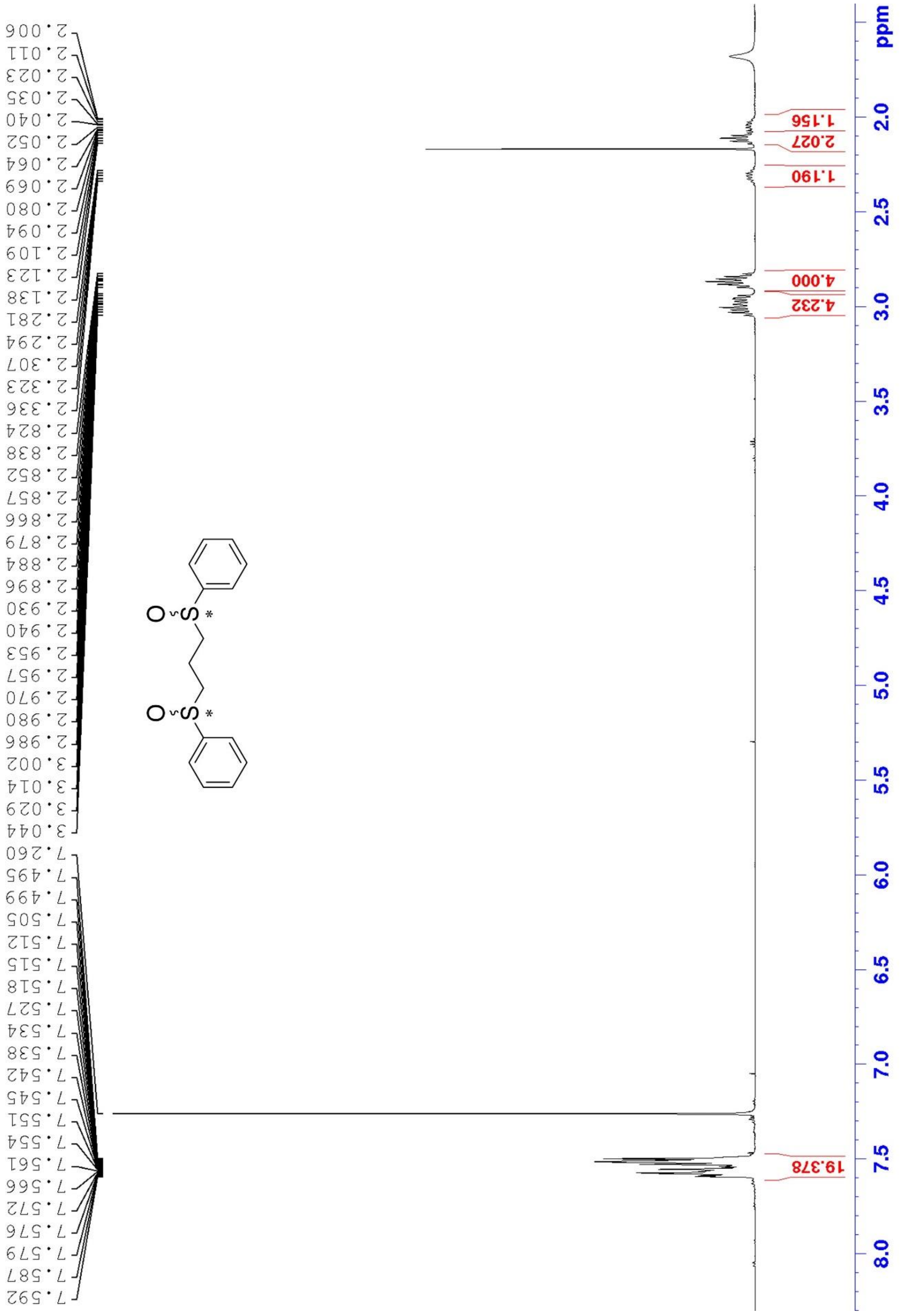

$^{13}C\{^{1}H\}$ NMR (125 MHz, $CDCl_3$)

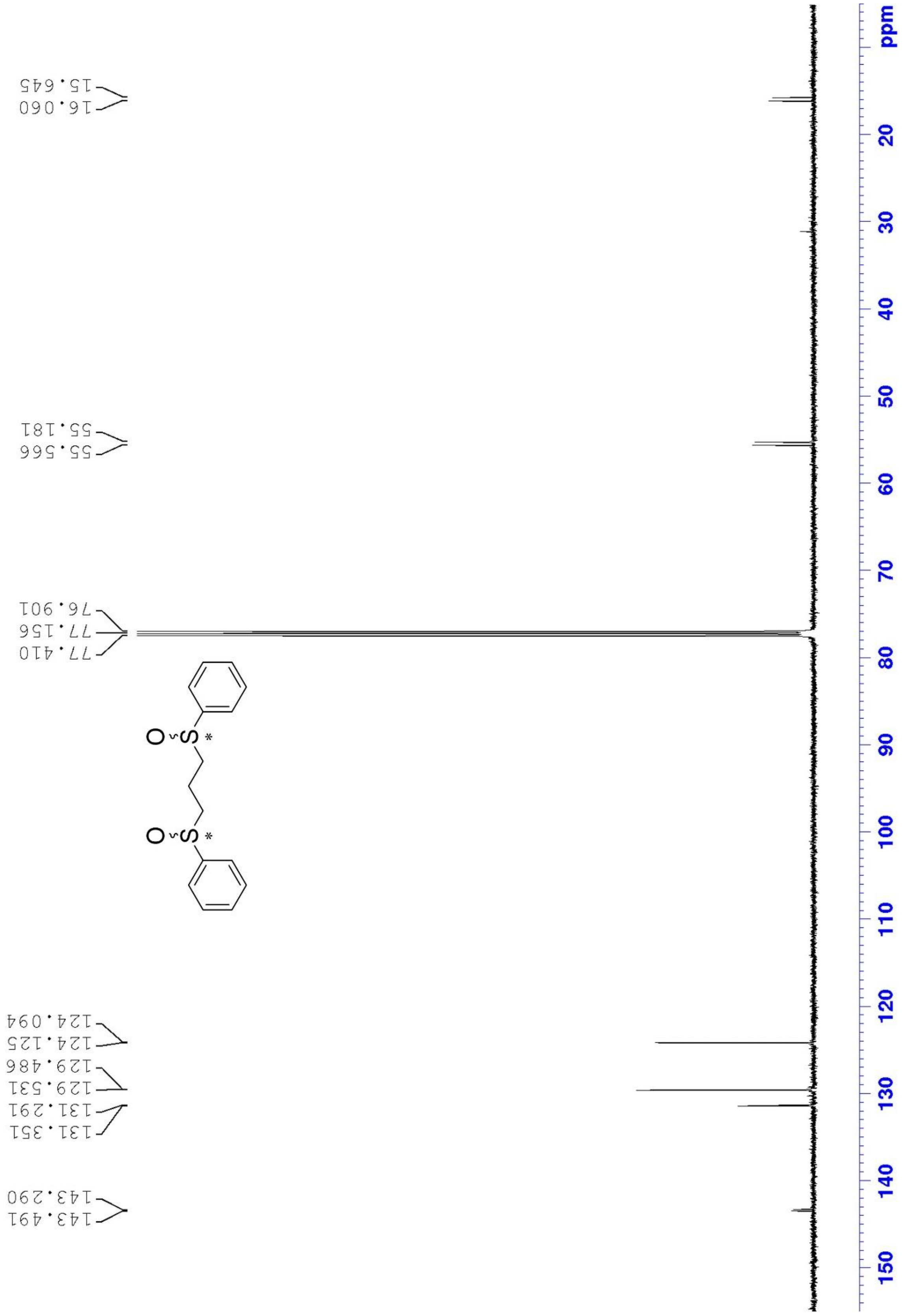

(S,S)/(R,R)/(S,R)-Bis(1-naphthylsulfinyl)propane, **9(*rac+meso*)**

^{1}H NMR (500 MHz, $CDCl_3$)

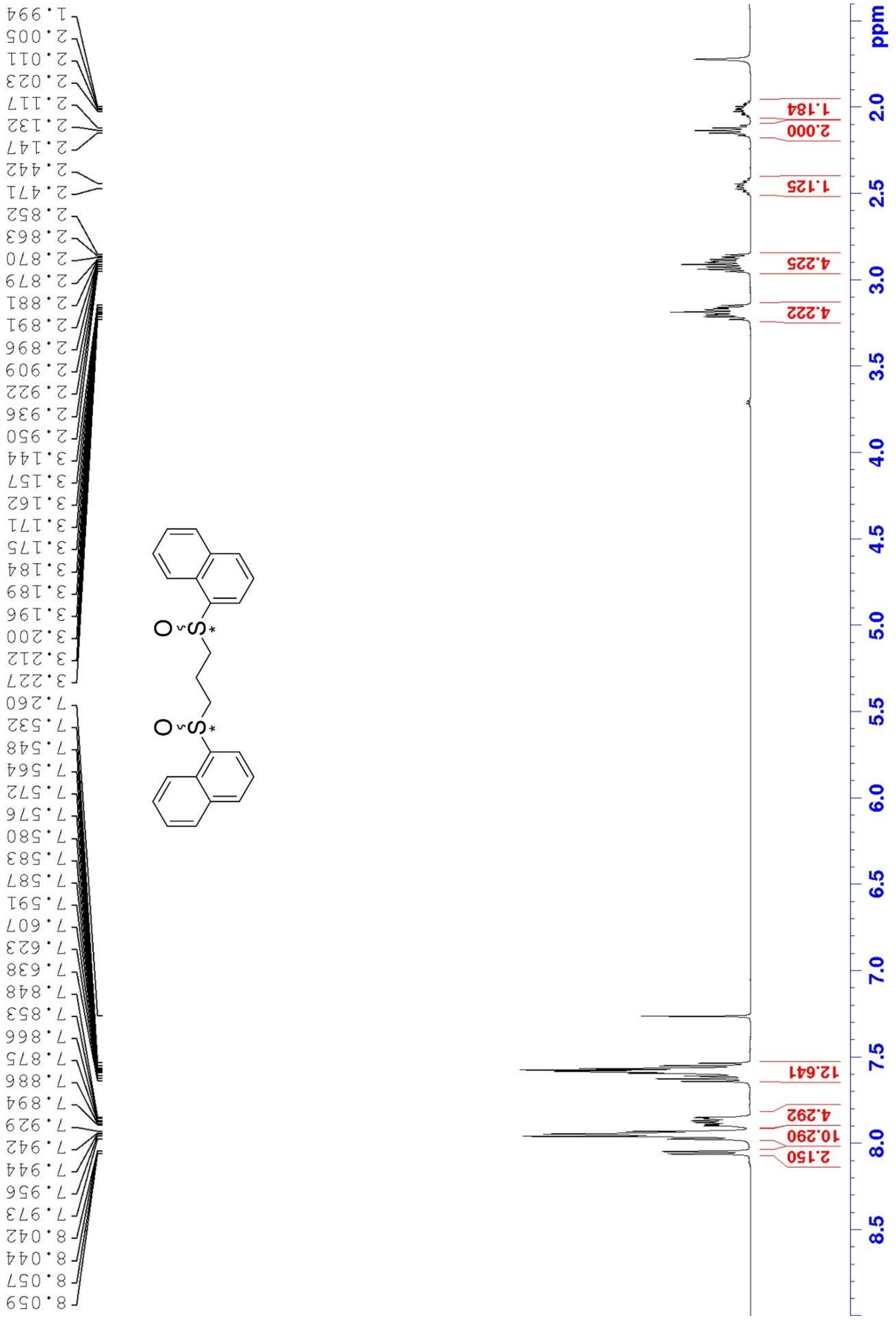

$^{13}C\{^{1}H\}$ NMR (125 MHz, $CDCl_3$)

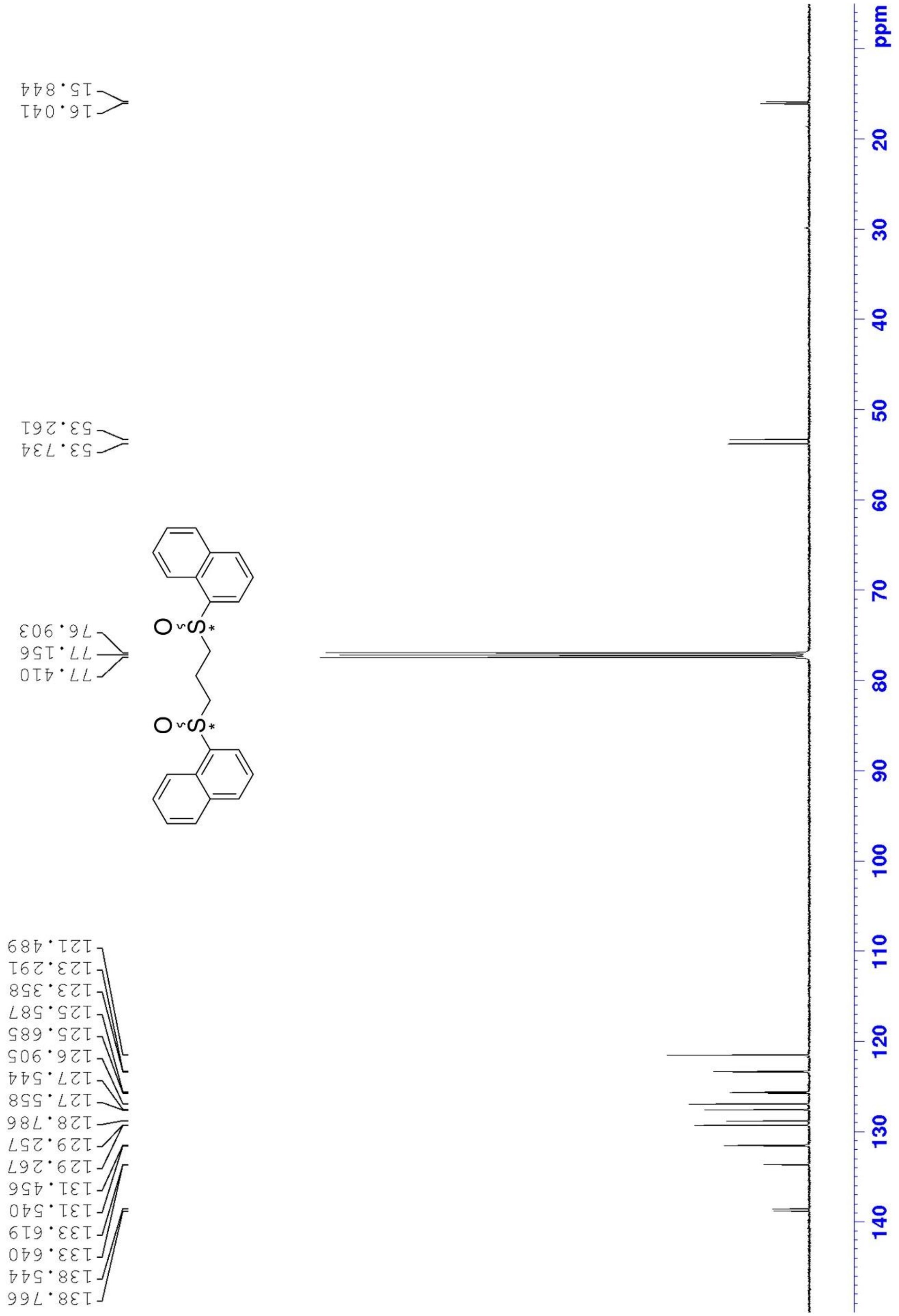

(S,S)/(R,R)/(S,R)-1,3-Bis[(2,6-dimethylphenyl)sulfinyl]propane, **10(*rac+meso*)**

^{1}H NMR (500 MHz, $CDCl_3$)

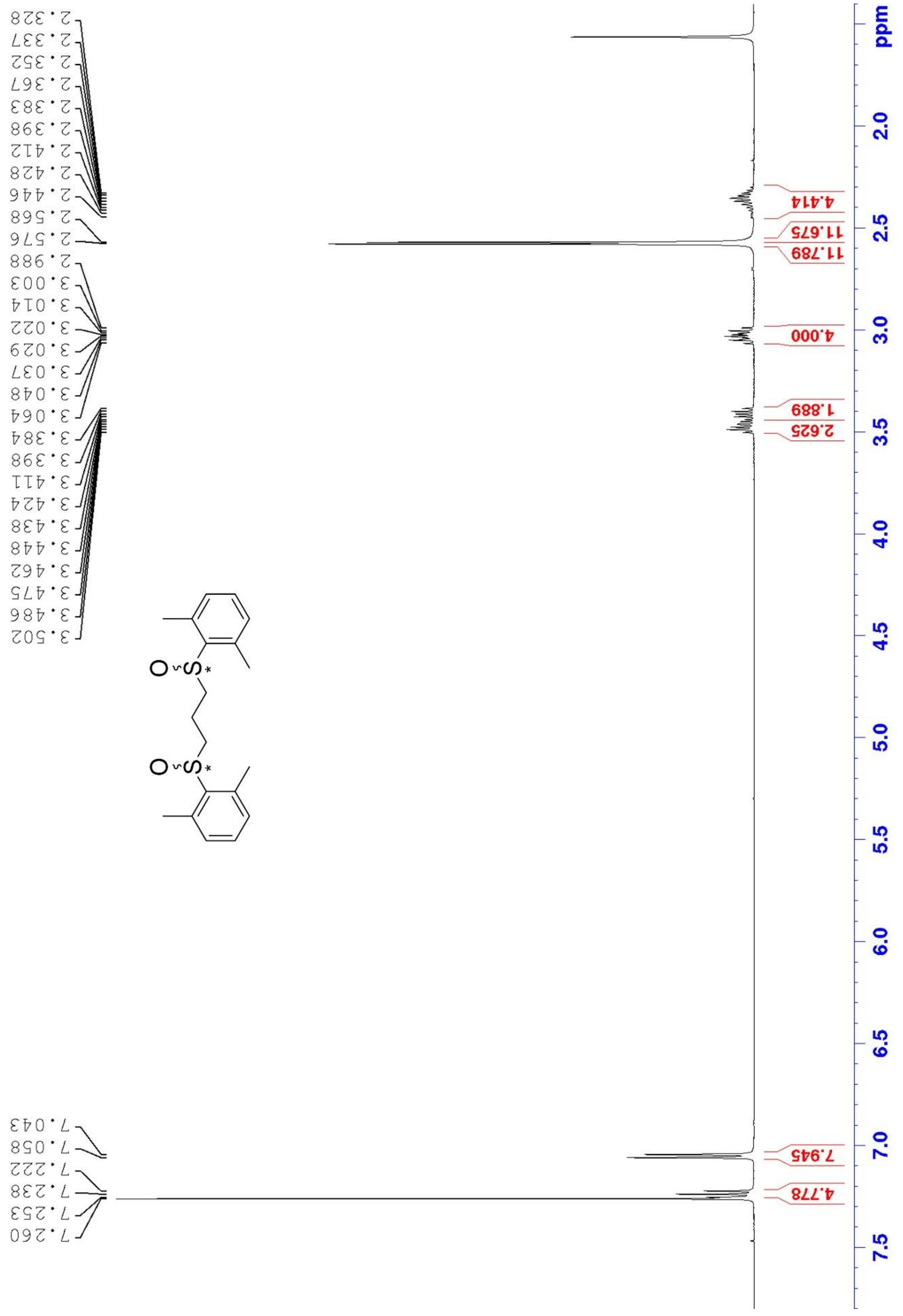

$^{13}C\{^{1}H\}$ NMR (125 MHz, $CDCl_3$)

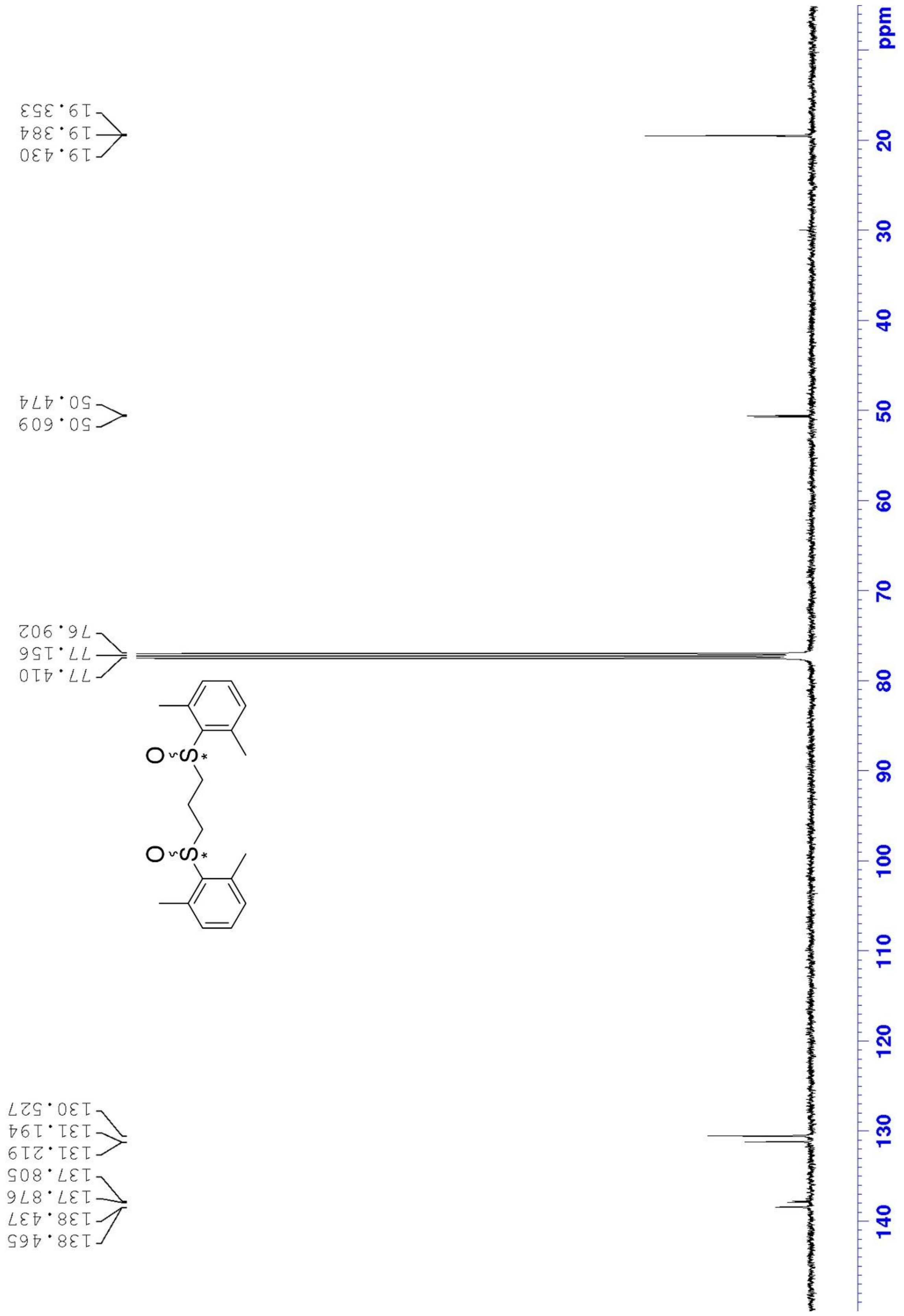

(S,S)/(R,R)/(S,R)-1,3-Bis(p-tolylsulfinyl)propane, **11(*rac+meso*)**

^{1}H NMR (500 MHz, MeOD)

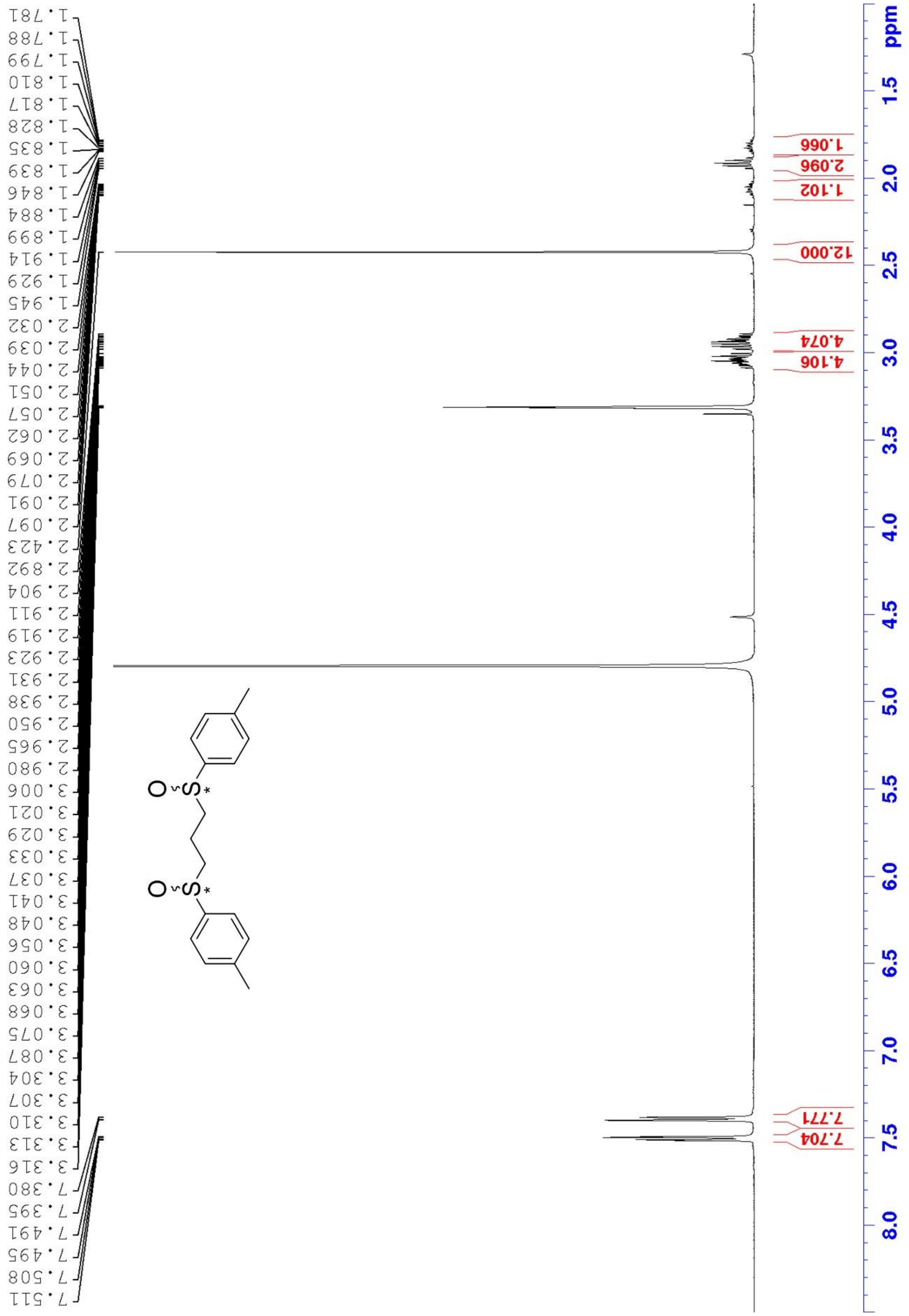

$^{13}C\{^{1}H\}$ NMR (125 MHz, MeOD)

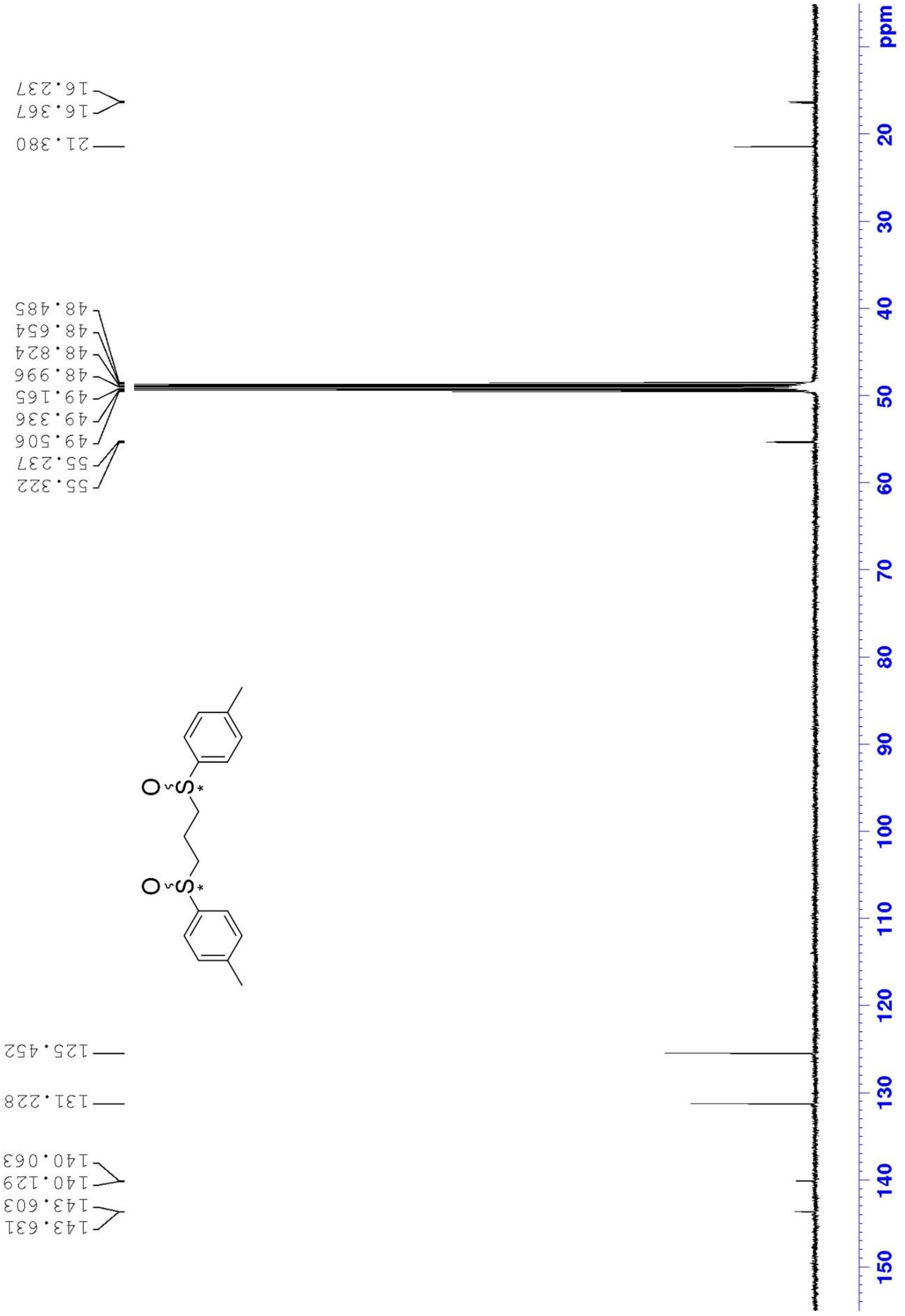

(S,S)/(R,R)/(S,R)-1,3-Bis(benzylsulfinyl)propane, **12(*rac*+*meso*)**

^{1}H NMR (500 MHz, $CDCl_3$)

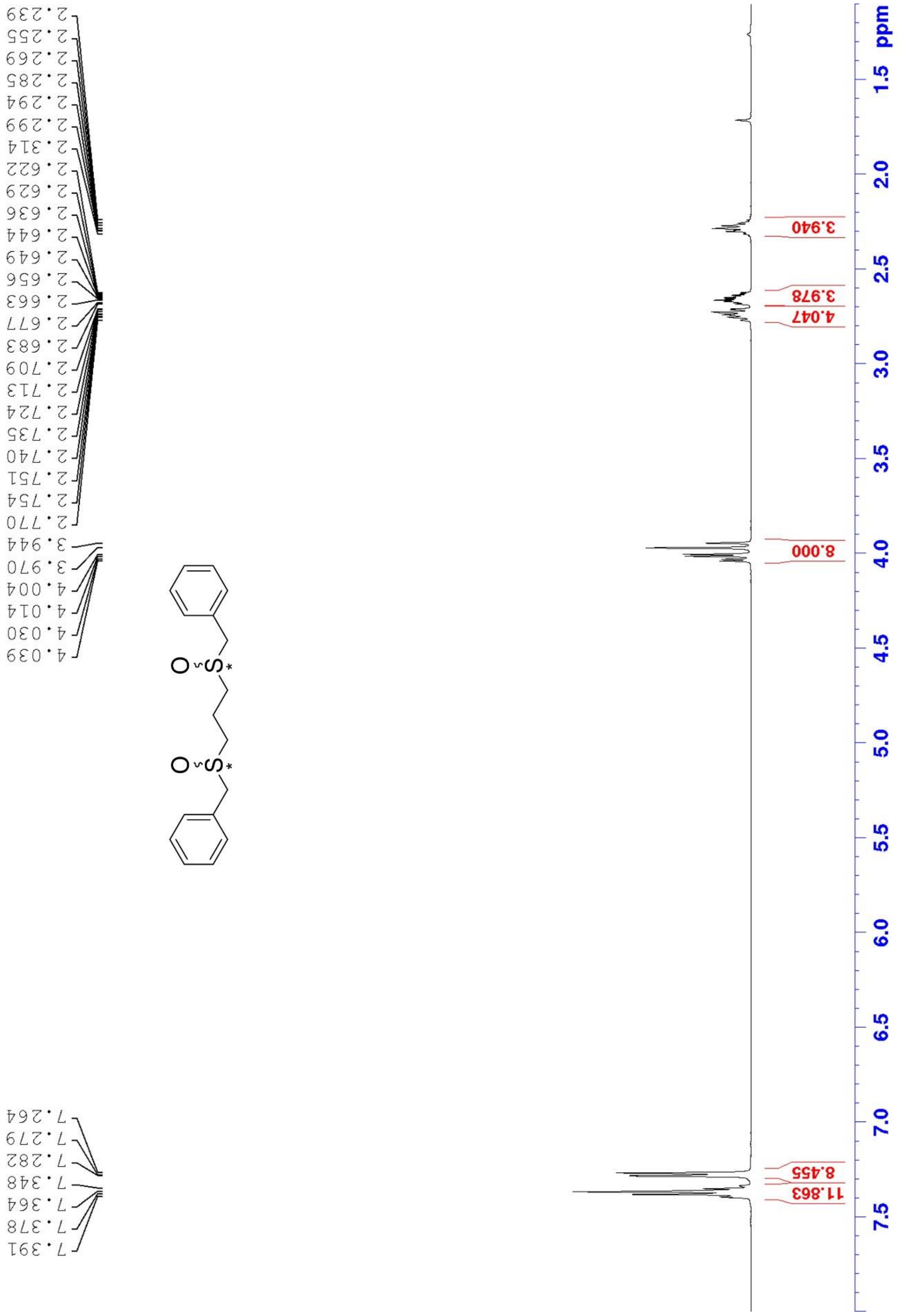

$^{13}C\{^{1}H\}$ NMR (125 MHz, $CDCl_3$)

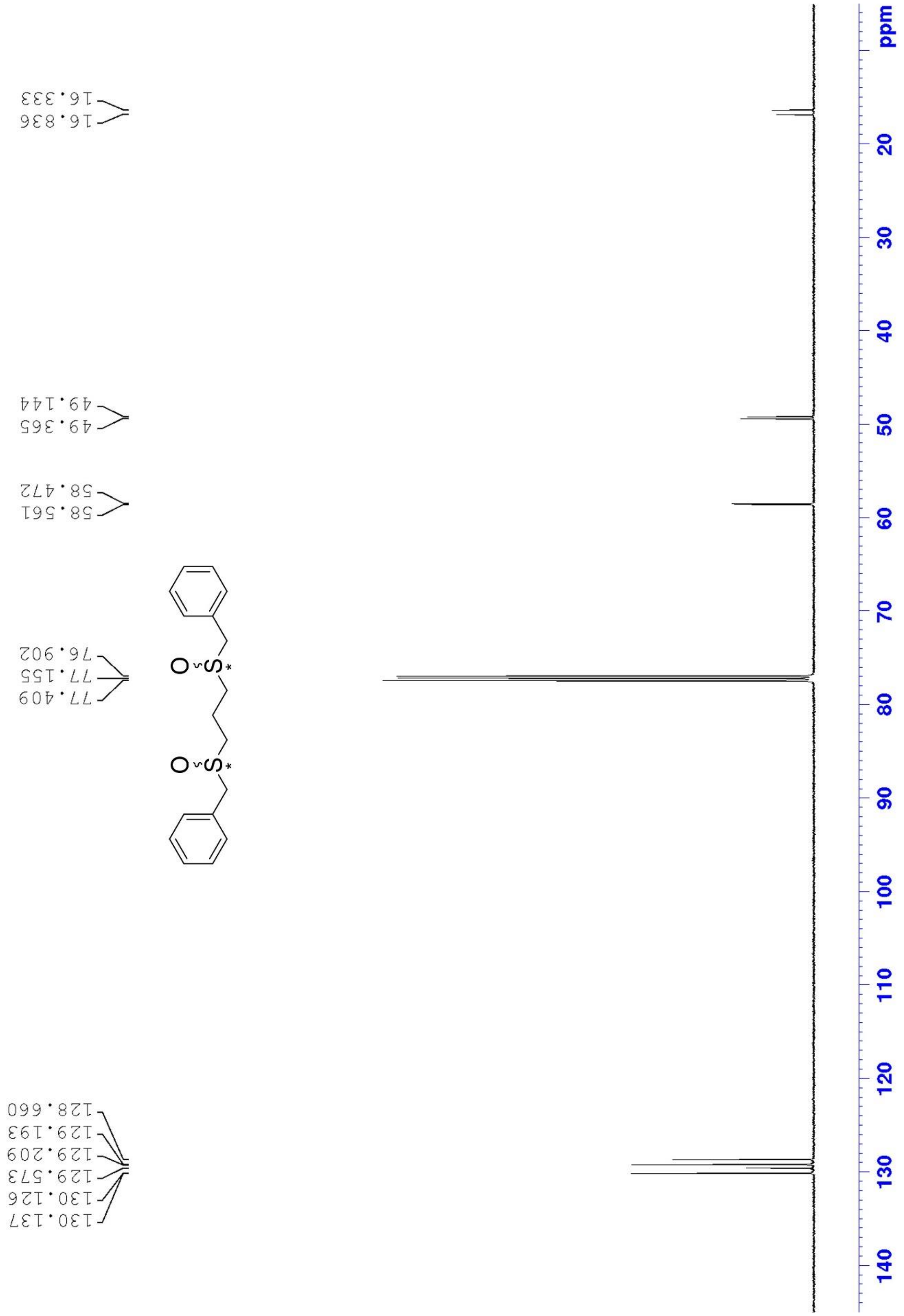

(S,S)/(R,R)/(S,R)-Bis[(pyridin-2-ylmethyl) sulfinyl]propane, **13(*rac+meso*)**

^{1}H NMR (500 MHz, $CDCl_3$)

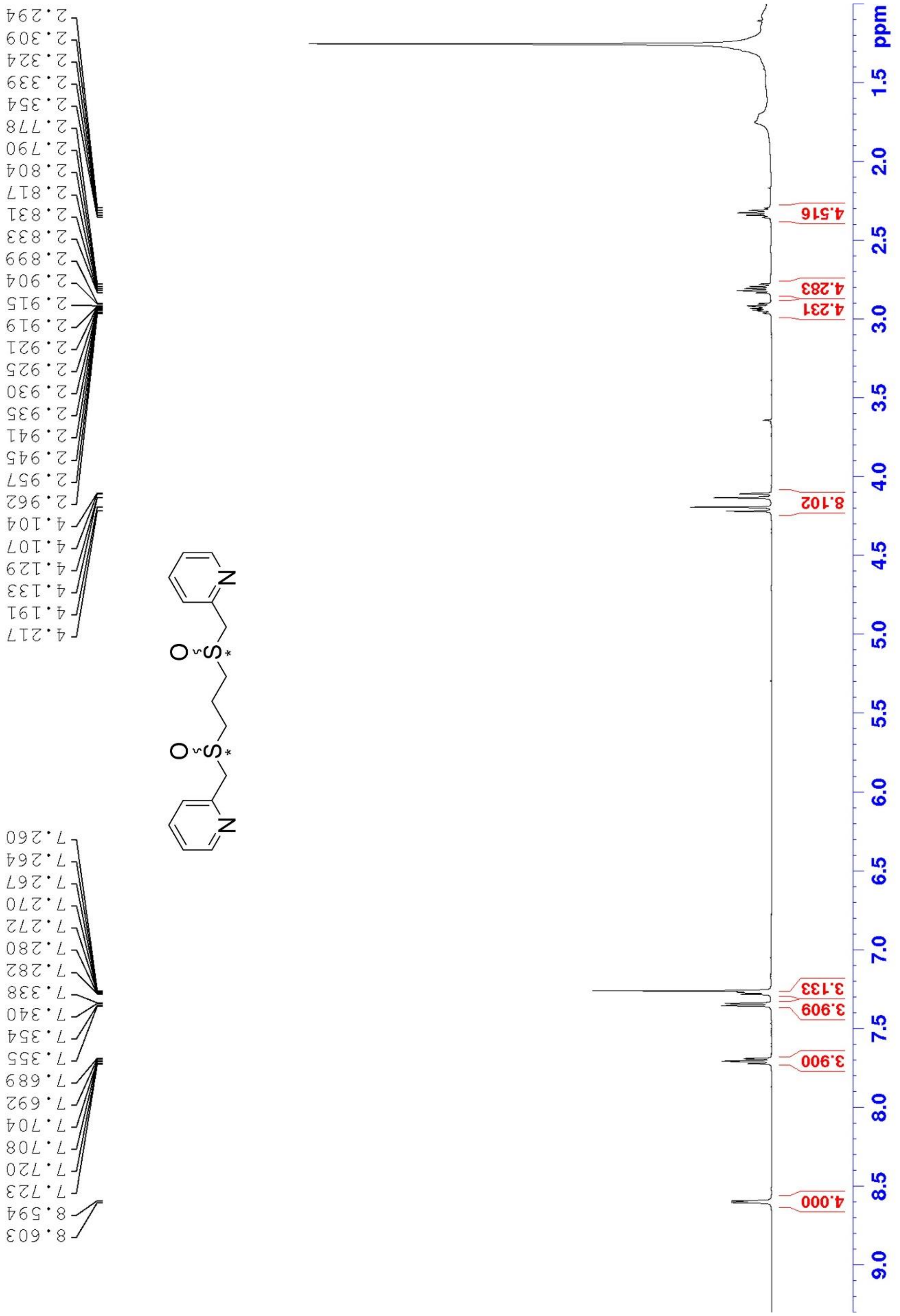

$^{13}C\{^1H\}$ NMR (125 MHz, $CDCl_3$)

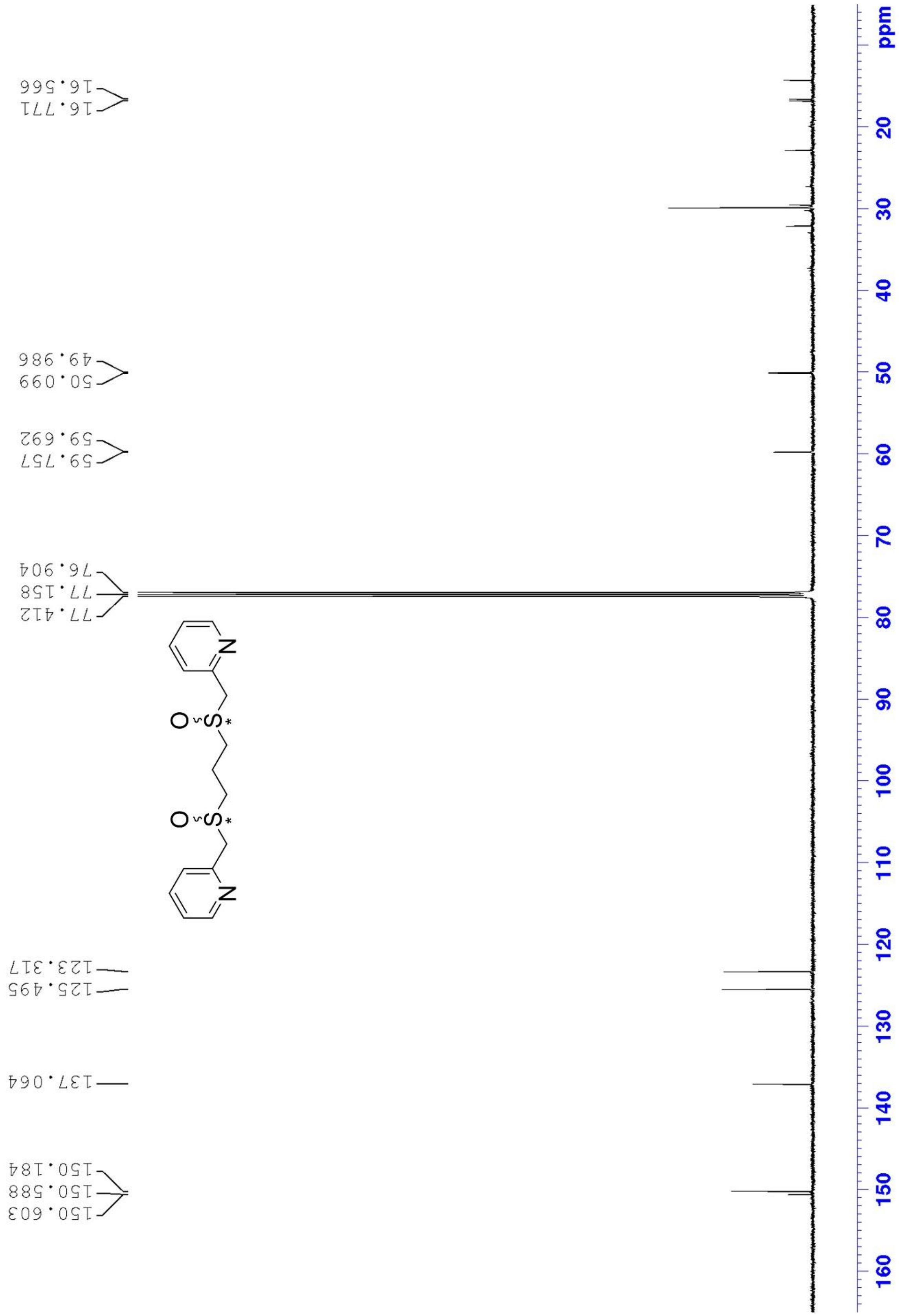

(S,S)/(R,R)/(S,R)-1,3-Bis(methylsulfinyl)propane, **14(*rac+meso*)**

^{1}H NMR (500 MHz, MeOD)

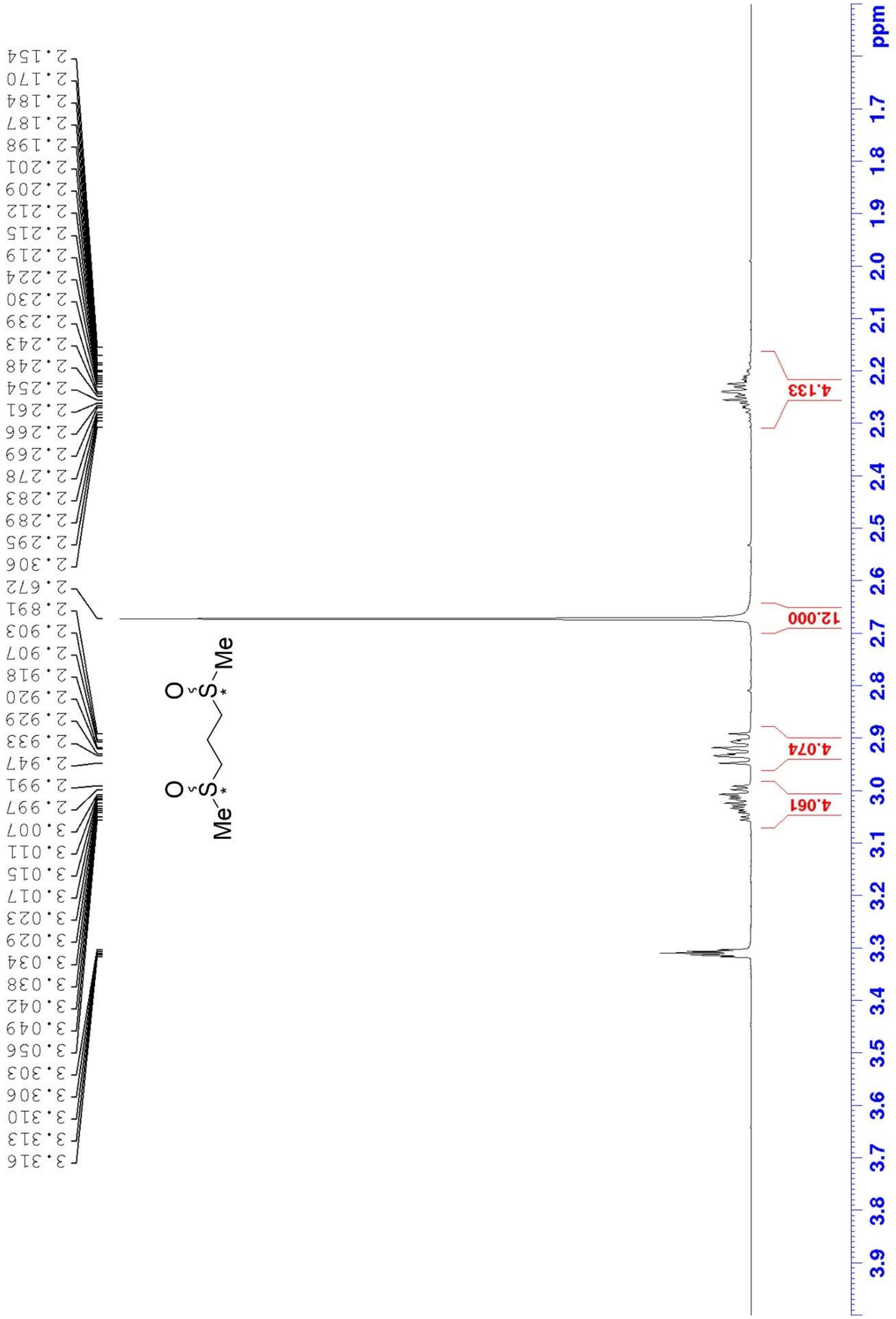

$^{13}C\{^{1}H\}$ NMR (125 MHz, MeOD)

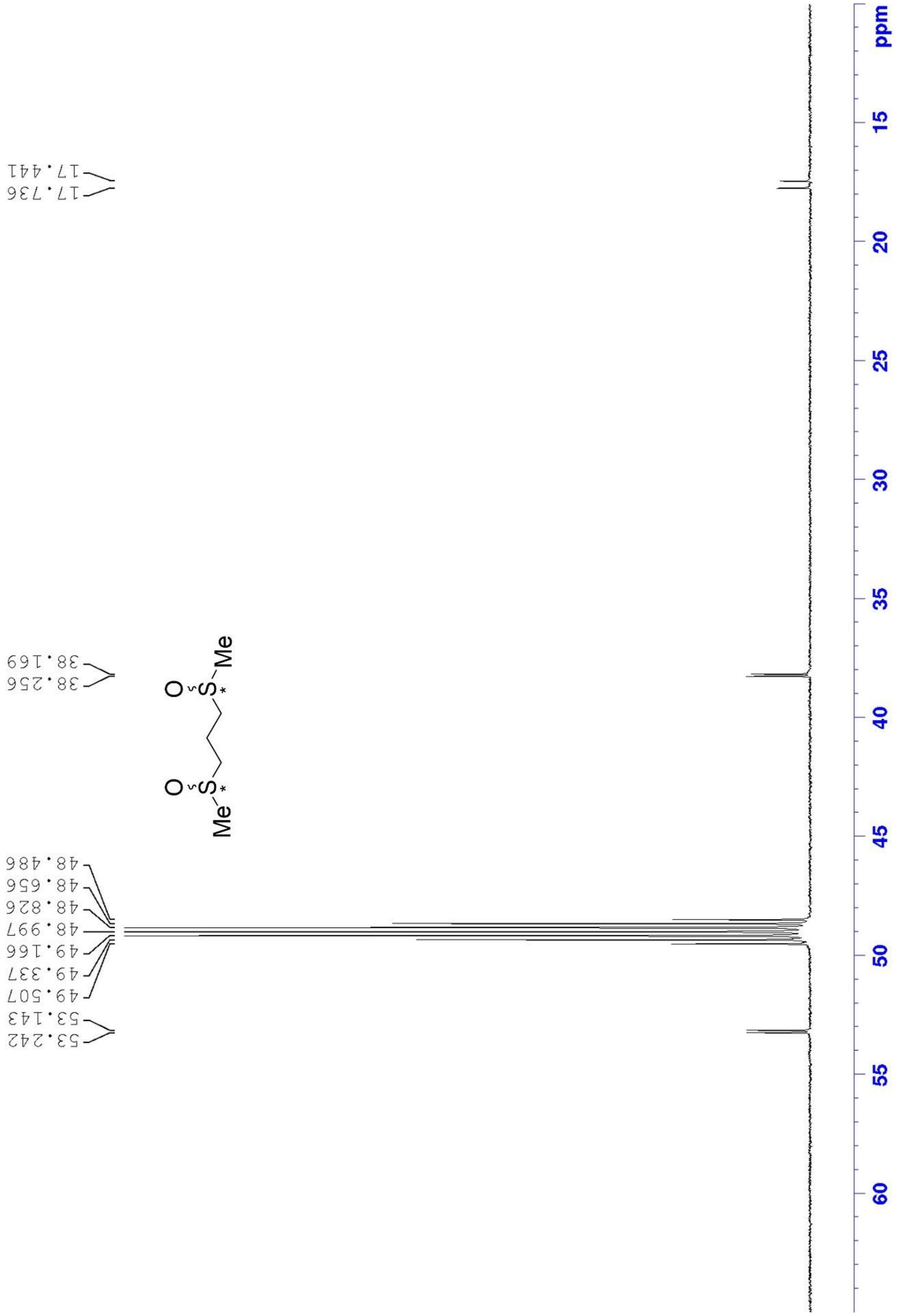

(S,S)/(R,R)/(S,R)-1,3-Bis(ethylsulfinyl)propane, **15(*rac+meso*)**

^{1}H NMR (500 MHz, MeOD)

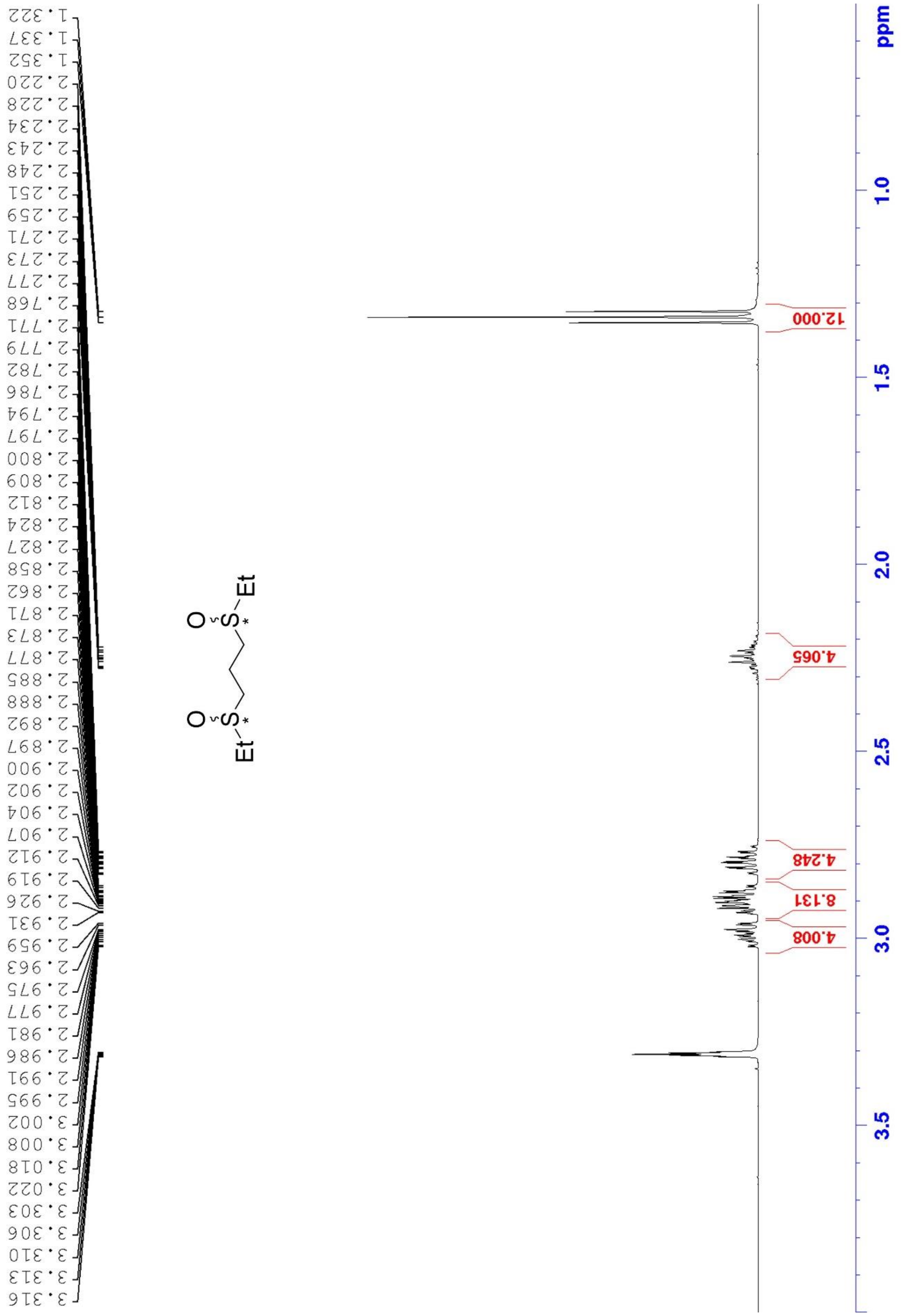

$^{13}C\{^1H\}$ NMR (125 MHz, MeOD)

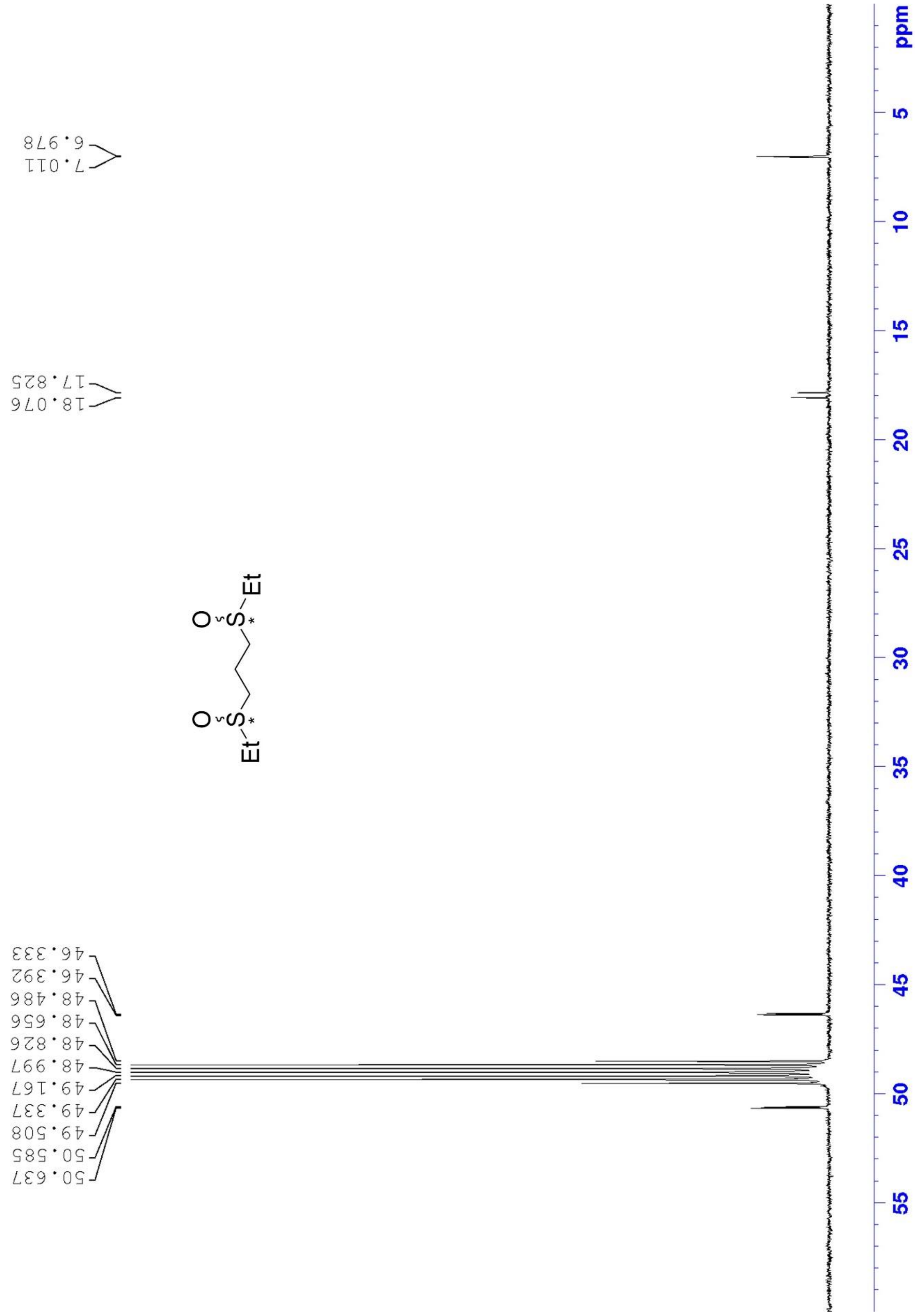

(S,S)/(R,R)/(S,R)-1,3-Bis(propylsulfinyl)propane, **16(*rac+meso*)**

^{1}H NMR (500 MHz, MeOD)

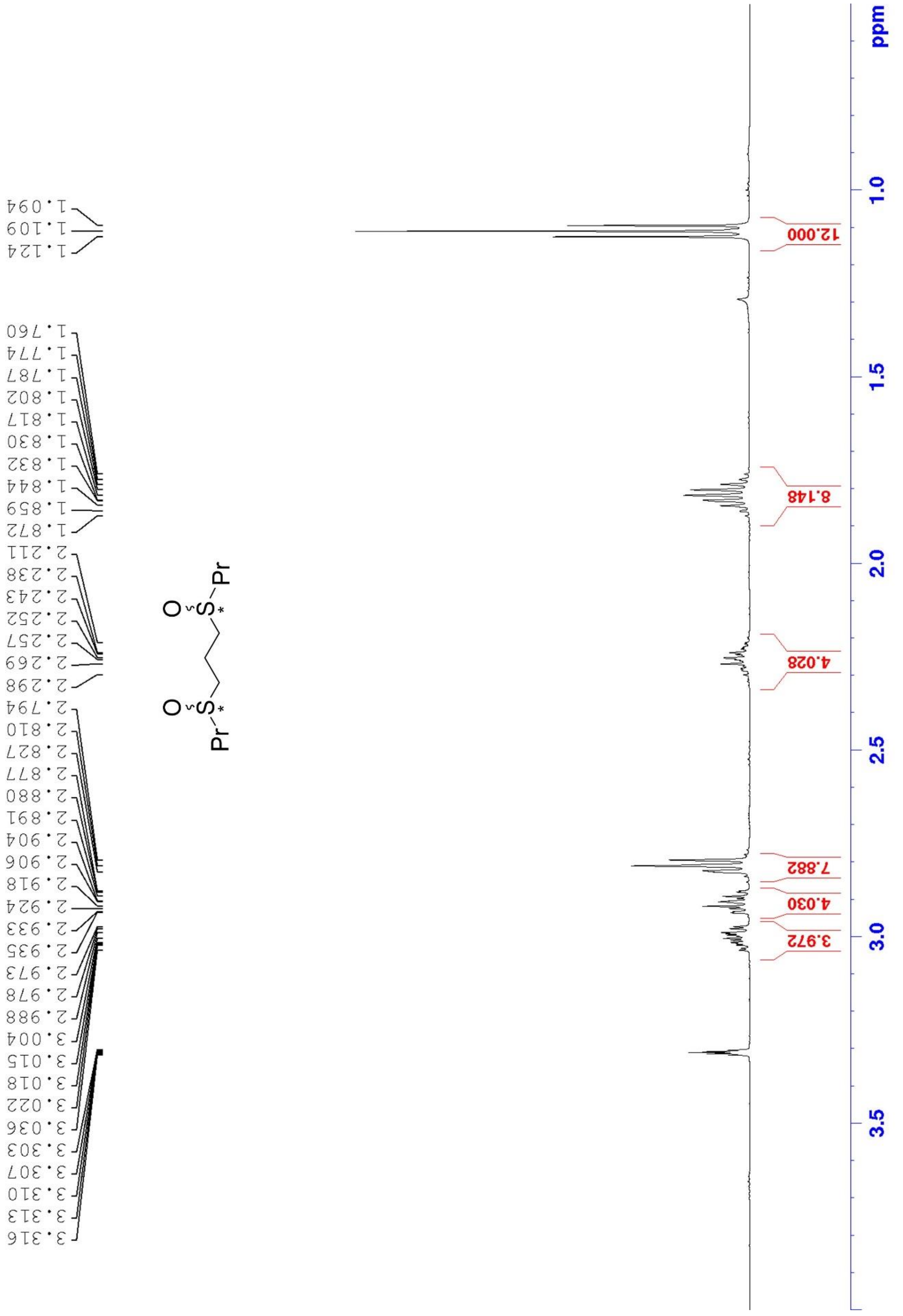

$^{13}C\{^1H\}$ NMR (125 MHz, MeOD)

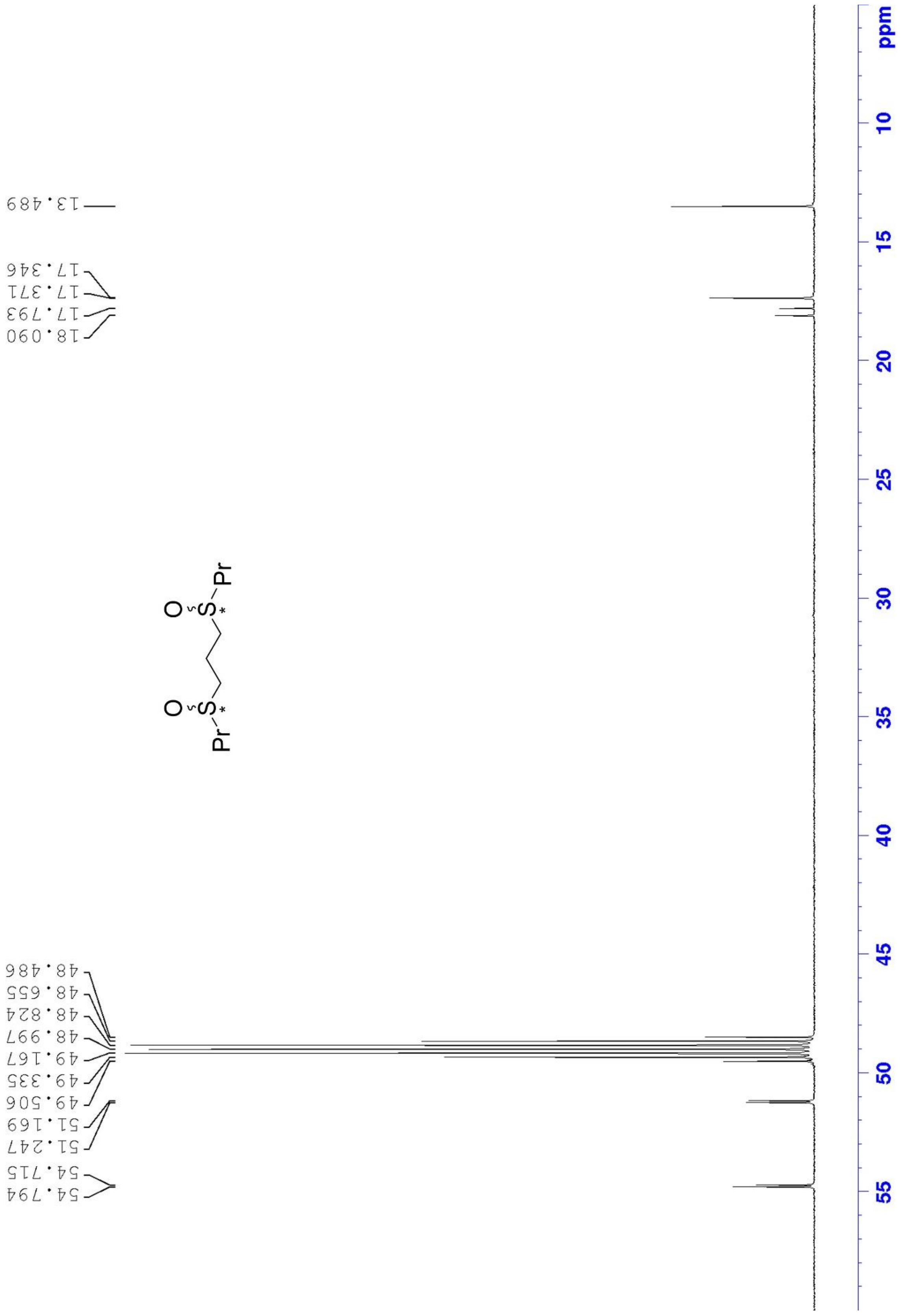

(S,S)/(R,R)/(S,R)-1,3-Bis(isopropylsulfinyl)propane, **17(*rac+meso*)**

^{1}H NMR (500 MHz, MeOD)

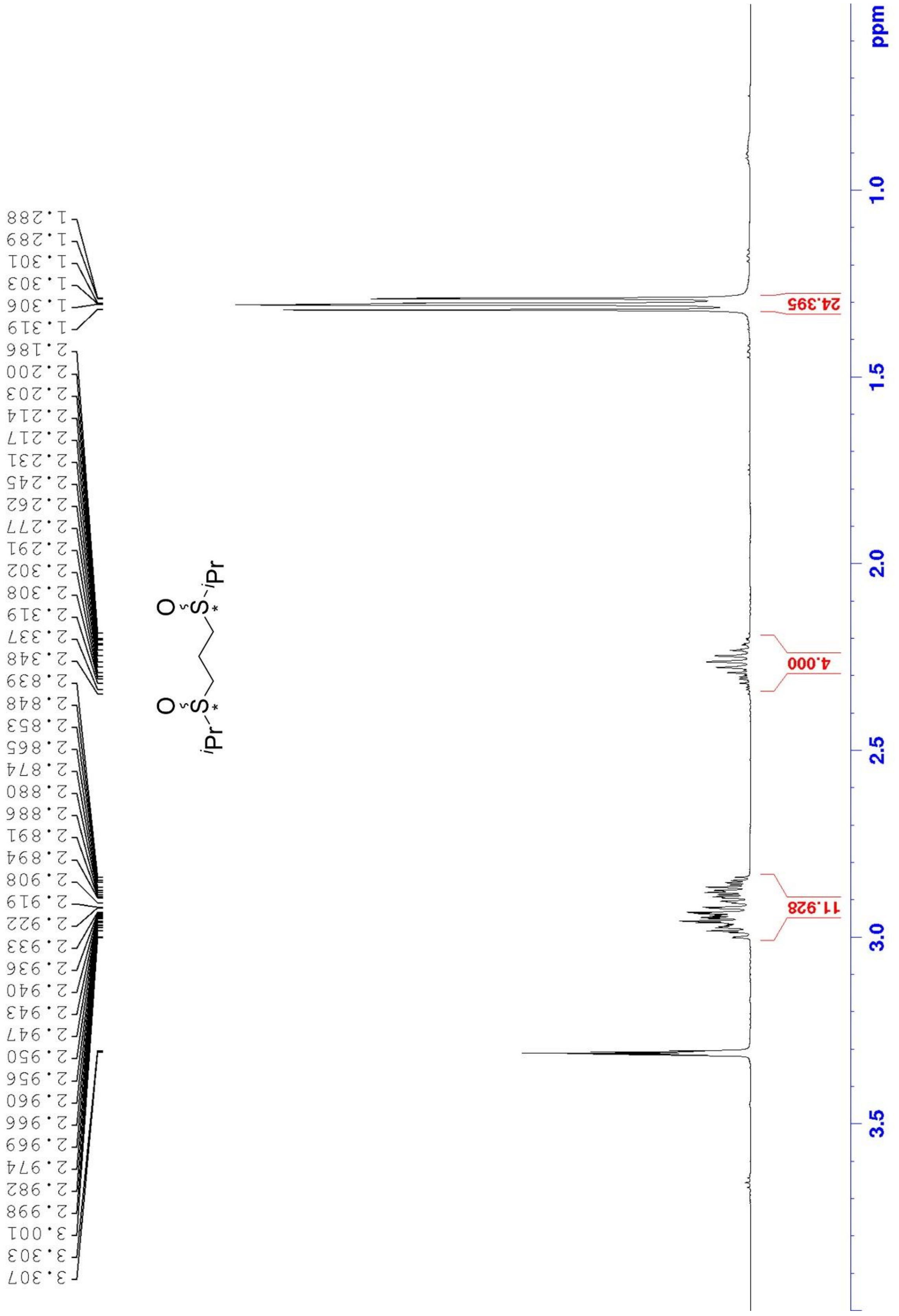

$^{13}C\{^{1}H\}$ NMR (125 MHz, MeOD)

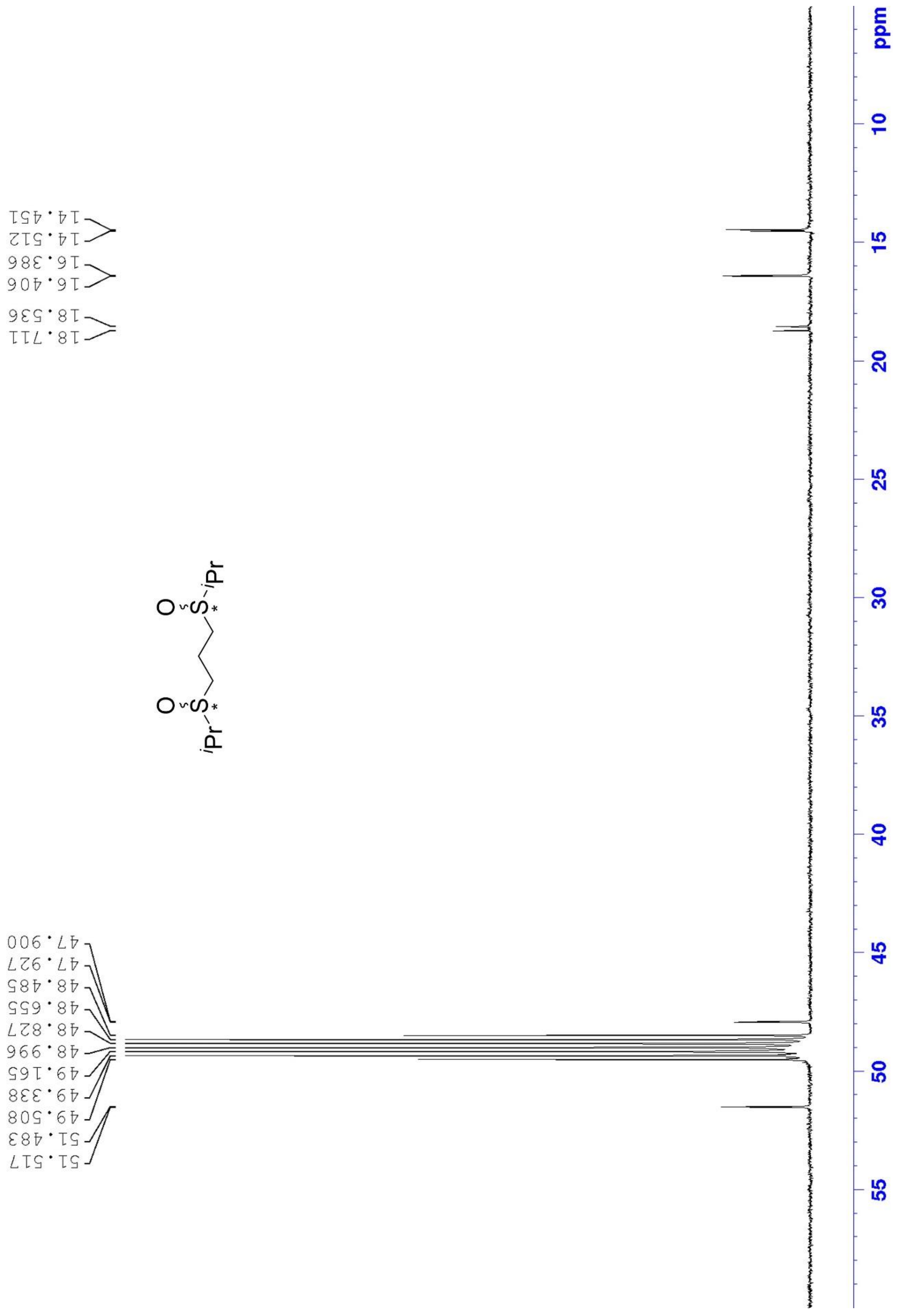

(S,S)/(R,R)/(S,R)-1,3-Bis(tert-butylsulfinyl)propane, **18(*rac+meso*)**

^{1}H NMR (500 MHz, MeOD)

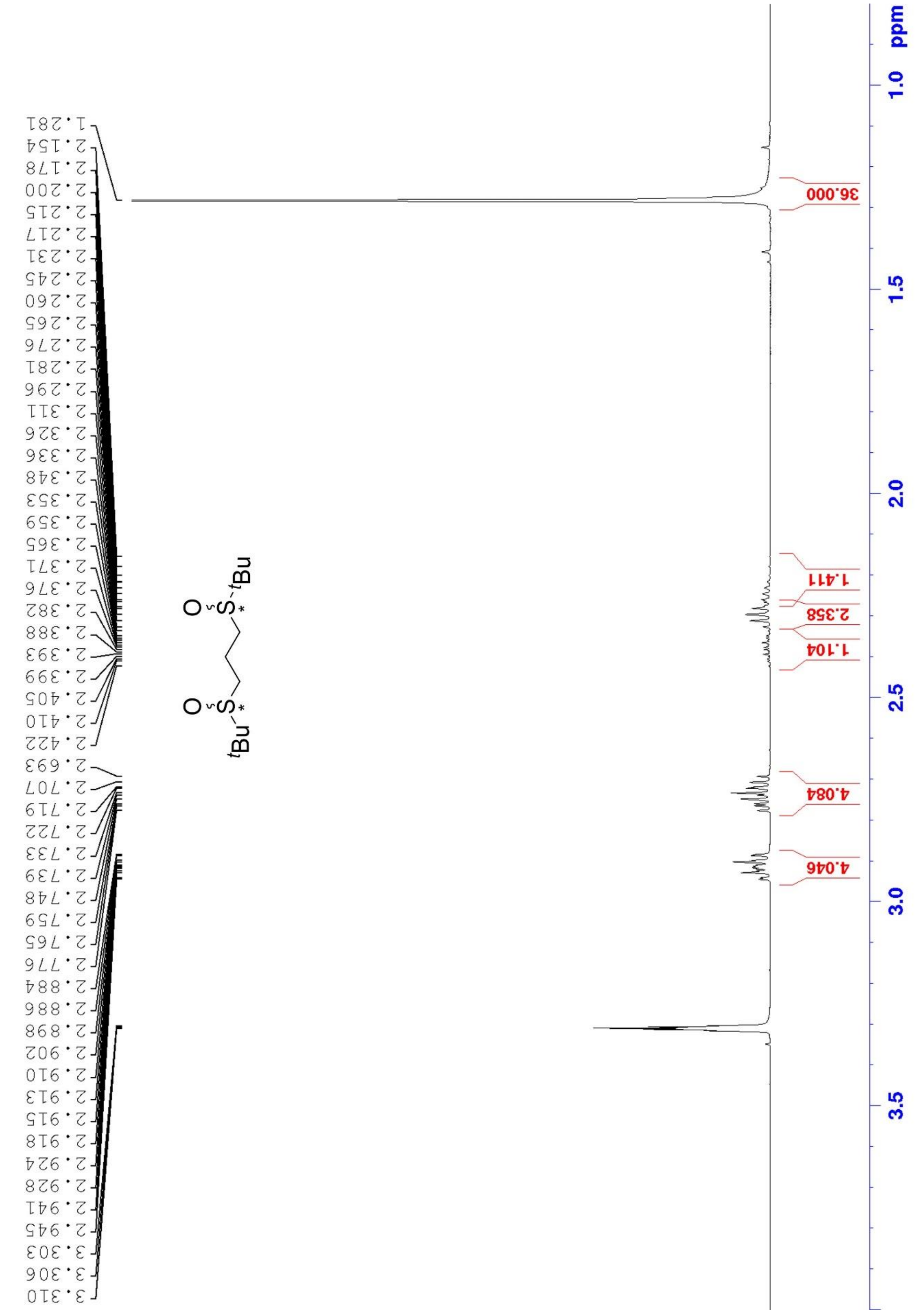

$^{13}C\{^{1}H\}$ NMR (125 MHz, MeOD)

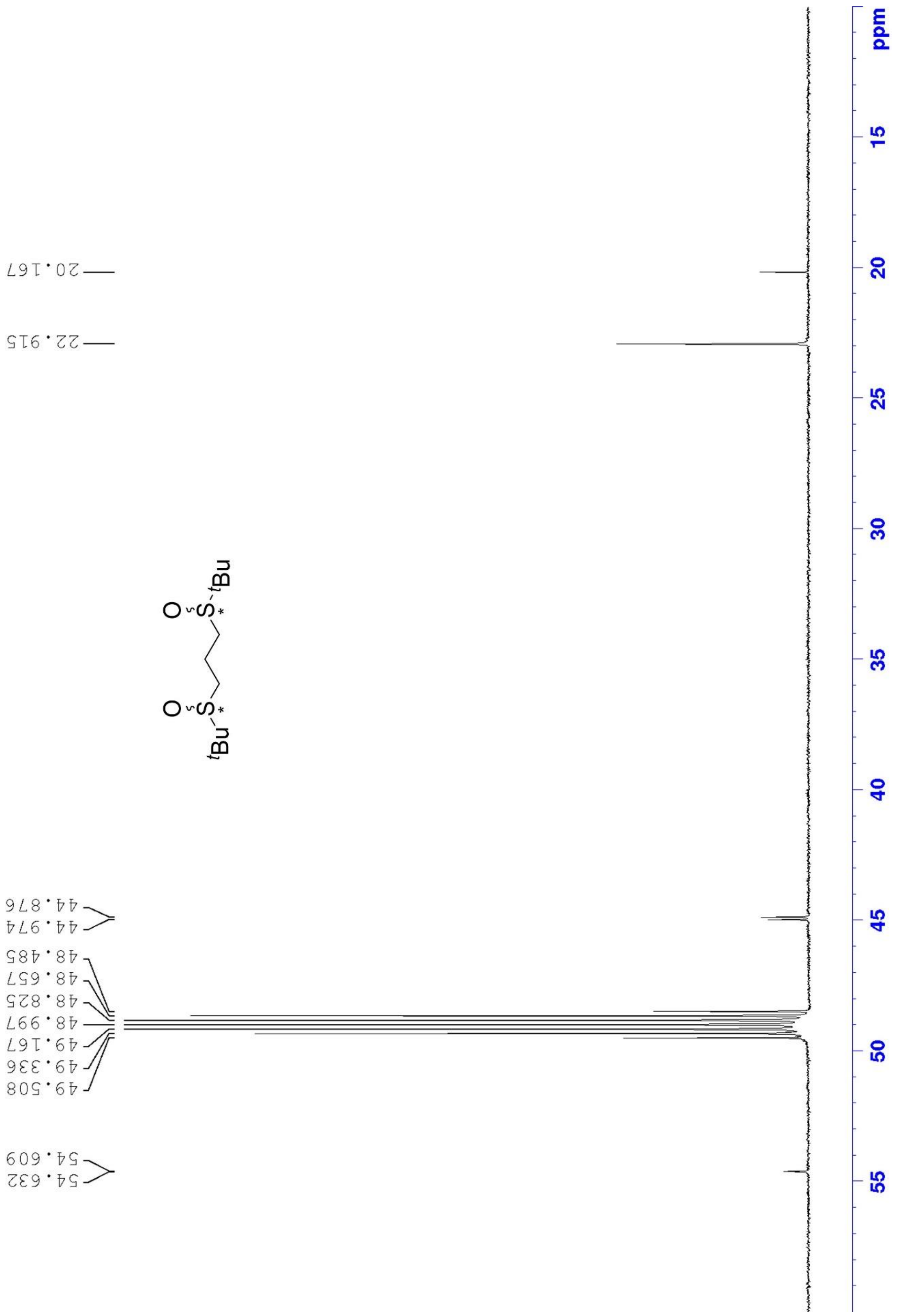

(R)-p-Tolyl vinyl sulfoxide, **25(*R*)**

^{1}H NMR (500 MHz, $CDCl_3$)

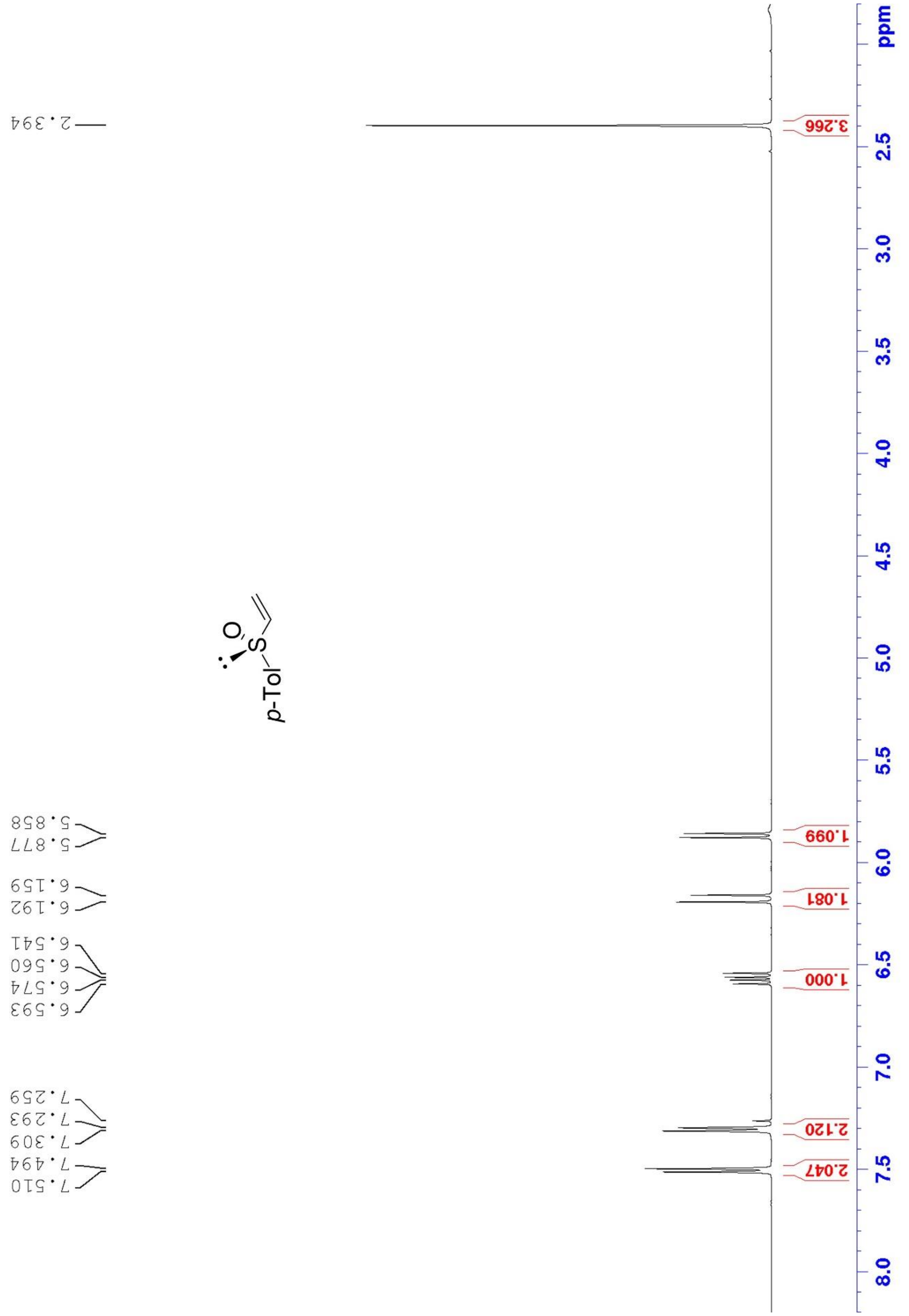

$^{13}C\{^{1}H\}$ NMR (125 MHz, $CDCl_3$)

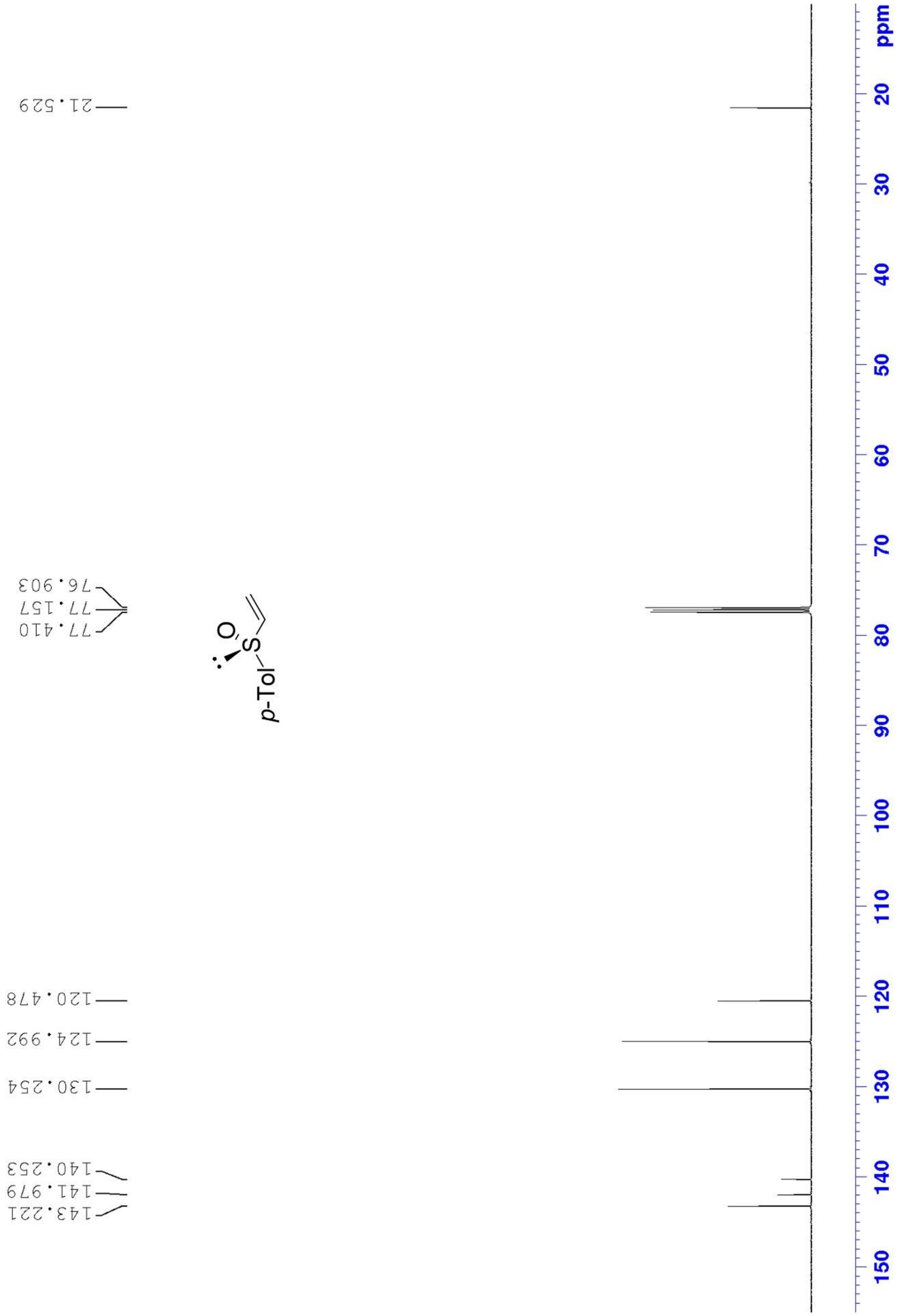

(S)-p-Tolyl vinyl sulfoxide, **25(*S*)**

^{1}H NMR (500 MHz, $CDCl_3$)

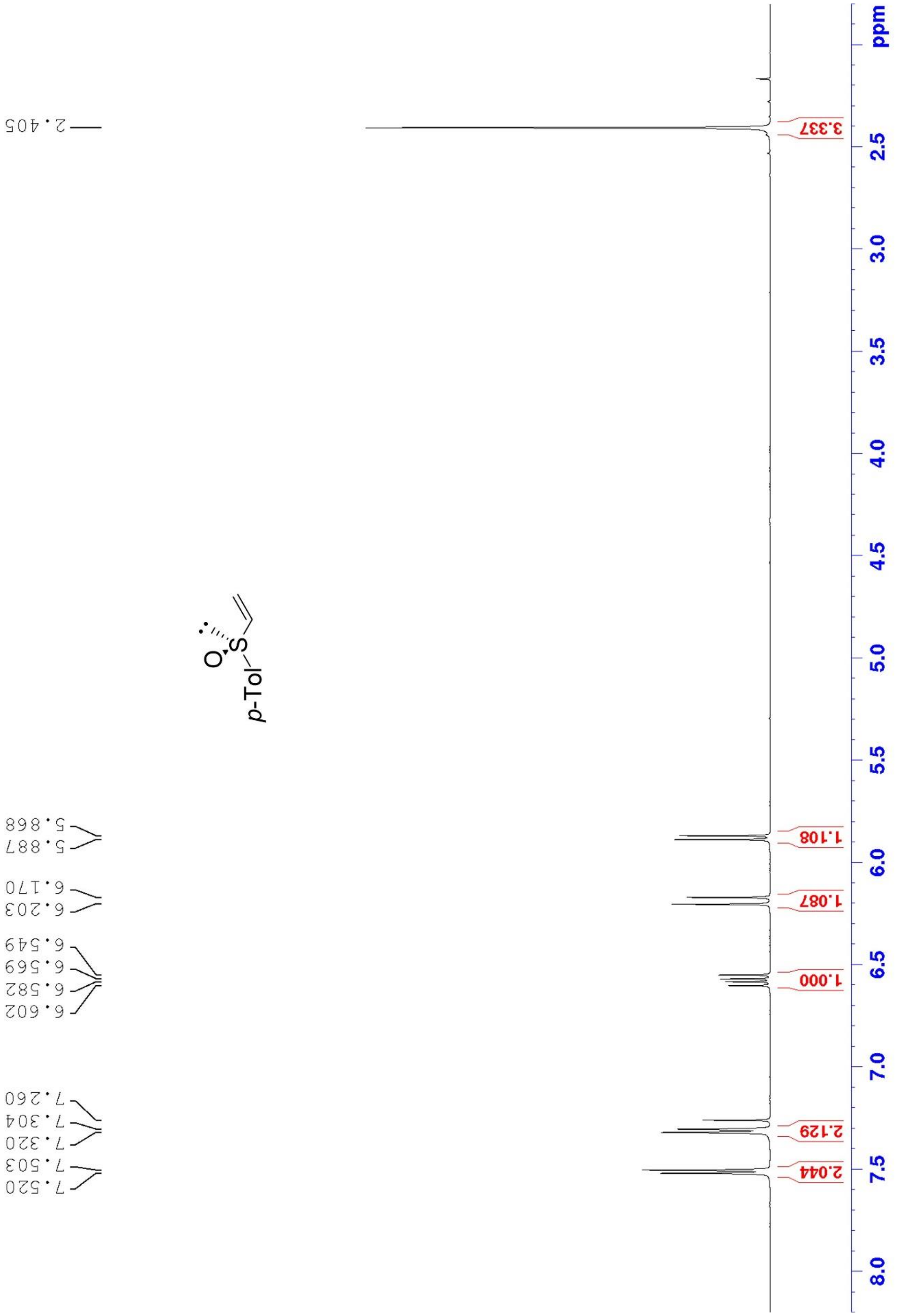

$^{13}C\{^{1}H\}$ NMR (125 MHz, $CDCl_3$)

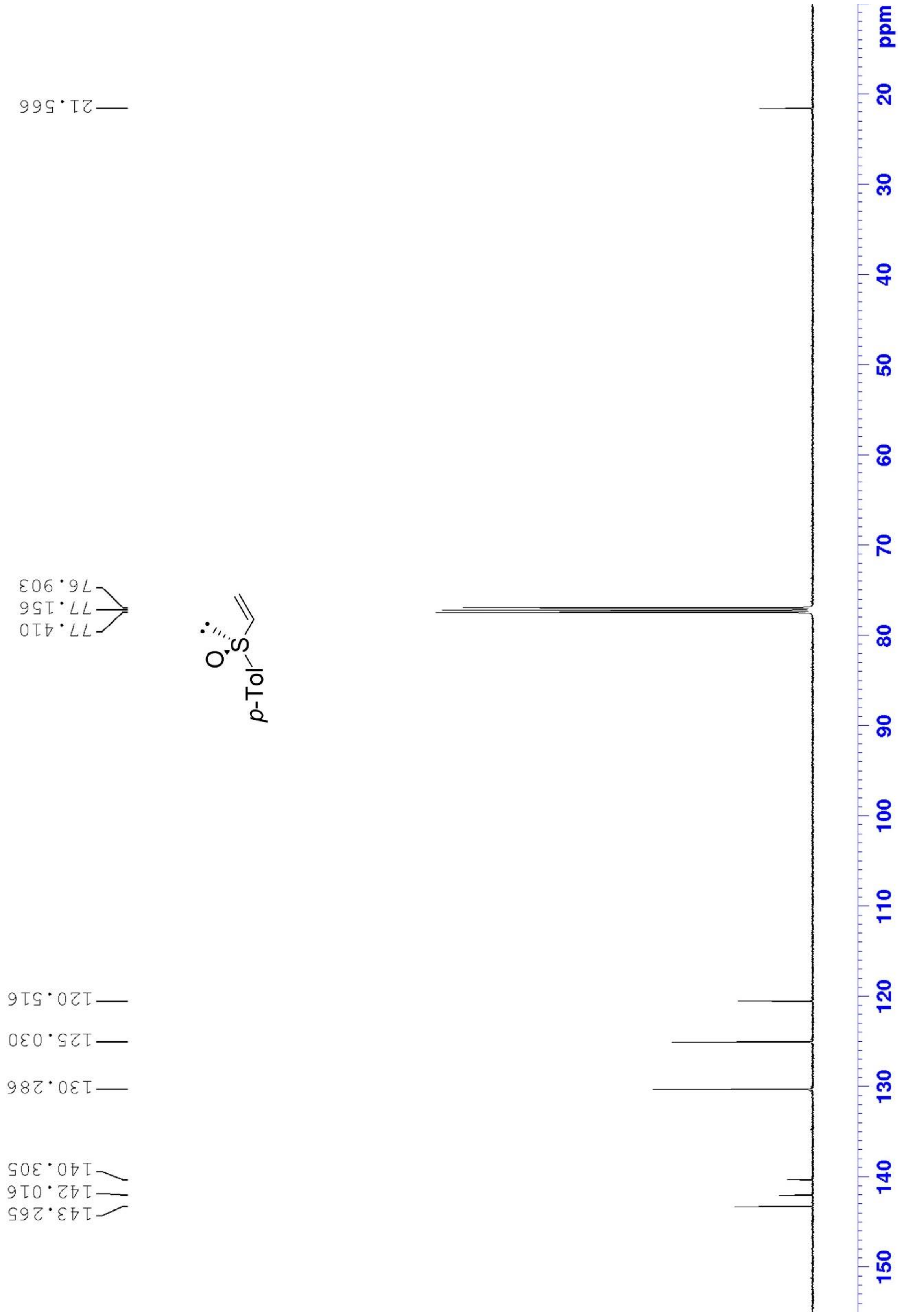

(R)-tert-Butyl vinyl sulfoxide, **26(*R*)**

^{1}H NMR (500 MHz, $CDCl_3$)

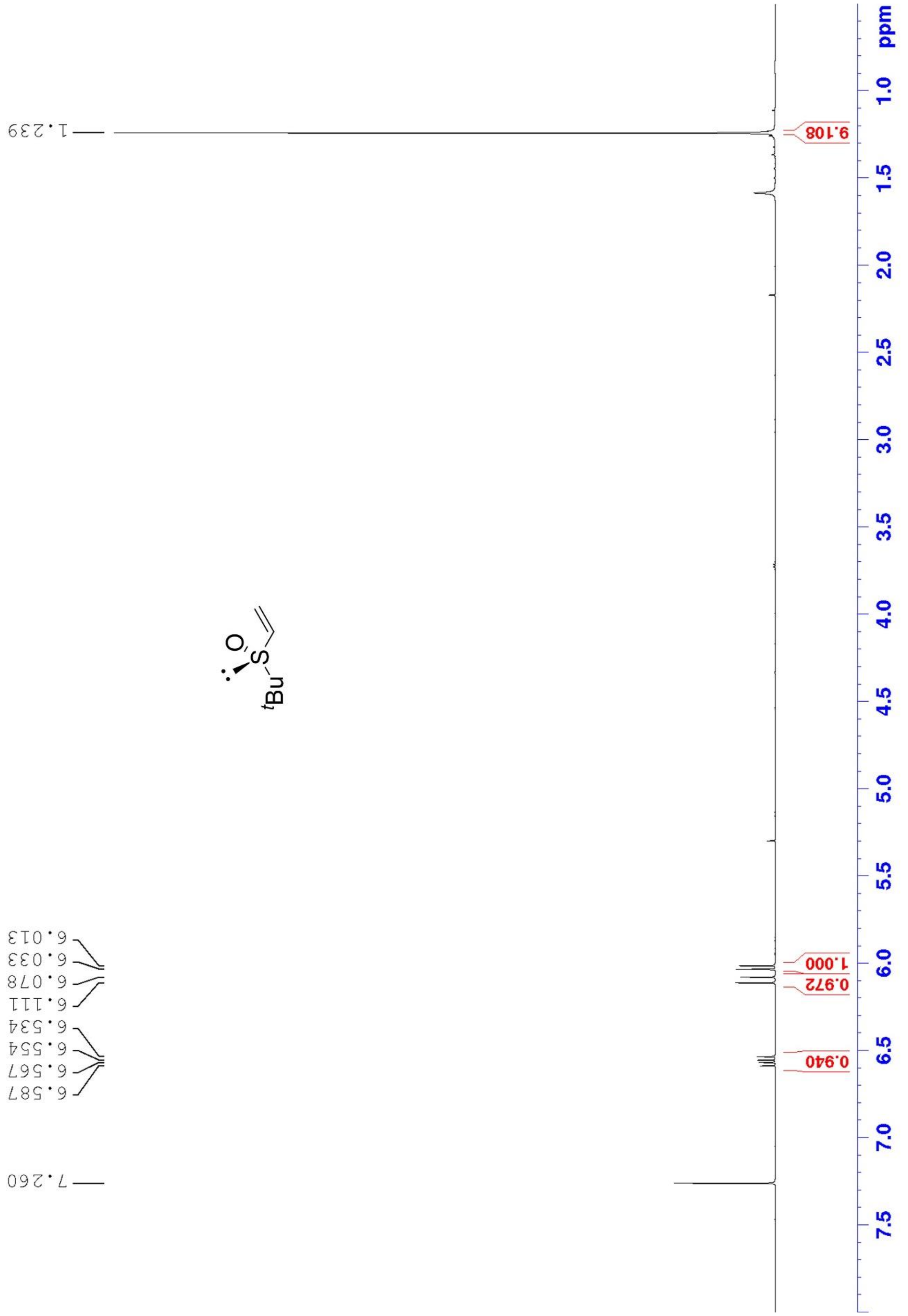

$^{13}C\{^1H\}$ NMR (125 MHz, $CDCl_3$)

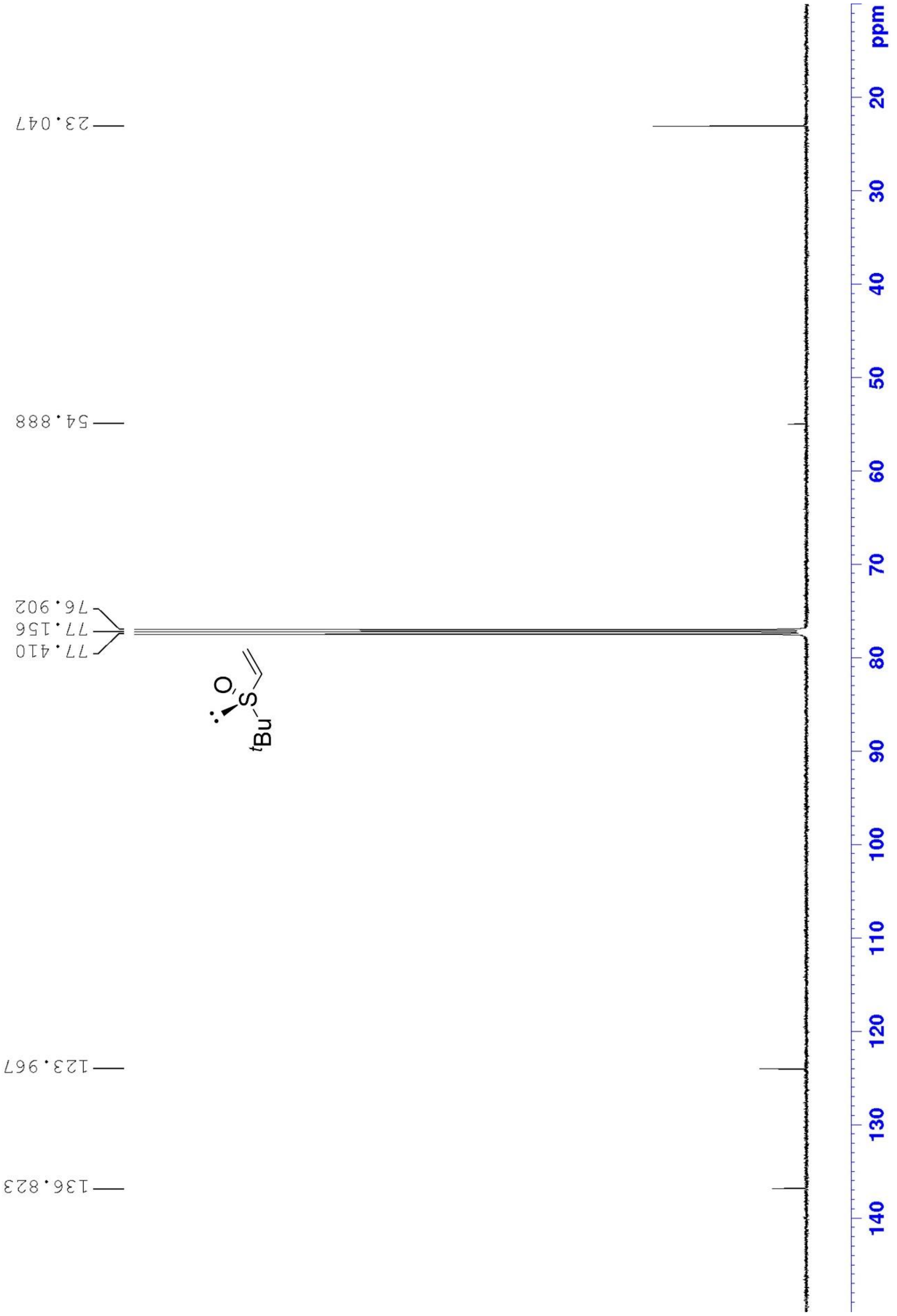

(S)-tert-Butyl vinyl sulfoxide, **26(*S*)**

^{1}H NMR (500 MHz, $CDCl_3$)

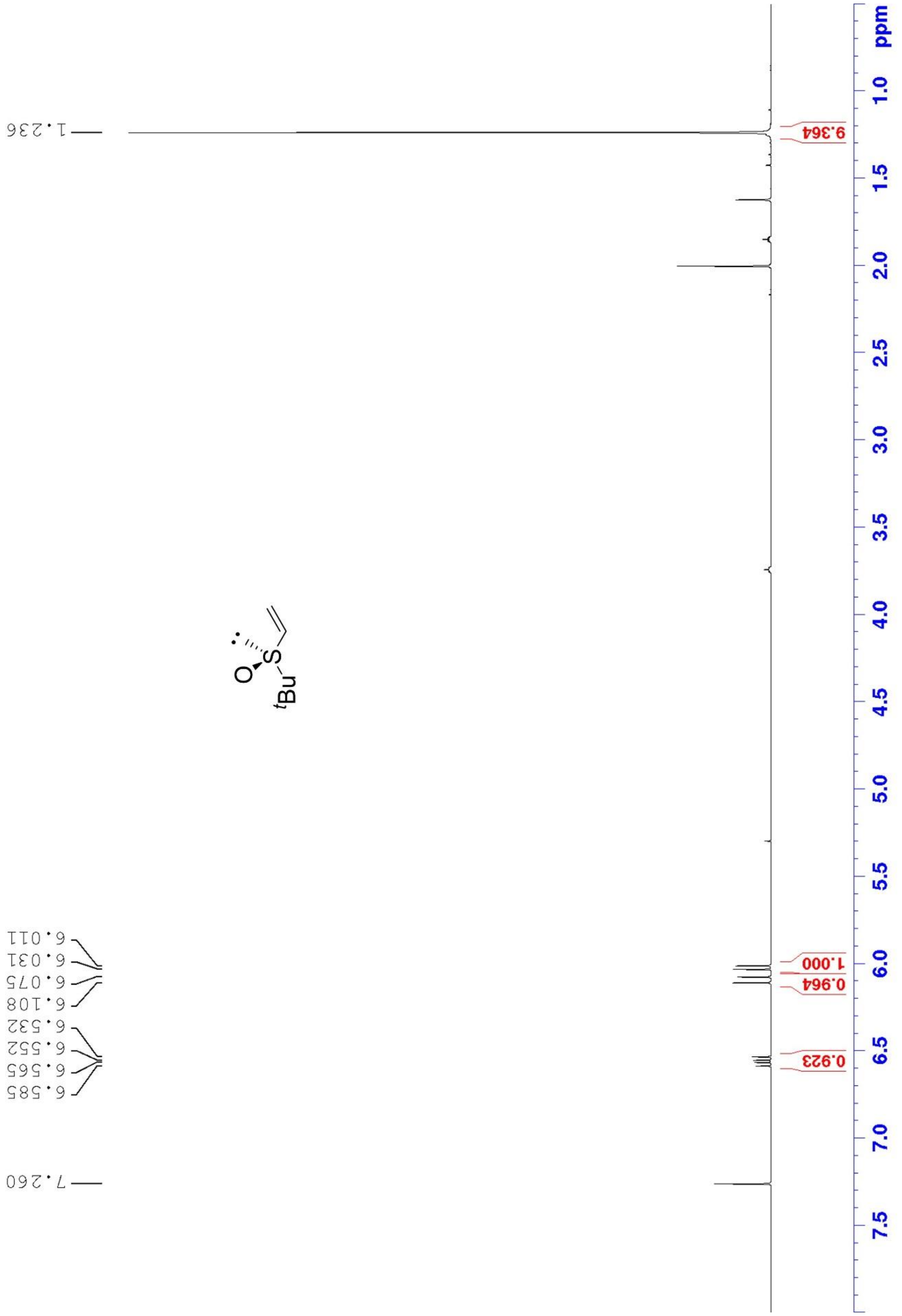

$^{13}C\{^1H\}$ NMR (125 MHz, $CDCl_3$)

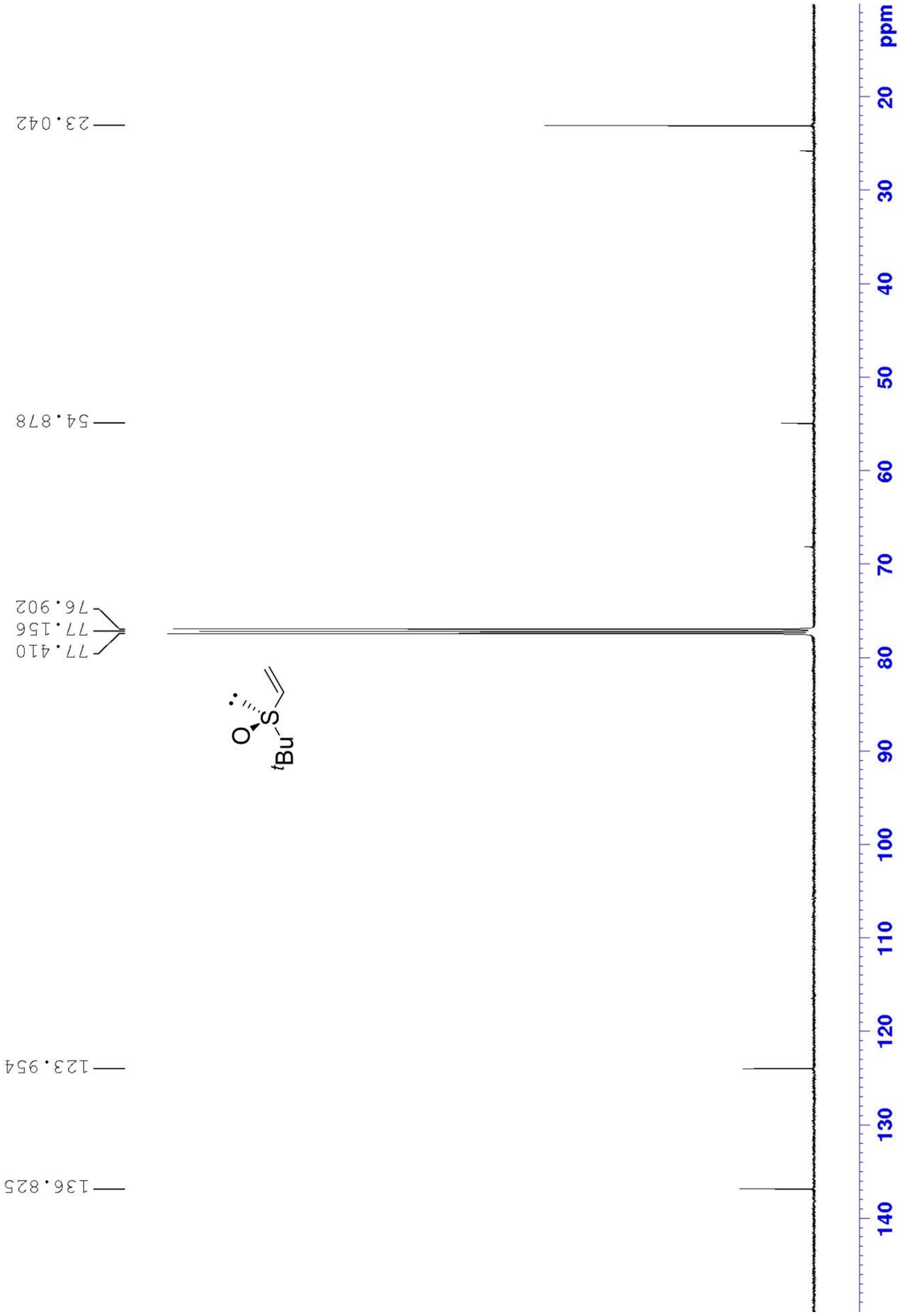

(S)-Methyl vinyl sulfoxide, **27(*S*)**

^{1}H NMR (500 MHz, $CDCl_3$)

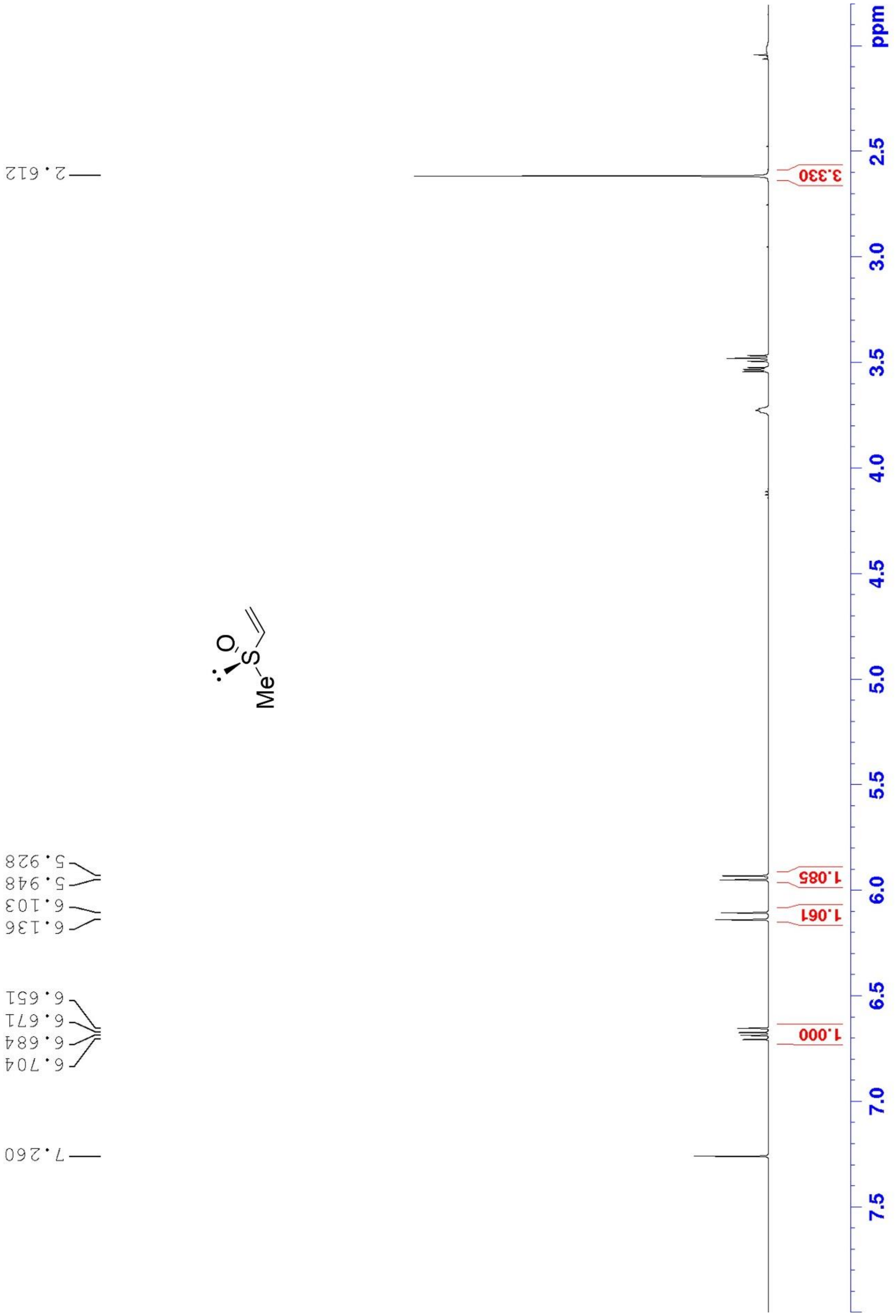

$^{13}C\{^{1}H\}$ NMR (125 MHz, $CDCl_3$)

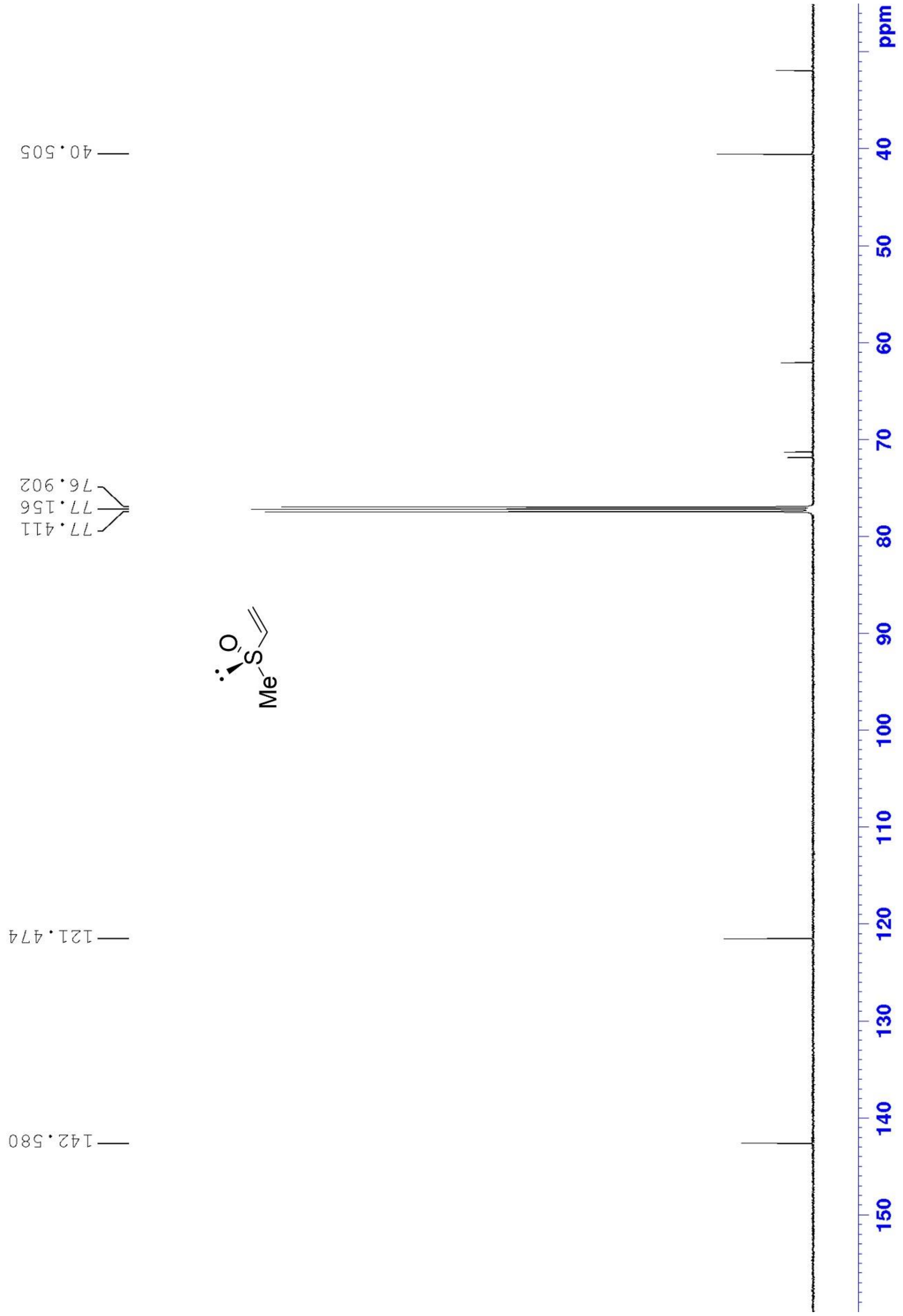

(R)-Methyl vinyl sulfoxide, **27(*R*)**

^{1}H NMR (500 MHz, $CDCl_3$)

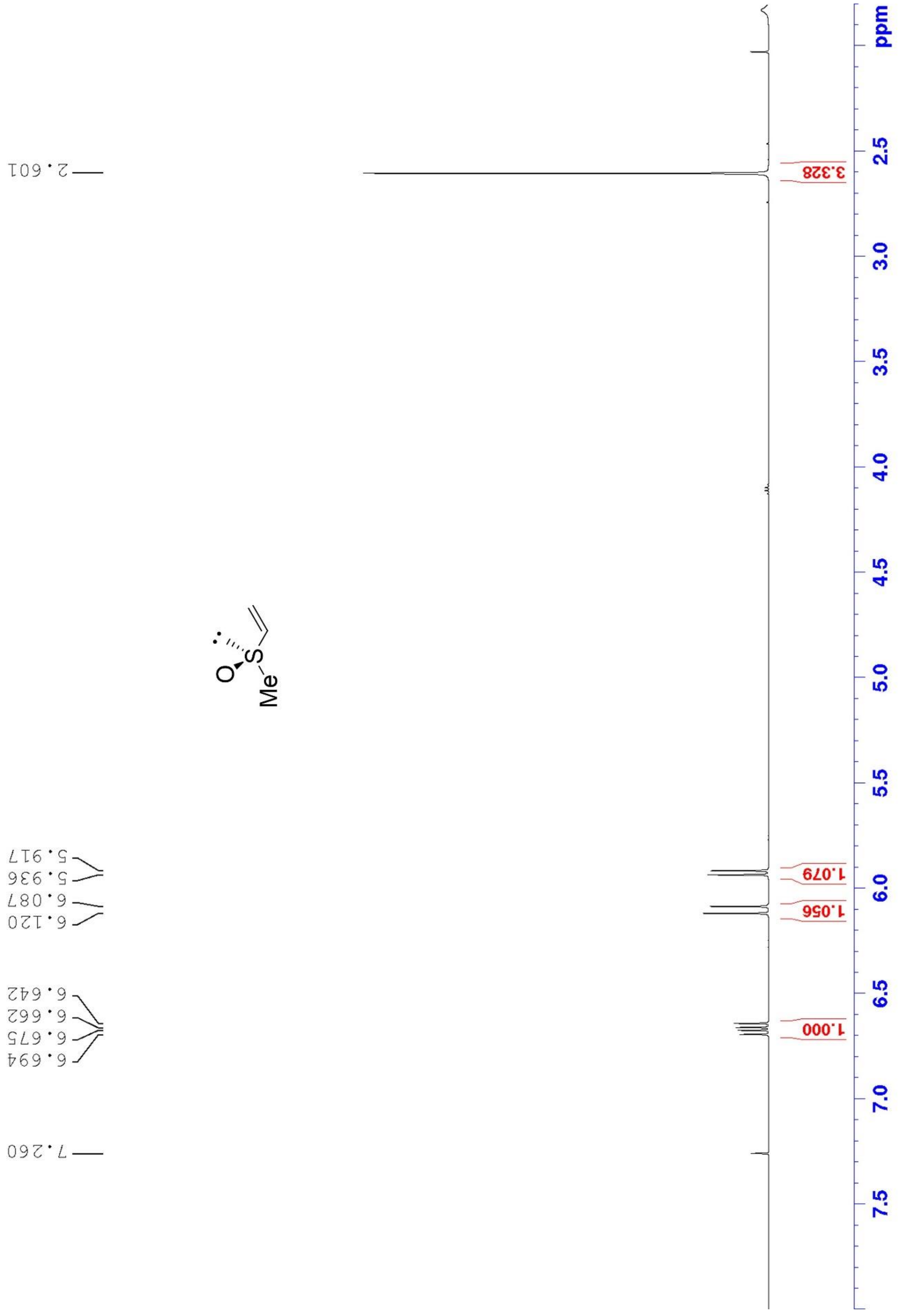

$^{13}C\{^{1}H\}$ NMR (125 MHz, $CDCl_3$)

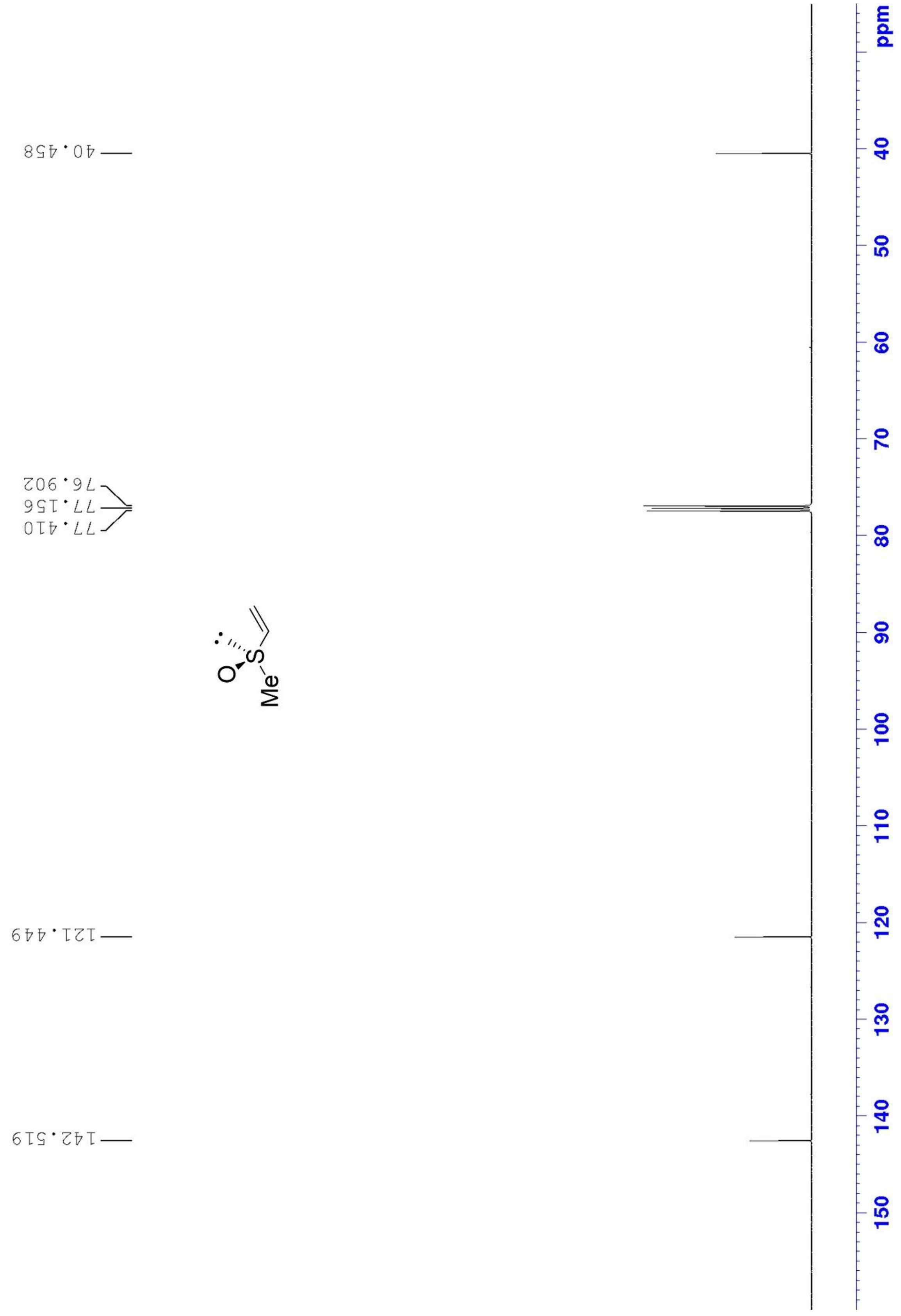

(rac)-p-Tolyl vinyl sulfoxide, **25(*rac*)**

^{1}H NMR (500 MHz, $CDCl_3$)

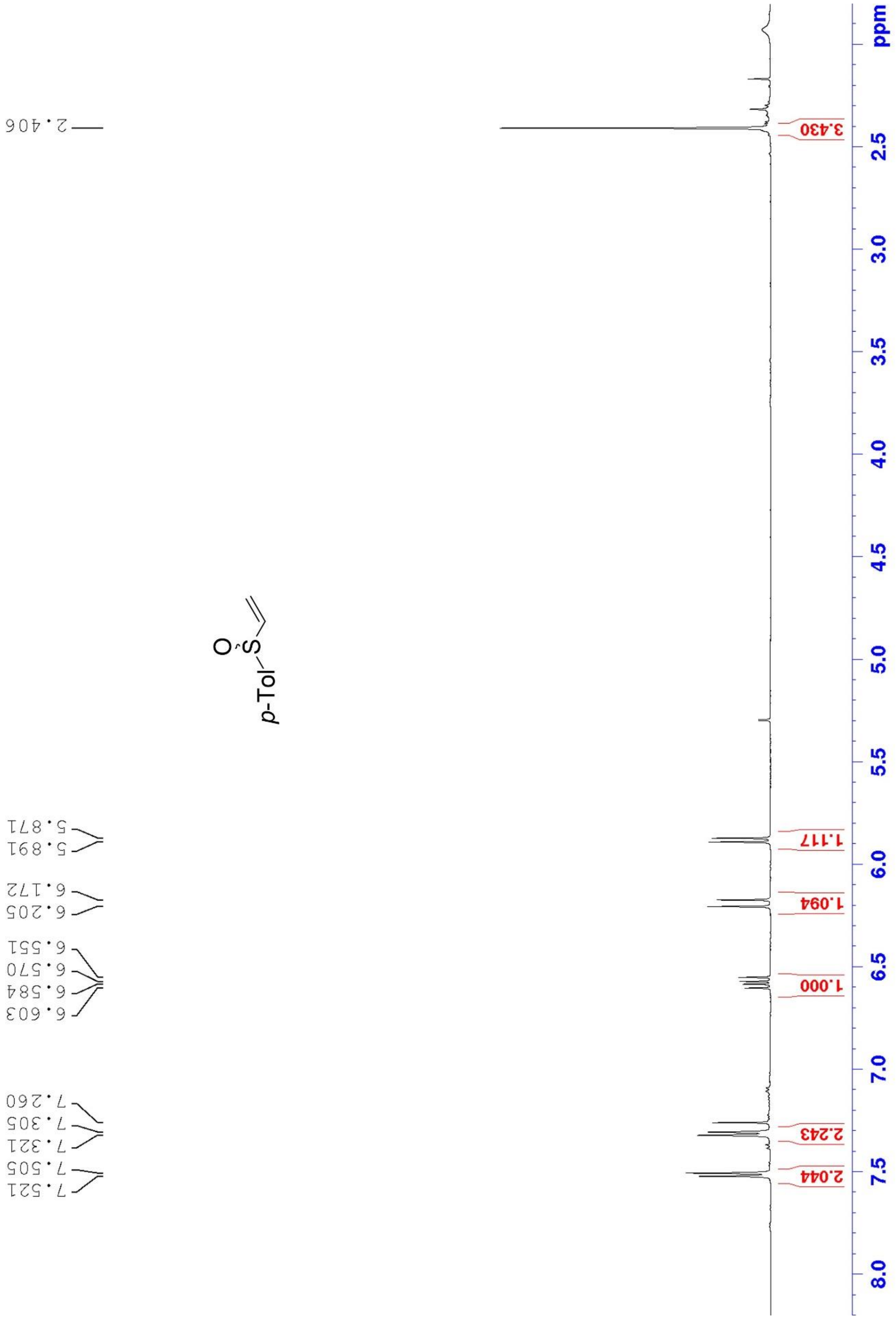

$^{13}C\{^1H\}$ NMR (125 MHz, $CDCl_3$)

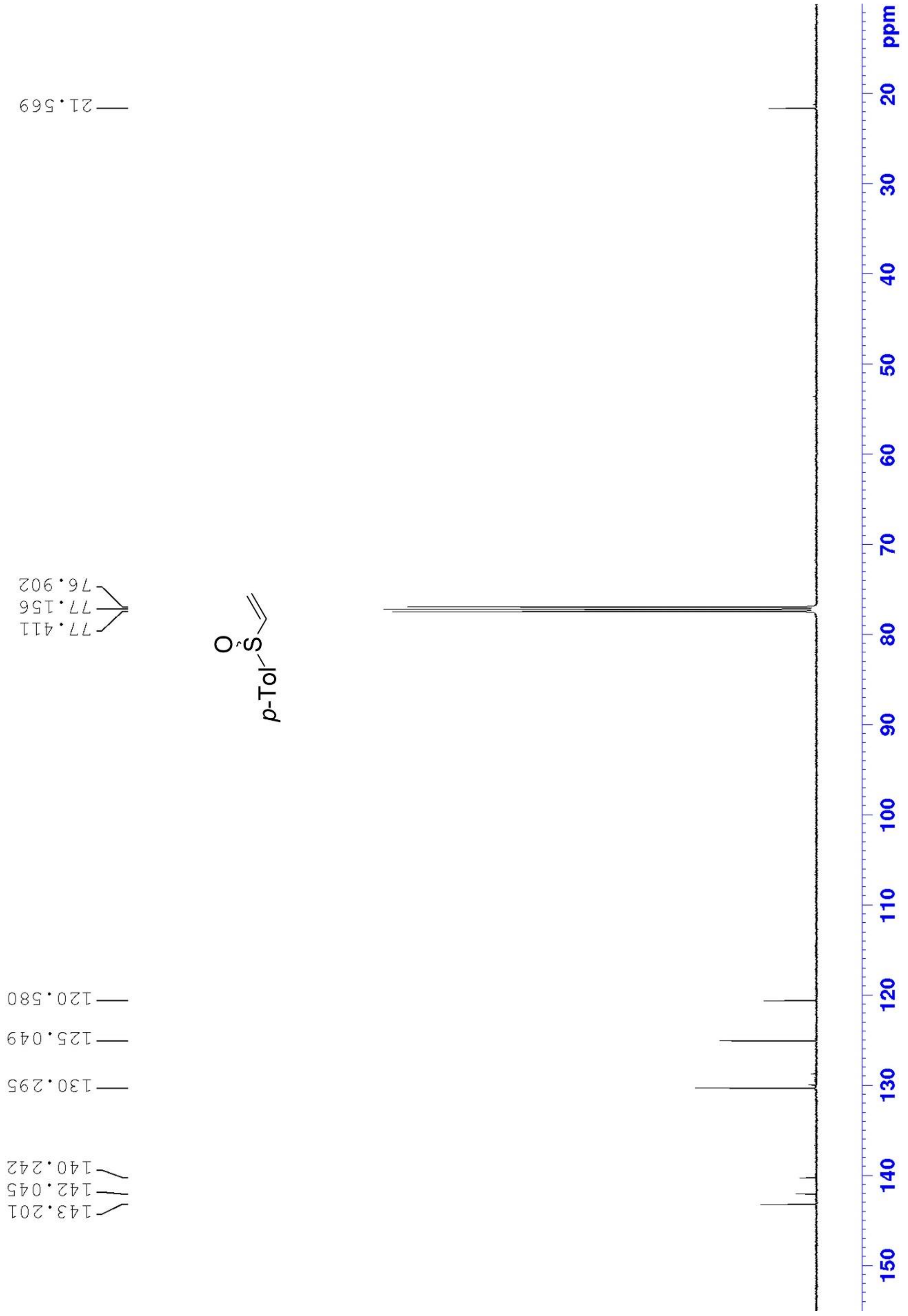

(rac)-tert-Butyl vinyl sulfoxide, **26(*rac*)**

^{1}H NMR (500 MHz, $CDCl_3$)

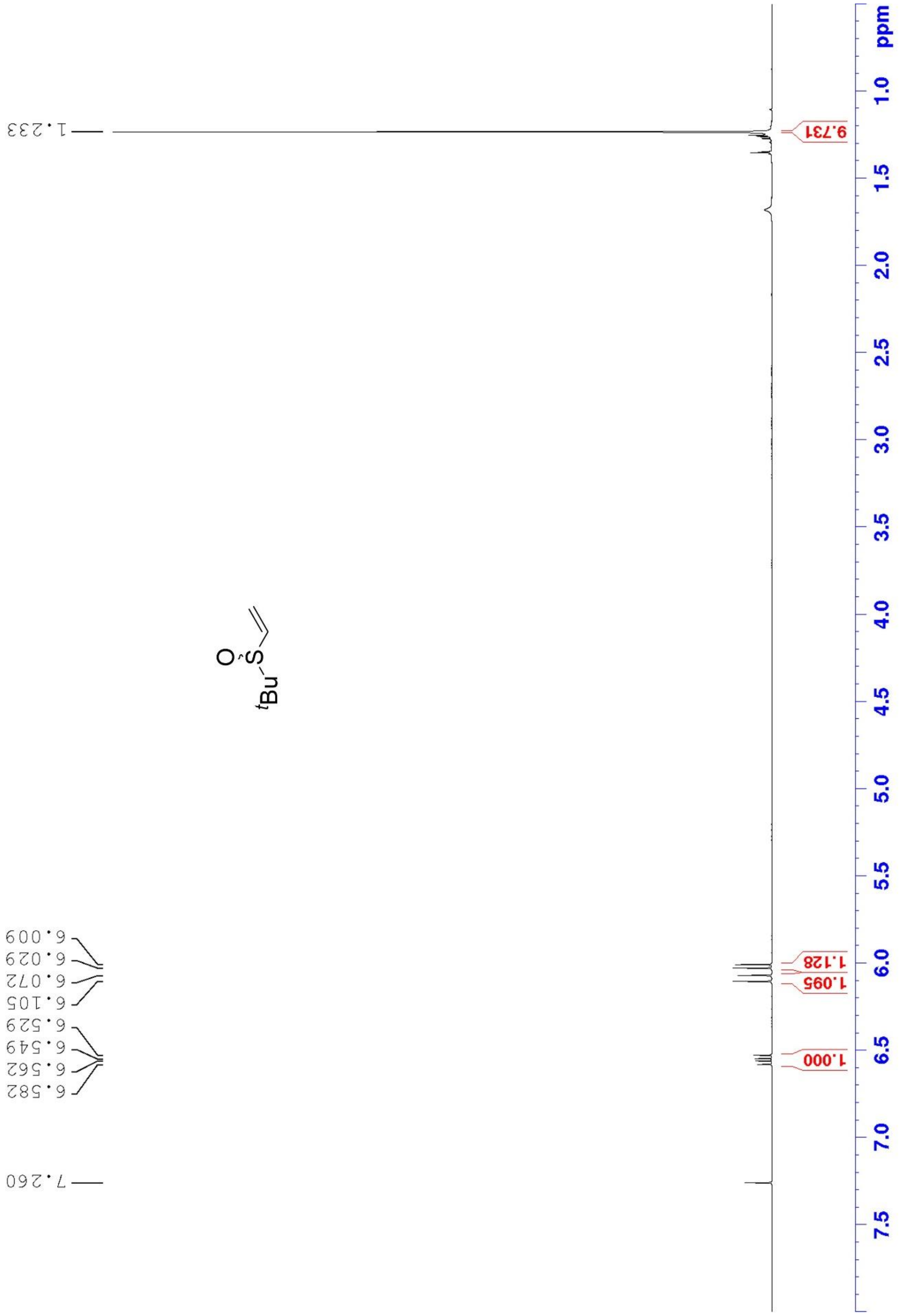

$^{13}C\{^1H\}$ NMR (125 MHz, $CDCl_3$)

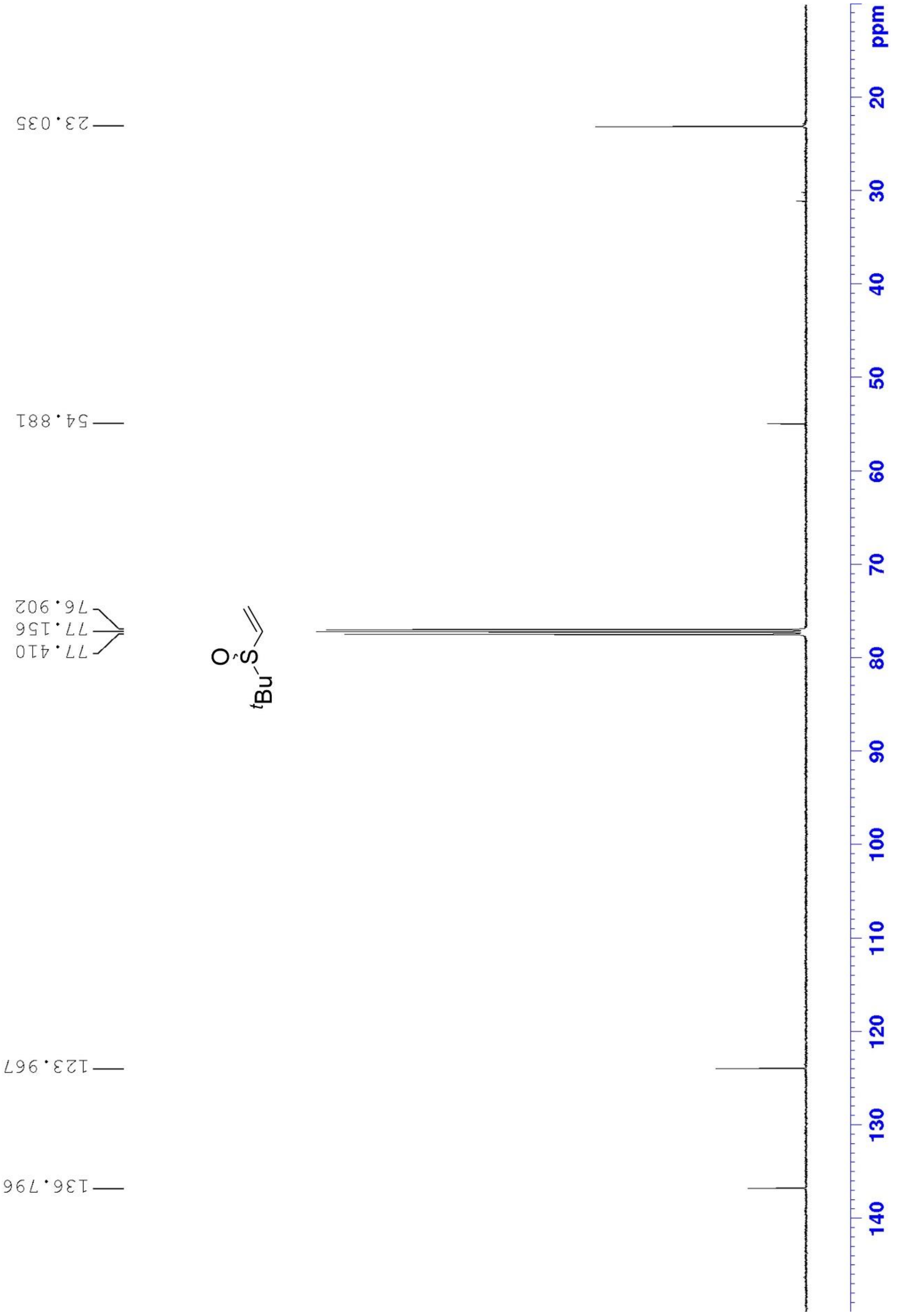

(rac)-Methyl vinyl sulfoxide, **27(*rac*)**

^{1}H NMR (500 MHz, $CDCl_3$)

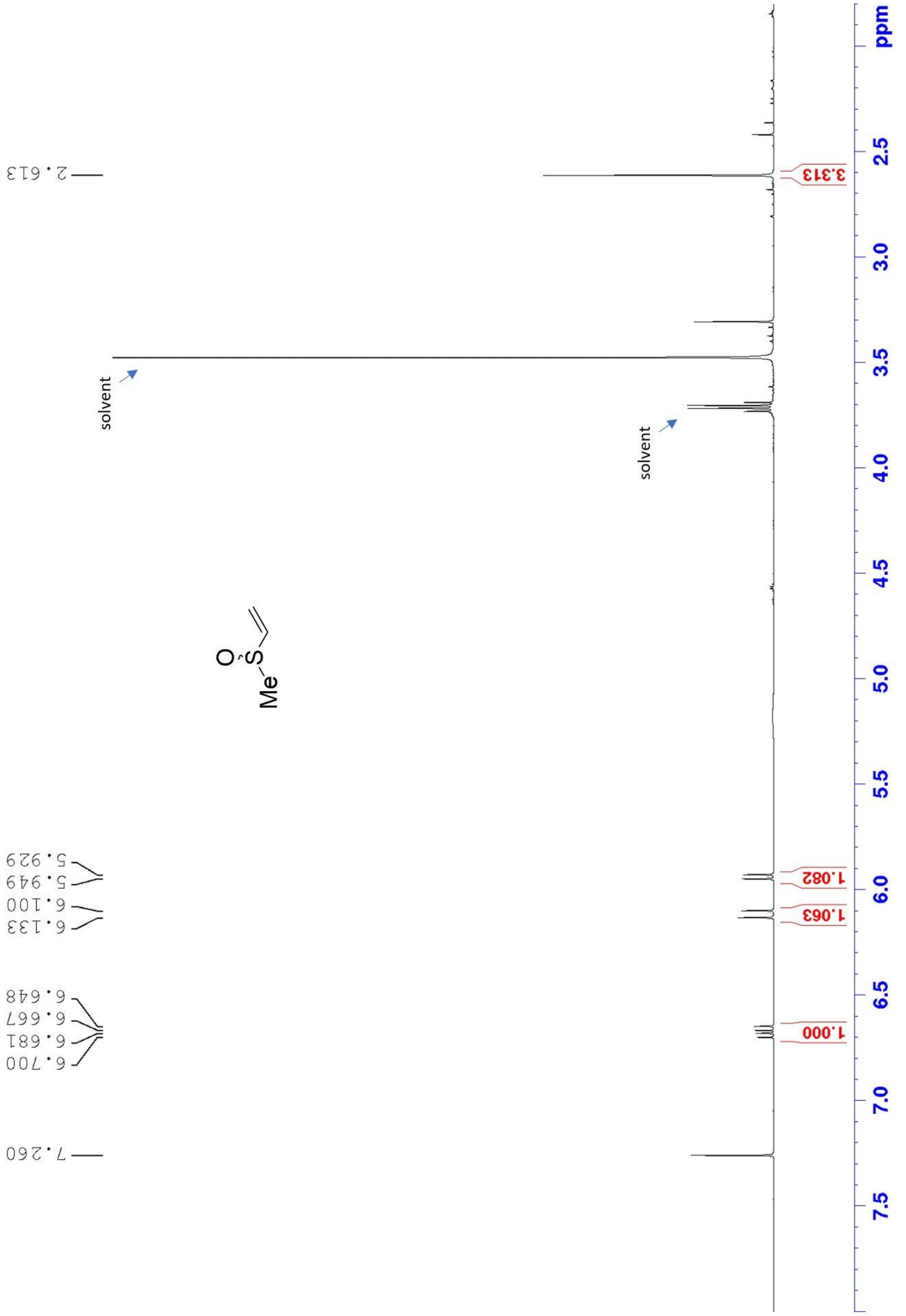

$^{13}C\{^1H\}$ NMR (125 MHz, $CDCl_3$)

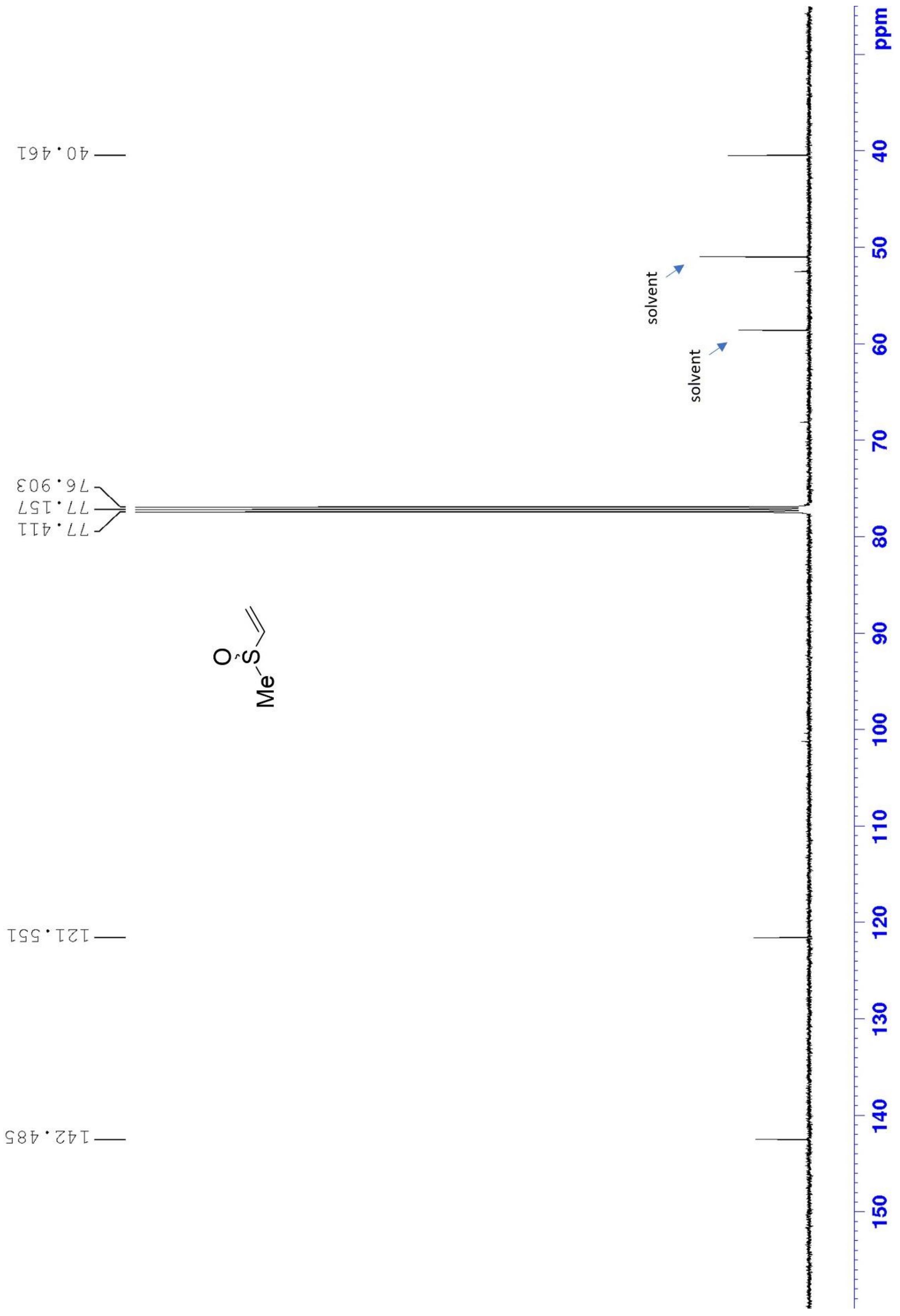

(R,R)-Bis[2-(p-tolylsulfinyl)ethyl] sulfide, **28(*R*,*R*)**

^{1}H NMR (500 MHz, $CDCl_3$)

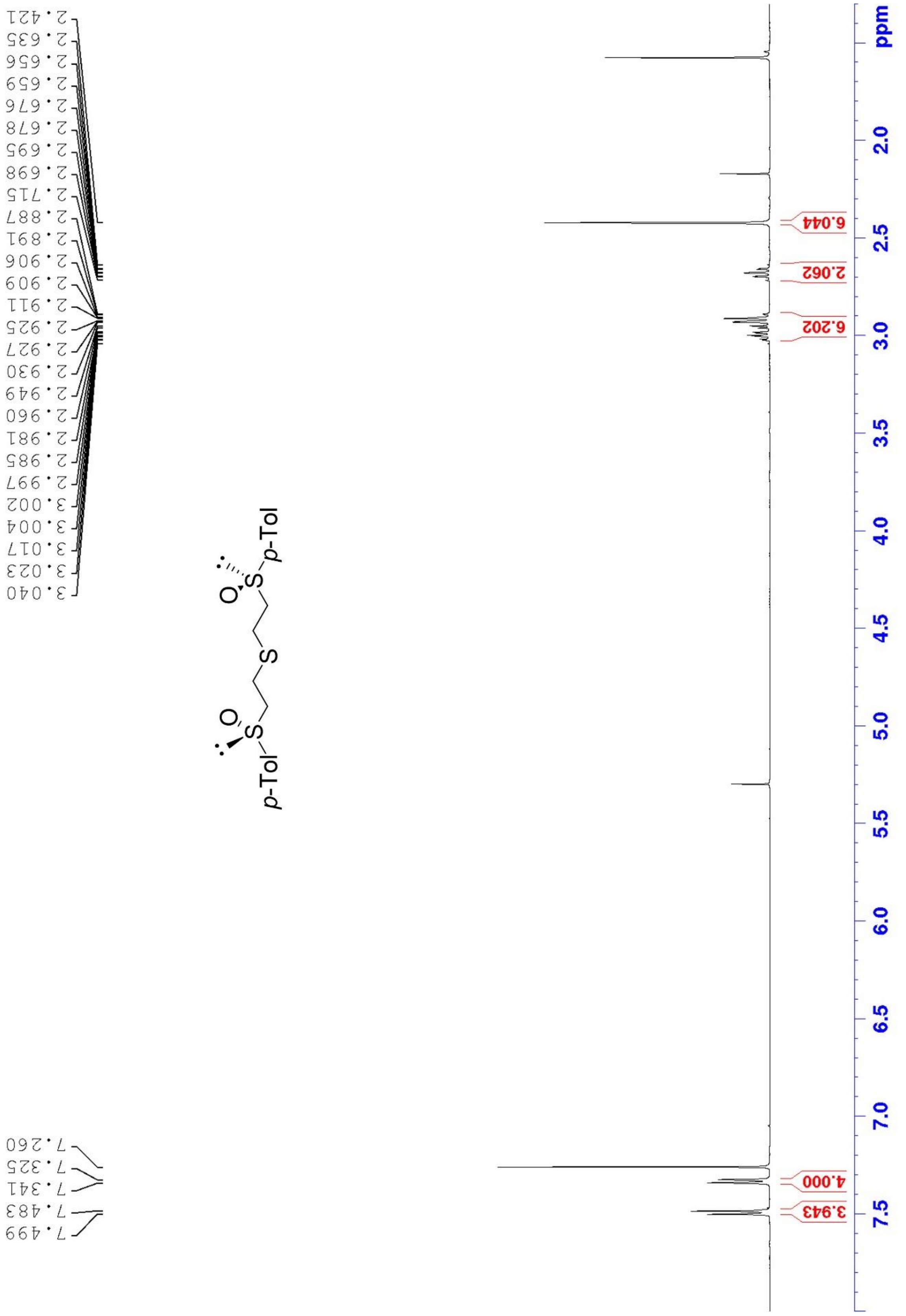

$^{13}C\{^{1}H\}$ NMR (125 MHz, $CDCl_3$)

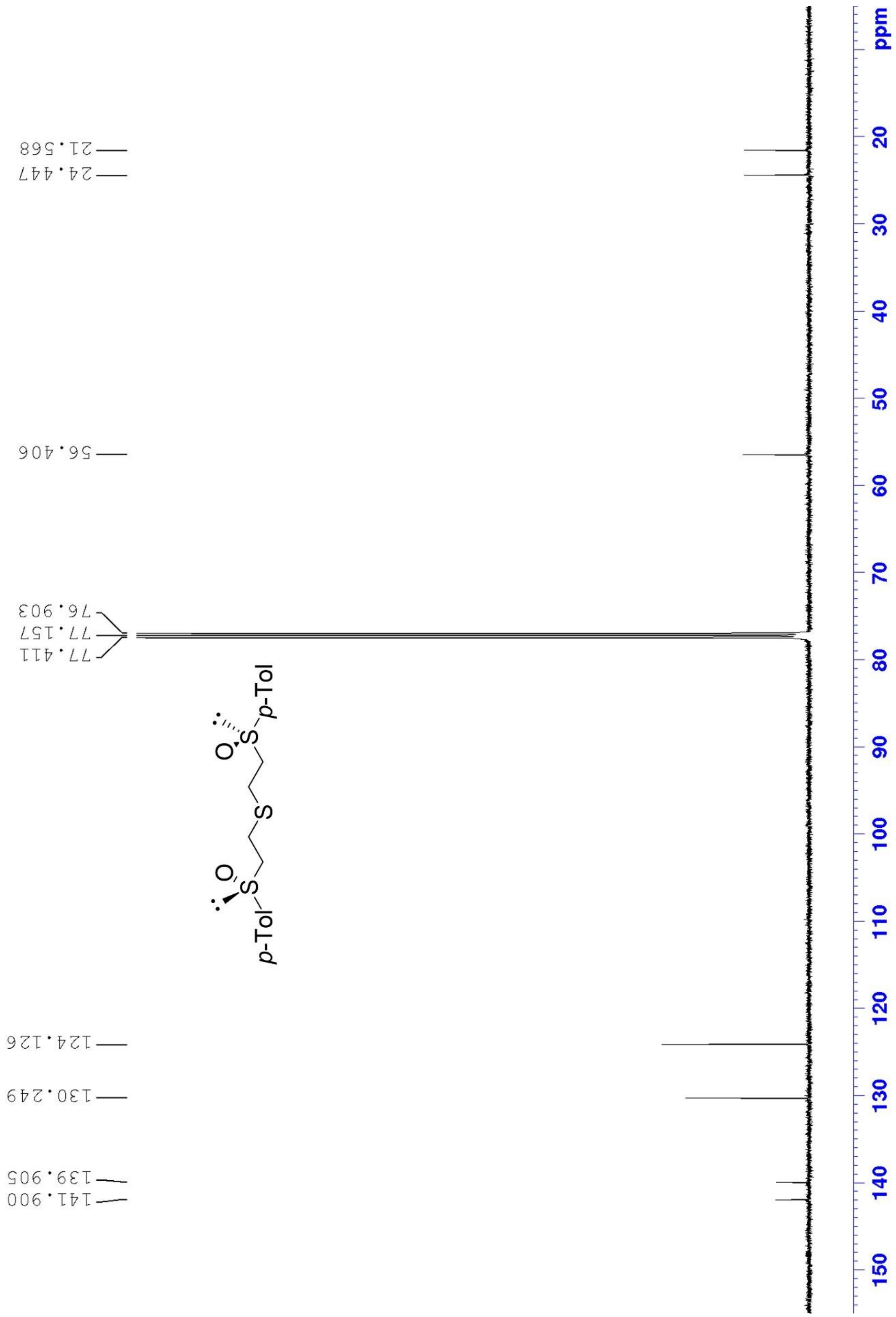

(S,S)-Bis[2-(p-tolylsulfinyl)ethyl] sulfide, **28(*S,S*)**

^{1}H NMR (500 MHz, $CDCl_3$)

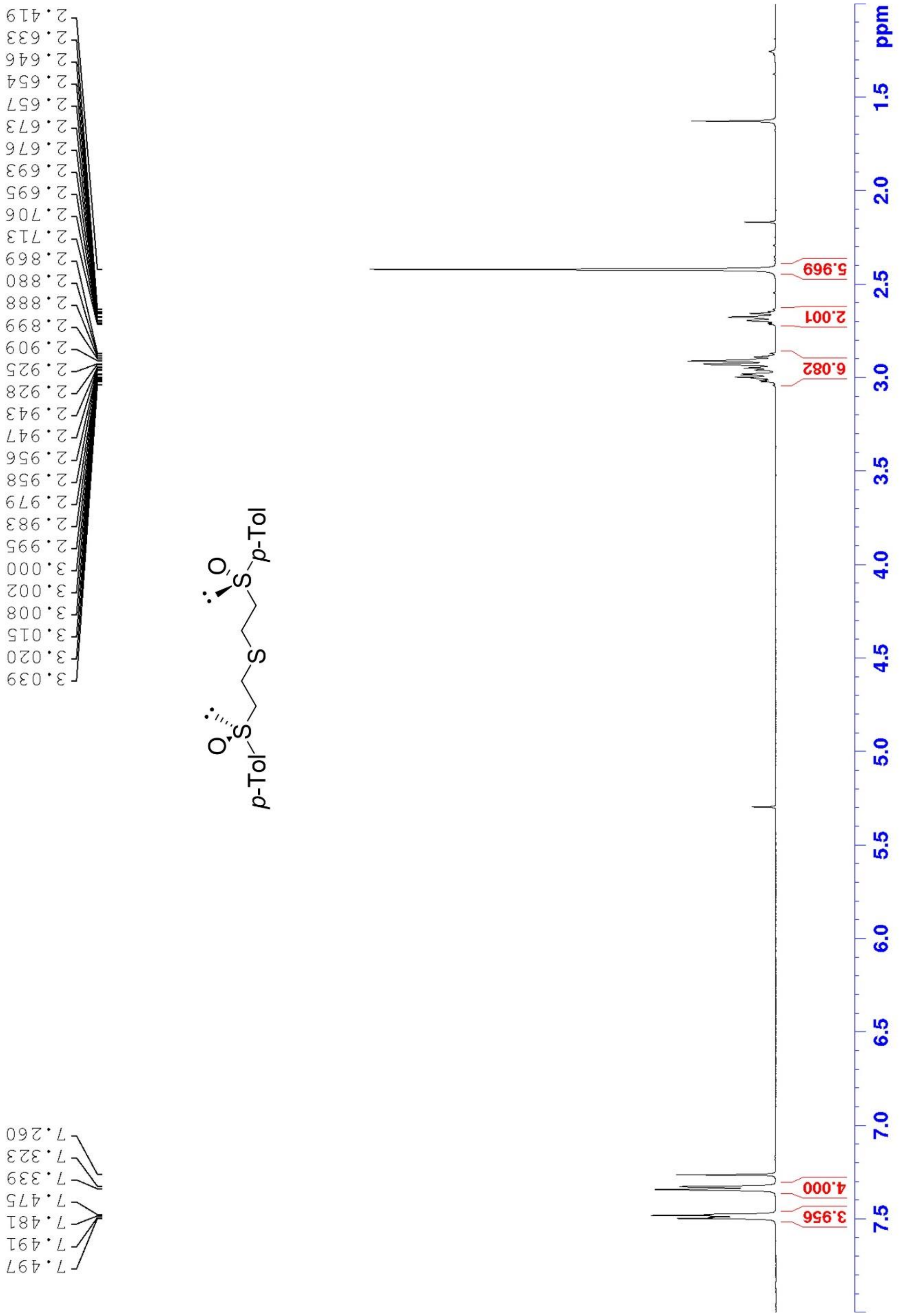

$^{13}C\{^1H\}$ NMR (125 MHz, $CDCl_3$)

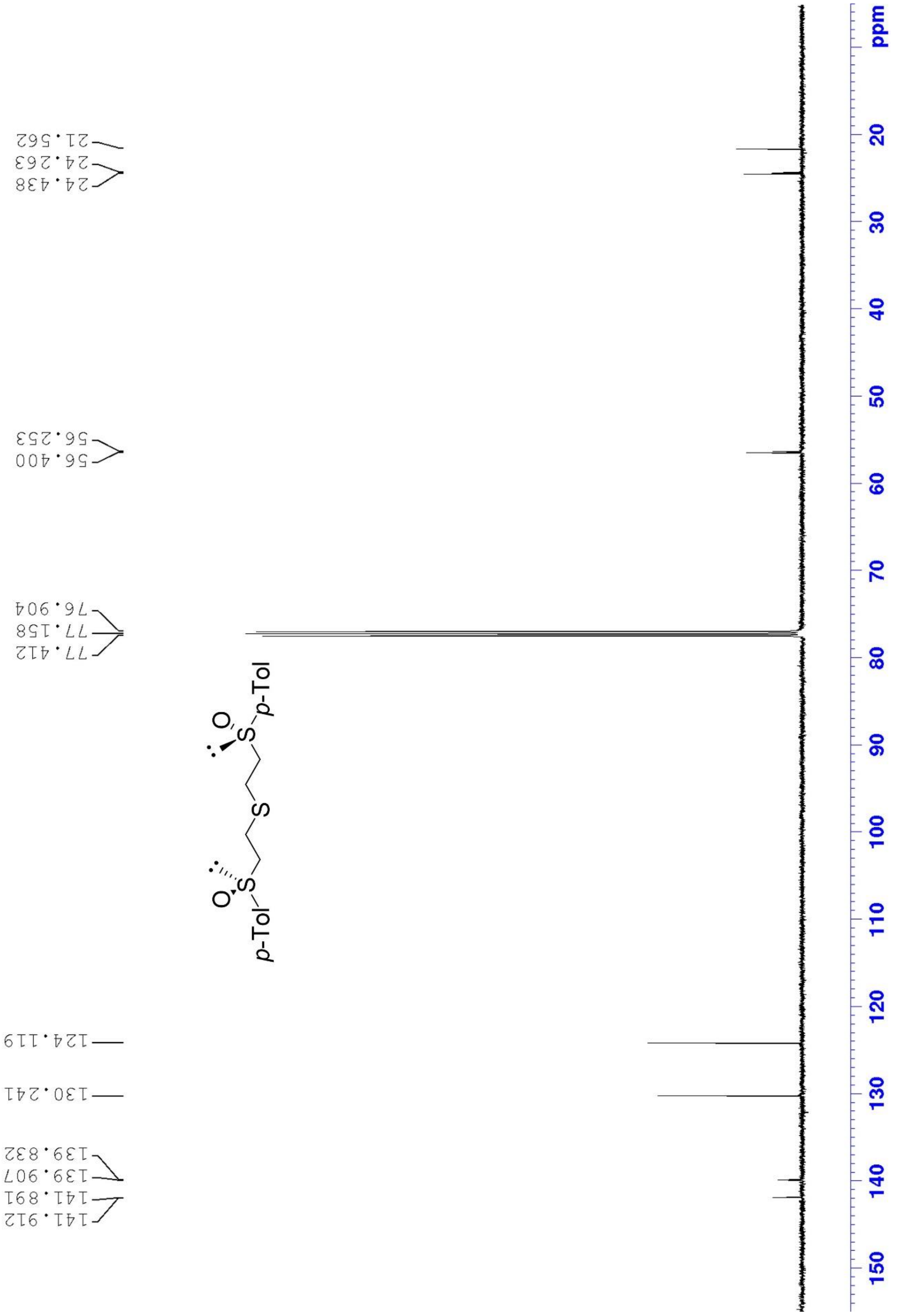

(S,S)/(R,R)/(S,R)-Bis[(2-(p-tolylsulfinyl)ethyl] sulfide, **28**(***rac*** **+** ***meso***)

^{1}H NMR (500 MHz, $CDCl_3$)

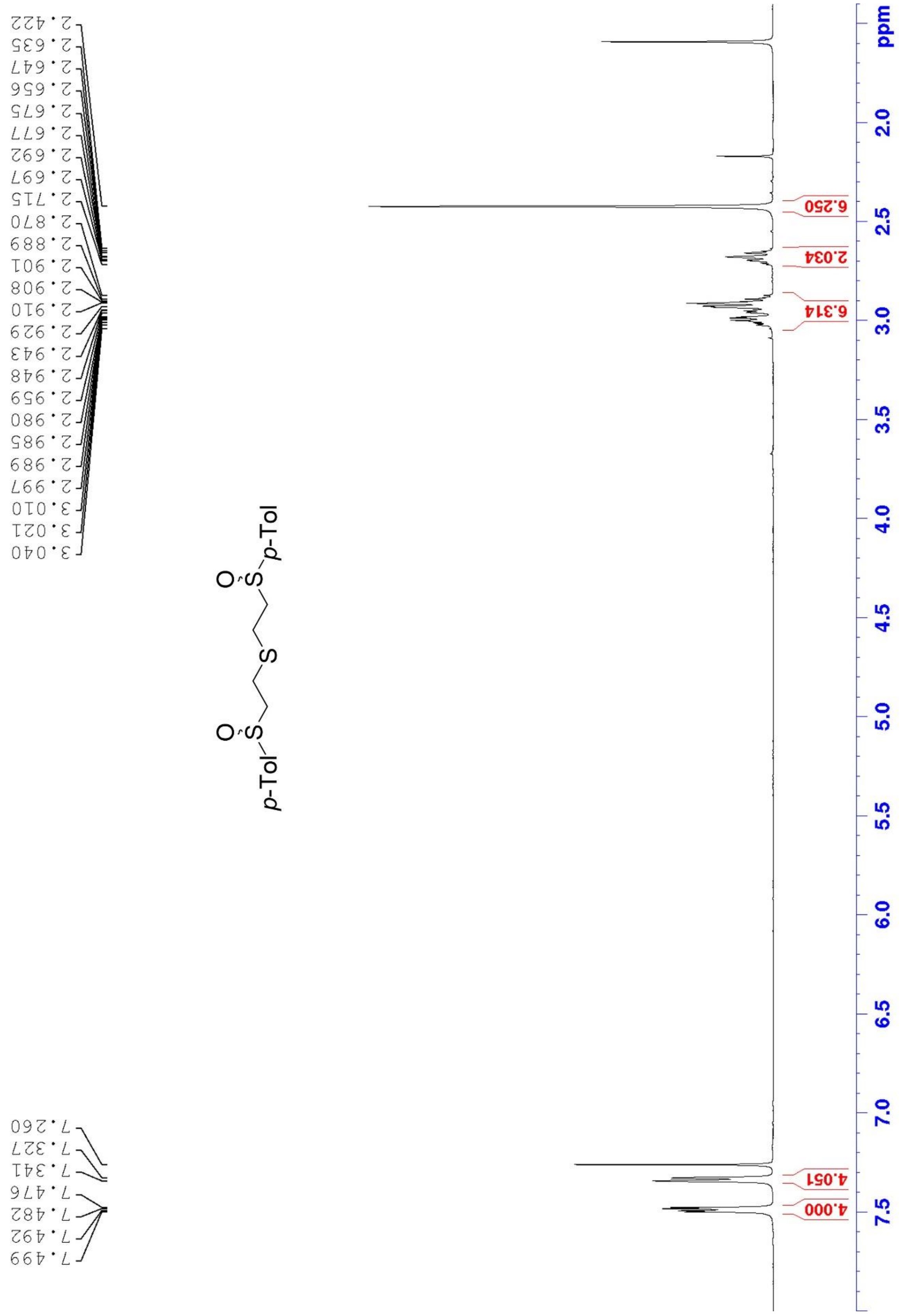

$^{13}C\{^1H\}$ NMR (125 MHz, $CDCl_3$)

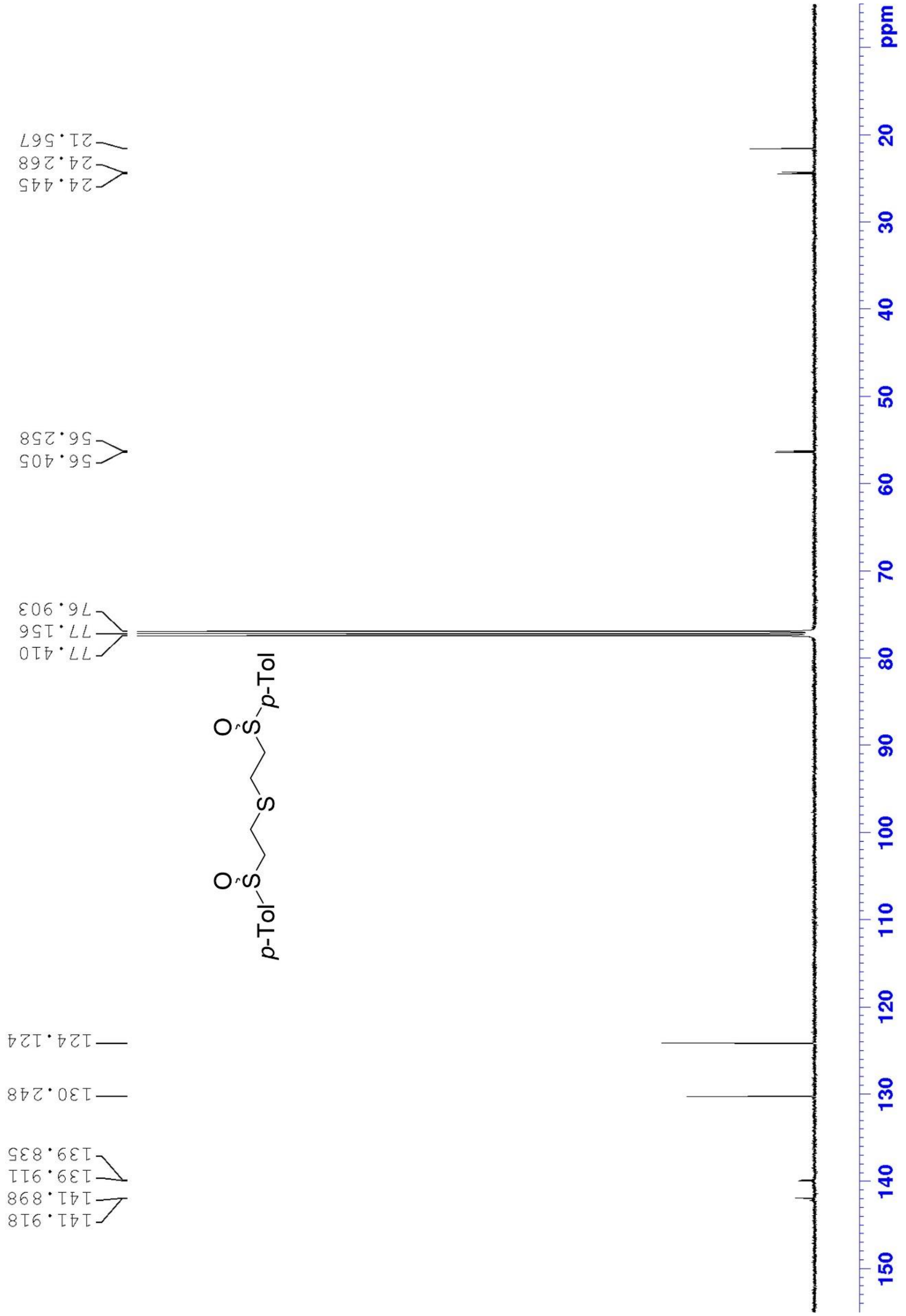

(R,R)-Bis[2-(tert-butylsulfinyl)ethyl] sulfide, **29(*R*,*R*)**

^{1}H NMR (500 MHz, MeOD)

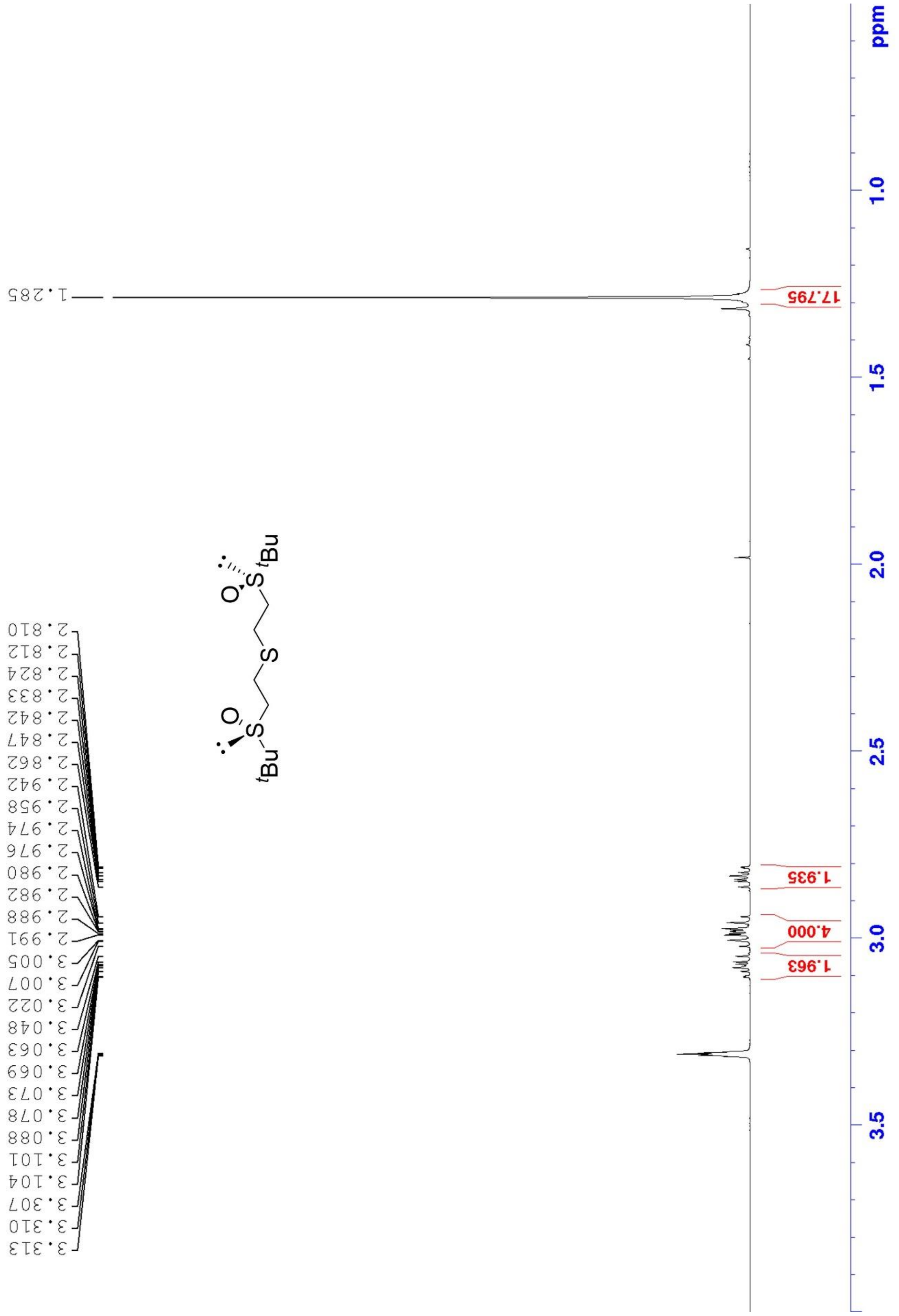

$^{13}C\{^{1}H\}$ NMR (125 MHz, MeOD)

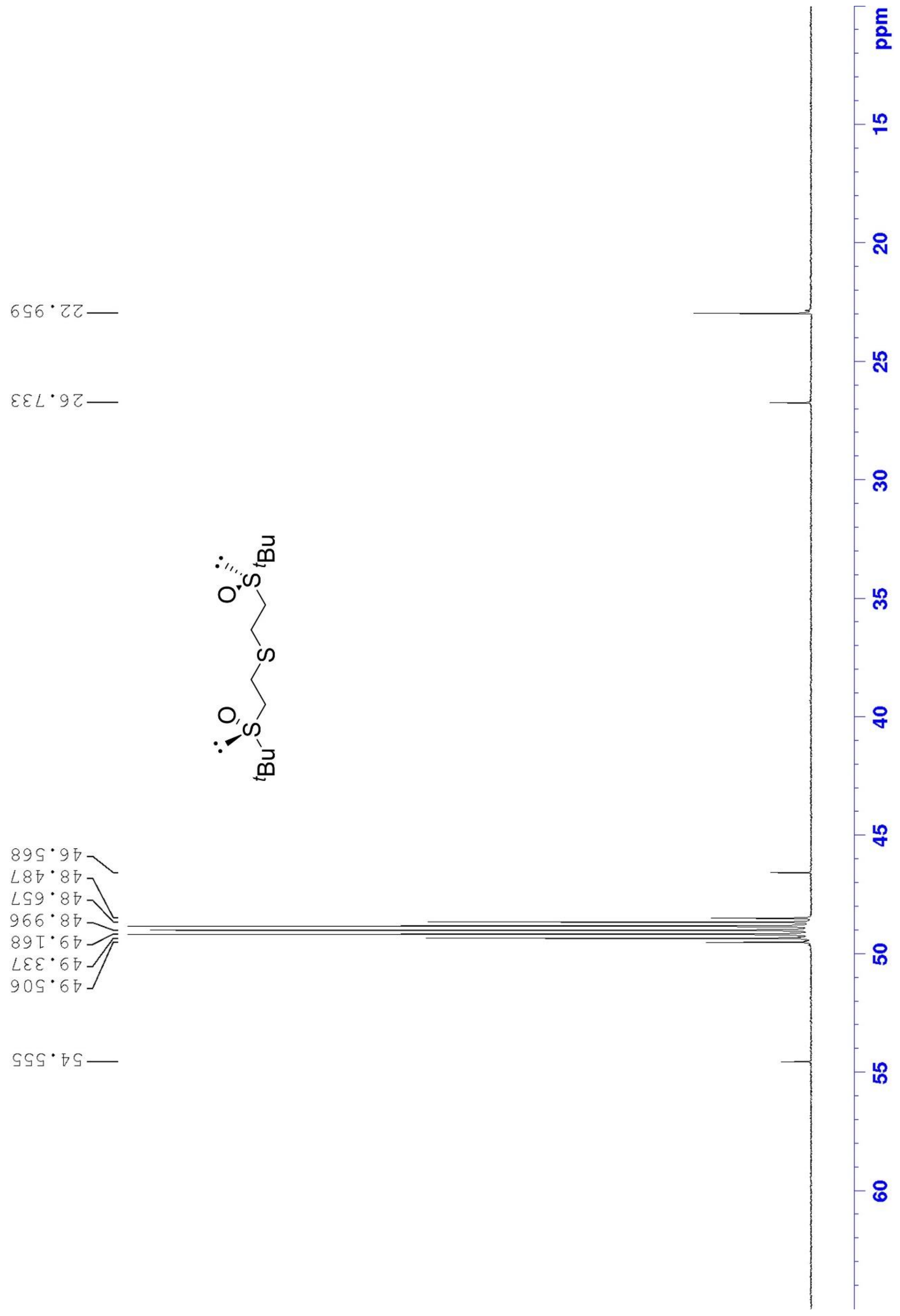

(S,S)-Bis[2-(tert-butylsulfinyl)ethyl] sulfide, **29(*S,S*)**

^{1}H NMR (500 MHz, MeOD)

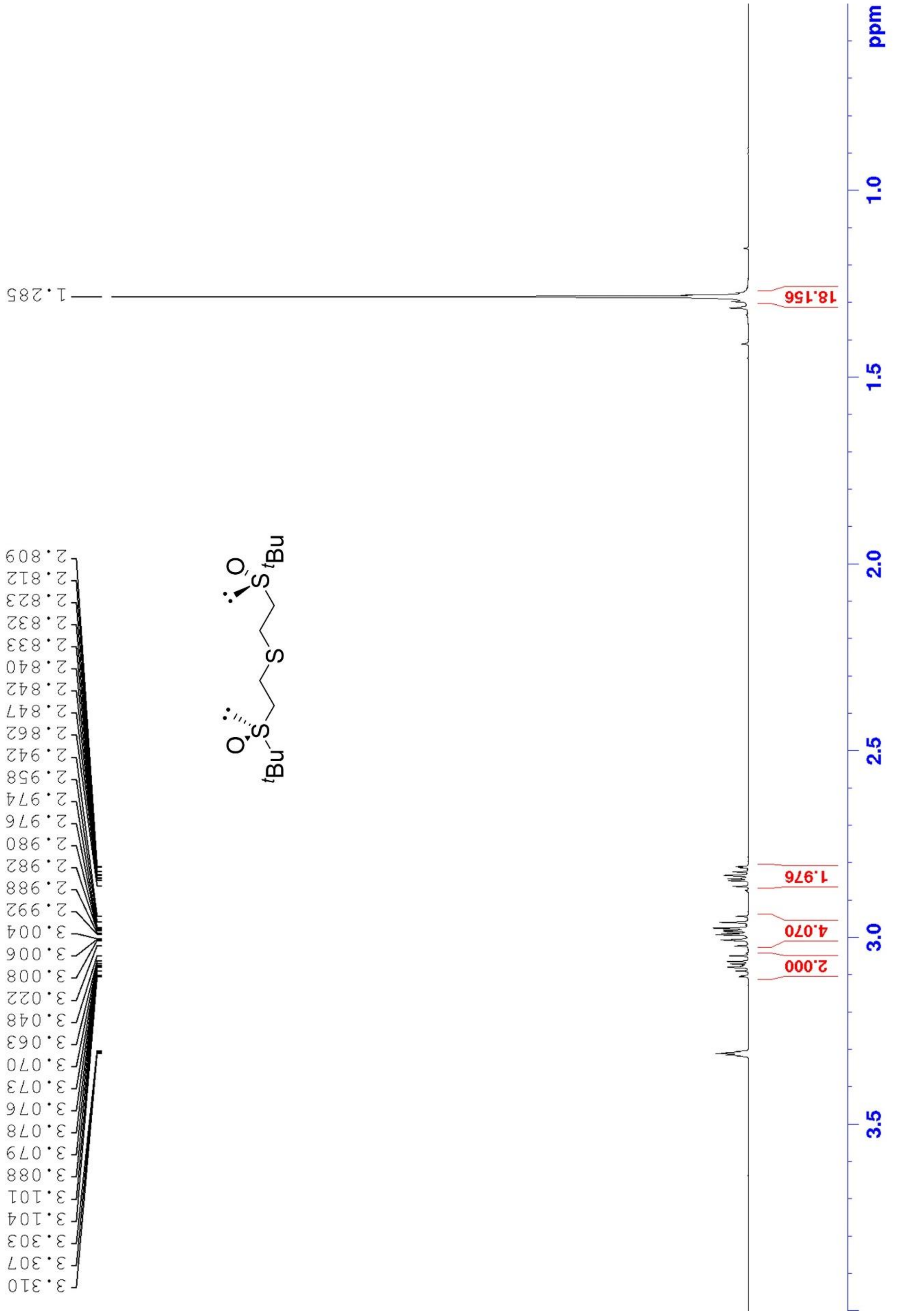

$^{13}C\{^{1}H\}$ NMR (125 MHz, MeOD)

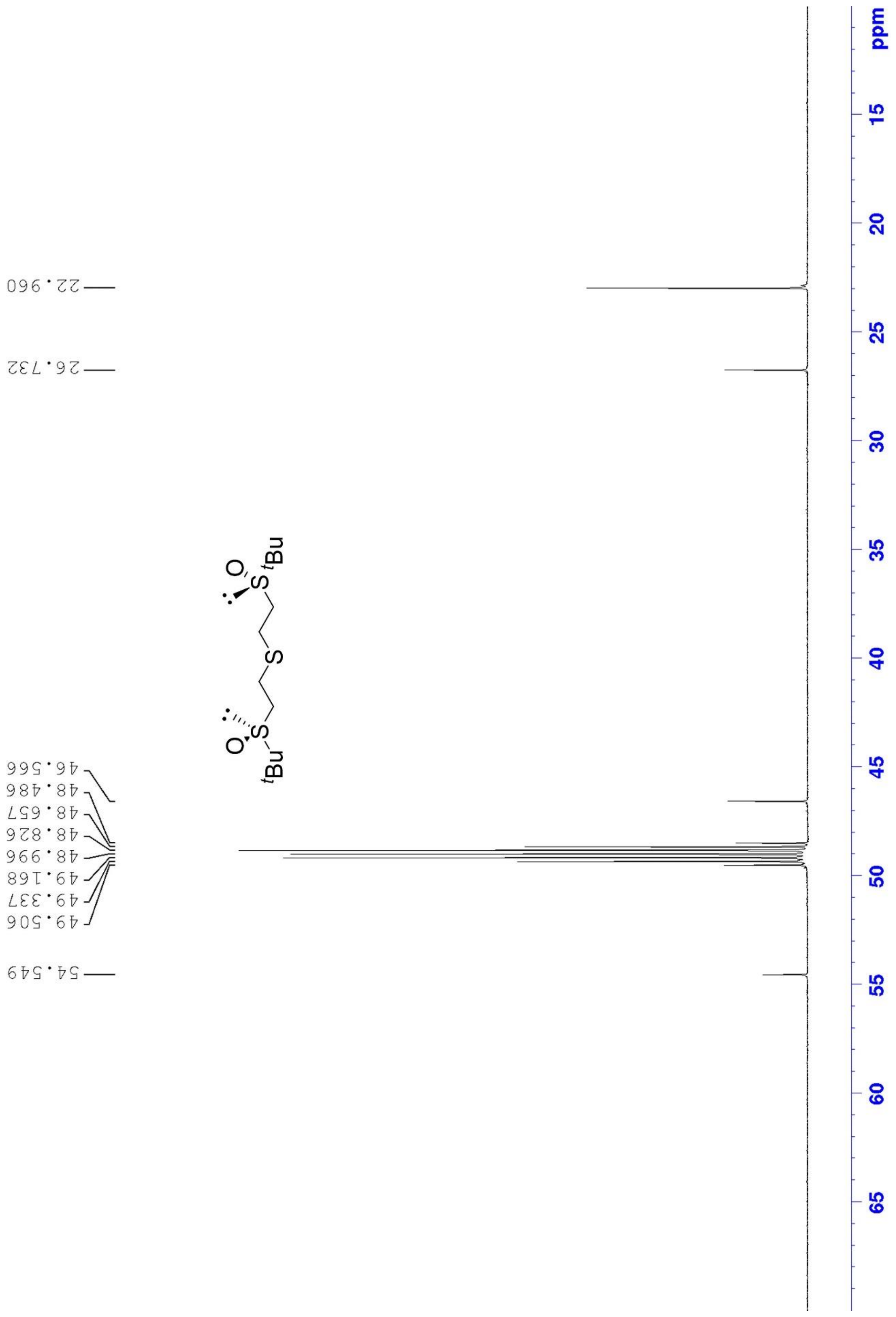

(S,S)/(R,R)/(S,R)-Bis[2-(tert-butylsulfinyl)ethyl] sulfide, **29(*rac* + *meso*)**

^{1}H NMR (500 MHz, MeOD)

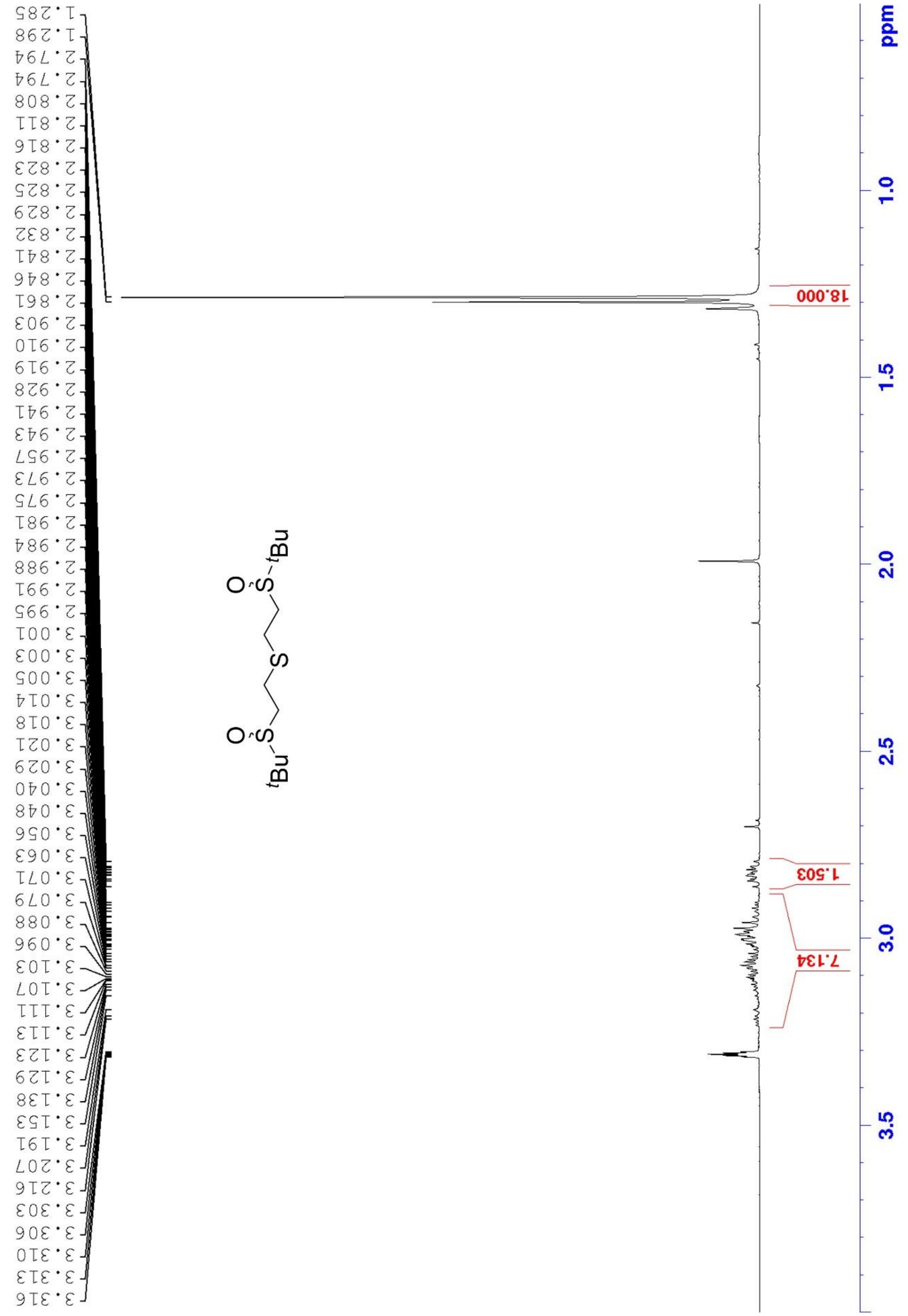

$^{13}C\{^{1}H\}$ NMR (125 MHz, MeOD)

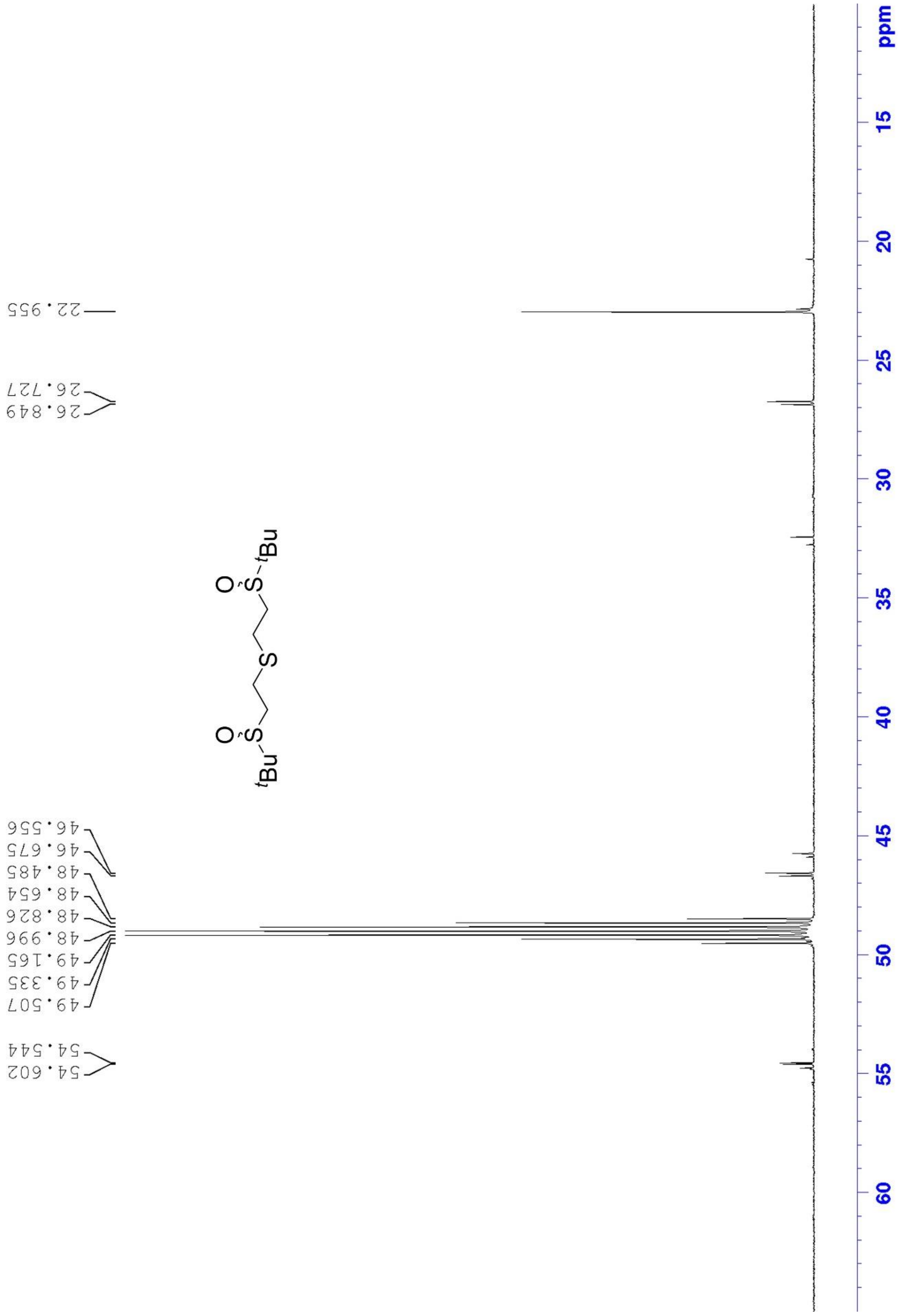

(S,S)-Bis[2-(methylsulfinyl)ethyl] sulfide, **30(*S*,*S*)**

^{1}H NMR (500 MHz, DMSO-d$_6$)

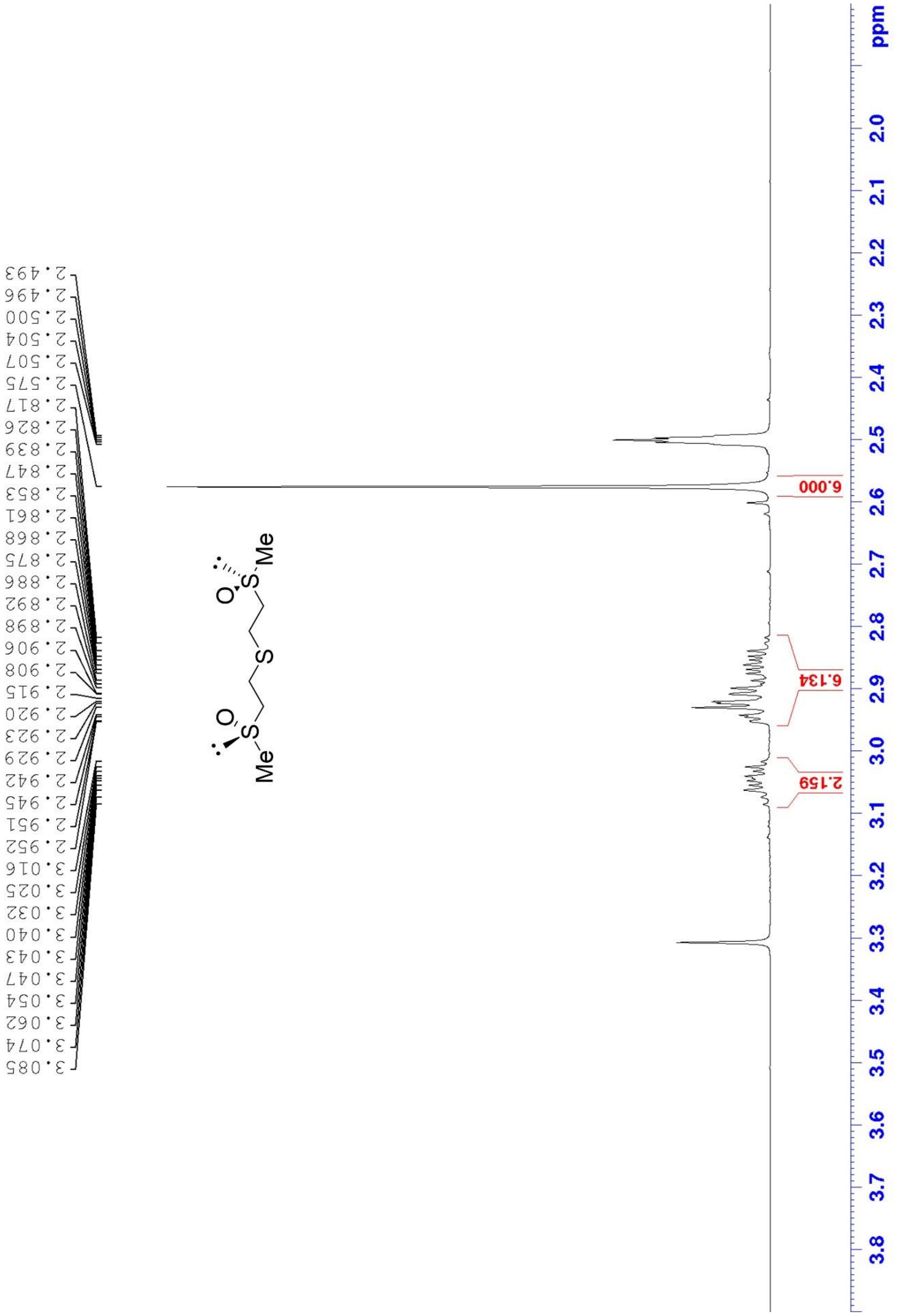

$^{13}C\{^{1}H\}$ NMR (125 MHz, DMSO-d_6)

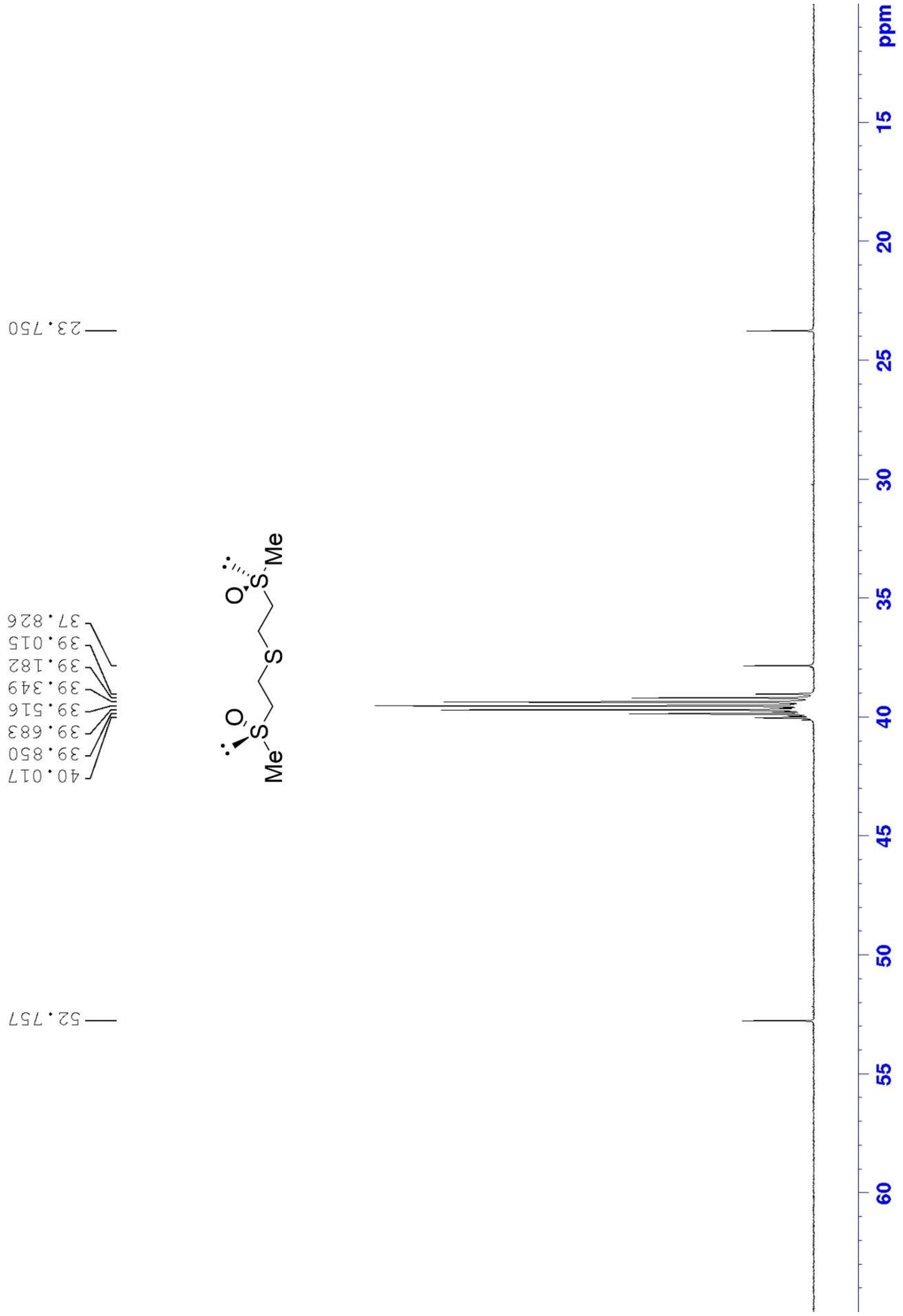

(S,S)-Bis[2-(methylsulfinyl)ethyl] sulfide, **30(*R*,*R*)**

^{1}H NMR (500 MHz, DMSO-d_6)

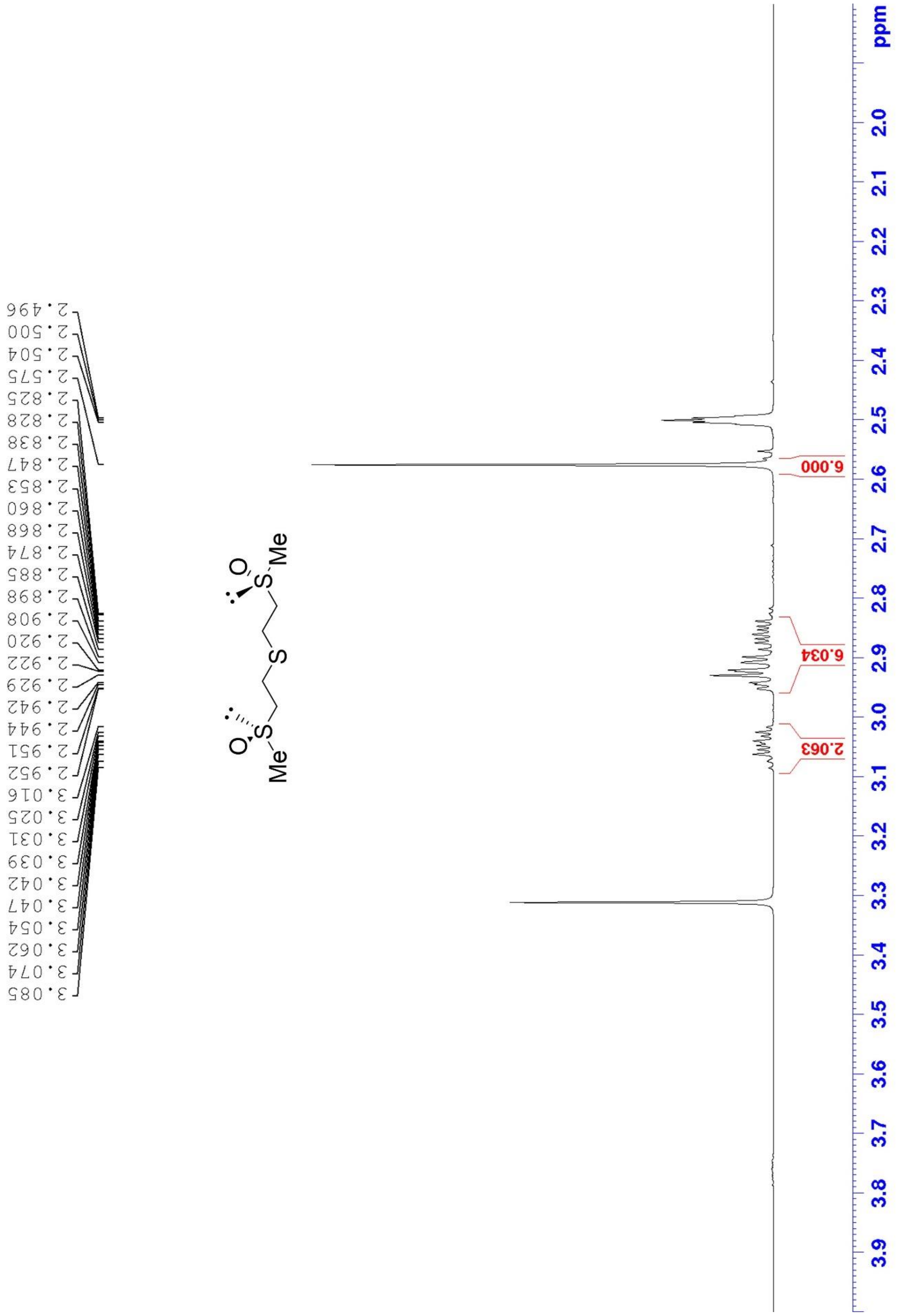

$^{13}C\{^{1}H\}$ NMR (125 MHz, DMSO-d_6)

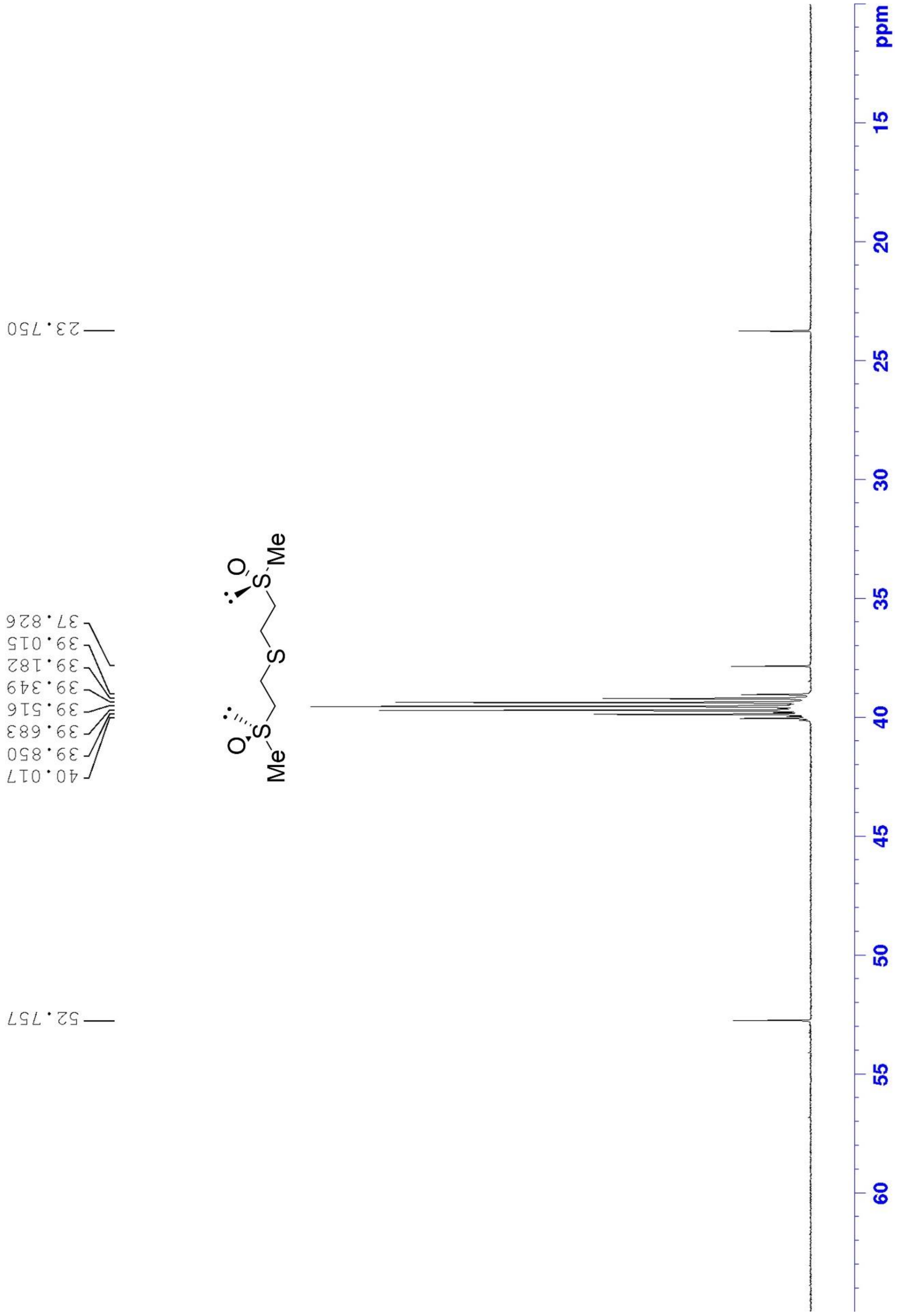

(S,S)/(R,R)/(S,R)-Bis[2-(methylsulfinyl)ethyl] sulfide, **30(*rac* + *meso*)**

^{1}H NMR (500 MHz, DMSO-d$_6$)

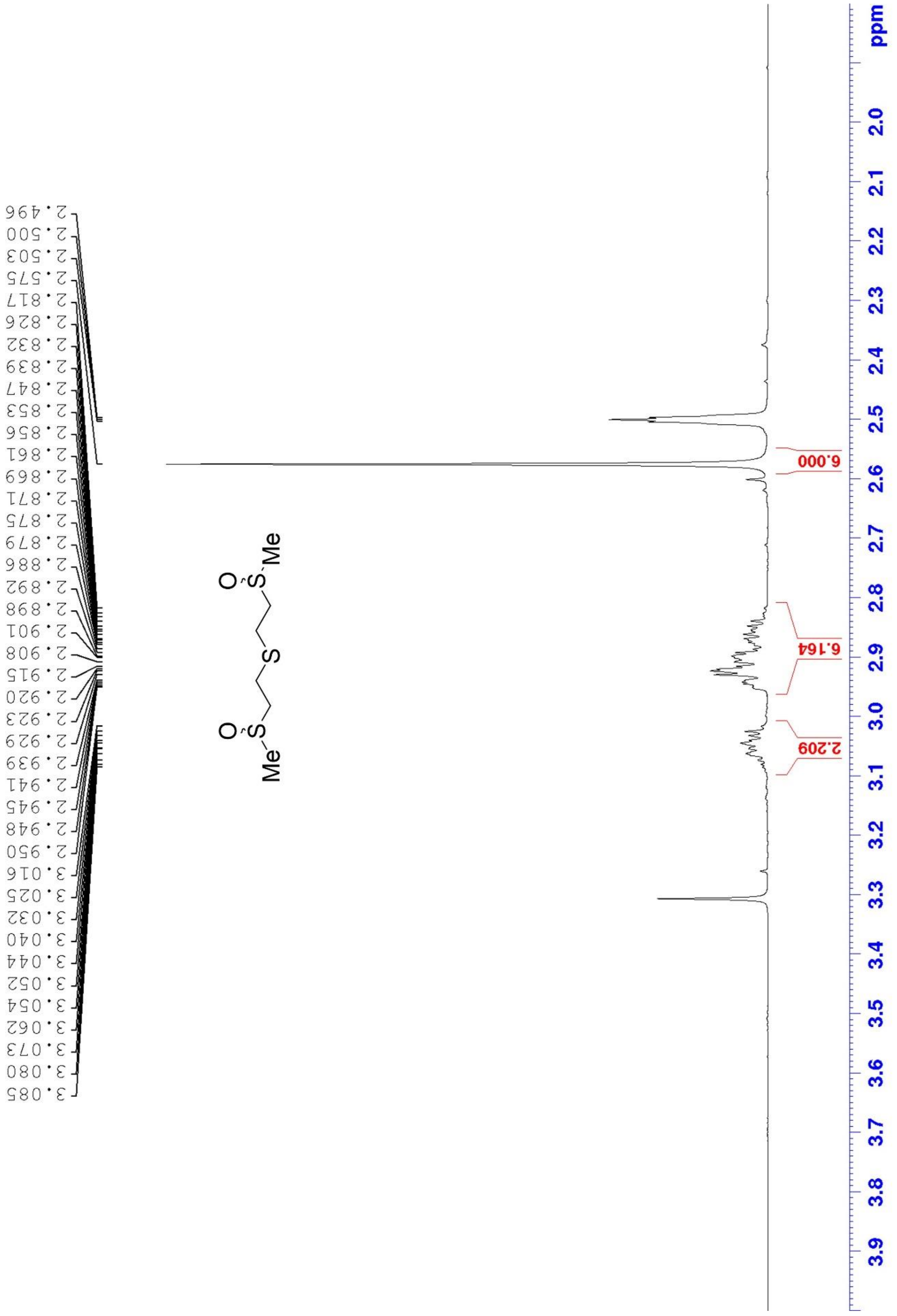

$^{13}C\{^1H\}$ NMR (125 MHz, DMSO-d_6)

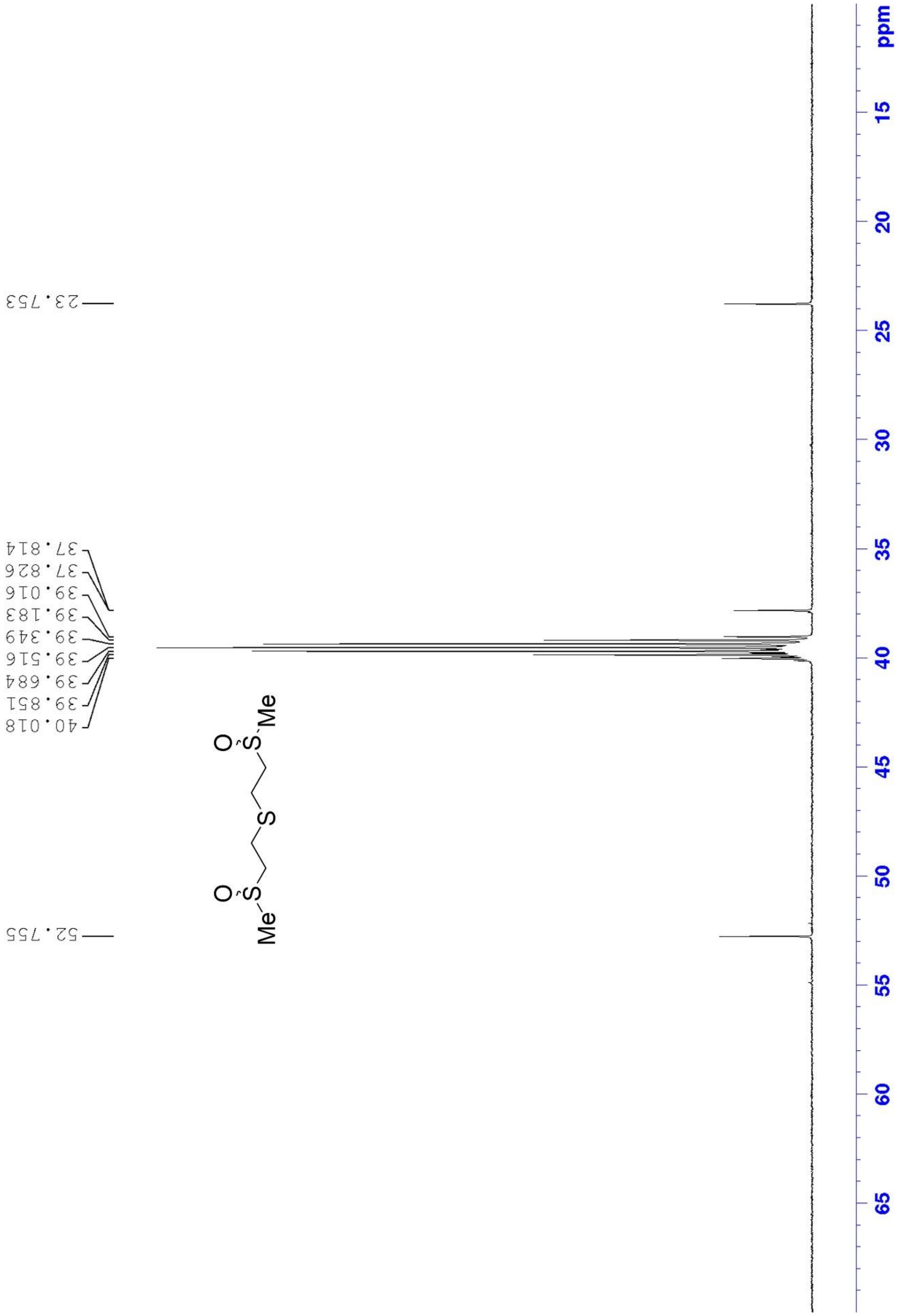

(R,R)-Bis[2-(p-tolylsulfinyl)ethyl] sulfoxide, **33(*R*,*R*)**

^{1}H NMR (500 MHz, MeOD)

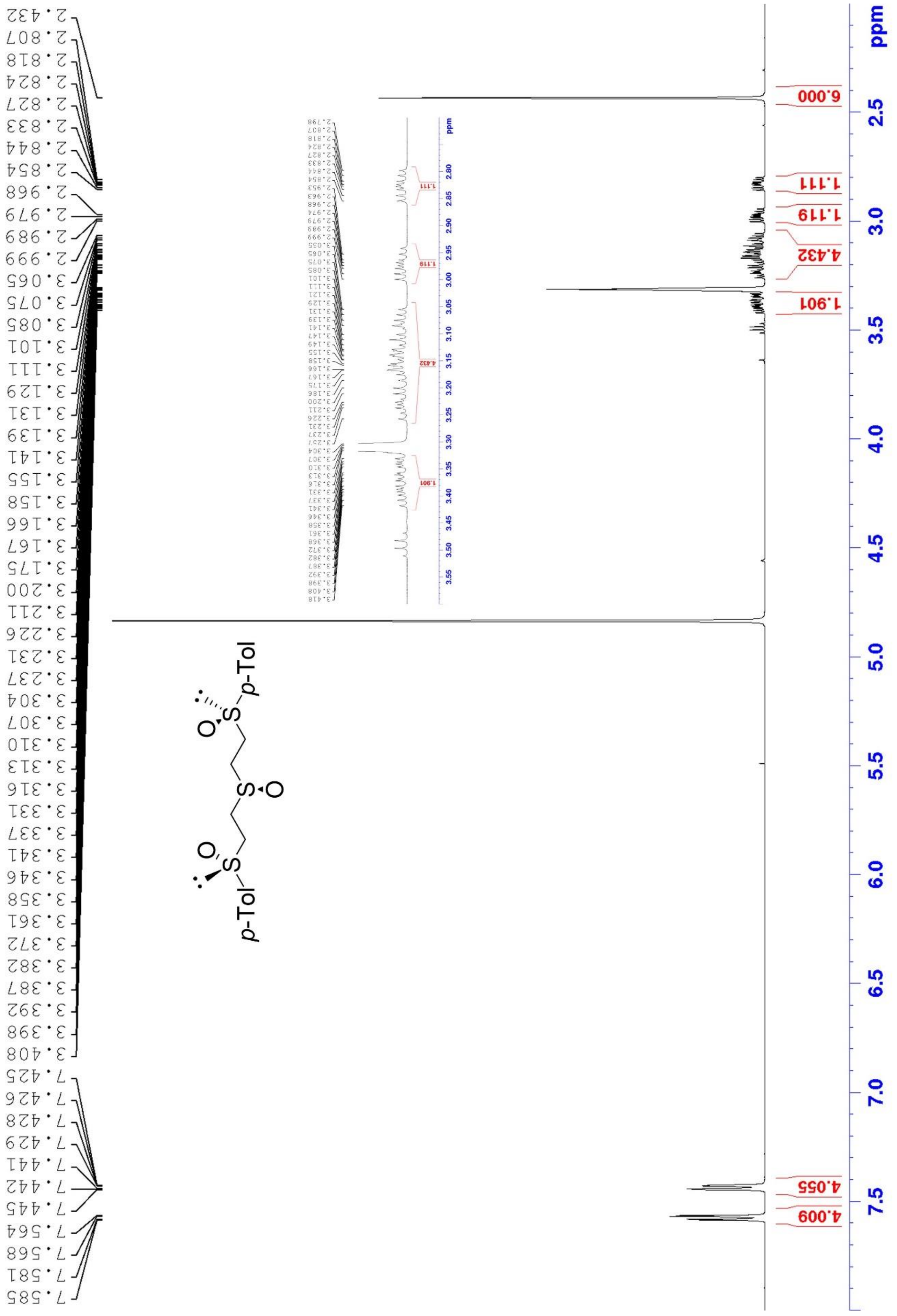

$^{13}C\{^{1}H\}$ NMR (125 MHz, MeOD)

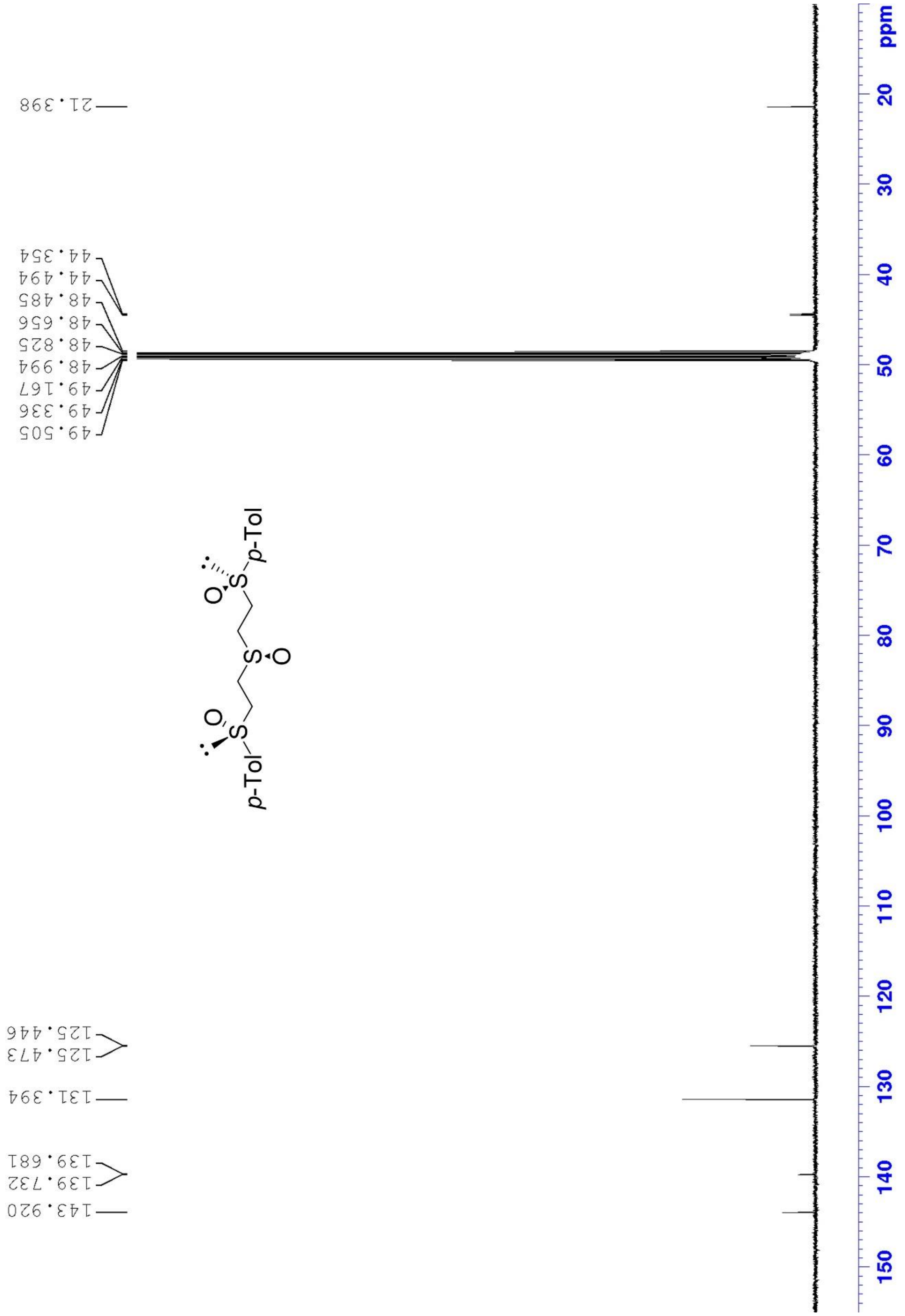

(R,R)-Bis[2-(tert-butylsulfinyl)ethyl] sulfoxide, **34(*R*,*R*)**

^{1}H NMR (500 MHz, MeOD)

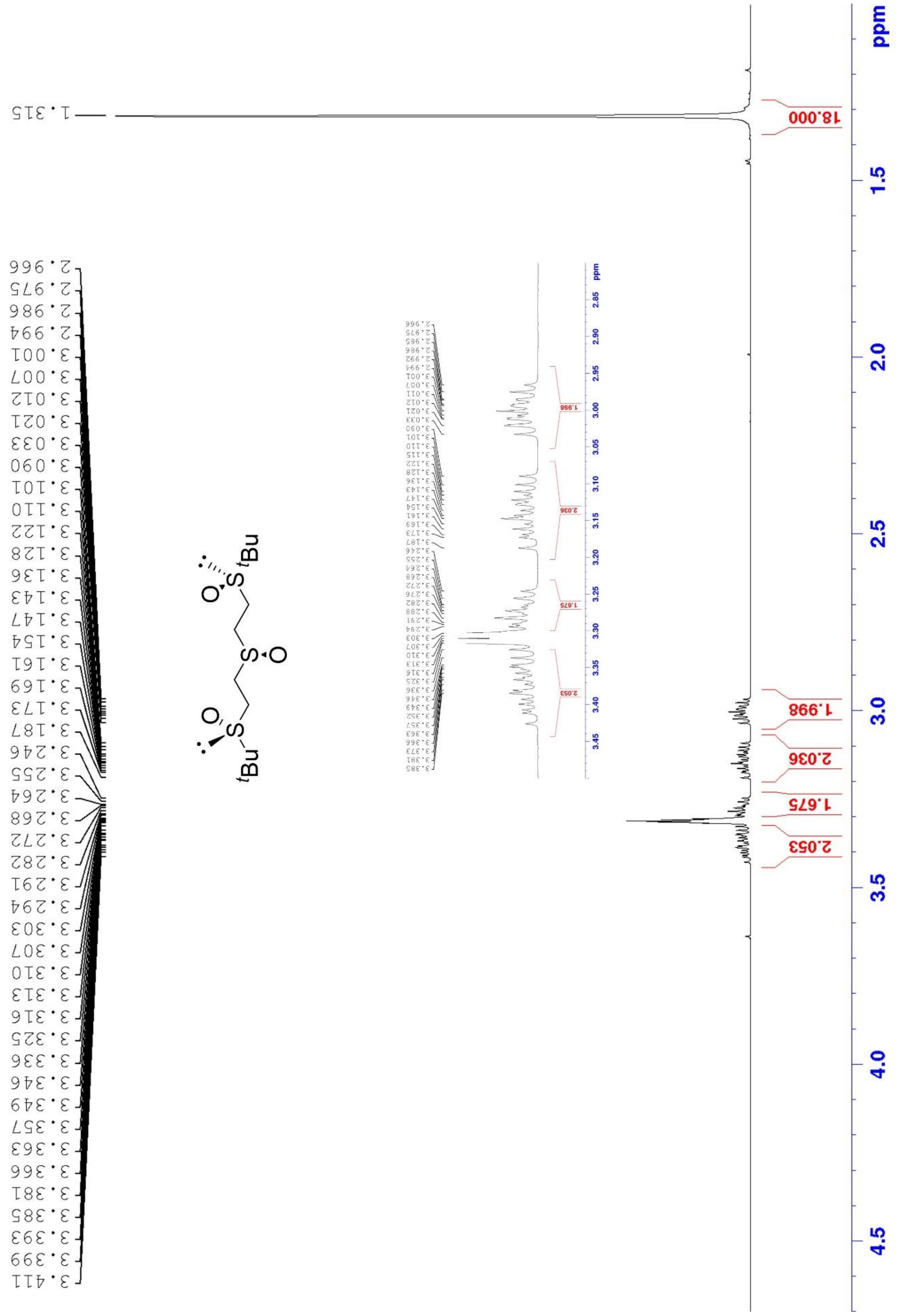

$^{13}C\{^1H\}$ NMR (125 MHz, MeOD)

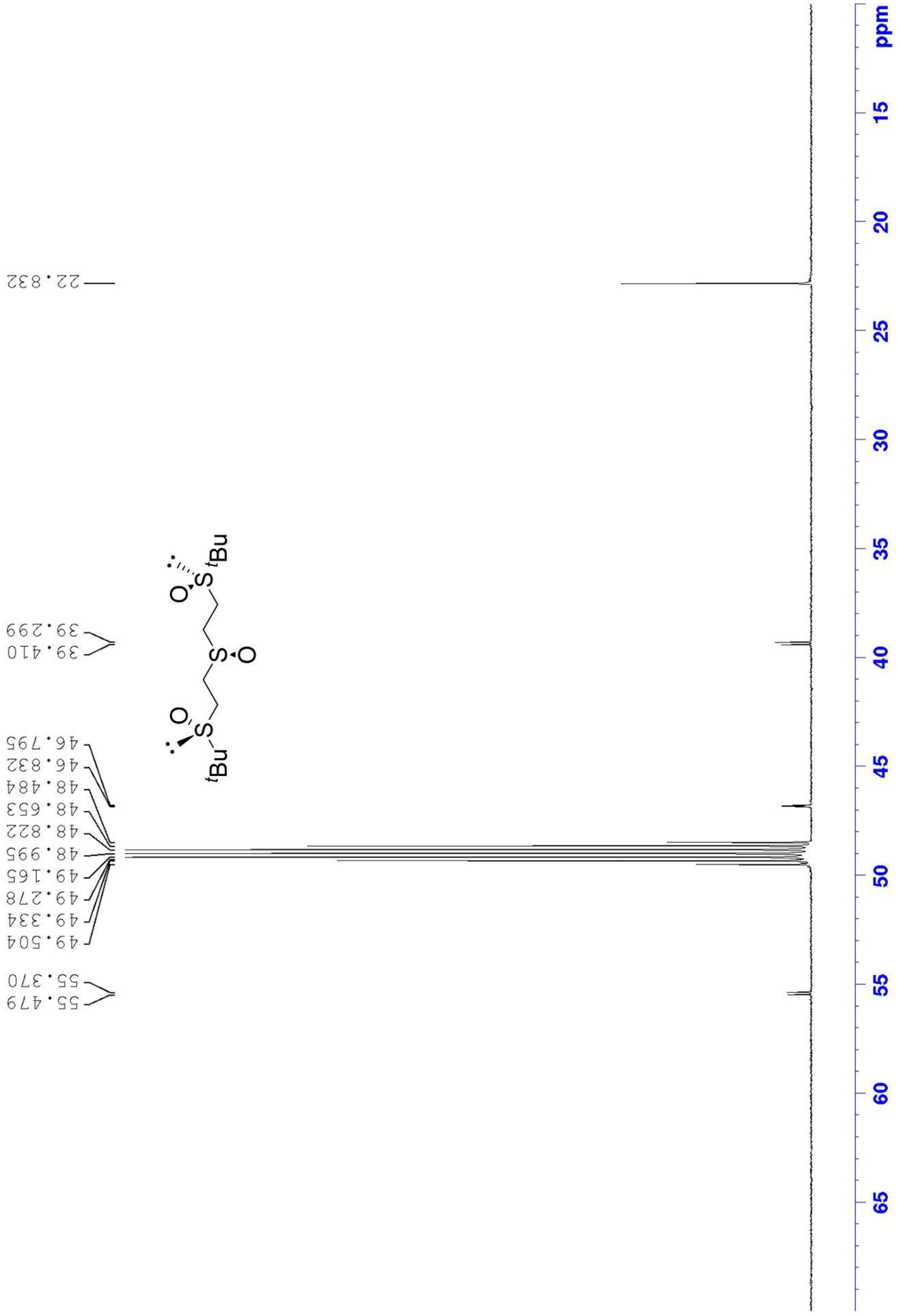

(S,S)-Bis[2-(tert-butylsulfinyl)ethyl] sulfoxide, **34(*S,S*)**

^{1}H NMR (500 MHz, MeOD)

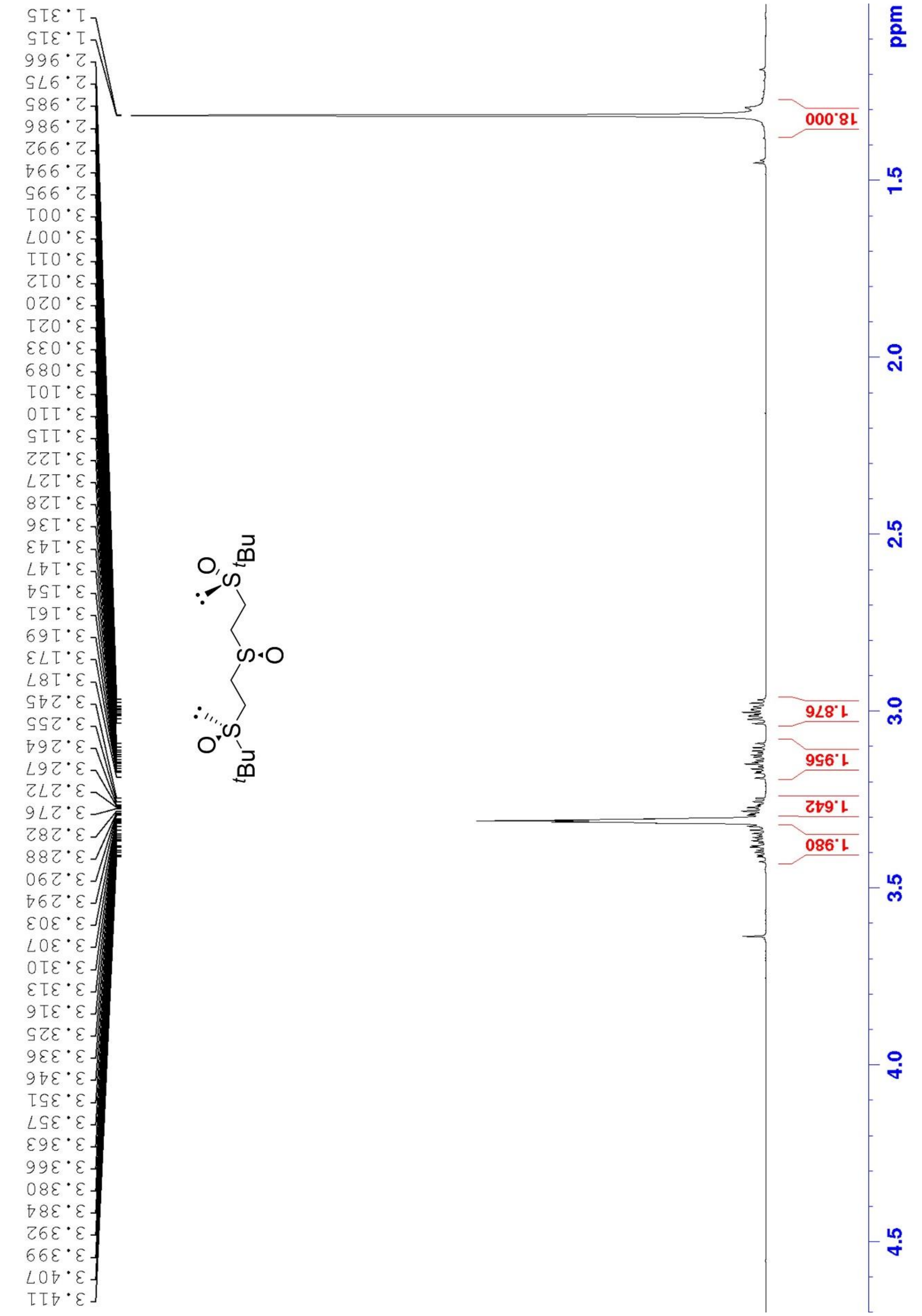

$^{13}C\{^{1}H\}$ NMR (125 MHz, MeOD)

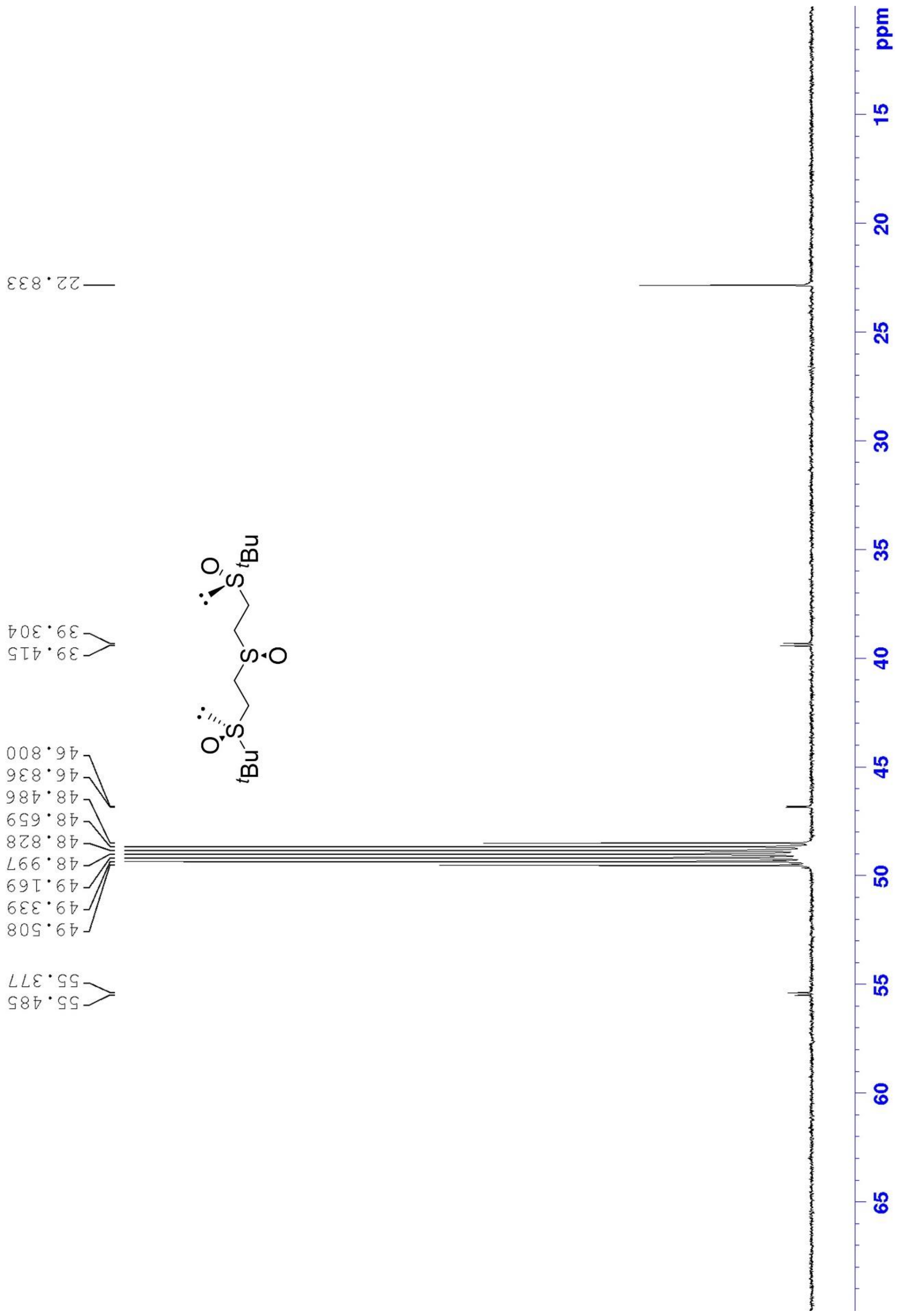

(S,S)-Bis[2-(methylsulfinyl)ethyl] sulfoxide, **35(*S*,*S*)**

^{1}H NMR (500 MHz, MeOD)

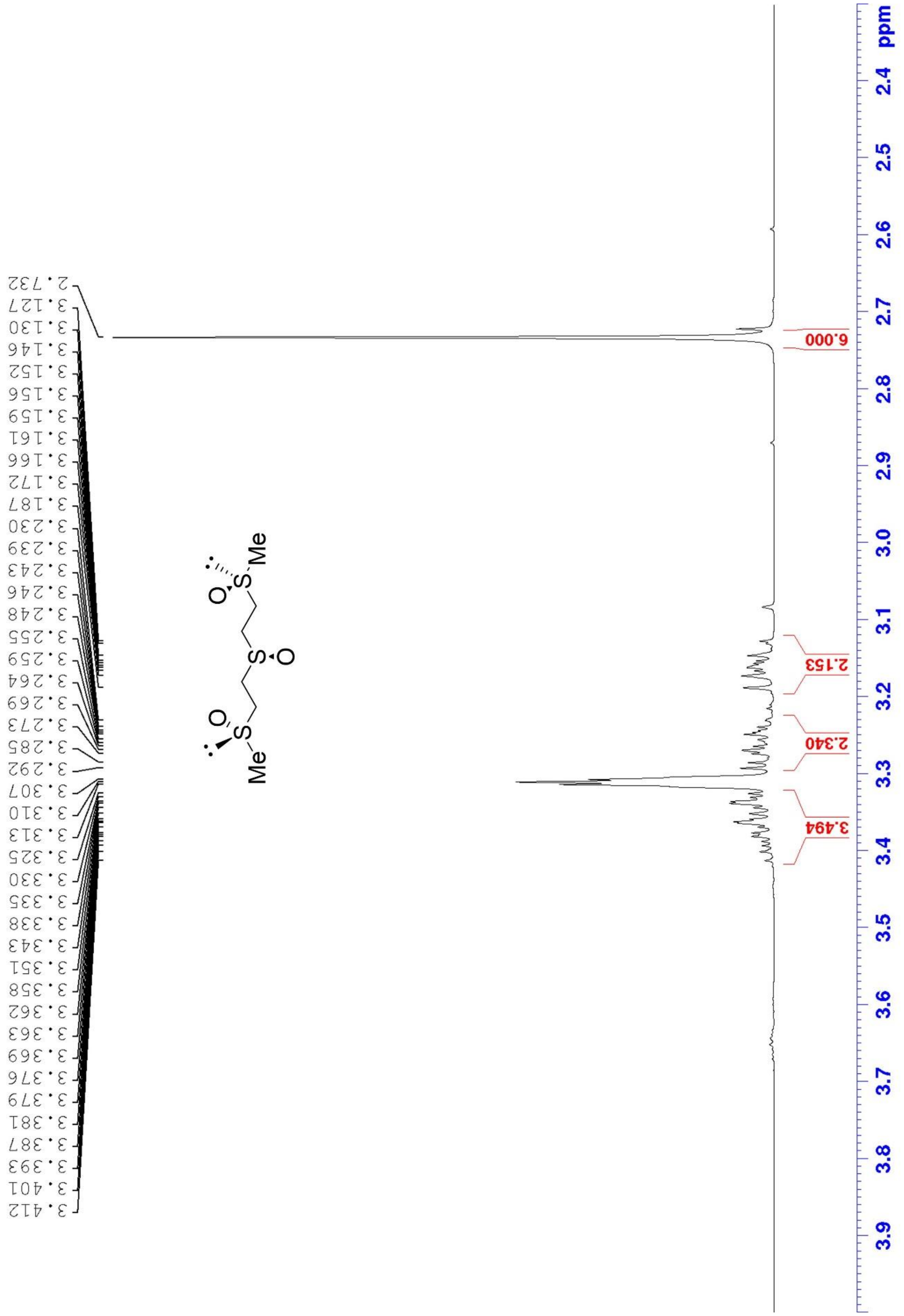

$^{13}C\{^1H\}$ NMR (125 MHz, MeOD)

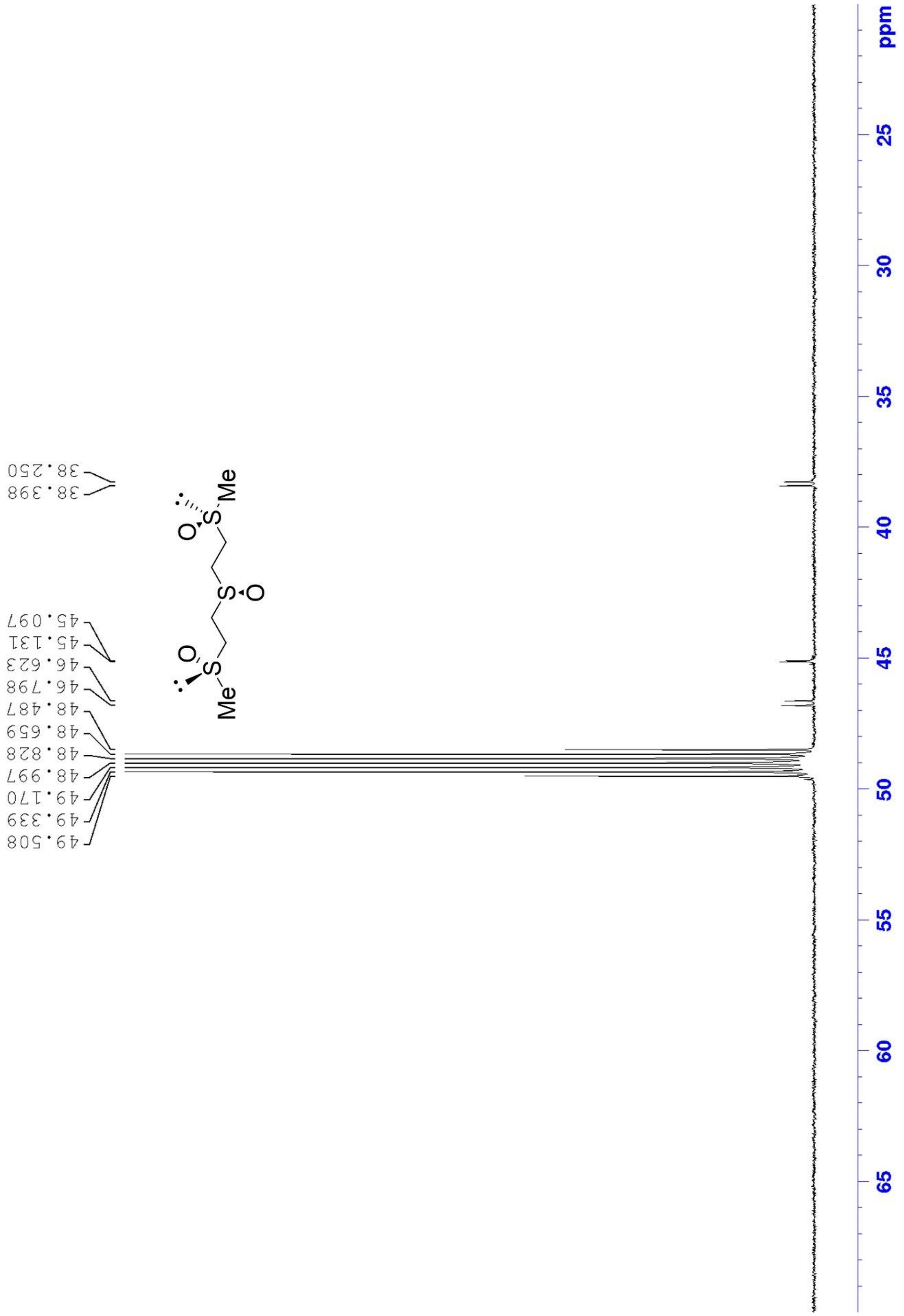

trans-Dichlorobis[(R,R)-1,3-bis(methylsulfinyl)propane]ruthenium (II), **36(*R,R*)**

^{1}H NMR (500 MHz, MeOD)

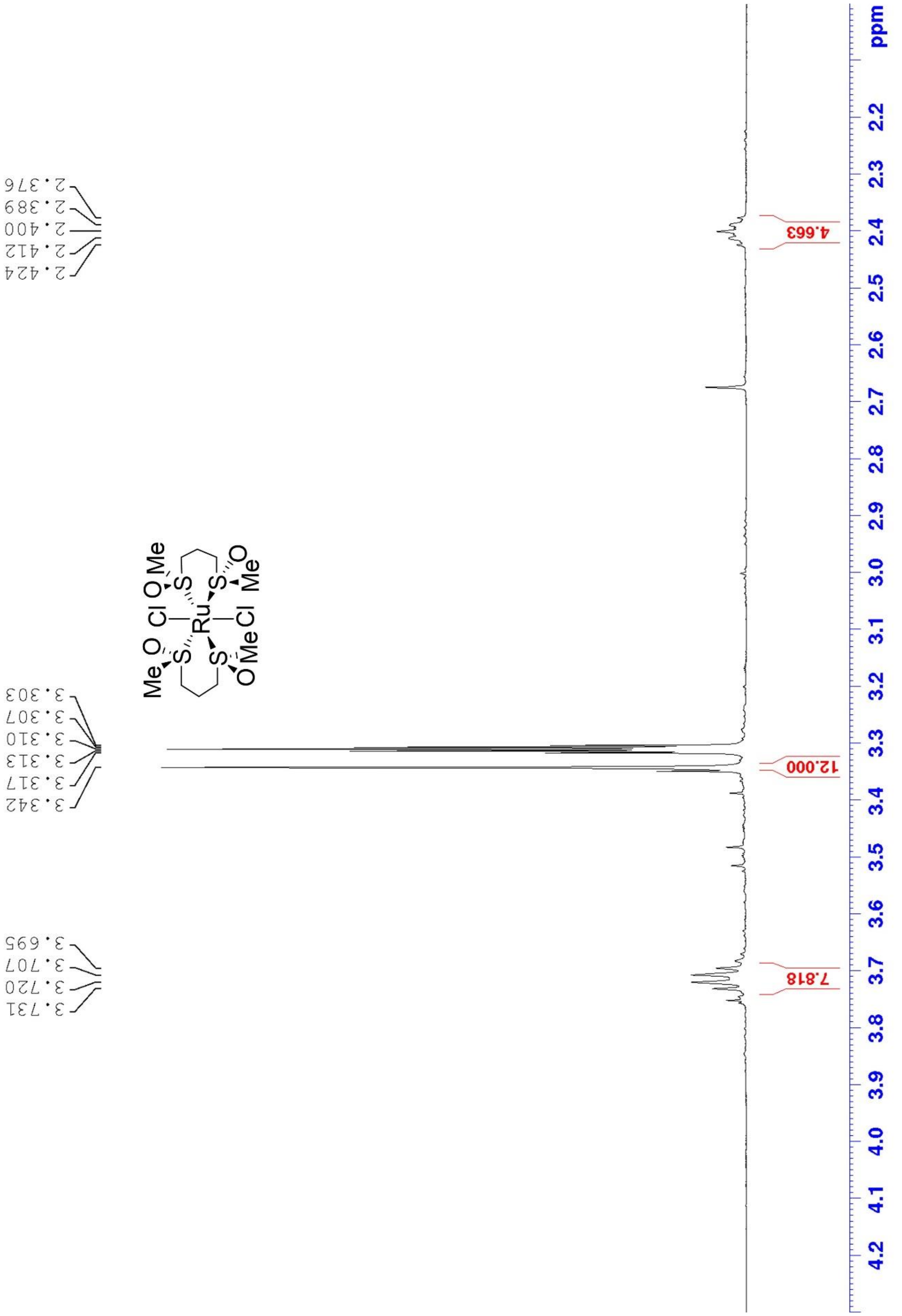

$^{13}C\{^{1}H\}$ NMR (125 MHz, MeOD)

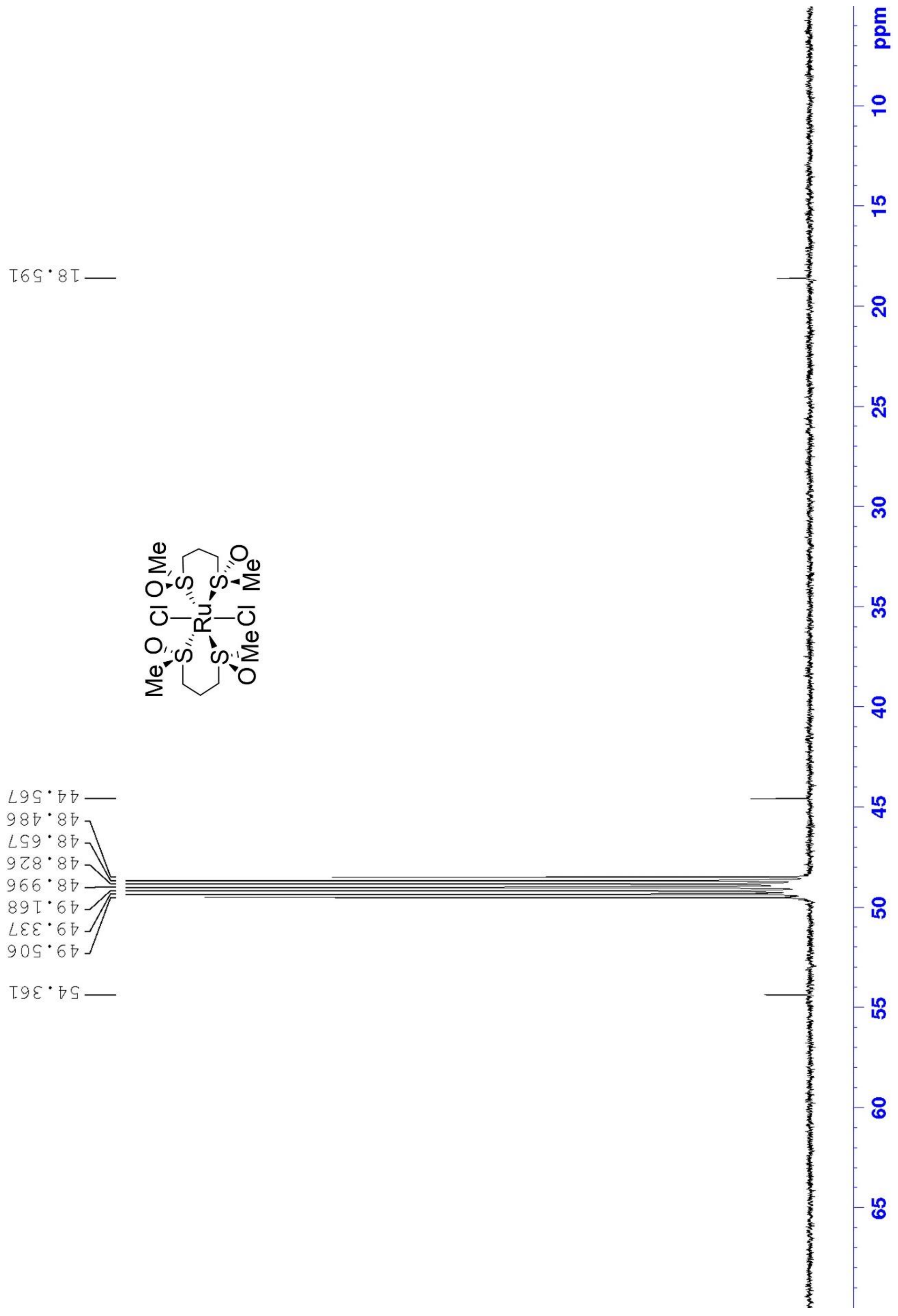

trans-Dichlorobis[(S,S)-1,3-bis(methylsulfinyl)propane]ruthenium (II), **36(*S,S*)**

^{1}H NMR (500 MHz, MeOD)

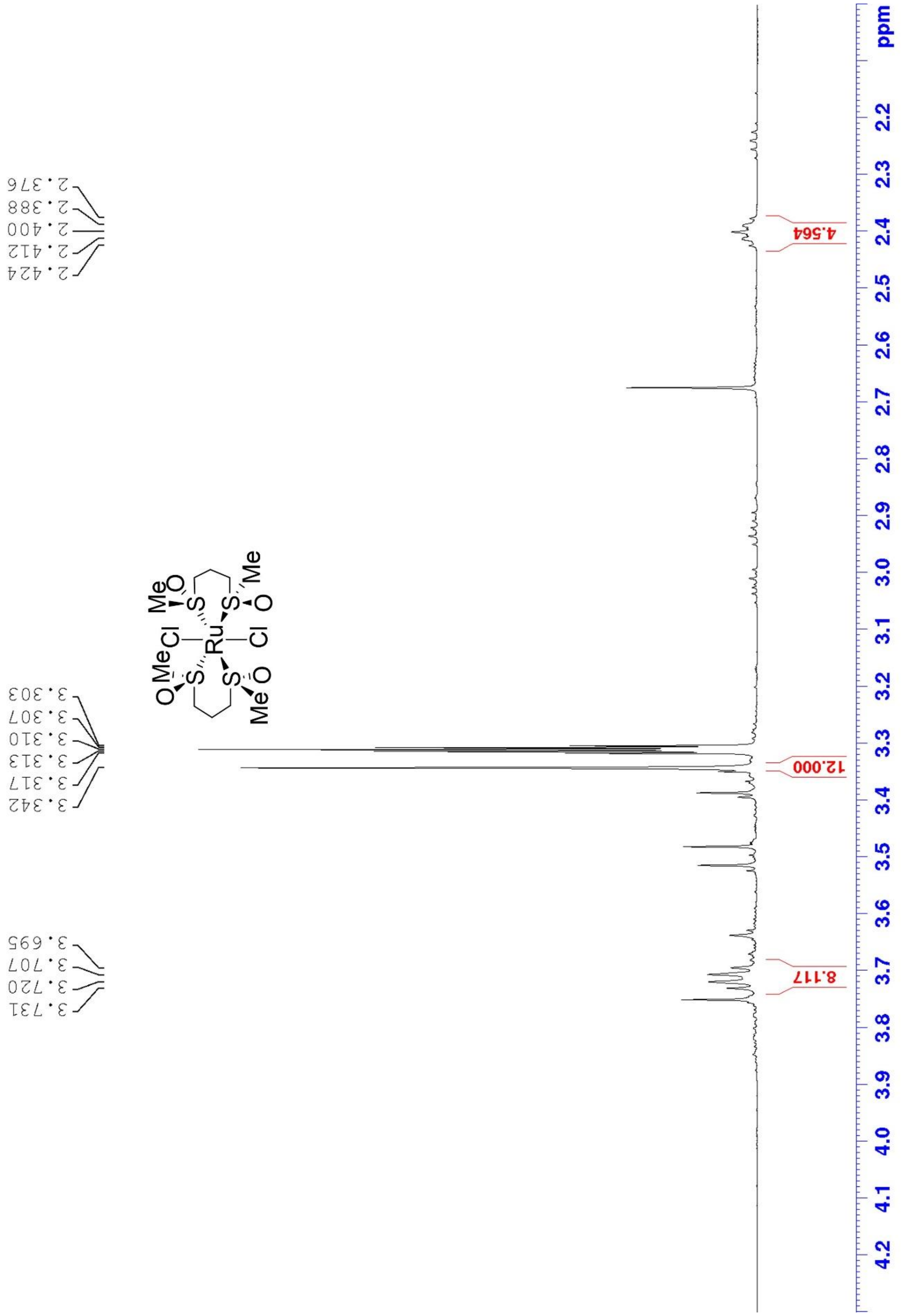

$^{13}C\{^1H\}$ NMR (125 MHz, MeOD)

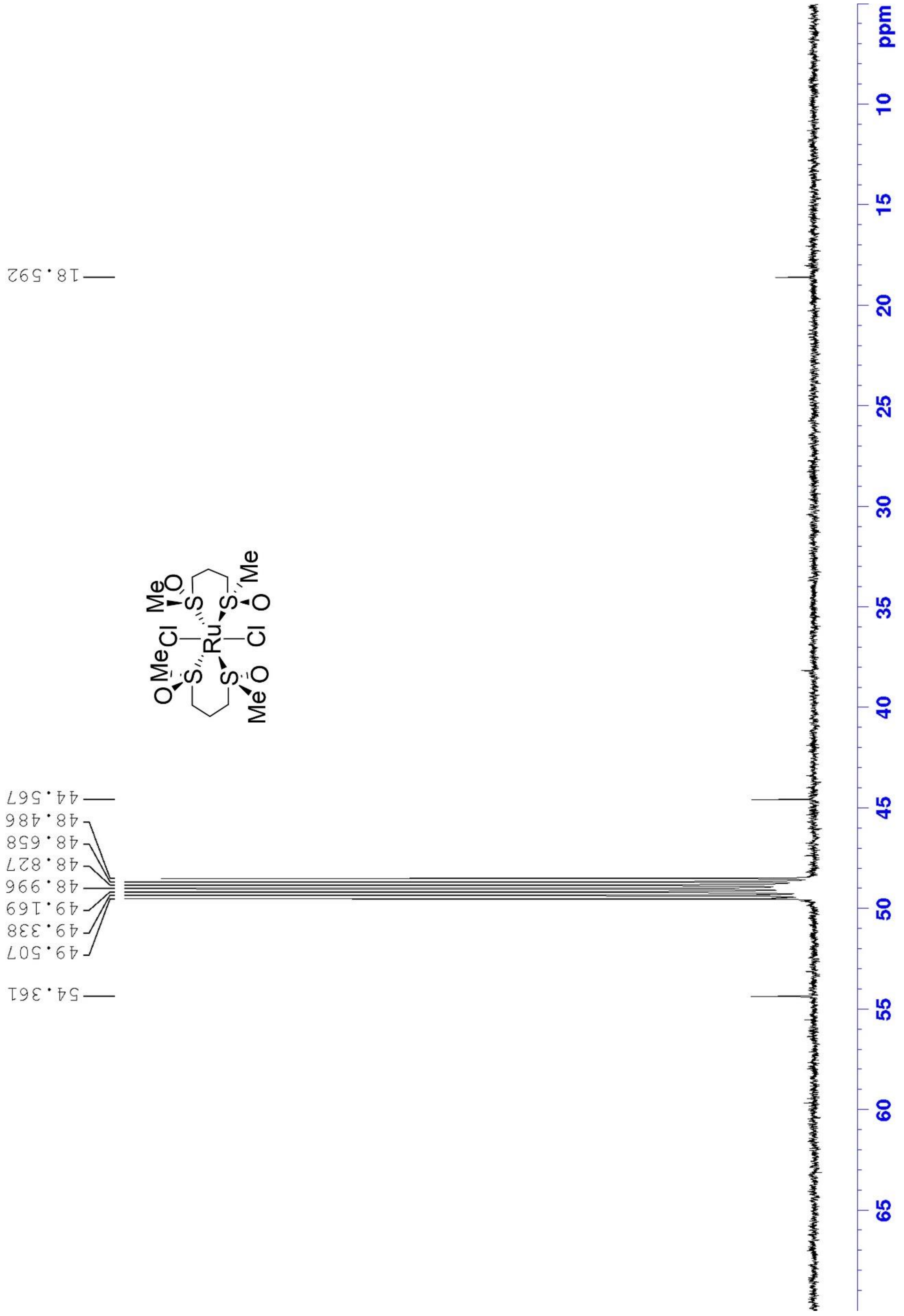

trans-Dichlorobis[(R,R)-1,3-bis(ethylsulfinyl)propane]ruthenium (II), **37(*R*,*R*)**

^{1}H NMR (500 MHz, MeOD)

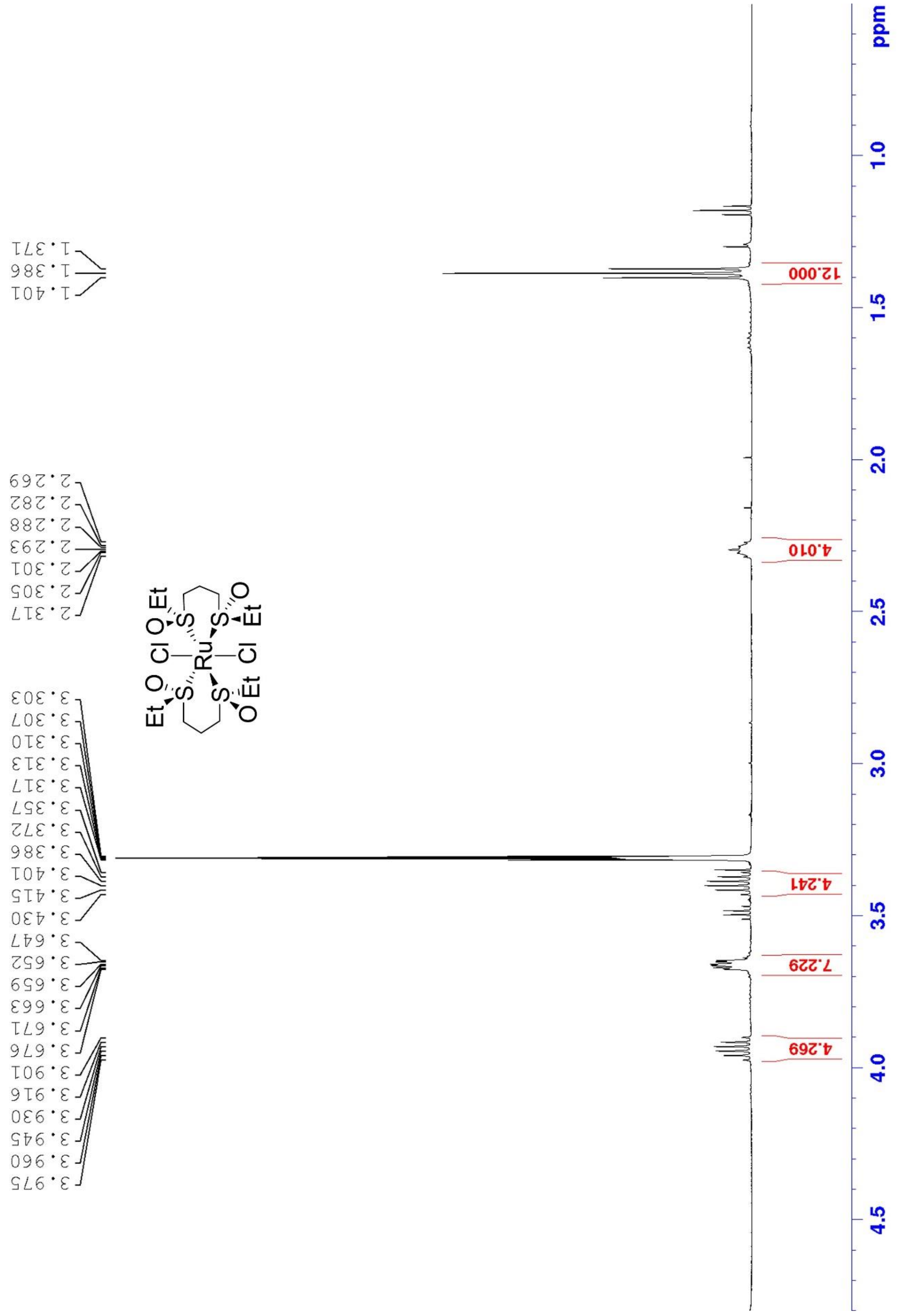

$^{13}C\{^{1}H\}$ NMR (125 MHz, MeOD)

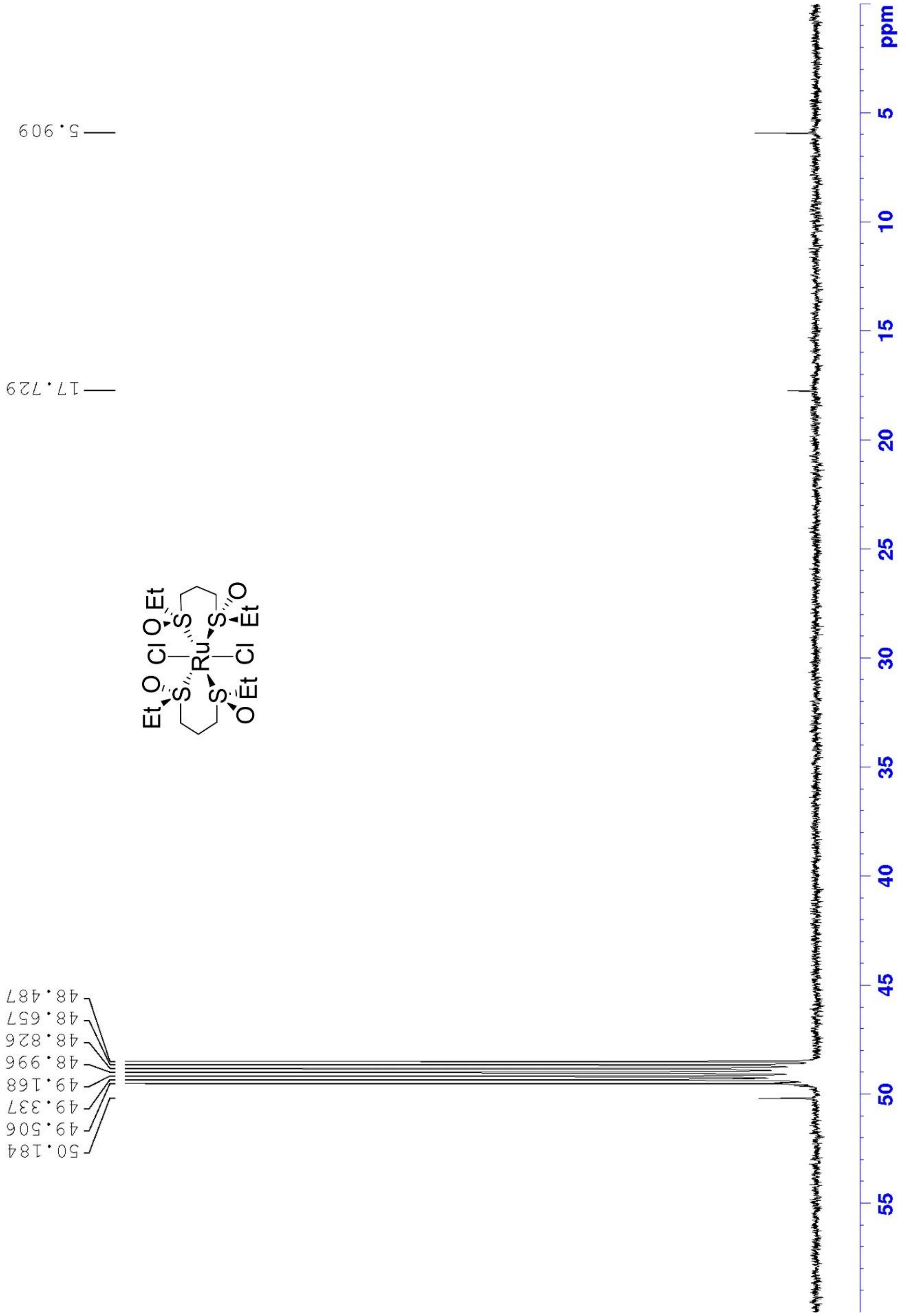

trans-Dichlorobis[(S,S)-1,3-bis(ethylsulfinyl)propane]ruthenium (II), **37(*S,S*)**

^{1}H NMR (500 MHz, MeOD)

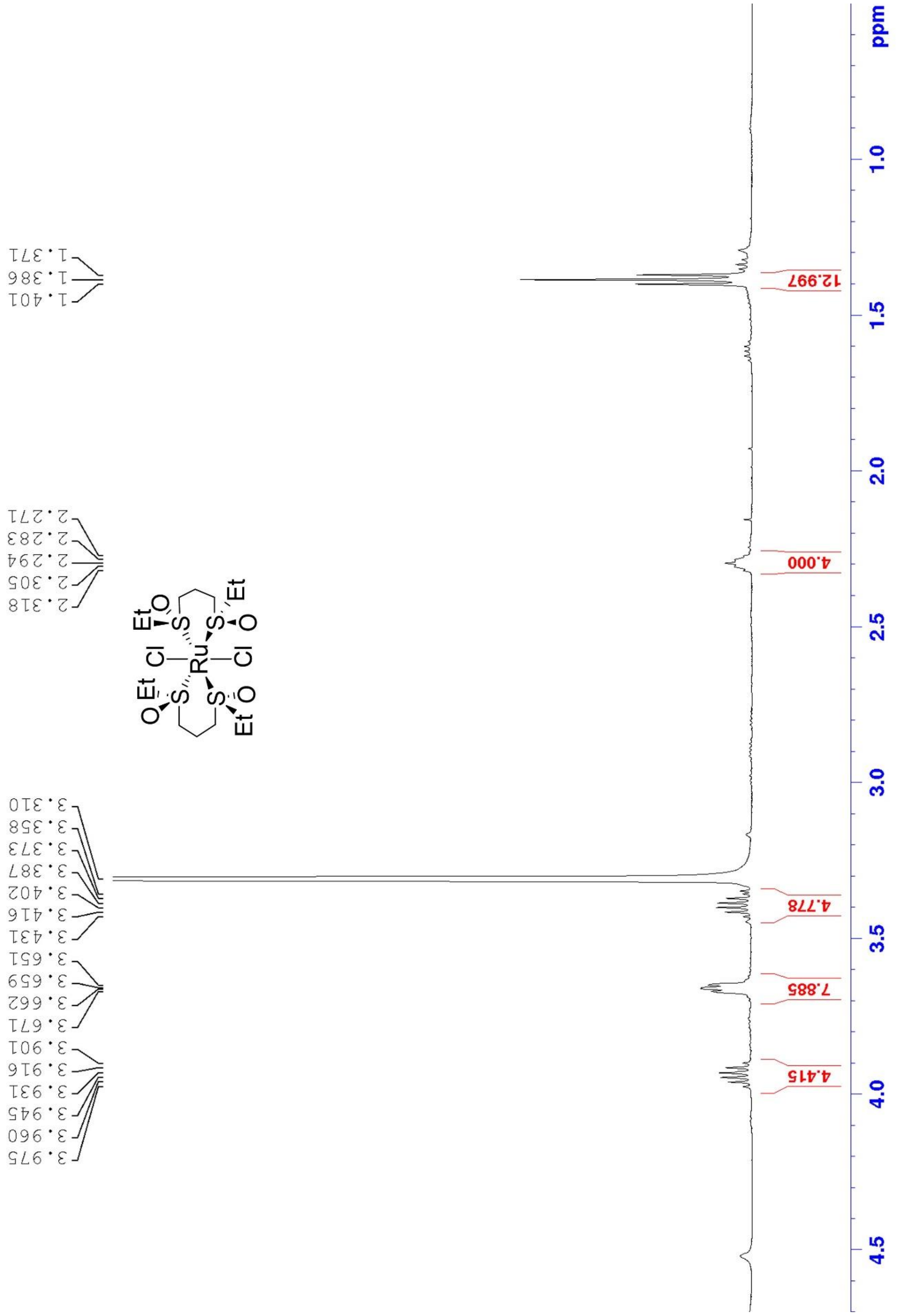

$^{13}C\{^1H\}$ NMR (125 MHz, MeOD)

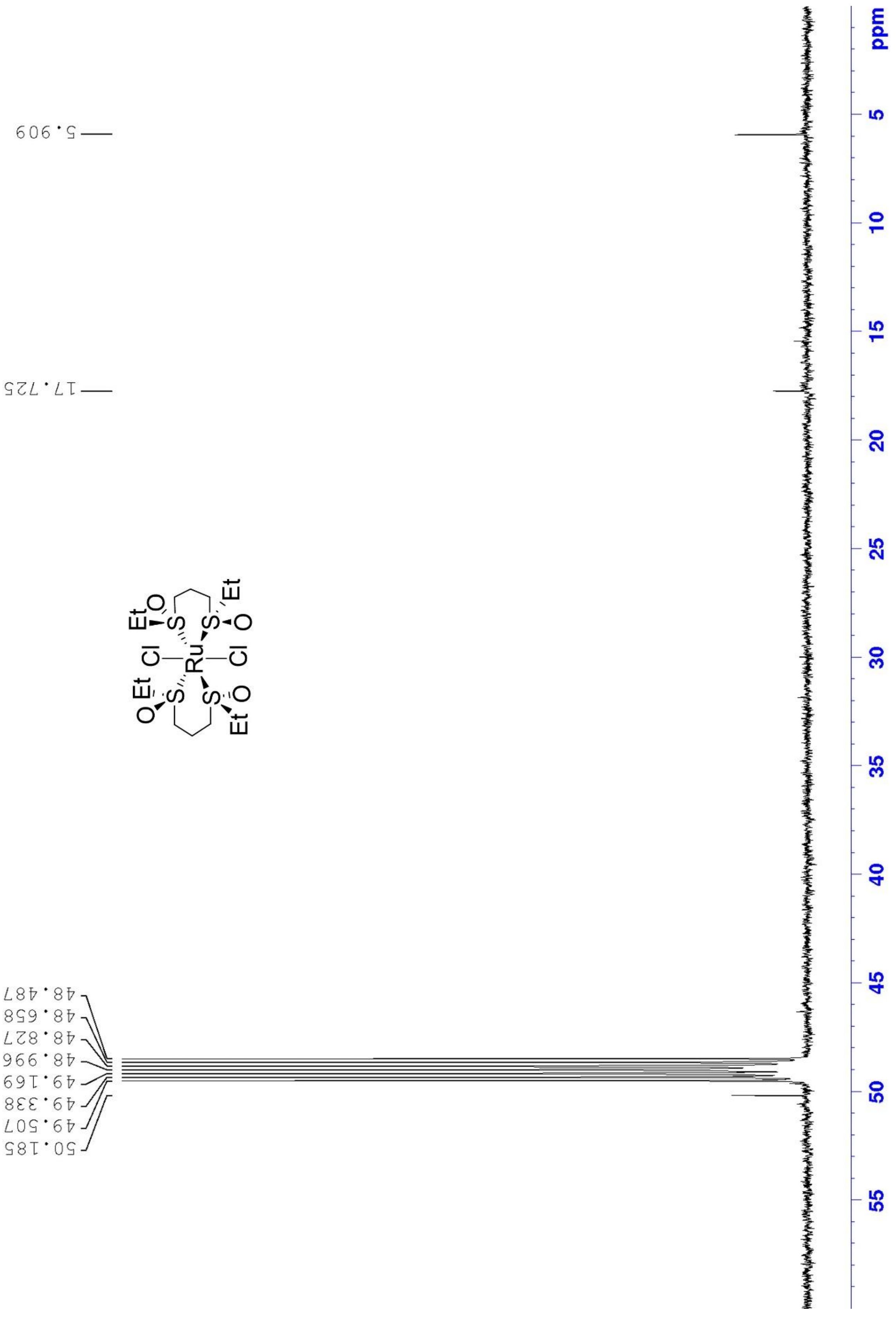

mer-[26(R,R)·RuCl$_2$]$_2$, **38(*S*,*S*)**

^{1}H NMR (500 MHz, MeOD)

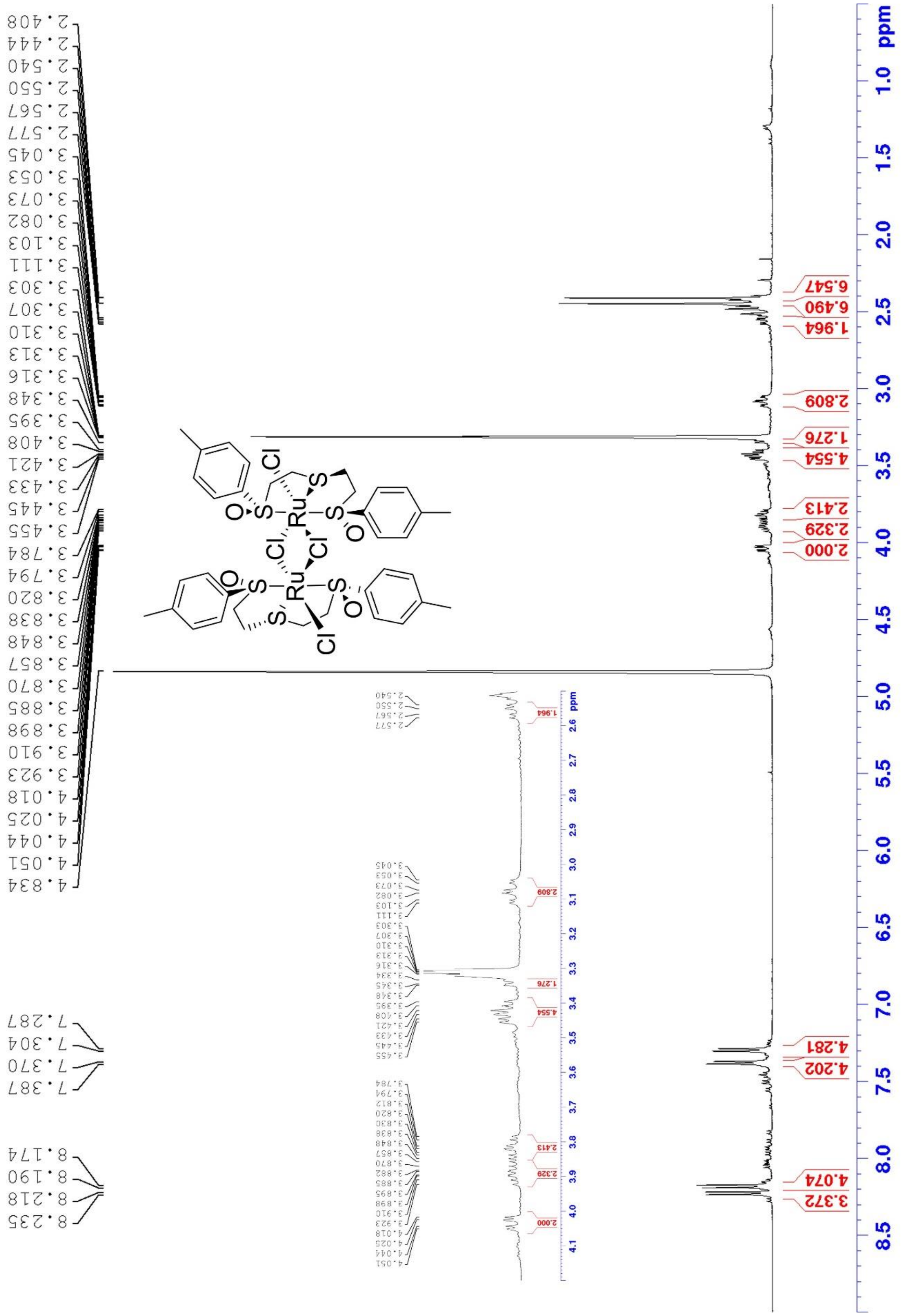

$^{13}C\{^{1}H\}$ NMR (125 MHz, MeOD)

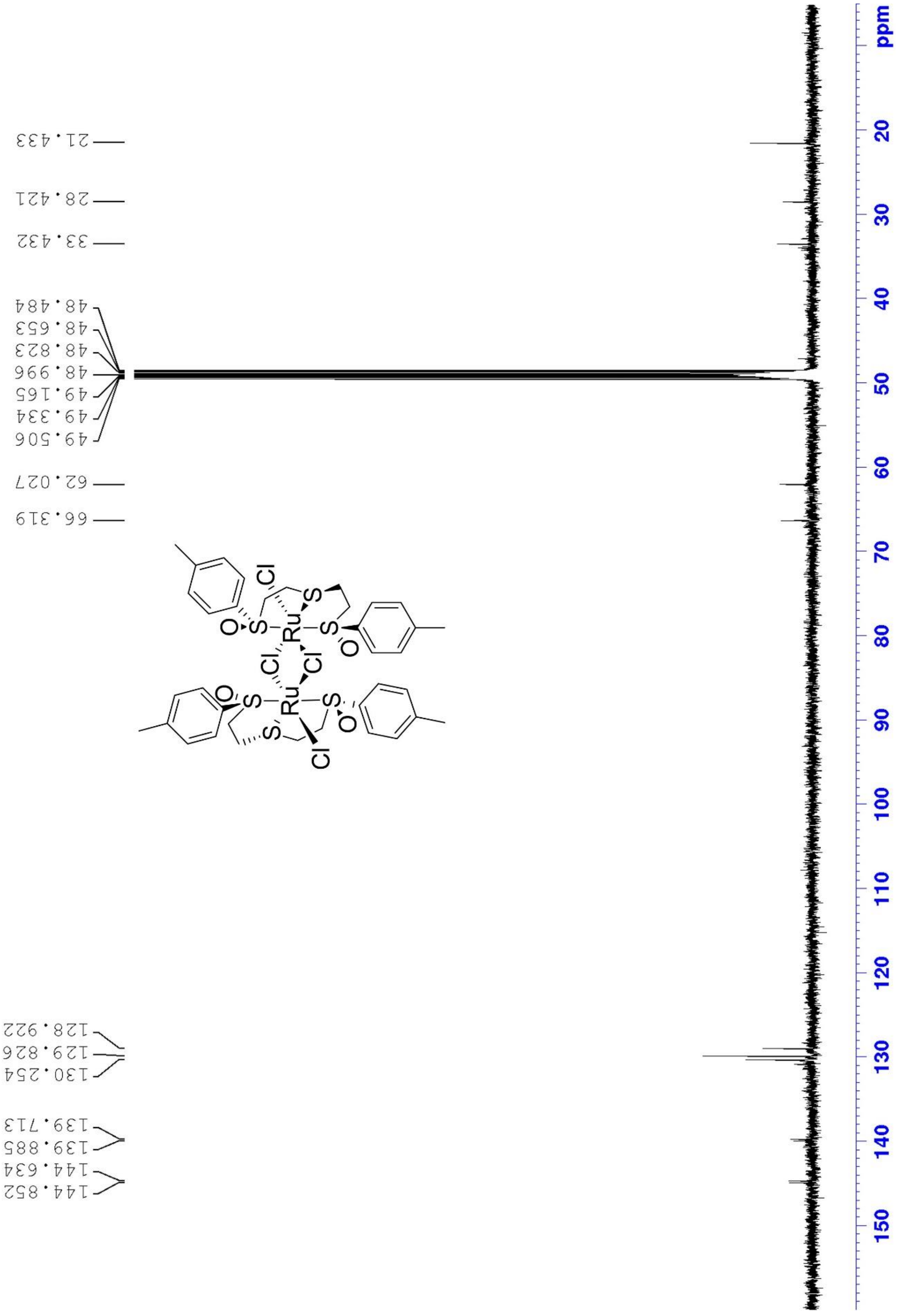

trans-Dichloro{[(S,S)-Bis(2-(p-tolylsulfinyl)ethyl)] sulfoxide-κ^{3}S}(methanol)ruthenium (II), **39(*S,S*)**

^{1}H NMR (500 MHz, MeOD)

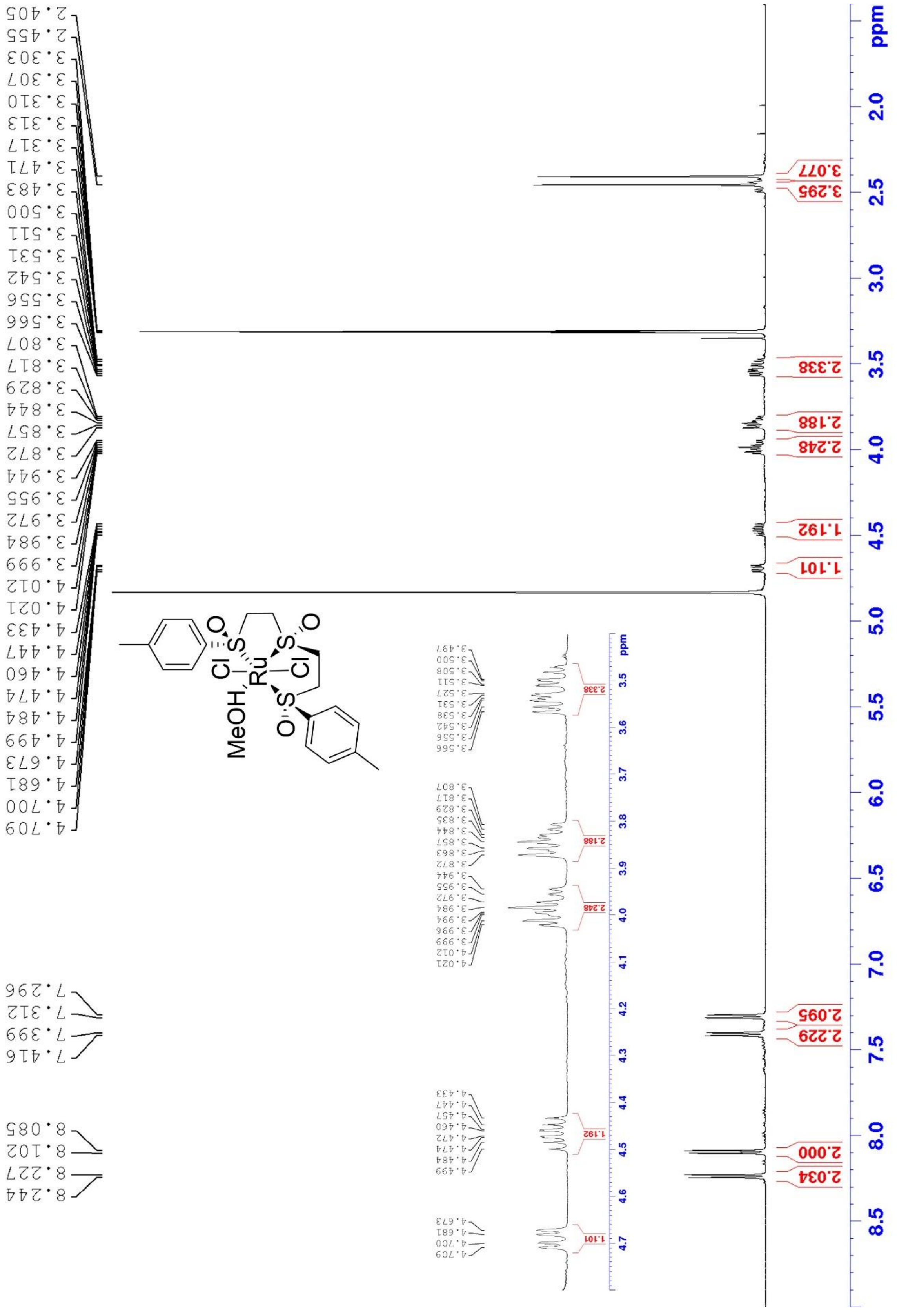

^{13}C{^{1}H} NMR (125 MHz, MeOD)

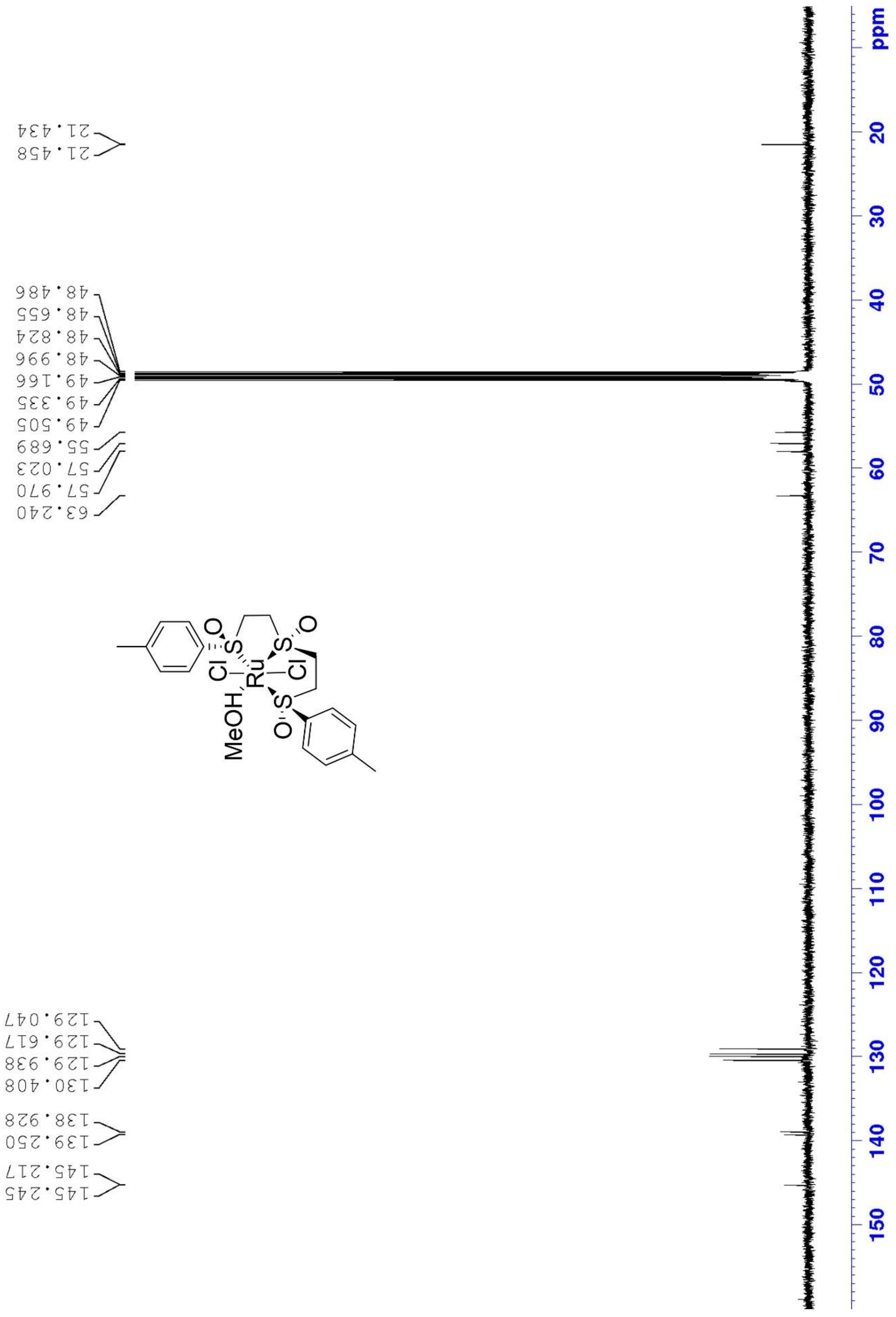

cis-[(R,R)-1,3-Bis(benzylsulfinyl)propane]palladium(II) trifluoroacetate, **12(*S*,*S*)·PdTFA$_2$**

^{1}H NMR (500 MHz, CDCl$_3$)

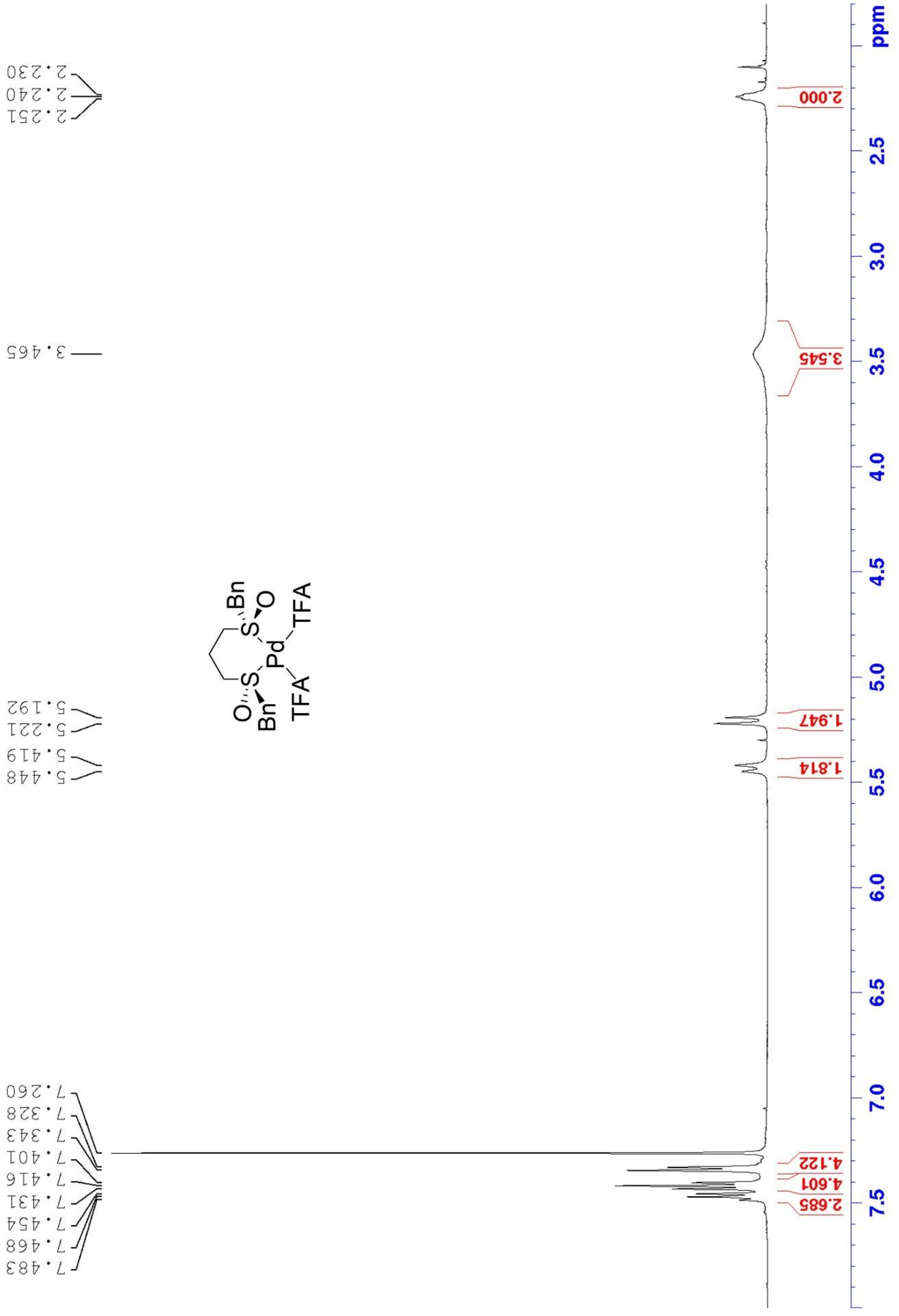

$^{13}C\{^{1}H\}$ NMR (125 MHz, $CDCl_3$)

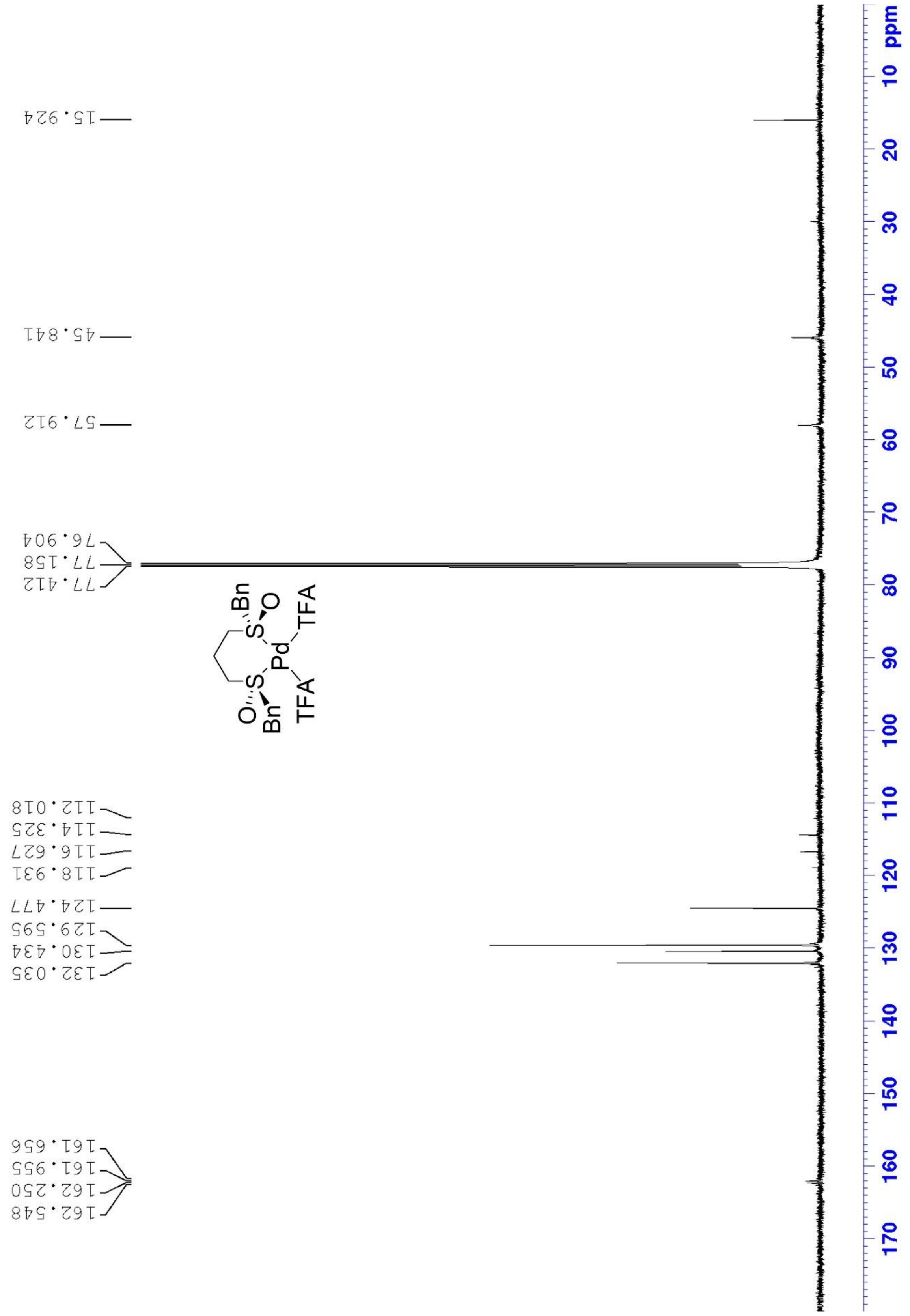

^{19}F NMR (470 MHz, $CDCl_3$)

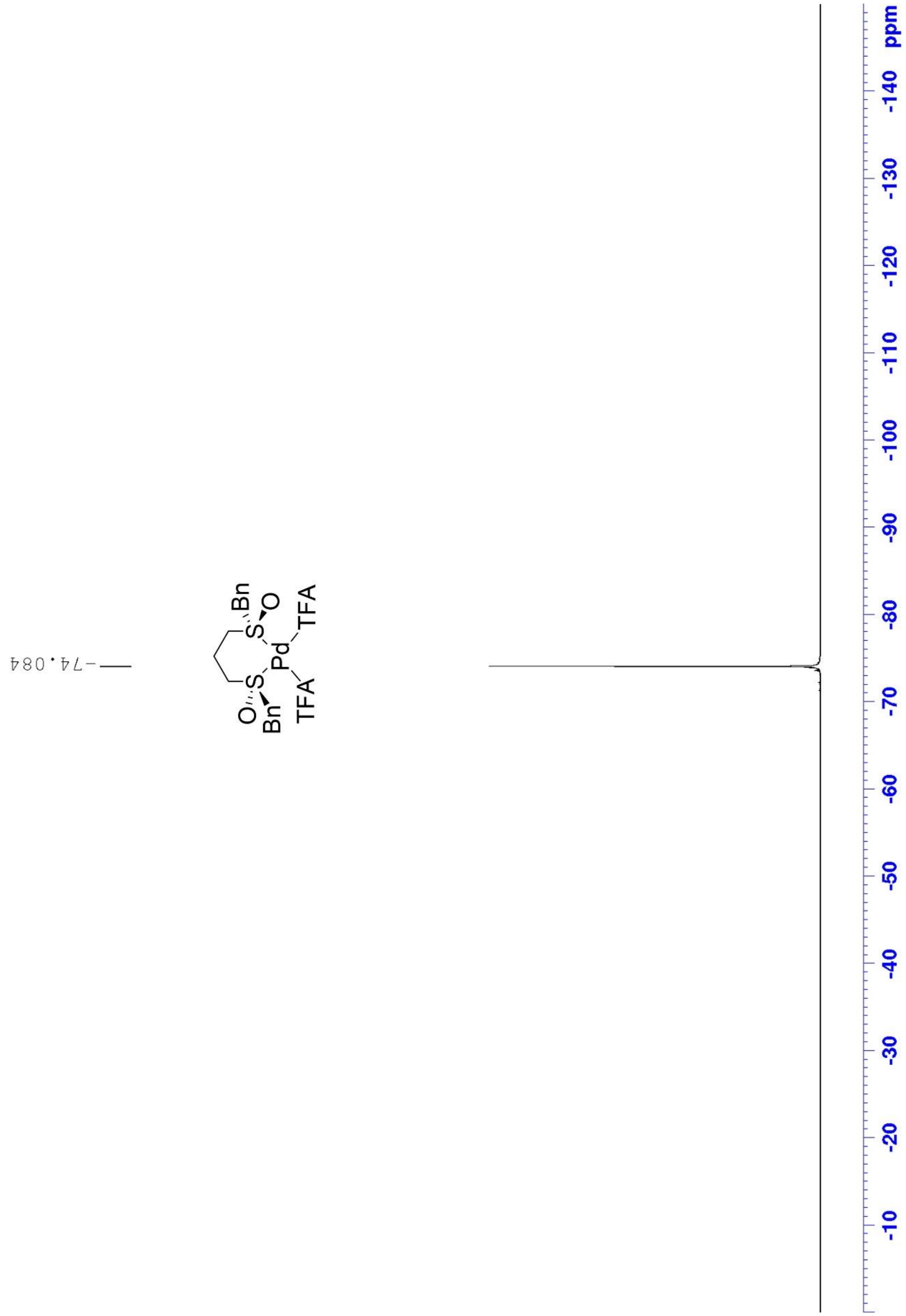

cis-[(R,R)-1,3-Bis(phenylsulfinyl)propane]palladium(II) trifluoroacetate, **8(*S*,*S*)·PdTFA$_2$**

^{1}H NMR (500 MHz, $CDCl_3$)

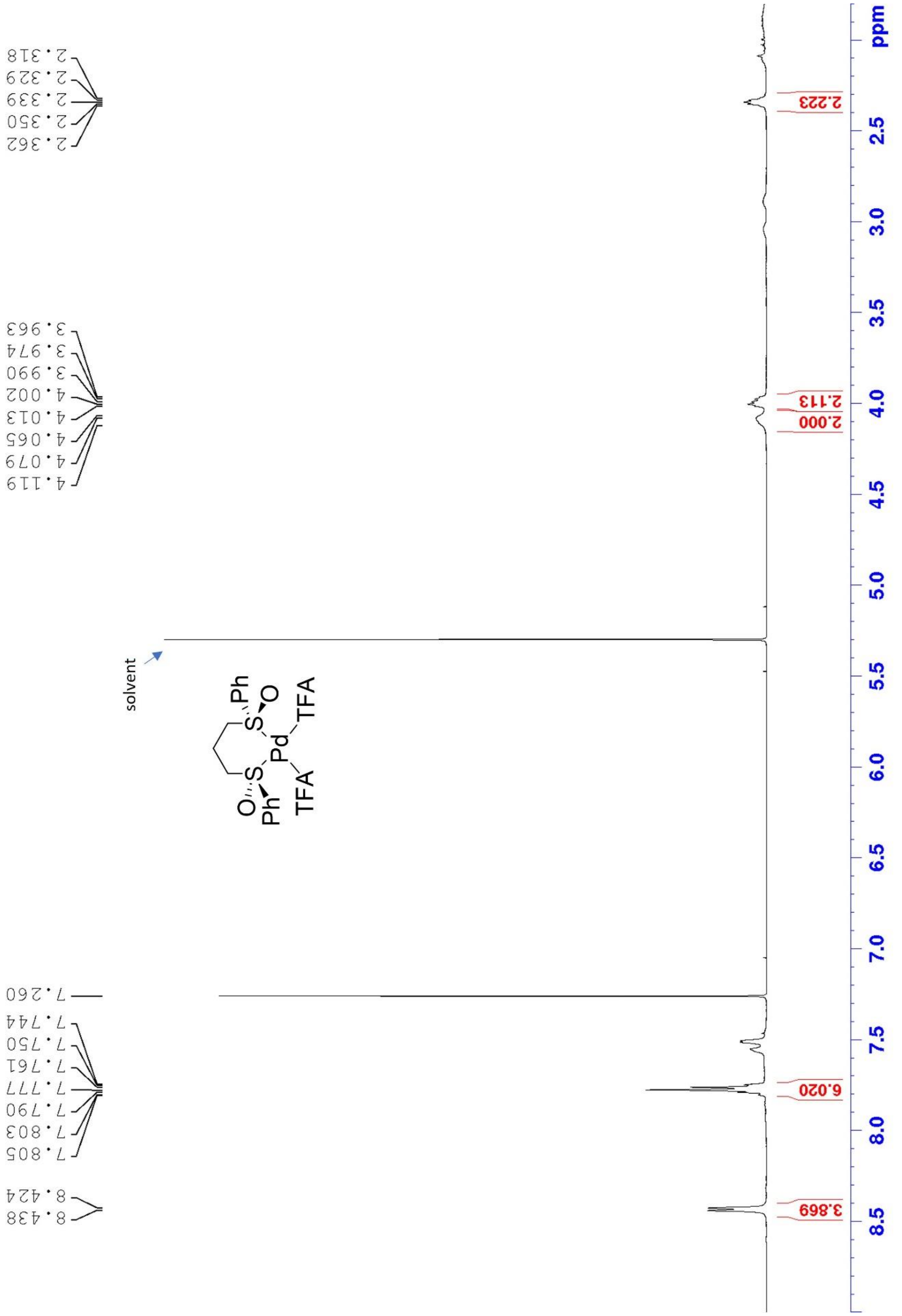

$^{13}C\{^{1}H\}$ NMR (125 MHz, $CDCl_3$)

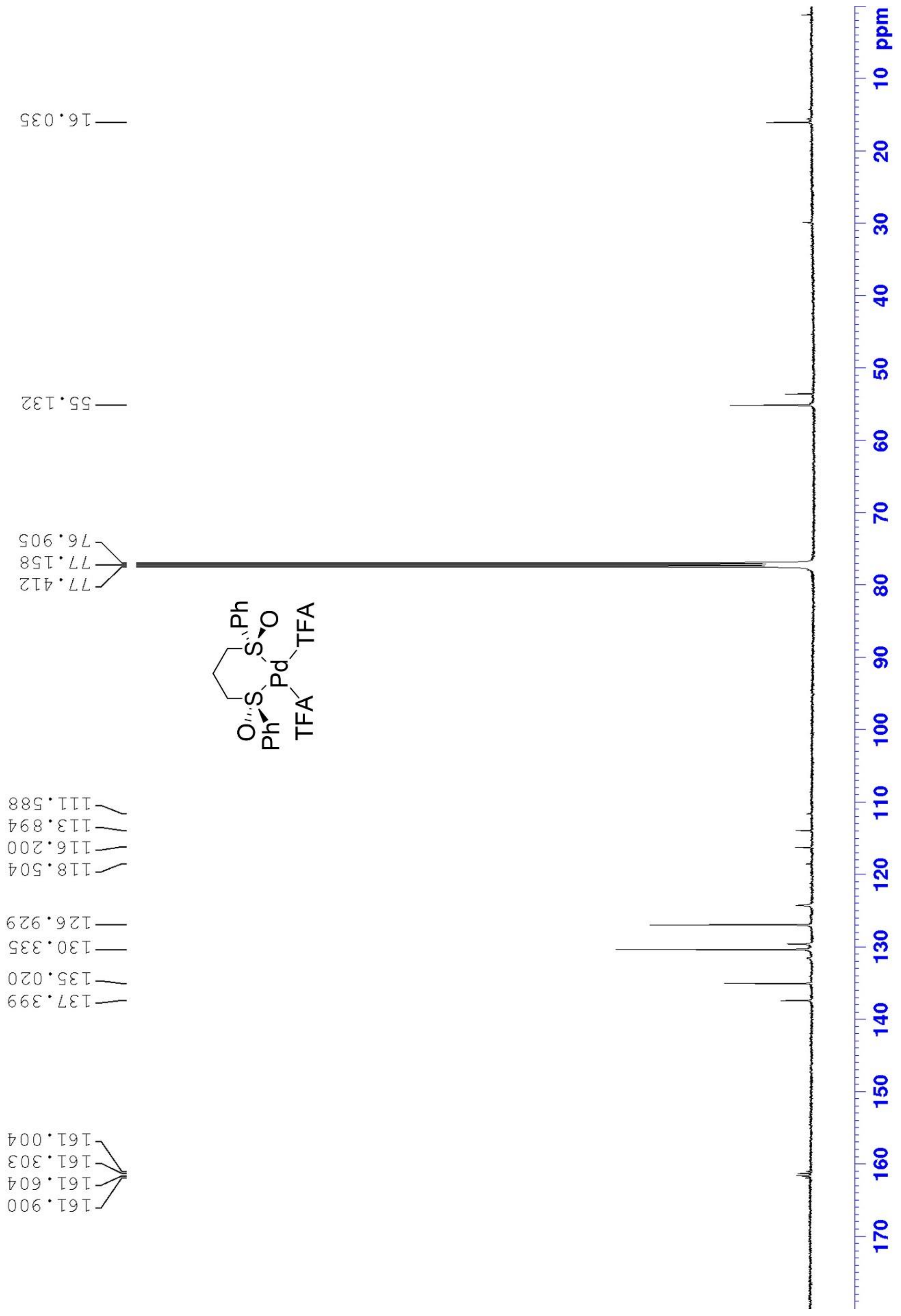

^{19}F NMR (470 MHz, $CDCl_3$)

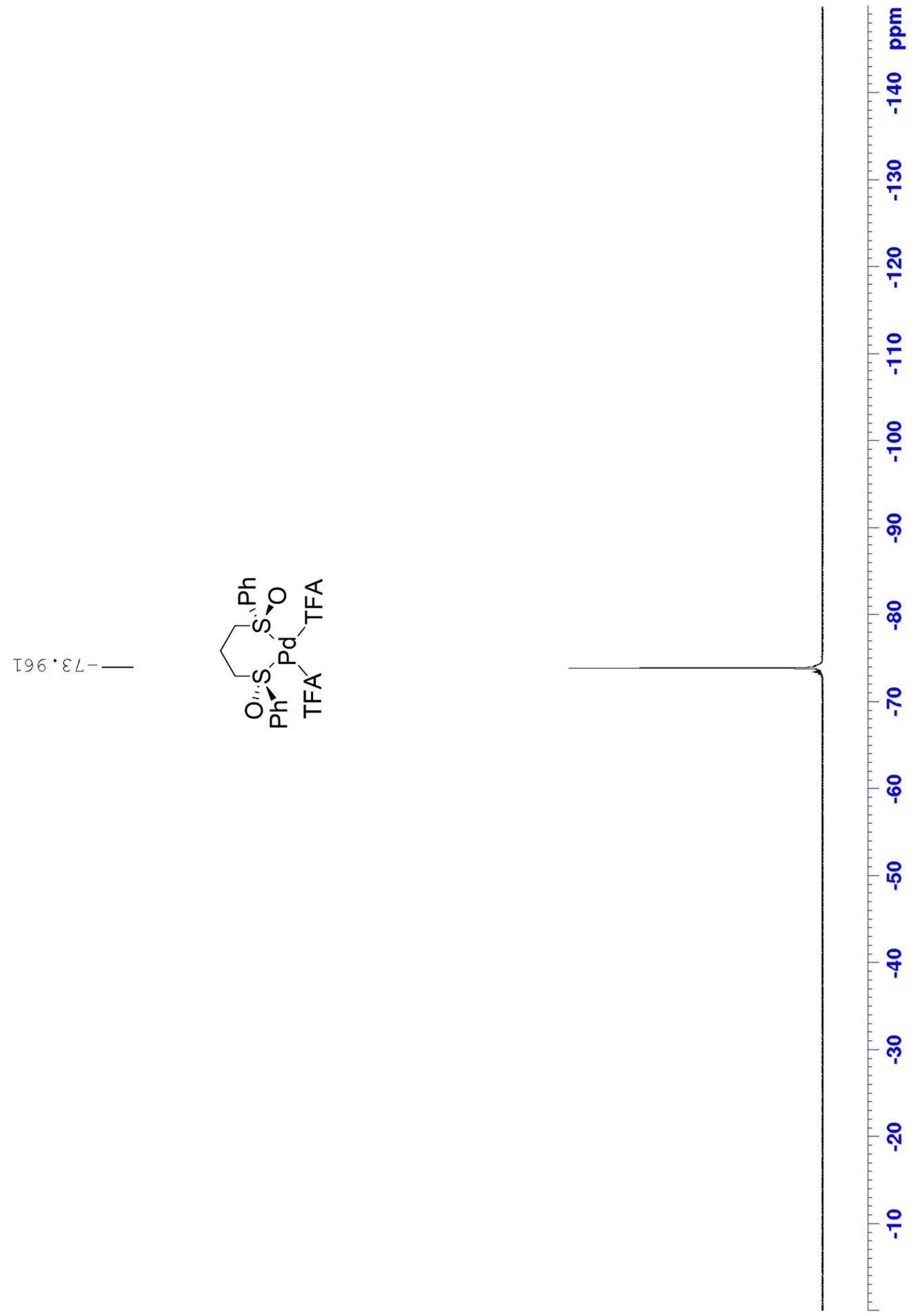

cis-[(S,S)-1,3-Bis(propylsulfinyl)propane]palladium(II) trifluoroacetate, **16(*R*,*R*)·PdTFA$_2$**

^{1}H NMR (500 MHz, $CDCl_3$)

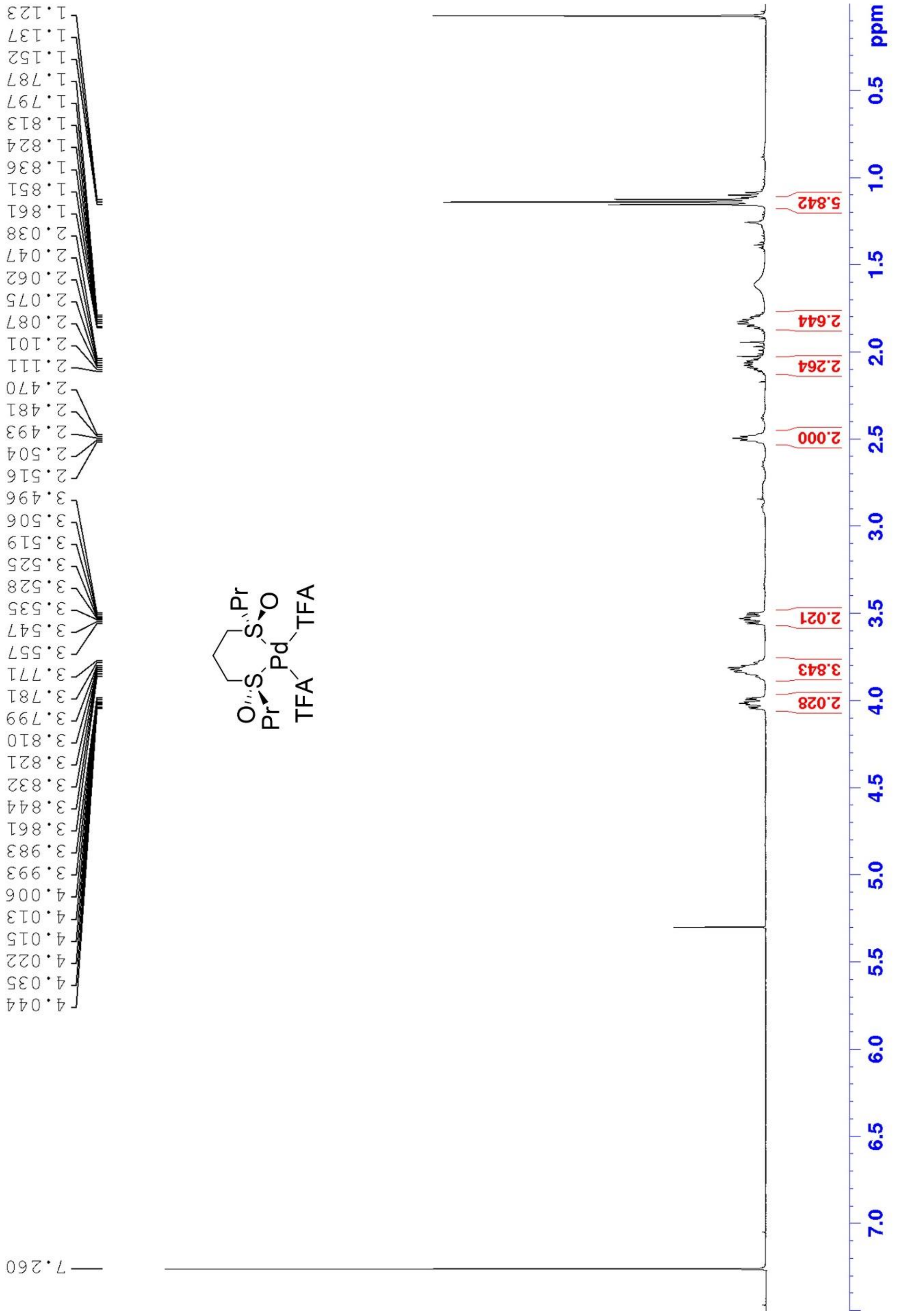

$^{13}C\{^{1}H\}$ NMR (125 MHz, $CDCl_3$)

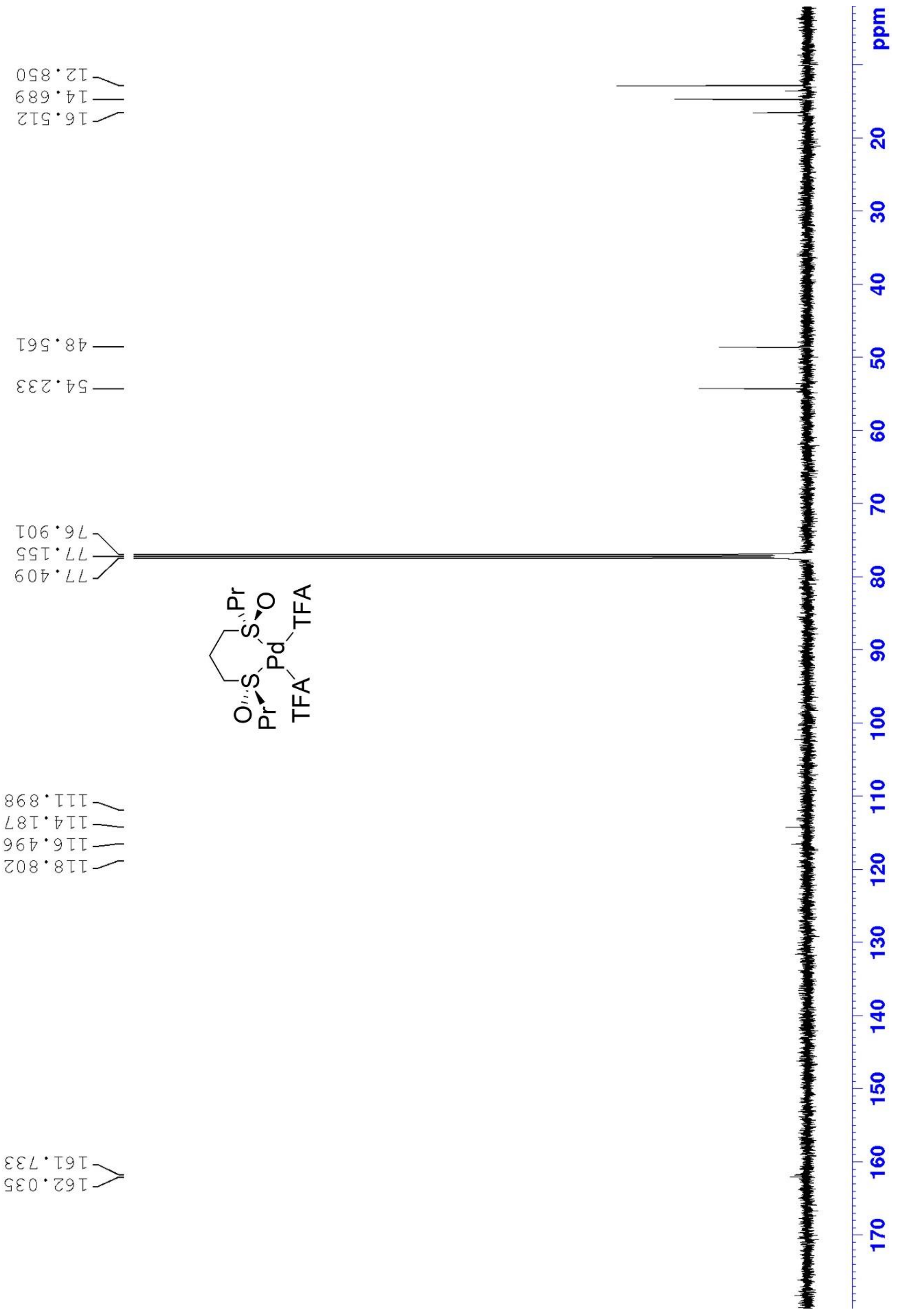

^{19}F NMR (470 MHz, $CDCl_3$)

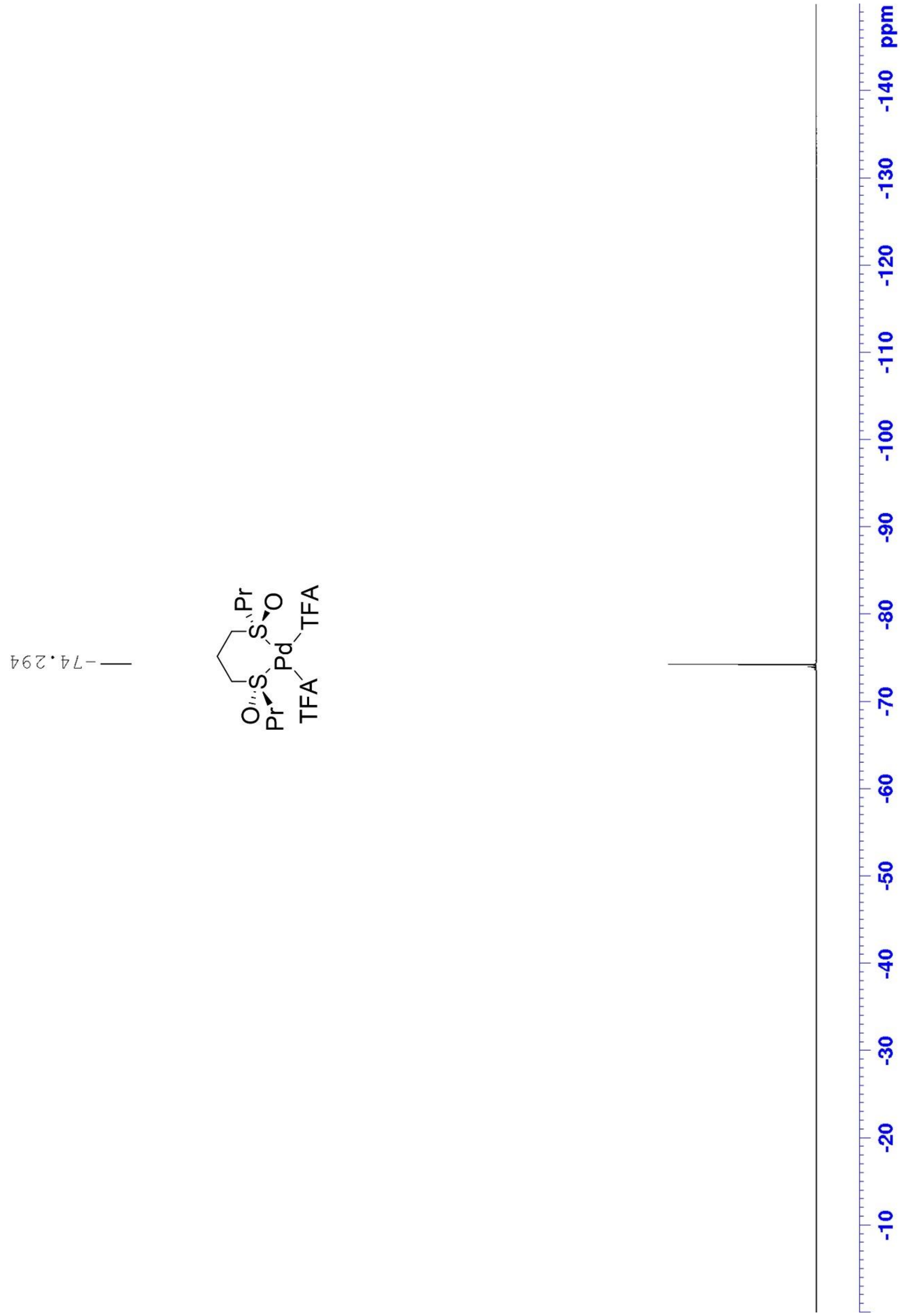

2D NMR of selected compounds

COSY NMR of mer-[26(R,R)·$RuCl_2$]$_2$, **38(*S*,*S*)**

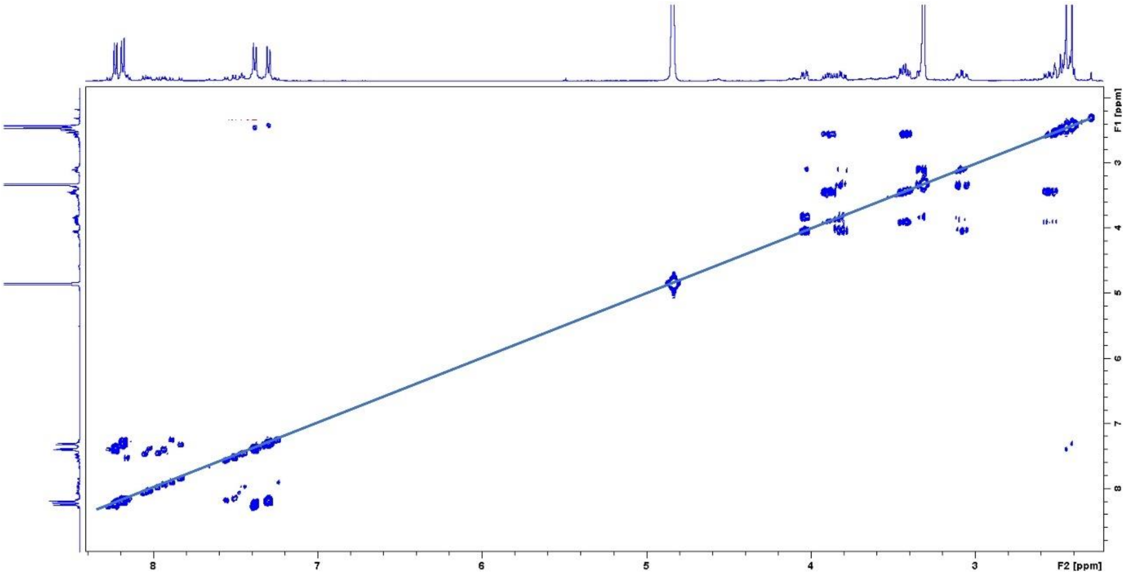

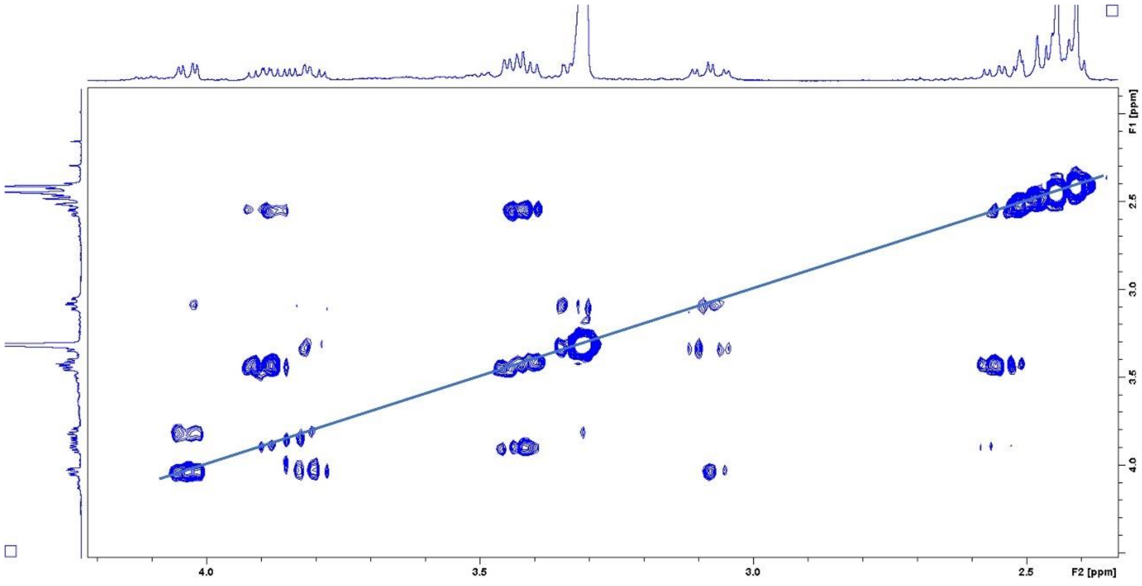

HSQC NMR of mer-[26(R,R)·RuCl$_2$]$_2$, **38(*S*,*S*)**

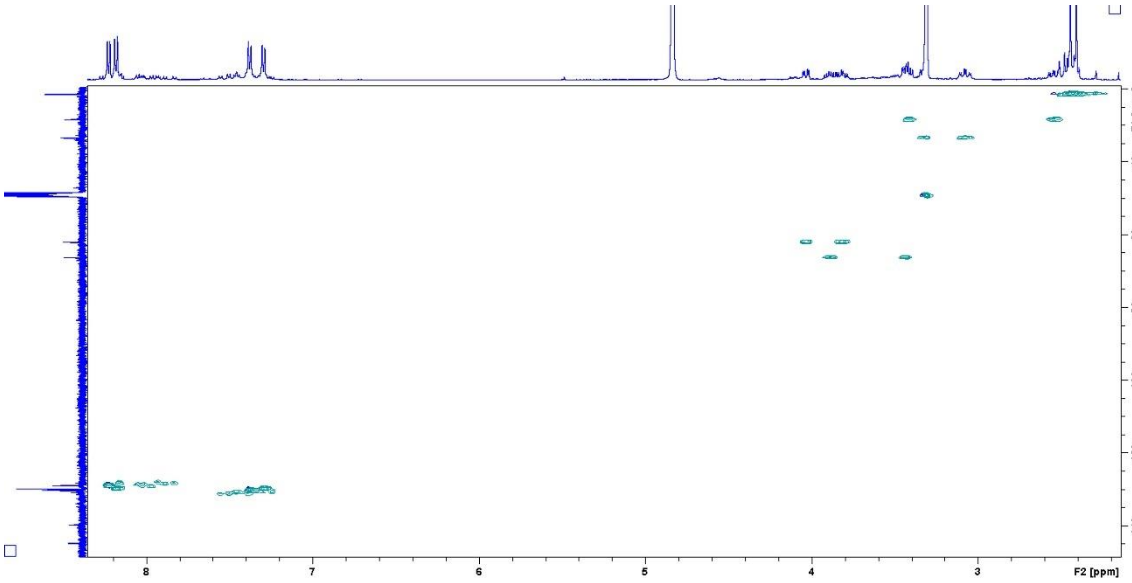

COSY NMR of trans-Dichloro{[(S,S)-Bis(2-(p-tolylsulfinyl)ethyl] sulfoxide-κ^3S}(methanol)ruthenium (II), **39(*S*,*S*)**

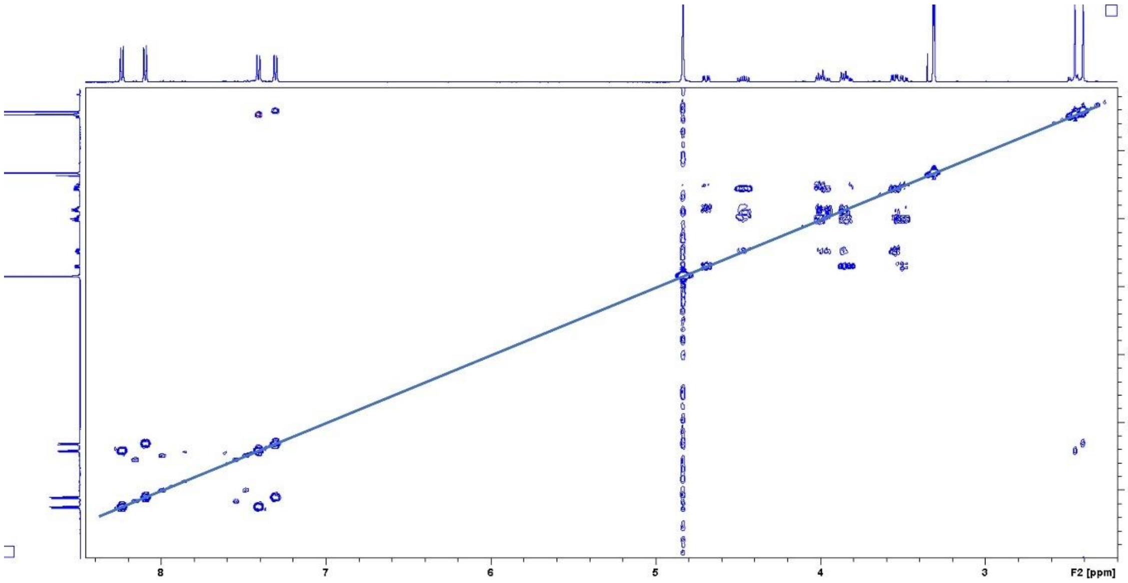

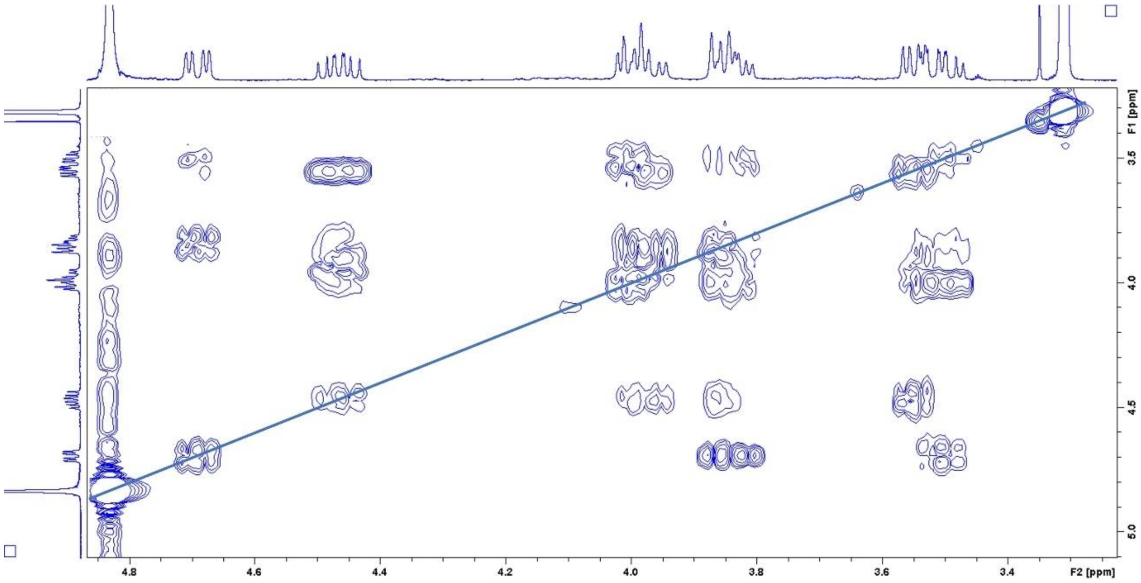

HSQC NMR of trans-Dichloro{[(S,S)-Bis(2-(p-tolylsulfinyl)ethyl] sulfoxide-κ^{3}S}(methanol) ruthenium (II), **39(*S,S*)**

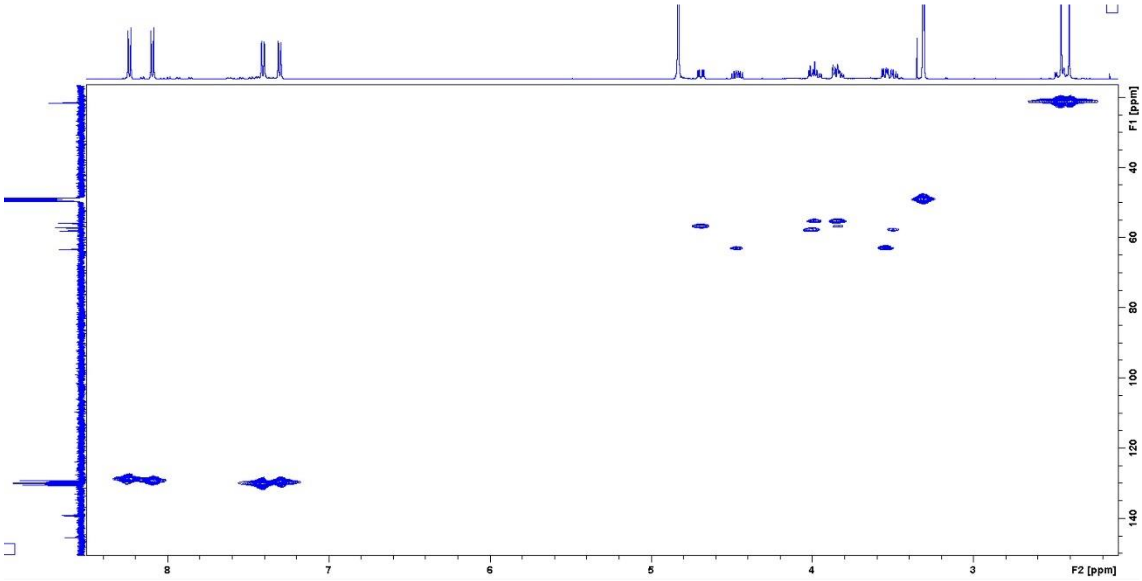

NOESY NMR of trans-Dichloro{[(S,S)-Bis(2-(p-tolylsulfinyl)ethyl] sulfoxide-κ^{3}S}(methanol) ruthenium (II), **39(*S*,*S*)**

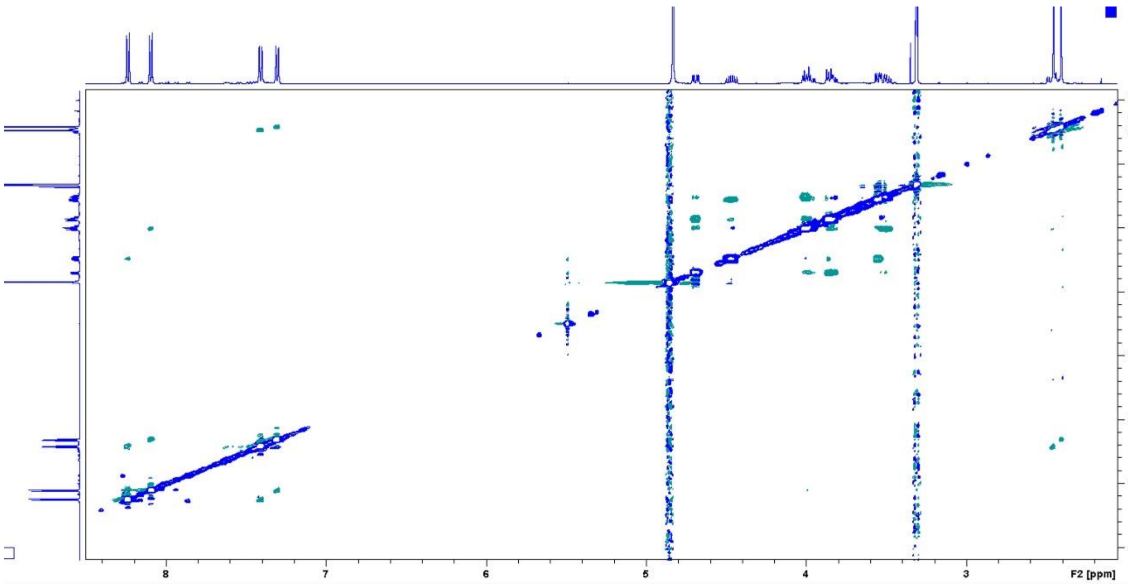

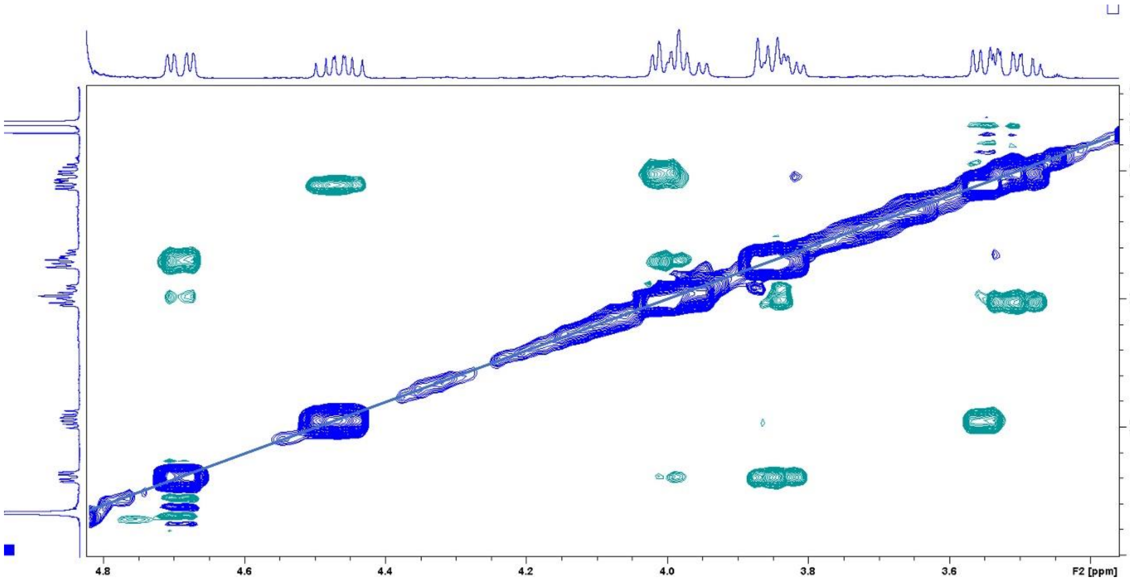

X-ray Structural Analysis for the compounds of this work.

A summary of the crystallographic data and the structure refinement results for compound **30(*R*,*R*)** are given in **Table S2**. Crystals of suitable size for X-ray diffraction analysis were grown in n-hexane/CH_2Cl_2 at r.t., coated with dry perfluoropolyether and mounted on glass fibers and fixed in a cold nitrogen stream (T = 193 K) to the goniometer head. Data collection was carried out on a Bruker-AXS D8 Quest ECO single crystal diffractometer with a Photon II detector X-ray source at the Instituto de Investigaciones Químicas (Sevilla). Data were collected by means of ω and ϕ scans using monochromatic radiation λ(Mo Kα) = 0.71073 Å. The structures were solved with SHELXT [2] and were refined against F^2 on all data by full-matrix least squares with SHELXL,[3] using Olex2 as graphical interface.[4] All non-hydrogen atoms were refined anisotropically. Hydrogen atoms were included in the model at geometrically calculated positions and refined using a riding model, unless otherwise noted. The isotropic displacement parameters of all hydrogen atoms were fixed to 1.2 times the U value of the atoms to which they are linked (1.5 times for methyl groups). CCDC 2309479 [**30(*R*,*R*)**] contain the supplementary crystallographic data for this paper. The data can be obtained free of charge via: https://www.ccdc.cam.ac.uk/structures/.

X-ray structure determination of selected ligands

(S,S)-Bis[(2-(methylsulfinyl)ethyl] sulfide, **30(*R*,*R*)**

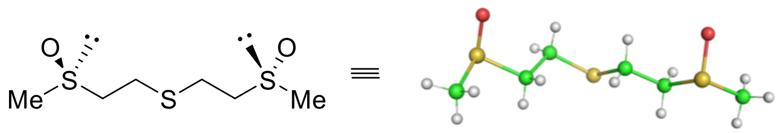

Table S2. Crystal data and structure refinement for 30(*R*,*R*).

Empirical formula	$C_6H_{14}O_2S_3$	
Formula weight	214.35	
Temperature	193.00 K	
Crystal system	orthorhombic	
Space group	$P2_12_12_1$	
Unit cell dimensions	a = 5.1136(2) Å	α = 90°
	b = 7.8031(3) Å	β = 90°
	c = 25.9126(10) Å	γ = 90°
Volume	1033.96(7) $Å^3$	
Z	4	
ρ_{calc}	1.377 g/cm^3	
μ	0.673 mm^{-1}	
F(000)	456.0	
Crystal size	0.4 × 0.05 × 0.05 mm^3	
Radiation	MoKα (λ = 0.71073)	
2Θ range for data collection	5.452 to 56.594°	
Index ranges	-6 ≤ h ≤ 6, -10 ≤ k ≤ 10, -34 ≤ l ≤ 34	
Reflections collected	16117	
Independent reflections	2574 [R_{int} = 0.0610, R_{sigma} = 0.0338]	
Data/restraints/parameters	2574/0/102	
Goodness-of-fit on F^2	1.130	
Final R indexes [I>=2σ (I)]	R_1 = 0.0322, wR_2 = 0.0741	
Final R indexes [all data]	R_1 = 0.0349, wR_2 = 0.0750	
Largest diff. peak/hole	0.68/-0.24 e $Å^{-3}$	
Flack parameter	0.07(4)	

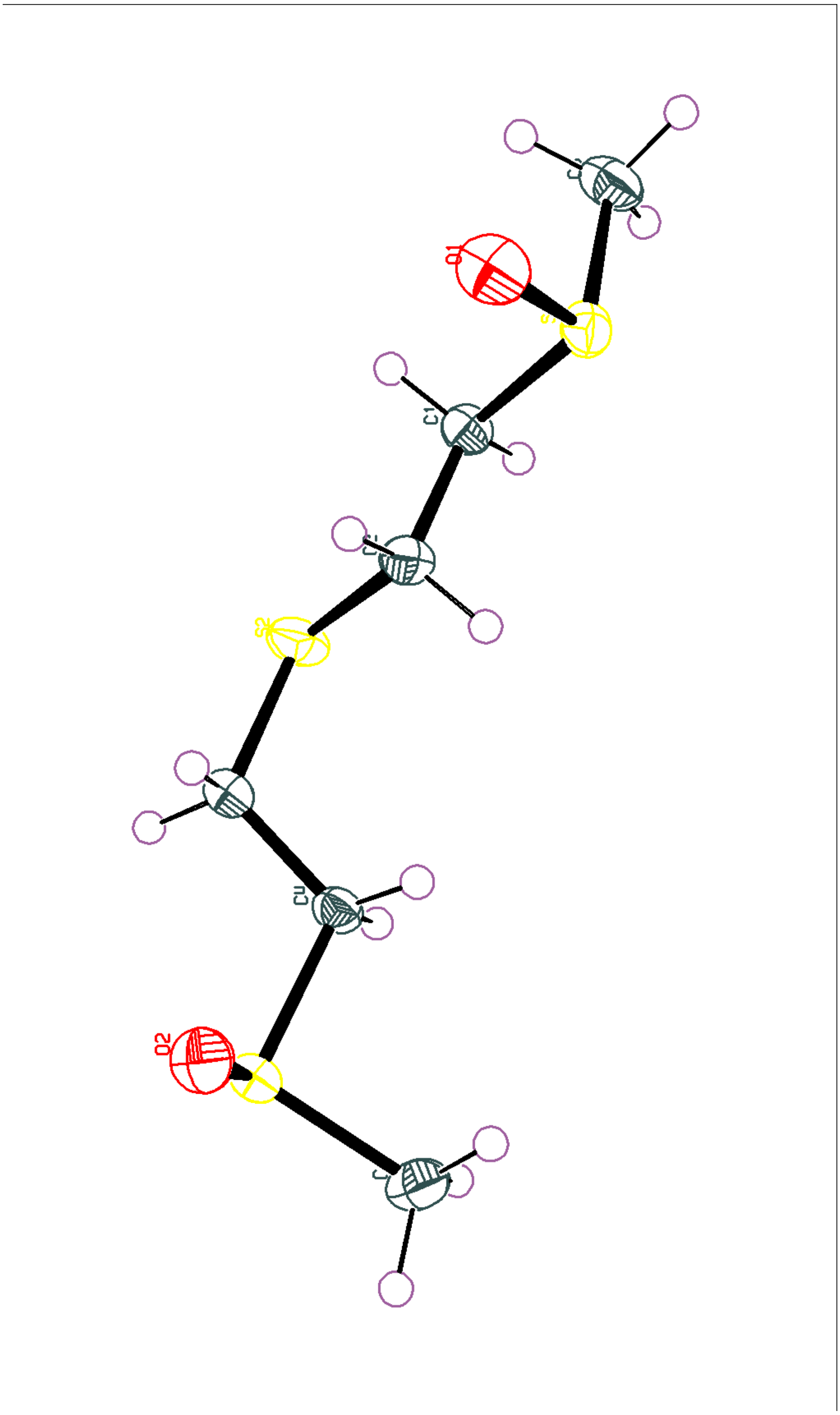

Figure S9. ORTEP drawings of **30(*R*,*R*)**; ellipsoid contour probability level = 50% (CCDC 2309479).

References

1. Fernández, I.; Valdivia, V.; Pernia Leal, M.; Khiar, N. C_2-Symmetric Bis-sulfoxides as Organocatalysts in the Allylation of Benzoyl Hydrazones: Spacer and Concentration Effects. *Org. Lett.* **2007**, *9*, 2215-2218.

2. Sheldrick, G.M. SHELXT-Integrated space-group and crystal-structure determination. *Acta Cryst.* **2015**, *A71*, 3–8.

3. Sheldrick, G.M. Crystal structure refinement with SHELXL. *Acta Cryst.* **2015**, *C71*, 3–8.

4. Dolomanov, O. V.; Bourhis, L. J.; Gildea, R. J.; Howard, J. A. K.; Puschmann, H. OLEX2: a complete structure solution, refinement and analysis program. *J. Appl. Cryst.* **2009**, *42*, 339–341.
