# Supplementary figures and images for: Stereoselective Synthesis of Chiral C2-Symmetric 1,3- and 1,5-Bis-Sulfoxides Guided by the Horeau Principle: Understanding the Influence of the Carbon Chain Nature in Its Ability for Metal Coordination
Source: J Org Chem. 2024 Oct 2;89(20):15048–61. doi: 10.1021/acs.joc.4c01729 (PMC11494659; doi:10.1021/acs.joc.4c01729)

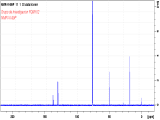

Supplement: Supplementary file 2 — jo4c01729_si_002.zip [file jo4c01729_si_002.zip › FID data/Compound 10(R,R)/Compound 10(R,R)_C13_NMR/11/pdata/1/thumb.png]

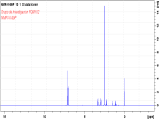

Supplement: Supplementary file 2 — jo4c01729_si_002.zip [file jo4c01729_si_002.zip › FID data/Compound 10(R,R)/Compound 10(R,R)_H1_NMR/10/pdata/1/thumb.png]

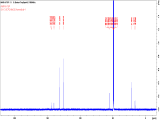

Supplement: Supplementary file 2 — jo4c01729_si_002.zip [file jo4c01729_si_002.zip › FID data/Compound 11(rac+meso)/Compound 11(rac+meso)_C13_NMR/3/pdata/1/thumb.png]
