# Supplementary material for: Stereoselective Synthesis of Chiral C2-Symmetric 1,3- and 1,5-Bis-Sulfoxides Guided by the Horeau Principle: Understanding the Influence of the Carbon Chain Nature in Its Ability for Metal Coordination
Source: J Org Chem. 2024 Oct 2;89(20):15048–61. doi: 10.1021/acs.joc.4c01729 (PMC11494659; doi:10.1021/acs.joc.4c01729)
Supplement: Supplementary file 2 — jo4c01729_si_002.zip [file jo4c01729_si_002.zip › NMR data.pdf]

NMR spectra were recorded with Bruker Avance 300 and 500 MHz spectrometers. The acquisition software and processing program used to analyze the data was TopSpin. Spectra of ^{1}H-NMR (300 MHz and 500 MHz), ^{13}C-NMR (125 MHz) and ^{19}F-NMR (470 MHz) were included in FID data.

<u>MolFile of Structure collected:</u>

<u>Compound **2**:</u>


```
ChemDraw07102421472D

13 12  0  0  0  0  0  0  0  0999 V2000
  -1.4289    0.4125    0.0000 O   0  0  0  0  0  0  0  0  0  0  0  0
  -0.7145   -0.0000    0.0000 C   0  0  0  0  0  0  0  0  0  0  0  0
   0.0000    0.4125    0.0000 C   0  0  0  0  0  0  0  0  0  0  0  0
   0.7145   -0.0000    0.0000 C   0  0  0  0  0  0  0  0  0  0  0  0
   1.4289    0.4125    0.0000 O   0  0  0  0  0  0  0  0  0  0  0  0
  -2.1434   -0.0000    0.0000 S   0  0  0  0  0  0  0  0  0  0  0  0
   2.1434   -0.0000    0.0000 S   0  0  0  0  0  0  0  0  0  0  0  0
   2.8579   -0.4125    0.0000 C   0  0  0  0  0  0  0  0  0  0  0  0
   1.7309   -0.7145    0.0000 O   0  0  0  0  0  0  0  0  0  0  0  0
   2.5559    0.7145    0.0000 O   0  0  0  0  0  0  0  0  0  0  0  0
  -2.8579   -0.4125    0.0000 C   0  0  0  0  0  0  0  0  0  0  0  0
  -1.7309   -0.7145    0.0000 O   0  0  0  0  0  0  0  0  0  0  0  0
  -2.5559    0.7145    0.0000 O   0  0  0  0  0  0  0  0  0  0  0  0
  1  2  1  0
  2  3  1  0
  3  4  1  0
  4  5  1  0
  1  6  1  0
  5  7  1  0
  7  8  1  0
  7  9  2  0
  7 10  2  0
```

```
  6 11  1  0
  6 12  2  0
  6 13  2  0
M  END
```

Compound **3**:

```
  ChemDraw07102421522D

 11 10  0  0  0  0  0  0  0  0999 V2000
   -1.4289    0.6187    0.0000 S   0  0  0  0  0  0  0  0  0  0  0  0
   -0.7145    0.2062    0.0000 C   0  0  0  0  0  0  0  0  0  0  0  0
    0.0000    0.6187    0.0000 C   0  0  0  0  0  0  0  0  0  0  0  0
    0.7145    0.2062    0.0000 C   0  0  0  0  0  0  0  0  0  0  0  0
    1.4289    0.6187    0.0000 S   0  0  0  0  0  0  0  0  0  0  0  0
    2.1434    0.2062    0.0000 C   0  0  0  0  0  0  0  0  0  0  0  0
    2.8579    0.6187    0.0000 C   0  0  0  0  0  0  0  0  0  0  0  0
    2.1434   -0.6187    0.0000 O   0  0  0  0  0  0  0  0  0  0  0  0
   -2.1434    0.2062    0.0000 C   0  0  0  0  0  0  0  0  0  0  0  0
   -2.8579    0.6187    0.0000 C   0  0  0  0  0  0  0  0  0  0  0  0
   -2.1434   -0.6187    0.0000 O   0  0  0  0  0  0  0  0  0  0  0  0
  1  2  1  0
  2  3  1  0
  3  4  1  0
  4  5  1  0
  5  6  1  0
  6  7  1  0
  6  8  2  0
  1  9  1  0
  9 10  1  0
  9 11  2  0
M  END
```

Compound **4**:

ChemDraw07102421532D

9 8 0 0 0 0 0 0 0 0999 V2000
-1.4289 -0.2063 0.0000 S 0 0 0 0 0 0 0 0 0 0 0 0
-0.7145 -0.6188 0.0000 C 0 0 0 0 0 0 0 0 0 0 0 0
0.0000 -0.2063 0.0000 C 0 0 0 0 0 0 0 0 0 0 0 0
0.7145 -0.6188 0.0000 C 0 0 0 0 0 0 0 0 0 0 0 0
1.4289 -0.2063 0.0000 S 0 0 0 0 0 0 0 0 0 0 0 0
-2.1434 -0.6188 0.0000 Cl 0 0 0 0 0 0 0 0 0 0 0 0
2.1434 -0.6188 0.0000 Cl 0 0 0 0 0 0 0 0 0 0 0 0
-1.4289 0.6188 0.0000 O 0 0 0 0 0 0 0 0 0 0 0 0
1.4289 0.6187 0.0000 O 0 0 0 0 0 0 0 0 0 0 0 0
1 2 1 0
2 3 1 0
3 4 1 0
4 5 1 0
1 6 1 0
5 7 1 0
1 8 1 4
5 9 1 4
M SUB 2 1 4 5 4
M END

Compound **5(*S*$_S$,*S*$_S$)**:

ChemDraw07102421542D

57 66 0 0 1 0 0 0 0 0999 V2000
-1.4451 -0.3991 0.0000 S 0 0 0 0 0 0 0 0 0 0 0 0
-0.7306 -0.8116 0.0000 C 0 0 0 0 0 0 0 0 0 0 0 0

-0.0161	-0.3991	0.0000	C	0	0	0	0	0	0	0	0	0	0	0	0
0.6984	-0.8116	0.0000	C	0	0	0	0	0	0	0	0	0	0	0	0
1.4128	-0.3991	0.0000	S	0	0	0	0	0	0	0	0	0	0	0	0
-2.1595	-0.8116	0.0000	O	0	0	0	0	0	0	0	0	0	0	0	0
2.1273	-0.8116	0.0000	O	0	0	0	0	0	0	0	0	0	0	0	0
-1.8232	0.3228	0.0000	C	0	0	0	0	0	0	0	0	0	0	0	0
0.9660	0.4259	0.0000	O	0	0	0	0	0	0	0	0	0	0	0	0
-1.1701	0.3498	0.0000	O	0	0	0	0	0	0	0	0	0	0	0	0
1.6878	0.3154	0.0000	C	0	0	0	0	0	0	0	0	0	0	0	0
3.9166	-0.3334	0.0000	C	0	0	0	0	0	0	0	0	0	0	0	0
2.9232	-0.3255	0.0000	C	0	0	0	0	0	0	0	0	0	0	0	0
2.6186	-0.0210	0.0000	C	0	0	0	0	0	0	0	0	0	0	0	0
3.4250	0.1588	0.0000	O	0	0	0	0	0	0	0	0	0	0	0	0
4.2272	-0.0337	0.0000	C	0	0	0	0	0	0	0	0	0	0	0	0
3.9166	-1.1584	0.0000	O	0	0	0	0	0	0	0	0	0	0	0	0
4.2345	-0.6931	0.0000	O	0	0	0	0	0	0	0	0	0	0	0	0
2.6186	0.8040	0.0000	C	0	0	0	0	0	0	0	0	0	0	0	0
2.0352	1.3874	0.0000	O	0	0	0	0	0	0	0	0	0	0	0	0
3.0311	1.5185	0.0000	C	0	0	0	0	0	0	0	0	0	0	0	0
2.6186	2.2330	0.0000	O	0	0	0	0	0	0	0	0	0	0	0	0
1.8217	2.1843	0.0000	C	0	0	0	0	0	0	0	0	0	0	0	0
1.9044	3.0051	0.0000	C	0	0	0	0	0	0	0	0	0	0	0	0
1.2349	3.4872	0.0000	C	0	0	0	0	0	0	0	0	0	0	0	0
0.4826	3.1484	0.0000	C	0	0	0	0	0	0	0	0	0	0	0	0
0.4000	2.3275	0.0000	C	0	0	0	0	0	0	0	0	0	0	0	0
1.0695	1.8455	0.0000	C	0	0	0	0	0	0	0	0	0	0	0	0
4.3291	-1.8729	0.0000	C	0	0	0	0	0	0	0	0	0	0	0	0
3.7155	-2.4243	0.0000	C	0	0	0	0	0	0	0	0	0	0	0	0
3.8862	-3.2315	0.0000	C	0	0	0	0	0	0	0	0	0	0	0	0
4.6706	-3.4872	0.0000	C	0	0	0	0	0	0	0	0	0	0	0	0
5.2842	-2.9357	0.0000	C	0	0	0	0	0	0	0	0	0	0	0	0

5.1135	-2.1286	0.0000	C	0	0	0	0	0	0	0	0	0	0	0	0
-2.9697	-0.3503	0.0000	C	0	0	0	0	0	0	0	0	0	0	0	0
-3.9631	-0.3503	0.0000	C	0	0	0	0	0	0	0	0	0	0	0	0
-4.2701	-0.0481	0.0000	C	0	0	0	0	0	0	0	0	0	0	0	0
-3.4652	0.1380	0.0000	O	0	0	0	0	0	0	0	0	0	0	0	0
-2.6615	-0.0481	0.0000	C	0	0	0	0	0	0	0	0	0	0	0	0
-3.9631	-1.1753	0.0000	O	0	0	0	0	0	0	0	0	0	0	0	0
-4.2701	-0.7356	0.0000	O	0	0	0	0	0	0	0	0	0	0	0	0
-2.6615	0.7769	0.0000	C	0	0	0	0	0	0	0	0	0	0	0	0
-1.9470	1.1894	0.0000	O	0	0	0	0	0	0	0	0	0	0	0	0
-2.8750	1.5738	0.0000	C	0	0	0	0	0	0	0	0	0	0	0	0
-2.2916	2.1572	0.0000	O	0	0	0	0	0	0	0	0	0	0	0	0
-1.4666	2.1572	0.0000	C	0	0	0	0	0	0	0	0	0	0	0	0
-0.7277	1.7904	0.0000	C	0	0	0	0	0	0	0	0	0	0	0	0
-0.0405	2.2469	0.0000	C	0	0	0	0	0	0	0	0	0	0	0	0
-0.0923	3.0703	0.0000	C	0	0	0	0	0	0	0	0	0	0	0	0
-0.8313	3.4371	0.0000	C	0	0	0	0	0	0	0	0	0	0	0	0
-1.5185	2.9805	0.0000	C	0	0	0	0	0	0	0	0	0	0	0	0
-4.2701	-1.6981	0.0000	C	0	0	0	0	0	0	0	0	0	0	0	0
-5.0678	-1.9087	0.0000	C	0	0	0	0	0	0	0	0	0	0	0	0
-5.2842	-2.7048	0.0000	C	0	0	0	0	0	0	0	0	0	0	0	0
-4.7030	-3.2903	0.0000	C	0	0	0	0	0	0	0	0	0	0	0	0
-3.9053	-3.0797	0.0000	C	0	0	0	0	0	0	0	0	0	0	0	0
-3.6889	-2.2836	0.0000	C	0	0	0	0	0	0	0	0	0	0	0	0

1	2	1	0
2	3	1	0
3	4	1	0
4	5	1	0
1	6	1	0
5	7	1	0
1	8	1	1

5	9	1	1
1	10	1	6
5	11	1	6
12	13	1	1
14	13	1	1
14	15	1	0
15	16	1	0
16	12	1	1
7	13	1	0
12	17	1	0
16	18	1	0
14	19	1	0
19	20	1	1
19	21	1	6
21	22	1	0
20	23	1	0
23	22	1	0
23	24	1	0
24	25	1	0
25	26	1	0
26	27	1	0
27	28	1	0
28	23	1	0
17	29	1	0
18	29	1	0
29	30	1	0
30	31	1	0
31	32	1	0
32	33	1	0
33	34	1	0
34	29	1	0

35 36 1 1

37 36 1 1

37 38 1 0

38 39 1 0

39 35 1 1

6 35 1 0

36 40 1 0

37 41 1 0

39 42 1 0

42 43 1 1

42 44 1 6

44 45 1 0

45 46 1 0

43 46 1 0

46 47 1 0

47 48 1 0

48 49 1 0

49 50 1 0

50 51 1 0

51 46 1 0

41 52 1 0

40 52 1 0

52 53 1 0

53 54 1 0

54 55 1 0

55 56 1 0

56 57 1 0

57 52 1 0

M RAD 2   8   1 11   1

M END

Compound **5(*R*$_S$,*R*$_S$)**:

ChemDraw07102421562D

```
57 66  0  0  1  0  0  0  0  0  0999 V2000
   -1.4451   -0.3991    0.0000 S   0  0  0  0  0  0  0  0  0  0  0  0
   -0.7306   -0.8116    0.0000 C   0  0  0  0  0  0  0  0  0  0  0  0
   -0.0161   -0.3991    0.0000 C   0  0  0  0  0  0  0  0  0  0  0  0
    0.6984   -0.8116    0.0000 C   0  0  0  0  0  0  0  0  0  0  0  0
    1.4128   -0.3991    0.0000 S   0  0  0  0  0  0  0  0  0  0  0  0
   -2.1595   -0.8116    0.0000 O   0  0  0  0  0  0  0  0  0  0  0  0
    2.1273   -0.8116    0.0000 O   0  0  0  0  0  0  0  0  0  0  0  0
   -1.7201    0.4603    0.0000 O   0  0  0  0  0  0  0  0  0  0  0  0
    1.0347    0.3915    0.0000 C   0  0  0  0  0  0  0  0  0  0  0  0
   -1.1701    0.3498    0.0000 C   0  0  0  0  0  0  0  0  0  0  0  0
    1.6191    0.3841    0.0000 O   0  0  0  0  0  0  0  0  0  0  0  0
    3.9166   -0.3334    0.0000 C   0  0  0  0  0  0  0  0  0  0  0  0
    2.9232   -0.3255    0.0000 C   0  0  0  0  0  0  0  0  0  0  0  0
    2.6186   -0.0210    0.0000 C   0  0  0  0  0  0  0  0  0  0  0  0
    3.4250    0.1588    0.0000 O   0  0  0  0  0  0  0  0  0  0  0  0
    4.2272   -0.0337    0.0000 C   0  0  0  0  0  0  0  0  0  0  0  0
    3.9166   -1.1584    0.0000 O   0  0  0  0  0  0  0  0  0  0  0  0
    4.2345   -0.6931    0.0000 O   0  0  0  0  0  0  0  0  0  0  0  0
    2.6186    0.8040    0.0000 C   0  0  0  0  0  0  0  0  0  0  0  0
    2.0352    1.3874    0.0000 O   0  0  0  0  0  0  0  0  0  0  0  0
    3.0311    1.5185    0.0000 C   0  0  0  0  0  0  0  0  0  0  0  0
    2.6186    2.2330    0.0000 O   0  0  0  0  0  0  0  0  0  0  0  0
    1.8217    2.1843    0.0000 C   0  0  0  0  0  0  0  0  0  0  0  0
    1.9044    3.0051    0.0000 C   0  0  0  0  0  0  0  0  0  0  0  0
    1.2349    3.4872    0.0000 C   0  0  0  0  0  0  0  0  0  0  0  0
    0.4826    3.1484    0.0000 C   0  0  0  0  0  0  0  0  0  0  0  0
    0.4000    2.3275    0.0000 C   0  0  0  0  0  0  0  0  0  0  0  0
```

1.0695	1.8455	0.0000	C	0	0	0	0	0	0	0	0	0	0	0	0
4.3291	-1.8729	0.0000	C	0	0	0	0	0	0	0	0	0	0	0	0
3.7155	-2.4243	0.0000	C	0	0	0	0	0	0	0	0	0	0	0	0
3.8862	-3.2315	0.0000	C	0	0	0	0	0	0	0	0	0	0	0	0
4.6706	-3.4872	0.0000	C	0	0	0	0	0	0	0	0	0	0	0	0
5.2842	-2.9357	0.0000	C	0	0	0	0	0	0	0	0	0	0	0	0
5.1135	-2.1286	0.0000	C	0	0	0	0	0	0	0	0	0	0	0	0
-2.9697	-0.3503	0.0000	C	0	0	0	0	0	0	0	0	0	0	0	0
-3.9631	-0.3503	0.0000	C	0	0	0	0	0	0	0	0	0	0	0	0
-4.2701	-0.0481	0.0000	C	0	0	0	0	0	0	0	0	0	0	0	0
-3.4652	0.1380	0.0000	O	0	0	0	0	0	0	0	0	0	0	0	0
-2.6615	-0.0481	0.0000	C	0	0	0	0	0	0	0	0	0	0	0	0
-3.9631	-1.1753	0.0000	O	0	0	0	0	0	0	0	0	0	0	0	0
-4.2701	-0.7356	0.0000	O	0	0	0	0	0	0	0	0	0	0	0	0
-2.6615	0.7769	0.0000	C	0	0	0	0	0	0	0	0	0	0	0	0
-1.9470	1.1894	0.0000	O	0	0	0	0	0	0	0	0	0	0	0	0
-2.8750	1.5738	0.0000	C	0	0	0	0	0	0	0	0	0	0	0	0
-2.2916	2.1572	0.0000	O	0	0	0	0	0	0	0	0	0	0	0	0
-1.4666	2.1572	0.0000	C	0	0	0	0	0	0	0	0	0	0	0	0
-0.7277	1.7904	0.0000	C	0	0	0	0	0	0	0	0	0	0	0	0
-0.0405	2.2469	0.0000	C	0	0	0	0	0	0	0	0	0	0	0	0
-0.0923	3.0703	0.0000	C	0	0	0	0	0	0	0	0	0	0	0	0
-0.8313	3.4371	0.0000	C	0	0	0	0	0	0	0	0	0	0	0	0
-1.5185	2.9805	0.0000	C	0	0	0	0	0	0	0	0	0	0	0	0
-4.2701	-1.6981	0.0000	C	0	0	0	0	0	0	0	0	0	0	0	0
-5.0678	-1.9087	0.0000	C	0	0	0	0	0	0	0	0	0	0	0	0
-5.2842	-2.7048	0.0000	C	0	0	0	0	0	0	0	0	0	0	0	0
-4.7030	-3.2903	0.0000	C	0	0	0	0	0	0	0	0	0	0	0	0
-3.9053	-3.0797	0.0000	C	0	0	0	0	0	0	0	0	0	0	0	0
-3.6889	-2.2836	0.0000	C	0	0	0	0	0	0	0	0	0	0	0	0

1 2 1 0

2 3 1 0
3 4 1 0
4 5 1 0
1 6 1 0
5 7 1 0
1 8 1 1
5 9 1 1
1 10 1 6
5 11 1 6
12 13 1 1
14 13 1 1
14 15 1 0
15 16 1 0
16 12 1 1
7 13 1 0
12 17 1 0
16 18 1 0
14 19 1 0
19 20 1 1
19 21 1 6
21 22 1 0
20 23 1 0
23 22 1 0
23 24 1 0
24 25 1 0
25 26 1 0
26 27 1 0
27 28 1 0
28 23 1 0
17 29 1 0
18 29 1 0

29 30 1 0

30 31 1 0

31 32 1 0

32 33 1 0

33 34 1 0

34 29 1 0

35 36 1 1

37 36 1 1

37 38 1 0

38 39 1 0

39 35 1 1

6 35 1 0

36 40 1 0

37 41 1 0

39 42 1 0

42 43 1 1

42 44 1 6

44 45 1 0

45 46 1 0

43 46 1 0

46 47 1 0

47 48 1 0

48 49 1 0

49 50 1 0

50 51 1 0

51 46 1 0

41 52 1 0

40 52 1 0

52 53 1 0

53 54 1 0

54 55 1 0

55 56 1 0

56 57 1 0

57 52 1 0

M RAD 2   9   1 10   1

M END

Compound **5(*R*S,*S*S)**:

ChemDraw07102421572D

```
 57 66  0  0  1  0  0  0  0  0999 V2000
   -1.4451   -0.3991    0.0000 S   0  0  0  0  0  0  0  0  0  0  0  0
   -0.7306   -0.8116    0.0000 C   0  0  0  0  0  0  0  0  0  0  0  0
   -0.0161   -0.3991    0.0000 C   0  0  0  0  0  0  0  0  0  0  0  0
    0.6984   -0.8116    0.0000 C   0  0  0  0  0  0  0  0  0  0  0  0
    1.4128   -0.3991    0.0000 S   0  0  0  0  0  0  0  0  0  0  0  0
   -2.1595   -0.8116    0.0000 O   0  0  0  0  0  0  0  0  0  0  0  0
    2.1273   -0.8116    0.0000 O   0  0  0  0  0  0  0  0  0  0  0  0
   -1.7201    0.4603    0.0000 O   0  0  0  0  0  0  0  0  0  0  0  0
    1.0347    0.3915    0.0000 O   0  0  0  0  0  0  0  0  0  0  0  0
   -1.1701    0.3498    0.0000 C   0  0  0  0  0  0  0  0  0  0  0  0
    1.6191    0.3841    0.0000 C   0  0  0  0  0  0  0  0  0  0  0  0
    3.9166   -0.3334    0.0000 C   0  0  0  0  0  0  0  0  0  0  0  0
    2.9232   -0.3255    0.0000 C   0  0  0  0  0  0  0  0  0  0  0  0
    2.6186   -0.0210    0.0000 C   0  0  0  0  0  0  0  0  0  0  0  0
    3.4250    0.1588    0.0000 O   0  0  0  0  0  0  0  0  0  0  0  0
    4.2272   -0.0337    0.0000 C   0  0  0  0  0  0  0  0  0  0  0  0
    3.9166   -1.1584    0.0000 O   0  0  0  0  0  0  0  0  0  0  0  0
    4.2345   -0.6931    0.0000 O   0  0  0  0  0  0  0  0  0  0  0  0
    2.6186    0.8040    0.0000 C   0  0  0  0  0  0  0  0  0  0  0  0
    2.0352    1.3874    0.0000 O   0  0  0  0  0  0  0  0  0  0  0  0
    3.0311    1.5185    0.0000 C   0  0  0  0  0  0  0  0  0  0  0  0
```

2.6186	2.2330	0.0000	O	0	0	0	0	0	0	0	0	0	0	0	0
1.8217	2.1843	0.0000	C	0	0	0	0	0	0	0	0	0	0	0	0
1.9044	3.0051	0.0000	C	0	0	0	0	0	0	0	0	0	0	0	0
1.2349	3.4872	0.0000	C	0	0	0	0	0	0	0	0	0	0	0	0
0.4826	3.1484	0.0000	C	0	0	0	0	0	0	0	0	0	0	0	0
0.4000	2.3275	0.0000	C	0	0	0	0	0	0	0	0	0	0	0	0
1.0695	1.8455	0.0000	C	0	0	0	0	0	0	0	0	0	0	0	0
4.3291	-1.8729	0.0000	C	0	0	0	0	0	0	0	0	0	0	0	0
3.7155	-2.4243	0.0000	C	0	0	0	0	0	0	0	0	0	0	0	0
3.8862	-3.2315	0.0000	C	0	0	0	0	0	0	0	0	0	0	0	0
4.6706	-3.4872	0.0000	C	0	0	0	0	0	0	0	0	0	0	0	0
5.2842	-2.9357	0.0000	C	0	0	0	0	0	0	0	0	0	0	0	0
5.1135	-2.1286	0.0000	C	0	0	0	0	0	0	0	0	0	0	0	0
-2.9697	-0.3503	0.0000	C	0	0	0	0	0	0	0	0	0	0	0	0
-3.9631	-0.3503	0.0000	C	0	0	0	0	0	0	0	0	0	0	0	0
-4.2701	-0.0481	0.0000	C	0	0	0	0	0	0	0	0	0	0	0	0
-3.4652	0.1380	0.0000	O	0	0	0	0	0	0	0	0	0	0	0	0
-2.6615	-0.0481	0.0000	C	0	0	0	0	0	0	0	0	0	0	0	0
-3.9631	-1.1753	0.0000	O	0	0	0	0	0	0	0	0	0	0	0	0
-4.2701	-0.7356	0.0000	O	0	0	0	0	0	0	0	0	0	0	0	0
-2.6615	0.7769	0.0000	C	0	0	0	0	0	0	0	0	0	0	0	0
-1.9470	1.1894	0.0000	O	0	0	0	0	0	0	0	0	0	0	0	0
-2.8750	1.5738	0.0000	C	0	0	0	0	0	0	0	0	0	0	0	0
-2.2916	2.1572	0.0000	O	0	0	0	0	0	0	0	0	0	0	0	0
-1.4666	2.1572	0.0000	C	0	0	0	0	0	0	0	0	0	0	0	0
-0.7277	1.7904	0.0000	C	0	0	0	0	0	0	0	0	0	0	0	0
-0.0405	2.2469	0.0000	C	0	0	0	0	0	0	0	0	0	0	0	0
-0.0923	3.0703	0.0000	C	0	0	0	0	0	0	0	0	0	0	0	0
-0.8313	3.4371	0.0000	C	0	0	0	0	0	0	0	0	0	0	0	0
-1.5185	2.9805	0.0000	C	0	0	0	0	0	0	0	0	0	0	0	0
-4.2701	-1.6981	0.0000	C	0	0	0	0	0	0	0	0	0	0	0	0

-5.0678 -1.9087 0.0000 C 0 0 0 0 0 0 0 0 0 0 0 0
-5.2842 -2.7048 0.0000 C 0 0 0 0 0 0 0 0 0 0 0 0
-4.7030 -3.2903 0.0000 C 0 0 0 0 0 0 0 0 0 0 0 0
-3.9053 -3.0797 0.0000 C 0 0 0 0 0 0 0 0 0 0 0 0
-3.6889 -2.2836 0.0000 C 0 0 0 0 0 0 0 0 0 0 0 0
1 2 1 0
2 3 1 0
3 4 1 0
4 5 1 0
1 6 1 0
5 7 1 0
1 8 1 1
5 9 1 1
1 10 1 6
5 11 1 6
12 13 1 1
14 13 1 1
14 15 1 0
15 16 1 0
16 12 1 1
7 13 1 0
12 17 1 0
16 18 1 0
14 19 1 0
19 20 1 1
19 21 1 6
21 22 1 0
20 23 1 0
23 22 1 0
23 24 1 0
24 25 1 0

25 26 1 0

26 27 1 0

27 28 1 0

28 23 1 0

17 29 1 0

18 29 1 0

29 30 1 0

30 31 1 0

31 32 1 0

32 33 1 0

33 34 1 0

34 29 1 0

35 36 1 1

37 36 1 1

37 38 1 0

38 39 1 0

39 35 1 1

6 35 1 0

36 40 1 0

37 41 1 0

39 42 1 0

42 43 1 1

42 44 1 6

44 45 1 0

45 46 1 0

43 46 1 0

46 47 1 0

47 48 1 0

48 49 1 0

49 50 1 0

50 51 1 0

51 46 1 0

41 52 1 0

40 52 1 0

52 53 1 0

53 54 1 0

54 55 1 0

55 56 1 0

56 57 1 0

57 52 1 0

M RAD 2 10   1 11   1

M END

Compound **8(*S*S,*S*S)**:

ChemDraw07102421592D

21 22 0 0 1 0 0 0 0 0 0999 V2000

-1.4289   0.3990   0.0000 S   0 0 0 0 0 0 0 0 0 0 0 0

-0.7145   -0.0135   0.0000 C   0 0 0 0 0 0 0 0 0 0 0 0

0.0000   0.3990   0.0000 C   0 0 0 0 0 0 0 0 0 0 0 0

0.7145   -0.0135   0.0000 C   0 0 0 0 0 0 0 0 0 0 0 0

1.4289   0.3990   0.0000 S   0 0 0 0 0 0 0 0 0 0 0 0

-2.1434   -0.0135   0.0000 C   0 0 0 0 0 0 0 0 0 0 0 0

2.1434   -0.0135   0.0000 C   0 0 0 0 0 0 0 0 0 0 0 0

-1.8758   1.2240   0.0000 O   0 0 0 0 0 0 0 0 0 0 0 0

0.9821   1.2240   0.0000 C   0 0 0 0 0 0 0 0 0 0 0 0

-1.0508   1.2166   0.0000 C   0 0 0 0 0 0 0 0 0 0 0 0

1.7727   1.2510   0.0000 O   0 0 0 0 0 0 0 0 0 0 0 0

2.1434   -0.8385   0.0000 C   0 0 0 0 0 0 0 0 0 0 0 0

2.8579   -1.2510   0.0000 C   0 0 0 0 0 0 0 0 0 0 0 0

```
    3.5724   -0.8385    0.0000 C   0  0  0  0  0  0  0  0  0  0  0  0
    3.5724   -0.0135    0.0000 C   0  0  0  0  0  0  0  0  0  0  0  0
    2.8579    0.3990    0.0000 C   0  0  0  0  0  0  0  0  0  0  0  0
   -2.8579    0.3990    0.0000 C   0  0  0  0  0  0  0  0  0  0  0  0
   -3.5724   -0.0135    0.0000 C   0  0  0  0  0  0  0  0  0  0  0  0
   -3.5724   -0.8385    0.0000 C   0  0  0  0  0  0  0  0  0  0  0  0
   -2.8579   -1.2510    0.0000 C   0  0  0  0  0  0  0  0  0  0  0  0
   -2.1434   -0.8385    0.0000 C   0  0  0  0  0  0  0  0  0  0  0  0
  1  2  1  0
  2  3  1  0
  3  4  1  0
  4  5  1  0
  1  6  1  0
  5  7  1  0
  1  8  1  1
  5  9  1  1
  1 10  1  6
  5 11  1  6
  7 12  2  0
 12 13  1  0
 13 14  2  0
 14 15  1  0
 15 16  2  0
 16  7  1  0
  6 17  2  0
 17 18  1  0
 18 19  2  0
 19 20  1  0
 20 21  2  0
 21  6  1  0
M  RAD  2   9   1  10   1
```

```
M  END
```

<u>Compound **8(*rac*+*meso*)**:</u>

```
  ChemDraw07102422002D

 19 20  0  0  0  0  0  0  0  0999 V2000
   -1.4289    0.4125    0.0000 S   0  0  0  0  0  0  0  0  0  0  0  0
   -0.7145   -0.0000    0.0000 C   0  0  0  0  0  0  0  0  0  0  0  0
    0.0000    0.4125    0.0000 C   0  0  0  0  0  0  0  0  0  0  0  0
    0.7145   -0.0000    0.0000 C   0  0  0  0  0  0  0  0  0  0  0  0
    1.4289    0.4125    0.0000 S   0  0  0  0  0  0  0  0  0  0  0  0
   -2.1434   -0.0000    0.0000 C   0  0  0  0  0  0  0  0  0  0  0  0
    2.1434   -0.0000    0.0000 C   0  0  0  0  0  0  0  0  0  0  0  0
    2.1434   -0.8250    0.0000 C   0  0  0  0  0  0  0  0  0  0  0  0
    2.8579   -1.2375    0.0000 C   0  0  0  0  0  0  0  0  0  0  0  0
    3.5724   -0.8250    0.0000 C   0  0  0  0  0  0  0  0  0  0  0  0
    3.5724   -0.0000    0.0000 C   0  0  0  0  0  0  0  0  0  0  0  0
    2.8579    0.4125    0.0000 C   0  0  0  0  0  0  0  0  0  0  0  0
   -2.8579    0.4125    0.0000 C   0  0  0  0  0  0  0  0  0  0  0  0
   -3.5724   -0.0000    0.0000 C   0  0  0  0  0  0  0  0  0  0  0  0
   -3.5724   -0.8250    0.0000 C   0  0  0  0  0  0  0  0  0  0  0  0
   -2.8579   -1.2375    0.0000 C   0  0  0  0  0  0  0  0  0  0  0  0
   -2.1434   -0.8250    0.0000 C   0  0  0  0  0  0  0  0  0  0  0  0
   -1.4289    1.2375    0.0000 O   0  0  0  0  0  0  0  0  0  0  0  0
    1.4289    1.2375    0.0000 O   0  0  0  0  0  0  0  0  0  0  0  0
  1  2  1  0
  2  3  1  0
  3  4  1  0
  4  5  1  0
  1  6  1  0
  5  7  1  0
```

 7 8 2 0
 8 9 1 0
 9 10 2 0
10 11 1 0
11 12 2 0
12 7 1 0
 6 13 2 0
13 14 1 0
14 15 2 0
15 16 1 0
16 17 2 0
17 6 1 0
 1 18 1 4
 5 19 1 4
M SUB 2   1   4   5   4
M END

<u>Compound **9(*S*$_S$,*S*$_S$)**:</u>

ChemDraw07102422012D

29 32 0 0 1 0 0 0 0 0999 V2000
-1.4289   0.2062   0.0000 S   0 0 0 0 0 0 0 0 0 0 0 0
-0.7145   -0.2063   0.0000 C   0 0 0 0 0 0 0 0 0 0 0 0
0.0000   0.2062   0.0000 C   0 0 0 0 0 0 0 0 0 0 0 0
0.7145   -0.2063   0.0000 C   0 0 0 0 0 0 0 0 0 0 0 0
1.4289   0.2062   0.0000 S   0 0 0 0 0 0 0 0 0 0 0 0
-2.1434   -0.2063   0.0000 C   0 0 0 0 0 0 0 0 0 0 0 0
2.1434   -0.2063   0.0000 C   0 0 0 0 0 0 0 0 0 0 0 0
-1.8758   1.0312   0.0000 O   0 0 0 0 0 0 0 0 0 0 0 0
0.9821   1.0312   0.0000 C   0 0 0 0 0 0 0 0 0 0 0 0
-1.0508   1.0238   0.0000 C   0 0 0 0 0 0 0 0 0 0 0 0

1.7727	1.0582	0.0000	O	0	0	0	0	0	0	0	0	0	0	0	0
2.1434	-1.0313	0.0000	C	0	0	0	0	0	0	0	0	0	0	0	0
2.8579	-1.4438	0.0000	C	0	0	0	0	0	0	0	0	0	0	0	0
3.5724	-1.0313	0.0000	C	0	0	0	0	0	0	0	0	0	0	0	0
3.5724	-0.2063	0.0000	C	0	0	0	0	0	0	0	0	0	0	0	0
2.8579	0.2062	0.0000	C	0	0	0	0	0	0	0	0	0	0	0	0
-2.8579	0.2062	0.0000	C	0	0	0	0	0	0	0	0	0	0	0	0
-3.5724	-0.2063	0.0000	C	0	0	0	0	0	0	0	0	0	0	0	0
-3.5724	-1.0313	0.0000	C	0	0	0	0	0	0	0	0	0	0	0	0
-2.8579	-1.4438	0.0000	C	0	0	0	0	0	0	0	0	0	0	0	0
-2.1434	-1.0313	0.0000	C	0	0	0	0	0	0	0	0	0	0	0	0
4.2868	0.2062	0.0000	C	0	0	0	0	0	0	0	0	0	0	0	0
4.2868	1.0313	0.0000	C	0	0	0	0	0	0	0	0	0	0	0	0
3.5724	1.4438	0.0000	C	0	0	0	0	0	0	0	0	0	0	0	0
2.8579	1.0313	0.0000	C	0	0	0	0	0	0	0	0	0	0	0	0
-2.8579	1.0313	0.0000	C	0	0	0	0	0	0	0	0	0	0	0	0
-3.5724	1.4438	0.0000	C	0	0	0	0	0	0	0	0	0	0	0	0
-4.2868	1.0313	0.0000	C	0	0	0	0	0	0	0	0	0	0	0	0
-4.2868	0.2062	0.0000	C	0	0	0	0	0	0	0	0	0	0	0	0

1	2	1	0
2	3	1	0
3	4	1	0
4	5	1	0
1	6	1	0
5	7	1	0
1	8	1	1
5	9	1	1
1	10	1	6
5	11	1	6
7	12	2	0
12	13	1	0

13 14 2 0

14 15 1 0

15 16 2 0

16 7 1 0

6 17 2 0

17 18 1 0

18 19 2 0

19 20 1 0

20 21 2 0

21 6 1 0

15 22 1 0

22 23 2 0

23 24 1 0

24 25 2 0

25 16 1 0

17 26 1 0

26 27 2 0

27 28 1 0

28 29 2 0

29 18 1 0

M RAD 2 9 1 10 1

M END

Compound **9(*rac*+*meso*)**:

ChemDraw07102422012D

27 30 0 0 0 0 0 0 0 0999 V2000

-1.4289 0.2062 0.0000 S 0 0 0 0 0 0 0 0 0 0 0 0

-0.7145 -0.2063 0.0000 C 0 0 0 0 0 0 0 0 0 0 0 0

0.0000 0.2062 0.0000 C 0 0 0 0 0 0 0 0 0 0 0 0

0.7145 -0.2063 0.0000 C 0 0 0 0 0 0 0 0 0 0 0 0

1.4289 0.2062 0.0000 S 0 0 0 0 0 0 0 0 0 0 0 0

-2.1434 -0.2063 0.0000 C 0 0 0 0 0 0 0 0 0 0 0 0

2.1434 -0.2063 0.0000 C 0 0 0 0 0 0 0 0 0 0 0 0

2.1434 -1.0313 0.0000 C 0 0 0 0 0 0 0 0 0 0 0 0

2.8579 -1.4438 0.0000 C 0 0 0 0 0 0 0 0 0 0 0 0

3.5724 -1.0313 0.0000 C 0 0 0 0 0 0 0 0 0 0 0 0

3.5724 -0.2063 0.0000 C 0 0 0 0 0 0 0 0 0 0 0 0

2.8579 0.2062 0.0000 C 0 0 0 0 0 0 0 0 0 0 0 0

-2.8579 0.2062 0.0000 C 0 0 0 0 0 0 0 0 0 0 0 0

-3.5724 -0.2063 0.0000 C 0 0 0 0 0 0 0 0 0 0 0 0

-3.5724 -1.0313 0.0000 C 0 0 0 0 0 0 0 0 0 0 0 0

-2.8579 -1.4438 0.0000 C 0 0 0 0 0 0 0 0 0 0 0 0

-2.1434 -1.0313 0.0000 C 0 0 0 0 0 0 0 0 0 0 0 0

-1.4289 1.0312 0.0000 O 0 0 0 0 0 0 0 0 0 0 0 0

1.4289 1.0312 0.0000 O 0 0 0 0 0 0 0 0 0 0 0 0

4.2868 0.2062 0.0000 C 0 0 0 0 0 0 0 0 0 0 0 0

4.2868 1.0313 0.0000 C 0 0 0 0 0 0 0 0 0 0 0 0

3.5724 1.4438 0.0000 C 0 0 0 0 0 0 0 0 0 0 0 0

2.8579 1.0313 0.0000 C 0 0 0 0 0 0 0 0 0 0 0 0

-2.8579 1.0313 0.0000 C 0 0 0 0 0 0 0 0 0 0 0 0

-3.5724 1.4438 0.0000 C 0 0 0 0 0 0 0 0 0 0 0 0

-4.2868 1.0313 0.0000 C 0 0 0 0 0 0 0 0 0 0 0 0

-4.2868 0.2062 0.0000 C 0 0 0 0 0 0 0 0 0 0 0 0

1 2 1 0

2 3 1 0

3 4 1 0

4 5 1 0

1 6 1 0

5 7 1 0

7 8 2 0

8 9 1 0

9 10 2 0

10 11 1 0

11 12 2 0

12 7 1 0

6 13 2 0

13 14 1 0

14 15 2 0

15 16 1 0

16 17 2 0

17 6 1 0

1 18 1 4

5 19 1 4

11 20 1 0

20 21 2 0

21 22 1 0

22 23 2 0

23 12 1 0

13 24 1 0

24 25 2 0

25 26 1 0

26 27 2 0

27 14 1 0

M SUB 2 1 4 5 4

M END

Compound **10(*S*$_S$,*S*$_S$)**:

ChemDraw07102422022D

25 26 0 0 1 0 0 0 0 0999 V2000

-1.4289 0.3990 0.0000 S 0 0 0 0 0 0 0 0 0 0 0 0

-0.7145 -0.0135 0.0000 C 0 0 0 0 0 0 0 0 0 0 0 0

0.0000	0.3990	0.0000	C	0	0	0	0	0	0	0	0	0	0	0	0
0.7145	-0.0135	0.0000	C	0	0	0	0	0	0	0	0	0	0	0	0
1.4289	0.3990	0.0000	S	0	0	0	0	0	0	0	0	0	0	0	0
-2.1434	-0.0135	0.0000	C	0	0	0	0	0	0	0	0	0	0	0	0
2.1434	-0.0135	0.0000	C	0	0	0	0	0	0	0	0	0	0	0	0
-1.8758	1.2240	0.0000	O	0	0	0	0	0	0	0	0	0	0	0	0
0.9821	1.2240	0.0000	C	0	0	0	0	0	0	0	0	0	0	0	0
-1.0508	1.2166	0.0000	C	0	0	0	0	0	0	0	0	0	0	0	0
1.7727	1.2510	0.0000	O	0	0	0	0	0	0	0	0	0	0	0	0
2.1434	-0.8385	0.0000	C	0	0	0	0	0	0	0	0	0	0	0	0
2.8579	-1.2510	0.0000	C	0	0	0	0	0	0	0	0	0	0	0	0
3.5724	-0.8385	0.0000	C	0	0	0	0	0	0	0	0	0	0	0	0
3.5724	-0.0135	0.0000	C	0	0	0	0	0	0	0	0	0	0	0	0
2.8579	0.3990	0.0000	C	0	0	0	0	0	0	0	0	0	0	0	0
-2.8579	0.3990	0.0000	C	0	0	0	0	0	0	0	0	0	0	0	0
-3.5724	-0.0135	0.0000	C	0	0	0	0	0	0	0	0	0	0	0	0
-3.5724	-0.8385	0.0000	C	0	0	0	0	0	0	0	0	0	0	0	0
-2.8579	-1.2510	0.0000	C	0	0	0	0	0	0	0	0	0	0	0	0
-2.1434	-0.8385	0.0000	C	0	0	0	0	0	0	0	0	0	0	0	0
-2.8579	1.2240	0.0000	C	0	0	0	0	0	0	0	0	0	0	0	0
-1.4289	-1.2510	0.0000	C	0	0	0	0	0	0	0	0	0	0	0	0
1.4289	-1.2510	0.0000	C	0	0	0	0	0	0	0	0	0	0	0	0
2.8579	1.2240	0.0000	C	0	0	0	0	0	0	0	0	0	0	0	0

1	2	1	0
2	3	1	0
3	4	1	0
4	5	1	0
1	6	1	0
5	7	1	0
1	8	1	1
5	9	1	1

```
  1 10  1  6
  5 11  1  6
  7 12  2  0
 12 13  1  0
 13 14  2  0
 14 15  1  0
 15 16  2  0
 16  7  1  0
  6 17  2  0
 17 18  1  0
 18 19  2  0
 19 20  1  0
 20 21  2  0
 21  6  1  0
 17 22  1  0
 21 23  1  0
 12 24  1  0
 16 25  1  0
M  RAD  2   9   1  10   1
M  END
```

Compound **10(*R*S,*R*S)**:

```
  ChemDraw07102422032D

 25 26  0  0  1  0  0  0  0  0  0999 V2000
   -1.4289    0.3990    0.0000 S   0  0  0  0  0  0  0  0  0  0  0  0
   -0.7145   -0.0135    0.0000 C   0  0  0  0  0  0  0  0  0  0  0  0
    0.0000    0.3990    0.0000 C   0  0  0  0  0  0  0  0  0  0  0  0
    0.7145   -0.0135    0.0000 C   0  0  0  0  0  0  0  0  0  0  0  0
    1.4289    0.3990    0.0000 S   0  0  0  0  0  0  0  0  0  0  0  0
   -2.1434   -0.0135    0.0000 C   0  0  0  0  0  0  0  0  0  0  0  0
```

2.1434	-0.0135	0.0000	C	0	0	0	0	0	0	0	0	0	0	0	0
-1.8758	1.2240	0.0000	C	0	0	0	0	0	0	0	0	0	0	0	0
0.9821	1.2240	0.0000	O	0	0	0	0	0	0	0	0	0	0	0	0
-1.0508	1.2166	0.0000	O	0	0	0	0	0	0	0	0	0	0	0	0
1.7727	1.2510	0.0000	C	0	0	0	0	0	0	0	0	0	0	0	0
2.1434	-0.8385	0.0000	C	0	0	0	0	0	0	0	0	0	0	0	0
2.8579	-1.2510	0.0000	C	0	0	0	0	0	0	0	0	0	0	0	0
3.5724	-0.8385	0.0000	C	0	0	0	0	0	0	0	0	0	0	0	0
3.5724	-0.0135	0.0000	C	0	0	0	0	0	0	0	0	0	0	0	0
2.8579	0.3990	0.0000	C	0	0	0	0	0	0	0	0	0	0	0	0
-2.8579	0.3990	0.0000	C	0	0	0	0	0	0	0	0	0	0	0	0
-3.5724	-0.0135	0.0000	C	0	0	0	0	0	0	0	0	0	0	0	0
-3.5724	-0.8385	0.0000	C	0	0	0	0	0	0	0	0	0	0	0	0
-2.8579	-1.2510	0.0000	C	0	0	0	0	0	0	0	0	0	0	0	0
-2.1434	-0.8385	0.0000	C	0	0	0	0	0	0	0	0	0	0	0	0
-2.8579	1.2240	0.0000	C	0	0	0	0	0	0	0	0	0	0	0	0
-1.4289	-1.2510	0.0000	C	0	0	0	0	0	0	0	0	0	0	0	0
1.4289	-1.2510	0.0000	C	0	0	0	0	0	0	0	0	0	0	0	0
2.8579	1.2240	0.0000	C	0	0	0	0	0	0	0	0	0	0	0	0

1	2	1	0
2	3	1	0
3	4	1	0
4	5	1	0
1	6	1	0
5	7	1	0
1	8	1	1
5	9	1	1
1	10	1	6
5	11	1	6
7	12	2	0
12	13	1	0

13 14 2 0

14 15 1 0

15 16 2 0

16 7 1 0

6 17 2 0

17 18 1 0

18 19 2 0

19 20 1 0

20 21 2 0

21 6 1 0

17 22 1 0

21 23 1 0

12 24 1 0

16 25 1 0

M RAD 2   8   1 11   1

M END

<u>Compound **10(*rac*+*meso*)**:</u>

ChemDraw07102422042D

23 24 0 0 0 0 0 0 0 0999 V2000

-1.4289   0.4125   0.0000 S   0 0 0 0 0 0 0 0 0 0 0 0

-0.7145   -0.0000   0.0000 C   0 0 0 0 0 0 0 0 0 0 0 0

0.0000   0.4125   0.0000 C   0 0 0 0 0 0 0 0 0 0 0 0

0.7145   -0.0000   0.0000 C   0 0 0 0 0 0 0 0 0 0 0 0

1.4289   0.4125   0.0000 S   0 0 0 0 0 0 0 0 0 0 0 0

-2.1434   -0.0000   0.0000 C   0 0 0 0 0 0 0 0 0 0 0 0

2.1434   -0.0000   0.0000 C   0 0 0 0 0 0 0 0 0 0 0 0

2.1434   -0.8250   0.0000 C   0 0 0 0 0 0 0 0 0 0 0 0

2.8579   -1.2375   0.0000 C   0 0 0 0 0 0 0 0 0 0 0 0

3.5724   -0.8250   0.0000 C   0 0 0 0 0 0 0 0 0 0 0 0

3.5724	-0.0000	0.0000	C	0	0	0	0	0	0	0	0	0	0	0	0
2.8579	0.4125	0.0000	C	0	0	0	0	0	0	0	0	0	0	0	0
-2.8579	0.4125	0.0000	C	0	0	0	0	0	0	0	0	0	0	0	0
-3.5724	-0.0000	0.0000	C	0	0	0	0	0	0	0	0	0	0	0	0
-3.5724	-0.8250	0.0000	C	0	0	0	0	0	0	0	0	0	0	0	0
-2.8579	-1.2375	0.0000	C	0	0	0	0	0	0	0	0	0	0	0	0
-2.1434	-0.8250	0.0000	C	0	0	0	0	0	0	0	0	0	0	0	0
-1.4289	1.2375	0.0000	O	0	0	0	0	0	0	0	0	0	0	0	0
1.4289	1.2375	0.0000	O	0	0	0	0	0	0	0	0	0	0	0	0
-2.8579	1.2375	0.0000	C	0	0	0	0	0	0	0	0	0	0	0	0
-1.4289	-1.2375	0.0000	C	0	0	0	0	0	0	0	0	0	0	0	0
1.4289	-1.2375	0.0000	C	0	0	0	0	0	0	0	0	0	0	0	0
2.8579	1.2375	0.0000	C	0	0	0	0	0	0	0	0	0	0	0	0

1	2	1	0
2	3	1	0
3	4	1	0
4	5	1	0
1	6	1	0
5	7	1	0
7	8	2	0
8	9	1	0
9	10	2	0
10	11	1	0
11	12	2	0
12	7	1	0
6	13	2	0
13	14	1	0
14	15	2	0
15	16	1	0
16	17	2	0
17	6	1	0

1 18 1 4

5 19 1 4

13 20 1 0

17 21 1 0

8 22 1 0

12 23 1 0

M SUB 2   1   4   5   4

M END

Compound **11(*S*S,*S*S)**:

ChemDraw07102422052D

```
 23 24  0  0  1  0  0  0  0  0999 V2000
   -1.4289    0.3990    0.0000 S   0  0  0  0  0  0  0  0  0  0  0  0
   -0.7145   -0.0135    0.0000 C   0  0  0  0  0  0  0  0  0  0  0  0
    0.0000    0.3990    0.0000 C   0  0  0  0  0  0  0  0  0  0  0  0
    0.7145   -0.0135    0.0000 C   0  0  0  0  0  0  0  0  0  0  0  0
    1.4289    0.3990    0.0000 S   0  0  0  0  0  0  0  0  0  0  0  0
   -2.1434   -0.0135    0.0000 C   0  0  0  0  0  0  0  0  0  0  0  0
    2.1434   -0.0135    0.0000 C   0  0  0  0  0  0  0  0  0  0  0  0
   -1.8758    1.2240    0.0000 O   0  0  0  0  0  0  0  0  0  0  0  0
    0.9821    1.2240    0.0000 C   0  0  0  0  0  0  0  0  0  0  0  0
   -1.0508    1.2166    0.0000 C   0  0  0  0  0  0  0  0  0  0  0  0
    1.7727    1.2510    0.0000 O   0  0  0  0  0  0  0  0  0  0  0  0
    2.1434   -0.8385    0.0000 C   0  0  0  0  0  0  0  0  0  0  0  0
    2.8579   -1.2510    0.0000 C   0  0  0  0  0  0  0  0  0  0  0  0
    3.5724   -0.8385    0.0000 C   0  0  0  0  0  0  0  0  0  0  0  0
    3.5724   -0.0135    0.0000 C   0  0  0  0  0  0  0  0  0  0  0  0
    2.8579    0.3990    0.0000 C   0  0  0  0  0  0  0  0  0  0  0  0
   -2.8579    0.3990    0.0000 C   0  0  0  0  0  0  0  0  0  0  0  0
   -3.5724   -0.0135    0.0000 C   0  0  0  0  0  0  0  0  0  0  0  0
```

```
  -3.5724   -0.8385    0.0000 C   0  0  0  0  0  0  0  0  0  0  0  0
  -2.8579   -1.2510    0.0000 C   0  0  0  0  0  0  0  0  0  0  0  0
  -2.1434   -0.8385    0.0000 C   0  0  0  0  0  0  0  0  0  0  0  0
   4.2868   -1.2510    0.0000 C   0  0  0  0  0  0  0  0  0  0  0  0
  -4.2868   -1.2510    0.0000 C   0  0  0  0  0  0  0  0  0  0  0  0
  1  2  1  0
  2  3  1  0
  3  4  1  0
  4  5  1  0
  1  6  1  0
  5  7  1  0
  1  8  1  1
  5  9  1  1
  1 10  1  6
  5 11  1  6
  7 12  2  0
 12 13  1  0
 13 14  2  0
 14 15  1  0
 15 16  2  0
 16  7  1  0
  6 17  2  0
 17 18  1  0
 18 19  2  0
 19 20  1  0
 20 21  2  0
 21  6  1  0
 14 22  1  0
 19 23  1  0
M  RAD  2   9   1  10   1
M  END
```

Compound **11(*rac*+*meso*)**:


```
ChemDraw07102422062D

21 22  0  0  0  0  0  0  0  0999 V2000
   -1.4289    0.4125    0.0000 S   0  0  0  0  0  0  0  0  0  0  0  0
   -0.7145   -0.0000    0.0000 C   0  0  0  0  0  0  0  0  0  0  0  0
    0.0000    0.4125    0.0000 C   0  0  0  0  0  0  0  0  0  0  0  0
    0.7145   -0.0000    0.0000 C   0  0  0  0  0  0  0  0  0  0  0  0
    1.4289    0.4125    0.0000 S   0  0  0  0  0  0  0  0  0  0  0  0
   -2.1434   -0.0000    0.0000 C   0  0  0  0  0  0  0  0  0  0  0  0
    2.1434   -0.0000    0.0000 C   0  0  0  0  0  0  0  0  0  0  0  0
    2.1434   -0.8250    0.0000 C   0  0  0  0  0  0  0  0  0  0  0  0
    2.8579   -1.2375    0.0000 C   0  0  0  0  0  0  0  0  0  0  0  0
    3.5724   -0.8250    0.0000 C   0  0  0  0  0  0  0  0  0  0  0  0
    3.5724   -0.0000    0.0000 C   0  0  0  0  0  0  0  0  0  0  0  0
    2.8579    0.4125    0.0000 C   0  0  0  0  0  0  0  0  0  0  0  0
   -2.8579    0.4125    0.0000 C   0  0  0  0  0  0  0  0  0  0  0  0
   -3.5724   -0.0000    0.0000 C   0  0  0  0  0  0  0  0  0  0  0  0
   -3.5724   -0.8250    0.0000 C   0  0  0  0  0  0  0  0  0  0  0  0
   -2.8579   -1.2375    0.0000 C   0  0  0  0  0  0  0  0  0  0  0  0
   -2.1434   -0.8250    0.0000 C   0  0  0  0  0  0  0  0  0  0  0  0
   -1.4289    1.2375    0.0000 O   0  0  0  0  0  0  0  0  0  0  0  0
    1.4289    1.2375    0.0000 O   0  0  0  0  0  0  0  0  0  0  0  0
    4.2868   -1.2375    0.0000 C   0  0  0  0  0  0  0  0  0  0  0  0
   -4.2868   -1.2375    0.0000 C   0  0  0  0  0  0  0  0  0  0  0  0
  1  2  1  0
  2  3  1  0
  3  4  1  0
  4  5  1  0
  1  6  1  0
```

```
  5  7  1  0
  7  8  2  0
  8  9  1  0
  9 10  2  0
 10 11  1  0
 11 12  2  0
 12  7  1  0
  6 13  2  0
 13 14  1  0
 14 15  2  0
 15 16  1  0
 16 17  2  0
 17  6  1  0
  1 18  1  4
  5 19  1  4
 10 20  1  0
 15 21  1  0
M  SUB  2   1   4   5   4
M  END
```

<u>Compound **12(*S*S,*S*S)**:</u>

```
  ChemDraw07102422072D

 23 24  0  0  1  0  0  0  0  0999 V2000
   -1.4289   -0.4125    0.0000 S   0  0  0  0  0  0  0  0  0  0  0  0
   -0.7145   -0.8250    0.0000 C   0  0  0  0  0  0  0  0  0  0  0  0
    0.0000   -0.4125    0.0000 C   0  0  0  0  0  0  0  0  0  0  0  0
    0.7145   -0.8250    0.0000 C   0  0  0  0  0  0  0  0  0  0  0  0
    1.4289   -0.4125    0.0000 S   0  0  0  0  0  0  0  0  0  0  0  0
   -2.1434   -0.8250    0.0000 C   0  0  0  0  0  0  0  0  0  0  0  0
    2.1434   -0.8250    0.0000 C   0  0  0  0  0  0  0  0  0  0  0  0
```

-1.8758	0.4125	0.0000	O	0	0	0	0	0	0	0	0	0	0	0	0
0.9821	0.4125	0.0000	C	0	0	0	0	0	0	0	0	0	0	0	0
-1.0508	0.4051	0.0000	C	0	0	0	0	0	0	0	0	0	0	0	0
1.7727	0.4395	0.0000	O	0	0	0	0	0	0	0	0	0	0	0	0
2.8579	-0.4125	0.0000	C	0	0	0	0	0	0	0	0	0	0	0	0
-2.8579	-0.4125	0.0000	C	0	0	0	0	0	0	0	0	0	0	0	0
3.5724	-0.8250	0.0000	C	0	0	0	0	0	0	0	0	0	0	0	0
4.2868	-0.4125	0.0000	C	0	0	0	0	0	0	0	0	0	0	0	0
4.2868	0.4125	0.0000	C	0	0	0	0	0	0	0	0	0	0	0	0
3.5724	0.8250	0.0000	C	0	0	0	0	0	0	0	0	0	0	0	0
2.8579	0.4125	0.0000	C	0	0	0	0	0	0	0	0	0	0	0	0
-2.8579	0.4125	0.0000	C	0	0	0	0	0	0	0	0	0	0	0	0
-3.5724	0.8250	0.0000	C	0	0	0	0	0	0	0	0	0	0	0	0
-4.2868	0.4125	0.0000	C	0	0	0	0	0	0	0	0	0	0	0	0
-4.2868	-0.4125	0.0000	C	0	0	0	0	0	0	0	0	0	0	0	0
-3.5724	-0.8250	0.0000	C	0	0	0	0	0	0	0	0	0	0	0	0

1	2	1	0
2	3	1	0
3	4	1	0
4	5	1	0
1	6	1	0
5	7	1	0
1	8	1	1
5	9	1	1
1	10	1	6
5	11	1	6
7	12	1	0
6	13	1	0
12	14	2	0
14	15	1	0
15	16	2	0

16 17 1 0

17 18 2 0

18 12 1 0

13 19 2 0

19 20 1 0

20 21 2 0

21 22 1 0

22 23 2 0

23 13 1 0

M RAD 2 9 1 10 1

M END

Compound **12(*rac*+*meso*)**:

ChemDraw07102422082D

21 22 0 0 0 0 0 0 0 0999 V2000

-1.4289 -0.4125 0.0000 S 0 0 0 0 0 0 0 0 0 0 0 0

-0.7145 -0.8250 0.0000 C 0 0 0 0 0 0 0 0 0 0 0 0

0.0000 -0.4125 0.0000 C 0 0 0 0 0 0 0 0 0 0 0 0

0.7145 -0.8250 0.0000 C 0 0 0 0 0 0 0 0 0 0 0 0

1.4289 -0.4125 0.0000 S 0 0 0 0 0 0 0 0 0 0 0 0

-2.1434 -0.8250 0.0000 C 0 0 0 0 0 0 0 0 0 0 0 0

2.1434 -0.8250 0.0000 C 0 0 0 0 0 0 0 0 0 0 0 0

2.8579 -0.4125 0.0000 C 0 0 0 0 0 0 0 0 0 0 0 0

-2.8579 -0.4125 0.0000 C 0 0 0 0 0 0 0 0 0 0 0 0

3.5724 -0.8250 0.0000 C 0 0 0 0 0 0 0 0 0 0 0 0

4.2868 -0.4125 0.0000 C 0 0 0 0 0 0 0 0 0 0 0 0

4.2868 0.4125 0.0000 C 0 0 0 0 0 0 0 0 0 0 0 0

3.5724 0.8250 0.0000 C 0 0 0 0 0 0 0 0 0 0 0 0

2.8579 0.4125 0.0000 C 0 0 0 0 0 0 0 0 0 0 0 0

-2.8579 0.4125 0.0000 C 0 0 0 0 0 0 0 0 0 0 0 0

```
   -3.5724    0.8250    0.0000 C   0  0  0  0  0  0  0  0  0  0  0  0
   -4.2868    0.4125    0.0000 C   0  0  0  0  0  0  0  0  0  0  0  0
   -4.2868   -0.4125    0.0000 C   0  0  0  0  0  0  0  0  0  0  0  0
   -3.5724   -0.8250    0.0000 C   0  0  0  0  0  0  0  0  0  0  0  0
   -1.4289    0.4125    0.0000 O   0  0  0  0  0  0  0  0  0  0  0  0
    1.4289    0.4125    0.0000 O   0  0  0  0  0  0  0  0  0  0  0  0
  1  2  1  0
  2  3  1  0
  3  4  1  0
  4  5  1  0
  1  6  1  0
  5  7  1  0
  7  8  1  0
  6  9  1  0
  8 10  2  0
 10 11  1  0
 11 12  2  0
 12 13  1  0
 13 14  2  0
 14  8  1  0
  9 15  2  0
 15 16  1  0
 16 17  2  0
 17 18  1  0
 18 19  2  0
 19  9  1  0
  1 20  1  4
  5 21  1  4
M  SUB  2   1   4   5   4
M  END
```

Compound **13(*S*S,*S*S)**:

ChemDraw07102422092D

```
23 24  0  0  1  0  0  0  0  0999 V2000
   -1.4289   -0.4125    0.0000 S   0  0  0  0  0  0  0  0  0  0  0  0
   -0.7145   -0.8250    0.0000 C   0  0  0  0  0  0  0  0  0  0  0  0
    0.0000   -0.4125    0.0000 C   0  0  0  0  0  0  0  0  0  0  0  0
    0.7145   -0.8250    0.0000 C   0  0  0  0  0  0  0  0  0  0  0  0
    1.4289   -0.4125    0.0000 S   0  0  0  0  0  0  0  0  0  0  0  0
   -2.1434   -0.8250    0.0000 C   0  0  0  0  0  0  0  0  0  0  0  0
    2.1434   -0.8250    0.0000 C   0  0  0  0  0  0  0  0  0  0  0  0
   -1.8758    0.4125    0.0000 O   0  0  0  0  0  0  0  0  0  0  0  0
    0.9821    0.4125    0.0000 C   0  0  0  0  0  0  0  0  0  0  0  0
   -1.0508    0.4051    0.0000 C   0  0  0  0  0  0  0  0  0  0  0  0
    1.7727    0.4395    0.0000 O   0  0  0  0  0  0  0  0  0  0  0  0
    2.8579   -0.4125    0.0000 C   0  0  0  0  0  0  0  0  0  0  0  0
   -2.8579   -0.4125    0.0000 C   0  0  0  0  0  0  0  0  0  0  0  0
    3.5724   -0.8250    0.0000 N   0  0  0  0  0  0  0  0  0  0  0  0
    4.2868   -0.4125    0.0000 C   0  0  0  0  0  0  0  0  0  0  0  0
    4.2868    0.4125    0.0000 C   0  0  0  0  0  0  0  0  0  0  0  0
    3.5724    0.8250    0.0000 C   0  0  0  0  0  0  0  0  0  0  0  0
    2.8579    0.4125    0.0000 C   0  0  0  0  0  0  0  0  0  0  0  0
   -2.8579    0.4125    0.0000 C   0  0  0  0  0  0  0  0  0  0  0  0
   -3.5724    0.8250    0.0000 C   0  0  0  0  0  0  0  0  0  0  0  0
   -4.2868    0.4125    0.0000 C   0  0  0  0  0  0  0  0  0  0  0  0
   -4.2868   -0.4125    0.0000 C   0  0  0  0  0  0  0  0  0  0  0  0
   -3.5724   -0.8250    0.0000 N   0  0  0  0  0  0  0  0  0  0  0  0
  1  2  1  0
  2  3  1  0
  3  4  1  0
  4  5  1  0
```

1 6 1 0
5 7 1 0
1 8 1 1
5 9 1 1
1 10 1 6
5 11 1 6
7 12 1 0
6 13 1 0
12 14 2 0
14 15 1 0
15 16 2 0
16 17 1 0
17 18 2 0
18 12 1 0
13 19 2 0
19 20 1 0
20 21 2 0
21 22 1 0
22 23 2 0
23 13 1 0
M RAD 2 9 1 10 1
M END

Compound **13(*rac*+*meso*)**:

ChemDraw07102422102D

21 22 0 0 0 0 0 0 0 0999 V2000
-1.4289 -0.4125 0.0000 S 0 0 0 0 0 0 0 0 0 0 0 0
-0.7145 -0.8250 0.0000 C 0 0 0 0 0 0 0 0 0 0 0 0
0.0000 -0.4125 0.0000 C 0 0 0 0 0 0 0 0 0 0 0 0
0.7145 -0.8250 0.0000 C 0 0 0 0 0 0 0 0 0 0 0 0

1.4289	-0.4125	0.0000	S	0	0	0	0	0	0	0	0	0	0	0	0
-2.1434	-0.8250	0.0000	C	0	0	0	0	0	0	0	0	0	0	0	0
2.1434	-0.8250	0.0000	C	0	0	0	0	0	0	0	0	0	0	0	0
2.8579	-0.4125	0.0000	C	0	0	0	0	0	0	0	0	0	0	0	0
-2.8579	-0.4125	0.0000	C	0	0	0	0	0	0	0	0	0	0	0	0
3.5724	-0.8250	0.0000	N	0	0	0	0	0	0	0	0	0	0	0	0
4.2868	-0.4125	0.0000	C	0	0	0	0	0	0	0	0	0	0	0	0
4.2868	0.4125	0.0000	C	0	0	0	0	0	0	0	0	0	0	0	0
3.5724	0.8250	0.0000	C	0	0	0	0	0	0	0	0	0	0	0	0
2.8579	0.4125	0.0000	C	0	0	0	0	0	0	0	0	0	0	0	0
-2.8579	0.4125	0.0000	C	0	0	0	0	0	0	0	0	0	0	0	0
-3.5724	0.8250	0.0000	C	0	0	0	0	0	0	0	0	0	0	0	0
-4.2868	0.4125	0.0000	C	0	0	0	0	0	0	0	0	0	0	0	0
-4.2868	-0.4125	0.0000	C	0	0	0	0	0	0	0	0	0	0	0	0
-3.5724	-0.8250	0.0000	N	0	0	0	0	0	0	0	0	0	0	0	0
-1.4289	0.4125	0.0000	O	0	0	0	0	0	0	0	0	0	0	0	0
1.4289	0.4125	0.0000	O	0	0	0	0	0	0	0	0	0	0	0	0

1	2	1	0
2	3	1	0
3	4	1	0
4	5	1	0
1	6	1	0
5	7	1	0
7	8	1	0
6	9	1	0
8	10	2	0
10	11	1	0
11	12	2	0
12	13	1	0
13	14	2	0
14	8	1	0

```
 9 15  2  0
15 16  1  0
16 17  2  0
17 18  1  0
18 19  2  0
19  9  1  0
 1 20  1  4
 5 21  1  4
M  SUB  2   1   4   5   4
M  END
```

Compound **14(*R*S,*R*S)**:

```
  ChemDraw07102422112D

 11 10  0  0  1  0  0  0  0  0999 V2000
   -1.4977   -0.1854    0.0000 S   0  0  0  0  0  0  0  0  0  0  0  0
   -0.7832   -0.5979    0.0000 C   0  0  0  0  0  0  0  0  0  0  0  0
   -0.0687   -0.1854    0.0000 C   0  0  0  0  0  0  0  0  0  0  0  0
    0.6457   -0.5979    0.0000 C   0  0  0  0  0  0  0  0  0  0  0  0
    1.3602   -0.1854    0.0000 S   0  0  0  0  0  0  0  0  0  0  0  0
   -1.9446    0.6396    0.0000 O   0  0  0  0  0  0  0  0  0  0  0  0
    0.9133    0.6396    0.0000 C   0  0  0  0  0  0  0  0  0  0  0  0
   -1.1196    0.6322    0.0000 C   0  0  0  0  0  0  0  0  0  0  0  0
    1.7039    0.6666    0.0000 O   0  0  0  0  0  0  0  0  0  0  0  0
   -2.2122   -0.5979    0.0000 C   0  0  0  0  0  0  0  0  0  0  0  0
    2.2122   -0.6666    0.0000 C   0  0  0  0  0  0  0  0  0  0  0  0
  1 10  1  0
  5 11  1  0
  1  2  1  0
  2  3  1  0
  3  4  1  0
```

```
  4  5  1  0
  1  6  1  1
  5  7  1  1
  1  8  1  6
  5  9  1  6
M  RAD  2   7   1   8   1
M  STY  1   1 SUP
M  SLB  1   1   1
M  SAL   1  1  10
M  SBL   1  1   1
M  SMT   1 Me
M  SBV   1   1    0.7145    0.4125
M  STY  1   2 SUP
M  SLB  1   2   2
M  SAL   2  1  11
M  SBL   2  1   2
M  SMT   2 Me
M  SBV   2   2   -0.8520    0.4812
M  END
```

<u>Compound **14(*S*S,*S*S)**:</u>

```
  ChemDraw07102422112D

 11 10  0  0  1  0  0  0  0  0  0999 V2000
   -1.4289   -0.2062    0.0000 S   0  0  0  0  0  0  0  0  0  0  0  0
   -0.7145   -0.6187    0.0000 C   0  0  0  0  0  0  0  0  0  0  0  0
    0.0000   -0.2062    0.0000 C   0  0  0  0  0  0  0  0  0  0  0  0
    0.7145   -0.6187    0.0000 C   0  0  0  0  0  0  0  0  0  0  0  0
    1.4289   -0.2062    0.0000 S   0  0  0  0  0  0  0  0  0  0  0  0
   -1.8758    0.6187    0.0000 C   0  0  0  0  0  0  0  0  0  0  0  0
    0.9821    0.6187    0.0000 O   0  0  0  0  0  0  0  0  0  0  0  0
```

```
 -1.0508    0.6113    0.0000 O   0  0  0  0  0  0  0  0  0  0  0  0
  1.7727    0.5770    0.0000 C   0  0  0  0  0  0  0  0  0  0  0  0
 -2.1434   -0.6187    0.0000 C   0  0  0  0  0  0  0  0  0  0  0  0
  2.1434   -0.6187    0.0000 C   0  0  0  0  0  0  0  0  0  0  0  0
 1 10  1  0
 5 11  1  0
 1  2  1  0
 2  3  1  0
 3  4  1  0
 4  5  1  0
 1  6  1  1
 5  7  1  1
 1  8  1  6
 5  9  1  6
M  RAD  2   6   1   9   1
M  STY  1   1 SUP
M  SLB  1   1   1
M  SAL   1  1  10
M  SBL   1  1   1
M  SMT   1 Me
M  SBV   1   1    0.7145    0.4125
M  STY  1   2 SUP
M  SLB  1   2   2
M  SAL   2  1  11
M  SBL   2  1   2
M  SMT   2 Me
M  SBV   2   2   -0.7145    0.4125
M  END
```

Compound **14(*rac*+*meso*)**:

ChemDraw07102422122D

```
  9  8  0  0  0  0  0  0  0  0999 V2000
   -1.5149   -0.1891    0.0000 S   0  0  0  0  0  0  0  0  0  0  0  0
   -0.8004   -0.6016    0.0000 C   0  0  0  0  0  0  0  0  0  0  0  0
   -0.0859   -0.1891    0.0000 C   0  0  0  0  0  0  0  0  0  0  0  0
    0.6285   -0.6016    0.0000 C   0  0  0  0  0  0  0  0  0  0  0  0
    1.3430   -0.1891    0.0000 S   0  0  0  0  0  0  0  0  0  0  0  0
   -1.5149    0.6359    0.0000 O   0  0  0  0  0  0  0  0  0  0  0  0
    1.3430    0.6359    0.0000 O   0  0  0  0  0  0  0  0  0  0  0  0
   -2.2294   -0.6016    0.0000 C   0  0  0  0  0  0  0  0  0  0  0  0
    2.2294   -0.6359    0.0000 C   0  0  0  0  0  0  0  0  0  0  0  0
  1  8  1  0
  5  9  1  0
  1  2  1  0
  2  3  1  0
  3  4  1  0
  4  5  1  0
  1  6  1  4
  5  7  1  4
M  SUB  2   1   4   5   4
M  STY  1   1 SUP
M  SLB  1   1   1
M  SAL   1  1   8
M  SBL   1  1   1
M  SMT   1 Me
M  SBV   1   1    0.7145    0.4125
M  STY  1   2 SUP
M  SLB  1   2   2
M  SAL   2  1   9
M  SBL   2  1   2
M  SMT   2 Me
```

M SBV   2   2   -0.8863   0.4469
M END

Compound **15(*R*S,*R*S)**:

ChemDraw07102422132D

```
 13 12  0  0  1  0  0  0  0  0  0999 V2000
   -1.4957   -0.1854    0.0000 S   0  0  0  0  0  0  0  0  0  0  0  0
   -0.7813   -0.5979    0.0000 C   0  0  0  0  0  0  0  0  0  0  0  0
   -0.0668   -0.1854    0.0000 C   0  0  0  0  0  0  0  0  0  0  0  0
    0.6477   -0.5979    0.0000 C   0  0  0  0  0  0  0  0  0  0  0  0
    1.3622   -0.1854    0.0000 S   0  0  0  0  0  0  0  0  0  0  0  0
   -1.9426    0.6396    0.0000 O   0  0  0  0  0  0  0  0  0  0  0  0
    0.9153    0.6396    0.0000 C   0  0  0  0  0  0  0  0  0  0  0  0
   -1.1176    0.6322    0.0000 C   0  0  0  0  0  0  0  0  0  0  0  0
    1.7059    0.6666    0.0000 O   0  0  0  0  0  0  0  0  0  0  0  0
   -2.2102   -0.5979    0.0000 C   0  0  0  0  0  0  0  0  0  0  0  0
    2.2141   -0.6666    0.0000 C   0  0  0  0  0  0  0  0  0  0  0  0
    2.9247   -0.2474    0.0000 C   0  0  0  0  0  0  0  0  0  0  0  0
   -2.9247   -0.1854    0.0000 C   0  0  0  0  0  0  0  0  0  0  0  0
  1  2  1  0
  2  3  1  0
  3  4  1  0
  4  5  1  0
  1 10  1  0
  5 11  1  0
  1  6  1  1
  5  7  1  1
  1  8  1  6
  5  9  1  6
 11 12  1  0
```

```
 10 13  1  0
M  RAD  2   7   1   8   1
M  END
```

Compound **15(*S*S,*S*S)**:

```
 ChemDraw07102422132D

 13 12  0  0  1  0  0  0  0  0  0999 V2000
   -1.4289   -0.2062    0.0000 S   0  0  0  0  0  0  0  0  0  0  0  0
   -0.7145   -0.6187    0.0000 C   0  0  0  0  0  0  0  0  0  0  0  0
    0.0000   -0.2062    0.0000 C   0  0  0  0  0  0  0  0  0  0  0  0
    0.7145   -0.6187    0.0000 C   0  0  0  0  0  0  0  0  0  0  0  0
    1.4289   -0.2062    0.0000 S   0  0  0  0  0  0  0  0  0  0  0  0
   -2.1434   -0.6187    0.0000 C   0  0  0  0  0  0  0  0  0  0  0  0
    2.1434   -0.6187    0.0000 C   0  0  0  0  0  0  0  0  0  0  0  0
   -1.8758    0.6187    0.0000 C   0  0  0  0  0  0  0  0  0  0  0  0
    0.9821    0.6187    0.0000 O   0  0  0  0  0  0  0  0  0  0  0  0
   -1.0508    0.6113    0.0000 O   0  0  0  0  0  0  0  0  0  0  0  0
    1.7727    0.5770    0.0000 C   0  0  0  0  0  0  0  0  0  0  0  0
    2.8579   -0.2062    0.0000 C   0  0  0  0  0  0  0  0  0  0  0  0
   -2.8579   -0.2062    0.0000 C   0  0  0  0  0  0  0  0  0  0  0  0
  1  2  1  0
  2  3  1  0
  3  4  1  0
  4  5  1  0
  1  6  1  0
  5  7  1  0
  1  8  1  1
  5  9  1  1
  1 10  1  6
  5 11  1  6
```

```
 7 12  1  0
 6 13  1  0
M  RAD  2   8   1  11   1
M  END
```

Compound **15(*rac*+*meso*)**:

```
  ChemDraw07102422142D

 11 10  0  0  0  0  0  0  0  0999 V2000
   -1.5026   -0.1891    0.0000 S   0  0  0  0  0  0  0  0  0  0  0  0
   -0.7882   -0.6016    0.0000 C   0  0  0  0  0  0  0  0  0  0  0  0
   -0.0737   -0.1891    0.0000 C   0  0  0  0  0  0  0  0  0  0  0  0
    0.6408   -0.6016    0.0000 C   0  0  0  0  0  0  0  0  0  0  0  0
    1.3552   -0.1891    0.0000 S   0  0  0  0  0  0  0  0  0  0  0  0
   -2.2171   -0.6016    0.0000 C   0  0  0  0  0  0  0  0  0  0  0  0
    2.2416   -0.6359    0.0000 C   0  0  0  0  0  0  0  0  0  0  0  0
   -1.5026    0.6359    0.0000 O   0  0  0  0  0  0  0  0  0  0  0  0
    1.3552    0.6359    0.0000 O   0  0  0  0  0  0  0  0  0  0  0  0
    2.9316   -0.1837    0.0000 C   0  0  0  0  0  0  0  0  0  0  0  0
   -2.9316   -0.1891    0.0000 C   0  0  0  0  0  0  0  0  0  0  0  0
  1  2  1  0
  2  3  1  0
  3  4  1  0
  4  5  1  0
  1  6  1  0
  5  7  1  0
  1  8  1  4
  5  9  1  4
  7 10  1  0
  6 11  1  0
M  SUB  2   1   4   5   4
```

```
M  END
```

Compound **16(*R*$_S$,*R*$_S$)**:

```
  ChemDraw07102422152D

 15 14  0  0  1  0  0  0  0  0999 V2000
   -1.4977   -0.1854    0.0000 S   0  0  0  0  0  0  0  0  0  0  0  0
   -0.7832   -0.5979    0.0000 C   0  0  0  0  0  0  0  0  0  0  0  0
   -0.0687   -0.1854    0.0000 C   0  0  0  0  0  0  0  0  0  0  0  0
    0.6458   -0.5979    0.0000 C   0  0  0  0  0  0  0  0  0  0  0  0
    1.3602   -0.1854    0.0000 S   0  0  0  0  0  0  0  0  0  0  0  0
   -1.9445    0.6396    0.0000 O   0  0  0  0  0  0  0  0  0  0  0  0
    0.9133    0.6396    0.0000 C   0  0  0  0  0  0  0  0  0  0  0  0
   -1.1195    0.6322    0.0000 C   0  0  0  0  0  0  0  0  0  0  0  0
    1.7040    0.6666    0.0000 O   0  0  0  0  0  0  0  0  0  0  0  0
   -2.2121   -0.5979    0.0000 C   0  0  0  0  0  0  0  0  0  0  0  0
    2.2122   -0.6666    0.0000 C   0  0  0  0  0  0  0  0  0  0  0  0
    2.9228   -0.2474    0.0000 C   0  0  0  0  0  0  0  0  0  0  0  0
   -2.9266   -0.1854    0.0000 C   0  0  0  0  0  0  0  0  0  0  0  0
    3.6411   -0.6532    0.0000 C   0  0  0  0  0  0  0  0  0  0  0  0
   -3.6411   -0.5979    0.0000 C   0  0  0  0  0  0  0  0  0  0  0  0
  1  2  1  0
  2  3  1  0
  3  4  1  0
  4  5  1  0
  1 10  1  0
  5 11  1  0
  1  6  1  1
  5  7  1  1
  1  8  1  6
  5  9  1  6
```

```
 11 12  1  0
 10 13  1  0
 12 14  1  0
 13 15  1  0
M  RAD  2   7   1   8   1
M  END
```


Compound **16(*rac*+*meso*)**:


```
  ChemDraw07102422172D

 13 12  0  0  0  0  0  0  0  0999 V2000
   -1.5137   -0.1891    0.0000 S   0  0  0  0  0  0  0  0  0  0  0  0
   -0.7993   -0.6016    0.0000 C   0  0  0  0  0  0  0  0  0  0  0  0
   -0.0848   -0.1891    0.0000 C   0  0  0  0  0  0  0  0  0  0  0  0
    0.6297   -0.6016    0.0000 C   0  0  0  0  0  0  0  0  0  0  0  0
    1.3442   -0.1891    0.0000 S   0  0  0  0  0  0  0  0  0  0  0  0
   -2.2282   -0.6016    0.0000 C   0  0  0  0  0  0  0  0  0  0  0  0
    2.2305   -0.6359    0.0000 C   0  0  0  0  0  0  0  0  0  0  0  0
   -1.5137    0.6359    0.0000 O   0  0  0  0  0  0  0  0  0  0  0  0
    1.3442    0.6359    0.0000 O   0  0  0  0  0  0  0  0  0  0  0  0
    2.9205   -0.1837    0.0000 C   0  0  0  0  0  0  0  0  0  0  0  0
   -2.9427   -0.1891    0.0000 C   0  0  0  0  0  0  0  0  0  0  0  0
    3.6571   -0.5551    0.0000 C   0  0  0  0  0  0  0  0  0  0  0  0
   -3.6571   -0.6016    0.0000 C   0  0  0  0  0  0  0  0  0  0  0  0
  1  2  1  0
  2  3  1  0
  3  4  1  0
  4  5  1  0
  1  6  1  0
  5  7  1  0
  1  8  1  4
```

5 9 1 4
7 10 1 0
6 11 1 0
10 12 1 0
11 13 1 0
M SUB 2 1 4 5 4
M END

Compound **17(*S*S,*S*S)**:

ChemDraw07102422162D

```
15 14  0  0  1  0  0  0  0  0999 V2000
  -1.4957    0.2271    0.0000 S   0  0  0  0  0  0  0  0  0  0  0  0
  -0.7813   -0.1854    0.0000 C   0  0  0  0  0  0  0  0  0  0  0  0
  -0.0668    0.2271    0.0000 C   0  0  0  0  0  0  0  0  0  0  0  0
   0.6477   -0.1854    0.0000 C   0  0  0  0  0  0  0  0  0  0  0  0
   1.3622    0.2271    0.0000 S   0  0  0  0  0  0  0  0  0  0  0  0
  -1.9426    1.0521    0.0000 O   0  0  0  0  0  0  0  0  0  0  0  0
   0.9153    1.0521    0.0000 C   0  0  0  0  0  0  0  0  0  0  0  0
  -1.1176    1.0447    0.0000 C   0  0  0  0  0  0  0  0  0  0  0  0
   1.7059    1.0791    0.0000 O   0  0  0  0  0  0  0  0  0  0  0  0
  -2.2102   -0.1854    0.0000 C   0  0  0  0  0  0  0  0  0  0  0  0
   2.2141   -0.2541    0.0000 C   0  0  0  0  0  0  0  0  0  0  0  0
   2.9247    0.1651    0.0000 C   0  0  0  0  0  0  0  0  0  0  0  0
  -2.9247    0.2271    0.0000 C   0  0  0  0  0  0  0  0  0  0  0  0
  -2.2102   -1.0104    0.0000 C   0  0  0  0  0  0  0  0  0  0  0  0
   2.2219   -1.0791    0.0000 C   0  0  0  0  0  0  0  0  0  0  0  0
  1  2  1  0
  2  3  1  0
  3  4  1  0
  4  5  1  0
```

```
  1 10  1  0
  5 11  1  0
  1  6  1  1
  5  7  1  1
  1  8  1  6
  5  9  1  6
 11 12  1  0
 10 13  1  0
 10 14  1  0
 11 15  1  0
M  RAD  2   7   1   8   1
M  END
```

<u>Compound **17(*rac*+*meso*)**:</u>

```
  ChemDraw07102422172D

 13 12  0  0  0  0  0  0  0  0999 V2000
   -1.5026    0.2228    0.0000 S   0  0  0  0  0  0  0  0  0  0  0  0
   -0.7882   -0.1897    0.0000 C   0  0  0  0  0  0  0  0  0  0  0  0
   -0.0737    0.2228    0.0000 C   0  0  0  0  0  0  0  0  0  0  0  0
    0.6408   -0.1897    0.0000 C   0  0  0  0  0  0  0  0  0  0  0  0
    1.3552    0.2228    0.0000 S   0  0  0  0  0  0  0  0  0  0  0  0
   -2.2171   -0.1897    0.0000 C   0  0  0  0  0  0  0  0  0  0  0  0
    2.2416   -0.2241    0.0000 C   0  0  0  0  0  0  0  0  0  0  0  0
   -1.5026    1.0478    0.0000 O   0  0  0  0  0  0  0  0  0  0  0  0
    1.3552    1.0478    0.0000 O   0  0  0  0  0  0  0  0  0  0  0  0
    2.9316    0.2282    0.0000 C   0  0  0  0  0  0  0  0  0  0  0  0
   -2.9316    0.2228    0.0000 C   0  0  0  0  0  0  0  0  0  0  0  0
   -2.2171   -1.0147    0.0000 C   0  0  0  0  0  0  0  0  0  0  0  0
    2.2883   -1.0478    0.0000 C   0  0  0  0  0  0  0  0  0  0  0  0
  1  2  1  0
```

2 3 1 0

3 4 1 0

4 5 1 0

1 6 1 0

5 7 1 0

1 8 1 4

5 9 1 4

7 10 1 0

6 11 1 0

6 12 1 0

7 13 1 0

M SUB 2 1 4 5 4

M END

<u>Compound **18(*S*S,*S*S)**:</u>

ChemDraw07102422182D

17 16 0 0 1 0 0 0 0 0 0999 V2000

-1.4977 0.2271 0.0000 S 0 0 0 0 0 0 0 0 0 0 0 0

-0.7832 -0.1854 0.0000 C 0 0 0 0 0 0 0 0 0 0 0 0

-0.0687 0.2271 0.0000 C 0 0 0 0 0 0 0 0 0 0 0 0

0.6457 -0.1854 0.0000 C 0 0 0 0 0 0 0 0 0 0 0 0

1.3602 0.2271 0.0000 S 0 0 0 0 0 0 0 0 0 0 0 0

-1.9446 1.0521 0.0000 O 0 0 0 0 0 0 0 0 0 0 0 0

0.9133 1.0521 0.0000 C 0 0 0 0 0 0 0 0 0 0 0 0

-1.1196 1.0447 0.0000 C 0 0 0 0 0 0 0 0 0 0 0 0

1.7039 1.0791 0.0000 O 0 0 0 0 0 0 0 0 0 0 0 0

-2.2122 -0.1854 0.0000 C 0 0 0 0 0 0 0 0 0 0 0 0

2.2122 -0.2541 0.0000 C 0 0 0 0 0 0 0 0 0 0 0 0

3.0091 -0.0406 0.0000 C 0 0 0 0 0 0 0 0 0 0 0 0

-3.0091 0.0282 0.0000 C 0 0 0 0 0 0 0 0 0 0 0 0

```
  -2.2122   -1.0104    0.0000 C   0  0  0  0  0  0  0  0  0  0  0  0
   2.2122   -1.0791    0.0000 C   0  0  0  0  0  0  0  0  0  0  0  0
   2.4257    0.5428    0.0000 C   0  0  0  0  0  0  0  0  0  0  0  0
  -2.4257    0.6115    0.0000 C   0  0  0  0  0  0  0  0  0  0  0  0
  1  2  1  0
  2  3  1  0
  3  4  1  0
  4  5  1  0
  1 10  1  0
  5 11  1  0
  1  6  1  1
  5  7  1  1
  1  8  1  6
  5  9  1  6
 11 12  1  0
 10 13  1  0
 10 14  1  0
 11 15  1  0
 11 16  1  0
 10 17  1  0
M  RAD  2   7   1   8   1
M  END
```

<u>Compound **18(*rac+meso*)**:</u>

```
  ChemDraw07102422192D

 15 14  0  0  0  0  0  0  0  0999 V2000
   -1.5149    0.2234    0.0000 S   0  0  0  0  0  0  0  0  0  0  0  0
   -0.8004   -0.1891    0.0000 C   0  0  0  0  0  0  0  0  0  0  0  0
   -0.0859    0.2234    0.0000 C   0  0  0  0  0  0  0  0  0  0  0  0
    0.6285   -0.1891    0.0000 C   0  0  0  0  0  0  0  0  0  0  0  0
```

```
  1.3430    0.2234    0.0000 S   0  0  0  0  0  0  0  0  0  0  0  0
 -2.2294   -0.1891    0.0000 C   0  0  0  0  0  0  0  0  0  0  0  0
  2.2294   -0.2234    0.0000 C   0  0  0  0  0  0  0  0  0  0  0  0
 -1.5149    1.0484    0.0000 O   0  0  0  0  0  0  0  0  0  0  0  0
  1.3430    1.0484    0.0000 O   0  0  0  0  0  0  0  0  0  0  0  0
  3.0262   -0.0099    0.0000 C   0  0  0  0  0  0  0  0  0  0  0  0
 -3.0262    0.0245    0.0000 C   0  0  0  0  0  0  0  0  0  0  0  0
 -2.2294   -1.0141    0.0000 C   0  0  0  0  0  0  0  0  0  0  0  0
  2.2294   -1.0484    0.0000 C   0  0  0  0  0  0  0  0  0  0  0  0
  2.4429    0.5735    0.0000 C   0  0  0  0  0  0  0  0  0  0  0  0
 -2.4429    0.6078    0.0000 C   0  0  0  0  0  0  0  0  0  0  0  0
 1  2  1  0
 2  3  1  0
 3  4  1  0
 4  5  1  0
 1  6  1  0
 5  7  1  0
 1  8  1  4
 5  9  1  4
 7 10  1  0
 6 11  1  0
 6 12  1  0
 7 13  1  0
 7 14  1  0
 6 15  1  0
M  SUB  2   1   4   5   4
M  END
```

<u>Compound **25(*R*S)**:</u>

```
ChemDraw07102422202D
```

```
 12 12  0  0  1  0  0  0  0  0999 V2000
    0.0000   -0.0135    0.0000 C   0  0  0  0  0  0  0  0  0  0  0  0
    0.7145    0.3990    0.0000 S   0  0  0  0  0  0  0  0  0  0  0  0
    0.3020    1.1135    0.0000 C   0  0  0  0  0  0  0  0  0  0  0  0
    1.0582    1.2510    0.0000 O   0  0  0  0  0  0  0  0  0  0  0  0
    1.4289   -0.0135    0.0000 C   0  0  0  0  0  0  0  0  0  0  0  0
    2.1434    0.3990    0.0000 C   0  0  0  0  0  0  0  0  0  0  0  0
   -0.7145    0.3990    0.0000 C   0  0  0  0  0  0  0  0  0  0  0  0
   -1.4289   -0.0135    0.0000 C   0  0  0  0  0  0  0  0  0  0  0  0
   -1.4289   -0.8385    0.0000 C   0  0  0  0  0  0  0  0  0  0  0  0
   -0.7145   -1.2510    0.0000 C   0  0  0  0  0  0  0  0  0  0  0  0
    0.0000   -0.8385    0.0000 C   0  0  0  0  0  0  0  0  0  0  0  0
   -2.1434   -1.2510    0.0000 C   0  0  0  0  0  0  0  0  0  0  0  0
  1  2  1  0
  2  3  1  1
  2  4  1  6
  2  5  1  0
  5  6  2  0
  1  7  2  0
  7  8  1  0
  8  9  2  0
  9 10  1  0
 10 11  2  0
 11  1  1  0
  9 12  1  0
M  RAD  1   3   1
M  END
```

<u>Compound **25(*S*S)**:</u>

```
  ChemDraw07102422202D
```

```
 12 12  0  0  1  0  0  0  0  0999 V2000
    0.0000   -0.0135    0.0000 C   0  0  0  0  0  0  0  0  0  0  0  0
    0.7145    0.3990    0.0000 S   0  0  0  0  0  0  0  0  0  0  0  0
    0.3020    1.1135    0.0000 O   0  0  0  0  0  0  0  0  0  0  0  0
    1.0582    1.2510    0.0000 C   0  0  0  0  0  0  0  0  0  0  0  0
    1.4289   -0.0135    0.0000 C   0  0  0  0  0  0  0  0  0  0  0  0
    2.1434    0.3990    0.0000 C   0  0  0  0  0  0  0  0  0  0  0  0
   -0.7145    0.3990    0.0000 C   0  0  0  0  0  0  0  0  0  0  0  0
   -1.4289   -0.0135    0.0000 C   0  0  0  0  0  0  0  0  0  0  0  0
   -1.4289   -0.8385    0.0000 C   0  0  0  0  0  0  0  0  0  0  0  0
   -0.7145   -1.2510    0.0000 C   0  0  0  0  0  0  0  0  0  0  0  0
    0.0000   -0.8385    0.0000 C   0  0  0  0  0  0  0  0  0  0  0  0
   -2.1434   -1.2510    0.0000 C   0  0  0  0  0  0  0  0  0  0  0  0
  1  2  1  0
  2  3  1  1
  2  4  1  6
  2  5  1  0
  5  6  2  0
  1  7  2  0
  7  8  1  0
  8  9  2  0
  9 10  1  0
 10 11  2  0
 11  1  1  0
  9 12  1  0
M  RAD  1   4   1
M  END
```

<u>Compound **25(*rac*)**:</u>

```
  ChemDraw07102422212D
```

```
 11 11  0  0  0  0  0  0  0  0999 V2000
    0.0000    0.0000    0.0000 C   0  0  0  0  0  0  0  0  0  0  0  0
    0.7145    0.4125    0.0000 S   0  0  0  0  0  0  0  0  0  0  0  0
    1.4289    0.0000    0.0000 C   0  0  0  0  0  0  0  0  0  0  0  0
    2.1434    0.4125    0.0000 C   0  0  0  0  0  0  0  0  0  0  0  0
    0.7145    1.2375    0.0000 O   0  0  0  0  0  0  0  0  0  0  0  0
   -0.7145    0.4125    0.0000 C   0  0  0  0  0  0  0  0  0  0  0  0
   -1.4289    0.0000    0.0000 C   0  0  0  0  0  0  0  0  0  0  0  0
   -1.4289   -0.8250    0.0000 C   0  0  0  0  0  0  0  0  0  0  0  0
   -0.7145   -1.2375    0.0000 C   0  0  0  0  0  0  0  0  0  0  0  0
    0.0000   -0.8250    0.0000 C   0  0  0  0  0  0  0  0  0  0  0  0
   -2.1434   -1.2375    0.0000 C   0  0  0  0  0  0  0  0  0  0  0  0
  1  2  1  0
  2  3  1  0
  3  4  2  0
  2  5  1  4
  1  6  2  0
  6  7  1  0
  7  8  2  0
  8  9  1  0
  9 10  2  0
 10  1  1  0
  8 11  1  0
M  SUB  1   2   4
M  END
```

Compound **26(*R*S)**:

```
  ChemDraw07102422222D

  9  8  0  0  1  0  0  0  0  0999 V2000
   -0.7145   -0.2338    0.0000 C   0  0  0  0  0  0  0  0  0  0  0  0
```

```
  0.0000    0.1787    0.0000 S   0  0  0  0  0  0  0  0  0  0  0  0
 -0.4125    0.8932    0.0000 C   0  0  0  0  0  0  0  0  0  0  0  0
  0.3437    1.0307    0.0000 O   0  0  0  0  0  0  0  0  0  0  0  0
  0.7145   -0.2338    0.0000 C   0  0  0  0  0  0  0  0  0  0  0  0
  1.4289    0.1787    0.0000 C   0  0  0  0  0  0  0  0  0  0  0  0
 -1.4289    0.1787    0.0000 C   0  0  0  0  0  0  0  0  0  0  0  0
 -0.5009   -1.0307    0.0000 C   0  0  0  0  0  0  0  0  0  0  0  0
 -1.4289   -0.6463    0.0000 C   0  0  0  0  0  0  0  0  0  0  0  0
  1  2  1  0
  2  3  1  1
  2  4  1  6
  2  5  1  0
  5  6  2  0
  1  7  1  0
  1  8  1  0
  1  9  1  0
M  RAD  1   3   1
M  END
```

<u>Compound **26(*S*S)**:</u>

```
  ChemDraw07102422232D

  9  8  0  0  1  0  0  0  0  0999 V2000
 -0.7145   -0.2338    0.0000 C   0  0  0  0  0  0  0  0  0  0  0  0
  0.0000    0.1787    0.0000 S   0  0  0  0  0  0  0  0  0  0  0  0
 -0.4125    0.8932    0.0000 O   0  0  0  0  0  0  0  0  0  0  0  0
  0.3437    1.0307    0.0000 C   0  0  0  0  0  0  0  0  0  0  0  0
  0.7145   -0.2338    0.0000 C   0  0  0  0  0  0  0  0  0  0  0  0
  1.4289    0.1787    0.0000 C   0  0  0  0  0  0  0  0  0  0  0  0
 -1.4289    0.1787    0.0000 C   0  0  0  0  0  0  0  0  0  0  0  0
 -0.5009   -1.0307    0.0000 C   0  0  0  0  0  0  0  0  0  0  0  0
```

-1.4289   -0.6463   0.0000 C   0 0 0 0 0 0 0 0 0 0 0 0

1 2 1 0

2 3 1 1

2 4 1 6

2 5 1 0

5 6 2 0

1 7 1 0

1 8 1 0

1 9 1 0

M RAD 1   4   1

M END

Compound **26(*rac*)**:

ChemDraw07102422232D

8 7 0 0 0 0 0 0 0 0999 V2000

-0.7145   -0.2203   0.0000 C   0 0 0 0 0 0 0 0 0 0 0 0

0.0000   0.1922   0.0000 S   0 0 0 0 0 0 0 0 0 0 0 0

0.7145   -0.2203   0.0000 C   0 0 0 0 0 0 0 0 0 0 0 0

1.4289   0.1922   0.0000 C   0 0 0 0 0 0 0 0 0 0 0 0

0.0000   1.0172   0.0000 O   0 0 0 0 0 0 0 0 0 0 0 0

-1.4289   0.1922   0.0000 C   0 0 0 0 0 0 0 0 0 0 0 0

-0.5009   -1.0172   0.0000 C   0 0 0 0 0 0 0 0 0 0 0 0

-1.4289   -0.6328   0.0000 C   0 0 0 0 0 0 0 0 0 0 0 0

1 2 1 0

2 3 1 0

3 4 2 0

2 5 1 4

1 6 1 0

1 7 1 0

1 8 1 0

```
M  SUB  1   2   4
M  END
```

<u>Compound **27(*S*S)**:</u>

```
  ChemDraw07102422242D

  6  5  0  0  1  0  0  0  0  0999 V2000
   -0.3572   -0.2197    0.0000 S   0  0  0  0  0  0  0  0  0  0  0  0
   -0.7697    0.4947    0.0000 C   0  0  0  0  0  0  0  0  0  0  0  0
   -0.0135    0.6322    0.0000 O   0  0  0  0  0  0  0  0  0  0  0  0
    0.3572   -0.6322    0.0000 C   0  0  0  0  0  0  0  0  0  0  0  0
    1.0717   -0.2197    0.0000 C   0  0  0  0  0  0  0  0  0  0  0  0
   -1.0717   -0.6322    0.0000 C   0  0  0  0  0  0  0  0  0  0  0  0
  1  6  1  0
  1  2  1  1
  1  3  1  6
  1  4  1  0
  4  5  2  0
M  RAD  1   2   1
M  STY  1   1 SUP
M  SLB  1   1   1
M  SAL   1  1   6
M  SBL   1  1   1
M  SMT   1 Me
M  SBV   1   1    0.7145    0.4125
M  END
```

<u>Compound **27(*R*S)**:</u>

```
  ChemDraw07102422252D

  6  5  0  0  1  0  0  0  0  0999 V2000
```

```
 -0.3572   -0.2197    0.0000 S   0  0  0  0  0  0  0  0  0  0  0  0
 -0.7697    0.4947    0.0000 O   0  0  0  0  0  0  0  0  0  0  0  0
 -0.0135    0.6322    0.0000 C   0  0  0  0  0  0  0  0  0  0  0  0
  0.3572   -0.6322    0.0000 C   0  0  0  0  0  0  0  0  0  0  0  0
  1.0717   -0.2197    0.0000 C   0  0  0  0  0  0  0  0  0  0  0  0
 -1.0717   -0.6322    0.0000 C   0  0  0  0  0  0  0  0  0  0  0  0
 1  6  1  0
 1  2  1  1
 1  3  1  6
 1  4  1  0
 4  5  2  0
M  RAD  1   3   1
M  STY  1   1 SUP
M  SLB  1   1   1
M  SAL   1  1   6
M  SBL   1  1   1
M  SMT   1 Me
M  SBV   1   1    0.7145    0.4125
M  END
```

Compound **27(*rac*)**:

```
 ChemDraw07102422252D

 5  4  0  0  0  0  0  0  0  0999 V2000
 -0.3572   -0.2062    0.0000 S   0  0  0  0  0  0  0  0  0  0  0  0
  0.3572   -0.6187    0.0000 C   0  0  0  0  0  0  0  0  0  0  0  0
  1.0717   -0.2062    0.0000 C   0  0  0  0  0  0  0  0  0  0  0  0
 -0.3572    0.6187    0.0000 O   0  0  0  0  0  0  0  0  0  0  0  0
 -1.0717   -0.6187    0.0000 C   0  0  0  0  0  0  0  0  0  0  0  0
 1  5  1  0
 1  2  1  0
```

```
  2  3  2  0
  1  4  1  4
M  SUB  1   1   4
M  STY  1   1 SUP
M  SLB  1   1   1
M  SAL   1  1   5
M  SBL   1  1   1
M  SMT   1 Me
M  SBV   1   1    0.7145    0.4125
M  END
```

Compound **28(*R*S,*R*S)**:

```
  ChemDraw07102422262D

 25 26  0  0  1  0  0  0  0  0999 V2000
   -2.8579   -0.0135    0.0000 C   0  0  0  0  0  0  0  0  0  0  0  0
   -2.1434    0.3990    0.0000 S   0  0  0  0  0  0  0  0  0  0  0  0
   -2.5559    1.1135    0.0000 C   0  0  0  0  0  0  0  0  0  0  0  0
   -1.7997    1.2510    0.0000 O   0  0  0  0  0  0  0  0  0  0  0  0
   -1.4289   -0.0135    0.0000 C   0  0  0  0  0  0  0  0  0  0  0  0
   -0.7145    0.3990    0.0000 C   0  0  0  0  0  0  0  0  0  0  0  0
    0.0000   -0.0135    0.0000 S   0  0  0  0  0  0  0  0  0  0  0  0
    0.7145    0.3990    0.0000 C   0  0  0  0  0  0  0  0  0  0  0  0
    1.4289   -0.0135    0.0000 C   0  0  0  0  0  0  0  0  0  0  0  0
    2.1434    0.3990    0.0000 S   0  0  0  0  0  0  0  0  0  0  0  0
    2.8579   -0.0135    0.0000 C   0  0  0  0  0  0  0  0  0  0  0  0
    1.7309    1.1135    0.0000 O   0  0  0  0  0  0  0  0  0  0  0  0
    2.5559    1.1135    0.0000 C   0  0  0  0  0  0  0  0  0  0  0  0
    2.8579   -0.8385    0.0000 C   0  0  0  0  0  0  0  0  0  0  0  0
    3.5724   -1.2510    0.0000 C   0  0  0  0  0  0  0  0  0  0  0  0
    4.2868   -0.8385    0.0000 C   0  0  0  0  0  0  0  0  0  0  0  0
```

4.2868 -0.0135 0.0000 C 0 0 0 0 0 0 0 0 0 0 0 0
3.5724 0.3990 0.0000 C 0 0 0 0 0 0 0 0 0 0 0 0
-3.5724 0.3990 0.0000 C 0 0 0 0 0 0 0 0 0 0 0 0
-4.2868 -0.0135 0.0000 C 0 0 0 0 0 0 0 0 0 0 0 0
-4.2868 -0.8385 0.0000 C 0 0 0 0 0 0 0 0 0 0 0 0
-3.5724 -1.2510 0.0000 C 0 0 0 0 0 0 0 0 0 0 0 0
-2.8579 -0.8385 0.0000 C 0 0 0 0 0 0 0 0 0 0 0 0
5.0013 -1.2510 0.0000 C 0 0 0 0 0 0 0 0 0 0 0 0
-5.0013 -1.2510 0.0000 C 0 0 0 0 0 0 0 0 0 0 0 0
1 2 1 0
2 3 1 1
2 4 1 6
2 5 1 0
5 6 1 0
6 7 1 0
7 8 1 0
8 9 1 0
9 10 1 0
10 11 1 0
10 12 1 1
10 13 1 6
11 14 2 0
14 15 1 0
15 16 2 0
16 17 1 0
17 18 2 0
18 11 1 0
1 19 2 0
19 20 1 0
20 21 2 0
21 22 1 0

22 23 2 0

23 1 1 0

16 24 1 0

21 25 1 0

M RAD 2 3 1 13 1

M END

Compound **28(*S*$_S$,*S*$_S$)**:

ChemDraw07102422272D

25 26 0 0 1 0 0 0 0 0999 V2000

-2.8579 -0.0135 0.0000 C 0 0 0 0 0 0 0 0 0 0 0 0

-2.1434 0.3990 0.0000 S 0 0 0 0 0 0 0 0 0 0 0 0

-2.5559 1.1135 0.0000 O 0 0 0 0 0 0 0 0 0 0 0 0

-1.7997 1.2510 0.0000 C 0 0 0 0 0 0 0 0 0 0 0 0

-1.4289 -0.0135 0.0000 C 0 0 0 0 0 0 0 0 0 0 0 0

-0.7145 0.3990 0.0000 C 0 0 0 0 0 0 0 0 0 0 0 0

0.0000 -0.0135 0.0000 S 0 0 0 0 0 0 0 0 0 0 0 0

0.7145 0.3990 0.0000 C 0 0 0 0 0 0 0 0 0 0 0 0

1.4289 -0.0135 0.0000 C 0 0 0 0 0 0 0 0 0 0 0 0

2.1434 0.3990 0.0000 S 0 0 0 0 0 0 0 0 0 0 0 0

2.8579 -0.0135 0.0000 C 0 0 0 0 0 0 0 0 0 0 0 0

1.7309 1.1135 0.0000 C 0 0 0 0 0 0 0 0 0 0 0 0

2.5559 1.1135 0.0000 O 0 0 0 0 0 0 0 0 0 0 0 0

2.8579 -0.8385 0.0000 C 0 0 0 0 0 0 0 0 0 0 0 0

3.5724 -1.2510 0.0000 C 0 0 0 0 0 0 0 0 0 0 0 0

4.2868 -0.8385 0.0000 C 0 0 0 0 0 0 0 0 0 0 0 0

4.2868 -0.0135 0.0000 C 0 0 0 0 0 0 0 0 0 0 0 0

3.5724 0.3990 0.0000 C 0 0 0 0 0 0 0 0 0 0 0 0

-3.5724 0.3990 0.0000 C 0 0 0 0 0 0 0 0 0 0 0 0

-4.2868 -0.0135 0.0000 C 0 0 0 0 0 0 0 0 0 0 0 0

-4.2868 -0.8385 0.0000 C 0 0 0 0 0 0 0 0 0 0 0 0
-3.5724 -1.2510 0.0000 C 0 0 0 0 0 0 0 0 0 0 0 0
-2.8579 -0.8385 0.0000 C 0 0 0 0 0 0 0 0 0 0 0 0
5.0013 -1.2510 0.0000 C 0 0 0 0 0 0 0 0 0 0 0 0
-5.0013 -1.2510 0.0000 C 0 0 0 0 0 0 0 0 0 0 0 0
1 2 1 0
2 3 1 1
2 4 1 6
2 5 1 0
5 6 1 0
6 7 1 0
7 8 1 0
8 9 1 0
9 10 1 0
10 11 1 0
10 12 1 1
10 13 1 6
11 14 2 0
14 15 1 0
15 16 2 0
16 17 1 0
17 18 2 0
18 11 1 0
1 19 2 0
19 20 1 0
20 21 2 0
21 22 1 0
22 23 2 0
23 1 1 0
16 24 1 0
21 25 1 0

M RAD 2 4 1 12 1
M END

Compound **28(*rac*+*meso*)**:

ChemDraw07102422282D

```
23 24  0  0  0  0  0  0  0  0999 V2000
  -2.8579    0.0000    0.0000 C   0  0  0  0  0  0  0  0  0  0  0  0
  -2.1434    0.4125    0.0000 S   0  0  0  0  0  0  0  0  0  0  0  0
  -1.4289    0.0000    0.0000 C   0  0  0  0  0  0  0  0  0  0  0  0
  -0.7145    0.4125    0.0000 C   0  0  0  0  0  0  0  0  0  0  0  0
   0.0000    0.0000    0.0000 S   0  0  0  0  0  0  0  0  0  0  0  0
   0.7145    0.4125    0.0000 C   0  0  0  0  0  0  0  0  0  0  0  0
   1.4289    0.0000    0.0000 C   0  0  0  0  0  0  0  0  0  0  0  0
   2.1434    0.4125    0.0000 S   0  0  0  0  0  0  0  0  0  0  0  0
   2.8579    0.0000    0.0000 C   0  0  0  0  0  0  0  0  0  0  0  0
  -2.1434    1.2375    0.0000 O   0  0  0  0  0  0  0  0  0  0  0  0
   2.1434    1.2375    0.0000 O   0  0  0  0  0  0  0  0  0  0  0  0
   2.8579   -0.8250    0.0000 C   0  0  0  0  0  0  0  0  0  0  0  0
   3.5724   -1.2375    0.0000 C   0  0  0  0  0  0  0  0  0  0  0  0
   4.2868   -0.8250    0.0000 C   0  0  0  0  0  0  0  0  0  0  0  0
   4.2868    0.0000    0.0000 C   0  0  0  0  0  0  0  0  0  0  0  0
   3.5724    0.4125    0.0000 C   0  0  0  0  0  0  0  0  0  0  0  0
  -3.5724    0.4125    0.0000 C   0  0  0  0  0  0  0  0  0  0  0  0
  -4.2868    0.0000    0.0000 C   0  0  0  0  0  0  0  0  0  0  0  0
  -4.2868   -0.8250    0.0000 C   0  0  0  0  0  0  0  0  0  0  0  0
  -3.5724   -1.2375    0.0000 C   0  0  0  0  0  0  0  0  0  0  0  0
  -2.8579   -0.8250    0.0000 C   0  0  0  0  0  0  0  0  0  0  0  0
   5.0013   -1.2375    0.0000 C   0  0  0  0  0  0  0  0  0  0  0  0
  -5.0013   -1.2375    0.0000 C   0  0  0  0  0  0  0  0  0  0  0  0
  1  2  1  0
```

2 3 1 0

3 4 1 0

4 5 1 0

5 6 1 0

6 7 1 0

7 8 1 0

8 9 1 0

2 10 1 4

8 11 1 4

9 12 2 0

12 13 1 0

13 14 2 0

14 15 1 0

15 16 2 0

16 9 1 0

1 17 2 0

17 18 1 0

18 19 2 0

19 20 1 0

20 21 2 0

21 1 1 0

14 22 1 0

19 23 1 0

M SUB 2 2 4 8 4

M END

<u>Compound **29(*R*S,*R*S)**:</u>

ChemDraw07102422292D

19 18 0 0 1 0 0 0 0 0 0999 V2000

-2.8579 -0.2338 0.0000 C 0 0 0 0 0 0 0 0 0 0 0 0

```
 -2.1434    0.1787    0.0000 S   0  0  0  0  0  0  0  0  0  0  0  0
 -2.5559    0.8932    0.0000 C   0  0  0  0  0  0  0  0  0  0  0  0
 -1.7997    1.0307    0.0000 O   0  0  0  0  0  0  0  0  0  0  0  0
 -1.4289   -0.2338    0.0000 C   0  0  0  0  0  0  0  0  0  0  0  0
 -0.7145    0.1787    0.0000 C   0  0  0  0  0  0  0  0  0  0  0  0
 -0.0000   -0.2338    0.0000 S   0  0  0  0  0  0  0  0  0  0  0  0
  0.7145    0.1787    0.0000 C   0  0  0  0  0  0  0  0  0  0  0  0
  1.4289   -0.2338    0.0000 C   0  0  0  0  0  0  0  0  0  0  0  0
  2.1434    0.1787    0.0000 S   0  0  0  0  0  0  0  0  0  0  0  0
  2.8579   -0.2338    0.0000 C   0  0  0  0  0  0  0  0  0  0  0  0
  1.7309    0.8932    0.0000 O   0  0  0  0  0  0  0  0  0  0  0  0
  2.5559    0.8932    0.0000 C   0  0  0  0  0  0  0  0  0  0  0  0
 -3.5724    0.1787    0.0000 C   0  0  0  0  0  0  0  0  0  0  0  0
 -2.6444   -1.0307    0.0000 C   0  0  0  0  0  0  0  0  0  0  0  0
 -3.5724   -0.6463    0.0000 C   0  0  0  0  0  0  0  0  0  0  0  0
  3.5724    0.1787    0.0000 C   0  0  0  0  0  0  0  0  0  0  0  0
  2.6444   -1.0307    0.0000 C   0  0  0  0  0  0  0  0  0  0  0  0
  3.5724   -0.6463    0.0000 C   0  0  0  0  0  0  0  0  0  0  0  0
  1  2  1  0
  2  3  1  1
  2  4  1  6
  2  5  1  0
  5  6  1  0
  6  7  1  0
  7  8  1  0
  8  9  1  0
  9 10  1  0
 10 11  1  0
 10 12  1  1
 10 13  1  6
  1 14  1  0
```

1 15 1 0

1 16 1 0

11 17 1 0

11 18 1 0

11 19 1 0

M RAD 2 3 1 13 1

M END

Compound **29(*S*S,*S*S)**:

ChemDraw07102422292D

19 18 0 0 1 0 0 0 0 0999 V2000

-2.8579 -0.2338 0.0000 C 0 0 0 0 0 0 0 0 0 0 0 0

-2.1434 0.1787 0.0000 S 0 0 0 0 0 0 0 0 0 0 0 0

-2.5559 0.8932 0.0000 O 0 0 0 0 0 0 0 0 0 0 0 0

-1.7997 1.0307 0.0000 C 0 0 0 0 0 0 0 0 0 0 0 0

-1.4289 -0.2338 0.0000 C 0 0 0 0 0 0 0 0 0 0 0 0

-0.7145 0.1787 0.0000 C 0 0 0 0 0 0 0 0 0 0 0 0

-0.0000 -0.2338 0.0000 S 0 0 0 0 0 0 0 0 0 0 0 0

0.7145 0.1787 0.0000 C 0 0 0 0 0 0 0 0 0 0 0 0

1.4289 -0.2338 0.0000 C 0 0 0 0 0 0 0 0 0 0 0 0

2.1434 0.1787 0.0000 S 0 0 0 0 0 0 0 0 0 0 0 0

2.8579 -0.2338 0.0000 C 0 0 0 0 0 0 0 0 0 0 0 0

1.7309 0.8932 0.0000 C 0 0 0 0 0 0 0 0 0 0 0 0

2.5559 0.8932 0.0000 O 0 0 0 0 0 0 0 0 0 0 0 0

-3.5724 0.1787 0.0000 C 0 0 0 0 0 0 0 0 0 0 0 0

-2.6444 -1.0307 0.0000 C 0 0 0 0 0 0 0 0 0 0 0 0

-3.5724 -0.6463 0.0000 C 0 0 0 0 0 0 0 0 0 0 0 0

3.5724 0.1787 0.0000 C 0 0 0 0 0 0 0 0 0 0 0 0

2.6444 -1.0307 0.0000 C 0 0 0 0 0 0 0 0 0 0 0 0

3.5724 -0.6463 0.0000 C 0 0 0 0 0 0 0 0 0 0 0 0

1 2 1 0
2 3 1 1
2 4 1 6
2 5 1 0
5 6 1 0
6 7 1 0
7 8 1 0
8 9 1 0
9 10 1 0
10 11 1 0
10 12 1 1
10 13 1 6
1 14 1 0
1 15 1 0
1 16 1 0
11 17 1 0
11 18 1 0
11 19 1 0
M RAD 2 4 1 12 1
M END

Compound **29(*rac*+*meso*)**:

ChemDraw07102422302D

17 16 0 0 0 0 0 0 0 0999 V2000
-2.8579 -0.2203 0.0000 C 0 0 0 0 0 0 0 0 0 0 0 0
-2.1434 0.1922 0.0000 S 0 0 0 0 0 0 0 0 0 0 0 0
-1.4289 -0.2203 0.0000 C 0 0 0 0 0 0 0 0 0 0 0 0
-0.7145 0.1922 0.0000 C 0 0 0 0 0 0 0 0 0 0 0 0
-0.0000 -0.2203 0.0000 S 0 0 0 0 0 0 0 0 0 0 0 0

```
  0.7145    0.1922    0.0000 C   0  0  0  0  0  0  0  0  0  0  0  0
  1.4289   -0.2203    0.0000 C   0  0  0  0  0  0  0  0  0  0  0  0
  2.1434    0.1922    0.0000 S   0  0  0  0  0  0  0  0  0  0  0  0
  2.8579   -0.2203    0.0000 C   0  0  0  0  0  0  0  0  0  0  0  0
 -2.1434    1.0172    0.0000 O   0  0  0  0  0  0  0  0  0  0  0  0
  2.1434    1.0172    0.0000 O   0  0  0  0  0  0  0  0  0  0  0  0
  3.5724    0.1922    0.0000 C   0  0  0  0  0  0  0  0  0  0  0  0
  2.6444   -1.0172    0.0000 C   0  0  0  0  0  0  0  0  0  0  0  0
  3.5724   -0.6328    0.0000 C   0  0  0  0  0  0  0  0  0  0  0  0
 -3.5724    0.1922    0.0000 C   0  0  0  0  0  0  0  0  0  0  0  0
 -2.6444   -1.0172    0.0000 C   0  0  0  0  0  0  0  0  0  0  0  0
 -3.5724   -0.6328    0.0000 C   0  0  0  0  0  0  0  0  0  0  0  0
  1  2  1  0
  2  3  1  0
  3  4  1  0
  4  5  1  0
  5  6  1  0
  6  7  1  0
  7  8  1  0
  8  9  1  0
  2 10  1  4
  8 11  1  4
  9 12  1  0
  9 13  1  0
  9 14  1  0
  1 15  1  0
  1 16  1  0
  1 17  1  0
M  SUB  2   2   4   8   4
M  END
```

Compound **30(*S*S,*S*S)**:

```
  ChemDraw07102422312D

 13 12  0  0  1  0  0  0  0  0999 V2000
   -2.1434   -0.2197    0.0000 S   0  0  0  0  0  0  0  0  0  0  0  0
   -2.5559    0.4947    0.0000 C   0  0  0  0  0  0  0  0  0  0  0  0
   -1.7997    0.6322    0.0000 O   0  0  0  0  0  0  0  0  0  0  0  0
   -1.4289   -0.6322    0.0000 C   0  0  0  0  0  0  0  0  0  0  0  0
   -0.7145   -0.2197    0.0000 C   0  0  0  0  0  0  0  0  0  0  0  0
   -0.0000   -0.6322    0.0000 S   0  0  0  0  0  0  0  0  0  0  0  0
    0.7145   -0.2197    0.0000 C   0  0  0  0  0  0  0  0  0  0  0  0
    1.4289   -0.6322    0.0000 C   0  0  0  0  0  0  0  0  0  0  0  0
    2.1434   -0.2197    0.0000 S   0  0  0  0  0  0  0  0  0  0  0  0
    1.7309    0.4947    0.0000 O   0  0  0  0  0  0  0  0  0  0  0  0
    2.5559    0.4947    0.0000 C   0  0  0  0  0  0  0  0  0  0  0  0
   -2.8579   -0.6322    0.0000 C   0  0  0  0  0  0  0  0  0  0  0  0
    2.8579   -0.6322    0.0000 C   0  0  0  0  0  0  0  0  0  0  0  0
  1 12  1  0
  9 13  1  0
  1  2  1  1
  1  3  1  6
  1  4  1  0
  4  5  1  0
  5  6  1  0
  6  7  1  0
  7  8  1  0
  8  9  1  0
  9 10  1  1
  9 11  1  6
M  RAD  2   2   1  11   1
M  STY  1   1 SUP
```

```
M  SLB  1   1   1
M  SAL   1  1  12
M  SBL   1  1   1
M  SMT   1 Me
M  SBV   1   1    0.7145    0.4125
M  STY  1   2 SUP
M  SLB  1   2   2
M  SAL   2  1  13
M  SBL   2  1   2
M  SMT   2 Me
M  SBV   2   2   -0.7145    0.4125
M  END
```

Compound **30(*R*S,*R*S)**:

```
  ChemDraw07102422312D

 13 12  0  0  1  0  0  0  0  0999 V2000
   -2.1434   -0.2197    0.0000 S   0  0  0  0  0  0  0  0  0  0  0  0
   -2.5559    0.4947    0.0000 O   0  0  0  0  0  0  0  0  0  0  0  0
   -1.7997    0.6322    0.0000 C   0  0  0  0  0  0  0  0  0  0  0  0
   -1.4289   -0.6322    0.0000 C   0  0  0  0  0  0  0  0  0  0  0  0
   -0.7145   -0.2197    0.0000 C   0  0  0  0  0  0  0  0  0  0  0  0
   -0.0000   -0.6322    0.0000 S   0  0  0  0  0  0  0  0  0  0  0  0
    0.7145   -0.2197    0.0000 C   0  0  0  0  0  0  0  0  0  0  0  0
    1.4289   -0.6322    0.0000 C   0  0  0  0  0  0  0  0  0  0  0  0
    2.1434   -0.2197    0.0000 S   0  0  0  0  0  0  0  0  0  0  0  0
    1.7309    0.4947    0.0000 C   0  0  0  0  0  0  0  0  0  0  0  0
    2.5559    0.4947    0.0000 O   0  0  0  0  0  0  0  0  0  0  0  0
   -2.8579   -0.6322    0.0000 C   0  0  0  0  0  0  0  0  0  0  0  0
    2.8579   -0.6322    0.0000 C   0  0  0  0  0  0  0  0  0  0  0  0
  1 12  1  0
```

9 13 1 0
1 2 1 1
1 3 1 6
1 4 1 0
4 5 1 0
5 6 1 0
6 7 1 0
7 8 1 0
8 9 1 0
9 10 1 1
9 11 1 6
M RAD 2 3 1 10 1
M STY 1 1 SUP
M SLB 1 1 1
M SAL 1 1 12
M SBL 1 1 1
M SMT 1 Me
M SBV 1 1 0.7145 0.4125
M STY 1 2 SUP
M SLB 1 2 2
M SAL 2 1 13
M SBL 2 1 2
M SMT 2 Me
M SBV 2 2 -0.7145 0.4125
M END

Compound **30(*rac*+*meso*)**:

ChemDraw07102422322D

11 10 0 0 0 0 0 0 0 0999 V2000
-2.1434 -0.2062 0.0000 S 0 0 0 0 0 0 0 0 0 0 0 0

```
   -1.4289   -0.6187    0.0000 C   0  0  0  0  0  0  0  0  0  0  0  0
   -0.7145   -0.2062    0.0000 C   0  0  0  0  0  0  0  0  0  0  0  0
   -0.0000   -0.6187    0.0000 S   0  0  0  0  0  0  0  0  0  0  0  0
    0.7145   -0.2062    0.0000 C   0  0  0  0  0  0  0  0  0  0  0  0
    1.4289   -0.6187    0.0000 C   0  0  0  0  0  0  0  0  0  0  0  0
    2.1434   -0.2062    0.0000 S   0  0  0  0  0  0  0  0  0  0  0  0
   -2.1434    0.6187    0.0000 O   0  0  0  0  0  0  0  0  0  0  0  0
    2.1434    0.6187    0.0000 O   0  0  0  0  0  0  0  0  0  0  0  0
   -2.8579   -0.6187    0.0000 C   0  0  0  0  0  0  0  0  0  0  0  0
    2.8579   -0.6187    0.0000 C   0  0  0  0  0  0  0  0  0  0  0  0
  1 10  1  0
  7 11  1  0
  1  2  1  0
  2  3  1  0
  3  4  1  0
  4  5  1  0
  5  6  1  0
  6  7  1  0
  1  8  1  4
  7  9  1  4
M  SUB  2   1   4   7   4
M  STY  1   1 SUP
M  SLB  1   1   1
M  SAL   1  1  10
M  SBL   1  1   1
M  SMT   1 Me
M  SBV   1   1    0.7145    0.4125
M  STY  1   2 SUP
M  SLB  1   2   2
M  SAL   2  1  11
M  SBL   2  1   2
```

M SMT   2 Me

M SBV   2   2   -0.7145   0.4125

M END

Compound **33(*R*S,*R*S)**:

ChemDraw07102422332D

```
 26 27  0  0  1  0  0  0  0  0  0999 V2000
   -2.8579   -0.0135    0.0000 C   0  0  0  0  0  0  0  0  0  0  0  0
   -2.1434    0.3990    0.0000 S   0  0  0  0  0  0  0  0  0  0  0  0
   -2.5559    1.1135    0.0000 C   0  0  0  0  0  0  0  0  0  0  0  0
   -1.7997    1.2510    0.0000 O   0  0  0  0  0  0  0  0  0  0  0  0
   -1.4289   -0.0135    0.0000 C   0  0  0  0  0  0  0  0  0  0  0  0
   -0.7145    0.3990    0.0000 C   0  0  0  0  0  0  0  0  0  0  0  0
    0.0000   -0.0135    0.0000 S   0  0  0  0  0  0  0  0  0  0  0  0
    0.7145    0.3990    0.0000 C   0  0  0  0  0  0  0  0  0  0  0  0
    1.4289   -0.0135    0.0000 C   0  0  0  0  0  0  0  0  0  0  0  0
    2.1434    0.3990    0.0000 S   0  0  0  0  0  0  0  0  0  0  0  0
    2.8579   -0.0135    0.0000 C   0  0  0  0  0  0  0  0  0  0  0  0
    1.7309    1.1135    0.0000 O   0  0  0  0  0  0  0  0  0  0  0  0
    2.5559    1.1135    0.0000 C   0  0  0  0  0  0  0  0  0  0  0  0
    2.8579   -0.8385    0.0000 C   0  0  0  0  0  0  0  0  0  0  0  0
    3.5724   -1.2510    0.0000 C   0  0  0  0  0  0  0  0  0  0  0  0
    4.2868   -0.8385    0.0000 C   0  0  0  0  0  0  0  0  0  0  0  0
    4.2868   -0.0135    0.0000 C   0  0  0  0  0  0  0  0  0  0  0  0
    3.5724    0.3990    0.0000 C   0  0  0  0  0  0  0  0  0  0  0  0
   -3.5724    0.3990    0.0000 C   0  0  0  0  0  0  0  0  0  0  0  0
   -4.2868   -0.0135    0.0000 C   0  0  0  0  0  0  0  0  0  0  0  0
   -4.2868   -0.8385    0.0000 C   0  0  0  0  0  0  0  0  0  0  0  0
   -3.5724   -1.2510    0.0000 C   0  0  0  0  0  0  0  0  0  0  0  0
   -2.8579   -0.8385    0.0000 C   0  0  0  0  0  0  0  0  0  0  0  0
```

```
    5.0013   -1.2510    0.0000 C   0  0  0  0  0  0  0  0  0  0  0  0
   -5.0013   -1.2510    0.0000 C   0  0  0  0  0  0  0  0  0  0  0  0
    0.0000   -0.8385    0.0000 O   0  0  0  0  0  0  0  0  0  0  0  0
  1  2  1  0
  2  3  1  1
  2  4  1  6
  2  5  1  0
  5  6  1  0
  6  7  1  0
  7  8  1  0
  8  9  1  0
  9 10  1  0
 10 11  1  0
 10 12  1  1
 10 13  1  6
 11 14  2  0
 14 15  1  0
 15 16  2  0
 16 17  1  0
 17 18  2  0
 18 11  1  0
  1 19  2  0
 19 20  1  0
 20 21  2  0
 21 22  1  0
 22 23  2  0
 23  1  1  0
 16 24  1  0
 21 25  1  0
  7 26  1  1
M  RAD  2   3   1  13   1
```

M END

Compound **34(*R*S,*R*S)**:

ChemDraw07102422342D

```
 20 19  0  0  1  0  0  0  0  0999 V2000
   -2.8579   -0.2197    0.0000 C   0  0  0  0  0  0  0  0  0  0  0  0
   -2.1434    0.1928    0.0000 S   0  0  0  0  0  0  0  0  0  0  0  0
   -2.5559    0.9072    0.0000 C   0  0  0  0  0  0  0  0  0  0  0  0
   -1.7997    1.0447    0.0000 O   0  0  0  0  0  0  0  0  0  0  0  0
   -1.4289   -0.2197    0.0000 C   0  0  0  0  0  0  0  0  0  0  0  0
   -0.7145    0.1928    0.0000 C   0  0  0  0  0  0  0  0  0  0  0  0
   -0.0000   -0.2197    0.0000 S   0  0  0  0  0  0  0  0  0  0  0  0
    0.7145    0.1928    0.0000 C   0  0  0  0  0  0  0  0  0  0  0  0
    1.4289   -0.2197    0.0000 C   0  0  0  0  0  0  0  0  0  0  0  0
    2.1434    0.1928    0.0000 S   0  0  0  0  0  0  0  0  0  0  0  0
    2.8579   -0.2197    0.0000 C   0  0  0  0  0  0  0  0  0  0  0  0
    1.7309    0.9072    0.0000 O   0  0  0  0  0  0  0  0  0  0  0  0
    2.5559    0.9072    0.0000 C   0  0  0  0  0  0  0  0  0  0  0  0
   -3.5724    0.1928    0.0000 C   0  0  0  0  0  0  0  0  0  0  0  0
   -2.6444   -1.0166    0.0000 C   0  0  0  0  0  0  0  0  0  0  0  0
   -3.5724   -0.6322    0.0000 C   0  0  0  0  0  0  0  0  0  0  0  0
    3.5724    0.1928    0.0000 C   0  0  0  0  0  0  0  0  0  0  0  0
    2.6444   -1.0166    0.0000 C   0  0  0  0  0  0  0  0  0  0  0  0
    3.5724   -0.6322    0.0000 C   0  0  0  0  0  0  0  0  0  0  0  0
   -0.0000   -1.0447    0.0000 O   0  0  0  0  0  0  0  0  0  0  0  0
  1  2  1  0
  2  3  1  1
  2  4  1  6
  2  5  1  0
  5  6  1  0
```

6 7 1 0

7 8 1 0

8 9 1 0

9 10 1 0

10 11 1 0

10 12 1 1

10 13 1 6

1 14 1 0

1 15 1 0

1 16 1 0

11 17 1 0

11 18 1 0

11 19 1 0

7 20 1 1

M RAD 2 3 1 13 1

M END

Compound **34(*S*$_S$,*S*$_S$)**:

ChemDraw07102422352D

20 19 0 0 1 0 0 0 0 0999 V2000

-2.8579 -0.2197 0.0000 C 0 0 0 0 0 0 0 0 0 0 0 0

-2.1434 0.1928 0.0000 S 0 0 0 0 0 0 0 0 0 0 0 0

-2.5559 0.9072 0.0000 O 0 0 0 0 0 0 0 0 0 0 0 0

-1.7997 1.0447 0.0000 C 0 0 0 0 0 0 0 0 0 0 0 0

-1.4289 -0.2197 0.0000 C 0 0 0 0 0 0 0 0 0 0 0 0

-0.7145 0.1928 0.0000 C 0 0 0 0 0 0 0 0 0 0 0 0

-0.0000 -0.2197 0.0000 S 0 0 0 0 0 0 0 0 0 0 0 0

0.7145 0.1928 0.0000 C 0 0 0 0 0 0 0 0 0 0 0 0

1.4289 -0.2197 0.0000 C 0 0 0 0 0 0 0 0 0 0 0 0

2.1434 0.1928 0.0000 S 0 0 0 0 0 0 0 0 0 0 0 0

```
    2.8579   -0.2197    0.0000 C   0  0  0  0  0  0  0  0  0  0  0  0
    1.7309    0.9072    0.0000 C   0  0  0  0  0  0  0  0  0  0  0  0
    2.5559    0.9072    0.0000 O   0  0  0  0  0  0  0  0  0  0  0  0
   -3.5724    0.1928    0.0000 C   0  0  0  0  0  0  0  0  0  0  0  0
   -2.6444   -1.0166    0.0000 C   0  0  0  0  0  0  0  0  0  0  0  0
   -3.5724   -0.6322    0.0000 C   0  0  0  0  0  0  0  0  0  0  0  0
    3.5724    0.1928    0.0000 C   0  0  0  0  0  0  0  0  0  0  0  0
    2.6444   -1.0166    0.0000 C   0  0  0  0  0  0  0  0  0  0  0  0
    3.5724   -0.6322    0.0000 C   0  0  0  0  0  0  0  0  0  0  0  0
   -0.0000   -1.0447    0.0000 O   0  0  0  0  0  0  0  0  0  0  0  0
  1  2  1  0
  2  3  1  1
  2  4  1  6
  2  5  1  0
  5  6  1  0
  6  7  1  0
  7  8  1  0
  8  9  1  0
  9 10  1  0
 10 11  1  0
 10 12  1  1
 10 13  1  6
  1 14  1  0
  1 15  1  0
  1 16  1  0
 11 17  1  0
 11 18  1  0
 11 19  1  0
  7 20  1  1
M  RAD  2   4   1  12   1
M  END
```

Compound **35(*S*S,*S*S)**:

```
ChemDraw07102422352D

14 13  0  0  1  0  0  0  0  0999 V2000
  -2.1434    0.1928    0.0000 S   0  0  0  0  0  0  0  0  0  0  0  0
  -2.5559    0.9072    0.0000 C   0  0  0  0  0  0  0  0  0  0  0  0
  -1.7997    1.0447    0.0000 O   0  0  0  0  0  0  0  0  0  0  0  0
  -1.4289   -0.2197    0.0000 C   0  0  0  0  0  0  0  0  0  0  0  0
  -0.7145    0.1928    0.0000 C   0  0  0  0  0  0  0  0  0  0  0  0
  -0.0000   -0.2197    0.0000 S   0  0  0  0  0  0  0  0  0  0  0  0
   0.7145    0.1928    0.0000 C   0  0  0  0  0  0  0  0  0  0  0  0
   1.4289   -0.2197    0.0000 C   0  0  0  0  0  0  0  0  0  0  0  0
   2.1434    0.1928    0.0000 S   0  0  0  0  0  0  0  0  0  0  0  0
   1.7309    0.9072    0.0000 O   0  0  0  0  0  0  0  0  0  0  0  0
   2.5559    0.9072    0.0000 C   0  0  0  0  0  0  0  0  0  0  0  0
  -0.0000   -1.0447    0.0000 O   0  0  0  0  0  0  0  0  0  0  0  0
  -2.8579   -0.2197    0.0000 C   0  0  0  0  0  0  0  0  0  0  0  0
   2.8579   -0.2197    0.0000 C   0  0  0  0  0  0  0  0  0  0  0  0
  1 13  1  0
  9 14  1  0
  1  2  1  1
  1  3  1  6
  1  4  1  0
  4  5  1  0
  5  6  1  0
  6  7  1  0
  7  8  1  0
  8  9  1  0
  9 10  1  1
  9 11  1  6
```

```
  6 12  1  1
M  RAD  2   2   1  11   1
M  STY  1   1 SUP
M  SLB  1   1   1
M  SAL   1  1  13
M  SBL   1  1   1
M  SMT   1 Me
M  SBV   1   1    0.7145    0.4125
M  STY  1   2 SUP
M  SLB  1   2   2
M  SAL   2  1  14
M  SBL   2  1   2
M  SMT   2 Me
M  SBV   2   2   -0.7145    0.4125
M  END
```

<u>Compound **12(*S*$_S$,*S*$_S$)·Pd(TFA)$_2$**:</u>

```
  ChemDraw07102422372D

 34 36  0  0  1  0  0  0  0  0  0999 V2000
   -1.0459    1.5726    0.0000 C   0  0  0  0  0  0  0  0  0  0  0  0
   -1.0459    0.8507    0.0000 S   0  0  0  0  0  0  0  0  0  0  0  0
   -0.1252   -0.2149    0.0000 Pd  0  0  0  0  0  0  0  0  0  0  0  0
    0.8987    0.8507    0.0000 S   0  0  0  0  0  0  0  0  0  0  0  0
    0.8987    1.5726    0.0000 C   0  0  0  0  0  0  0  0  0  0  0  0
   -0.0564    2.0882    0.0000 C   0  0  0  0  0  0  0  0  0  0  0  0
    0.8299   -0.9712    0.0000 C   0  0  0  0  0  0  0  0  0  0  0  0
   -0.9771   -0.9368    0.0000 C   0  0  0  0  0  0  0  0  0  0  0  0
   -1.8428    0.6372    0.0000 C   0  0  0  0  0  0  0  0  0  0  0  0
   -1.7604    1.2632    0.0000 O   0  0  0  0  0  0  0  0  0  0  0  0
    1.7507    0.4038    0.0000 O   0  0  0  0  0  0  0  0  0  0  0  0
```

1.6819	1.2632	0.0000	C	0	0	0	0	0	0	0	0	0	0	0	0
1.5969	-0.6671	0.0000	O	0	0	0	0	0	0	0	0	0	0	0	0
-1.6506	-0.6927	0.0000	O	0	0	0	0	0	0	0	0	0	0	0	0
-2.4261	1.2205	0.0000	C	0	0	0	0	0	0	0	0	0	0	0	0
-2.2126	2.0174	0.0000	C	0	0	0	0	0	0	0	0	0	0	0	0
-2.7960	2.6008	0.0000	C	0	0	0	0	0	0	0	0	0	0	0	0
-3.5929	2.3872	0.0000	C	0	0	0	0	0	0	0	0	0	0	0	0
-3.8064	1.5904	0.0000	C	0	0	0	0	0	0	0	0	0	0	0	0
-3.2230	1.0070	0.0000	C	0	0	0	0	0	0	0	0	0	0	0	0
2.3798	0.8232	0.0000	C	0	0	0	0	0	0	0	0	0	0	0	0
2.3323	-0.0004	0.0000	C	0	0	0	0	0	0	0	0	0	0	0	0
3.0219	-0.4533	0.0000	C	0	0	0	0	0	0	0	0	0	0	0	0
3.7589	-0.0826	0.0000	C	0	0	0	0	0	0	0	0	0	0	0	0
3.8064	0.7410	0.0000	C	0	0	0	0	0	0	0	0	0	0	0	0
3.1168	1.1939	0.0000	C	0	0	0	0	0	0	0	0	0	0	0	0
-0.1036	-1.6677	0.0000	F	0	0	0	0	0	0	0	0	0	0	0	0
0.7098	-1.7874	0.0000	C	0	0	0	0	0	0	0	0	0	0	0	0
0.5901	-2.6008	0.0000	F	0	0	0	0	0	0	0	0	0	0	0	0
1.5232	-1.9071	0.0000	F	0	0	0	0	0	0	0	0	0	0	0	0
-1.6389	-1.8952	0.0000	F	0	0	0	0	0	0	0	0	0	0	0	0
-0.8300	-1.7486	0.0000	C	0	0	0	0	0	0	0	0	0	0	0	0
-0.6833	-2.5575	0.0000	F	0	0	0	0	0	0	0	0	0	0	0	0
-0.0210	-1.6019	0.0000	F	0	0	0	0	0	0	0	0	0	0	0	0

28	29	1	0
28	30	1	0
27	28	1	0
7	28	1	0
32	33	1	0
32	34	1	0
31	32	1	0
8	32	1	0

```
  1  2  1  0
  2  3  1  0
  3  4  1  0
  4  5  1  0
  5  6  1  0
  6  1  1  0
  3  7  1  0
  3  8  1  0
  2  9  1  1
  2 10  1  6
  4 11  1  1
  4 12  1  6
  7 13  2  0
  8 14  2  0
  9 15  1  0
 15 16  2  0
 16 17  1  0
 17 18  2  0
 18 19  1  0
 19 20  2  0
 20 15  1  0
 12 21  1  0
 21 22  2  0
 22 23  1  0
 23 24  2  0
 24 25  1  0
 25 26  2  0
 26 21  1  0
M  STY  1   1 SUP
M  SLB  1   1   1
M  SAL   1  4  27  28  29  30
```

M SBL 1 1 4

M SMT 1 CF3

M SBV 1 4 0.1201 0.8162

M STY 1 2 SUP

M SLB 1 2 2

M SAL 2 4 31 32 33 34

M SBL 2 1 8

M SMT 2 CF3

M SBV 2 8 -0.1472 0.8118

M END

Compound **8(*S*$_{S}$,*S*$_{S}$)·Pd(TFA)$_{2}$**:

ChemDraw07102422372D

32 34 0 0 1 0 0 0 0 0999 V2000

-0.9129 1.6522 0.0000 C 0 0 0 0 0 0 0 0 0 0 0 0

-0.9129 0.9303 0.0000 S 0 0 0 0 0 0 0 0 0 0 0 0

0.0078 -0.1353 0.0000 Pd 0 0 0 0 0 0 0 0 0 0 0 0

1.0317 0.9303 0.0000 S 0 0 0 0 0 0 0 0 0 0 0 0

1.0317 1.6522 0.0000 C 0 0 0 0 0 0 0 0 0 0 0 0

0.0766 2.1678 0.0000 C 0 0 0 0 0 0 0 0 0 0 0 0

0.9629 -0.8916 0.0000 C 0 0 0 0 0 0 0 0 0 0 0 0

-0.8441 -0.8572 0.0000 C 0 0 0 0 0 0 0 0 0 0 0 0

-1.7098 0.7168 0.0000 C 0 0 0 0 0 0 0 0 0 0 0 0

-1.6274 1.3428 0.0000 O 0 0 0 0 0 0 0 0 0 0 0 0

1.8836 0.4834 0.0000 O 0 0 0 0 0 0 0 0 0 0 0 0

1.8149 1.3428 0.0000 C 0 0 0 0 0 0 0 0 0 0 0 0

1.7298 -0.5875 0.0000 O 0 0 0 0 0 0 0 0 0 0 0 0

2.4952 0.8761 0.0000 C 0 0 0 0 0 0 0 0 0 0 0 0

3.2395 1.2319 0.0000 C 0 0 0 0 0 0 0 0 0 0 0 0

3.3036 2.0544 0.0000 C 0 0 0 0 0 0 0 0 0 0 0 0

2.6233	2.5211	0.0000	C	0	0	0	0	0	0	0	0	0	0	0	0
1.8789	2.1653	0.0000	C	0	0	0	0	0	0	0	0	0	0	0	0
-1.5176	-0.6131	0.0000	O	0	0	0	0	0	0	0	0	0	0	0	0
-2.2931	1.3002	0.0000	C	0	0	0	0	0	0	0	0	0	0	0	0
-3.0900	1.0866	0.0000	C	0	0	0	0	0	0	0	0	0	0	0	0
-3.3036	0.2897	0.0000	C	0	0	0	0	0	0	0	0	0	0	0	0
-2.7202	-0.2936	0.0000	C	0	0	0	0	0	0	0	0	0	0	0	0
-1.9233	-0.0801	0.0000	C	0	0	0	0	0	0	0	0	0	0	0	0
0.0294	-1.5881	0.0000	F	0	0	0	0	0	0	0	0	0	0	0	0
0.8428	-1.7078	0.0000	C	0	0	0	0	0	0	0	0	0	0	0	0
0.7231	-2.5211	0.0000	F	0	0	0	0	0	0	0	0	0	0	0	0
1.6562	-1.8275	0.0000	F	0	0	0	0	0	0	0	0	0	0	0	0
-1.5059	-1.8156	0.0000	F	0	0	0	0	0	0	0	0	0	0	0	0
-0.6970	-1.6690	0.0000	C	0	0	0	0	0	0	0	0	0	0	0	0
-0.5503	-2.4779	0.0000	F	0	0	0	0	0	0	0	0	0	0	0	0
0.1120	-1.5223	0.0000	F	0	0	0	0	0	0	0	0	0	0	0	0

26	27	1	0
26	28	1	0
25	26	1	0
7	26	1	0
30	31	1	0
30	32	1	0
29	30	1	0
8	30	1	0
1	2	1	0
2	3	1	0
3	4	1	0
4	5	1	0
5	6	1	0
6	1	1	0
3	7	1	0

```
  3  8  1  0
  2  9  1  1
  2 10  1  6
  4 11  1  1
  4 12  1  6
  7 13  2  0
 12 14  2  0
 14 15  1  0
 15 16  2  0
 16 17  1  0
 17 18  2  0
 18 12  1  0
  8 19  2  0
  9 20  2  0
 20 21  1  0
 21 22  2  0
 22 23  1  0
 23 24  2  0
 24  9  1  0
M  STY  1   1 SUP
M  SLB  1   1   1
M  SAL   1  4  25  26  27  28
M  SBL   1  1    4
M  SMT   1 CF3
M  SBV   1    4    0.1201    0.8162
M  STY  1   2 SUP
M  SLB  1   2   2
M  SAL   2  4  29  30  31  32
M  SBL   2  1    8
M  SMT   2 CF3
M  SBV   2    8   -0.1472    0.8118
```

M END

Compound **16(*R*S,*R*S)·Pd(TFA)$_2$**:

```
  ChemDraw07102422382D

 26 26  0  0  1  0  0  0  0  0999 V2000
   -0.9893    1.8289    0.0000 C   0  0  0  0  0  0  0  0  0  0  0  0
   -0.9893    1.1070    0.0000 S   0  0  0  0  0  0  0  0  0  0  0  0
   -0.0685    0.0414    0.0000 Pd  0  0  0  0  0  0  0  0  0  0  0  0
    0.9553    1.1070    0.0000 S   0  0  0  0  0  0  0  0  0  0  0  0
    0.9553    1.8289    0.0000 C   0  0  0  0  0  0  0  0  0  0  0  0
    0.0002    2.3445    0.0000 C   0  0  0  0  0  0  0  0  0  0  0  0
    0.8866   -0.7149    0.0000 C   0  0  0  0  0  0  0  0  0  0  0  0
   -0.9205   -0.6805    0.0000 C   0  0  0  0  0  0  0  0  0  0  0  0
   -1.7861    0.8935    0.0000 C   0  0  0  0  0  0  0  0  0  0  0  0
   -1.7037    1.5195    0.0000 O   0  0  0  0  0  0  0  0  0  0  0  0
    1.8073    0.6601    0.0000 O   0  0  0  0  0  0  0  0  0  0  0  0
    1.7385    1.5195    0.0000 C   0  0  0  0  0  0  0  0  0  0  0  0
    1.6535   -0.4108    0.0000 O   0  0  0  0  0  0  0  0  0  0  0  0
   -1.5940   -0.4364    0.0000 O   0  0  0  0  0  0  0  0  0  0  0  0
   -2.3695    1.4768    0.0000 C   0  0  0  0  0  0  0  0  0  0  0  0
   -3.1664    1.2633    0.0000 C   0  0  0  0  0  0  0  0  0  0  0  0
    2.4364    1.0795    0.0000 C   0  0  0  0  0  0  0  0  0  0  0  0
    3.1664    1.4640    0.0000 C   0  0  0  0  0  0  0  0  0  0  0  0
   -0.0469   -1.4114    0.0000 F   0  0  0  0  0  0  0  0  0  0  0  0
    0.7664   -1.5311    0.0000 C   0  0  0  0  0  0  0  0  0  0  0  0
```

0.6467	-2.3445	0.0000	F	0	0	0	0	0	0	0	0	0	0	0	0
1.5798	-1.6508	0.0000	F	0	0	0	0	0	0	0	0	0	0	0	0
-1.5823	-1.6389	0.0000	F	0	0	0	0	0	0	0	0	0	0	0	0
-0.7734	-1.4923	0.0000	C	0	0	0	0	0	0	0	0	0	0	0	0
-0.6267	-2.3012	0.0000	F	0	0	0	0	0	0	0	0	0	0	0	0
0.0356	-1.3457	0.0000	F	0	0	0	0	0	0	0	0	0	0	0	0

20 21 1 0

20 22 1 0

19 20 1 0

7 20 1 0

24 25 1 0

24 26 1 0

23 24 1 0

8 24 1 0

1 2 1 0

2 3 1 0

3 4 1 0

4 5 1 0

5 6 1 0

6 1 1 0

3 7 1 0

3 8 1 0

2 9 1 1

2 10 1 6

4 11 1 1

4 12 1 6

7 13 2 0

8 14 2 0

 9 15 1 0
 15 16 1 0
 12 17 1 0
 17 18 1 0
M STY 1   1 SUP
M SLB 1   1   1
M SAL   1 4 19 20 21 22
M SBL   1 1   4
M SMT   1 CF3
M SBV   1   4   0.1201   0.8162
M STY 1   2 SUP
M SLB 1   2   2
M SAL   2 4 23 24 25 26
M SBL   2 1   8
M SMT   2 CF3
M SBV   2   8   -0.1472   0.8118
M END

Compound **36(*R*$_S$,*R*$_S$):**

ChemDraw07102422392D

21 22 0 0 1 0 0 0 0 0999 V2000
0.1141   -0.0203   0.0000 Ru 0 0 0 0 0 0 0 0 0 0 0 0
-0.9969   -0.5130   0.0000 S   0 0 0 0 0 0 0 0 0 0 0 0
-0.9510   0.5182   0.0000 S   0 0 0 0 0 0 0 0 0 0 0 0
1.1344   0.4953   0.0000 S   0 0 0 0 0 0 0 0 0 0 0 0
1.1573   -0.4901   0.0000 S   0 0 0 0 0 0 0 0 0 0 0 0
0.1141   1.2287   0.0000 Cl 0 0 0 0 0 0 0 0 0 0 0 0
0.1141   -1.2808   0.0000 Cl 0 0 0 0 0 0 0 0 0 0 0 0

```
  1.8677    0.5182    0.0000 C   0  0  0  0  0  0  0  0  0  0  0  0
  1.8677   -0.4901    0.0000 C   0  0  0  0  0  0  0  0  0  0  0  0
  2.0969    0.0599    0.0000 C   0  0  0  0  0  0  0  0  0  0  0  0
 -1.7531   -0.5130    0.0000 C   0  0  0  0  0  0  0  0  0  0  0  0
 -1.7760    0.5182    0.0000 C   0  0  0  0  0  0  0  0  0  0  0  0
 -2.0969    0.0141    0.0000 C   0  0  0  0  0  0  0  0  0  0  0  0
  1.8438   -1.0047    0.0000 O   0  0  0  0  0  0  0  0  0  0  0  0
  0.9208    1.2922    0.0000 O   0  0  0  0  0  0  0  0  0  0  0  0
 -1.4094   -1.2275    0.0000 O   0  0  0  0  0  0  0  0  0  0  0  0
 -0.7375    1.3151    0.0000 O   0  0  0  0  0  0  0  0  0  0  0  0
  1.1573   -1.3151    0.0000 C   0  0  0  0  0  0  0  0  0  0  0  0
  1.5469    1.2098    0.0000 C   0  0  0  0  0  0  0  0  0  0  0  0
 -1.3635    1.2327    0.0000 C   0  0  0  0  0  0  0  0  0  0  0  0
 -0.7833   -1.3099    0.0000 C   0  0  0  0  0  0  0  0  0  0  0  0
 5 18  1  1
 4 19  1  6
 3 20  1  1
 2 21  1  6
 1  2  1  1
 1  3  1  6
 1  4  1  6
 1  5  1  1
 1  6  1  0
 1  7  1  0
 4  8  1  0
 5  9  1  0
 9 10  1  0
10  8  1  0
 2 11  1  0
 3 12  1  0
12 13  1  0
```

```
 13 11  1  0
  5 14  1  6
  4 15  1  1
  2 16  1  1
  3 17  1  6
M  STY  1   1 SUP
M  SLB  1   1   1
M  SAL   1  1  18
M  SBL   1  1   1
M  SMT   1 Me
M  SBV   1   1    0.0000    0.8250
M  STY  1   2 SUP
M  SLB  1   2   2
M  SAL   2  1  19
M  SBL   2  1   2
M  SMT   2 Me
M  SBV   2   2   -0.4125   -0.7145
M  STY  1   3 SUP
M  SLB  1   3   3
M  SAL   3  1  20
M  SBL   3  1   3
M  SMT   3 Me
M  SBV   3   3    0.4125   -0.7145
M  STY  1   4 SUP
M  SLB  1   4   4
M  SAL   4  1  21
M  SBL   4  1   4
M  SMT   4 Me
M  SBV   4   4   -0.2135    0.7969
M  END
```

Compound **36(S_S,S_S):**

```
ChemDraw07102422402D

21 22  0  0  1  0  0  0  0  0999 V2000
   0.1141   -0.0203    0.0000 Ru  0  0  0  0  0  0  0  0  0  0  0  0
  -0.9969   -0.5130    0.0000 S   0  0  0  0  0  0  0  0  0  0  0  0
  -0.9510    0.5182    0.0000 S   0  0  0  0  0  0  0  0  0  0  0  0
   1.1344    0.4953    0.0000 S   0  0  0  0  0  0  0  0  0  0  0  0
   1.1573   -0.4901    0.0000 S   0  0  0  0  0  0  0  0  0  0  0  0
   0.1141    1.2287    0.0000 Cl  0  0  0  0  0  0  0  0  0  0  0  0
   0.1141   -1.2808    0.0000 Cl  0  0  0  0  0  0  0  0  0  0  0  0
   1.8677    0.5182    0.0000 C   0  0  0  0  0  0  0  0  0  0  0  0
   1.8677   -0.4901    0.0000 C   0  0  0  0  0  0  0  0  0  0  0  0
   2.0969    0.0599    0.0000 C   0  0  0  0  0  0  0  0  0  0  0  0
  -1.7531   -0.5130    0.0000 C   0  0  0  0  0  0  0  0  0  0  0  0
  -1.7760    0.5182    0.0000 C   0  0  0  0  0  0  0  0  0  0  0  0
  -2.0969    0.0141    0.0000 C   0  0  0  0  0  0  0  0  0  0  0  0
   1.1573   -1.3151    0.0000 O   0  0  0  0  0  0  0  0  0  0  0  0
   1.8488    0.9078    0.0000 O   0  0  0  0  0  0  0  0  0  0  0  0
  -1.3635    1.2327    0.0000 O   0  0  0  0  0  0  0  0  0  0  0  0
  -0.7833   -1.3099    0.0000 O   0  0  0  0  0  0  0  0  0  0  0  0
   1.8438   -1.0047    0.0000 C   0  0  0  0  0  0  0  0  0  0  0  0
   1.0927    1.2922    0.0000 C   0  0  0  0  0  0  0  0  0  0  0  0
  -1.4094   -1.2275    0.0000 C   0  0  0  0  0  0  0  0  0  0  0  0
  -0.7375    1.3151    0.0000 C   0  0  0  0  0  0  0  0  0  0  0  0
  5 18  1  6
  4 19  1  1
  2 20  1  1
  3 21  1  6
  1  2  1  1
  1  3  1  6
```

```
  1  4  1  6
  1  5  1  1
  1  6  1  0
  1  7  1  0
  4  8  1  0
  5  9  1  0
  9 10  1  0
 10  8  1  0
  2 11  1  0
  3 12  1  0
 12 13  1  0
 13 11  1  0
  5 14  1  1
  4 15  1  6
  3 16  1  1
  2 17  1  6
M  STY  1   1 SUP
M  SLB  1   1   1
M  SAL   1  1  18
M  SBL   1  1    1
M  SMT   1 Me
M  SBV   1   1   -0.6865    0.5146
M  STY  1   2 SUP
M  SLB  1   2   2
M  SAL   2  1  19
M  SBL   2  1    2
M  SMT   2 Me
M  SBV   2   2    0.0417   -0.7969
M  STY  1   3 SUP
M  SLB  1   3   3
M  SAL   3  1  20
```

M SBL   3 1   3

M SMT   3 Me

M SBV   3   3   0.4125   0.7145

M STY 1   4 SUP

M SLB 1   4   4

M SAL   4 1 21

M SBL   4 1   4

M SMT   4 Me

M SBV   4   4   -0.2135   -0.7969

M END

<u>Compound **37(*R*$_S$,*R*$_S$):**</u>

ChemDraw07102422412D

25 26 0 0 1 0 0 0 0 0999 V2000

0.1141   -0.0473   0.0000 Ru 0 0 0 0 0 0 0 0 0 0 0 0

-0.9969   -0.5400   0.0000 S   0 0 0 0 0 0 0 0 0 0 0 0

-0.9510   0.4913   0.0000 S   0 0 0 0 0 0 0 0 0 0 0 0

1.1344   0.4684   0.0000 S   0 0 0 0 0 0 0 0 0 0 0 0

1.1573   -0.5171   0.0000 S   0 0 0 0 0 0 0 0 0 0 0 0

0.1141   1.2017   0.0000 Cl 0 0 0 0 0 0 0 0 0 0 0 0

0.1141   -1.3077   0.0000 Cl 0 0 0 0 0 0 0 0 0 0 0 0

1.8677   0.4913   0.0000 C   0 0 0 0 0 0 0 0 0 0 0 0

1.8677   -0.5171   0.0000 C   0 0 0 0 0 0 0 0 0 0 0 0

2.0969   0.0329   0.0000 C   0 0 0 0 0 0 0 0 0 0 0 0

-1.7531   -0.5400   0.0000 C   0 0 0 0 0 0 0 0 0 0 0 0

-1.7760   0.4913   0.0000 C   0 0 0 0 0 0 0 0 0 0 0 0

-2.0969   -0.0129   0.0000 C   0 0 0 0 0 0 0 0 0 0 0 0

1.1573   -1.3421   0.0000 C   0 0 0 0 0 0 0 0 0 0 0 0

1.8438   -1.0317   0.0000 O   0 0 0 0 0 0 0 0 0 0 0 0

1.5469   1.1828   0.0000 C   0 0 0 0 0 0 0 0 0 0 0 0

0.9208	1.2653	0.0000	O	0	0	0	0	0	0	0	0	0	0	0	0
-1.4094	-1.2544	0.0000	O	0	0	0	0	0	0	0	0	0	0	0	0
-1.3635	1.2058	0.0000	C	0	0	0	0	0	0	0	0	0	0	0	0
-0.7375	1.2882	0.0000	O	0	0	0	0	0	0	0	0	0	0	0	0
-0.7833	-1.3369	0.0000	C	0	0	0	0	0	0	0	0	0	0	0	0
-0.9510	1.9202	0.0000	C	0	0	0	0	0	0	0	0	0	0	0	0
-1.3667	-1.9202	0.0000	C	0	0	0	0	0	0	0	0	0	0	0	0
1.8718	-1.7546	0.0000	C	0	0	0	0	0	0	0	0	0	0	0	0
1.1344	1.8973	0.0000	C	0	0	0	0	0	0	0	0	0	0	0	0

1	2	1	1
1	3	1	6
1	4	1	6
1	5	1	1
1	6	1	0
1	7	1	0
4	8	1	0
5	9	1	0
9	10	1	0
10	8	1	0
2	11	1	0
3	12	1	0
12	13	1	0
13	11	1	0
5	14	1	1
5	15	1	6
4	16	1	6
4	17	1	1
2	18	1	1
3	19	1	1
3	20	1	6
2	21	1	6

```
 19 22  1  0
 21 23  1  0
 14 24  1  0
 16 25  1  0
M  END
```

Compound **37(*S*$_S$,*S*$_S$):**

```
  ChemDraw07102422412D

 25 26  0  0  1  0  0  0  0  0999 V2000
    0.1141    0.0014    0.0000 Ru  0  0  0  0  0  0  0  0  0  0  0  0
   -0.9969   -0.4913    0.0000 S   0  0  0  0  0  0  0  0  0  0  0  0
   -0.9510    0.5400    0.0000 S   0  0  0  0  0  0  0  0  0  0  0  0
    1.1344    0.5171    0.0000 S   0  0  0  0  0  0  0  0  0  0  0  0
    1.1573   -0.4684    0.0000 S   0  0  0  0  0  0  0  0  0  0  0  0
    0.1141    1.2504    0.0000 Cl  0  0  0  0  0  0  0  0  0  0  0  0
    0.1141   -1.2590    0.0000 Cl  0  0  0  0  0  0  0  0  0  0  0  0
    1.8677    0.5400    0.0000 C   0  0  0  0  0  0  0  0  0  0  0  0
    1.8677   -0.4684    0.0000 C   0  0  0  0  0  0  0  0  0  0  0  0
    2.0969    0.0816    0.0000 C   0  0  0  0  0  0  0  0  0  0  0  0
   -1.7531   -0.4913    0.0000 C   0  0  0  0  0  0  0  0  0  0  0  0
   -1.7760    0.5400    0.0000 C   0  0  0  0  0  0  0  0  0  0  0  0
   -2.0969    0.0358    0.0000 C   0  0  0  0  0  0  0  0  0  0  0  0
    1.1573   -1.2934    0.0000 O   0  0  0  0  0  0  0  0  0  0  0  0
    1.8438   -0.9830    0.0000 C   0  0  0  0  0  0  0  0  0  0  0  0
    1.7177    1.1004    0.0000 O   0  0  0  0  0  0  0  0  0  0  0  0
    1.0927    1.3139    0.0000 C   0  0  0  0  0  0  0  0  0  0  0  0
   -1.4094   -1.2058    0.0000 C   0  0  0  0  0  0  0  0  0  0  0  0
   -1.3635    1.2544    0.0000 O   0  0  0  0  0  0  0  0  0  0  0  0
   -0.7375    1.3369    0.0000 C   0  0  0  0  0  0  0  0  0  0  0  0
   -0.7833   -1.2882    0.0000 O   0  0  0  0  0  0  0  0  0  0  0  0
```

-0.9969 -1.9202 0.0000 C 0 0 0 0 0 0 0 0 0 0 0 0
1.7453 -1.8021 0.0000 C 0 0 0 0 0 0 0 0 0 0 0 0
1.7847 1.7632 0.0000 C 0 0 0 0 0 0 0 0 0 0 0 0
-1.3209 1.9202 0.0000 C 0 0 0 0 0 0 0 0 0 0 0 0
1 2 1 1
1 3 1 6
1 4 1 6
1 5 1 1
1 6 1 0
1 7 1 0
4 8 1 0
5 9 1 0
9 10 1 0
10 8 1 0
2 11 1 0
3 12 1 0
12 13 1 0
13 11 1 0
5 14 1 1
5 15 1 6
4 16 1 6
4 17 1 1
2 18 1 1
3 19 1 1
3 20 1 6
2 21 1 6
18 22 1 0
15 23 1 0
17 24 1 0
20 25 1 0
M END

Compound **38(*S*S,*S*S):**

ChemDraw07102422422D

```
52 60  0  0  1  0  0  0  0  0999 V2000
  -1.5237    0.0838    0.0000 Ru  0  0  0  0  0  0  0  0  0  0  0  0
  -2.4258    0.6000    0.0000 S   0  0  0  0  0  0  0  0  0  0  0  0
  -1.5237    1.1494    0.0000 S   0  0  0  0  0  0  0  0  0  0  0  0
  -1.4925   -1.1016    0.0000 S   0  0  0  0  0  0  0  0  0  0  0  0
  -2.7485    1.5022    0.0000 C   0  0  0  0  0  0  0  0  0  0  0  0
  -2.1768    1.8052    0.0000 C   0  0  0  0  0  0  0  0  0  0  0  0
  -2.1793   -1.5587    0.0000 O   0  0  0  0  0  0  0  0  0  0  0  0
  -1.6549   -1.9104    0.0000 C   0  0  0  0  0  0  0  0  0  0  0  0
  -2.4366   -2.1742    0.0000 C   0  0  0  0  0  0  0  0  0  0  0  0
  -2.5990   -2.9831    0.0000 C   0  0  0  0  0  0  0  0  0  0  0  0
  -1.9797   -3.5281    0.0000 C   0  0  0  0  0  0  0  0  0  0  0  0
  -1.1980   -3.2643    0.0000 C   0  0  0  0  0  0  0  0  0  0  0  0
  -1.0356   -2.4555    0.0000 C   0  0  0  0  0  0  0  0  0  0  0  0
  -2.1421   -4.3370    0.0000 C   0  0  0  0  0  0  0  0  0  0  0  0
  -1.7372    1.9463    0.0000 C   0  0  0  0  0  0  0  0  0  0  0  0
  -1.3102    1.9463    0.0000 O   0  0  0  0  0  0  0  0  0  0  0  0
  -1.1539    2.5297    0.0000 C   0  0  0  0  0  0  0  0  0  0  0  0
  -1.3674    3.3266    0.0000 C   0  0  0  0  0  0  0  0  0  0  0  0
  -2.1643    3.5401    0.0000 C   0  0  0  0  0  0  0  0  0  0  0  0
  -2.7477    2.9567    0.0000 C   0  0  0  0  0  0  0  0  0  0  0  0
  -2.5341    2.1599    0.0000 C   0  0  0  0  0  0  0  0  0  0  0  0
  -2.3778    4.3370    0.0000 C   0  0  0  0  0  0  0  0  0  0  0  0
   0.5560    0.0322    0.0000 Ru  0  0  0  0  0  0  0  0  0  0  0  0
   0.5814    0.9781    0.0000 S   0  0  0  0  0  0  0  0  0  0  0  0
   1.4960   -0.5636    0.0000 S   0  0  0  0  0  0  0  0  0  0  0  0
   1.1648    1.5615    0.0000 C   0  0  0  0  0  0  0  0  0  0  0  0
```

0.5814 1.8031 0.0000 O 0 0 0 0 0 0 0 0 0 0 0 0
0.5560 -1.0334 0.0000 S 0 0 0 0 0 0 0 0 0 0 0 0
1.1523 -1.6407 0.0000 C 0 0 0 0 0 0 0 0 0 0 0 0
1.7252 -1.3657 0.0000 C 0 0 0 0 0 0 0 0 0 0 0 0
0.5560 -1.8584 0.0000 C 0 0 0 0 0 0 0 0 0 0 0 0
-0.0274 -1.6168 0.0000 O 0 0 0 0 0 0 0 0 0 0 0 0
-0.1585 -2.2709 0.0000 C 0 0 0 0 0 0 0 0 0 0 0 0
-0.1585 -3.0959 0.0000 C 0 0 0 0 0 0 0 0 0 0 0 0
0.5560 -3.5084 0.0000 C 0 0 0 0 0 0 0 0 0 0 0 0
1.2704 -3.0959 0.0000 C 0 0 0 0 0 0 0 0 0 0 0 0
1.2704 -2.2709 0.0000 C 0 0 0 0 0 0 0 0 0 0 0 0
0.5560 -4.3334 0.0000 C 0 0 0 0 0 0 0 0 0 0 0 0
1.9617 1.3480 0.0000 C 0 0 0 0 0 0 0 0 0 0 0 0
2.5451 1.9313 0.0000 C 0 0 0 0 0 0 0 0 0 0 0 0
2.3315 2.7282 0.0000 C 0 0 0 0 0 0 0 0 0 0 0 0
1.5346 2.9418 0.0000 C 0 0 0 0 0 0 0 0 0 0 0 0
0.9513 2.3584 0.0000 C 0 0 0 0 0 0 0 0 0 0 0 0
2.9149 3.3116 0.0000 C 0 0 0 0 0 0 0 0 0 0 0 0
1.9929 0.7713 0.0000 Cl 0 0 0 0 0 0 0 0 0 0 0 0
1.3783 0.7646 0.0000 C 0 0 0 0 0 0 0 0 0 0 0 0
1.7049 0.1533 0.0000 C 0 0 0 0 0 0 0 0 0 0 0 0
-2.9149 -0.7918 0.0000 Cl 0 0 0 0 0 0 0 0 0 0 0 0
-0.5171 -0.5694 0.0000 Cl 0 0 0 0 0 0 0 0 0 0 0 0
-2.6492 -0.1660 0.0000 C 0 0 0 0 0 0 0 0 0 0 0 0
-2.3065 -0.8525 0.0000 C 0 0 0 0 0 0 0 0 0 0 0 0
-0.5514 0.6510 0.0000 Cl 0 0 0 0 0 0 0 0 0 0 0 0
1 2 1 6
1 3 1 0
2 5 1 6
5 6 1 0
6 3 1 0

4 7 1 1

4 8 1 6

8 9 2 0

9 10 1 0

10 11 2 0

11 12 1 0

12 13 2 0

13 8 1 0

11 14 1 0

3 15 1 1

3 16 1 6

15 17 2 0

17 18 1 0

18 19 2 0

19 20 1 0

20 21 2 0

21 15 1 0

19 22 1 0

23 25 1 1

24 26 1 6

24 27 1 1

23 28 1 0

28 29 1 0

29 30 1 0

25 30 1 1

28 31 1 1

28 32 1 6

31 33 2 0

33 34 1 0

34 35 2 0

35 36 1 0

36 37 2 0

37 31 1 0

35 38 1 0

26 39 2 0

39 40 1 0

40 41 2 0

41 42 1 0

42 43 2 0

43 26 1 0

41 44 1 0

23 24 1 0

23 45 1 6

24 46 1 0

46 47 1 0

47 25 1 0

1 4 1 0

1 49 1 1

2 50 1 0

50 51 1 0

51 4 1 0

1 52 1 6

23 52 1 6

23 49 1 1

1 48 1 1

M END

Compound **39(*S*S,*S*S):**

ChemDraw07102422432D

29 32 0 0 1 0 0 0 0 0 0999 V2000

0.5705   -0.2281   0.0000 Ru 0 0 0 0 0 0 0 0 0 0 0 0

-0.2310 -1.0238 0.0000 S 0 0 0 0 0 0 0 0 0 0 0 0
1.3351 0.3913 0.0000 S 0 0 0 0 0 0 0 0 0 0 0 0
1.4060 -1.0238 0.0000 S 0 0 0 0 0 0 0 0 0 0 0 0
0.5705 0.8376 0.0000 Cl 0 0 0 0 0 0 0 0 0 0 0 0
0.6049 -1.4313 0.0000 Cl 0 0 0 0 0 0 0 0 0 0 0 0
1.0779 -1.9925 0.0000 C 0 0 0 0 0 0 0 0 0 0 0 0
1.9904 -1.7382 0.0000 O 0 0 0 0 0 0 0 0 0 0 0 0
1.9184 0.9746 0.0000 O 0 0 0 0 0 0 0 0 0 0 0 0
-0.7467 -1.7113 0.0000 C 0 0 0 0 0 0 0 0 0 0 0 0
-1.0560 -1.0238 0.0000 O 0 0 0 0 0 0 0 0 0 0 0 0
2.2317 -0.6712 0.0000 C 0 0 0 0 0 0 0 0 0 0 0 0
2.2247 0.0558 0.0000 C 0 0 0 0 0 0 0 0 0 0 0 0
0.1314 -1.9925 0.0000 C 0 0 0 0 0 0 0 0 0 0 0 0
1.3351 1.2163 0.0000 C 0 0 0 0 0 0 0 0 0 0 0 0
-1.5658 -1.6126 0.0000 C 0 0 0 0 0 0 0 0 0 0 0 0
-2.0608 -2.2726 0.0000 C 0 0 0 0 0 0 0 0 0 0 0 0
-1.7367 -3.0313 0.0000 C 0 0 0 0 0 0 0 0 0 0 0 0
-0.9176 -3.1299 0.0000 C 0 0 0 0 0 0 0 0 0 0 0 0
-0.4226 -2.4699 0.0000 C 0 0 0 0 0 0 0 0 0 0 0 0
-2.2317 -3.6913 0.0000 C 0 0 0 0 0 0 0 0 0 0 0 0
2.0495 1.6288 0.0000 C 0 0 0 0 0 0 0 0 0 0 0 0
2.0495 2.4538 0.0000 C 0 0 0 0 0 0 0 0 0 0 0 0
1.3351 2.8663 0.0000 C 0 0 0 0 0 0 0 0 0 0 0 0
0.6206 2.4538 0.0000 C 0 0 0 0 0 0 0 0 0 0 0 0
0.6206 1.6288 0.0000 C 0 0 0 0 0 0 0 0 0 0 0 0
1.3351 3.6913 0.0000 C 0 0 0 0 0 0 0 0 0 0 0 0
-0.1940 0.3913 0.0000 O 0 0 0 0 0 0 0 0 0 0 0 0
-0.9616 0.0968 0.0000 C 0 0 0 0 0 0 0 0 0 0 0 0
28 29 1 0
1 28 1 6
1 2 1 1

1 3 1 6
1 4 1 1
1 5 1 0
1 6 1 0
4 7 1 1
4 8 1 6
3 9 1 1
2 10 1 1
2 11 1 6
4 12 1 0
12 13 1 0
13 3 1 0
2 14 1 0
14 7 1 0
3 15 1 6
10 16 2 0
16 17 1 0
17 18 2 0
18 19 1 0
19 20 2 0
20 10 1 0
18 21 1 0
15 22 2 0
22 23 1 0
23 24 2 0
24 25 1 0
25 26 2 0
26 15 1 0
24 27 1 0
M STY 1 1 SUP
M SLB 1 1 1

M SAL   1 2 28 29

M SBL   1 1   2

M SMT   1 HOMe

M SBV   1   2   0.7645   -0.6194

M END
